# A systems biology network analysis of nutri(epi)genomic changes in endothelial cells exposed to epicatechin metabolites

**DOI:** 10.1038/s41598-018-33959-x

**Published:** 2018-10-19

**Authors:** Dragan Milenkovic, Wim Vanden Berghe, Christine Morand, Sylvain Claude, Annette van de Sandt, Simone Gorressen, Laurent-Emmanuel Monfoulet, Chandra S. Chirumamilla, Ken Declerck, Katarzyna Szarc vel Szic, Maija Lahtela-Kakkonen, Clarissa Gerhauser, Marc W. Merx, Malte Kelm

**Affiliations:** 1 INRA, UMR 1019, UNH, CRNH Auvergne, F-63000 Clermont-Ferrand; Clermont Université, Université d’Auvergne, Unité de Nutrition Humaine, BP 10448, F-63000 Clermont-Ferrand, France; 2 Department of Internal Medicine, Division of Cardiovascular Medicine, School of Medicine, University of California Davis, Davis, California, 95616 United States of America; 3 PPES, Department of Biomedical Sciences, University of Antwerp (UA), Wilrijk, Belgium; 4 L-GEST, Department of Biochemistry and Microbiology, University of Gent, Ghent, Belgium; 5 Division of Cardiology, Pulmonology, and Vascular Medicine, Medical Faculty, University Hospital Düsseldorf, Düsseldorf, Germany; 6 School of Pharmacy, University of Eastern Finland, Kuopio, Finland; 7 Division of Epigenomics and Cancer Risk Factors, DKFZ, Heidelberg, Germany; 8 Present Address: KRH Klinikum Robert Koch Gehrden, Department of Cardiovascular Diseases and Internal Intensive Medicine, Von-Reden-Straße 1, 30989 Gehrden, Germany

## Abstract

Although vasculo-protective effects of flavan-3-ols are widely accepted today, their impact on endothelial cell functions and molecular mechanisms of action involved is not completely understood. The aim of this study was to characterize the potential endothelium-protective effects of circulating epicatechin metabolites and to define underlying mechanisms of action by an integrated systems biology approach. Reduced leukocyte rolling over vascular endothelium was observed following epicatechin supplementation in a mouse model of inflammation. Integrative pathway analysis of transcriptome, miRNome and epigenome profiles of endothelial cells exposed to epicatechin metabolites revealed that by acting at these different levels of regulation, metabolites affect cellular pathways involved in endothelial permeability and interaction with immune cells. *In-vitro* experiments on endothelial cells confirmed that epicatechin metabolites reduce monocyte adhesion and their transendothelial migration. Altogether, our *in-vivo* and *in-vitro* results support the outcome of a systems biology based network analysis which suggests that epicatechin metabolites mediate their vasculoprotective effects through dynamic regulation of endothelial cell monocyte adhesion and permeability. This study illustrates complex and multimodal mechanisms of action by which epicatechin modulate endothelial cell integrity.

## Introduction

Flavan-3-ols represent a major group of flavonoids found in the Western diets and include monomeric, oligomeric and polymeric forms of catechin (C) and epicatechin (EC). These compounds are found in most foods and are particularly abundant in cocoa, green tea, red wine and various fruits. A recent systematic review of prospective cohort studies has reported that consumption of flavonoids is inversely associated with the risk of CVD when comparing the highest and lowest categories of intake^1^. Together with these epidemiological data, accumulating clinical evidence from acute and chronic intervention studies with flavan-3-ol-rich cocoa/chocolate indicates that there are significant improvements in some intermediate biomarkers associated with cardiovascular risk^2,3^. Data are particularly persuasive regarding the improvement of flow-mediated vasodilation (FMD), which measures endothelial function^4–7^. This beneficial effect has been causally linked to flavan-3-ol monomers in flavanol-rich cocoa^8^. In a mouse model of atherosclerosis, supplementation of the diet with a flavan-3-ol monomer has been shown to reduce lipid deposits in the aortic roots and to induce changes in aortic gene expression profiles^9^. Many of the observed gene expression changes were involved in controlling the early steps of vascular dysfunction and the development of atherosclerosis.

Even though there is a plethora of convincing *in vivo* evidence regarding the vasculoprotective effects of flavan-3-ols, the mechanisms by which these compounds exert their effects are not fully understood. *In vitro* studies aiming to identify these underlying mechanism(s) of action present several limitations. Firstly, most *in vitro* studies use high concentrations of parent compounds or extracts rather than physiologically relevant concentrations of circulating plasma metabolites. For cocoa flavan-3-ols, these circulating plasma metabolites consist predominantly of glucuronide, sulfate and methyl sulfate derivatives of EC^10,11^. Secondly, *in vitro* mechanistic studies frequently use candidate approaches that are not appropriate to fully consider the multi-target modes of action of these compounds^12^.

We have previously shown that the exposure of endothelial cells to individual sulfate, glucuronide and methyl-glucuronide derivatives of EC, used in a physiological range of concentrations, decreased monocyte adhesion to TNFα-activated endothelial cells^13^. This effect was observed together with the ability of these EC metabolites to modulate endothelial expression of a large set of genes that are involved in cell processes regulating monocyte adhesion and transmigration across the vascular wall. Recent nutrigenomic studies have also shown that polyphenols can regulate the expression of microRNAs (miRNAs)^14^. These non-coding small RNAs are post-transcriptional regulators of gene expression and may be key regulators of the cardiovascular system^15^. Nevertheless, the impact of flavan-3-ols on miRNA expression is still largely unknown. DNA methylation is a major epigenetic process which controls gene and microRNA transcription through changes in chromatin architecture. Alterations in DNA methylation have been reported to be causally involved in the development of several human diseases, including cardiovascular diseases^16,17^. The ability of polyphenols to induce epigenetic changes has been recently highlighted^18,19^. In particular, cocoa flavan-3-ols have been shown to modulate DNA methylation of peripheral leukocytes in humans^20–22^.

The aims of this study were to provide molecular biological evidence of the vasculo-protective effect of plasma EC metabolites by evaluating their effect on cellular processes involved in the initial steps of vascular dysfunction and atherosclerosis development, and to decipher the underlying mechanisms of action using a systems biology approach. To study the effect of EC on the interaction between immune cells and vascular endothelial cells *in vivo*, a dietary supplementation study with EC was performed in a mouse model of inflammation. Using a combination of transcriptomics, epigenomics and miRNomics, the associated mechanisms of action were examined *in vitro* using endothelial cells exposed to a mixture of plasma EC metabolites at physiologically-relevant concentrations. Hypotheses built from these systems biology analyses were then validated using *in vitro* assays of the cellular processes revealed as modulated by EC metabolites, namely monocyte adhesion and their transendothelial migration.

## Materials and Methods

### Microcirculation mouse model - Intravital fluorescence microcopy

Male C57BL/6 wild type (WT) mice were kept according to federal regulations. All experiments on animals were performed in accordance with the national guidelines on animal care and were approved by the local Research Board for animal experimentation (LANUV = State Agency for Nature, Environment and Consumer Protection, # 84-02.04.2011.A235). Mice ranged in body weight from 20–25 g and in age from 10–14 weeks. Animals received a semi-synthetic diet (Supplemental Fig. S1A) and water *ad libitum*.

Mice, 5 per group, were given either epicatechin-free diet or diet supplemented with epicatechin at 0.06 g/kg of diet *ad libitum* over a period of 7 days (Supplemental Fig. S1B) and subsequently starved for 4 hours before sepsis induction by cecum ligation and puncture (CLP) as described in the supplemental materiel. During the onset of sepsis, mice were given an epicatechin-free diet over a period of 12 h. After 12 h, microcirculation was determined.

For intravital fluorescence microscopy we used the dorsal skin fold chamber preparation which contains one layer of striated muscle and skin and allows the *in-vivo* observation of the microcirculation in the awake animal over a prolonged time^23,24^. The model permits the determination of leukocyte/endothelial cell interaction (rolling and adherent leukocytes) and the quantification of micro hemodynamic parameters, including functional capillary density, red blood cell velocity and evaluation of diameters. For more details please see Supplemental Method Note.

### Flavan-3-ols

Purified epicatechin metabolites: 3′-*O*-methyl(-)-epicatechin (3′MEC), 4′-*O*-methyl(-)-epicatechin-7-β-D-glucuronide (4′MEC7G) and (-)-epicatechin-4′-sulfate (EC4′S) were gifted by Mars Inc (McLean, VA, USA). Chemical structures are presented in the Supplemental Fig. S2. Conjugated metabolites were dissolved in 50% ethanol in double distilled water. TNFα was obtained from R&D Systems (Lille, France) and was dissolved in 0.1% BSA/PBS (Pan Biotech, Aidenbach, Germany) to obtain a stock solution at 100 µg/ml. MCP-1 was obtained from clinisciences (Nanterre, France).

### Cell culture conditions

Human umbilical vein endothelial cells (HUVEC) (Lonza, Walkersville, MD, USA), a pool from four donors, at passage 3 were cultured in a endothelial growth medium (EGM) that is phenol-free and supplemented with 2% fetal bovine serum (FBS), 0.4% fibroblast growth factor, 0.1% vascular endothelial growth factor, 0.1% heparin, 0.1% insulin-like growth factor, 0.1% ascorbic acid, 0.1% epidermal growth factor and 0.04% hydrocortisone (all from Lonza). A human monocytic cell line (THP-1) was obtained from American Type Culture Collection (ATCC, Manassas, VA, USA) and cultured in the RPMI medium (Pan Biotech, Aidenbach, Germany) supplemented with 2% FBS (Sigma, Saint-Quentin Fallavier, France).

HUVECs, at passage 4, were seeded into the 24-well plates (BD-Falcon, Le Pont-De-Claix, France) and grown to reach 60–70% confluence. The cell culture medium was then substituted to expose the cells for 3 h to the cell culture medium including either solvent alone (ethanol 0.5‰, control wells, vehicle) or mixture of metabolites (3′MEC, 4′MEC7G and EC4′S) at 1 µM each. After the 3-hours period, the medium was substituted with medium without flavanols and left for another 18 hours until confluence. The confluent single layer of endothelial cells was stimulated for 4 hours with TNFα (R&D Systems Lille, France) at 0.1 ng/mL or only incubated with PBS/BSA (0,01‰, negative control).

### Total RNA extraction

After stimulation with TNFα for 4 hours, HUVECs monolayers from 4 replicates were rinsed twice with cold Phosphate Buffered Saline (PBS) before cell lysis using Lysing Buffer solution from the RNeasy Micro Kit (Qiagen, Hilden, Germany). Total RNA extraction, including small RNAs, was performed as recommended by the manufacturer. The quality and quantity of RNA were checked by 1% agarose gel electrophoresis and using the absorbencies at 260 and 280 nm on NanoDrop ND-1000 spectrophotometer (Thermo Scientific, Wilmington, DE, USA). The total RNA samples were stored at −80 °C until used.

### Microarray analysis

Total RNA (50 ng per sample) for 12 RNA samples (4 replicates per condition) was amplified and fluorescently labelled to produce Cy5 or Cy3 cRNA using the Low Input Quick Amp Labeling two color Kit (Agilent, Santa Clara, USA) in the presence of spike-in two colors control as recommended by the manufacturer. After purification, 825 ng of labeled cRNA were hybridized onto G4845A Human GE 4 × 44 K v2 microarray (Agilent, USA) according to the manufacturer’s instructions. The G4845A Human GE 4 × 44 K v2 microarray contains 27958 Entrez Gene RNAs sequences. Following hybridization, Agilent G2505 scanner (Agilent, USA) was used to scan microarrays and data were extracted using Feature Extraction software (Agilent, USA) using linear and Lowess normalization. Statistical analyses were done using R 2.1 software (http://www.r-project.org) after controlling the variance of genes. Data were analyzed with a Student’s t test to identify differentially expressed genes and values were adjusted using the Benjamini-Hochberg correction at 1% aiming to eliminate false positives. Genes obtained were referred to as the “differentially expressed genes”.

### miRNA expression analysis

The impact of epicatechin metabolites on the expression of miRNAs was analyzed using Human miRNA (V3) 8 × 15 K microarrays (Agilent, Santa Clara, USA) covering 866 mature miRNAs. MiRNAs (n = 12) were labeled using miRNA labeling and hybridization kit from Agilent technologies (Agilent, Santa Clara, USA) as recommended by the manufacturer. Briefly, 100 ng of each total RNA sample were treated with calf intestinal phosphatase for 30 min at 37 °C before denaturing the samples using pure DMSO at 100 °C for 5 min and rapid transfer in an ice water bath to prevent RNA reannealing. RNA samples were labeled with pCp-Cy3 using T4 RNA ligase by incubation at 16 °C for 2 h. After purification with microBioSpin columns, labeled samples were hybridized to Agilent human miRNA microarrays, which contain probes for human microRNAs from the Sanger database. Hybridizations were performed for 24 h at 55 °C after which the microarrays were washed in GE Wash Buffer 1 and GE Wash Buffer 2 for 5 min. Following washing step, the microarrays were scanned with Aligent Microarray Scanner (Agilent, Santa Clara, CA, USA). The scanned images were analyzed using Feature Extraction Software (Agilent, Santa Clara, CA, USA). Genespring GX10 software (Agilent, Santa Clara, CA, USA) was used to quantify the signal and background intensity for each feature and to substantially normalize the data by the 75th percentile method. Statistical analyses were done using R 2.1 software (http://www.r-project.org) after verifying the variance of genes. Data were analyzed with a Student’s t test to detect differentially expressed miRNAs and the p-values were adjusted by using the Benjamini–Hochberg correction to eliminate false positives. miRNAs selected by these criteria are referred to as the “differentially expressed miRNAs”.

### Western blot

Proteins were prepared from endothelial cells using lysis buffer containing 150 mM NaCl, 10 mM Tris HCl, 1 mM EGTA, 1 mM EDTA, 100 mM fluoride sodium, 4 mM pyrophosphate sodium, 2 mM orthovanadate sodium, 1% Triton X100, 0.5% octylphenoxypolyethoxyethanol (IGEPAL) CA-630, all from Sigma (St Quentin Fallavier, France).

After washing with PBS, the cells were lysed using the lysis buffer supplemented with the protease inhibitor cocktail (Sigma, St Quentin Fallavier, France). The lysate was kept at −80 °C overnight. Following thawing and centrifugation at 15.000 × g during 30 min at 4 °C, protein concentrations in the supernatant were evaluated using the BCA Protein Assay Kit (Interchim, Montluçon, France) as suggested by the manufacturer. 10 µg of total proteins were electrophoretically separated on 8% polyacrylamide gels and transferred for 1 h on to nitrocellulose membranes Genetex (Euromedex, Mundolsheim, France). The membranes were then blocked for 1 h with 5% BSA Tris-buffered saline/0.1% Tween 20. Following washing with Tris-buffered saline/0.1% Tween 20, the membranes were incubated with primary antibodies against p38 MAPK (1/1000), Phospho p38 MAPK (Thr180/Tyr182) (1/1000), all from Cell Signaling (Ozyme, Saint Quentin Yvelines, France) and Actin Beta (1/10000) from Genetex (Euromedex, Mundolsheim, France). The membranes were incubated with secondary fluorescent goat anti-rabbit and goat anti-mouse IgG (H + L) antibodies (1/20000) from Genetex (Euromedex, Mundolsheim, France). Proteins were visualized by Odyssey Li-Cor (Lincoln, Nebraska, USA) detection system and density of the signals was quantified using Odyssey software. Samples were normalized to β Actin housekeeping protein. The expression of β Actin was used as the control for protein loading.

### Docking metabolites/protein interaction analyses

Human p38α MAPK crystal structure 4F9Y^25^ was retrieved from the RCSB Protein Data Bank (PDB). The retrieved structure of P38MAPK was preprocessed in Schrödinger maestro, which includes assignment of partial charges, addition of missing atoms and chain residues. Restrained energy minimization was performed by OPLS5 force field to obtain stable structure for docking. Ligand structure of three epicatechin structures: 3′-O-Methyl Epicatechin (3-MEC), 4-O-methyl epicatechin 7-beta-glucuronate (4MEG) and Epicatechin 4-sulfate (EC4S), were prepared using the Ligand Preparation Wizard in Maestro. The OPLS5 force field was performed, the ionization states and tautomers of these molecules were generated^26^. Regardless of the crystallized ligand based binding location, the active site of the p38MAPK was performed by SiteMap tool of glide using default parameters (charge cutoff 0.25, Vander der Waals scaling factor 1.0). The amino acids in the ligand binding region were identified by SiteMap acids (Supplementary Table S1). We used the top predicted binding site for carrying out the molecular docking. We performed initial molecular docking with Glide using standard precision followed by extra precision (XP) to predict the binding mode^27^. Post docking minimization with OPLS5 force field was also applied. After docking, top ranked compounds were arranged based on the glide score, with more negative glide scores representing a more favorable binding. Three-dimensional molecular interactions of the docked ligands in p38MAPK were displayed, by using pymol, arbitrary distances from the active sites of ligands with top binding mode towards the specific cysteines were calculated by distance measurement wizard in PyMol (https://www.pymol.org).

### DNA-methylation arrays

Genomic DNA (gDNA) from HUVEC was extracted using DNeasy kit (Qiagen, Courtaboeuf, France). DNA purity and concentrations were determined by UV-VIS spectrophotometry (NanoDrop, Thermo Fisher Scientific Inc, Wilmington, DE, USA) and stored at −80 °C until further use. Bisulfite converted DNA from the HUVECs was hybridized to the Illumina HumanMethylation450 BeadChip arrays (Illumina, San Diego, CA, USA). Briefly, for each sample, 1 μg of genomic DNA was bisulfite-converted using an EZ DNA methylation Kit (ZYMO research, Irvine, CA, USA) according to the manufacturer’s recommendations. Converted gDNA was eluted in 22 μl of elution buffer. DNA methylation level was measured using the Illumina Infinium HD Methylation Assay (Illumina, San Diego, CA, USA) according to the manufacturer’s instructions. Briefly, 4 μl of bisulfite-converted DNA (±150 ng) was isothermally amplified overnight (20–24 h) and fragmented enzymatically. Precipitated DNA was resuspended in hybridization buffer and dispensed onto the Infinium HumanMethylation450 BeadChips (12 samples/chip) using a Freedom EVO robot (Tecan, Männedorf, Switzerland). The hybridization procedure was performed at 48 °C overnight (16–20 h) using an Illumina Hybridization oven. After hybridization, free DNA was washed away and the BeadChips were processed through a single nucleotide extension followed by fluorescent readout of the incorporated base using a Freedom EVO robot. Finally, the BeadChips were imaged using an Illumina iScan (Illumina, San Diego, CA, USA). Detection p-values were calculated to identify failed probes as per Illumina’s recommendations. No arrays exceeded our quality threshold of >5% failed probes. Filtering of bad quality probes and normalization of raw methylation beta values was conducted using RnBeads package in R software^28^. Probes with detection p-values higher than 0.01, overlapping with SNPs at the last 3 bases in its sequence or containing missing values were excluded. Beta mixture quantile dilation (BMIQ) was used to normalize between the two different probe designs (Infinium I and Infinium II)^29^. Differentially methylated probes were identified using limma R package. Probes with a Benjamini-Hochberg adjusted p-value below 0.1 and a methylation difference of at least 10% were defined as differentially methylated probes (DMPs). Probes were assigned to genes, CpG island annotations (CpG island, CGI shores, CGI shelves, and open sea) and gene regions (TSS1500, TSS200, 5′UTR, 1st exon, gene body and 3′UTR) based on the HumanMethylation450 v1.2 Manifest file from Illumina. The online software tool EpiExplorer was used to identify enrichment of DMPs in different genomic regions (such as promoters, enhancers or chromatin marks)^30^. Differentially methylated regions (DMRs) were identified using the DMRcate R package. Significant DMRs (FDR 5%) must contain at least 3 Illumina probes whereof at least one CpG have a DNA methylation difference of more than 10%.

### Bioinformatics analysis

Bioinformatics analyses were done using different softwares. For canonical pathway analysis, KEGG (Kyoto Encyclopedia of Genes and Genomes) (http://www.genome.jp/kegg/tool/map_pathway2.html)^31,32^ and Metacore software packages Metacore™ (GeneGo, St Joseph, MO, USA; https://portal.genego.com) were used to identify significantly overrepresented pathways using. Enrichment statistics were calculated for these data sets assuming a hypergeometric distribution to identify significantly overrepresented pathways. Gene Ontology (GO), gene network by data-mining and transcription factor analysis was also done using Metacore™. MicroRNA validated targets and target prediction was searched using the miRWalk database^33^ that enables retrieval of genes predicted by different algorithms including Diana-microT, miRANDA, miRDB, miRWalk, RNAhybrid, PICTAR5, PITA, RNA22 and TargetScan. In this work, only target genes predicted by five or more algorithms were included in order to improve specificity. Venn diagrams were obtained using BioVenn (http://www.cmbi.ru.nl/cdd/biovenn) and Venny (http://bioinfogp.cnb.csic.es/tools/venny/index.html).

### Monocyte adhesion assay

The assay of monocytes adhesion to endothelial cells was performed as previously described^13^. Briefly, to TNFα-activated HUVECs obtained as described in the section 2.2, 50 µL of a 5 × 10^6^ THP-1/mL cell suspension was added and cells were further co-incubated for 1 hour. Non-adhering THP-1 cells were washed away using PBS and the cells were fixed using crystal violet (Sigma, Saint-Quentin Fallavier, France) 0.5% in ethanol. The amount of adherent THP-1 cells was counted in three random microscopic surfaces defined by an eyepiece for each well. Triplicates for each condition were done in three different experiments.

### Transendothelial migration

HUVECs, 15.000 cells at passage 4, were seeded on 6.5-mm Transwell® gelatin-coated insert with pores of 5 µm in diameter (Corning, Corning, NY, USA). At confluence, the medium in the upper chamber was substituted to expose HUVECs for 3 h period to a medium containing either vehicle alone (ethanol 0.5‰) or mixture of metabolites (3′MEC, 4′MEC7G and EC4′S) at 1 µM. After the 3 h incubation period, the medium was substituted with medium that does not contain flavanol metabolites and left for additional 18 hours. After the 18 h period, 50.000 THP-1 cells in 100 µl final volume were added to the upper chamber and 50 ng/mL final concentration of monocyte chemotactic protein 1 (MCP-1) in the lower chamber. HUVECs and THP-1 were co-incubated for 3 h at 37 °C to allow cells to transmigrate. Transmigrated THP-1 cells in the lower chamber were counted using the coulter Z1 particle counter (Beckman, USA). The percentage of cells that transmigrated in the presence of epicatechin mixture was compared to the percentage of cells that transmigrated under the control condition (HUVECs exposed to vehicle alone).

### Statistical analyses

For the monocytes to endothelial cell adhesion assay, data were analyzed using two-way analysis of variance (ANOVA) and Tukey’s HSD (honest significant difference) test to reveal the effect of epicatechin metabolites. For Western blots modifications between means/positive controls were analyzed using the Student’s t-test. For transendothelial migration, data were analyzed using one-way analysis of variance (ANOVA) and Dunnett’s multiple comparisons test. p values lower than 0.05 was taken to imply statistical significance. Data obtained from *in-vivo* studies were analyzed using two-way ANOVA analysis with Bonferroni’s posthoc test.

## Results

### Epicatechin metabolites reduce leukocyte rolling *in vivo* upon vascular inflammation

**Figure 1:**
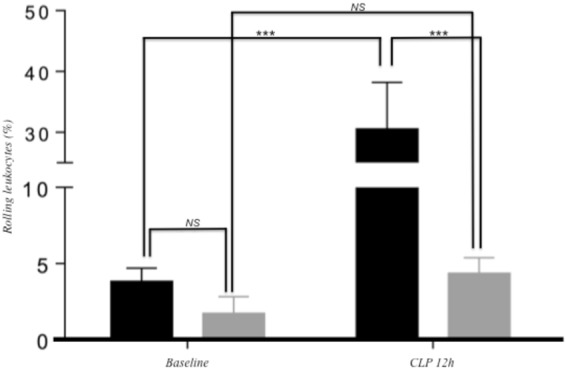
Microcirculatory measurements in the dorsal skinfold chamber in post-capilary venules at baseline and 12 h after sepsis induction in mice were treated with flavanol-free or flavanol rich diet (0.06%g/kg of diet) over a period of 7 days. In black: control diet; in grey: control diet +0.06 g of EC/kg of diet. (n = 5; mean +/− SEM; ***p < 0.01; 2-way ANOVA and Bonferroni’s posttest).

To obtain proof of concept for *in vivo* efficacy of flavan-3-ols, we evaluated the effect of a 7-day dietary supplementation with epicatechin, 0.06 g/kg of diet, on leukocyte rolling in a mouse model of vascular inflammation. Microcirculatory measurements in the dorsal skinfold chamber in postcapillary venules showed no difference in rolling of leukocytes between mice on control and on epicatechin-rich diet at baseline (3.82% ± 0.87 vs. 1.72% ± 1.09) (p < 0.01) (Fig. 1). Mice on control diet exhibited a significant increase in rolling leukocytes 12 h after CLP induction, 30.56% ± 7.65 **(**Fig. 1). This effect was not observed in mice on epicatechin-rich diet 12 h post CLP induction, 4,36% ± 1.02 **(**Fig. 1). A slight, but not significant, decrease of white blood cell count was observed in both groups 12 h after CLP induction compared to baseline (Supplemental Fig. S3).

Different parameters that could affect leukocyte rolling have also been measured. Twelve hours after CLP induction, venules diameter was elevated in both groups (control: 87.8 µm ± 8.9 vs. epicatechin-rich diet: 78.0 µm ± 12.4) compared to baseline (control: 70.2 µm ± 18.3 vs. epicatechin-rich diet: 54.2 µm ± 7.6) (Supplemental Fig. S4). Red blood cell velocity was significantly diminished 12 h post CLP induction in control mice (baseline: 1,12 mm/s ± 0.27 vs. 12 h CLP: 0.44 mm/s ± 0.04) compared to mice on epicatechin-rich diet (baseline: 0.83 mm/s ± 0.08 vs. 12 h CLP: 0.42 mm/s ± 0.11) (Supplemental Fig. S5), while red blood cell velocity did not differ between both groups (control: 0.44 mm/s ± 0.04 vs. epicatechin-rich diet: 0.42 mm/s ± 0.11) 12 h post CLP induction.

### Epicatechin metabolites modulate expression of gene in endothelial cells associated with cell adhesion and transendothelial migration

To assess the molecular mechanisms of action of epicatechin metabolites in vascular inflammation, we performed a pangenomic gene expression in endothelial cells, 20 h after exposure of the cells for 3 h to 1 μM of the mixture of the three metabolites of epicatechin: 3′-*O*-methyl(-)-epicatechin, 4′-*O*-methyl(-)-epicatechin-7-β-D-glucuronide and (-)-epicatechin-4′-sulfate prior to an inflammatory, TNFα, stimulus. First, the transcriptome analysis of TNFα-stimulated cells not pre-exposed to the metabolite mixture showed modifications in the expression of 4784 genes when compared to TNFα non-stimulated cells (Supplemental Table S2). The differentially expressed genes included those coding for cell adhesion proteins, such as ICAM1, VCAM1 and CXCL2, inflammation or chemotaxis. The observed changes in gene expression indicated a pro-inflammatory profile in TNFα-activated cells. Second, the exposure of endothelial cells to the mixture of the three epicatechin metabolites prior to their stimulation with TNFα modulated the expression of 253 genes (Supplemental Table S3) when compared to TNFα-activated cells non-exposed to epicatechin metabolites. Among these 253 genes, 112 genes were identified up-regulated and 141 genes were down-regulated by the epicatechin metabolites. Comparison of the 253 differentially expressed genes with the 4787 TNFα-modulated genes revealed an overlap of 66 genes. Interestingly, among these 66 common genes, 44 presented opposite gene expression profiles, suggesting that epicatechin metabolites could partially counteract the genomic effect induced by TNFα in endothelial cells.

**Figure 2:**
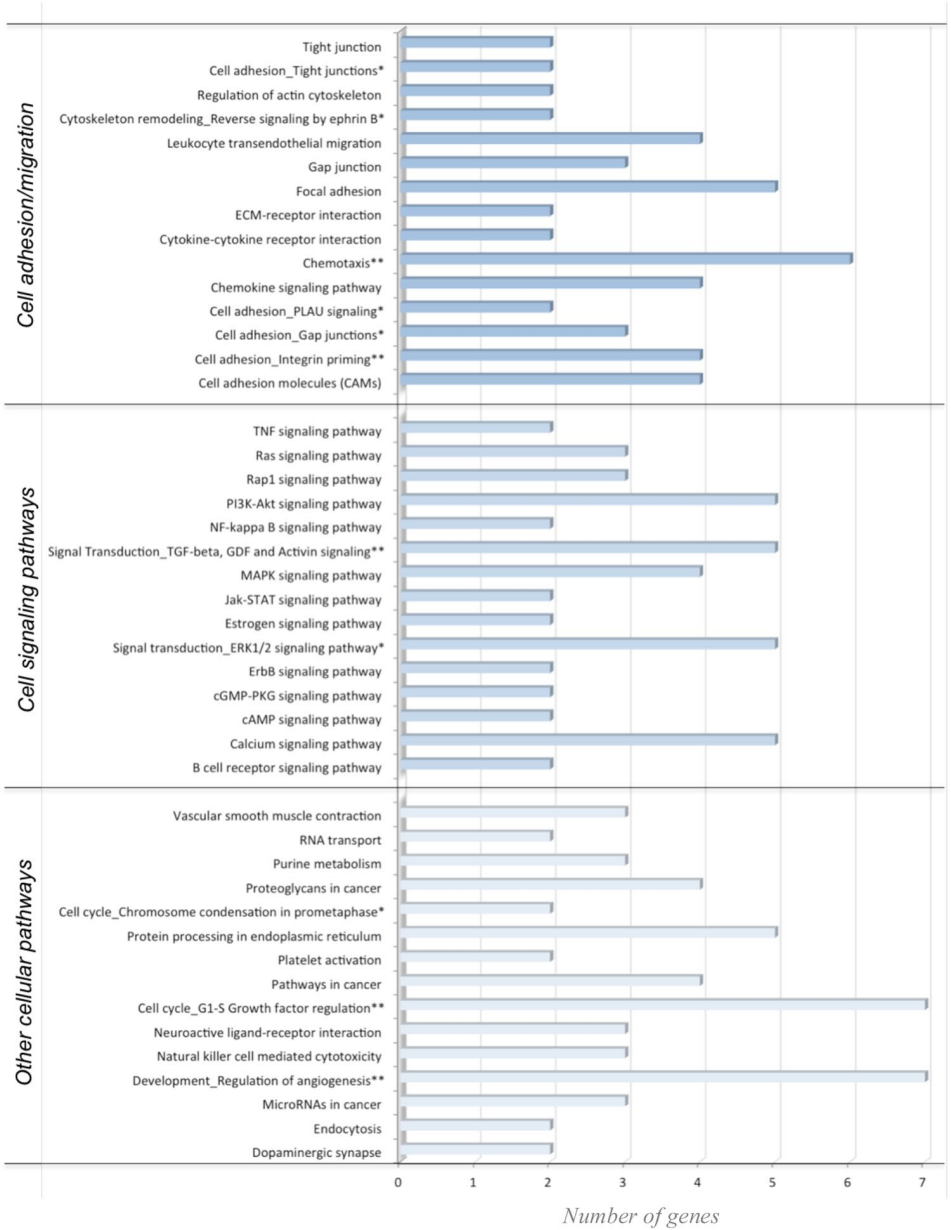
Pathways and networks enriched with differentially expressed genes in response to flavanol metabolites in HUVEC. Pathways identified from KEGG database are marked *and ene networks identified using Metacore are marked with**.

Bioinformatic analyses were performed to identify biological functions of the 253 genes which expression was modulated by the mixture of epicatechin metabolites. First, gene networks built with the data-mining approach using Metacore software suggested that the flavanol metabolites change the expression of genes involved in different biological processes. Among the most significant networks, we identified a group of genes (CCR9, PLAU or NCAM1) involved in the regulation of chemotaxis but also networks regulating cell adhesion, cell junctions and cellular cytoskeleton organization. Among the genes identified in these networks are VE-cadherin, CDX1, SOS1, CLD11 and MAP2. These genes encode proteins involved in the control of cellular junctional complexes that control endothelial cell integrity and permeability. To further refine biological functions of these differentially expressed genes in response to epicatechin metabolites, we performed the pathway analysis using KEGG and Metacore databases. Interestingly, similar to results obtained from network analysis, the most over-represented pathways were those involved in cell adhesion, leukocyte transendothelial migration, Ras signaling pathway, calcium signaling pathway, focal adhesion or gap junctions (Fig. 2). Among the genes identified in these pathways are PPP1R12B, SOS1, CLD11, CDH5, RASGRP3 and RAPGEF3. All these identified over-represented pathways are involved in regulation of adhesion and transmigration of leukocyte through the endothelium.

**Figure 3:**
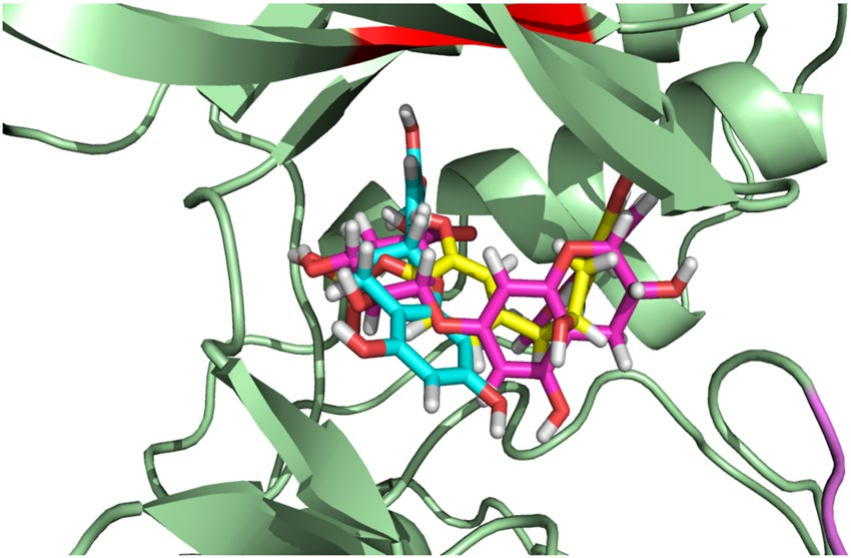
In silico docking of flavanol metabolites into ATP binding pocket of p38-MAPK. 3′-O-methyl(-)-epicatechin is presented in bleu; 4′-O-methyl(-)-epicatechin-7-β-D-glucuronide in yellow and (-)-epicatechin-4′-sulfate in pink.

We than used Transcription Regulation algorithms (Metacore software) to ascertain transcription factors which activity could be modulated by epicatechin metabolites and explain downstream gene expression changes. Among the most significant transcription factors identified were NF-κB, CREB1, cMyc, c-JUN, STAT3 or SP1. Different signaling proteins regulate these transcription factors, but they all can be regulated by p38-MAPK. Western-blot analysis showed that the mixture of metabolites decreased TNFα-induced phosphorylation of p38 by 15% (Supplemental Fig. S7). Subsequently, molecular docking analysis was used to investigate potential binding modes of (-)-epicatechin metabolites to this cell signaling protein. Interestingly, favorable binding of the three metabolites to the ATP binding cavity was reveiled **(**Fig. 3 and Supplemental Fig. S8) with 4MEC7G presenting potential the most favorable binding with p38-MAPK (Supplemental Table S4, Supplemental Video 1). A closer look at the interactions reveals that the glucuronide group of 4MEC7G forms six hydrogen bonds with p38-MAPK (Fig. 3) while the others metabolites are forming less bonds. Moreover, the measured arbitrary distance of 4MEC7G with residue Thr-180 is 8.1 AU in close proximity towards key residues of phosphorylation. We also performed induced fit docking studies that confirmed our previous findings. In line with the western blot results, our molecular modeling further suggests that flavan-3-ols metabolites might bind in the catalytic pocket of p38 MAPK to impact its phosphorylation status especially at Thr-180 and Tyr-182 residues, leading to decrease in p38-MAPK activation and the downstream signaling cascade.

### Epicatechin metabolite responsive miRNAs contributes in fine-tuning transcriptional regulation of cell adhesion and transendothelial migration pathways

**Figure 4:**
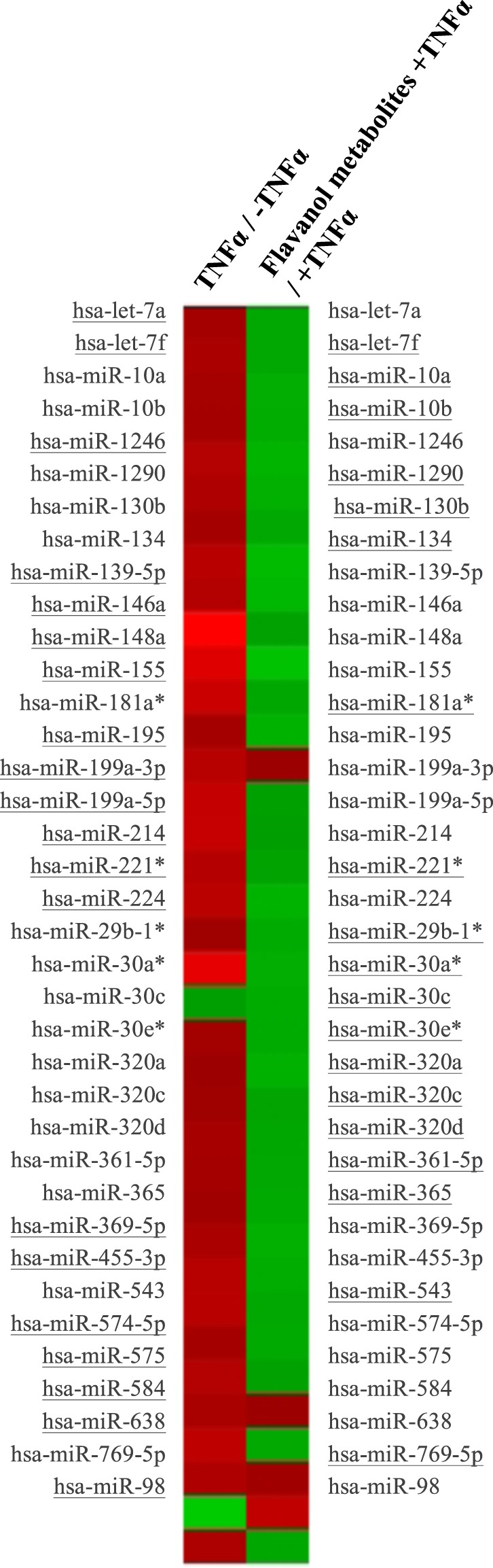
Expression profiles of miRNA in response to TNFα and epicatechin metabolites in HUVECs. Heat map was performed using PermutMatrix software. MiRNAs identified as differentially expressed in at least one condition are underlined. Red represents up-regulation and green down-regulation of expression.

The capacity of epicatechin metabolites to modulate the expression of miRNAs in HUVECs was analyzed. First, stimulation with TNFα significantly changed the expression of 19 miRNAs in comparison to non-stimulated cells (Supplemental Table S5). These 19 miRNAs were identified as up-regulated (Fig. 4). When endothelial cells were exposed to the mixture epicatechin metabolites prior to their stimulation with TNFα, the expression of 20 miRNA was significantly affected (Supplemental Table S5). Among them, 19 were identified as down-regulated (Fig. 4). These results reveal that the mixture of epicatechin metabolites at a physiologically relevant concentration can modulate the expression of multiple miRNAs in endothelial cells. Two miRNAs, hsa-let-7f and hsa-miR-221, were identified as modulated by the activation with TNFα and the pre-exposure to the mixture of metabolites, and interestingly these two miRNAs presented opposite expression patterns in the two conditions.

To identify potential target genes of the differentially expressed miRNAs and consequently the cellular processes that could be affected, the miRWalk database was used. In total, 11899 potential targets genes were identified for all miRNAs with differential expression after TNFα treatment, and 7857 for the miRNAs which expression was affected by the mixture of three epicatechin metabolites. Comparison of the target genes identified for the two conditions showed that 6516 genes were in common.

When we further compared the 253 differentially expressed genes with the 7857 potential target genes of differentially expressed miRNAs, both detected in HUVECs pre-exposed to the epicatechin mixture, we identified that 73 were in common. This suggests that the expression of 30% of the differentially expressed genes in response to epicatechin metabolites could be regulated through modulation of miRNA expression.

**Figure 5:**
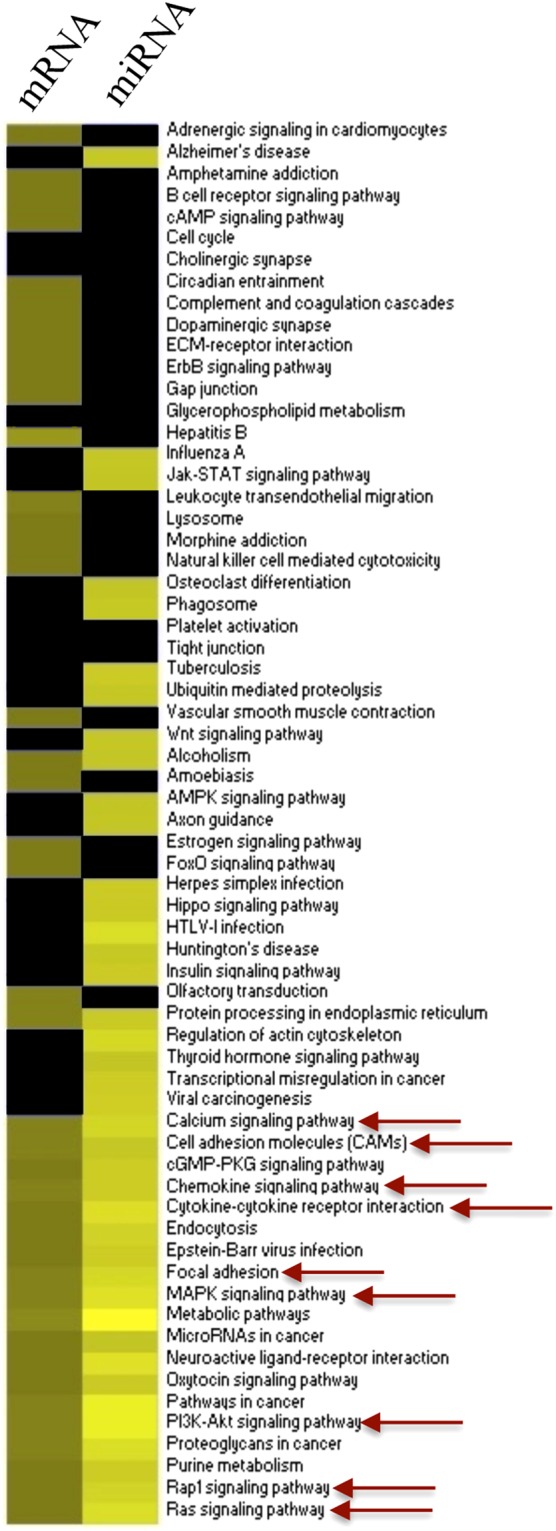
Comparison of the top pathways identified using KEGG database with differentially expressed genes and with target genes of differentially expressed miRNAs in response to exposure of HUVECs to flavanol metabolites. In yellow: number of genes in pathways, bleu: no gene present in pathway; underlined: pathways of interest regarding adhesion and transendothelial migration.

Bioinformatic analyses using KEGG database were performed to identify cellular processes in which the 7857 potential target genes of the 20 miRNAs differentially expressed after epicatechin metabolite treatment are involved in. Over 100 pathways were identified indicating that these miRNA could regulate multiple cellular processes. Comparison of the top 40 most over-presented pathways represented by the target genes of differentially expressed miRNAs with those identified with differentially expressed genes revealed that 26 were identified in common (Fig. 5). Interestingly, among these 26 pathways are pathways regulating of focal adhesion, cytoskeleton organization, cell adhesion, Ras signaling pathway or chemokine signaling pathway. This comparison suggests that the pathways identified using both differentially expressed genes and target genes of miRNA are particularly involved in the regulation of cell adhesion and transendothelial migration.

Since we identified the processes regulating monocyte adhesion and transendothelial migration as an interesting cellular target of epicatechin metabolites, miRWalk database was used to identify the miRNAs involved in the regulation of these processes. Accordingly, 247 miRNAs were identified as post-transcriptional regulators of genes involved in the leukocyte transendothelial migration pathway. Comparison of these miRNAs with the 20 miRNAs identified as modulated by the epicatechin metabolites in endothelial cells revealed that 12 of them are in common. This suggesting that over half of differentially expressed miRNAs are potentially involved in the regulation of endothelial cell function and permeability.

Together with miRwalk, Metacore software was used to identify potential target genes of miRNA which expression has been identified as modulated by epicatechin metabolites and to create gene networks for both mRNA and miRNA targets. Using this approach, several gene networks were obtained. Merging the three most significant networks resulted in a complex network containing 308 target genes (Supplemental Fig. S6). Interestingly, these 308 genes are involved in regulating focal adhesion, chemotaxis, cytoskeleton organization or cell adhesion. Taken together, results obtained using different bioinformatic approaches suggest that differentially expressed miRNA in response to the mixture of epicatechin metabolites are involved in the regulation of different cellular processes, particularly adhesion, cytoskeleton organization or focal adhesion. Therefore, by modulating miRNA expression, epicatechin metabolites could affect cell adhesion and transendothelial migration, thus regulating endothelial permeability.

### Epicatechin metabolites modulate DNA methylation of genes regulating cell adhesion, migration or cytoskeleton organization

**Figure 6:**
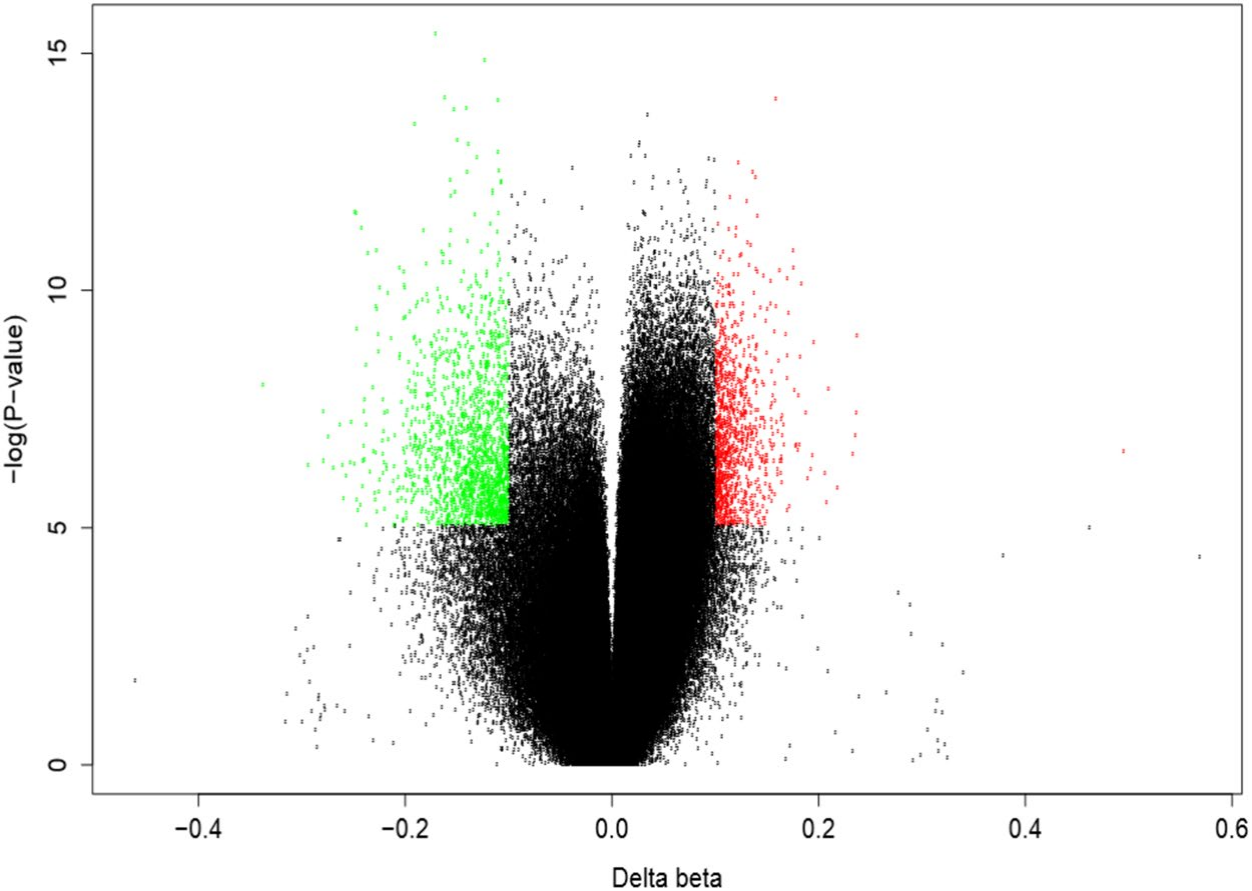
Distribution of individual sites that exhibit differential DNA methylation in HUVECs in response to flavanol metabolites. Volcano plot showing the differentially methylated positions. Hypo-methylated and hyper-methylated positions are colored in green and red, respectively.

To complement nutrigenomic studies, we also performed genome-wide DNA methylation of gDNA samples isolated from HUVECs (Supplemental Fig. S9). Pre-exposure of endothelial cells to the mixture of epicatechin metabolites prior to their stimulation with TNFα induced significant DNA methylation changes at 2521 positions of 1845 genes in comparison to TNFα-stimulated cells (Fig. 6; Supplemental Table S6). This suggests that 3 h exposure of endothelial cells to the mixture of epicatechin metabolites can trigger DNA methylation changes of several hundreds genes in a time frame of 24 h. Remarkably, when comparing DNA methylation changes following exposure to a short TNFα stimulus, no CpG site fulfilled the stringent differentially methylation criteria, suggesting that the impact of TNFα alone on DNA methylation is less potent than a mixture of epicatechin metabolites.

The list of genes identified as presenting changes in methylation when pre-exposed to epicatechin metabolites was compared with genes presenting changes in their expression in the same experimental condition. This analysis showed that 21 genes are in common, suggesting that the observed changes in the expression of 8.3% of genes could be associated with changes in their DNA methylation levels. Amongst the genes that presented nutriepigenetic changes following pre-exposure to epicatechin metabolites were genes coding for microRNAs. Comparison of these genes with the list of miRNAs which expression has been identified as modulated by the epicatechin mixture reveled 2 common miRNAs: let-7a and miR-130b, suggesting that difference in their expression could be due to changes in the methylation of their gene.

Bioinformatic analyses were performed with genes presenting changes in the DNA methylation to identify cellular processes and function in which they are involved. Gene ontology analysis revealed that amongst the most significant group of genes are those involved in cellular processes regulating cell adhesion, cell junction organization, cell junction assembly or chemotaxis. Gene networks search identified 32 significant networks and among the most significant ones, 9 networks are involved in the regulation of cell adhesion, cytoskeleton actin filaments or cell junctions. As with gene ontology analysis, these gene networks plat a role in the regulation of cell adhesion and transendothelial migration. Further pathway analysis revealed that among the most over presented pathways are those regulating actin cytoskeleton organization, focal adhesion, cytoskeleton remodeling, cell adhesion, Ras signaling, chemokine signaling or Rap1 signaling. In conclusion, gene ontology, gene network and pathway analyses with genes presenting differences in methylation in endothelial cells exposed to epicatechin metabolites suggest that they are involved in regulating monocyte adhesion and transendothelial migration.

Comparison of the KEGG pathways identified with differentially methylated genes with those identified with differentially expressed genes, revealed that among the most over-presented ones, 33 are in common. These common pathways include those regulating focal adhesion, cell adhesion, chemokine signaling pathways or regulation of actin cytoskeleton.

### Integrative pathway enrichment analysis of nutri(epi)genomic data reveals common regulation of cell adhesion and transendothelial migration pathways

**Figure 7:**
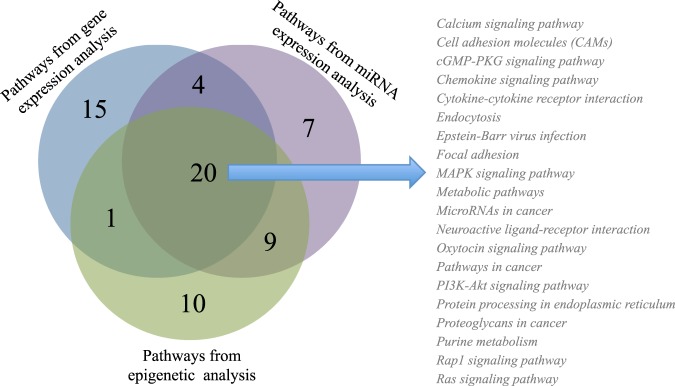
Venn diagram of top pathways identified from gene expression, miRNA expression and epigenetic analyses.

**Figure 8:**
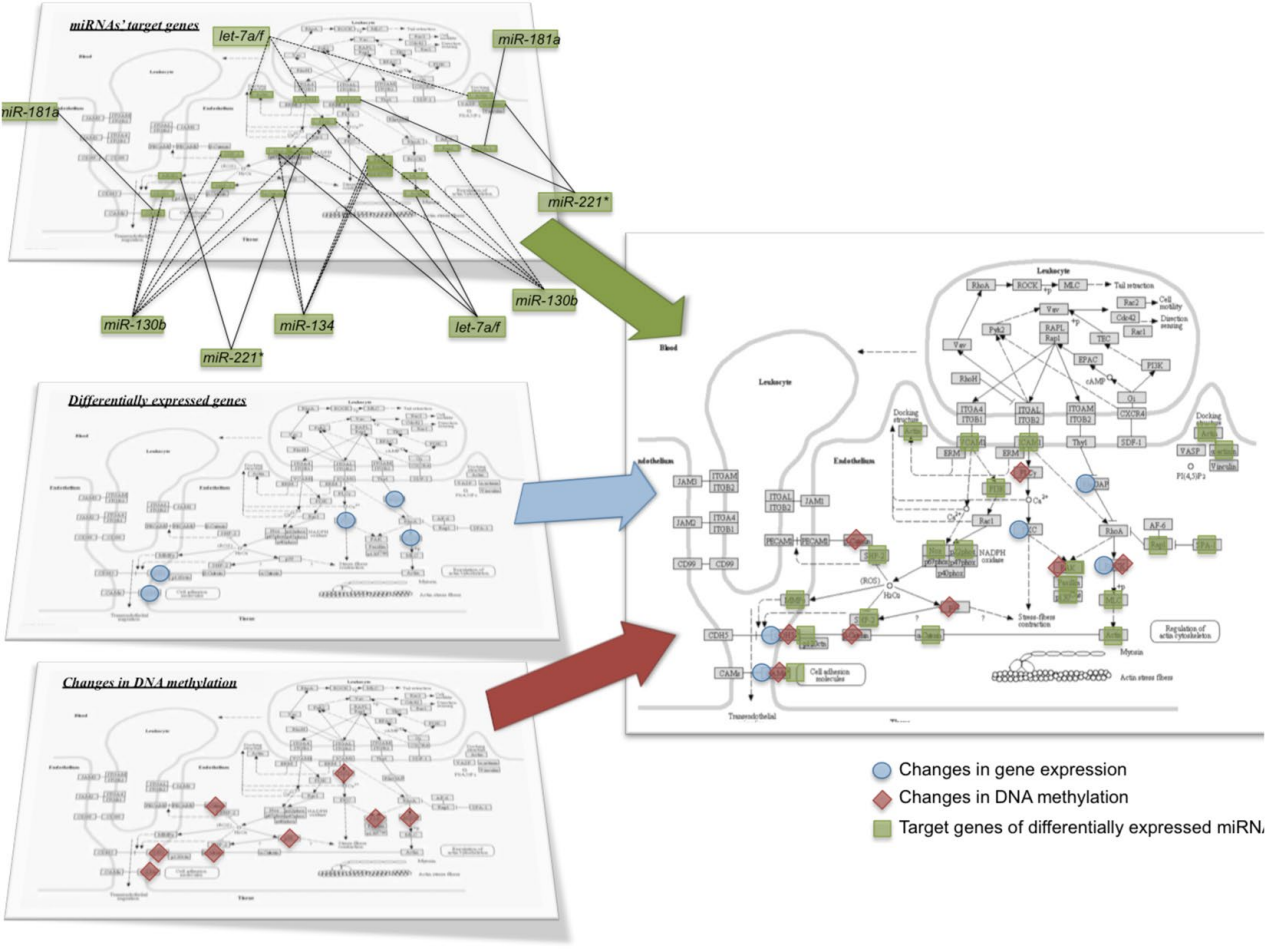
Integrative analysis of the nutri(epi)genomic data in response to flavanol metabolites related to transendothelial migration pathway. The pathway map (KEGG ID: HSA04670) is obtained from Kyoto Encyclopedia of Genes and Genomes (KEGG) database^31,32^, http://www.kegg.jp/kegg/kegg1.html.

Next, we performed integrative enrichment analysis of transcriptomic, miRNomic, and epigenomic analyzes by comparing biological pathways identified using these three nutrigenomic approaches. Amongst the most over-represented pathways identified in the 3 omic analyses, 20 pathways are in common. Interestingly, nearly half of them regulate cell adhesion and transendothelial migration (Fig. 7) such as focal adhesion, cell adhesion molecules or Ras signaling pathway. Figure 8 represents the integrated analysis of nutrigenomics results for the genes of the leukocyte transendothelial migration pathway. This integrated analysis shows that by acting at different levels of regulation, epicatechin metabolites can potentially affect most of the genes in this cellular process which could result in significant modulation of endothelial permeability and interaction with immune cell.

### Epicatechin metabolites decrease monocyte adhesion to activated endothelial cells

**Figure 9:**
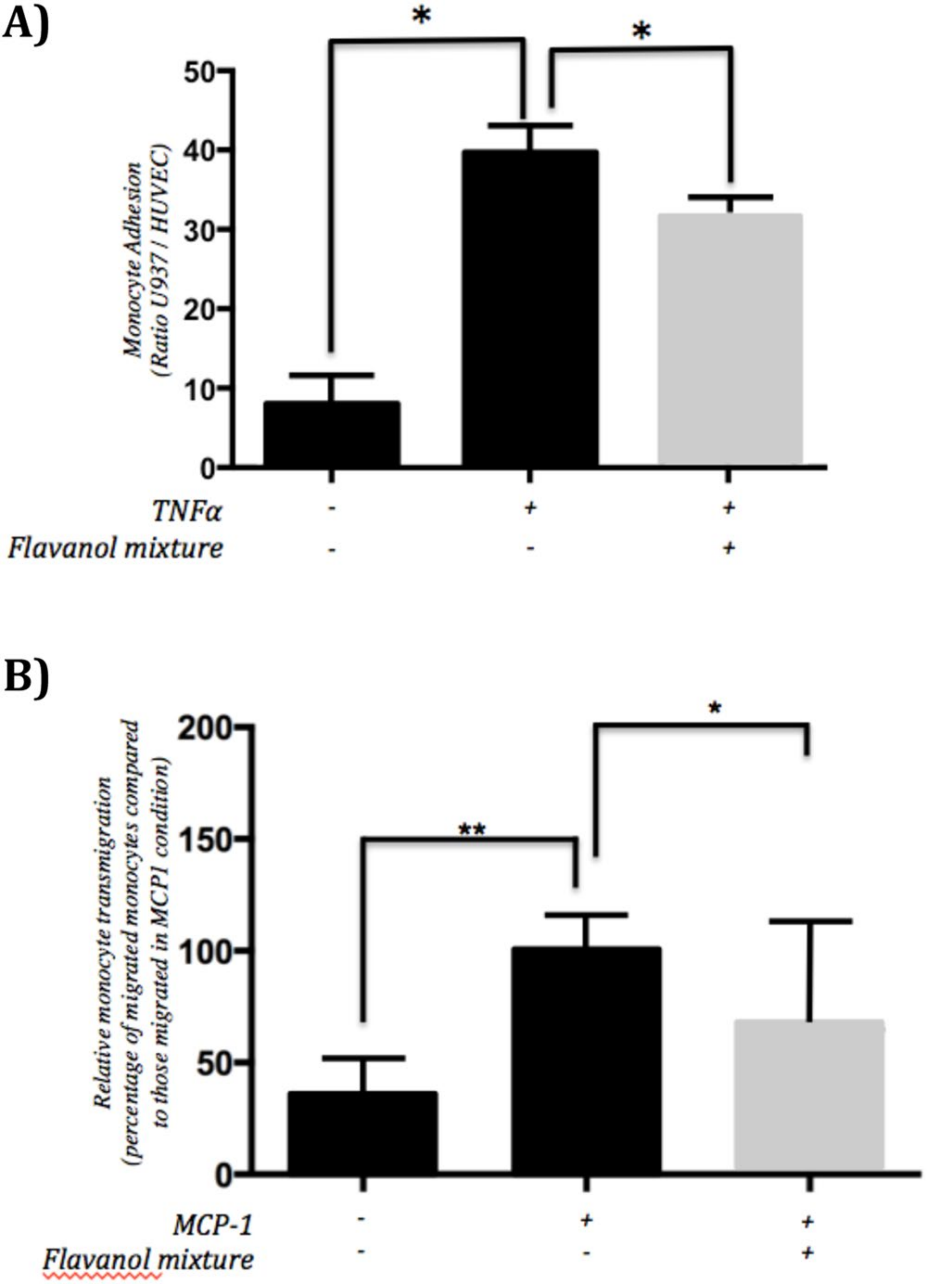
Impact of pre-exposure of endothelial cells to flavanol mixtures on endothelial cells function. (**A**) monocyte to endothelial cell adhesion (*p < 0.005; n = 9; mean +/− SEM; 1-way ANOVA and Tukey’s HSD test). (**B**) transendothelial migration of monocytes (*p < 0.005; **p < 0.05; n = 10; mean +/− SEM; 1-way ANOVA and Dunnett’s multiple comparison test).

For biological validation of hypotheses raised from bioinformatic analyses of nutri(epi)genomics data that epicatechin metabolites can induce changes in cell adhesion, we investigated monocyte adhesion to endothelial cells *in vitro*. Monocytes presented a small adhesion to unstimulated HUVECs monolayer (Fig. 9A). In response to TNFα, as expected, a significant 5.3-fold increase in adherence of monocyte to endothelial cell was identified compared to unstimulated cells. In agreement with our hypothesis, pre-exposure of HUVECs to the mixture of epicatechin metabolites resulted in a significantly reduced (−32%) the adhesion of monocytes to a TNFα- activated endothelial cell monolayer (Fig. 9A).

### Epicatechin metabolites reduce monocyte transendothelial migration

Furthermore, for biological validation of hypothesis that epicatechin metabolites can induce changes in transendothelial migration, HUVECs were cultured on transwells and exposed to epicatechin metabolites with/without chemoattractant. The transmigration of THP-1 cells through the endothelial layer increased threefold after addition of chemoattractant MCP1 in the lower chamber (Fig. 9B). This transmigration of monocytes was significantly decreased by 27% when endothelial cells were exposed to epicatechin metabolites (Fig. 9B) in accordance with our bioinformatic analyses of nutri(epi)genomic data.

## Discussion

Our results provide novel mechanistic evidence supporting the beneficial vascular effects of cocoa flavan-3-ols. In a seven-day dietary epicatechin supplementation, reduced leukocyte rolling was observed upon vascular inflammation in mice. To elucidate the mechanism of action, we applied a systems biology approach utilizing transcriptome, miRNome and epigenome data obtained in human endothelial cells exposed to physiologically-relevant concentrations of the epicatechin metabolites identified in plasma after the consumption of epicatechin-rich foods. As predicted from systems biology analyses of nutri(epi)genomics data, the mixture of the three major circulating forms of epicatechin metabolites was found to attenuate the detrimental effects induced by inflammatory conditions on endothelial cell function, resulting in reduced monocyte to endothelial cell adhesion and transendothelial migration processes.

We used a mouse model of vascular inflammation induced by severe sepsis and intravital microscopy to examine the subcutaneous microcirculation in postcapillary venules and to ascertain the effects of epicatechin metabolites on leukocyte interactions with the endothelium *in vivo*. Dorsal skin fold chamber preparation represents an ideal tool to study the effects of metabolites on leukocyte-endothelial cell interaction since it is well-characterized for the analysis of leukocyte/endothelial cell interaction and the analysis of capillary perfusion^34^. Severe sepsis induces an enhanced inflammatory response resulting in rolling and adherence of leukocytes to endothelial cells^35,36^. In our study, sepsis was induced by puncture in the cecum as it has been described that mice with sublethal cecal ligation puncture (two punctures with a 24-G needle) exhibited increased neutrophil migration to the inflammatory sites^37^. In mice fed with an epicatechin-rich diet, we observed a significant decrease in leukocyte/endothelial interaction in postcapillary venules 12 h post-CLP induction when compared to mice on flavanol-free diet, while red blood cell velocity and venule diameter were similar in both analyzed groups. As red blood cell velocity and the diameter of post-capillary venules are important parameters of the microcirculation, our results suggest that the observed decrease in leukocyte-endothelial cell interaction was not related to either red blood cell velocity or venule diameter. Instead, the decrease in cell interaction observed is more likely due to the capacity of circulating epicatechin metabolites to maintain endothelial integrity. This may represent cellular mechanisms underlying atherosclerotic-protective effects of flavan-3-ols. By attenuating the process of leukocyte recruitment into the vascular wall, which is an early step of atherosclerosis development, flavan-3-ols could exert anti-atherogenic effects, as previously described. Indeed, a dietary supplementation with flavan-3-ols has been shown to significantly lower fat deposits in the aortas of ApoE knockout mice^9^.

To deepen the cellular and molecular impact of epicatechin metabolites on endothelial cells function, we performed *in vitro* studies under conditions replicating as much as possible the pharmacokinetics of ECs in humans following consumption of EC-rich foods; more specifically, we addressed the nature and diversity of the plasma metabolites (sulfate, glucuronide and methyl-glucuronide derivatives) and their low concentrations and short resident time in circulation^10,11^. To evaluate the long-lasting effect of these metabolites, which are just transiently found in plasma, their cellular and molecular impacts in endothelial cells were measured 18 hours after a three-hour exposure of endothelial cells to the mixture of epicatechin metabolites. We first assessed the impact of these metabolites on gene expression using pangenomic microarrays. Our results show that the mixture of EC metabolites can affect the expression of over 200 endothelial genes, which is similar to treatment with the individual metabolites, as previously reported^13^. Bioinformatic analysis of these differentially-expressed genes showed that the most over-represented pathways and related gene networks are those regulating cell adhesion, gap and adherent junctions, chemotaxis and cytoskeleton remodeling. These pathways are known to play an important role in immune cell adhesion and transendothelial migration. Among the genes involved in these processes is vascular endothelial growth factor (VEGF). Indeed, VEGF has been shown both to stimulate monocytes adhesion to HUVECs and increase their transmigration through the endothelial monolayer^38^. In this study, the decreased expression of *VEGF* in response to the mixture of metabolites could result in a lower adhesion and transendothelial migration of monocytes. In contrast, we observed an up-regulation of the expression of myosin light chain phosphatase (*MLCP*) by EC metabolites. Inhibition of *MLCP* has been previously shown to increase MLCK-dependent phosphorylation of MLC and actomyosin contractility causing retraction in cell junctions that contribute to an increase in endothelial hyperpermeability^39^. Therefore, flavanol-induced *MLCK* gene induction might result in lowering MLC phosphorylation and actomyosin contraction and thereby decreasing endothelial permeability. Together with *MLCK*, several other genes encoding members of Ras and Rho family of Small GTPases (*RAPGEF3*, *RASGRP3*, *RHOC*, *ARHGAP27*, *ARHGAP42*, *ARHGEF35*) were identified as differentially-expressed by the mixture of EC metabolites. These genes play an important role in the regulation of cytoskeletal contractility, cytoskeletal changes, MLC phosphorylation and actomyosin (stress-fiber) contractility, pathways that regulate the endothelial permeability by controlling gaps between adjacent endothelial cells^40–42^. It has been shown that the expression of phosphatidylinositol (3,4,5)-trisphosphate-dependent Rac exchanger 1 (*P-Rex1*), a Rac-specific guanine nucleotide exchange factors, is induced by TNFα and that the inhibition of its expression significantly reduced leukocyte transendothelial migration^15^. This study also reported that P-Rex1 is a critical mediator of vascular barrier disruption and that targeting P-Rex1 may effectively protect against TNFα-induced endothelial junction disruption and vascular hyperpermeability. In our study, we observed a decreased expression of the P-Rex1 gene in response to the mixture of epicatechin metabolites, which is suggestive of a decrease in TNFα-induced endothelial hyperpermeability. Taken together, the observed expression profile of these differentially expressed genes suggest a decrease in adhesion and transendothelial migration of circulating immune cells in response to the mixture of EC metabolites.

Analysis for enriched transcription factors involved in the nutrigenomic effect of EC metabolites revealed NF-κB, CREB1, cMyc, c-JUN, STAT3 or SP1 as top ranked transcription factors. Interestingly, it has been shown that these transcription factors are involved in the regulation of vascular inflammation, immune cell adhesion and transendothelial migration, for example *CREB1*^43^, *c-Myc*^44^, *c-Jun*^45^ or *Sp-1*^46^. The phosphorylation and subsequent activation of these transcription factors are regulated by different intracellular cell signaling proteins, one of which is p38. We observed that the pre-exposure of cells to mixture of epicatechin metabolites results in significant reduction of p38-MAPK phosphorylation. It has been reported that decreased p38 activity is associated with reduced neutrophil adhesion to endothelial cells as well as reduced transmigration^47^. Taken together, theses results suggest that epicatechin metabolites exert part of their nutrigenomic effect through proteins of cell signaling and transcription factors that control expression of genes involved in regulation of endothelial cell integrity and permeability. To identify potential interactions between the cell signaling proteins and flavanol metabolites that could affect cell-signaling activity of these proteins, we performed *in silico* docking analyses. Only a few docking binding studies have been reported for polyphenols. For example cocoa and tea catechins have been shown to have strong binding affinities for glucosamine-6-phosphate synthase (Fikrika *et al*., 2016) or other polyphenols (rosmarinic acid, sinensetin or eupatorin) to angiotensin-converting enzyme^48^. However, these analyzes have been performed for native compounds and not for circulating metabolites. In our *in silico* study, we observed that the EC metabolites can bind to ATP binding pocket of the p38-MAPK cell signaling protein. It has been shown that compounds like pyridinylimidazole can bind to the ATP binding pocket of p38-MAPK, which results in the inhibition of the activity of this protein^49^. Inhibition of p38 in activated endothelial cells reduces lymphocyte adhesion and transmigration across the endothelial monolayer^50^. Accordingly, our *in vitro* analyses suggest that EC metabolites can bind to the ATP-binding pocket of the p38-MAPK signaling protein, decrease its activation and consequently decrease monocyte adhesion and transendothelial migration in accordance with our *in-vitro* cell assays.

Regulation of gene expression can also involve small non-coding RNAs known as microRNAs. The mechanism of gene expression regulation by miRNA is complex, as one miRNA can regulate the expression of several hundred genes and one mRNA can be regulated by several miRNAs. The miRNAs are involved in the development of different chronic diseases and dysfunctions, including endothelial dysfunction. It has been described that polyphenols can also modulate the expression of miRNAs^14^. In the present study, we observed that treatment of HUVECs with TNFα triggers the expression of 19 miRNA when compared to non-stimulated HUVECs. This observation is in agreement with a previous study demonstrating the capacity of TNFα to modulate the expression of miRNA, such as miR-155, miR-146a or miR-638, in endothelial cells^51,52^. In cells pre-exposed to the EC metabolites mixture, we identified 20 differentially expressed miRNAs. Two miRNAs were found to be modulated by both TNFα and the EC mixture, but in opposite directions. MiR-let-7f has been reported to be expressed in endothelial cells and an increased expression of this miRNA has been described as pro-angiogenic^53^. Regarding miR-221, the expression of this miRNA was reported to promote the expression of adhesion molecules in endothelial cells^54,55^. Consequently, the expression profile of these two miRNAs in endothelial cells pre-exposed to EC metabolites is suggestive of an anti-inflammatory effect and decreased monocyte adhesion to endothelial cells.

Among the miRNAs identified as differentially expressed by EC metabolites, several are involved in the regulation of endothelial cell function. For example, increased expression of miR-181a* is associated with the development of atherosclerosis^56^. In ApoE(−/−) mice, expression of miR-320a was shown to induce endothelial cell dysfunction and to promote atherogenesis^57^. In our study, the expression of these two miRNAs are down-regulated in endothelial cells that are pre-exposed to the metabolites. Other miRNAs have been associated with alternative functions in endothelial cell functions. For example miR-10a is involved in the regulation of inflammatory response of endothelial cells^58^, miR-1290 is involved in the regulation of the endothelial barrier^59^, miR-130b and^60^ miR-361-5p can regulate angiogenesis^61^, whereas miR-365 modulates apoptosis^62^. Taken together, our results suggest that EC metabolites can modulate the expression of miRNAs involved in the regulation of different endothelial cell functions, including monocyte adhesion and transendothelial migration.

Upon linking differentially-expressed miRNAs with potential mRNA targets in the miRWalk database, we observed that nearly a third of the differentially expressed mRNAs are in common with the predicted miRNA target genes of differentially expressed miRNAs. This observation suggests that the EC metabolites can exert post-transcriptional fine-tuning of gene expression by modulating the expression of miRNAs. Interestingly, comparison of the miRNA-associated pathways with the pathways identified with differential mRNA expression showed that half are involved in the regulation of cell adhesion and endothelium permeability. Similarly, comparing the list of leukocyte transendothelial migration-associated miRNAs and EC responsive miRNAs reveals a 50% overlap. Taken together, our findings suggest that EC-responsive miRNAs play an important role in the post-transcriptional regulation of gene expression associated with regulation of monocyte-endothelial cell interactions.

Besides transcriptional, post-transcriptional and translational mechanisms, epigenetic DNA methylation changes have recently been added to the hierarchical regulation of the endothelial phenotype. Recent DNA methylation profiling studies have demonstrated significant epigenetic changes in the endothelium of atherosclerosis-susceptible pre-lesional arteries^63–65^. Dietary interventions that improve cardiovascular function may also trigger epigenetic changes to modulate endothelial cell functions^21,66^. Genome-wide DNA methylation profiling of TNFα-primed endothelial cells pre-treated for three h with a mixture of EC metabolites revealed changes in the DNA methylation of genes involved in endothelial cell adhesion, migration and cytoskeletal reorganization functions. Interestingly, one of the hypermethylated gene loci, C6orf10-BTNL2, has been associated in GWAS with susceptibility loci for coronary artery disease^67^. Expression levels of another hypermethylated locus, RPS24, have been associated with dilated cardiomyopathy^68^. Finally, the hypermethylation gene locus, ADAMTS2, has been associated with increased risk for vascular injury, stroke and aneurysm^69,70^. Remarkably, short-tem exposure (three h) to metabolites appears to be sufficient to elicit significant DNA methylation changes within 18 h following the time of exposure. In line with our results, dynamic changes in DNA hydroxymethylation have recently been reported in response to changes in oxidative stress or variable concentrations of metabolic cofactors of chromatin writers, readers naderasers^71,72^. Similarly, EC-gallate polyphenols were shown to prevent PCB-induced endothelial cell inflammation via epigenetic modifications of NF-κB target genes in human endothelial cells^73,74^.

Altogether, our systems biology based integration of transcriptomic, microRNomic and epigenomic data of inflamed HUVEC cells exposed to a mixture of plasma metabolites of EC revealed common pathways associated with reduced endothelial cell adhesion and transmigration functions. Biological support of the predicted effect of epicatechin metabolites on endothelial cell adhesion and transmigration was obtained using cell *in vitro* assays performed under the same experimental conditions as those used for nutrigenomic analyses. Adhesion of monocytes to endothelial cells, their rolling over vascular endothelium and successive migration into intima layer of vascular wall are processes constituting first steps of atherosclerotic development that lead to cardiovascular disease development^75^. In accordance with our hypothesis, we showed that the pre-incubation of endothelial cells with EC metabolites not only resulted in the reduction of monocyte adhesion, but also in that of endothelial permeability as shown by a decrease in monocyte transendothelial migration. To the best of our knowledge, this is the first study demonstrating that plasma EC metabolites at low physiologically-relevant concentrations can modulate monocyte transendothelial migration, a key step to immune cell extravasation and development of atherosclerosis. The ability of polyphenols to modulate monocyte transendothelial migration has been reported in few *in vitro* studies. However, these studies were of low physiological relevance because they have been performed using native molecules or polyphenol-rich extracts. Taken together, results from our study provide convincing evidence that EC metabolites trigger complex nutri(epi)genomic changes that subsequently modulate endothelial cell adhesion function and permeability. This study also identified key molecular and cellular targets of EC associated with their vascular protective effects *in vivo*.

## Electronic supplementary material

in silicon docking

supplemental figures and tables
