# Supplementary material for: A systems biology network analysis of nutri(epi)genomic changes in endothelial cells exposed to epicatechin metabolites
Source: Sci Rep. 2018 Oct 19;8:15487. doi: 10.1038/s41598-018-33959-x (PMC6195584; doi:10.1038/s41598-018-33959-x)
Supplement: Supplementary file 2 — supplemental figures and tables [file 41598_2018_33959_MOESM2_ESM.pdf]

Supplemental information

A systems biology network analysis of nutri(epi)genomic changes in endothelial cells exposed to epicatechin metabolites

Dragan Milenkovic[a,b*], Wim Vanden Berghe[c,d], Christine Morand[a], Sylvain Claude[a], Annette van de Sandt[e], Simone Gorressen[e], Laurent-Emmanuel Monfoulet[a], Chandra S. Chirumamilla[c], Ken Declerck[c], Katarzyna Szarc vel Szic[c], Maija Lahtela-Kakkonen[f], Clarissa Gerhauser[g], Marc W. Merx[d#], Malte Kelm[c]

[a] INRA, UMR 1019, UNH, CRNH Auvergne, F-63000 Clermont-Ferrand ; Clermont Université, Université d'Auvergne, Unité de Nutrition Humaine, BP 10448, F-63000 Clermont-Ferrand, France

[b] Department of Internal Medicine, Division of Cardiovascular Medicine, School of Medicine, University of California Davis, Davis, California, 95616, United States of America.

[c] PPES, Department of Biomedical Sciences, University of Antwerp (UA), Wilrijk, Belgium

[d] L-GEST, Department of Biochemistry and Microbiology, UGent, Ghent, Belgium

[e] Department of Medicine, Division of Cardiology, Pneumology and Angiology, University Hospital Düsseldorf, Germany

[f] School of Pharmacy, University of Eastern Finland, Kuopio, Finland

[g] Division of Epigenomics and Cancer Risk Factors, DKFZ, Heidelberg, Germany

[#] Actual address: KRH Klinikum Robert Koch Gehrden, Department of Cardiovascular Diseases and Internal Intensive Medicine, Von-Reden-Straße 1, 30989 Gehrden, Germany

Supplemental Note:

Microcirculation mouse model - Intravital fluorescence microcopy

Mice were anesthetized by an intraperitoneal (i.p.) administration of ketamine (60 µg/g body weight) and xylazine (10 µg/g body weight). Upon surgical anesthesia, the entire back of the mice was shaven and a chemical hair remover was used to remove any remaining hair. The technique of implanting an Aluminium access chamber in the dorsal skin flap of rats, originally described by Papenfuss et al. (Papenfuss et al, 1979), was modified and adapted to meet carefully established criteria of stability (thin, translucent skin) in mice. Therefore, spacers made of stainless steel were used yielding in a frame-to-frame distance of 400-450 µm while the chamber was affixed in a position perpendicular to the animal's back. The thickness of the preparation and thus the distance between both frames were chosen on the basis of optical translucency needed for proper visualization of microscopic blood channels without compressing larger blood vessels. This chamber was tolerated by all animals.

The surgical procedure was done by placing the animal on a surgical stage. A midpoint line was drawn along the back and a dorsal skin fold was affixed to the surgical stage by means of silk sutures (5-0) to stabilize the preparation. One part of the chamber frame consisting of the frame, two lower bolts and spacers was slightly pushed under the skin-fold causing the skin to protrude. Two holes were cut carefully through the skin, this part of the chamber introduced through the holes and fixed using two baby mosquito hemostats. A template, equivalent to the outer diameter of the chamber's collar served to mark a circle outlining the subsequent incision. A crisscross cut resulting in four skin flaps was made, and each of the four flaps was removed with a fine, curved scissor tracing precisely the perimeter of the outline with an effort to follow the hypodermis. The

area exposed was freed from overlaying fascia utilizing an operating microscope leaving one layer of s.c. tissue at the opposite side of the epidermis intact. During this surgical procedure, the area was kept moist by allowing drops of warmed normal saline to irrigate the preparation that was limited to one side of the skin-fold only. After trimming the area under observation, the matching side of the chamber was inserted. Mechanical connection of the two chamber frames was accomplished using metal nuts for top and lower bolts. At seven points, the 5-0 sutures were fixed around the chamber to support the position of the skin flap. Animals were allowed for recovery for 72h before first intravital fluorescence microscopy was performed. Pain medication (buphrenorphine 0.05mg/kg body weight) was administered subcutaneously immediately after surgery and every 8 hours thereafter.

For the in vivo microscopy analysis, mice were immobilized and the skin fold preparation was attached to the microscope stage. After intravenous injection of 0.05 ml of 5% Fluorescein isothiocyanate-dextran (Sigma-Aldrich, Germany) which stains blood plasma, and in vivo leukocyte staining by 0.05 ml 1% Rhodamine 6G chloride (Invitrogen, Germany) injection in a tail vein or the retro-bulbar venous plexus, in vivo microscopy was performed using a Leica DM 4000M microscope. The observations were recorded by video camera and transferred to a video system for off-line evaluation. The microscope images were recorded on DVD and analyzed during playback by using CapImage (Dr. Zeintl, Germany). Microscopy was performed at baseline (72 h after application of dorsal skin fold chamber) and 12h after CLP induction.

Microcirculatory analysis

Stained plasma allowed the measurement of vessel diameters and red blood cell velocity (RBCV). RBCV was assessed in the centerline of the respective vessel by frame-to-frame analysis. Stained rolling leukocytes were defined as cells moving along the endothelial lining at a

velocity markedly slower than that of the surrounding red cell and are given as percentage of the total number of calculated leukocytes passing through the observed vessel segment in 1 minute.

Sepsis induction

Mice were rendered septic by cecum ligation and puncture (CLP) (Merx et al, 2005; Merx et al, 2004) 6 h after baseline intravital fluorescence microscopy was performed. Anesthesia was induced by intraperitoneal (i.p.) administration of ketamine (60 µg/g body weight) and xylazine (10 µg/g body weight). Through a 1-cm abdominal midline incision, the cecum was ligated below the ileo-cecal valve with careful attention to avoid obstruction of the ileum or colon. The cecum was then subjected to a single "through and through" perforation with a 20-gauge needle. After repositioning of the bowel, the abdominal incision was closed in layers with standard silk surgical suture 4-0 (Ethicon, Somerville, New Jersey). Sham operated mice underwent the same procedure, except for ligation and perforation of the cecum. Pain medication (buphrenorphine 0.05mg/kg body weight) and volume support (NaCl 0.9%, 0.05 ml/g BW) were administered subcutaneously immediately after sepsis induction and every 8 hours thereafter. All mice had unlimited access to food and water. Twelve hours after sepsis induction, intravital microscopy was performed again to analyze the effects of microbial sepsis in microcirculation.

Supplemental Figure S1: Mice study. (A): schematic presentation of study design. (B): composition of used diets

A)

Diet	Flavanol free diet		flavanol rich diet	
	gm%	kcal%	gm%	kcal%
Protein	16	15	16	15
Carbohydrate	57	50	57	50
**Fat**	**18**	**36**	**18**	**36**
Total		100		100
Kcal/gm	4,5		4,5	
**Ingredients**	**gm**	**kcal**	**gm**	**kcal**
Casein	167,5	670	167,5	670
DL-Methionine	2,57	10,28	2,57	10,28
Corn starch	286,5	1146	286,5	1146
Fructose	143,25	573	143,25	573
Dextrose (Glucose)	143,25	573	143,25	573
Cellulose,BW200	50	0	50	0
Milk Fat (Anhydrous)	176	1584	176	1584
Corn Oil	10	90	10	90
Ethoxyquin	0,04	0	0,04	0
Mineral Mix S10001	35	0	35	0
Calcium Carbonate	4	0	4	0
Vitamin Mix V13005	10	40	10	40
Vitamin K1	0,0005	0	0,0005	0
Choline Bitartrate	2	0	2	0
Cholesterol	1,5	0	1,5	0
epicatechin	0	0	0,06	0
Nitrate	0	0	0	0
Yellow-Orange Dye	0	0	0,05	0
Blue Dye	0	0	0	0

B)

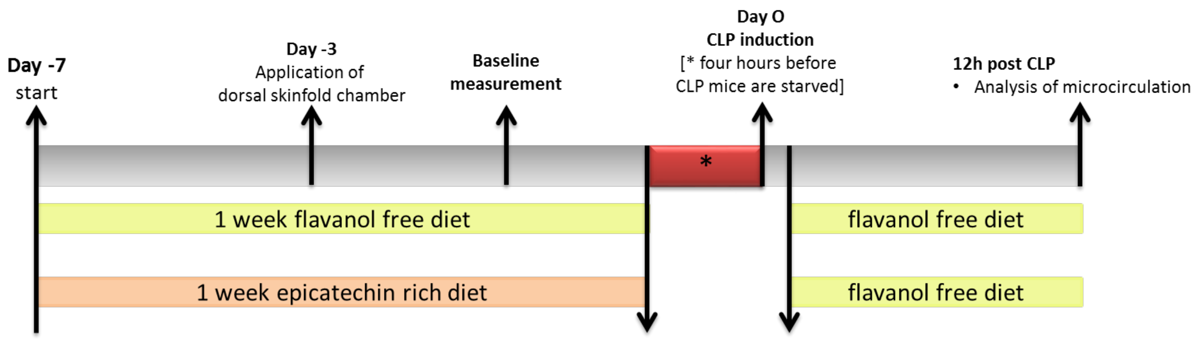

Supplemental Figure S2: Chemical structures of epicatechin metabolites used for in-vitro studies

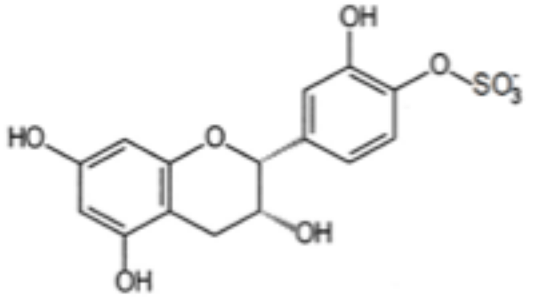

(−)-epicatechin-4′-sulfate

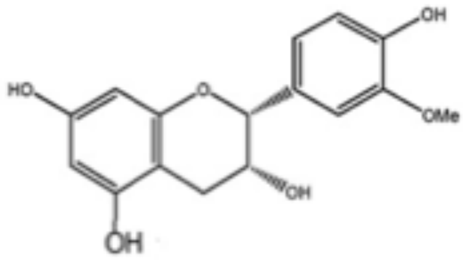

3′-*O*-methyl(−)-epicatechin

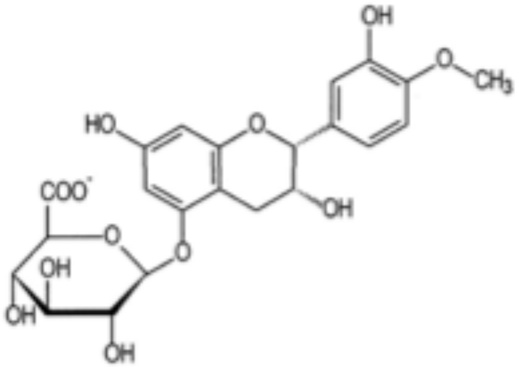

4′-*O*-methyl(−)-epicatechin-7-β-D-glucuronide

Supplemental Figure S3: Circulating white blood cells were analyzed at baseline and 12h after sepsis induction after treatment with flavanol-free and flavanol-rich diet over a period of 7 days. Both groups slightly diminished circulating white blood cells 12h post CLP compared to baseline, while circulating white blood cells did not differ at baseline and 12h post CLP induction between both groups (n=5, mean +/- SEM; 2-way ANOVA and Bonferoni's posttest).

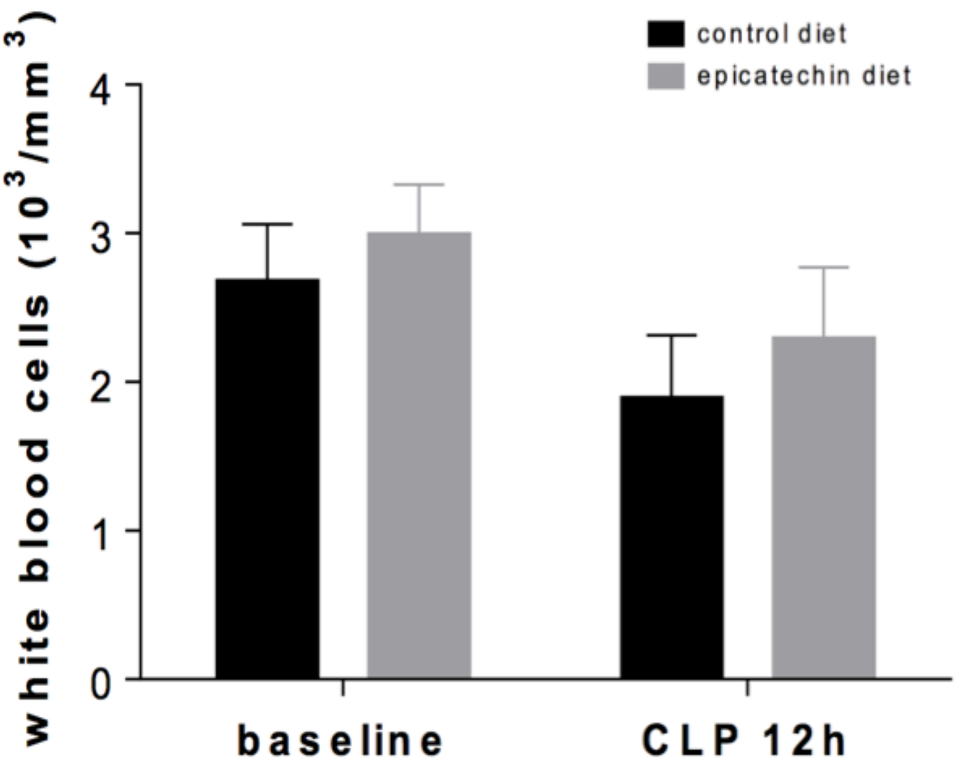

Supplemental Figure S4: Venules diameter measurements in the dorsal skinfold chamber in post-capillary venules at baseline and 12h after sepsis induction. Mice are treated with flavanol-free and flavanol-rich diet over a period of 7 days. A: picture of measurements in venules (D: diameter; V: velocity) B: Venules diameter measured 12h after CLP induction in mice compared to baseline (n=5, mean +/- SEM; **p< 0,01; 2-way ANOVA and Bonferoni's posttest).

A

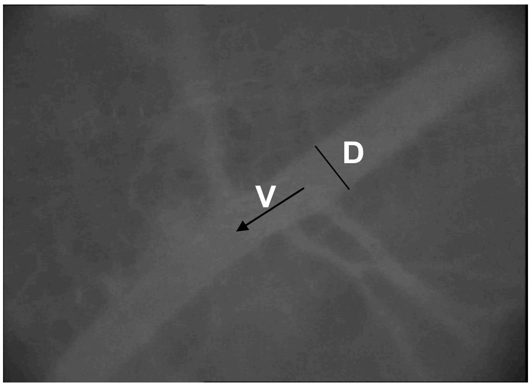

B

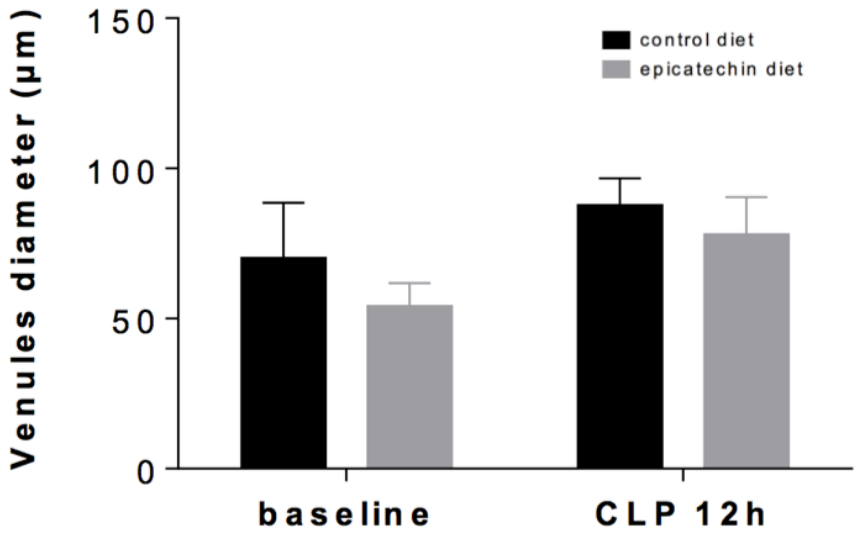

Supplemental Figure S5: Red blood cell velocity measurements in the dorsal skinfold chamber in post-capillary venules at baseline and 12h after sepsis induction (n=5, mean +/- SEM; **p< 0,01; 2-way ANOVA and Bonferoni's posttest).

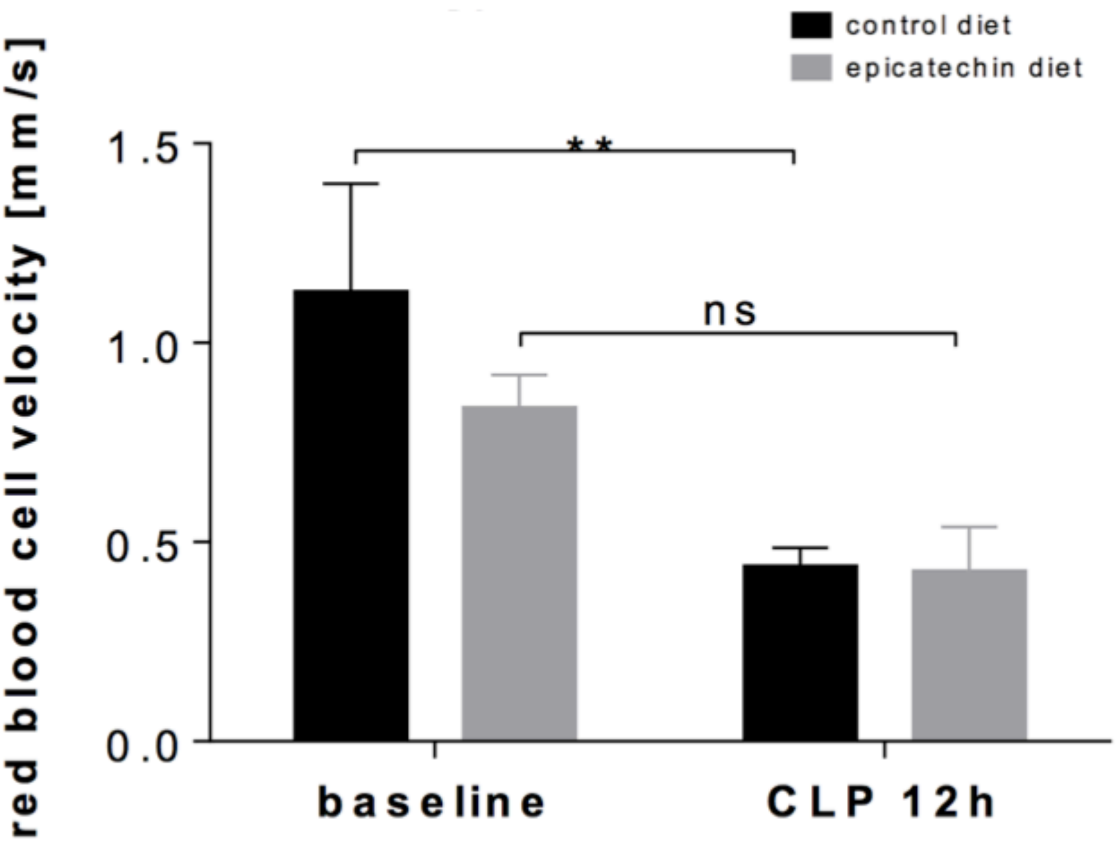

Supplemental Figure S6: Merge of three top scoring gene networks obtained from the list of differentially expressed miRNA in response to epicatechin metabolites in HUVEC with their validated target genes. The networks and target genes were obtained from MetaCore database. Red, bleu and orange lines represent the three networks.

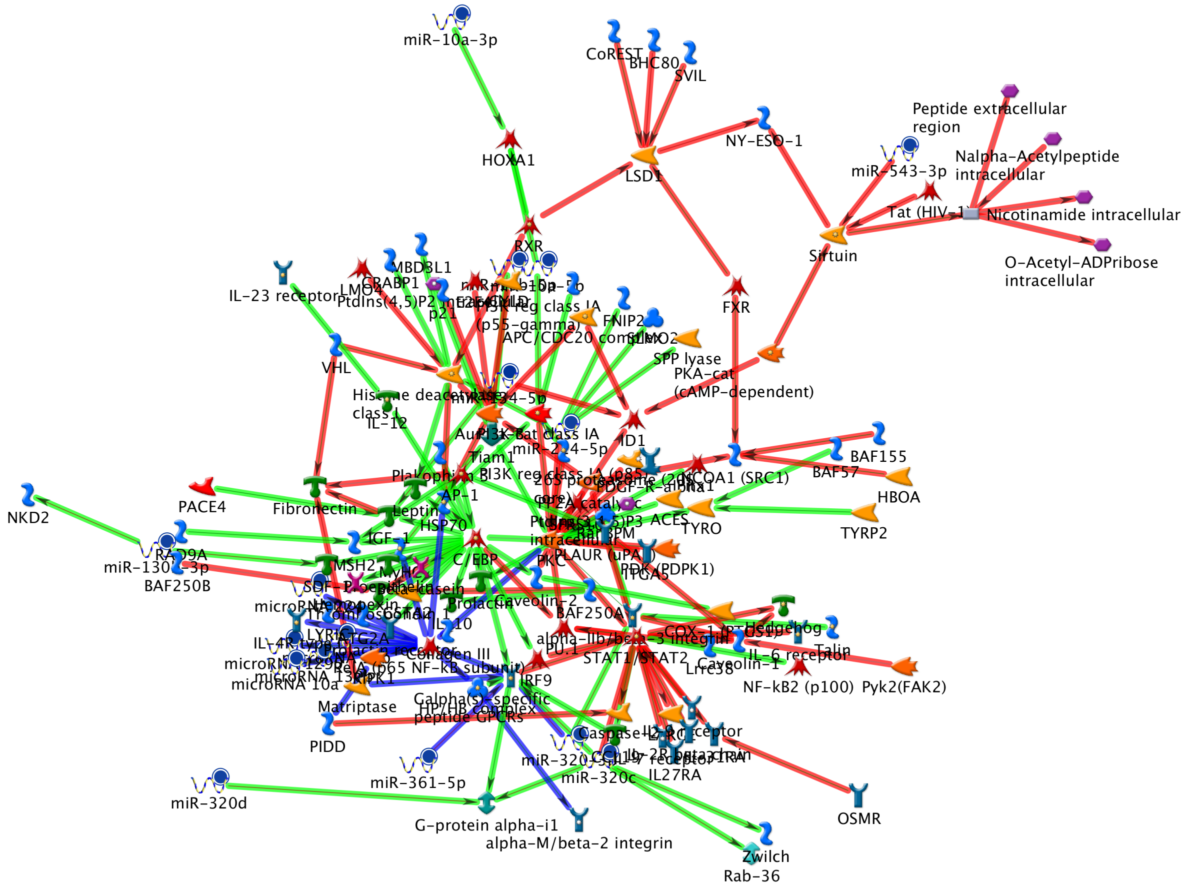

Supplemental Figure S7: Western blot analysis of the impact of flavanol metabolites on phosphorylation of p38 cell signalling proteins in HUVECs. n=3 ; mean +/- SEM; *: p < 0.01; 2-way ANOVA.

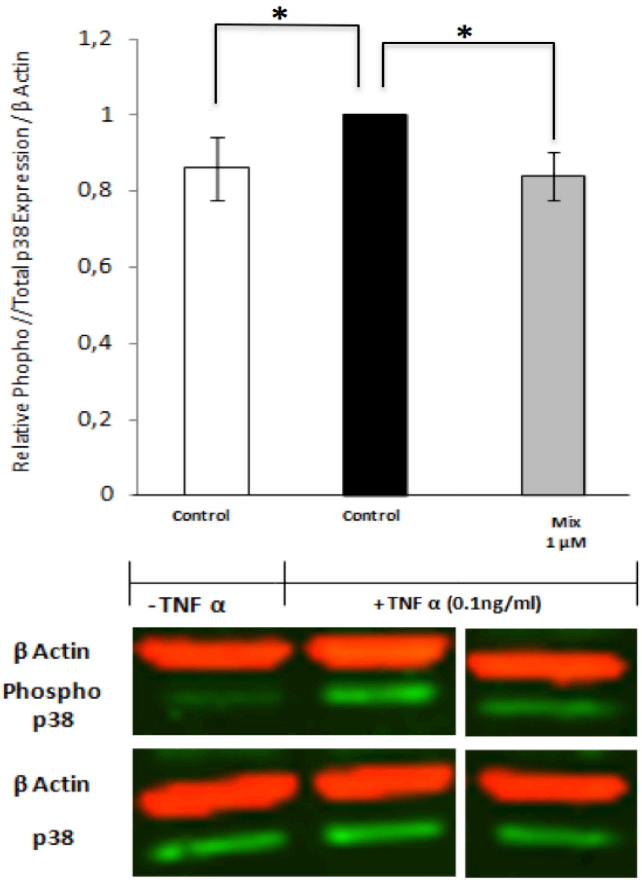

Supplemental information: western-blot gels for p38 and Phospho-p38. Gels were analyzed using Odyssey Li-Cor (Lincoln, Nebraska, USA) detection system and density of the signals was quantified using Odyssey software. TNF: tumor necrosis factor; MIX: mixture of studied flavanol metabolites; NR: samples not related to this work

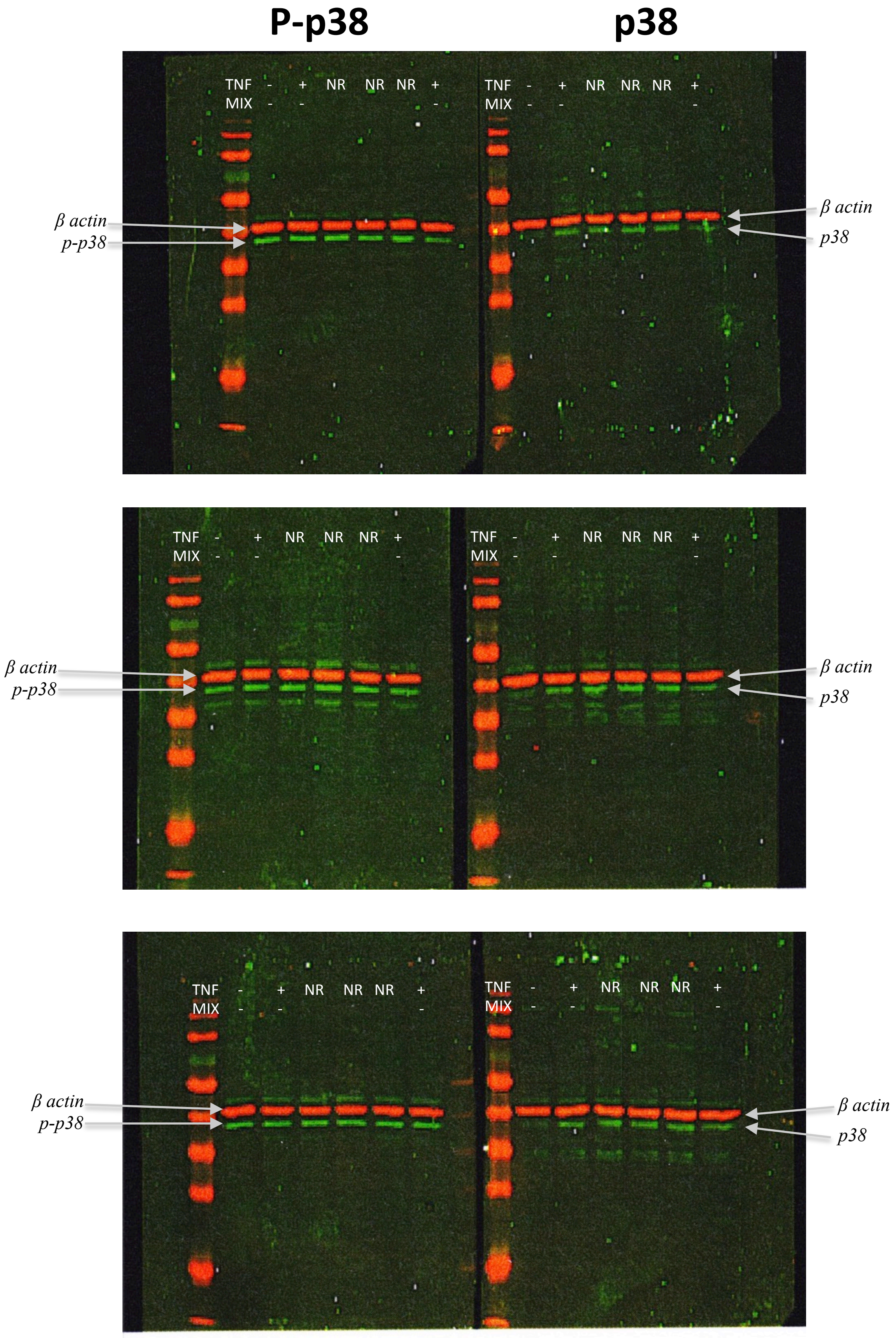

Supplemental Figure S8: Docking studies showing the binding of the metabolites to the ATP binding cavity of the P38-MAPK. A) 3’-O-methyl(-)-epicatechin; B) 4’-O-methyl(-)-epicatechin-7-β-D-glucuronide; C) (-)-epicatechin-4’-sulfate

A) 3′-*O*-methyl(-)-epicatechin

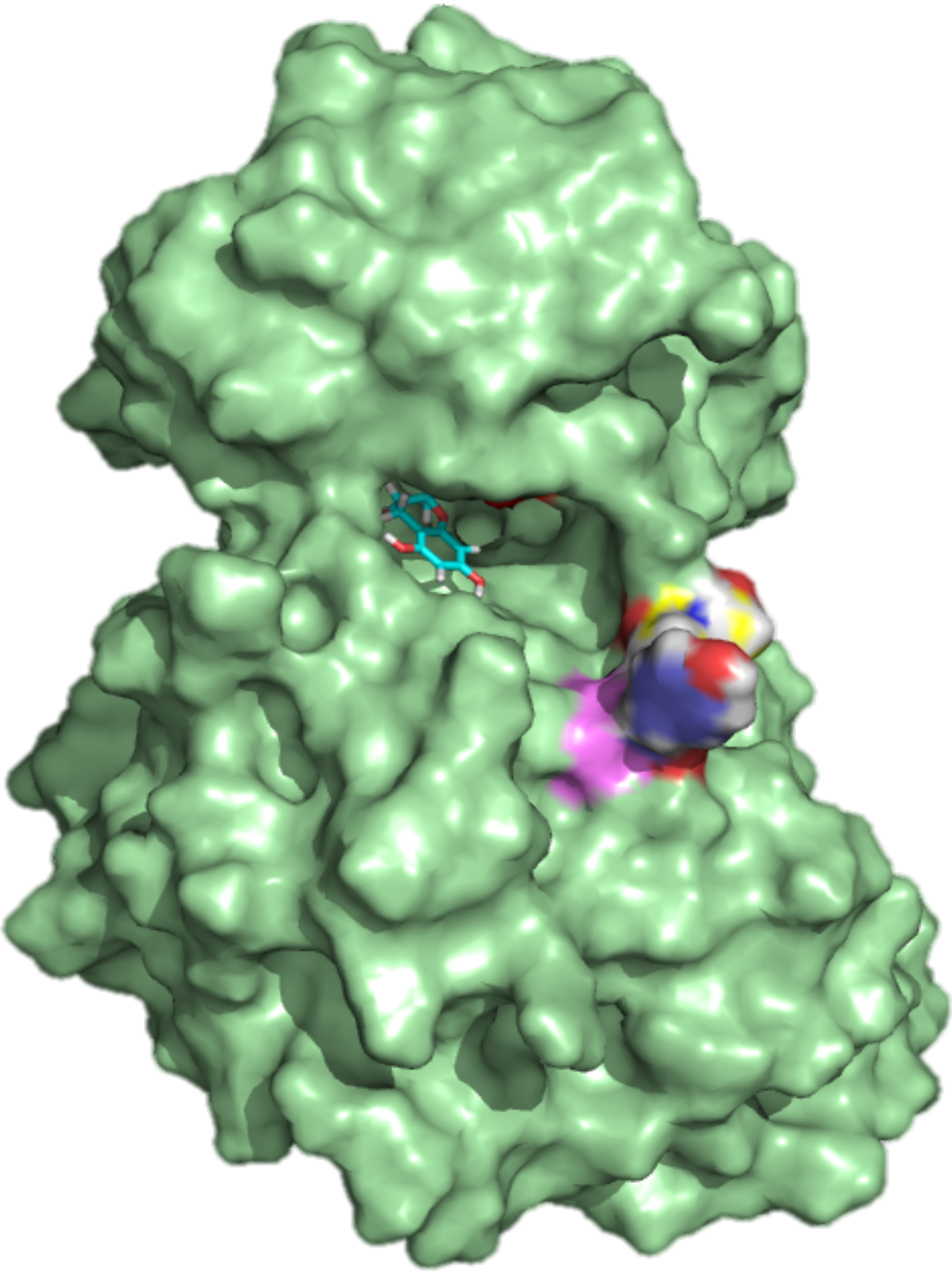

B) 4′-*O*-methyl(-)-epicatechin-7-β-D-glucuronide

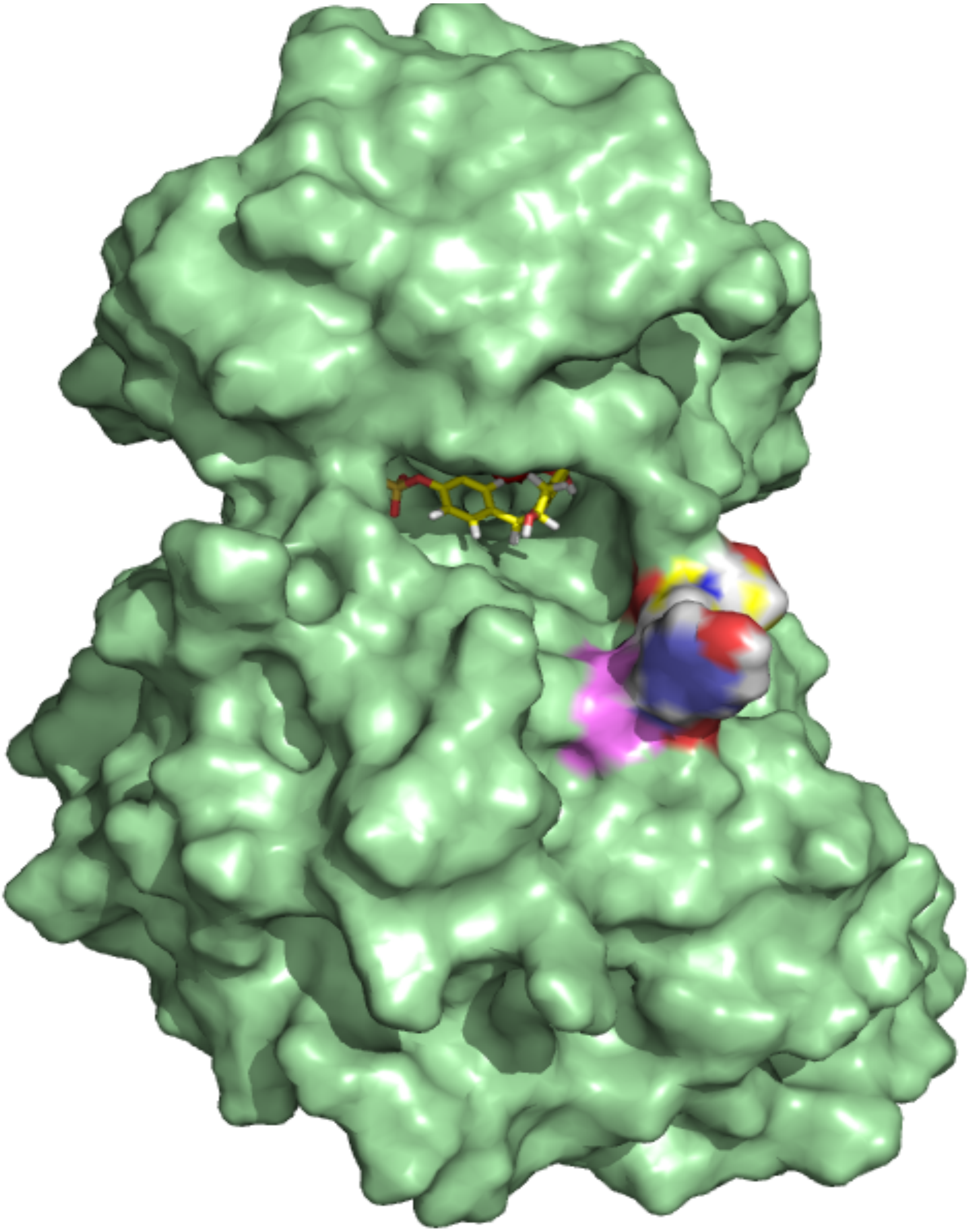

C) (-)-epicatechin-4′-sulfate

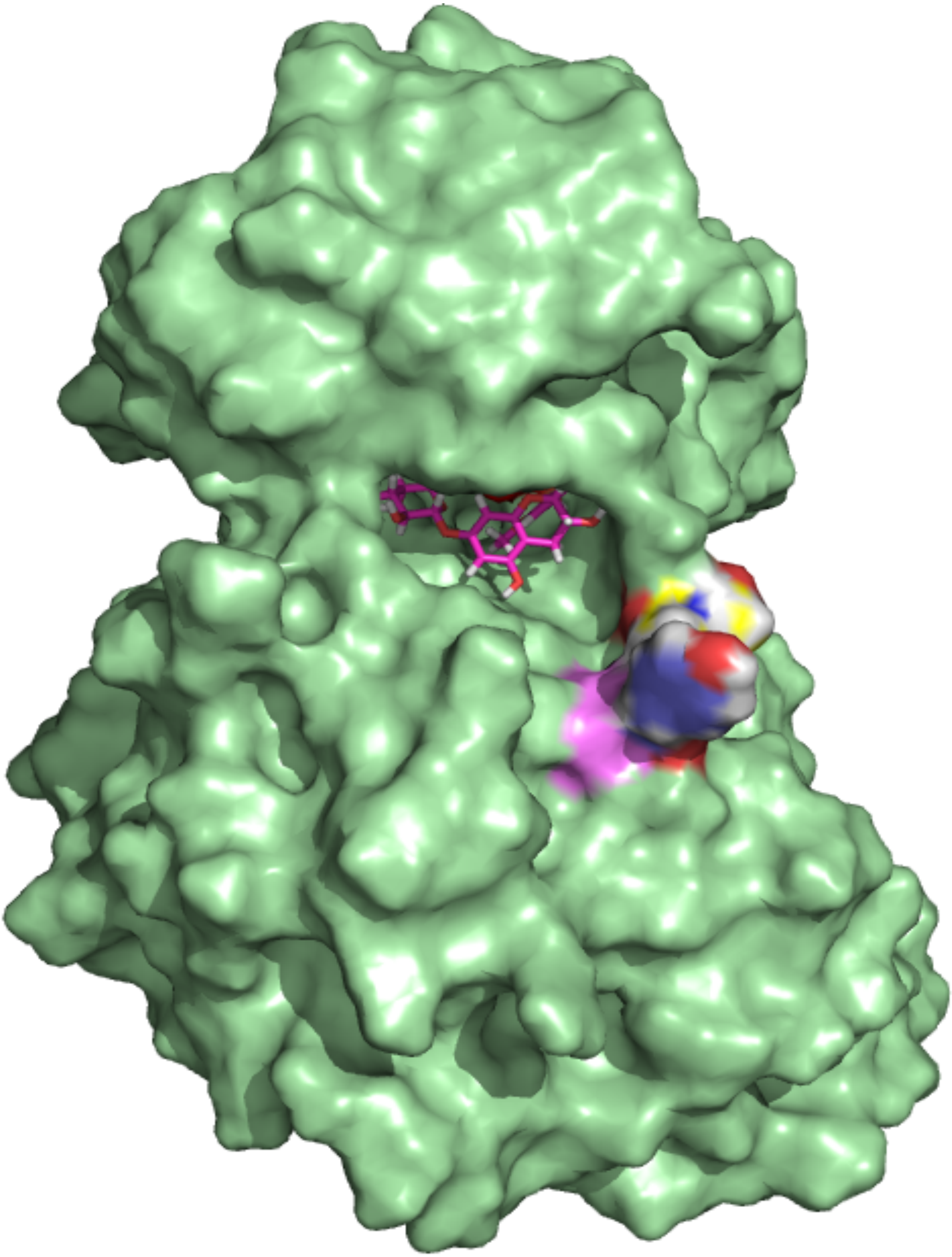

Supplemental Figure S9: Flow chart describing workflow of DNA methylation analysis.

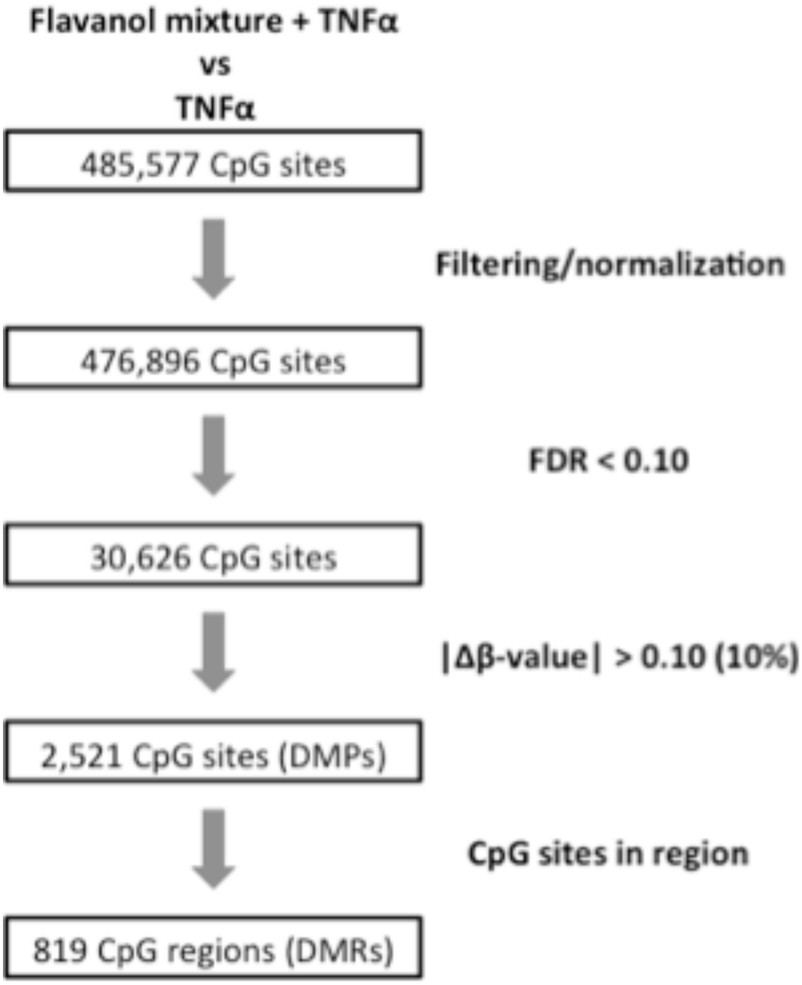

Supplemental Table S1: Site map property values and Dscore ranks for the p38 MAPK (Pdb id: 4F9Y)

**Entry Name (Top ranked)**	**SiteScore**	**Dscore**	**Volume**	**Important residues of site map predicted binding site**
**4F9Yproteinsitemap_site_1**	1.119	1.166	535.08	**Chain A:** 71,171,104,74,105,75,51,106,52,107,53,108,152,109,178,30,154,179,31,155,32 33,157,35,36,111,**180**,38,112,**181**,84,**183**,86,165,167,168,169
**4F9Yproteinsitemap_site_2**	1.107	1.149	168.41	**Chain A:** 191,294,192,296,195,196,250,197,251,198,252,255,232,259,236,242,246,291,292,249,293
**4F9Yproteinsitemap_site_3**	0.96	0.991	300.12	**Chain A**: 351,129,75,106,76,107,78,79,132,133,80,316,81,136,82,83,84,85,86,87,163,165,347,348,349
**4F9Yproteinsitemap_site_4**	0.892	0.797	184.19	**Chain A**: 147,200,328,202,149,193,70,170,196,172,197,173,74,198,174,175,323,325,145,146,327
**4F9Yproteinsitemap_site_5**	0.793	0.77	218.83	**Chain A**: 23,48,24,93,94,350,351,353,50,105,5,6,8,87,88,89,345,346,43,348,45,46,90,21,91

Supplemental table S2 : Genes differentially expressed by TNF in endothelial cells

Gene Symbol	Description	FC_mean
A2M	alpha-2-macroglobulin [Source:HGNC Symbol;Acc:HGNC:7]	-1.295993
AAAS	aladin WD repeat nucleoporin [Source:HGNC Symbol;Acc:HGNC:13666]	1.2532548
AADAC	arylacetamide deacetylase [Source:HGNC Symbol;Acc:HGNC:17]	1.2346227
AADAT	aminoadipate aminotransferase [Source:HGNC Symbol;Acc:HGNC:17929]	1.1930633
AASDH	aminoadipate-semialdehyde dehydrogenase [Source:HGNC Symbol;Acc:HGNC:23993]	1.2574718
AASDHPPT	lipate-semialdehyde dehydrogenase-phosphopantetheinyl transferase [Source:HGNC Symbol;Acc:HGN(	1.4679608
AASS	aminoadipate-semialdehyde synthase [Source:HGNC Symbol;Acc:HGNC:17366]	1.3107485
ABAT	4-aminobutyrate aminotransferase [Source:HGNC Symbol;Acc:HGNC:23]	1.1162478
ABCA1	ATP binding cassette subfamily A member 1 [Source:HGNC Symbol;Acc:HGNC:29]	-1.23403375
ABCA2	ATP binding cassette subfamily A member 2 [Source:HGNC Symbol;Acc:HGNC:32]	1.1289406
ABCA7	ATP binding cassette subfamily A member 7 [Source:HGNC Symbol;Acc:HGNC:37]	1.0874552
ABCB10	ATP binding cassette subfamily B member 10 [Source:HGNC Symbol;Acc:HGNC:41]	1.1608669
ABCB6	TP binding cassette subfamily B member 6 (Langereis blood group) [Source:HGNC Symbol;Acc:HGNC:47	1.390825
ABCB9	ATP binding cassette subfamily B member 9 [Source:HGNC Symbol;Acc:HGNC:50]	1.1446027
ABCC1	ATP binding cassette subfamily C member 1 [Source:HGNC Symbol;Acc:HGNC:51]	1.1526519
ABCC10	ATP binding cassette subfamily C member 10 [Source:HGNC Symbol;Acc:HGNC:52]	-1.2424773
ABCC13	ATP binding cassette subfamily C member 13 (pseudogene) [Source:HGNC Symbol;Acc:HGNC:16022]	1.1197207
ABCC4	ATP binding cassette subfamily C member 4 [Source:HGNC Symbol;Acc:HGNC:55]	-1.1140414
ABCC9	ATP binding cassette subfamily C member 9 [Source:HGNC Symbol;Acc:HGNC:60]	1.1195009
ABCD1	ATP binding cassette subfamily D member 1 [Source:HGNC Symbol;Acc:HGNC:61]	1.3689568
ABCD2	ATP binding cassette subfamily D member 2 [Source:HGNC Symbol;Acc:HGNC:66]	1.111039
ABCD3	ATP binding cassette subfamily D member 3 [Source:HGNC Symbol;Acc:HGNC:67]	-1.2039258
ABCD4	ATP binding cassette subfamily D member 4 [Source:HGNC Symbol;Acc:HGNC:68]	-1.0678612
ABCF1	ATP binding cassette subfamily F member 1 [Source:HGNC Symbol;Acc:HGNC:70]	-1.6139004
ABCG1	ATP binding cassette subfamily G member 1 [Source:HGNC Symbol;Acc:HGNC:73]	-2.2921114
ABHD15	abhydrolase domain containing 15 [Source:HGNC Symbol;Acc:HGNC:26971]	1.284376
ABHD6	abhydrolase domain containing 6 [Source:HGNC Symbol;Acc:HGNC:21398]	1.1806154
ABL2	ABL proto-oncogene 2, non-receptor tyrosine kinase [Source:HGNC Symbol;Acc:HGNC:77]	-1.3186278
ABR	active BCR-related [Source:HGNC Symbol;Acc:HGNC:81]	-1.40119205
ABRA	actin binding Rho activating protein [Source:HGNC Symbol;Acc:HGNC:30655]	1.1575011
ABT1	activator of basal transcription 1 [Source:HGNC Symbol;Acc:HGNC:17369]	-1.1810149
ABTB2	ankyrin repeat and BTB domain containing 2 [Source:HGNC Symbol;Acc:HGNC:23842]	-2.4537582
ACACB	acetyl-CoA carboxylase beta [Source:HGNC Symbol;Acc:HGNC:85]	1.1930591
ACAD9	acyl-CoA dehydrogenase family member 9 [Source:HGNC Symbol;Acc:HGNC:21497]	-1.1158419
ACAN	aggrecan [Source:HGNC Symbol;Acc:HGNC:319]	-1.0911885
ACBD7	acyl-CoA binding domain containing 7 [Source:HGNC Symbol;Acc:HGNC:17715]	-1.0948354
ACO1	aconitase 1 [Source:HGNC Symbol;Acc:HGNC:117]	-1.3130834
ACOT1	acyl-CoA thioesterase 1 [Source:HGNC Symbol;Acc:HGNC:33128]	-1.2267752
ACOT13	acyl-CoA thioesterase 13 [Source:HGNC Symbol;Acc:HGNC:20999]	1.078244
ACOT4	acyl-CoA thioesterase 4 [Source:HGNC Symbol;Acc:HGNC:19748]	-1.2729762
ACP2	acid phosphatase 2, lysosomal [Source:HGNC Symbol;Acc:HGNC:123]	-1.1342864
ACSS1	acyl-CoA synthetase short-chain family member 1 [Source:HGNC Symbol;Acc:HGNC:16091]	-1.2038226
ACTL9	actin like 9 [Source:HGNC Symbol;Acc:HGNC:28494]	-1.087294
ACTN4	actinin alpha 4 [Source:HGNC Symbol;Acc:HGNC:166]	1.1720281
ACTR3B	ARP3 actin related protein 3 homolog B [Source:HGNC Symbol;Acc:HGNC:17256]	1.0671289
ACTR3C	ARP3 actin-related protein 3 homolog C [Source:HGNC Symbol;Acc:HGNC:37282]	-1.1309211
ACTR8	ARP8 actin-related protein 8 homolog [Source:HGNC Symbol;Acc:HGNC:14672]	1.1478686
ACTRT2	actin related protein T2 [Source:HGNC Symbol;Acc:HGNC:24026]	1.1693397
ACVRL1	activin A receptor like type 1 [Source:HGNC Symbol;Acc:HGNC:175]	-1.2399968
ACYP1	acylphosphatase 1 [Source:HGNC Symbol;Acc:HGNC:179]	1.2725029
ADAL	adenosine deaminase like [Source:HGNC Symbol;Acc:HGNC:31853]	1.1278715
ADAM15	ADAM metallopeptidase domain 15 [Source:HGNC Symbol;Acc:HGNC:193]	1.1599994
ADAM19	ADAM metallopeptidase domain 19 [Source:HGNC Symbol;Acc:HGNC:197]	-1.232372
ADAM28	ADAM metallopeptidase domain 28 [Source:HGNC Symbol;Acc:HGNC:206]	-1.0864991
ADAM33	ADAM metallopeptidase domain 33 [Source:HGNC Symbol;Acc:HGNC:15478]	-1.1671146
ADAM8	ADAM metallopeptidase domain 8 [Source:HGNC Symbol;Acc:HGNC:215]	-1.6214597
ADAMTS10	ADAM metallopeptidase with thrombospondin type 1 motif 10 [Source:HGNC Symbol;Acc:HGNC:13201	-1.4123731
ADAMTS13	ADAM metallopeptidase with thrombospondin type 1 motif 13 [Source:HGNC Symbol;Acc:HGNC:1366]	1.3268881
ADAMTS14	ADAM metallopeptidase with thrombospondin type 1 motif 14 [Source:HGNC Symbol;Acc:HGNC:14899	-1.1410211
ADAMTS15	ADAM metallopeptidase with thrombospondin type 1 motif 15 [Source:HGNC Symbol;Acc:HGNC:16305	-1.0838786
ADAMTS16	ADAM metallopeptidase with thrombospondin type 1 motif 16 [Source:HGNC Symbol;Acc:HGNC:17108	-1.1026491
ADAMTS17	ADAM metallopeptidase with thrombospondin type 1 motif 17 [Source:HGNC Symbol;Acc:HGNC:17109	-1.0519661
ADAMTS19	ADAM metallopeptidase with thrombospondin type 1 motif 19 [Source:HGNC Symbol;Acc:HGNC:17111	-1.2041692
ADAMTS20	ADAM metallopeptidase with thrombospondin type 1 motif 20 [Source:HGNC Symbol;Acc:HGNC:17178	1.0593333
ADAMTS4	ADAM metallopeptidase with thrombospondin type 1 motif 4 [Source:HGNC Symbol;Acc:HGNC:220]	-2.1937551
ADAMTS7	ADAM metallopeptidase with thrombospondin type 1 motif 7 [Source:HGNC Symbol;Acc:HGNC:223]	1.2491819
ADAMTS9	ADAM metallopeptidase with thrombospondin type 1 motif 9 [Source:HGNC Symbol;Acc:HGNC:13202]	-1.2689196
ADAMTSL1	ADAMTS like 1 [Source:HGNC Symbol;Acc:HGNC:14632]	1.1048983
ADAMTSL2	ADAMTS like 2 [Source:HGNC Symbol;Acc:HGNC:14631]	-1.1920514
ADAMTSL3	ADAMTS like 3 [Source:HGNC Symbol;Acc:HGNC:14633]	1.0863901
ADAMTSL4	ADAMTS like 4 [Source:HGNC Symbol;Acc:HGNC:19706]	-1.1369889
ADAP1	ArfGAP with dual PH domains 1 [Source:HGNC Symbol;Acc:HGNC:16486]	-1.2168728
ADARB1	adenosine deaminase, RNA specific B1 [Source:HGNC Symbol;Acc:HGNC:226]	1.2508187
ADAT2	adenosine deaminase, tRNA specific 2 [Source:HGNC Symbol;Acc:HGNC:21172]	-1.4781575
ADC	antizyme inhibitor 2	-1.1236424
ADCK3	coenzyme Q8A	-1.187149
ADCK4	coenzyme Q8B	1.2047751
ADCY4	adenylate cyclase 4 [Source:HGNC Symbol;Acc:HGNC:235]	-1.2466023
ADCYAP1R1	ADCYAP receptor type I [Source:HGNC Symbol;Acc:HGNC:242]	-1.1824162
ADH1A	alcohol dehydrogenase 1A (class I), alpha polypeptide [Source:HGNC Symbol;Acc:HGNC:249]	1.0718597
ADH5	alcohol dehydrogenase 5 (class III), chi polypeptide [Source:HGNC Symbol;Acc:HGNC:253]	1.2090188
ADH6	alcohol dehydrogenase 6 (class V) [Source:HGNC Symbol;Acc:HGNC:255]	1.11222915
ADK	adenosine kinase [Source:HGNC Symbol;Acc:HGNC:257]	1.1093177

ADM	adrenomedullin [Source:HGNC Symbol;Acc:HGNC:259]	1.7993667
ADNP	activity dependent neuroprotector homeobox [Source:HGNC Symbol;Acc:HGNC:15766]	1.3423048
ADO	2-aminoethanethiol dioxygenase [Source:HGNC Symbol;Acc:HGNC:23506]	1.2320936
ADPRH	ADP-ribosylarginine hydrolase [Source:HGNC Symbol;Acc:HGNC:269]	-1.3570276
ADPRHL2	ADP-ribosylhydrolase like 2 [Source:HGNC Symbol;Acc:HGNC:21304]	-1.3022674
ADRA1B	adrenoceptor alpha 1B [Source:HGNC Symbol;Acc:HGNC:278]	1.5798137
ADRA1D	adrenoceptor alpha 1D [Source:HGNC Symbol;Acc:HGNC:280]	1.1034441
ADRB1	adrenoceptor beta 1 [Source:HGNC Symbol;Acc:HGNC:285]	1.4576823
ADRB3	adrenoceptor beta 3 [Source:HGNC Symbol;Acc:HGNC:288]	-1.1099663
ADSS	adenylosuccinate synthase [Source:HGNC Symbol;Acc:HGNC:292]	1.4793819
AFAP1L1	actin filament associated protein 1 like 1 [Source:HGNC Symbol;Acc:HGNC:26714]	-1.1824666
AFF2	AF4/FMR2 family member 2 [Source:HGNC Symbol;Acc:HGNC:3776]	-1.119592
AFMID	arylformamidase [Source:HGNC Symbol;Acc:HGNC:20910]	1.0874438
AGAP1	ArfGAP with GTPase domain, ankyrin repeat and PH domain 1 [Source:HGNC Symbol;Acc:HGNC:16922]	-1.1519274
AGAP2	ArfGAP with GTPase domain, ankyrin repeat and PH domain 2 [Source:HGNC Symbol;Acc:HGNC:16921]	1.2381144
AGAP3	ArfGAP with GTPase domain, ankyrin repeat and PH domain 3 [Source:HGNC Symbol;Acc:HGNC:16923]	1.0960298
AGER	advanced glycosylation end-product specific receptor [Source:HGNC Symbol;Acc:HGNC:320]	1.12015
AGFG1	ArfGAP with FG repeats 1 [Source:HGNC Symbol;Acc:HGNC:5175]	-1.0805941
AGPAT1	1-acylglycerol-3-phosphate O-acyltransferase 1 [Source:HGNC Symbol;Acc:HGNC:324]	1.2362485
AGPAT3	1-acylglycerol-3-phosphate O-acyltransferase 3 [Source:HGNC Symbol;Acc:HGNC:326]	-1.1128453
AGPAT6	glycerol-3-phosphate acyltransferase 4	-1.3704916
AGPAT9	lysophosphatidylcholine acyltransferase 1	1.5517836
AGPS	alkylglycerone phosphate synthase [Source:HGNC Symbol;Acc:HGNC:327]	1.3030115
AGRN	agrin [Source:HGNC Symbol;Acc:HGNC:329]	-1.450005
AGSK1	golgin A2 pseudogene 10	1.1697268
AGXT2	alanine--glyoxylate aminotransferase 2 [Source:HGNC Symbol;Acc:HGNC:14412]	1.1135411
AGXT2L2	5-phosphohydroxy-L-lysine phospho-lyase	-1.1433324
AHCYL1	adenosylhomocysteinase like 1 [Source:HGNC Symbol;Acc:HGNC:344]	-1.2469922
AHDC1	AT-hook DNA binding motif containing 1 [Source:HGNC Symbol;Acc:HGNC:25230]	-2.3617847
AHR	aryl hydrocarbon receptor [Source:HGNC Symbol;Acc:HGNC:348]	1.2535999
AHRR	aryl-hydrocarbon receptor repressor [Source:HGNC Symbol;Acc:HGNC:346]	-1.3035115
AHSA2	1, activator of heat shock 90kDa protein ATPase homolog 2 (yeast) [Source:HGNC Symbol;Acc:HGNC:20	1.2514814
AIF1	allograft inflammatory factor 1 [Source:HGNC Symbol;Acc:HGNC:352]	-1.1333927
AIF1L	allograft inflammatory factor 1 like [Source:HGNC Symbol;Acc:HGNC:28904]	-1.1981606
AIG1	androgen induced 1 [Source:HGNC Symbol;Acc:HGNC:21607]	-1.16302
AIM2	absent in melanoma 2 [Source:HGNC Symbol;Acc:HGNC:357]	1.1194698
AIMP1	›acyl tRNA synthetase complex interacting multifunctional protein 1 [Source:HGNC Symbol;Acc:HGNC:1	1.2470869
AIPL1	aryl hydrocarbon receptor interacting protein like 1 [Source:HGNC Symbol;Acc:HGNC:359]	1.0694968
AK3	adenylate kinase 3 [Source:HGNC Symbol;Acc:HGNC:17376]	1.2033451
AKAP17A	A-kinase anchoring protein 17A [Source:HGNC Symbol;Acc:HGNC:18783]	-1.1262562
AKAP2	A-kinase anchoring protein 2 [Source:HGNC Symbol;Acc:HGNC:372]	-1.1450834
AKAP8	A-kinase anchoring protein 8 [Source:HGNC Symbol;Acc:HGNC:378]	1.1265244
AKD1	adenylate kinase 9	-1.1273862
AKIRIN1	akirin 1 [Source:HGNC Symbol;Acc:HGNC:25744]	-1.2322332
AKIRIN2	akirin 2 [Source:HGNC Symbol;Acc:HGNC:21407]	-1.2217575
AKNA	AT-hook transcription factor [Source:HGNC Symbol;Acc:HGNC:24108]	-1.1536517
AKT1	AKT serine/threonine kinase 1 [Source:HGNC Symbol;Acc:HGNC:391]	1.134962
AKT1S1	AKT1 substrate 1 [Source:HGNC Symbol;Acc:HGNC:28426]	1.2209451
ALAD	aminolevulinate dehydratase [Source:HGNC Symbol;Acc:HGNC:395]	-1.1701211
ALAS1	5'-aminolevulinate synthase 1 [Source:HGNC Symbol;Acc:HGNC:396]	-1.4068297
ALDH1A3	aldehyde dehydrogenase 1 family member A3 [Source:HGNC Symbol;Acc:HGNC:409]	-1.1932596
ALDH3B1	aldehyde dehydrogenase 3 family member B1 [Source:HGNC Symbol;Acc:HGNC:410]	-1.1427356
ALDH7A1	aldehyde dehydrogenase 7 family member A1 [Source:HGNC Symbol;Acc:HGNC:877]	1.0728403
ALG2	ALG2, alpha-1,3/1,6-mannosyltransferase [Source:HGNC Symbol;Acc:HGNC:23159]	-1.3034068
ALG6	ALG6, alpha-1,3-glucosyltransferase [Source:HGNC Symbol;Acc:HGNC:23157]	1.1889843
ALG8	ALG8, alpha-1,3-glucosyltransferase [Source:HGNC Symbol;Acc:HGNC:23161]	-1.238926
ALKBH1	alkB homolog 1, histone H2A dioxygenase [Source:HGNC Symbol;Acc:HGNC:17911]	-1.1765679
ALKBH5	alkB homolog 5, RNA demethylase [Source:HGNC Symbol;Acc:HGNC:25996]	1.1238653
ALOX15	arachidonate 15-lipoxygenase [Source:HGNC Symbol;Acc:HGNC:433]	-1.1474167
ALPK1	alpha kinase 1 [Source:HGNC Symbol;Acc:HGNC:20917]	-1.1219901
ALPK3	alpha kinase 3 [Source:HGNC Symbol;Acc:HGNC:17574]	-1.1666327
ALPP	alkaline phosphatase, placental [Source:HGNC Symbol;Acc:HGNC:439]	-1.147836
ALS2	ALS2, alsin Rho guanine nucleotide exchange factor [Source:HGNC Symbol;Acc:HGNC:443]	1.1730324
ALS2CL	ALS2 C-terminal like [Source:HGNC Symbol;Acc:HGNC:20605]	-1.6029048
ALX1	ALX homeobox 1 [Source:HGNC Symbol;Acc:HGNC:1494]	1.4387568
AMBN	ameloblastin [Source:HGNC Symbol;Acc:HGNC:452]	-1.1854475
AMBRA1	autophagy and beclin 1 regulator 1 [Source:HGNC Symbol;Acc:HGNC:25990]	-1.2786399
AMD1	adenosylmethionine decarboxylase 1 [Source:HGNC Symbol;Acc:HGNC:457]	1.7828411
AMELX	amelogenin, X-linked [Source:HGNC Symbol;Acc:HGNC:461]	-1.090403
AMHR2	anti-Mullerian hormone receptor type 2 [Source:HGNC Symbol;Acc:HGNC:465]	-1.0604098
AMICA1	junction adhesion molecule like	1.0956241
AMMECR1	ental retardation, midface hypoplasia and elliptocytosis chromosomal region gene 1 [Source:HGNC Syn	-1.3696743
AMOT	angiomotin [Source:HGNC Symbol;Acc:HGNC:17810]	1.100864
AMOTL2	angiomotin like 2 [Source:HGNC Symbol;Acc:HGNC:17812]	1.0528084
ANAPC13	anaphase promoting complex subunit 13 [Source:HGNC Symbol;Acc:HGNC:24540]	1.1336751
ANGPT1	angiopoietin 1 [Source:HGNC Symbol;Acc:HGNC:484]	1.1155918
ANGPTL4	angiopoietin like 4 [Source:HGNC Symbol;Acc:HGNC:16039]	1.940775
ANGPTL5	angiopoietin like 5 [Source:HGNC Symbol;Acc:HGNC:19705]	1.1307821
ANK1	ankyrin 1 [Source:HGNC Symbol;Acc:HGNC:492]	-1.1840773
ANKFY1	ankyrin repeat and FYVE domain containing 1 [Source:HGNC Symbol;Acc:HGNC:20763]	-1.2805209
ANKH	ANKH inorganic pyrophosphate transport regulator [Source:HGNC Symbol;Acc:HGNC:15492]	1.1340746
ANKK1	ankyrin repeat and kinase domain containing 1 [Source:HGNC Symbol;Acc:HGNC:21027]	-1.1366614
ANKLE2	ankyrin repeat and LEM domain containing 2 [Source:HGNC Symbol;Acc:HGNC:29101]	-1.5028042
ANKRD11	ankyrin repeat domain 11 [Source:HGNC Symbol;Acc:HGNC:21316]	1.1364534
ANKRD13D	ankyrin repeat domain 13D [Source:HGNC Symbol;Acc:HGNC:27880]	1.1911414
ANKRD20A2	ankyrin repeat domain 20 family member A2 [Source:HGNC Symbol;Acc:HGNC:31979]	1.32748785

ANKRD20A9P	ankyrin repeat domain 20 family member A9, pseudogene [Source:HGNC Symbol;Acc:HGNC:42023]	1.1827317
ANKRD23	ankyrin repeat domain 23 [Source:HGNC Symbol;Acc:HGNC:24470]	-1.1301494
ANKRD26	ankyrin repeat domain 26 [Source:HGNC Symbol;Acc:HGNC:29186]	1.1245983
ANKRD27	ankyrin repeat domain 27 [Source:HGNC Symbol;Acc:HGNC:25310]	-1.39345035
ANKRD28	ankyrin repeat domain 28 [Source:HGNC Symbol;Acc:HGNC:29024]	1.4562614
ANKRD30BL	ankyrin repeat domain 30B-like [Source:HGNC Symbol;Acc:HGNC:35167]	-1.0791416
ANKRD30BP2	ankyrin repeat domain 30B pseudogene 2 [Source:HGNC Symbol;Acc:HGNC:16620]	1.1488045
ANKRD33B	ankyrin repeat domain 33B [Source:HGNC Symbol;Acc:HGNC:35240]	-1.3332356
ANKRD35	ankyrin repeat domain 35 [Source:HGNC Symbol;Acc:HGNC:26323]	1.4072214
ANKS1A	ankyrin repeat and sterile alpha motif domain containing 1A [Source:HGNC Symbol;Acc:HGNC:20961]	1.2282481
ANKS3	ankyrin repeat and sterile alpha motif domain containing 3 [Source:HGNC Symbol;Acc:HGNC:29422]	1.1742829
ANO10	anoctamin 10 [Source:HGNC Symbol;Acc:HGNC:25519]	-1.1463088
ANO2	anoctamin 2 [Source:HGNC Symbol;Acc:HGNC:1183]	1.2004906
ANO4	anoctamin 4 [Source:HGNC Symbol;Acc:HGNC:23837]	1.1382886
ANO8	anoctamin 8 [Source:HGNC Symbol;Acc:HGNC:29329]	1.0660795
ANO9	anoctamin 9 [Source:HGNC Symbol;Acc:HGNC:20679]	-1.5962213
ANP32A	acidic nuclear phosphoprotein 32 family member A [Source:HGNC Symbol;Acc:HGNC:13233]	1.1652434
ANP32D	acidic nuclear phosphoprotein 32 family member D [Source:HGNC Symbol;Acc:HGNC:16676]	1.2596217
ANTXRL	anthrax toxin receptor-like [Source:HGNC Symbol;Acc:HGNC:27277]	1.1517117
ANXA1	annexin A1 [Source:HGNC Symbol;Acc:HGNC:533]	1.6887745
ANXA11	annexin A11 [Source:HGNC Symbol;Acc:HGNC:535]	-1.1404953
AOC2	amine oxidase, copper containing 2 [Source:HGNC Symbol;Acc:HGNC:549]	1.2001185
AOX1	aldehyde oxidase 1 [Source:HGNC Symbol;Acc:HGNC:553]	-1.1059792
AP1AR	daptor related protein complex 1 associated regulatory protein [Source:HGNC Symbol;Acc:HGNC:2880	1.3158482
AP1B1	adaptor related protein complex 1 beta 1 subunit [Source:HGNC Symbol;Acc:HGNC:554]	1.1775063
AP1S1	adaptor related protein complex 1 sigma 1 subunit [Source:HGNC Symbol;Acc:HGNC:559]	1.3231467
AP1S2	adaptor related protein complex 1 sigma 2 subunit [Source:HGNC Symbol;Acc:HGNC:560]	1.2719432
AP1S3	adaptor related protein complex 1 sigma 3 subunit [Source:HGNC Symbol;Acc:HGNC:18971]	-1.1543322
AP2A1	adaptor related protein complex 2 alpha 1 subunit [Source:HGNC Symbol;Acc:HGNC:561]	1.3732315
AP3D1	adaptor related protein complex 3 delta 1 subunit [Source:HGNC Symbol;Acc:HGNC:568]	-1.247038
AP3M2	adaptor related protein complex 3 mu 2 subunit [Source:HGNC Symbol;Acc:HGNC:570]	1.2480363
AP3S2	adaptor related protein complex 3 sigma 2 subunit [Source:HGNC Symbol;Acc:HGNC:571]	-1.2104422
AP4B1	adaptor related protein complex 4 beta 1 subunit [Source:HGNC Symbol;Acc:HGNC:572]	1.1523722
APBA3	amyloid beta precursor protein binding family A member 3 [Source:HGNC Symbol;Acc:HGNC:580]	-1.5584203
APCDD1	APC down-regulated 1 [Source:HGNC Symbol;Acc:HGNC:15718]	1.4026713
APLN	apelin [Source:HGNC Symbol;Acc:HGNC:16665]	1.8412083
APOA4	apolipoprotein A4 [Source:HGNC Symbol;Acc:HGNC:602]	-1.0561597
APOA5	apolipoprotein A5 [Source:HGNC Symbol;Acc:HGNC:17288]	-1.1250774
APOB	apolipoprotein B [Source:HGNC Symbol;Acc:HGNC:603]	1.0794657
APOBEC3B	apolipoprotein B mRNA editing enzyme catalytic subunit 3B [Source:HGNC Symbol;Acc:HGNC:17352]	-1.1613922
APOBEC3C	apolipoprotein B mRNA editing enzyme catalytic subunit 3C [Source:HGNC Symbol;Acc:HGNC:17353]	1.1980796
APOBEC3G	apolipoprotein B mRNA editing enzyme catalytic subunit 3G [Source:HGNC Symbol;Acc:HGNC:17357]	-1.2448577
APOC3	apolipoprotein C3 [Source:HGNC Symbol;Acc:HGNC:610]	1.0713217
APOC4	apolipoprotein C4 [Source:HGNC Symbol;Acc:HGNC:611]	-1.0723298
APOL2	apolipoprotein L2 [Source:HGNC Symbol;Acc:HGNC:619]	-2.0766153
APOL3	apolipoprotein L3 [Source:HGNC Symbol;Acc:HGNC:14868]	-2.6731124
APOL4	apolipoprotein L4 [Source:HGNC Symbol;Acc:HGNC:14867]	-2.1201253
APOL5	apolipoprotein L5 [Source:HGNC Symbol;Acc:HGNC:14869]	1.242814
APOL6	apolipoprotein L6 [Source:HGNC Symbol;Acc:HGNC:14870]	-1.84664745
APPL2	otein, phosphotyrosine interacting with PH domain and leucine zipper 2 [Source:HGNC Symbol;Acc:HG	1.1289085
AQP2	aquaporin 2 [Source:HGNC Symbol;Acc:HGNC:634]	-1.1617019
AQP6	aquaporin 6 [Source:HGNC Symbol;Acc:HGNC:639]	-1.0822448
AQP7P1	aquaporin 7 pseudogene 1 [Source:HGNC Symbol;Acc:HGNC:32048]	-1.1152661
AQP9	aquaporin 9 [Source:HGNC Symbol;Acc:HGNC:643]	1.0620369
ARAP3	ArfGAP with RhoGAP domain, ankyrin repeat and PH domain 3 [Source:HGNC Symbol;Acc:HGNC:24097	1.2647666
ARF3	ADP ribosylation factor 3 [Source:HGNC Symbol;Acc:HGNC:654]	-1.23373055
ARFRP1	ADP ribosylation factor related protein 1 [Source:HGNC Symbol;Acc:HGNC:662]	-1.3252905
ARG1	arginase 1 [Source:HGNC Symbol;Acc:HGNC:663]	1.0760386
ARGFX	arginine-fifty homeobox [Source:HGNC Symbol;Acc:HGNC:30146]	-1.1389344
ARHGAP12	Rho GTPase activating protein 12 [Source:HGNC Symbol;Acc:HGNC:16348]	1.5420827
ARHGAP18	Rho GTPase activating protein 18 [Source:HGNC Symbol;Acc:HGNC:21035]	2.1440883
ARHGAP19	Rho GTPase activating protein 19 [Source:HGNC Symbol;Acc:HGNC:23724]	-1.13050945
ARHGAP20	Rho GTPase activating protein 20 [Source:HGNC Symbol;Acc:HGNC:18357]	1.1477607
ARHGAP22	Rho GTPase activating protein 22 [Source:HGNC Symbol;Acc:HGNC:30320]	-1.3118076
ARHGAP24	Rho GTPase activating protein 24 [Source:HGNC Symbol;Acc:HGNC:25361]	1.4855636
ARHGAP25	Rho GTPase activating protein 25 [Source:HGNC Symbol;Acc:HGNC:28951]	-1.2206204
ARHGAP31	Rho GTPase activating protein 31 [Source:HGNC Symbol;Acc:HGNC:29216]	-1.9456972
ARHGAP32	Rho GTPase activating protein 32 [Source:HGNC Symbol;Acc:HGNC:17399]	-1.3427783
ARHGAP35	Rho GTPase activating protein 35 [Source:HGNC Symbol;Acc:HGNC:4591]	1.1647383
ARHGAP36	Rho GTPase activating protein 36 [Source:HGNC Symbol;Acc:HGNC:26388]	1.0910088
ARHGAP5	Rho GTPase activating protein 5 [Source:HGNC Symbol;Acc:HGNC:675]	1.1743265
ARHGAP6	Rho GTPase activating protein 6 [Source:HGNC Symbol;Acc:HGNC:676]	-1.1165998
ARHGEF10L	Rho guanine nucleotide exchange factor 10 like [Source:HGNC Symbol;Acc:HGNC:25540]	-1.32104525
ARHGEF12	Rho guanine nucleotide exchange factor 12 [Source:HGNC Symbol;Acc:HGNC:14193]	1.1657554
ARHGEF15	Rho guanine nucleotide exchange factor 15 [Source:HGNC Symbol;Acc:HGNC:15590]	-1.285653
ARHGEF16	Rho guanine nucleotide exchange factor 16 [Source:HGNC Symbol;Acc:HGNC:15515]	-1.709941
ARHGEF17	Rho guanine nucleotide exchange factor 17 [Source:HGNC Symbol;Acc:HGNC:21726]	1.210676
ARHGEF26	Rho guanine nucleotide exchange factor 26 [Source:HGNC Symbol;Acc:HGNC:24490]	1.1715456
ARHGEF37	Rho guanine nucleotide exchange factor 37 [Source:HGNC Symbol;Acc:HGNC:34430]	1.2946156
ARHGEF38	Rho guanine nucleotide exchange factor 38 [Source:HGNC Symbol;Acc:HGNC:25968]	1.103191
ARHGEF40	Rho guanine nucleotide exchange factor 40 [Source:HGNC Symbol;Acc:HGNC:25516]	-3.796834
ARHGEF6	Rac/Cdc42 guanine nucleotide exchange factor 6 [Source:HGNC Symbol;Acc:HGNC:685]	1.0740124
ARHGEF7	Rho guanine nucleotide exchange factor 7 [Source:HGNC Symbol;Acc:HGNC:15607]	-1.17362945
ARID1B	AT-rich interaction domain 1B [Source:HGNC Symbol;Acc:HGNC:18040]	-1.1709648
ARID3B	AT-rich interaction domain 3B [Source:HGNC Symbol;Acc:HGNC:14350]	-1.6698583
ARID5A	AT-rich interaction domain 5A [Source:HGNC Symbol;Acc:HGNC:17361]	-1.5348624

ARL17B	ADP ribosylation factor like GTPase 17B [Source:HGNC Symbol;Acc:HGNC:32387]	-1.2318981
ARL2BP	ADP ribosylation factor like GTPase 2 binding protein [Source:HGNC Symbol;Acc:HGNC:17146]	-1.3956972
ARL4A	ADP ribosylation factor like GTPase 4A [Source:HGNC Symbol;Acc:HGNC:695]	-1.2045508
ARL4C	ADP ribosylation factor like GTPase 4C [Source:HGNC Symbol;Acc:HGNC:698]	-1.4356825
ARL5A	ADP ribosylation factor like GTPase 5A [Source:HGNC Symbol;Acc:HGNC:696]	1.4994498
ARL5C	ADP ribosylation factor like GTPase 5C [Source:HGNC Symbol;Acc:HGNC:31111]	-1.1733562
ARL6IP5	ADP ribosylation factor like GTPase 6 interacting protein 5 [Source:HGNC Symbol;Acc:HGNC:16937]	-1.3173611
ARL8A	ADP ribosylation factor like GTPase 8A [Source:HGNC Symbol;Acc:HGNC:25192]	1.1317644
ARMC1	armadillo repeat containing 1 [Source:HGNC Symbol;Acc:HGNC:17684]	1.3632983
ARMC4	armadillo repeat containing 4 [Source:HGNC Symbol;Acc:HGNC:25583]	1.1648505
ARMC8	armadillo repeat containing 8 [Source:HGNC Symbol;Acc:HGNC:24999]	1.3305542
ARMC9	armadillo repeat containing 9 [Source:HGNC Symbol;Acc:HGNC:20730]	-1.1706734
ARMCX6	armadillo repeat containing, X-linked 6 [Source:HGNC Symbol;Acc:HGNC:26094]	-1.0861462
ARNT2	aryl hydrocarbon receptor nuclear translocator 2 [Source:HGNC Symbol;Acc:HGNC:16876]	-1.1736228
ARNTL	aryl hydrocarbon receptor nuclear translocator like [Source:HGNC Symbol;Acc:HGNC:701]	-1.0527279
ARPC1A	actin related protein 2/3 complex subunit 1A [Source:HGNC Symbol;Acc:HGNC:703]	1.2115438
ARPM1	actin related protein T3	-1.0826732
ARPP19	cAMP regulated phosphoprotein 19 [Source:HGNC Symbol;Acc:HGNC:16967]	1.322349
ARRB1	arrestin beta 1 [Source:HGNC Symbol;Acc:HGNC:711]	-1.6861901
ARRDC2	arrestin domain containing 2 [Source:HGNC Symbol;Acc:HGNC:25225]	-1.4012861
ARRDC3	arrestin domain containing 3 [Source:HGNC Symbol;Acc:HGNC:29263]	1.2088153
ARSD	arylsulfatase D [Source:HGNC Symbol;Acc:HGNC:717]	-1.2720516
ART1	ADP-ribosyltransferase 1 [Source:HGNC Symbol;Acc:HGNC:723]	-1.0607938
ART4	ADP-ribosyltransferase 4 (Dombrock blood group) [Source:HGNC Symbol;Acc:HGNC:726]	-2.2783897
ART5	ADP-ribosyltransferase 5 [Source:HGNC Symbol;Acc:HGNC:24049]	-1.1922164
ASAH2	N-acylsphingosine amidohydrolase 2 [Source:HGNC Symbol;Acc:HGNC:18860]	1.1645775
ASB13	ankyrin repeat and SOCS box containing 13 [Source:HGNC Symbol;Acc:HGNC:19765]	1.423418
ASB17	ankyrin repeat and SOCS box containing 17 [Source:HGNC Symbol;Acc:HGNC:19769]	1.1808741
ASB7	ankyrin repeat and SOCS box containing 7 [Source:HGNC Symbol;Acc:HGNC:17182]	-1.2424747
ASGR1	asialoglycoprotein receptor 1 [Source:HGNC Symbol;Acc:HGNC:742]	-1.1838837
ASIC1	acid sensing ion channel subunit 1	1.1748358
ASIP	agouti signaling protein [Source:HGNC Symbol;Acc:HGNC:745]	-1.0596741
ASNA1	arsA arsenite transporter, ATP-binding, homolog 1 (bacterial) [Source:HGNC Symbol;Acc:HGNC:752]	1.2656978
ASNSD1	asparagine synthetase domain containing 1 [Source:HGNC Symbol;Acc:HGNC:24910]	1.5138249
ASPH	aspartate beta-hydroxylase [Source:HGNC Symbol;Acc:HGNC:757]	1.3792418
ASPHD2	aspartate beta-hydroxylase domain containing 2 [Source:HGNC Symbol;Acc:HGNC:30437]	-1.2865282
ASPSCR1	ASPSCR1, UBX domain containing tether for SLC2A4 [Source:HGNC Symbol;Acc:HGNC:13825]	-1.0610112
ASS1	argininosuccinate synthase 1 [Source:HGNC Symbol;Acc:HGNC:758]	-1.07619
ASTN2	astrotactin 2 [Source:HGNC Symbol;Acc:HGNC:17021]	1.1385942
ATAD2	ATPase family, AAA domain containing 2 [Source:HGNC Symbol;Acc:HGNC:30123]	1.5684117
ATAD3B	ATPase family, AAA domain containing 3B [Source:HGNC Symbol;Acc:HGNC:24007]	1.1467226
ATF1	activating transcription factor 1 [Source:HGNC Symbol;Acc:HGNC:783]	1.1707162
ATF3	activating transcription factor 3 [Source:HGNC Symbol;Acc:HGNC:785]	-2.5273751
ATF5	activating transcription factor 5 [Source:HGNC Symbol;Acc:HGNC:790]	-1.2639709
ATF6	activating transcription factor 6 [Source:HGNC Symbol;Acc:HGNC:791]	-1.2513235
ATF7IP2	activating transcription factor 7 interacting protein 2 [Source:HGNC Symbol;Acc:HGNC:20397]	1.3261163
ATG13	autophagy related 13 [Source:HGNC Symbol;Acc:HGNC:29091]	-1.2528231
ATG14	autophagy related 14 [Source:HGNC Symbol;Acc:HGNC:19962]	1.1349314
ATG16L2	autophagy related 16 like 2 [Source:HGNC Symbol;Acc:HGNC:25464]	-1.2765108
ATG4A	autophagy related 4A cysteine peptidase [Source:HGNC Symbol;Acc:HGNC:16489]	1.3133653
ATG7	autophagy related 7 [Source:HGNC Symbol;Acc:HGNC:16935]	-1.2043744
ATMIN	ATM interactor [Source:HGNC Symbol;Acc:HGNC:29034]	1.2705523
ATP11B	ATPase phospholipid transporting 11B (putative) [Source:HGNC Symbol;Acc:HGNC:13553]	1.1881589
ATP1A4	ATPase Na+/K+ transporting subunit alpha 4 [Source:HGNC Symbol;Acc:HGNC:14073]	-1.1978463
ATP1B1	ATPase Na+/K+ transporting subunit beta 1 [Source:HGNC Symbol;Acc:HGNC:804]	-1.9287975
ATP2A1	ATPase sarcoplasmic/endoplasmic reticulum Ca2+ transporting 1 [Source:HGNC Symbol;Acc:HGNC:811]	1.1828079
ATP2B4	ATPase plasma membrane Ca2+ transporting 4 [Source:HGNC Symbol;Acc:HGNC:817]	-1.15855565
ATP4B	ATPase H+/K+ transporting beta subunit [Source:HGNC Symbol;Acc:HGNC:820]	1.1430212
ATP5D	synthase, H+ transporting, mitochondrial F1 complex, delta subunit [Source:HGNC Symbol;Acc:HGNC:[illegible]	-1.1162703
ATP5H	TP synthase, H+ transporting, mitochondrial Fo complex subunit D [Source:HGNC Symbol;Acc:HGNC:84[illegible]	1.0855707
ATP5I	TP synthase, H+ transporting, mitochondrial Fo complex subunit E [Source:HGNC Symbol;Acc:HGNC:84[illegible]	-1.1508979
ATP6AP1	ATPase H+ transporting accessory protein 1 [Source:HGNC Symbol;Acc:HGNC:868]	-1.0536419
ATP6AP1L	ATPase H+ transporting accessory protein 1 like [Source:HGNC Symbol;Acc:HGNC:28091]	1.2490357
ATP6V0A1	ATPase H+ transporting V0 subunit a1 [Source:HGNC Symbol;Acc:HGNC:865]	1.1078784
ATP6V0D1	ATPase H+ transporting V0 subunit d1 [Source:HGNC Symbol;Acc:HGNC:13724]	-1.1738416
ATP6V0E1	ATPase H+ transporting V0 subunit e1 [Source:HGNC Symbol;Acc:HGNC:863]	-1.0720892
ATP6V1C1	ATPase H+ transporting V1 subunit C1 [Source:HGNC Symbol;Acc:HGNC:856]	1.3770074
ATP6V1E1	ATPase H+ transporting V1 subunit E1 [Source:HGNC Symbol;Acc:HGNC:857]	-1.0743483
ATP6V1G2	ATPase H+ transporting V1 subunit G2 [Source:HGNC Symbol;Acc:HGNC:862]	-1.1512762
ATP6V1G3	ATPase H+ transporting V1 subunit G3 [Source:HGNC Symbol;Acc:HGNC:18265]	1.1707712
ATP7A	ATPase copper transporting alpha [Source:HGNC Symbol;Acc:HGNC:869]	1.3624587
ATP8B2	ATPase phospholipid transporting 8B2 [Source:HGNC Symbol;Acc:HGNC:13534]	-1.2168714
ATP8B3	ATPase phospholipid transporting 8B3 [Source:HGNC Symbol;Acc:HGNC:13535]	1.1277881
ATP8B5P	ATPase phospholipid transporting 8B5, pseudogene [Source:HGNC Symbol;Acc:HGNC:27245]	1.1684906
ATP9B	ATPase phospholipid transporting 9B (putative) [Source:HGNC Symbol;Acc:HGNC:13541]	-1.1588206
ATPAF1-AS1	testis expressed 38	1.136972
ATPIF1	ATPase inhibitory factor 1 [Source:HGNC Symbol;Acc:HGNC:871]	1.1442711
ATRNL1	attractin like 1 [Source:HGNC Symbol;Acc:HGNC:29063]	-1.1539842
ATXN2L	ataxin 2 like [Source:HGNC Symbol;Acc:HGNC:31326]	-1.23803355
ATXN3L	ataxin 3 like [Source:HGNC Symbol;Acc:HGNC:24173]	1.0700265
ATXN7L1	ataxin 7 like 1 [Source:HGNC Symbol;Acc:HGNC:22210]	-1.0865167
AURKB	aurora kinase B [Source:HGNC Symbol;Acc:HGNC:11390]	-1.1043131
AVEN	apoptosis and caspase activation inhibitor [Source:HGNC Symbol;Acc:HGNC:13509]	-1.1246307
AZGP1P1	alpha-2-glycoprotein 1, zinc-binding pseudogene 1 [Source:HGNC Symbol;Acc:HGNC:911]	1.0757635
AZI2	5-azacytidine induced 2 [Source:HGNC Symbol;Acc:HGNC:24002]	1.1558623
B4GALNT3	beta-1,4-N-acetyl-galactosaminyltransferase 3 [Source:HGNC Symbol;Acc:HGNC:24137]	1.0571241

B4GALT1	beta-1,4-galactosyltransferase 1 [Source:HGNC Symbol;Acc:HGNC:924]	-1.9519022
B4GALT2	beta-1,4-galactosyltransferase 2 [Source:HGNC Symbol;Acc:HGNC:925]	1.2084926
B4GALT4	beta-1,4-galactosyltransferase 4 [Source:HGNC Symbol;Acc:HGNC:927]	-1.1900249
B4GALT5	beta-1,4-galactosyltransferase 5 [Source:HGNC Symbol;Acc:HGNC:928]	-1.7326291
BAALC	brain and acute leukemia, cytoplasmic [Source:HGNC Symbol;Acc:HGNC:14333]	-1.1608124
BABAM1	BRISC and BRCA1 A complex member 1 [Source:HGNC Symbol;Acc:HGNC:25008]	1.1690655
BACE1	beta-secretase 1 [Source:HGNC Symbol;Acc:HGNC:933]	-1.2863574
BAD	BCL2 associated agonist of cell death [Source:HGNC Symbol;Acc:HGNC:936]	-1.1132538
BAG2	BCL2 associated athanogene 2 [Source:HGNC Symbol;Acc:HGNC:938]	1.2176627
BAG4	BCL2 associated athanogene 4 [Source:HGNC Symbol;Acc:HGNC:940]	1.1364249
BAGE	B melanoma antigen	-1.1304287
BAIAP2	BAI1 associated protein 2 [Source:HGNC Symbol;Acc:HGNC:947]	1.12370395
BAMBI	BMP and activin membrane bound inhibitor [Source:HGNC Symbol;Acc:HGNC:30251]	-2.4658017
BANF1	barrier to autointegration factor 1 [Source:HGNC Symbol;Acc:HGNC:17397]	1.0583279
BANP	BTG3 associated nuclear protein [Source:HGNC Symbol;Acc:HGNC:13450]	-1.0843782
BARD1	BRCA1 associated RING domain 1 [Source:HGNC Symbol;Acc:HGNC:952]	1.3892603
BATF	basic leucine zipper ATF-like transcription factor [Source:HGNC Symbol;Acc:HGNC:958]	-1.0835506
BATF3	basic leucine zipper ATF-like transcription factor 3 [Source:HGNC Symbol;Acc:HGNC:28915]	-1.5118812
BAX	BCL2 associated X, apoptosis regulator [Source:HGNC Symbol;Acc:HGNC:959]	1.4119049
BAZ1B	bromodomain adjacent to zinc finger domain 1B [Source:HGNC Symbol;Acc:HGNC:961]	1.4825696
BBC3	BCL2 binding component 3 [Source:HGNC Symbol;Acc:HGNC:17868]	-1.987462
BBS1	Bardet-Biedl syndrome 1 [Source:HGNC Symbol;Acc:HGNC:966]	-1.1490573
BBS4	Bardet-Biedl syndrome 4 [Source:HGNC Symbol;Acc:HGNC:969]	-1.0916607
BBS5	Bardet-Biedl syndrome 5 [Source:HGNC Symbol;Acc:HGNC:970]	1.137248
BCAN	brevican [Source:HGNC Symbol;Acc:HGNC:23059]	-1.0868853
BCAT1	branched chain amino acid transaminase 1 [Source:HGNC Symbol;Acc:HGNC:976]	2.0375526
BCDIN3D	BCDIN3 domain containing RNA methyltransferase [Source:HGNC Symbol;Acc:HGNC:27050]	1.328748
BCL11A	B-cell CLL/lymphoma 11A [Source:HGNC Symbol;Acc:HGNC:13221]	1.09127815
BCL2	BCL2, apoptosis regulator [Source:HGNC Symbol;Acc:HGNC:990]	1.1681875
BCL2A1	BCL2 related protein A1 [Source:HGNC Symbol;Acc:HGNC:991]	-2.484996
BCL2L1	BCL2 like 1 [Source:HGNC Symbol;Acc:HGNC:992]	1.4653032
BCL2L10	BCL2 like 10 [Source:HGNC Symbol;Acc:HGNC:993]	1.1074464
BCL2L11	BCL2 like 11 [Source:HGNC Symbol;Acc:HGNC:994]	-1.705509
BCL2L15	BCL2 like 15 [Source:HGNC Symbol;Acc:HGNC:33624]	-1.1152124
BCL2L2	BCL2 like 2 [Source:HGNC Symbol;Acc:HGNC:995]	-1.2008884
BCL3	B-cell CLL/lymphoma 3 [Source:HGNC Symbol;Acc:HGNC:998]	-3.5352957
BCL6	B-cell CLL/lymphoma 6 [Source:HGNC Symbol;Acc:HGNC:1001]	-1.1438493
BCL6B	B-cell CLL/lymphoma 6B [Source:HGNC Symbol;Acc:HGNC:1002]	-2.321052
BCL9L	B-cell CLL/lymphoma 9-like [Source:HGNC Symbol;Acc:HGNC:23688]	-1.22514
BCO2	beta-carotene oxygenase 2 [Source:HGNC Symbol;Acc:HGNC:18503]	1.1213925
BCOR	BCL6 corepressor [Source:HGNC Symbol;Acc:HGNC:20893]	-1.28321335
BCR	BCR, RhoGEF and GTPase activating protein [Source:HGNC Symbol;Acc:HGNC:1014]	-1.1858041
BCRP2	breakpoint cluster region pseudogene 2 [Source:HGNC Symbol;Acc:HGNC:1015]	-1.283097
BDH1	3-hydroxybutyrate dehydrogenase, type 1 [Source:HGNC Symbol;Acc:HGNC:1027]	1.4033644
BDKRB1	bradykinin receptor B1 [Source:HGNC Symbol;Acc:HGNC:1029]	-1.1134189
BDKRB2	bradykinin receptor B2 [Source:HGNC Symbol;Acc:HGNC:1030]	-1.9707477
BDNF	brain derived neurotrophic factor [Source:HGNC Symbol;Acc:HGNC:1033]	1.41973505
BEGAIN	brain enriched guanylate kinase associated [Source:HGNC Symbol;Acc:HGNC:24163]	-1.4803464
BEND7	BEN domain containing 7 [Source:HGNC Symbol;Acc:HGNC:23514]	-1.2252234
BEST1	bestrophin 1 [Source:HGNC Symbol;Acc:HGNC:12703]	1.0681973
BET3L	trafficking protein particle complex 3 like	-1.0716788
BEX2	brain expressed X-linked 2 [Source:HGNC Symbol;Acc:HGNC:30933]	-1.2434828
BEX4	brain expressed X-linked 4 [Source:HGNC Symbol;Acc:HGNC:25475]	-1.0661619
BFSP1	beaded filament structural protein 1 [Source:HGNC Symbol;Acc:HGNC:1040]	1.1312419
BGN	biglycan [Source:HGNC Symbol;Acc:HGNC:1044]	1.8416222
BHLHA15	basic helix-loop-helix family member a15 [Source:HGNC Symbol;Acc:HGNC:22265]	-1.0989157
BHLHE22	basic helix-loop-helix family member e22 [Source:HGNC Symbol;Acc:HGNC:11963]	1.0699666
BHLHE40	basic helix-loop-helix family member e40 [Source:HGNC Symbol;Acc:HGNC:1046]	-1.6723416
BHLHE41	basic helix-loop-helix family member e41 [Source:HGNC Symbol;Acc:HGNC:16617]	-1.7422471
BICC1	BicC family RNA binding protein 1 [Source:HGNC Symbol;Acc:HGNC:19351]	-1.1615374
BID	BH3 interacting domain death agonist [Source:HGNC Symbol;Acc:HGNC:1050]	-1.1472667
BIK	BCL2 interacting killer [Source:HGNC Symbol;Acc:HGNC:1051]	-1.8745261
BIRC3	baculoviral IAP repeat containing 3 [Source:HGNC Symbol;Acc:HGNC:591]	-8.632974
BIVM	basic, immunoglobulin-like variable motif containing [Source:HGNC Symbol;Acc:HGNC:16034]	1.4044118
BLM	Bloom syndrome RecQ like helicase [Source:HGNC Symbol;Acc:HGNC:1058]	1.358415
BLOC1S2	biogenesis of lysosomal organelles complex 1 subunit 2 [Source:HGNC Symbol;Acc:HGNC:20984]	1.2011064
BMF	Bcl2 modifying factor [Source:HGNC Symbol;Acc:HGNC:24132]	-1.6515329
BMI1	BMI1 proto-oncogene, polycomb ring finger [Source:HGNC Symbol;Acc:HGNC:1066]	1.1267371
BMP2	bone morphogenetic protein 2 [Source:HGNC Symbol;Acc:HGNC:1069]	-2.7585454
BMP2K	BMP2 inducible kinase [Source:HGNC Symbol;Acc:HGNC:18041]	1.1979887
BMP3	bone morphogenetic protein 3 [Source:HGNC Symbol;Acc:HGNC:1070]	1.0753007
BMP4	bone morphogenetic protein 4 [Source:HGNC Symbol;Acc:HGNC:1071]	1.3945035
BNIP1	BCL2 interacting protein 1 [Source:HGNC Symbol;Acc:HGNC:1082]	-1.332247
BNIP3L	BCL2 interacting protein 3 like [Source:HGNC Symbol;Acc:HGNC:1085]	1.6140515
BOD1	biorientation of chromosomes in cell division 1 [Source:HGNC Symbol;Acc:HGNC:25114]	-1.1513828
BOK	BOK, BCL2 family apoptosis regulator [Source:HGNC Symbol;Acc:HGNC:1087]	-1.3769368
BPGM	bisphosphoglycerate mutase [Source:HGNC Symbol;Acc:HGNC:1093]	-1.39353
BPNT1	3'(2'), 5'-bisphosphate nucleotidase 1 [Source:HGNC Symbol;Acc:HGNC:1096]	-1.1938287
BRCA2	BRCA2, DNA repair associated [Source:HGNC Symbol;Acc:HGNC:1101]	1.0850444
BRCC3	BRCA1/BRCA2-containing complex subunit 3 [Source:HGNC Symbol;Acc:HGNC:24185]	1.5193005
BRD3	bromodomain containing 3 [Source:HGNC Symbol;Acc:HGNC:1104]	-1.0745567
BRD4	bromodomain containing 4 [Source:HGNC Symbol;Acc:HGNC:13575]	-1.2179859
BRDT	bromodomain testis associated [Source:HGNC Symbol;Acc:HGNC:1105]	1.195707
BRF1	1, RNA polymerase III transcription initiation factor 90 kDa subunit [Source:HGNC Symbol;Acc:HGNC:11	1.3878995
BRP44L	mitochondrial pyruvate carrier 1	-1.3508238
BRPF1	bromodomain and PHD finger containing 1 [Source:HGNC Symbol;Acc:HGNC:14255]	-1.2391176

BRPF3	bromodomain and PHD finger containing 3 [Source:HGNC Symbol;Acc:HGNC:14256]	-1.1694245
BSDC1	BSD domain containing 1 [Source:HGNC Symbol;Acc:HGNC:25501]	-1.251499833
BSN	bassoon presynaptic cytomatrix protein [Source:HGNC Symbol;Acc:HGNC:1117]	1.0774626
BST1	bone marrow stromal cell antigen 1 [Source:HGNC Symbol;Acc:HGNC:1118]	-1.2324651
BTBD1	BTB domain containing 1 [Source:HGNC Symbol;Acc:HGNC:1120]	1.3135189
BTBD17	BTB domain containing 17 [Source:HGNC Symbol;Acc:HGNC:33758]	-1.1142645
BTBD2	BTB domain containing 2 [Source:HGNC Symbol;Acc:HGNC:15504]	1.4108995
BTBD3	BTB domain containing 3 [Source:HGNC Symbol;Acc:HGNC:15854]	1.1184211
BTBD7	BTB domain containing 7 [Source:HGNC Symbol;Acc:HGNC:18269]	1.2374692
BTBD9	BTB domain containing 9 [Source:HGNC Symbol;Acc:HGNC:21228]	-1.155976
BTG2	BTG anti-proliferation factor 2 [Source:HGNC Symbol;Acc:HGNC:1131]	-1.2182752
BTK	Bruton tyrosine kinase [Source:HGNC Symbol;Acc:HGNC:1133]	1.1353592
BTN2A1	butyrophilin subfamily 2 member A1 [Source:HGNC Symbol;Acc:HGNC:1136]	-1.39907195
BTN2A2	butyrophilin subfamily 2 member A2 [Source:HGNC Symbol;Acc:HGNC:1137]	-2.1564157
BTN2A3	butyrophilin subfamily 2 member A3, pseudogene	-1.4954653
BTN3A1	butyrophilin subfamily 3 member A1 [Source:HGNC Symbol;Acc:HGNC:1138]	-1.3293505
BTN3A2	butyrophilin subfamily 3 member A2 [Source:HGNC Symbol;Acc:HGNC:1139]	-1.23984015
BTRC	beta-transducin repeat containing E3 ubiquitin protein ligase [Source:HGNC Symbol;Acc:HGNC:1144]	1.1071655
BUB1	BUB1 mitotic checkpoint serine/threonine kinase [Source:HGNC Symbol;Acc:HGNC:1148]	1.3283908
BUB1B	BUB1 mitotic checkpoint serine/threonine kinase B [Source:HGNC Symbol;Acc:HGNC:1149]	1.3888223
BUD31	BUD31 homolog [Source:HGNC Symbol;Acc:HGNC:29629]	-1.1293468
BZRAP1	TSPO associated protein 1	1.1130446
BZW1	basic leucine zipper and W2 domains 1 [Source:HGNC Symbol;Acc:HGNC:18380]	1.5280015
CA13	carbonic anhydrase 13 [Source:HGNC Symbol;Acc:HGNC:14914]	-1.5966207
CA5BP1	carbonic anhydrase 5B pseudogene 1 [Source:HGNC Symbol;Acc:HGNC:29544]	1.0731641
CA7	carbonic anhydrase 7 [Source:HGNC Symbol;Acc:HGNC:1381]	-1.0907911
CA8	carbonic anhydrase 8 [Source:HGNC Symbol;Acc:HGNC:1382]	-1.2134297
CAB39	calcium binding protein 39 [Source:HGNC Symbol;Acc:HGNC:20292]	-1.3557761
CABLES1	Cdk5 and Abl enzyme substrate 1 [Source:HGNC Symbol;Acc:HGNC:25097]	1.8719213
CABS1	calcium binding protein, spermatid associated 1 [Source:HGNC Symbol;Acc:HGNC:30710]	1.1244646
CABYR	calcium binding tyrosine phosphorylation regulated [Source:HGNC Symbol;Acc:HGNC:15569]	-1.551064
CACNA2D3	calcium voltage-gated channel auxiliary subunit alpha2delta 3 [Source:HGNC Symbol;Acc:HGNC:15460]	1.1084201
CACNG3	calcium voltage-gated channel auxiliary subunit gamma 3 [Source:HGNC Symbol;Acc:HGNC:1407]	-1.130643
CACYBP	calcyclin binding protein [Source:HGNC Symbol;Acc:HGNC:30423]	1.1253649
CADPS	calcium dependent secretion activator [Source:HGNC Symbol;Acc:HGNC:1426]	-1.208708
CADPS2	calcium dependent secretion activator 2 [Source:HGNC Symbol;Acc:HGNC:16018]	-1.1154019
CALB2	calbindin 2 [Source:HGNC Symbol;Acc:HGNC:1435]	-1.222056
CALCA	calcitonin related polypeptide alpha [Source:HGNC Symbol;Acc:HGNC:1437]	-1.0827203
CALCRL	calcitonin receptor like receptor [Source:HGNC Symbol;Acc:HGNC:16709]	1.731325
CALHM2	calcium homeostasis modulator 2 [Source:HGNC Symbol;Acc:HGNC:23493]	1.814719
CALM1	calmodulin 1 [Source:HGNC Symbol;Acc:HGNC:1442]	-1.1997496
CALM2	calmodulin 2 [Source:HGNC Symbol;Acc:HGNC:1445]	1.3150295
CALM3	calmodulin 3 [Source:HGNC Symbol;Acc:HGNC:1449]	1.155265
CAMK2D	calcium/calmodulin dependent protein kinase II delta [Source:HGNC Symbol;Acc:HGNC:1462]	1.10263
CAMK2G	calcium/calmodulin dependent protein kinase II gamma [Source:HGNC Symbol;Acc:HGNC:1463]	1.1799176
CAMKK2	calcium/calmodulin dependent protein kinase kinase 2 [Source:HGNC Symbol;Acc:HGNC:1470]	-1.4172559
CAMTA2	calmodulin binding transcription activator 2 [Source:HGNC Symbol;Acc:HGNC:18807]	1.1752386
CAND1	cullin associated and neddylation dissociated 1 [Source:HGNC Symbol;Acc:HGNC:30688]	-1.2486426
CAPN12	calpain 12 [Source:HGNC Symbol;Acc:HGNC:13249]	-1.0674342
CAPN14	calpain 14 [Source:HGNC Symbol;Acc:HGNC:16664]	1.1103263
CAPS	calcyphosine [Source:HGNC Symbol;Acc:HGNC:1487]	1.1143286
CAPZA2	capping actin protein of muscle Z-line alpha subunit 2 [Source:HGNC Symbol;Acc:HGNC:1490]	1.5588872
CAPZB	capping actin protein of muscle Z-line beta subunit [Source:HGNC Symbol;Acc:HGNC:1491]	1.1272553
CARD10	caspase recruitment domain family member 10 [Source:HGNC Symbol;Acc:HGNC:16422]	1.6181695
CARD11	caspase recruitment domain family member 11 [Source:HGNC Symbol;Acc:HGNC:16393]	-1.0986353
CARD6	caspase recruitment domain family member 6 [Source:HGNC Symbol;Acc:HGNC:16394]	1.2187955
CARD8	caspase recruitment domain family member 8 [Source:HGNC Symbol;Acc:HGNC:17057]	1.9096254
CARD9	caspase recruitment domain family member 9 [Source:HGNC Symbol;Acc:HGNC:16391]	-1.2662306
CARM1	coactivator associated arginine methyltransferase 1 [Source:HGNC Symbol;Acc:HGNC:23393]	-1.0642453
CARS	cysteinyl-tRNA synthetase [Source:HGNC Symbol;Acc:HGNC:1493]	-1.2984945
CARS2	cysteinyl-tRNA synthetase 2, mitochondrial (putative) [Source:HGNC Symbol;Acc:HGNC:25695]	1.0972837
CASC3	cancer susceptibility candidate 3 [Source:HGNC Symbol;Acc:HGNC:17040]	-1.1128179
CASC5	kinetochore scaffold 1	1.0782192
CASK	calcium/calmodulin dependent serine protein kinase [Source:HGNC Symbol;Acc:HGNC:1497]	1.0503185
CASP10	caspase 10 [Source:HGNC Symbol;Acc:HGNC:1500]	-1.1915936
CASP2	caspase 2 [Source:HGNC Symbol;Acc:HGNC:1503]	1.2206196
CASS4	Cas scaffolding protein family member 4 [Source:HGNC Symbol;Acc:HGNC:15878]	-1.2246492
CAT	catalase [Source:HGNC Symbol;Acc:HGNC:1516]	-1.1889257
CBLL1	Cbl proto-oncogene like 1 [Source:HGNC Symbol;Acc:HGNC:21225]	-1.321054
CBLN2	cerebellin 2 precursor [Source:HGNC Symbol;Acc:HGNC:1544]	-1.5886966
CBLN3	cerebellin 3 precursor [Source:HGNC Symbol;Acc:HGNC:20146]	-2.8616996
CBR3	carbonyl reductase 3 [Source:HGNC Symbol;Acc:HGNC:1549]	-2.6813447
CBWD5	COBW domain containing 5 [Source:HGNC Symbol;Acc:HGNC:24584]	1.21022255
CBX1	chromobox 1 [Source:HGNC Symbol;Acc:HGNC:1551]	1.2934592
CBX2	chromobox 2 [Source:HGNC Symbol;Acc:HGNC:1552]	-1.2486842
CBX3	chromobox 3 [Source:HGNC Symbol;Acc:HGNC:1553]	1.4430742
CBX8	chromobox 8 [Source:HGNC Symbol;Acc:HGNC:15962]	1.272581
CC2D1A	coiled-coil and C2 domain containing 1A [Source:HGNC Symbol;Acc:HGNC:30237]	1.1964161
CC2D1B	coiled-coil and C2 domain containing 1B [Source:HGNC Symbol;Acc:HGNC:29386]	-1.1948502
CC2D2A	coiled-coil and C2 domain containing 2A [Source:HGNC Symbol;Acc:HGNC:29253]	1.1013218
CC2D2B	coiled-coil and C2 domain containing 2B [Source:HGNC Symbol;Acc:HGNC:31666]	1.0635815
CCBE1	collagen and calcium binding EGF domains 1 [Source:HGNC Symbol;Acc:HGNC:29426]	1.0840408
CCBP2	atypical chemokine receptor 4	-1.0664968
CCDC102A	coiled-coil domain containing 102A [Source:HGNC Symbol;Acc:HGNC:28097]	-2.074409
CCDC102B	coiled-coil domain containing 102B [Source:HGNC Symbol;Acc:HGNC:26295]	1.06603725
CCDC114	coiled-coil domain containing 114 [Source:HGNC Symbol;Acc:HGNC:26560]	1.1013131

CCDC117	coiled-coil domain containing 117 [Source:HGNC Symbol;Acc:HGNC:26599]	1.268229
CCDC122	coiled-coil domain containing 122 [Source:HGNC Symbol;Acc:HGNC:26478]	1.0873597
CCDC125	coiled-coil domain containing 125 [Source:HGNC Symbol;Acc:HGNC:28924]	1.262252
CCDC127	coiled-coil domain containing 127 [Source:HGNC Symbol;Acc:HGNC:30520]	-1.2325678
CCDC130	coiled-coil domain containing 130 [Source:HGNC Symbol;Acc:HGNC:28118]	-1.5990587
CCDC135	dynein regulatory complex subunit 7	-1.0597217
CCDC136	coiled-coil domain containing 136 [Source:HGNC Symbol;Acc:HGNC:22225]	1.0772107
CCDC147	cilia and flagella associated protein 58	1.1415145
CCDC158	coiled-coil domain containing 158 [Source:HGNC Symbol;Acc:HGNC:26374]	-1.2003424
CCDC165	microtubule crosslinking factor 1	1.4233621
CCDC17	coiled-coil domain containing 17 [Source:HGNC Symbol;Acc:HGNC:26574]	-1.1202221
CCDC18	coiled-coil domain containing 18 [Source:HGNC Symbol;Acc:HGNC:30370]	1.3375928
CCDC34	coiled-coil domain containing 34 [Source:HGNC Symbol;Acc:HGNC:25079]	1.1961079
CCDC43	coiled-coil domain containing 43 [Source:HGNC Symbol;Acc:HGNC:26472]	1.2030979
CCDC48	EF-hand and coiled-coil domain containing 1	1.4252007
CCDC57	coiled-coil domain containing 57 [Source:HGNC Symbol;Acc:HGNC:27564]	-1.1053814
CCDC6	coiled-coil domain containing 6 [Source:HGNC Symbol;Acc:HGNC:18782]	1.3472356
CCDC66	coiled-coil domain containing 66 [Source:HGNC Symbol;Acc:HGNC:27709]	1.2598557
CCDC68	coiled-coil domain containing 68 [Source:HGNC Symbol;Acc:HGNC:24350]	1.0944777
CCDC69	coiled-coil domain containing 69 [Source:HGNC Symbol;Acc:HGNC:24487]	-1.5884665
CCDC7	coiled-coil domain containing 7 [Source:HGNC Symbol;Acc:HGNC:26533]	-1.2096258
CCDC72	translation machinery associated 7 homolog	-1.1235614
CCDC77	coiled-coil domain containing 77 [Source:HGNC Symbol;Acc:HGNC:28203]	1.3094445
CCDC87	coiled-coil domain containing 87 [Source:HGNC Symbol;Acc:HGNC:25579]	-1.1664268
CCDC88B	coiled-coil domain containing 88B [Source:HGNC Symbol;Acc:HGNC:26757]	1.2318027
CCDC9	coiled-coil domain containing 9 [Source:HGNC Symbol;Acc:HGNC:24560]	-1.4933194
CCDC90A	mitochondrial calcium uniporter regulator 1	-1.1656632
CCDC92	coiled-coil domain containing 92 [Source:HGNC Symbol;Acc:HGNC:29563]	-1.1962309
CCDC94	coiled-coil domain containing 94 [Source:HGNC Symbol;Acc:HGNC:25518]	-1.2415799
CCIN	calicin [Source:HGNC Symbol;Acc:HGNC:1568]	-1.7423061
CCL1	C-C motif chemokine ligand 1 [Source:HGNC Symbol;Acc:HGNC:10609]	-1.1278946
CCL11	C-C motif chemokine ligand 11 [Source:HGNC Symbol;Acc:HGNC:10610]	-1.2430913
CCL16	C-C motif chemokine ligand 16 [Source:HGNC Symbol;Acc:HGNC:10614]	1.1499176
CCL17	C-C motif chemokine ligand 17 [Source:HGNC Symbol;Acc:HGNC:10615]	-1.4459815
CCL20	C-C motif chemokine ligand 20 [Source:HGNC Symbol;Acc:HGNC:10619]	-16.189316
CCL27	C-C motif chemokine ligand 27 [Source:HGNC Symbol;Acc:HGNC:10626]	1.0958531
CCL3L3	C-C motif chemokine ligand 3 like 3 [Source:HGNC Symbol;Acc:HGNC:30554]	-1.1380721
CCL4	C-C motif chemokine ligand 4 [Source:HGNC Symbol;Acc:HGNC:10630]	-1.1003438
CCL5	C-C motif chemokine ligand 5 [Source:HGNC Symbol;Acc:HGNC:10632]	-1.4648074
CCL7	C-C motif chemokine ligand 7 [Source:HGNC Symbol;Acc:HGNC:10634]	-1.6453692
CCND3	cyclin D3 [Source:HGNC Symbol;Acc:HGNC:1585]	1.4880316
CCNDBP1	cyclin D1 binding protein 1 [Source:HGNC Symbol;Acc:HGNC:1587]	1.051129
CCNE1	cyclin E1 [Source:HGNC Symbol;Acc:HGNC:1589]	1.1546259
CCNE2	cyclin E2 [Source:HGNC Symbol;Acc:HGNC:1590]	1.38179315
CCNG1	cyclin G1 [Source:HGNC Symbol;Acc:HGNC:1592]	1.1649122
CCNH	cyclin H [Source:HGNC Symbol;Acc:HGNC:1594]	1.3526845
CCNJL	cyclin J like [Source:HGNC Symbol;Acc:HGNC:25876]	-1.2287031
CCNK	cyclin K [Source:HGNC Symbol;Acc:HGNC:1596]	-1.1897988
CCNO	cyclin O [Source:HGNC Symbol;Acc:HGNC:18576]	-1.3829342
CCR10	C-C motif chemokine receptor 10 [Source:HGNC Symbol;Acc:HGNC:4474]	-1.685271
CCRL2	C-C motif chemokine receptor like 2 [Source:HGNC Symbol;Acc:HGNC:1612]	-1.3415862
CCRN4L	nocturnin	-1.8238574
CCT3	chaperonin containing TCP1 subunit 3 [Source:HGNC Symbol;Acc:HGNC:1616]	-1.233663
CCT8L2	chaperonin containing TCP1 subunit 8 like 2 [Source:HGNC Symbol;Acc:HGNC:15553]	1.1354038
CD14	CD14 molecule [Source:HGNC Symbol;Acc:HGNC:1628]	-1.2066199
CD164	CD164 molecule [Source:HGNC Symbol;Acc:HGNC:1632]	-1.0877779
CD19	CD19 molecule [Source:HGNC Symbol;Acc:HGNC:1633]	-1.0952975
CD2	CD2 molecule [Source:HGNC Symbol;Acc:HGNC:1639]	-1.1567912
CD200R1	CD200 receptor 1 [Source:HGNC Symbol;Acc:HGNC:24235]	1.116739
CD247	CD247 molecule [Source:HGNC Symbol;Acc:HGNC:1677]	1.2551904
CD274	CD274 molecule [Source:HGNC Symbol;Acc:HGNC:17635]	-1.2411003
CD300E	CD300e molecule [Source:HGNC Symbol;Acc:HGNC:28874]	-1.1087426
CD300LD	CD300 molecule like family member d [Source:HGNC Symbol;Acc:HGNC:16848]	1.0899926
CD34	CD34 molecule [Source:HGNC Symbol;Acc:HGNC:1662]	-1.3446221
CD3EAP	CD3e molecule associated protein [Source:HGNC Symbol;Acc:HGNC:24219]	1.2518119
CD40	CD40 molecule [Source:HGNC Symbol;Acc:HGNC:11919]	-1.50629995
CD46	CD46 molecule [Source:HGNC Symbol;Acc:HGNC:6953]	1.1097405
CD47	CD47 molecule [Source:HGNC Symbol;Acc:HGNC:1682]	-1.4642583
CD52	CD52 molecule [Source:HGNC Symbol;Acc:HGNC:1804]	1.0710961
CD58	CD58 molecule [Source:HGNC Symbol;Acc:HGNC:1688]	-1.4621334
CD69	CD69 molecule [Source:HGNC Symbol;Acc:HGNC:1694]	-3.9310902
CD70	CD70 molecule [Source:HGNC Symbol;Acc:HGNC:11937]	-2.034662
CD72	CD72 molecule [Source:HGNC Symbol;Acc:HGNC:1696]	1.0671585
CD81	CD81 molecule [Source:HGNC Symbol;Acc:HGNC:1701]	-1.11832445
CD82	CD82 molecule [Source:HGNC Symbol;Acc:HGNC:6210]	-1.274367
CD83	CD83 molecule [Source:HGNC Symbol;Acc:HGNC:1703]	-8.712782
CD8A	CD8a molecule [Source:HGNC Symbol;Acc:HGNC:1706]	-1.1076034
CD99	CD99 molecule [Source:HGNC Symbol;Acc:HGNC:7082]	-1.2449781
CD99L2	CD99 molecule like 2 [Source:HGNC Symbol;Acc:HGNC:18237]	-1.1827847
CDADC1	cytidine and dCMP deaminase domain containing 1 [Source:HGNC Symbol;Acc:HGNC:20299]	1.101491
CDC14A	cell division cycle 14A [Source:HGNC Symbol;Acc:HGNC:1718]	-1.2947338
CDC14C	cell division cycle 14C, pseudogene [Source:HGNC Symbol;Acc:HGNC:22427]	-1.0666852
CDC20B	cell division cycle 20B [Source:HGNC Symbol;Acc:HGNC:24222]	1.1306446
CDC27	cell division cycle 27 [Source:HGNC Symbol;Acc:HGNC:1728]	1.3274866
CDC37	cell division cycle 37 [Source:HGNC Symbol;Acc:HGNC:1735]	-1.1099256
CDC40	cell division cycle 40 [Source:HGNC Symbol;Acc:HGNC:17350]	1.429435

CDC42EP2	CDC42 effector protein 2 [Source:HGNC Symbol;Acc:HGNC:16263]	-1.526188
CDC42SE1	CDC42 small effector 1 [Source:HGNC Symbol;Acc:HGNC:17719]	-1.3183223
CDC45	cell division cycle 45 [Source:HGNC Symbol;Acc:HGNC:1739]	1.198193
CDC7	cell division cycle 7 [Source:HGNC Symbol;Acc:HGNC:1745]	1.404828
CDCA2	cell division cycle associated 2 [Source:HGNC Symbol;Acc:HGNC:14623]	1.392837
CDH11	cadherin 11 [Source:HGNC Symbol;Acc:HGNC:1750]	-1.3912827
CDH3	cadherin 3 [Source:HGNC Symbol;Acc:HGNC:1762]	-1.0770509
CDH5	cadherin 5 [Source:HGNC Symbol;Acc:HGNC:1764]	-1.2215142
CDH6	cadherin 6 [Source:HGNC Symbol;Acc:HGNC:1765]	1.0934659
CDHR1	cadherin related family member 1 [Source:HGNC Symbol;Acc:HGNC:14550]	-1.0701951
CDHR2	cadherin related family member 2 [Source:HGNC Symbol;Acc:HGNC:18231]	-1.186482
CDHR4	cadherin related family member 4 [Source:HGNC Symbol;Acc:HGNC:34527]	-1.0907234
CDK1	cyclin dependent kinase 1 [Source:HGNC Symbol;Acc:HGNC:1722]	1.5477147
CDK19	cyclin dependent kinase 19 [Source:HGNC Symbol;Acc:HGNC:19338]	1.3349775
CDK2	cyclin dependent kinase 2 [Source:HGNC Symbol;Acc:HGNC:1771]	1.0927821
CDK2AP2	cyclin dependent kinase 2 associated protein 2 [Source:HGNC Symbol;Acc:HGNC:30833]	-1.1945862
CDK5R1	cyclin dependent kinase 5 regulatory subunit 1 [Source:HGNC Symbol;Acc:HGNC:1775]	1.0657651
CDKL1	cyclin dependent kinase like 1 [Source:HGNC Symbol;Acc:HGNC:1781]	-1.1402655
CDKN2A	cyclin dependent kinase inhibitor 2A [Source:HGNC Symbol;Acc:HGNC:1787]	-1.1714987
CDKN3	cyclin dependent kinase inhibitor 3 [Source:HGNC Symbol;Acc:HGNC:1791]	1.2285981
CDNF	cerebral dopamine neurotrophic factor [Source:HGNC Symbol;Acc:HGNC:24913]	1.1389828
CDV3	CDV3 homolog [Source:HGNC Symbol;Acc:HGNC:26928]	-1.52176005
CDY2A	chromodomain Y-linked 2A [Source:HGNC Symbol;Acc:HGNC:1810]	1.068769
CEACAM20	carcinoembryonic antigen related cell adhesion molecule 20 [Source:HGNC Symbol;Acc:HGNC:24879]	-1.2031957
CEACAM4	carcinoembryonic antigen related cell adhesion molecule 4 [Source:HGNC Symbol;Acc:HGNC:1816]	-1.1137035
CEBPG	CCAAT/enhancer binding protein gamma [Source:HGNC Symbol;Acc:HGNC:1837]	1.2769895
CECR2	CECR2, histone acetyl-lysine reader [Source:HGNC Symbol;Acc:HGNC:1840]	-1.2035571
CECR3	ye syndrome chromosome region, candidate 3 (non-protein coding) [Source:HGNC Symbol;Acc:HGNC:1	-1.1294549
CECR6	cat eye syndrome chromosome region, candidate 6 [Source:HGNC Symbol;Acc:HGNC:1844]	1.289251
CELF3	CUGBP, Elav-like family member 3 [Source:HGNC Symbol;Acc:HGNC:11967]	1.382329
CELF6	CUGBP, Elav-like family member 6 [Source:HGNC Symbol;Acc:HGNC:14059]	-1.2530304
CELSR1	cadherin EGF LAG seven-pass G-type receptor 1 [Source:HGNC Symbol;Acc:HGNC:1850]	-1.1888963
CEMP1	cementum protein 1 [Source:HGNC Symbol;Acc:HGNC:32553]	1.1171341
CENPB	centromere protein B [Source:HGNC Symbol;Acc:HGNC:1852]	1.4983394
CENPN	centromere protein N [Source:HGNC Symbol;Acc:HGNC:30873]	1.1702483
CENPO	centromere protein O [Source:HGNC Symbol;Acc:HGNC:28152]	-1.1749657
CENPQ	centromere protein Q [Source:HGNC Symbol;Acc:HGNC:21347]	1.4293792
CENPT	centromere protein T [Source:HGNC Symbol;Acc:HGNC:25787]	-1.2762923
CEP44	centrosomal protein 44 [Source:HGNC Symbol;Acc:HGNC:29356]	1.274821
CEP55	centrosomal protein 55 [Source:HGNC Symbol;Acc:HGNC:1161]	1.5273156
CEP68	centrosomal protein 68 [Source:HGNC Symbol;Acc:HGNC:29076]	-1.0627037
CEP78	centrosomal protein 78 [Source:HGNC Symbol;Acc:HGNC:25740]	1.112971
CERS1	ceramide synthase 1 [Source:HGNC Symbol;Acc:HGNC:14253]	1.3349206
CERS4	ceramide synthase 4 [Source:HGNC Symbol;Acc:HGNC:23747]	-1.2284675
CERS6	ceramide synthase 6 [Source:HGNC Symbol;Acc:HGNC:23826]	1.5767936
CETN3	centrin 3 [Source:HGNC Symbol;Acc:HGNC:1868]	1.4286331
CFL1	cofilin 1 [Source:HGNC Symbol;Acc:HGNC:1874]	1.6365959
CFLAR	CASP8 and FADD like apoptosis regulator [Source:HGNC Symbol;Acc:HGNC:1876]	-2.2210145
CGGBP1	CGG triplet repeat binding protein 1 [Source:HGNC Symbol;Acc:HGNC:1888]	1.3796169
CGN	cingulin [Source:HGNC Symbol;Acc:HGNC:17429]	1.173265
CGRRF1	cell growth regulator with ring finger domain 1 [Source:HGNC Symbol;Acc:HGNC:15528]	-1.211295
CHAF1B	chromatin assembly factor 1 subunit B [Source:HGNC Symbol;Acc:HGNC:1911]	1.1342682
CHCHD2	coiled-coil-helix-coiled-coil-helix domain containing 2 [Source:HGNC Symbol;Acc:HGNC:21645]	-1.2115042
CHCHD7	coiled-coil-helix-coiled-coil-helix domain containing 7 [Source:HGNC Symbol;Acc:HGNC:28314]	1.3429021
CHD3	chromodomain helicase DNA binding protein 3 [Source:HGNC Symbol;Acc:HGNC:1918]	-1.2134196
CHD4	chromodomain helicase DNA binding protein 4 [Source:HGNC Symbol;Acc:HGNC:1919]	-1.492511
CHD5	chromodomain helicase DNA binding protein 5 [Source:HGNC Symbol;Acc:HGNC:16816]	-1.1523012
CHD7	chromodomain helicase DNA binding protein 7 [Source:HGNC Symbol;Acc:HGNC:20626]	-1.3585821
CHGB	chromogranin B [Source:HGNC Symbol;Acc:HGNC:1930]	1.078956
CHI3L2	chitinase 3 like 2 [Source:HGNC Symbol;Acc:HGNC:1933]	1.0916927
CHIC2	cysteine rich hydrophobic domain 2 [Source:HGNC Symbol;Acc:HGNC:1935]	-1.5332059
CHMP2B	charged multivesicular body protein 2B [Source:HGNC Symbol;Acc:HGNC:24537]	1.2864021
CHMP5	charged multivesicular body protein 5 [Source:HGNC Symbol;Acc:HGNC:26942]	1.2925906
CHN1	chimerin 1 [Source:HGNC Symbol;Acc:HGNC:1943]	-1.1034654
CHPF	chondroitin polymerizing factor [Source:HGNC Symbol;Acc:HGNC:24291]	-1.0709256
CHPF2	chondroitin polymerizing factor 2 [Source:HGNC Symbol;Acc:HGNC:29270]	1.1258259
CHRDL2	chordin like 2 [Source:HGNC Symbol;Acc:HGNC:24168]	1.1278981
CHRM5	cholinergic receptor muscarinic 5 [Source:HGNC Symbol;Acc:HGNC:1954]	-1.0505883
CHRNA1	cholinergic receptor nicotinic alpha 1 subunit [Source:HGNC Symbol;Acc:HGNC:1955]	-1.0992494
CHRNA4	cholinergic receptor nicotinic alpha 4 subunit [Source:HGNC Symbol;Acc:HGNC:1958]	1.0896581
CHRNB1	cholinergic receptor nicotinic beta 1 subunit [Source:HGNC Symbol;Acc:HGNC:1961]	-1.3048493
CHRNB4	cholinergic receptor nicotinic beta 4 subunit [Source:HGNC Symbol;Acc:HGNC:1964]	1.0629847
CHST11	carbohydrate sulfotransferase 11 [Source:HGNC Symbol;Acc:HGNC:17422]	-1.1847728
CHST4	carbohydrate sulfotransferase 4 [Source:HGNC Symbol;Acc:HGNC:1972]	-1.1798018
CHST5	carbohydrate sulfotransferase 5 [Source:HGNC Symbol;Acc:HGNC:1973]	-1.1764448
CHST9	carbohydrate sulfotransferase 9 [Source:HGNC Symbol;Acc:HGNC:19898]	-1.0786827
CHTOP	chromatin target of PRMT1 [Source:HGNC Symbol;Acc:HGNC:24511]	-1.0715858
CHUK	conserved helix-loop-helix ubiquitous kinase [Source:HGNC Symbol;Acc:HGNC:1974]	1.3930103
CIAPIN1	cytokine induced apoptosis inhibitor 1 [Source:HGNC Symbol;Acc:HGNC:28050]	-1.15125755
CIDEC	cell death inducing DFFA like effector c [Source:HGNC Symbol;Acc:HGNC:24229]	-1.2266004
CIDECP	cell death-inducing DFFA-like effector c pseudogene [Source:HGNC Symbol;Acc:HGNC:24230]	-1.4041673
CIITA	class II major histocompatibility complex transactivator [Source:HGNC Symbol;Acc:HGNC:7067]	-1.0939559
CIRBP	cold inducible RNA binding protein [Source:HGNC Symbol;Acc:HGNC:1982]	1.1738772
CITED2	) interacting transactivator with Glu/Asp rich carboxy-terminal domain 2 [Source:HGNC Symbol;Acc:HG	1.4461203
CITED4	interacting transactivator with Glu/Asp rich carboxy-terminal domain 4 [Source:HGNC Symbol;Acc:HGI	-6.7478375
CKLF	chemokine like factor [Source:HGNC Symbol;Acc:HGNC:13253]	-1.1130064

CKS1B	CDC28 protein kinase regulatory subunit 1B [Source:HGNC Symbol;Acc:HGNC:19083]	1.0569695
CLASRP	CLK4 associating serine/arginine rich protein [Source:HGNC Symbol;Acc:HGNC:17731]	1.1578135
CLCA1	chloride channel accessory 1 [Source:HGNC Symbol;Acc:HGNC:2015]	1.1619898
CLCN5	chloride voltage-gated channel 5 [Source:HGNC Symbol;Acc:HGNC:2023]	-1.2384921
CLCNKA	chloride voltage-gated channel Ka [Source:HGNC Symbol;Acc:HGNC:2026]	-1.15006
CLDN1	claudin 1 [Source:HGNC Symbol;Acc:HGNC:2032]	-1.153556
CLDN10	claudin 10 [Source:HGNC Symbol;Acc:HGNC:2033]	-1.097779
CLDN14	claudin 14 [Source:HGNC Symbol;Acc:HGNC:2035]	-2.4382842
CLDN2	claudin 2 [Source:HGNC Symbol;Acc:HGNC:2041]	-1.4316212
CLDN20	claudin 20 [Source:HGNC Symbol;Acc:HGNC:2042]	1.0906242
CLDN24	claudin 24 [Source:HGNC Symbol;Acc:HGNC:37200]	-1.1404729
CLDN3	claudin 3 [Source:HGNC Symbol;Acc:HGNC:2045]	1.1285096
CLDN5	claudin 5 [Source:HGNC Symbol;Acc:HGNC:2047]	-1.8893173
CLDN6	claudin 6 [Source:HGNC Symbol;Acc:HGNC:2048]	-1.1232823
CLEC14A	C-type lectin domain family 14 member A [Source:HGNC Symbol;Acc:HGNC:19832]	1.2823393
CLEC17A	C-type lectin domain family 17 member A [Source:HGNC Symbol;Acc:HGNC:34520]	-1.1239182
CLEC1A	C-type lectin domain family 1 member A [Source:HGNC Symbol;Acc:HGNC:24355]	-2.30176
CLEC2D	C-type lectin domain family 2 member D [Source:HGNC Symbol;Acc:HGNC:14351]	-1.2593296
CLEC4GP1	C-type lectin domain family 4 member G pseudogene 1 [Source:HGNC Symbol;Acc:HGNC:33502]	2.1335766
CLEC5A	C-type lectin domain family 5 member A [Source:HGNC Symbol;Acc:HGNC:2054]	1.1410142
CLEC7A	C-type lectin domain family 7 member A [Source:HGNC Symbol;Acc:HGNC:14558]	1.11641765
CLEC9A	C-type lectin domain family 9 member A [Source:HGNC Symbol;Acc:HGNC:26705]	1.1081402
CLIC4	chloride intracellular channel 4 [Source:HGNC Symbol;Acc:HGNC:13518]	-1.325485
CLIP2	CAP-Gly domain containing linker protein 2 [Source:HGNC Symbol;Acc:HGNC:2586]	-1.49145165
CLIP4	CAP-Gly domain containing linker protein family member 4 [Source:HGNC Symbol;Acc:HGNC:26108]	-1.0971203
CLK3	CDC like kinase 3 [Source:HGNC Symbol;Acc:HGNC:2071]	-1.0954059
CLLU1OS	chronic lymphocytic leukemia up-regulated 1 opposite strand [Source:HGNC Symbol;Acc:HGNC:24070]	-1.1460944
CLMN	calmin [Source:HGNC Symbol;Acc:HGNC:19972]	1.4177538
CLN8	ceroid-lipofuscinosis, neuronal 8 [Source:HGNC Symbol;Acc:HGNC:2079]	-1.2263199
CLNK	cytokine dependent hematopoietic cell linker [Source:HGNC Symbol;Acc:HGNC:17438]	1.0978724
CLP1	cleavage and polyadenylation factor I subunit 1 [Source:HGNC Symbol;Acc:HGNC:16999]	-1.156904
CLPP	caseinolytic mitochondrial matrix peptidase proteolytic subunit [Source:HGNC Symbol;Acc:HGNC:2084]	1.2030133
CLPTM1	CLPTM1, transmembrane protein [Source:HGNC Symbol;Acc:HGNC:2087]	1.3943906
CLRN1	clarin 1 [Source:HGNC Symbol;Acc:HGNC:12605]	1.1275384
CLRN2	clarin 2 [Source:HGNC Symbol;Acc:HGNC:33939]	1.1069058
CLSTN1	calsyntenin 1 [Source:HGNC Symbol;Acc:HGNC:17447]	-1.1261196
CLSTN3	calsyntenin 3 [Source:HGNC Symbol;Acc:HGNC:18371]	1.1784119
CLTA	clathrin light chain A [Source:HGNC Symbol;Acc:HGNC:2090]	-1.1609766
CLU	clusterin [Source:HGNC Symbol;Acc:HGNC:2095]	-1.1240908
CLUAP1	clusterin associated protein 1 [Source:HGNC Symbol;Acc:HGNC:19009]	1.3289251
CMBL	carboxymethylenebutenolidase homolog [Source:HGNC Symbol;Acc:HGNC:25090]	1.1177225
CMTM3	CKLF like MARVEL transmembrane domain containing 3 [Source:HGNC Symbol;Acc:HGNC:19174]	-1.1710814
CMTM7	CKLF like MARVEL transmembrane domain containing 7 [Source:HGNC Symbol;Acc:HGNC:19178]	1.1628163
CMTM8	CKLF like MARVEL transmembrane domain containing 8 [Source:HGNC Symbol;Acc:HGNC:19179]	1.4079307
CNDP2	CNDP dipeptidase 2 (metallopeptidase M20 family) [Source:HGNC Symbol;Acc:HGNC:24437]	-1.2163818
CNGA1	cyclic nucleotide gated channel alpha 1 [Source:HGNC Symbol;Acc:HGNC:2148]	1.1465813
CNIH4	cornichon family AMPA receptor auxiliary protein 4 [Source:HGNC Symbol;Acc:HGNC:25013]	-1.10078285
CNKSR2	connector enhancer of kinase suppressor of Ras 2 [Source:HGNC Symbol;Acc:HGNC:19701]	1.0963234
CNKSR3	CNKSR family member 3 [Source:HGNC Symbol;Acc:HGNC:23034]	-3.14431505
CNOT2	CCR4-NOT transcription complex subunit 2 [Source:HGNC Symbol;Acc:HGNC:7878]	1.2214175
CNOT4	CCR4-NOT transcription complex subunit 4 [Source:HGNC Symbol;Acc:HGNC:7880]	1.0868953
CNP	2',3'-cyclic nucleotide 3' phosphodiesterase [Source:HGNC Symbol;Acc:HGNC:2158]	1.0699669
CNPY2	canopy FGF signaling regulator 2 [Source:HGNC Symbol;Acc:HGNC:13529]	1.0882498
CNPY4	canopy FGF signaling regulator 4 [Source:HGNC Symbol;Acc:HGNC:28631]	-1.0825522
CNTN3	contactin 3 [Source:HGNC Symbol;Acc:HGNC:2173]	1.087419
CNTNAP1	contactin associated protein 1 [Source:HGNC Symbol;Acc:HGNC:8011]	-1.3719059
CNTNAP2	contactin associated protein-like 2 [Source:HGNC Symbol;Acc:HGNC:13830]	1.1986312
CNTNAP3	contactin associated protein-like 3 [Source:HGNC Symbol;Acc:HGNC:13834]	1.6613365
CNTNAP3B	contactin associated protein-like 3B [Source:HGNC Symbol;Acc:HGNC:32035]	1.1038735
CNTRL	centriolin [Source:HGNC Symbol;Acc:HGNC:1858]	1.15269
COBL	cordon-bleu WH2 repeat protein [Source:HGNC Symbol;Acc:HGNC:22199]	-1.0788543
COG1	component of oligomeric golgi complex 1 [Source:HGNC Symbol;Acc:HGNC:6545]	-1.1609445
COG4	component of oligomeric golgi complex 4 [Source:HGNC Symbol;Acc:HGNC:18620]	-1.2365643
COG8	component of oligomeric golgi complex 8 [Source:HGNC Symbol;Acc:HGNC:18623]	1.1469045
COL11A2	collagen type XI alpha 2 chain [Source:HGNC Symbol;Acc:HGNC:2187]	-1.1690038
COL19A1	collagen type XIX alpha 1 chain [Source:HGNC Symbol;Acc:HGNC:2196]	1.0917976
COL20A1	collagen type XX alpha 1 chain [Source:HGNC Symbol;Acc:HGNC:14670]	-1.1107092
COL27A1	collagen type XXVII alpha 1 chain [Source:HGNC Symbol;Acc:HGNC:22986]	-1.94497425
COL4A1	collagen type IV alpha 1 chain [Source:HGNC Symbol;Acc:HGNC:2202]	-1.3767632
COL5A1	collagen type V alpha 1 chain [Source:HGNC Symbol;Acc:HGNC:2209]	-1.14863495
COL5A3	collagen type V alpha 3 chain [Source:HGNC Symbol;Acc:HGNC:14864]	1.1443648
COL6A4P2	collagen type VI alpha 4 pseudogene 2 [Source:HGNC Symbol;Acc:HGNC:38501]	1.106017
COL7A1	collagen type VII alpha 1 chain [Source:HGNC Symbol;Acc:HGNC:2214]	-1.0596586
COL9A2	collagen type IX alpha 2 chain [Source:HGNC Symbol;Acc:HGNC:2218]	-1.4563527
COMMD8	COMM domain containing 8 [Source:HGNC Symbol;Acc:HGNC:26036]	1.6332517
COMT	catechol-O-methyltransferase [Source:HGNC Symbol;Acc:HGNC:2228]	-1.1229084
COPS4	COP9 signalosome subunit 4 [Source:HGNC Symbol;Acc:HGNC:16702]	1.3179946
COQ2	coenzyme Q2, polyprenyltransferase [Source:HGNC Symbol;Acc:HGNC:25223]	1.1170671
CORO2B	coronin 2B [Source:HGNC Symbol;Acc:HGNC:2256]	-1.1370021
COTL1	coactosin like F-actin binding protein 1 [Source:HGNC Symbol;Acc:HGNC:18304]	-1.225889
COX15	COX15, cytochrome c oxidase assembly homolog [Source:HGNC Symbol;Acc:HGNC:2263]	-1.0603908
COX17	COX17, cytochrome c oxidase copper chaperone [Source:HGNC Symbol;Acc:HGNC:2264]	-1.1280407
COX19	COX19, cytochrome c oxidase assembly factor [Source:HGNC Symbol;Acc:HGNC:28074]	-1.1987144
COX4I1	cytochrome c oxidase subunit 4I1 [Source:HGNC Symbol;Acc:HGNC:2265]	-1.2745554
COX6A2	cytochrome c oxidase subunit 6A2 [Source:HGNC Symbol;Acc:HGNC:2279]	-1.2210068
CP	ceruloplasmin [Source:HGNC Symbol;Acc:HGNC:2295]	1.097947

CPA2	carboxypeptidase A2 [Source:HGNC Symbol;Acc:HGNC:2297]	-1.090973
CPB1	carboxypeptidase B1 [Source:HGNC Symbol;Acc:HGNC:2299]	-1.1146188
CPNE2	copine 2 [Source:HGNC Symbol;Acc:HGNC:2315]	-1.0834568
CPNE3	copine 3 [Source:HGNC Symbol;Acc:HGNC:2316]	1.2675887
CPOX	coproporphyrinogen oxidase [Source:HGNC Symbol;Acc:HGNC:2321]	1.2864143
CPPED1	calcineurin like phosphoesterase domain containing 1 [Source:HGNC Symbol;Acc:HGNC:25632]	-1.2824229
CPSF2	cleavage and polyadenylation specific factor 2 [Source:HGNC Symbol;Acc:HGNC:2325]	1.2245158
CPSF3L	cleavage and polyadenylation specific factor 3-like [Source:HGNC Symbol;Acc:HGNC:26052]	-1.1432475
CPSF7	cleavage and polyadenylation specific factor 7 [Source:HGNC Symbol;Acc:HGNC:30098]	-1.0677586
CPT1B	carnitine palmitoyltransferase 1B [Source:HGNC Symbol;Acc:HGNC:2329]	1.1522236
CR1	complement component 3b/4b receptor 1 (Knops blood group) [Source:HGNC Symbol;Acc:HGNC:2334]	1.1302441
CRABP2	cellular retinoic acid binding protein 2 [Source:HGNC Symbol;Acc:HGNC:2339]	-1.1324071
CRB2	crumbs 2, cell polarity complex component [Source:HGNC Symbol;Acc:HGNC:18688]	1.1038187
CREB3	cAMP responsive element binding protein 3 [Source:HGNC Symbol;Acc:HGNC:2347]	-1.6444906
CREB5	cAMP responsive element binding protein 5 [Source:HGNC Symbol;Acc:HGNC:16844]	-1.6096094
CRELD1	cysteine rich with EGF like domains 1 [Source:HGNC Symbol;Acc:HGNC:14630]	-1.1253083
CRHR1	corticotropin releasing hormone receptor 1 [Source:HGNC Symbol;Acc:HGNC:2357]	-1.1627062
CRHR2	corticotropin releasing hormone receptor 2 [Source:HGNC Symbol;Acc:HGNC:2358]	-1.1677796
CRIP2	cysteine rich protein 2 [Source:HGNC Symbol;Acc:HGNC:2361]	1.1835985
CRMP1	collapsin response mediator protein 1 [Source:HGNC Symbol;Acc:HGNC:2365]	-1.1747061
CRNN	cornulin [Source:HGNC Symbol;Acc:HGNC:1230]	-1.1120282
CROCCP2	ciliary rootlet coiled-coil, rootletin pseudogene 2 [Source:HGNC Symbol;Acc:HGNC:28170]	1.2071775
CRP	C-reactive protein, pentraxin-related [Source:HGNC Symbol;Acc:HGNC:2367]	-1.1338637
CRTAC1	cartilage acidic protein 1 [Source:HGNC Symbol;Acc:HGNC:14882]	-1.3316295
CRX	cone-rod homeobox [Source:HGNC Symbol;Acc:HGNC:2383]	-1.1038014
CRYBA4	crystallin beta A4 [Source:HGNC Symbol;Acc:HGNC:2396]	-1.0678827
CRYGA	crystallin gamma A [Source:HGNC Symbol;Acc:HGNC:2408]	-1.1254486
CRYGD	crystallin gamma D [Source:HGNC Symbol;Acc:HGNC:2411]	-1.0746359
CSE1L	chromosome segregation 1 like [Source:HGNC Symbol;Acc:HGNC:2431]	1.36635325
CSF1	colony stimulating factor 1 [Source:HGNC Symbol;Acc:HGNC:2432]	-1.859645067
CSF2	colony stimulating factor 2 [Source:HGNC Symbol;Acc:HGNC:2434]	-6.7446213
CSF2RB	colony stimulating factor 2 receptor beta common subunit [Source:HGNC Symbol;Acc:HGNC:2436]	-1.2695988
CSF3	colony stimulating factor 3 [Source:HGNC Symbol;Acc:HGNC:2438]	1.0935498
CSGALNACT1	chondroitin sulfate N-acetylgalactosaminyltransferase 1 [Source:HGNC Symbol;Acc:HGNC:24290]	1.1393949
CSMD2	CUB and Sushi multiple domains 2 [Source:HGNC Symbol;Acc:HGNC:19290]	1.078597
CSNK1G1	casein kinase 1 gamma 1 [Source:HGNC Symbol;Acc:HGNC:2454]	-1.1858975
CSNK1G2	casein kinase 1 gamma 2 [Source:HGNC Symbol;Acc:HGNC:2455]	-1.3353855
CSNK2A2	casein kinase 2 alpha 2 [Source:HGNC Symbol;Acc:HGNC:2459]	-1.1260507
CSRNP1	cysteine and serine rich nuclear protein 1 [Source:HGNC Symbol;Acc:HGNC:14300]	-1.66625475
CSRP2	cysteine and glycine rich protein 2 [Source:HGNC Symbol;Acc:HGNC:2470]	-1.5023353
CSRP2BP	lysine acetyltransferase 14	1.4702252
CST3	cystatin C [Source:HGNC Symbol;Acc:HGNC:2475]	-1.2424277
CST6	cystatin E/M [Source:HGNC Symbol;Acc:HGNC:2478]	-1.0778985
CST8	cystatin 8 [Source:HGNC Symbol;Acc:HGNC:2480]	1.0998218
CT45A1	cancer/testis antigen family 45, member A1 [Source:HGNC Symbol;Acc:HGNC:33267]	1.1072483
CTC1	CTS telomere maintenance complex component 1 [Source:HGNC Symbol;Acc:HGNC:26169]	-1.363353
CTDNEP1	CTD nuclear envelope phosphatase 1 [Source:HGNC Symbol;Acc:HGNC:19085]	1.1867591
CTDSP1	CTD small phosphatase 1 [Source:HGNC Symbol;Acc:HGNC:21614]	1.4793745
CTDSP2	CTD small phosphatase 2 [Source:HGNC Symbol;Acc:HGNC:17077]	1.2208004
CTDSPL	CTD small phosphatase like [Source:HGNC Symbol;Acc:HGNC:16890]	1.2641559
CTH	cystathionine gamma-lyase [Source:HGNC Symbol;Acc:HGNC:2501]	1.2443477
CTHRC1	collagen triple helix repeat containing 1 [Source:HGNC Symbol;Acc:HGNC:18831]	-1.9386941
CTLA4	cytotoxic T-lymphocyte associated protein 4 [Source:HGNC Symbol;Acc:HGNC:2505]	1.1018863
CTNNA2	catenin alpha 2 [Source:HGNC Symbol;Acc:HGNC:2510]	1.0531777
CTNNAL1	catenin alpha like 1 [Source:HGNC Symbol;Acc:HGNC:2512]	1.6667547
CTNND1	catenin delta 1 [Source:HGNC Symbol;Acc:HGNC:2515]	1.0600032
CTNND2	catenin delta 2 [Source:HGNC Symbol;Acc:HGNC:2516]	-1.0800072
CTRL	chymotrypsin like [Source:HGNC Symbol;Acc:HGNC:2524]	-1.1494532
CTSB	cathepsin B [Source:HGNC Symbol;Acc:HGNC:2527]	-1.2449077
CTSC	cathepsin C [Source:HGNC Symbol;Acc:HGNC:2528]	1.3861682
CTSE	cathepsin E [Source:HGNC Symbol;Acc:HGNC:2530]	-1.0979679
CTSG	cathepsin G [Source:HGNC Symbol;Acc:HGNC:2532]	-1.1364323
CTSK	cathepsin K [Source:HGNC Symbol;Acc:HGNC:2536]	-1.837117
CTSW	cathepsin W [Source:HGNC Symbol;Acc:HGNC:2546]	1.1355982
CUL1	cullin 1 [Source:HGNC Symbol;Acc:HGNC:2551]	1.1490462
CUL3	cullin 3 [Source:HGNC Symbol;Acc:HGNC:2553]	1.1502621
CUL4B	cullin 4B [Source:HGNC Symbol;Acc:HGNC:2555]	1.5395381
CUTC	cutC copper transporter [Source:HGNC Symbol;Acc:HGNC:24271]	1.0982262
CWH43	cell wall biogenesis 43 C-terminal homolog [Source:HGNC Symbol;Acc:HGNC:26133]	1.0515262
CX3CL1	C-X3-C motif chemokine ligand 1 [Source:HGNC Symbol;Acc:HGNC:10647]	-7.8765554
CXADR	coxsackie virus and adenovirus receptor [Source:HGNC Symbol;Acc:HGNC:2559]	1.3749342
CXCL1	C-X-C motif chemokine ligand 1 [Source:HGNC Symbol;Acc:HGNC:4602]	-9.5333325
CXCL11	C-X-C motif chemokine ligand 11 [Source:HGNC Symbol;Acc:HGNC:10638]	-1.2888006
CXCL12	C-X-C motif chemokine ligand 12 [Source:HGNC Symbol;Acc:HGNC:10672]	-1.0890541
CXCL13	C-X-C motif chemokine ligand 13 [Source:HGNC Symbol;Acc:HGNC:10639]	1.0681758
CXCL14	C-X-C motif chemokine ligand 14 [Source:HGNC Symbol;Acc:HGNC:10640]	1.1251385
CXCL2	C-X-C motif chemokine ligand 2 [Source:HGNC Symbol;Acc:HGNC:4603]	-12.50969735
CXCL3	C-X-C motif chemokine ligand 3 [Source:HGNC Symbol;Acc:HGNC:4604]	-5.859939
CXCL6	C-X-C motif chemokine ligand 6 [Source:HGNC Symbol;Acc:HGNC:10643]	-1.4627546
CXCR1	C-X-C motif chemokine receptor 1 [Source:HGNC Symbol;Acc:HGNC:6026]	-1.1601862
CXCR3	C-X-C motif chemokine receptor 3 [Source:HGNC Symbol;Acc:HGNC:4540]	-1.0888819
CXCR4	C-X-C motif chemokine receptor 4 [Source:HGNC Symbol;Acc:HGNC:2561]	1.108087
CXCR6	C-X-C motif chemokine receptor 6 [Source:HGNC Symbol;Acc:HGNC:16647]	1.055194
CXCR7	atypical chemokine receptor 3	-14.334749
CXorf18	SPANXA2 overlapping transcript 1	1.095253
CXorf21	chromosome X open reading frame 21 [Source:HGNC Symbol;Acc:HGNC:25667]	1.1078659

CXorf36	chromosome X open reading frame 36 [Source:HGNC Symbol;Acc:HGNC:25866]	-1.1323607
CXorf38	chromosome X open reading frame 38 [Source:HGNC Symbol;Acc:HGNC:28589]	-1.2250423
CXorf51	chromosome X open reading frame 51A	1.0736921
CXorf64	proline rich 32	-1.1249257
CXXC5	CXXC finger protein 5 [Source:HGNC Symbol;Acc:HGNC:26943]	-1.5005684
CYB5A	cytochrome b5 type A [Source:HGNC Symbol;Acc:HGNC:2570]	-1.29712405
CYB5D1	cytochrome b5 domain containing 1 [Source:HGNC Symbol;Acc:HGNC:26516]	1.10247
CYB5R2	cytochrome b5 reductase 2 [Source:HGNC Symbol;Acc:HGNC:24376]	-1.6740136
CYB5R3	cytochrome b5 reductase 3 [Source:HGNC Symbol;Acc:HGNC:2873]	1.3956097
CYB5R4	cytochrome b5 reductase 4 [Source:HGNC Symbol;Acc:HGNC:20147]	1.1720557
CYB5RL	cytochrome b5 reductase like [Source:HGNC Symbol;Acc:HGNC:32220]	1.0829794
CYBRD1	cytochrome b reductase 1 [Source:HGNC Symbol;Acc:HGNC:20797]	1.2041178
CYC1	cytochrome c1 [Source:HGNC Symbol;Acc:HGNC:2579]	-1.2096454
CYFIP1	cytoplasmic FMR1 interacting protein 1 [Source:HGNC Symbol;Acc:HGNC:13759]	-1.1765275
CYLD	CYLD lysine 63 deubiquitinase [Source:HGNC Symbol;Acc:HGNC:2584]	-1.3167466
CYorf15B	taxilin gamma pseudogene, Y-linked	1.0742285
CYP11A1	cytochrome P450 family 11 subfamily A member 1 [Source:HGNC Symbol;Acc:HGNC:2590]	-1.3378371
CYP19A1	cytochrome P450 family 19 subfamily A member 1 [Source:HGNC Symbol;Acc:HGNC:2594]	1.1069825
CYP1A1	cytochrome P450 family 1 subfamily A member 1 [Source:HGNC Symbol;Acc:HGNC:2595]	1.3860935
CYP26B1	cytochrome P450 family 26 subfamily B member 1 [Source:HGNC Symbol;Acc:HGNC:20581]	1.0655626
CYP2B6	cytochrome P450 family 2 subfamily B member 6 [Source:HGNC Symbol;Acc:HGNC:2615]	-1.1604466
CYP2C18	cytochrome P450 family 2 subfamily C member 18 [Source:HGNC Symbol;Acc:HGNC:2620]	1.0651631
CYP2C19	cytochrome P450 family 2 subfamily C member 19 [Source:HGNC Symbol;Acc:HGNC:2621]	1.223847
CYP2R1	cytochrome P450 family 2 subfamily R member 1 [Source:HGNC Symbol;Acc:HGNC:20580]	1.2569096
CYP2U1	cytochrome P450 family 2 subfamily U member 1 [Source:HGNC Symbol;Acc:HGNC:20582]	1.2846699
CYP4A11	cytochrome P450 family 4 subfamily A member 11 [Source:HGNC Symbol;Acc:HGNC:2642]	-1.0868634
CYP4A22	cytochrome P450 family 4 subfamily A member 22 [Source:HGNC Symbol;Acc:HGNC:20575]	-1.1514848
CYP4V2	cytochrome P450 family 4 subfamily V member 2 [Source:HGNC Symbol;Acc:HGNC:23198]	1.1050348
CYSLTR1	cysteinyl leukotriene receptor 1 [Source:HGNC Symbol;Acc:HGNC:17451]	-1.1258904
CYTH1	cytohesin 1 [Source:HGNC Symbol;Acc:HGNC:9501]	-1.4833946
CYTH3	cytohesin 3 [Source:HGNC Symbol;Acc:HGNC:9504]	-1.1836414
CYTH4	cytohesin 4 [Source:HGNC Symbol;Acc:HGNC:9505]	-1.2447225
CYYR1	cysteine and tyrosine rich 1 [Source:HGNC Symbol;Acc:HGNC:16274]	1.1770687
D2HGDH	D-2-hydroxyglutarate dehydrogenase [Source:HGNC Symbol;Acc:HGNC:28358]	1.2745272
DAAM2	dishevelled associated activator of morphogenesis 2 [Source:HGNC Symbol;Acc:HGNC:18143]	-1.1322964
DAB2IP	DAB2 interacting protein [Source:HGNC Symbol;Acc:HGNC:17294]	1.1102239
DACH1	dachshund family transcription factor 1 [Source:HGNC Symbol;Acc:HGNC:2663]	1.5632034
DACT2	dishevelled binding antagonist of beta catenin 2 [Source:HGNC Symbol;Acc:HGNC:21231]	1.097339
DACT3	dishevelled binding antagonist of beta catenin 3 [Source:HGNC Symbol;Acc:HGNC:30745]	1.3489164
DAD1	defender against cell death 1 [Source:HGNC Symbol;Acc:HGNC:2664]	-1.0709051
DAG1	dystroglycan 1 [Source:HGNC Symbol;Acc:HGNC:2666]	1.08992
DAGLB	diacylglycerol lipase beta [Source:HGNC Symbol;Acc:HGNC:28923]	1.2434747
DAK	triokinase and FMN cyclase	-1.1756042
DAND5	DAN domain BMP antagonist family member 5 [Source:HGNC Symbol;Acc:HGNC:26780]	1.1164163
DAPK2	death associated protein kinase 2 [Source:HGNC Symbol;Acc:HGNC:2675]	-2.6219275
DAPK3	death associated protein kinase 3 [Source:HGNC Symbol;Acc:HGNC:2676]	-1.7319168
DAPL1	death associated protein like 1 [Source:HGNC Symbol;Acc:HGNC:21490]	1.1656348
DARC	atypical chemokine receptor 1 (Duffy blood group)	-1.2593654
DARS	aspartyl-tRNA synthetase [Source:HGNC Symbol;Acc:HGNC:2678]	1.16403
DAXX	death domain associated protein [Source:HGNC Symbol;Acc:HGNC:2681]	-1.4579426
DAZ2	deleted in azoospermia 2 [Source:HGNC Symbol;Acc:HGNC:15964]	1.060838
DAZAP1	DAZ associated protein 1 [Source:HGNC Symbol;Acc:HGNC:2683]	1.0811741
DBC1	cell cycle and apoptosis regulator 2	1.0608325
DBIL5P2	diazepam binding inhibitor-like 5 pseudogene 2 [Source:HGNC Symbol;Acc:HGNC:38518]	-1.190495
DBT	dihydrolipoamide branched chain transacylase E2 [Source:HGNC Symbol;Acc:HGNC:2698]	1.0566113
DCAF10	DDB1 and CUL4 associated factor 10 [Source:HGNC Symbol;Acc:HGNC:23686]	-1.1582558
DCAF12L1	DDB1 and CUL4 associated factor 12 like 1 [Source:HGNC Symbol;Acc:HGNC:29395]	1.348303
DCAF12L2	DDB1 and CUL4 associated factor 12 like 2 [Source:HGNC Symbol;Acc:HGNC:32950]	-1.1793085
DCAF17	DDB1 and CUL4 associated factor 17 [Source:HGNC Symbol;Acc:HGNC:25784]	1.4117918
DCAF5	DDB1 and CUL4 associated factor 5 [Source:HGNC Symbol;Acc:HGNC:20224]	-1.160475
DCAF6	DDB1 and CUL4 associated factor 6 [Source:HGNC Symbol;Acc:HGNC:30002]	-1.0571734
DCAF8	DDB1 and CUL4 associated factor 8 [Source:HGNC Symbol;Acc:HGNC:24891]	-1.3338059
DCAF8L1	DDB1 and CUL4 associated factor 8 like 1 [Source:HGNC Symbol;Acc:HGNC:31810]	-1.0860978
DCBLD1	discoidin, CUB and LCCL domain containing 1 [Source:HGNC Symbol;Acc:HGNC:21479]	1.4889072
DCBLD2	discoidin, CUB and LCCL domain containing 2 [Source:HGNC Symbol;Acc:HGNC:24627]	1.5104313
DCDC2B	doublecortin domain containing 2B [Source:HGNC Symbol;Acc:HGNC:32576]	1.0810536
DCDC5	doublecortin domain containing 5	1.0958408
DCHS1	dachsous cadherin-related 1 [Source:HGNC Symbol;Acc:HGNC:13681]	1.1983539
DCLRE1A	DNA cross-link repair 1A [Source:HGNC Symbol;Acc:HGNC:17660]	1.4012119
DCN	decorin [Source:HGNC Symbol;Acc:HGNC:2705]	1.0989908
DCP1B	decapping mRNA 1B [Source:HGNC Symbol;Acc:HGNC:24451]	1.1064767
DCST2	DC-STAMP domain containing 2 [Source:HGNC Symbol;Acc:HGNC:26562]	-1.1777972
DCTN5	dynactin subunit 5 [Source:HGNC Symbol;Acc:HGNC:24594]	-1.2802501
DCUN1D3	defective in cullin neddylation 1 domain containing 3 [Source:HGNC Symbol;Acc:HGNC:28734]	-1.4613856
DDB1	damage specific DNA binding protein 1 [Source:HGNC Symbol;Acc:HGNC:2717]	-1.1855358
DDB2	damage specific DNA binding protein 2 [Source:HGNC Symbol;Acc:HGNC:2718]	1.1020458
DDC	dopa decarboxylase [Source:HGNC Symbol;Acc:HGNC:2719]	1.1106675
DDIT3	DNA damage inducible transcript 3 [Source:HGNC Symbol;Acc:HGNC:2726]	-1.2038928
DDN	dendrin [Source:HGNC Symbol;Acc:HGNC:24458]	-1.0941209
DDRGK1	DDRGK domain containing 1 [Source:HGNC Symbol;Acc:HGNC:16110]	-1.2230808
DDTL	D-dopachrome tautomerase-like [Source:HGNC Symbol;Acc:HGNC:33446]	-1.1296269
DDX1	DEAD/H-box helicase 1 [Source:HGNC Symbol;Acc:HGNC:2734]	1.2417631
DDX10	DEAD-box helicase 10 [Source:HGNC Symbol;Acc:HGNC:2735]	1.4677984
DDX11	DEAD/H-box helicase 11 [Source:HGNC Symbol;Acc:HGNC:2736]	1.2632715
DDX19B	DEAD-box helicase 19B [Source:HGNC Symbol;Acc:HGNC:2742]	-1.136775
DDX27	DEAD-box helicase 27 [Source:HGNC Symbol;Acc:HGNC:15837]	-1.1201895

DDX31	DEAD-box helicase 31 [Source:HGNC Symbol;Acc:HGNC:16715]	-1.1445807
DDX39A	DEAD-box helicase 39A [Source:HGNC Symbol;Acc:HGNC:17821]	-1.1351616
DDX50	DEAD-box helicase 50 [Source:HGNC Symbol;Acc:HGNC:17906]	1.1113956
DDX55	DEAD-box helicase 55 [Source:HGNC Symbol;Acc:HGNC:20085]	1.2344874
DDX58	DEXD/H-box helicase 58 [Source:HGNC Symbol;Acc:HGNC:19102]	-2.03503335
DDX6	DEAD-box helicase 6 [Source:HGNC Symbol;Acc:HGNC:2747]	1.494621
DEDD	death effector domain containing [Source:HGNC Symbol;Acc:HGNC:2755]	-1.4066712
DEF6	DEF6, guanine nucleotide exchange factor [Source:HGNC Symbol;Acc:HGNC:2760]	-1.1234981
DEFA7P	defensin alpha 7, pseudogene [Source:HGNC Symbol;Acc:HGNC:31798]	-1.1195017
DEFB1	defensin beta 1 [Source:HGNC Symbol;Acc:HGNC:2766]	-1.1559272
DEFB103A	defensin beta 103A [Source:HGNC Symbol;Acc:HGNC:15967]	1.1889591
DEFB109P1	defensin beta 109 pseudogene 1 [Source:HGNC Symbol;Acc:HGNC:18090]	-1.0843436
DEFB109P1B	defensin beta 109 pseudogene 1B [Source:HGNC Symbol;Acc:HGNC:33469]	1.1380223
DEFB110	defensin beta 110 [Source:HGNC Symbol;Acc:HGNC:18091]	-1.0916909
DEFB114	defensin beta 114 [Source:HGNC Symbol;Acc:HGNC:18095]	1.134733
DEFB119	defensin beta 119 [Source:HGNC Symbol;Acc:HGNC:18099]	1.1038206
DEFB121	defensin beta 121 [Source:HGNC Symbol;Acc:HGNC:18101]	-1.0653747
DEFB125	defensin beta 125 [Source:HGNC Symbol;Acc:HGNC:18105]	1.0649292
DEFB126	defensin beta 126 [Source:HGNC Symbol;Acc:HGNC:15900]	-1.0762001
DEFB127	defensin beta 127 [Source:HGNC Symbol;Acc:HGNC:16206]	-1.0581785
DEFB129	defensin beta 129 [Source:HGNC Symbol;Acc:HGNC:16218]	-1.1291511
DEFB131	defensin beta 131 [Source:HGNC Symbol;Acc:HGNC:18108]	1.0855083
DENND2C	DENN domain containing 2C [Source:HGNC Symbol;Acc:HGNC:24748]	1.1418628
DENND2D	DENN domain containing 2D [Source:HGNC Symbol;Acc:HGNC:26192]	-1.9576846
DENND3	DENN domain containing 3 [Source:HGNC Symbol;Acc:HGNC:29134]	-1.368438333
DEPDC1	DEP domain containing 1 [Source:HGNC Symbol;Acc:HGNC:22949]	1.7942548
DEPTOR	DEP domain containing MTOR-interacting protein [Source:HGNC Symbol;Acc:HGNC:22953]	-1.0528218
DEXI	Dexi homolog [Source:HGNC Symbol;Acc:HGNC:13267]	-1.1713661
DFNB59	deafness, autosomal recessive 59 [Source:HGNC Symbol;Acc:HGNC:29502]	1.0894096
DGKA	diacylglycerol kinase alpha [Source:HGNC Symbol;Acc:HGNC:2849]	1.247584
DGKD	diacylglycerol kinase delta [Source:HGNC Symbol;Acc:HGNC:2851]	-1.6001997
DGKE	diacylglycerol kinase epsilon [Source:HGNC Symbol;Acc:HGNC:2852]	1.1149938
DGUOK	deoxyguanosine kinase [Source:HGNC Symbol;Acc:HGNC:2858]	-1.0898392
DHCR24	24-dehydrocholesterol reductase [Source:HGNC Symbol;Acc:HGNC:2859]	1.2931651
DHCR7	7-dehydrocholesterol reductase [Source:HGNC Symbol;Acc:HGNC:2860]	-1.2607217
DHDDS	dehydrodolichyl diphosphate synthase subunit [Source:HGNC Symbol;Acc:HGNC:20603]	-1.1430659
DHFR	dihydrofolate reductase [Source:HGNC Symbol;Acc:HGNC:2861]	-1.0714201
DHRS3	dehydrogenase/reductase 3 [Source:HGNC Symbol;Acc:HGNC:17693]	-1.5317779
DHRS4	dehydrogenase/reductase 4 [Source:HGNC Symbol;Acc:HGNC:16985]	-1.0874914
DHRS7	dehydrogenase/reductase 7 [Source:HGNC Symbol;Acc:HGNC:21524]	-1.1704454
DHRSX	dehydrogenase/reductase X-linked [Source:HGNC Symbol;Acc:HGNC:18399]	-1.1266266
DHTKD1	dehydrogenase E1 and transketolase domain containing 1 [Source:HGNC Symbol;Acc:HGNC:23537]	-1.4028864
DHX29	DEAH-box helicase 29 [Source:HGNC Symbol;Acc:HGNC:15815]	1.4496934
DHX32	DEAH-box helicase 32 (putative) [Source:HGNC Symbol;Acc:HGNC:16717]	1.1722004
DHX57	DEAH-box helicase 57 [Source:HGNC Symbol;Acc:HGNC:20086]	1.1725931
DHX58	DEXH-box helicase 58 [Source:HGNC Symbol;Acc:HGNC:29517]	-1.6872457
DIABLO	diablo IAP-binding mitochondrial protein [Source:HGNC Symbol;Acc:HGNC:21528]	-1.0802038
DIDO1	death inducer-obliterator 1 [Source:HGNC Symbol;Acc:HGNC:2680]	-1.250554667
DIP2A	disco interacting protein 2 homolog A [Source:HGNC Symbol;Acc:HGNC:17217]	-1.1740835
DIRAS2	DIRAS family GTPase 2 [Source:HGNC Symbol;Acc:HGNC:19323]	1.0895224
DIRC2	disrupted in renal carcinoma 2 [Source:HGNC Symbol;Acc:HGNC:16628]	-1.14381065
DIS3	3 homolog, exosome endoribonuclease and 3'-5' exoribonuclease [Source:HGNC Symbol;Acc:HGNC:20(	1.176632
DIS3L	DIS3 like exosome 3'-5' exoribonuclease [Source:HGNC Symbol;Acc:HGNC:28698]	1.3491099
DISP1	dispatched RND transporter family member 1 [Source:HGNC Symbol;Acc:HGNC:19711]	1.0960468
DIXDC1	DIX domain containing 1 [Source:HGNC Symbol;Acc:HGNC:23695]	1.1372764
DKFZP434K028	uncharacterized LOC26070	-1.077371
DKFZP434L187	uncharacterized LOC26082	1.1501323
DKFZP686I15217	long intergenic non-protein coding RNA 1011	-1.1108372
DKFZp686O1327	testis expressed 41 (non-protein coding)	-1.3920181
DKK2	dickkopf WNT signaling pathway inhibitor 2 [Source:HGNC Symbol;Acc:HGNC:2892]	1.0658523
DKK3	dickkopf WNT signaling pathway inhibitor 3 [Source:HGNC Symbol;Acc:HGNC:2893]	-1.2618476
DLAT	dihydrolipoamide S-acetyltransferase [Source:HGNC Symbol;Acc:HGNC:2896]	1.3758544
DLEU1	deleted in lymphocytic leukemia 1 [Source:HGNC Symbol;Acc:HGNC:13747]	1.1873877
DLEU2L	deleted in lymphocytic leukemia 2-like [Source:HGNC Symbol;Acc:HGNC:13225]	1.0803314
DLGAP3	DLG associated protein 3 [Source:HGNC Symbol;Acc:HGNC:30368]	-1.1644888
DLGAP4	DLG associated protein 4 [Source:HGNC Symbol;Acc:HGNC:24476]	-1.1471112
DLGAP5	DLG associated protein 5 [Source:HGNC Symbol;Acc:HGNC:16864]	1.671802
DLK2	delta like non-canonical Notch ligand 2 [Source:HGNC Symbol;Acc:HGNC:21113]	-1.2348998
DLL1	delta like canonical Notch ligand 1 [Source:HGNC Symbol;Acc:HGNC:2908]	1.0888776
DLX2	distal-less homeobox 2 [Source:HGNC Symbol;Acc:HGNC:2915]	1.0856155
DLX5	distal-less homeobox 5 [Source:HGNC Symbol;Acc:HGNC:2918]	1.0703852
DMD	dystrophin [Source:HGNC Symbol;Acc:HGNC:2928]	1.1004931
DMRTA1	DMRT like family A1 [Source:HGNC Symbol;Acc:HGNC:13826]	1.1460854
DMXL2	Dmx like 2 [Source:HGNC Symbol;Acc:HGNC:2938]	1.1173317
DNAAF1	dynein axonemal assembly factor 1 [Source:HGNC Symbol;Acc:HGNC:30539]	1.1140326
DNAH10	dynein axonemal heavy chain 10 [Source:HGNC Symbol;Acc:HGNC:2941]	1.1869098
DNAH10OS	dynein axonemal heavy chain 10 opposite strand [Source:HGNC Symbol;Acc:HGNC:37121]	-1.1655846
DNAH17	dynein axonemal heavy chain 17 [Source:HGNC Symbol;Acc:HGNC:2946]	1.12843
DNAH2	dynein axonemal heavy chain 2 [Source:HGNC Symbol;Acc:HGNC:2948]	-1.1530064
DNAI1	dynein axonemal intermediate chain 1 [Source:HGNC Symbol;Acc:HGNC:2954]	-1.1280396
DNAJA1	DnaJ heat shock protein family (Hsp40) member A1 [Source:HGNC Symbol;Acc:HGNC:5229]	-1.666193
DNAJA1P5	naJ heat shock protein family (Hsp40) member A1 pseudogene 5 [Source:HGNC Symbol;Acc:HGNC:3934	-1.5662454
DNAJB4	DnaJ heat shock protein family (Hsp40) member B4 [Source:HGNC Symbol;Acc:HGNC:14886]	2.4465613
DNAJB5	DnaJ heat shock protein family (Hsp40) member B5 [Source:HGNC Symbol;Acc:HGNC:14887]	-1.28777755
DNAJB7	DnaJ heat shock protein family (Hsp40) member B7 [Source:HGNC Symbol;Acc:HGNC:24986]	1.1513389
DNAJC2	DnaJ heat shock protein family (Hsp40) member C2 [Source:HGNC Symbol;Acc:HGNC:13192]	1.4854336

DNAJC5	DnaJ heat shock protein family (Hsp40) member C5 [Source:HGNC Symbol;Acc:HGNC:16235]	-1.2373856
DNAJC5G	DnaJ heat shock protein family (Hsp40) member C5 gamma [Source:HGNC Symbol;Acc:HGNC:24844]	-1.0776639
DNAJC7	DnaJ heat shock protein family (Hsp40) member C7 [Source:HGNC Symbol;Acc:HGNC:12392]	1.2758703
DND1	DND microRNA-mediated repression inhibitor 1 [Source:HGNC Symbol;Acc:HGNC:23799]	1.1042858
DNER	delta/notch like EGF repeat containing [Source:HGNC Symbol;Acc:HGNC:24456]	1.0853204
DNHD1	dynein heavy chain domain 1 [Source:HGNC Symbol;Acc:HGNC:26532]	1.0947062
DNM2	dynamin 2 [Source:HGNC Symbol;Acc:HGNC:2974]	1.2012839
DNM3	dynamin 3 [Source:HGNC Symbol;Acc:HGNC:29125]	1.0869591
DNMT3A	DNA methyltransferase 3 alpha [Source:HGNC Symbol;Acc:HGNC:2978]	-1.3591165
DNMT3B	DNA methyltransferase 3 beta [Source:HGNC Symbol;Acc:HGNC:2979]	1.1827402
DNMT3L	DNA methyltransferase 3 like [Source:HGNC Symbol;Acc:HGNC:2980]	-1.0724828
DNPEP	aspartyl aminopeptidase [Source:HGNC Symbol;Acc:HGNC:2981]	-1.320053
DOCK6	dedicator of cytokinesis 6 [Source:HGNC Symbol;Acc:HGNC:19189]	1.1956887
DOCK7	dedicator of cytokinesis 7 [Source:HGNC Symbol;Acc:HGNC:19190]	1.5407163
DOCK9	dedicator of cytokinesis 9 [Source:HGNC Symbol;Acc:HGNC:14132]	-1.223552
DOK4	docking protein 4 [Source:HGNC Symbol;Acc:HGNC:19868]	1.3267583
DOK5	docking protein 5 [Source:HGNC Symbol;Acc:HGNC:16173]	-1.7620904
DOK7	docking protein 7 [Source:HGNC Symbol;Acc:HGNC:26594]	1.0998422
DONSON	downstream neighbor of SON [Source:HGNC Symbol;Acc:HGNC:2993]	1.1744759
DOT1L	DOT1 like histone lysine methyltransferase [Source:HGNC Symbol;Acc:HGNC:24948]	-1.2630701
DPCD	deleted in primary ciliary dyskinesia homolog (mouse) [Source:HGNC Symbol;Acc:HGNC:24542]	-1.1380336
DPF2	double PHD fingers 2 [Source:HGNC Symbol;Acc:HGNC:9964]	1.1379308
DPF3	double PHD fingers 3 [Source:HGNC Symbol;Acc:HGNC:17427]	-1.517012925
DPH3	diphthamide biosynthesis 3 [Source:HGNC Symbol;Acc:HGNC:27717]	-1.1696813
DPH3P1	diphthamide biosynthesis 3 pseudogene 1 [Source:HGNC Symbol;Acc:HGNC:16136]	-1.1200739
DPH5	diphthamide biosynthesis 5 [Source:HGNC Symbol;Acc:HGNC:24270]	-1.206536
DPP9	dipeptidyl peptidase 9 [Source:HGNC Symbol;Acc:HGNC:18648]	-1.1943061
DPT	dermatopontin [Source:HGNC Symbol;Acc:HGNC:3011]	-1.217323
DPY19L1	dpy-19 like 1 [Source:HGNC Symbol;Acc:HGNC:22205]	1.2373129
DPY19L2P2	DPY19L2 pseudogene 2 [Source:HGNC Symbol;Acc:HGNC:21764]	1.1666224
DQX1	DEAQ-box RNA dependent ATPase 1 [Source:HGNC Symbol;Acc:HGNC:20410]	-1.133204
DR1	down-regulator of transcription 1 [Source:HGNC Symbol;Acc:HGNC:3017]	-1.3932658
DRAM1	DNA damage regulated autophagy modulator 1 [Source:HGNC Symbol;Acc:HGNC:25645]	-1.6728582
DRD4	dopamine receptor D4 [Source:HGNC Symbol;Acc:HGNC:3025]	1.1841228
DRD5	dopamine receptor D5 [Source:HGNC Symbol;Acc:HGNC:3026]	-1.1729592
DSCAM	DS cell adhesion molecule [Source:HGNC Symbol;Acc:HGNC:3039]	-1.1684315
DSCR10	Down syndrome critical region 10 (non-protein coding) [Source:HGNC Symbol;Acc:HGNC:16302]	-1.2796013
DSCR3	DSCR3 arrestin fold containing [Source:HGNC Symbol;Acc:HGNC:3044]	-1.110164
DSCR8	Down syndrome critical region 8 [Source:HGNC Symbol;Acc:HGNC:16707]	1.097571
DSE	dermatan sulfate epimerase [Source:HGNC Symbol;Acc:HGNC:21144]	-1.4986777
DSEL	dermatan sulfate epimerase-like [Source:HGNC Symbol;Acc:HGNC:18144]	-1.2486271
DSPP	dentin sialophosphoprotein [Source:HGNC Symbol;Acc:HGNC:3054]	1.07887185
DSTYK	dual serine/threonine and tyrosine protein kinase [Source:HGNC Symbol;Acc:HGNC:29043]	-1.1395392
DTL	denticleless E3 ubiquitin protein ligase homolog [Source:HGNC Symbol;Acc:HGNC:30288]	1.5213585
DTNA	dystrobrevin alpha [Source:HGNC Symbol;Acc:HGNC:3057]	1.0739483
DTX2P1-UPK3BP1-PMS2P11	X2P1-UPK3BP1-PMS2P11 readthrough, transcribed pseudogene [Source:HGNC Symbol;Acc:HGNC:423	-1.1842337
DTX3	deltex E3 ubiquitin ligase 3 [Source:HGNC Symbol;Acc:HGNC:24457]	1.1237551
DTX3L	deltex E3 ubiquitin ligase 3L [Source:HGNC Symbol;Acc:HGNC:30323]	-1.88932
DTYMK	deoxythymidylate kinase [Source:HGNC Symbol;Acc:HGNC:3061]	-1.0985533
DUS1L	dihydrouridine synthase 1 like [Source:HGNC Symbol;Acc:HGNC:30086]	-1.1729836
DUSP11	dual specificity phosphatase 11 [Source:HGNC Symbol;Acc:HGNC:3066]	-1.1125304
DUSP14	dual specificity phosphatase 14 [Source:HGNC Symbol;Acc:HGNC:17007]	1.1705328
DUSP16	dual specificity phosphatase 16 [Source:HGNC Symbol;Acc:HGNC:17909]	-1.1610054
DUSP22	dual specificity phosphatase 22 [Source:HGNC Symbol;Acc:HGNC:16077]	-1.1626757
DUSP23	dual specificity phosphatase 23 [Source:HGNC Symbol;Acc:HGNC:21480]	1.3084495
DUSP27	dual specificity phosphatase 27 (putative) [Source:HGNC Symbol;Acc:HGNC:25034]	-1.0605184
DUSP3	dual specificity phosphatase 3 [Source:HGNC Symbol;Acc:HGNC:3069]	-1.4581567
DUSP4	dual specificity phosphatase 4 [Source:HGNC Symbol;Acc:HGNC:3070]	1.9665419
DUSP5	dual specificity phosphatase 5 [Source:HGNC Symbol;Acc:HGNC:3071]	-1.6911609
DUSP8	dual specificity phosphatase 8 [Source:HGNC Symbol;Acc:HGNC:3074]	-1.2854617
DUXA	double homeobox A [Source:HGNC Symbol;Acc:HGNC:32179]	-1.1215689
DVL1	dishevelled segment polarity protein 1 [Source:HGNC Symbol;Acc:HGNC:3084]	1.2205336
DVL2	dishevelled segment polarity protein 2 [Source:HGNC Symbol;Acc:HGNC:3086]	1.464206
DYNC1LI1	dynein cytoplasmic 1 light intermediate chain 1 [Source:HGNC Symbol;Acc:HGNC:18745]	1.2483132
DYNC2LI1	dynein cytoplasmic 2 light intermediate chain 1 [Source:HGNC Symbol;Acc:HGNC:24595]	-1.1046063
DYNLRB1	dynein light chain roadblock-type 1 [Source:HGNC Symbol;Acc:HGNC:15468]	-1.1551585
DYNLT1	dynein light chain Tctex-type 1 [Source:HGNC Symbol;Acc:HGNC:11697]	-1.1994211
DYRK3	dual specificity tyrosine phosphorylation regulated kinase 3 [Source:HGNC Symbol;Acc:HGNC:3094]	-1.2630743
E2F4	E2F transcription factor 4 [Source:HGNC Symbol;Acc:HGNC:3118]	1.2095329
E2F8	E2F transcription factor 8 [Source:HGNC Symbol;Acc:HGNC:24727]	1.6150968
EAPP	E2F associated phosphoprotein [Source:HGNC Symbol;Acc:HGNC:19312]	1.1342822
EARS2	glutamyl-tRNA synthetase 2, mitochondrial [Source:HGNC Symbol;Acc:HGNC:29419]	1.2250125
EBF2	early B-cell factor 2 [Source:HGNC Symbol;Acc:HGNC:19090]	-1.1474425
EBF3	early B-cell factor 3 [Source:HGNC Symbol;Acc:HGNC:19087]	1.26155255
EBF4	early B-cell factor 4 [Source:HGNC Symbol;Acc:HGNC:29278]	-1.1566467
EBI3	Epstein-Barr virus induced 3 [Source:HGNC Symbol;Acc:HGNC:3129]	-7.3279433
EBP	emopamil binding protein (sterol isomerase) [Source:HGNC Symbol;Acc:HGNC:3133]	1.174145
EBPL	emopamil binding protein like [Source:HGNC Symbol;Acc:HGNC:18061]	1.286869
ECE1	endothelin converting enzyme 1 [Source:HGNC Symbol;Acc:HGNC:3146]	-1.1795846
ECE2	endothelin converting enzyme 2 [Source:HGNC Symbol;Acc:HGNC:13275]	-1.1198297
ECHDC1	ethylmalonyl-CoA decarboxylase 1 [Source:HGNC Symbol;Acc:HGNC:21489]	1.1304486
ECHS1	enoyl-CoA hydratase, short chain 1 [Source:HGNC Symbol;Acc:HGNC:3151]	-1.2449684
EDA	ectodysplasin A [Source:HGNC Symbol;Acc:HGNC:3157]	1.1657732
EDAR	ectodysplasin A receptor [Source:HGNC Symbol;Acc:HGNC:2895]	1.0503049
EDC3	enhancer of mRNA decapping 3 [Source:HGNC Symbol;Acc:HGNC:26114]	-1.3001764
EDC4	enhancer of mRNA decapping 4 [Source:HGNC Symbol;Acc:HGNC:17157]	-1.2234017

EDEM3	ER degradation enhancing alpha-mannosidase like protein 3 [Source:HGNC Symbol;Acc:HGNC:16787]	1.1182569
EDN3	endothelin 3 [Source:HGNC Symbol;Acc:HGNC:3178]	-1.1606373
EDNRA	endothelin receptor type A [Source:HGNC Symbol;Acc:HGNC:3179]	-1.147842
EFCAB2	EF-hand calcium binding domain 2 [Source:HGNC Symbol;Acc:HGNC:28166]	1.1428593
EFCAB4B	calcium release activated channel regulator 2A	-1.132601
EFCAB5	EF-hand calcium binding domain 5 [Source:HGNC Symbol;Acc:HGNC:24801]	1.0997818
EFCAB9	EF-hand calcium binding domain 9 [Source:HGNC Symbol;Acc:HGNC:34530]	-1.4674324
EFHA1	mitochondrial calcium uptake 2	1.4516697
EFHD2	EF-hand domain family member D2 [Source:HGNC Symbol;Acc:HGNC:28670]	-1.121864
EFNA1	ephrin A1 [Source:HGNC Symbol;Acc:HGNC:3221]	-4.0122566
EFNB1	ephrin B1 [Source:HGNC Symbol;Acc:HGNC:3226]	-2.367385
EFNB3	ephrin B3 [Source:HGNC Symbol;Acc:HGNC:3228]	-1.1695634
EFR3A	EFR3 homolog A [Source:HGNC Symbol;Acc:HGNC:28970]	1.5596405
EGFL7	EGF like domain multiple 7 [Source:HGNC Symbol;Acc:HGNC:20594]	1.8818054
EGFR	epidermal growth factor receptor [Source:HGNC Symbol;Acc:HGNC:3236]	-1.299817
EGLN1	egl-9 family hypoxia inducible factor 1 [Source:HGNC Symbol;Acc:HGNC:1232]	-1.338289
EGLN2	egl-9 family hypoxia inducible factor 2 [Source:HGNC Symbol;Acc:HGNC:14660]	-1.2420547
EGR1	early growth response 1 [Source:HGNC Symbol;Acc:HGNC:3238]	-1.3900765
EGR3	early growth response 3 [Source:HGNC Symbol;Acc:HGNC:3240]	-1.0901271
EHBP1	EH domain binding protein 1 [Source:HGNC Symbol;Acc:HGNC:29144]	1.1500909
EHBP1L1	EH domain binding protein 1 like 1 [Source:HGNC Symbol;Acc:HGNC:30682]	-1.1504953
EHD1	EH domain containing 1 [Source:HGNC Symbol;Acc:HGNC:3242]	-2.0393786
EHD2	EH domain containing 2 [Source:HGNC Symbol;Acc:HGNC:3243]	1.1929669
EHD4	EH domain containing 4 [Source:HGNC Symbol;Acc:HGNC:3245]	-1.1534959
EHMT1	euchromatic histone lysine methyltransferase 1 [Source:HGNC Symbol;Acc:HGNC:24650]	-1.1291439
EHMT2	euchromatic histone lysine methyltransferase 2 [Source:HGNC Symbol;Acc:HGNC:14129]	1.3164546
EIF1AX	eukaryotic translation initiation factor 1A, X-linked [Source:HGNC Symbol;Acc:HGNC:3250]	1.3421187
EIF1B	eukaryotic translation initiation factor 1B [Source:HGNC Symbol;Acc:HGNC:30792]	-1.1926552
EIF2AK1	eukaryotic translation initiation factor 2 alpha kinase 1 [Source:HGNC Symbol;Acc:HGNC:24921]	-1.207122
EIF2C1	argonaute 1, RISC catalytic component	-1.1766923
EIF2C2	argonaute 2, RISC catalytic component	-1.4192165
EIF2C3	argonaute 3, RISC catalytic component	-1.2021523
EIF2S1	eukaryotic translation initiation factor 2 subunit alpha [Source:HGNC Symbol;Acc:HGNC:3265]	1.2025362
EIF3D	eukaryotic translation initiation factor 3 subunit D [Source:HGNC Symbol;Acc:HGNC:3278]	-1.2524226
EIF3E	eukaryotic translation initiation factor 3 subunit E [Source:HGNC Symbol;Acc:HGNC:3277]	1.657031
EIF3F	eukaryotic translation initiation factor 3 subunit F [Source:HGNC Symbol;Acc:HGNC:3275]	-1.065591
EIF4A1	eukaryotic translation initiation factor 4A1 [Source:HGNC Symbol;Acc:HGNC:3282]	1.1480283
EIF4A3	eukaryotic translation initiation factor 4A3 [Source:HGNC Symbol;Acc:HGNC:18683]	1.183338
EIF4E2	eukaryotic translation initiation factor 4E family member 2 [Source:HGNC Symbol;Acc:HGNC:3293]	-1.1510102
EIF4EBP1	eukaryotic translation initiation factor 4E binding protein 1 [Source:HGNC Symbol;Acc:HGNC:3288]	1.1354073
EIF4EBP2	eukaryotic translation initiation factor 4E binding protein 2 [Source:HGNC Symbol;Acc:HGNC:3289]	1.1208414
EIF4ENIF1	ukaryotic translation initiation factor 4E nuclear import factor 1 [Source:HGNC Symbol;Acc:HGNC:1668	-1.1831337
EIF4H	eukaryotic translation initiation factor 4H [Source:HGNC Symbol;Acc:HGNC:12741]	-1.1992096
EIF5A	eukaryotic translation initiation factor 5A [Source:HGNC Symbol;Acc:HGNC:3300]	1.2836711
ELAVL2	ELAV like neuron-specific RNA binding protein 2 [Source:HGNC Symbol;Acc:HGNC:3313]	1.1683027
ELF4	E74 like ETS transcription factor 4 [Source:HGNC Symbol;Acc:HGNC:3319]	-1.3874252
ELK1	ELK1, ETS transcription factor [Source:HGNC Symbol;Acc:HGNC:3321]	1.1894788
ELL2	elongation factor for RNA polymerase II 2 [Source:HGNC Symbol;Acc:HGNC:17064]	-1.2212749
ELMO2	engulfment and cell motility 2 [Source:HGNC Symbol;Acc:HGNC:17233]	-1.1928166
ELN	elastin [Source:HGNC Symbol;Acc:HGNC:3327]	-1.1903949
ELOVL1	ELOVL fatty acid elongase 1 [Source:HGNC Symbol;Acc:HGNC:14418]	-1.1828214
ELOVL4	ELOVL fatty acid elongase 4 [Source:HGNC Symbol;Acc:HGNC:14415]	1.2773852
ELTD1	adhesion G protein-coupled receptor L4	1.60149285
EMCN	endomucin [Source:HGNC Symbol;Acc:HGNC:16041]	1.4838629
EMG1	EMG1, N1-specific pseudouridine methyltransferase [Source:HGNC Symbol;Acc:HGNC:16912]	-1.1863599
EMILIN3	elastin microfibril interfacer 3 [Source:HGNC Symbol;Acc:HGNC:16123]	-1.2361492
EML1	echinoderm microtubule associated protein like 1 [Source:HGNC Symbol;Acc:HGNC:3330]	1.1799785
EML3	echinoderm microtubule associated protein like 3 [Source:HGNC Symbol;Acc:HGNC:26666]	1.3895365
EML5	echinoderm microtubule associated protein like 5 [Source:HGNC Symbol;Acc:HGNC:18197]	1.1199621
EML6	echinoderm microtubule associated protein like 6 [Source:HGNC Symbol;Acc:HGNC:35412]	1.1467834
EMP3	epithelial membrane protein 3 [Source:HGNC Symbol;Acc:HGNC:3335]	-1.223551
EMX2OS	EMX2 opposite strand/antisense RNA [Source:HGNC Symbol;Acc:HGNC:18511]	1.060556
ENAH	enabled homolog (Drosophila) [Source:HGNC Symbol;Acc:HGNC:18271]	1.1233554
ENDOD1	endonuclease domain containing 1 [Source:HGNC Symbol;Acc:HGNC:29129]	1.3225248
ENG	endoglin [Source:HGNC Symbol;Acc:HGNC:3349]	1.2331437
ENHO	energy homeostasis associated [Source:HGNC Symbol;Acc:HGNC:24838]	-1.1172864
ENO2	enolase 2 [Source:HGNC Symbol;Acc:HGNC:3353]	1.228249
ENPP5	ctonucleotide pyrophosphatase/phosphodiesterase 5 (putative) [Source:HGNC Symbol;Acc:HGNC:1371	1.0907967
ENTPD1	ectonucleoside triphosphate diphosphohydrolase 1 [Source:HGNC Symbol;Acc:HGNC:3363]	1.0548581
ENTPD2	ectonucleoside triphosphate diphosphohydrolase 2 [Source:HGNC Symbol;Acc:HGNC:3364]	-1.2220347
ENTPD5	ectonucleoside triphosphate diphosphohydrolase 5 [Source:HGNC Symbol;Acc:HGNC:3367]	1.0896422
ENTPD6	ectonucleoside triphosphate diphosphohydrolase 6 (putative) [Source:HGNC Symbol;Acc:HGNC:3368]	-1.1892891
ENTPD7	ectonucleoside triphosphate diphosphohydrolase 7 [Source:HGNC Symbol;Acc:HGNC:19745]	-1.1319438
EOMES	eomesodermin [Source:HGNC Symbol;Acc:HGNC:3372]	1.0848895
EP400NL	EP400 N-terminal like [Source:HGNC Symbol;Acc:HGNC:26602]	-1.1098138
EPB41L1	erythrocyte membrane protein band 4.1 like 1 [Source:HGNC Symbol;Acc:HGNC:3378]	-1.1830696
EPB49	dematin actin binding protein	1.3098614
EPC2	enhancer of polycomb homolog 2 [Source:HGNC Symbol;Acc:HGNC:24543]	1.2227528
EPHA2	EPH receptor A2 [Source:HGNC Symbol;Acc:HGNC:3386]	-1.2691249
EPHB4	EPH receptor B4 [Source:HGNC Symbol;Acc:HGNC:3395]	1.2669405
EPHX1	epoxide hydrolase 1 [Source:HGNC Symbol;Acc:HGNC:3401]	1.1209075
EPHX2	epoxide hydrolase 2 [Source:HGNC Symbol;Acc:HGNC:3402]	-1.0825481
EPM2A	epilepsy, progressive myoclonus type 2A, Lafora disease (laforin) [Source:HGNC Symbol;Acc:HGNC:3413	-1.1401364
EPRS	glutamyl-prolyl-tRNA synthetase [Source:HGNC Symbol;Acc:HGNC:3418]	1.7862285
EPS8L1	EPS8 like 1 [Source:HGNC Symbol;Acc:HGNC:21295]	-2.1828158
ERCC5	ERCC excision repair 5, endonuclease [Source:HGNC Symbol;Acc:HGNC:3437]	1.5793844

ERCC6	ERCC excision repair 6, chromatin remodeling factor [Source:HGNC Symbol;Acc:HGNC:3438]	1.4538237
ERCC6L	RCC excision repair 6 like, spindle assembly checkpoint helicase [Source:HGNC Symbol;Acc:HGNC:2079	1.2393249
ERF	ETS2 repressor factor [Source:HGNC Symbol;Acc:HGNC:3444]	1.4446235
ERI1	exoribonuclease 1 [Source:HGNC Symbol;Acc:HGNC:23994]	1.2119555
ERLEC1	endoplasmic reticulum lectin 1 [Source:HGNC Symbol;Acc:HGNC:25222]	1.2333854
ERLIN2	ER lipid raft associated 2 [Source:HGNC Symbol;Acc:HGNC:1356]	-1.1322606
ERP27	endoplasmic reticulum protein 27 [Source:HGNC Symbol;Acc:HGNC:26495]	1.0941213
ERP29	endoplasmic reticulum protein 29 [Source:HGNC Symbol;Acc:HGNC:13799]	1.1336172
ERRFI1	ERBB receptor feedback inhibitor 1 [Source:HGNC Symbol;Acc:HGNC:18185]	1.4890271
ERV3-1	endogenous retrovirus group 3 member 1 [Source:HGNC Symbol;Acc:HGNC:3454]	1.0638402
ERVV-2	endogenous retrovirus group V member 2 [Source:HGNC Symbol;Acc:HGNC:39051]	1.102484
ESAM	endothelial cell adhesion molecule [Source:HGNC Symbol;Acc:HGNC:17474]	1.197814
ESCO1	stablishment of sister chromatid cohesion N-acetyltransferase 1 [Source:HGNC Symbol;Acc:HGNC:2464	1.1774127
ESD	esterase D [Source:HGNC Symbol;Acc:HGNC:3465]	1.2406377
ETF1	eukaryotic translation termination factor 1 [Source:HGNC Symbol;Acc:HGNC:3477]	1.2201648
ETNK1	ethanolamine kinase 1 [Source:HGNC Symbol;Acc:HGNC:24649]	-1.1791266
ETNK2	ethanolamine kinase 2 [Source:HGNC Symbol;Acc:HGNC:25575]	1.1992302
ETS1	ETS proto-oncogene 1, transcription factor [Source:HGNC Symbol;Acc:HGNC:3488]	-1.9341439
ETS2	ETS proto-oncogene 2, transcription factor [Source:HGNC Symbol;Acc:HGNC:3489]	-1.6591127
ETV1	ETS variant 1 [Source:HGNC Symbol;Acc:HGNC:3490]	1.37350565
ETV3	ETS variant 3 [Source:HGNC Symbol;Acc:HGNC:3492]	-1.2142553
ETV3L	ETS variant 3 like [Source:HGNC Symbol;Acc:HGNC:33834]	-1.1681204
ETV6	ETS variant 6 [Source:HGNC Symbol;Acc:HGNC:3495]	-1.4474646
ETV7	ETS variant 7 [Source:HGNC Symbol;Acc:HGNC:18160]	-2.5817695
EVC2	EvC ciliary complex subunit 2 [Source:HGNC Symbol;Acc:HGNC:19747]	-1.30770775
EXO1	exonuclease 1 [Source:HGNC Symbol;Acc:HGNC:3511]	1.3401127
EXOC3	exocyst complex component 3 [Source:HGNC Symbol;Acc:HGNC:30378]	-1.0914062
EXOC3L2	exocyst complex component 3 like 2 [Source:HGNC Symbol;Acc:HGNC:30162]	1.2802544
EXOC3L4	exocyst complex component 3 like 4 [Source:HGNC Symbol;Acc:HGNC:20120]	-1.1522777
EXOC4	exocyst complex component 4 [Source:HGNC Symbol;Acc:HGNC:30389]	-1.0870023
EXOC6	exocyst complex component 6 [Source:HGNC Symbol;Acc:HGNC:23196]	1.5559857
EXOSC2	exosome component 2 [Source:HGNC Symbol;Acc:HGNC:17097]	1.1353625
EXT1	exostosin glycosyltransferase 1 [Source:HGNC Symbol;Acc:HGNC:3512]	-1.6120268
EYS	eyes shut homolog (Drosophila) [Source:HGNC Symbol;Acc:HGNC:21555]	1.12744795
EZR	ezrin [Source:HGNC Symbol;Acc:HGNC:12691]	-1.2226564
F11R	F11 receptor [Source:HGNC Symbol;Acc:HGNC:14685]	-1.3764684
F13A1	coagulation factor XIII A chain [Source:HGNC Symbol;Acc:HGNC:3531]	1.1117668
F13B	coagulation factor XIII B chain [Source:HGNC Symbol;Acc:HGNC:3534]	1.1365911
F2RL1	F2R like trypsin receptor 1 [Source:HGNC Symbol;Acc:HGNC:3538]	-2.863564
F3	coagulation factor III, tissue factor [Source:HGNC Symbol;Acc:HGNC:3541]	-4.0262837
FADS3	fatty acid desaturase 3 [Source:HGNC Symbol;Acc:HGNC:3576]	-1.3494232
FAM101A	family with sequence similarity 101 member A [Source:HGNC Symbol;Acc:HGNC:27051]	-2.4509072
FAM102A	family with sequence similarity 102 member A [Source:HGNC Symbol;Acc:HGNC:31419]	1.271664
FAM103A1	family with sequence similarity 103 member A1 [Source:HGNC Symbol;Acc:HGNC:31022]	-1.1619539
FAM106CP	family with sequence similarity 106 member C, pseudogene [Source:HGNC Symbol;Acc:HGNC:38396]	-1.0610504
FAM107A	family with sequence similarity 107 member A [Source:HGNC Symbol;Acc:HGNC:30827]	-1.1839452
FAM107B	family with sequence similarity 107 member B [Source:HGNC Symbol;Acc:HGNC:23726]	1.260671967
FAM108A1	abhydrolase domain containing 17A	1.1142707
FAM109A	family with sequence similarity 109 member A [Source:HGNC Symbol;Acc:HGNC:26509]	1.3533446
FAM110A	family with sequence similarity 110 member A [Source:HGNC Symbol;Acc:HGNC:16188]	-1.918569
FAM110C	family with sequence similarity 110 member C [Source:HGNC Symbol;Acc:HGNC:33340]	-1.1273499
FAM114A1	family with sequence similarity 114 member A1 [Source:HGNC Symbol;Acc:HGNC:25087]	-1.2067163
FAM115A	TRPM8 channel associated factor 1	1.3824047
FAM117A	family with sequence similarity 117 member A [Source:HGNC Symbol;Acc:HGNC:24179]	1.5592538
FAM117B	family with sequence similarity 117 member B [Source:HGNC Symbol;Acc:HGNC:14440]	1.3739785
FAM118A	family with sequence similarity 118 member A [Source:HGNC Symbol;Acc:HGNC:1313]	-1.6688662
FAM118B	family with sequence similarity 118 member B [Source:HGNC Symbol;Acc:HGNC:26110]	-1.3104504
FAM123B	APC membrane recruitment protein 1	-1.0628511
FAM124B	family with sequence similarity 124 member B [Source:HGNC Symbol;Acc:HGNC:26224]	1.5430545
FAM125B	multivesicular body subunit 12B	1.24662245
FAM129A	family with sequence similarity 129 member A [Source:HGNC Symbol;Acc:HGNC:16784]	-2.7730992
FAM134C	family with sequence similarity 134 member C [Source:HGNC Symbol;Acc:HGNC:27258]	-1.2070445
FAM138D	family with sequence similarity 138 member D [Source:HGNC Symbol;Acc:HGNC:33583]	1.1140243
FAM13A	family with sequence similarity 13 member A [Source:HGNC Symbol;Acc:HGNC:19367]	1.2420425
FAM13AOS	FAM13A antisense RNA 1	1.131773
FAM150B	family with sequence similarity 150 member B [Source:HGNC Symbol;Acc:HGNC:27683]	-1.448254
FAM156A	family with sequence similarity 156 member A [Source:HGNC Symbol;Acc:HGNC:30114]	1.1270336
FAM159A	family with sequence similarity 159 member A [Source:HGNC Symbol;Acc:HGNC:28757]	-1.3886821
FAM162B	family with sequence similarity 162 member B [Source:HGNC Symbol;Acc:HGNC:21549]	1.1003072
FAM165B	small integral membrane protein 11A	-1.1030816
FAM168B	family with sequence similarity 168 member B [Source:HGNC Symbol;Acc:HGNC:27016]	-1.1253661
FAM172BP	family with sequence similarity 172 member B, pseudogene [Source:HGNC Symbol;Acc:HGNC:34336]	-1.0506604
FAM174B	family with sequence similarity 174 member B [Source:HGNC Symbol;Acc:HGNC:34339]	2.1517942
FAM175A	family with sequence similarity 175 member A [Source:HGNC Symbol;Acc:HGNC:25829]	1.2244804
FAM184A	family with sequence similarity 184 member A [Source:HGNC Symbol;Acc:HGNC:20991]	-1.1058563
FAM185A	family with sequence similarity 185 member A [Source:HGNC Symbol;Acc:HGNC:22412]	1.1930611
FAM189A2	family with sequence similarity 189 member A2 [Source:HGNC Symbol;Acc:HGNC:24820]	-1.58587025
FAM189B	family with sequence similarity 189 member B [Source:HGNC Symbol;Acc:HGNC:1233]	1.0977932
FAM18B2-CDRT4	TVP23C-CDRT4 readthrough	1.131101
FAM194A	glutamate rich 6	-1.0867585
FAM198A	family with sequence similarity 198 member A [Source:HGNC Symbol;Acc:HGNC:24485]	1.125563
FAM19A1	y with sequence similarity 19 member A1, C-C motif chemokine like [Source:HGNC Symbol;Acc:HGNC:2	1.0895251
FAM19A2	y with sequence similarity 19 member A2, C-C motif chemokine like [Source:HGNC Symbol;Acc:HGNC:2	1.1190819
FAM19A4	y with sequence similarity 19 member A4, C-C motif chemokine like [Source:HGNC Symbol;Acc:HGNC:2	-1.1012409
FAM204A	family with sequence similarity 204 member A [Source:HGNC Symbol;Acc:HGNC:25794]	-1.1335496
FAM20A	family with sequence similarity 20 member A [Source:HGNC Symbol;Acc:HGNC:23015]	-1.1926072

FAM20B	family with sequence similarity 20 member B [Source:HGNC Symbol;Acc:HGNC:23017]	1.1993611
FAM21C	family with sequence similarity 21 member C [Source:HGNC Symbol;Acc:HGNC:23414]	-1.1797668
FAM22A	NUT family member 2A	-1.1235532
FAM22D	NUT family member 2D	-1.1509987
FAM26E	family with sequence similarity 26 member E [Source:HGNC Symbol;Acc:HGNC:21568]	1.1049467
FAM27A	family with sequence similarity 27 member C	1.3954761
FAM27E3	family with sequence similarity 27 member E3 [Source:HGNC Symbol;Acc:HGNC:28655]	1.9286112
FAM38A	piezo type mechanosensitive ion channel component 1	-1.3118521
FAM38B	piezo type mechanosensitive ion channel component 2	1.0849895
FAM43A	family with sequence similarity 43 member A [Source:HGNC Symbol;Acc:HGNC:26888]	1.1076658
FAM43B	family with sequence similarity 43 member B [Source:HGNC Symbol;Acc:HGNC:31791]	1.0879297
FAM45A	family with sequence similarity 45 member A [Source:HGNC Symbol;Acc:HGNC:31793]	1.25016075
FAM46B	family with sequence similarity 46 member B [Source:HGNC Symbol;Acc:HGNC:28273]	-1.3675963
FAM47A	family with sequence similarity 47 member A [Source:HGNC Symbol;Acc:HGNC:29962]	1.1372169
FAM48B2	SPT20 homolog, SAGA complex component-like 2	-1.0854527
FAM50B	family with sequence similarity 50 member B [Source:HGNC Symbol;Acc:HGNC:18789]	1.3184817
FAM59A	GRB2 associated regulator of MAPK1 subtype 1	1.0546161
FAM65A	family with sequence similarity 65 member A [Source:HGNC Symbol;Acc:HGNC:25836]	-1.6511898
FAM69A	family with sequence similarity 69 member A [Source:HGNC Symbol;Acc:HGNC:32213]	1.1170827
FAM69B	family with sequence similarity 69 member B [Source:HGNC Symbol;Acc:HGNC:28290]	1.1853181
FAM69C	family with sequence similarity 69 member C [Source:HGNC Symbol;Acc:HGNC:31729]	1.1429052
FAM72D	family with sequence similarity 72 member D [Source:HGNC Symbol;Acc:HGNC:33593]	1.2885058
FAM74A1	family with sequence similarity 74 member A1 [Source:HGNC Symbol;Acc:HGNC:32029]	1.6329926
FAM74A3	family with sequence similarity 74 member A3 [Source:HGNC Symbol;Acc:HGNC:32031]	1.3101009
FAM78A	family with sequence similarity 78 member A [Source:HGNC Symbol;Acc:HGNC:25465]	1.6823552
FAM82A1	regulator of microtubule dynamics 2	1.149435
FAM82B	regulator of microtubule dynamics 1	1.1300373
FAM84B	family with sequence similarity 84 member B [Source:HGNC Symbol;Acc:HGNC:24166]	-1.2021307
FAM86B1	family with sequence similarity 86 member B1 [Source:HGNC Symbol;Acc:HGNC:28268]	1.2195119
FAN1	FANCD2/FANCI-associated nuclease 1 [Source:HGNC Symbol;Acc:HGNC:29170]	1.2757679
FANCC	Fanconi anemia complementation group C [Source:HGNC Symbol;Acc:HGNC:3584]	-1.0678729
FANCE	Fanconi anemia complementation group E [Source:HGNC Symbol;Acc:HGNC:3586]	1.5591723
FANCI	Fanconi anemia complementation group I [Source:HGNC Symbol;Acc:HGNC:25568]	1.2206393
FANCM	Fanconi anemia complementation group M [Source:HGNC Symbol;Acc:HGNC:23168]	1.9093301
FANK1	fibronectin type III and ankyrin repeat domains 1 [Source:HGNC Symbol;Acc:HGNC:23527]	1.07005115
FAP	fibroblast activation protein alpha [Source:HGNC Symbol;Acc:HGNC:3590]	1.1096455
FARP1	FERM, ARH/RhoGEF and pleckstrin domain protein 1 [Source:HGNC Symbol;Acc:HGNC:3591]	-1.14852865
FASLG	Fas ligand [Source:HGNC Symbol;Acc:HGNC:11936]	1.0540824
FASTKD2	FAST kinase domains 2 [Source:HGNC Symbol;Acc:HGNC:29160]	1.2151566
FASTKD3	FAST kinase domains 3 [Source:HGNC Symbol;Acc:HGNC:28758]	1.1788312
FASTKD5	FAST kinase domains 5 [Source:HGNC Symbol;Acc:HGNC:25790]	-1.3081359
FAT3	FAT atypical cadherin 3 [Source:HGNC Symbol;Acc:HGNC:23112]	-1.1210229
FBLIM1	filamin binding LIM protein 1 [Source:HGNC Symbol;Acc:HGNC:24686]	1.2096055
FBLN7	fibulin 7 [Source:HGNC Symbol;Acc:HGNC:26740]	-1.1630198
FBRS	fibrosin [Source:HGNC Symbol;Acc:HGNC:20442]	-1.37614435
FBXL13	F-box and leucine rich repeat protein 13 [Source:HGNC Symbol;Acc:HGNC:21658]	1.1086694
FBXL2	F-box and leucine rich repeat protein 2 [Source:HGNC Symbol;Acc:HGNC:13598]	-1.1044519
FBXL4	F-box and leucine rich repeat protein 4 [Source:HGNC Symbol;Acc:HGNC:13601]	1.3806888
FBXL6	F-box and leucine rich repeat protein 6 [Source:HGNC Symbol;Acc:HGNC:13603]	-1.139332
FBXO10	F-box protein 10 [Source:HGNC Symbol;Acc:HGNC:13589]	-1.3518378
FBXO16	F-box protein 16 [Source:HGNC Symbol;Acc:HGNC:13618]	-1.117609
FBXO28	F-box protein 28 [Source:HGNC Symbol;Acc:HGNC:29046]	1.207461
FBXO31	F-box protein 31 [Source:HGNC Symbol;Acc:HGNC:16510]	-1.0754695
FBXO32	F-box protein 32 [Source:HGNC Symbol;Acc:HGNC:16731]	-1.240741
FBXO44	F-box protein 44 [Source:HGNC Symbol;Acc:HGNC:24847]	1.2248933
FBXO5	F-box protein 5 [Source:HGNC Symbol;Acc:HGNC:13584]	1.4749461
FBXO7	F-box protein 7 [Source:HGNC Symbol;Acc:HGNC:13586]	-1.1708609
FBXW12	F-box and WD repeat domain containing 12 [Source:HGNC Symbol;Acc:HGNC:20729]	-1.1190117
FCAMR	Fc fragment of IgA and IgM receptor [Source:HGNC Symbol;Acc:HGNC:24692]	-1.0647765
FCER1G	Fc fragment of IgE receptor Ig [Source:HGNC Symbol;Acc:HGNC:3611]	-1.1729678
FCGR2A	Fc fragment of IgG receptor IIa [Source:HGNC Symbol;Acc:HGNC:3616]	-1.104438
FCRLB	Fc receptor like B [Source:HGNC Symbol;Acc:HGNC:26431]	1.0973054
FECH	ferrochelatase [Source:HGNC Symbol;Acc:HGNC:3647]	-1.2471945
FEM1B	fem-1 homolog B [Source:HGNC Symbol;Acc:HGNC:3649]	-1.305102
FER1L4	fer-1 like family member 4, pseudogene [Source:HGNC Symbol;Acc:HGNC:15801]	-1.0951308
FEZF2	FEZ family zinc finger 2 [Source:HGNC Symbol;Acc:HGNC:13506]	1.1047986
FFAR2	free fatty acid receptor 2 [Source:HGNC Symbol;Acc:HGNC:4501]	-1.1299802
FGA	fibrinogen alpha chain [Source:HGNC Symbol;Acc:HGNC:3661]	1.0830665
FGD2	FYVE, RhoGEF and PH domain containing 2 [Source:HGNC Symbol;Acc:HGNC:3664]	-1.0993179
FGD5	FYVE, RhoGEF and PH domain containing 5 [Source:HGNC Symbol;Acc:HGNC:19117]	1.1117337
FGF1	fibroblast growth factor 1 [Source:HGNC Symbol;Acc:HGNC:3665]	-1.1520204
FGF10	fibroblast growth factor 10 [Source:HGNC Symbol;Acc:HGNC:3666]	1.0655729
FGF14	fibroblast growth factor 14 [Source:HGNC Symbol;Acc:HGNC:3671]	1.0926294
FGF22	fibroblast growth factor 22 [Source:HGNC Symbol;Acc:HGNC:3679]	-1.1360885
FGF5	fibroblast growth factor 5 [Source:HGNC Symbol;Acc:HGNC:3683]	-1.1695783
FGF7	fibroblast growth factor 7 [Source:HGNC Symbol;Acc:HGNC:3685]	1.2131114
FGF8	fibroblast growth factor 8 [Source:HGNC Symbol;Acc:HGNC:3686]	-1.1355691
FGFBP2	fibroblast growth factor binding protein 2 [Source:HGNC Symbol;Acc:HGNC:29451]	1.070655
FGFR1	fibroblast growth factor receptor 1 [Source:HGNC Symbol;Acc:HGNC:3688]	1.18937735
FGFR3	fibroblast growth factor receptor 3 [Source:HGNC Symbol;Acc:HGNC:3690]	1.1737288
FGFRL1	fibroblast growth factor receptor-like 1 [Source:HGNC Symbol;Acc:HGNC:3693]	1.3149416
FGR	FGR proto-oncogene, Src family tyrosine kinase [Source:HGNC Symbol;Acc:HGNC:3697]	-1.1315042
FHIT	fragile histidine triad [Source:HGNC Symbol;Acc:HGNC:3701]	1.0965632
FHL2	four and a half LIM domains 2 [Source:HGNC Symbol;Acc:HGNC:3703]	1.41391795
FIGLA	folliculogenesis specific bHLH transcription factor [Source:HGNC Symbol;Acc:HGNC:24669]	1.1515675
FIGNL2	fidgetin like 2 [Source:HGNC Symbol;Acc:HGNC:13287]	-1.7447519

FILIP1L	filamin A interacting protein 1 like [Source:HGNC Symbol;Acc:HGNC:24589]	-1.258265
FKBP10	FK506 binding protein 10 [Source:HGNC Symbol;Acc:HGNC:18169]	1.3032496
FKBP15	FK506 binding protein 15 [Source:HGNC Symbol;Acc:HGNC:23397]	-1.2941095
FKBP3	FK506 binding protein 3 [Source:HGNC Symbol;Acc:HGNC:3719]	1.3089318
FKRP	fukutin related protein [Source:HGNC Symbol;Acc:HGNC:17997]	-1.1508261
FKSG2	tumor protein, translationally-controlled 1 pseudogene 8	-1.3412471
FKSG83	VN1R10P vomeronasal 1 receptor 10 pseudogene	-1.1020849
FLCN	folliculin [Source:HGNC Symbol;Acc:HGNC:27310]	-1.132727
FLI1	Fli-1 proto-oncogene, ETS transcription factor [Source:HGNC Symbol;Acc:HGNC:3749]	-1.6368232
FLJ10489	long intergenic non-protein coding RNA 1181	1.0700691
FLJ10661	family with sequence similarity 86 member C1	1.1481845
FLJ12334	long intergenic non-protein coding RNA 954	-1.146683
FLJ14186	long intergenic non-protein coding RNA 1061	1.1104034
FLJ16124	uncharacterized LOC440867	-1.0694792
FLJ16341	long intergenic non-protein coding RNA 1185	1.106076
FLJ16734	uncharacterized LOC641928	-1.068993
FLJ22447	uncharacterized LOC400221	-1.1611881
FLJ25363	long intergenic non-protein coding RNA 1205	-1.133348
FLJ27352	chromosome 15 open reading frame 65	1.1378055
FLJ27502	uncharacterized LOC388406	1.1445552
FLJ31104	uncharacterized LOC441072	-2.1868813
FLJ31713	uncharacterized protein FLJ31713	-1.1838213
FLJ31715	uncharacterized FLJ31715	-1.2119403
FLJ31813	family with sequence similarity 21 member E, pseudogene	-1.14375555
FLJ39632	double homeobox A pseudogene 9	-1.1978531
FLJ42392	long intergenic non-protein coding RNA 548	-1.0953307
FLJ42709	NR2F1 antisense RNA 1	1.136296
FLNA	filamin A [Source:HGNC Symbol;Acc:HGNC:3754]	1.3844755
FLOT1	flotillin 1 [Source:HGNC Symbol;Acc:HGNC:3757]	1.1725982
FLT3LG	fms related tyrosine kinase 3 ligand [Source:HGNC Symbol;Acc:HGNC:3766]	-1.3767232
FLVCR1	feline leukemia virus subgroup C cellular receptor 1 [Source:HGNC Symbol;Acc:HGNC:24682]	-1.0953585
FMNL1	formin like 1 [Source:HGNC Symbol;Acc:HGNC:1212]	-1.3301752
FMNL3	formin like 3 [Source:HGNC Symbol;Acc:HGNC:23698]	-1.65875855
FMOD	fibromodulin [Source:HGNC Symbol;Acc:HGNC:3774]	-1.0742397
FMR1-AS1	FMR1 antisense RNA 1 [Source:HGNC Symbol;Acc:HGNC:39081]	-1.0894126
FN1	fibronectin 1 [Source:HGNC Symbol;Acc:HGNC:3778]	-1.2455231
FNBP1	formin binding protein 1 [Source:HGNC Symbol;Acc:HGNC:17069]	-1.50575015
FNBP1L	formin binding protein 1 like [Source:HGNC Symbol;Acc:HGNC:20851]	2.0401242
FNDC1	fibronectin type III domain containing 1 [Source:HGNC Symbol;Acc:HGNC:21184]	-1.096427
FNDC3B	fibronectin type III domain containing 3B [Source:HGNC Symbol;Acc:HGNC:24670]	-1.2591299
FNDC4	fibronectin type III domain containing 4 [Source:HGNC Symbol;Acc:HGNC:20239]	-1.3297535
FOLR3	folate receptor 3 [Source:HGNC Symbol;Acc:HGNC:3795]	-1.0726671
FOSL1	FOS like 1, AP-1 transcription factor subunit [Source:HGNC Symbol;Acc:HGNC:13718]	-1.2878714
FOSL2	FOS like 2, AP-1 transcription factor subunit [Source:HGNC Symbol;Acc:HGNC:3798]	-1.7207865
FOXA2	forkhead box A2 [Source:HGNC Symbol;Acc:HGNC:5022]	-1.0560454
FOXC1	forkhead box C1 [Source:HGNC Symbol;Acc:HGNC:3800]	1.5014796
FOXC2	forkhead box C2 [Source:HGNC Symbol;Acc:HGNC:3801]	-1.3353944
FOXD1	forkhead box D1 [Source:HGNC Symbol;Acc:HGNC:3802]	1.0579338
FOXE3	forkhead box E3 [Source:HGNC Symbol;Acc:HGNC:3808]	-1.0983759
FOXF1	forkhead box F1 [Source:HGNC Symbol;Acc:HGNC:3809]	-1.7702779
FOXI2	forkhead box I2 [Source:HGNC Symbol;Acc:HGNC:32448]	-1.2707447
FOXJ1	forkhead box J1 [Source:HGNC Symbol;Acc:HGNC:3816]	-1.4464251
FOXJ2	forkhead box J2 [Source:HGNC Symbol;Acc:HGNC:24818]	1.193658
FOXK1	forkhead box K1 [Source:HGNC Symbol;Acc:HGNC:23480]	-1.158866
FOXK2	forkhead box K2 [Source:HGNC Symbol;Acc:HGNC:6036]	-1.1412911
FOXL1	forkhead box L1 [Source:HGNC Symbol;Acc:HGNC:3817]	-1.1813451
FOXL2	forkhead box L2 [Source:HGNC Symbol;Acc:HGNC:1092]	1.0664017
FOXN2	forkhead box N2 [Source:HGNC Symbol;Acc:HGNC:5281]	1.4048625
FOXO1	forkhead box O1 [Source:HGNC Symbol;Acc:HGNC:3819]	-1.5311533
FOXO3	forkhead box O3 [Source:HGNC Symbol;Acc:HGNC:3821]	-1.4313021
FOXO4	forkhead box O4 [Source:HGNC Symbol;Acc:HGNC:7139]	1.1233342
FOXP1	forkhead box P1 [Source:HGNC Symbol;Acc:HGNC:3823]	-1.2593139
FRA10AC1	site, folic acid type, rare, fra(10)(q23.3) or fra(10)(q24.2) candidate 1 [Source:HGNC Symbol;Acc:HGNC	1.0955932
FRAS1	Fraser extracellular matrix complex subunit 1 [Source:HGNC Symbol;Acc:HGNC:19185]	1.1359186
FRAT1	frequently rearranged in advanced T-cell lymphomas 1 [Source:HGNC Symbol;Acc:HGNC:3944]	1.1383861
FREM3	FRAS1 related extracellular matrix 3 [Source:HGNC Symbol;Acc:HGNC:25172]	1.2057382
FRG1	FSHD region gene 1 [Source:HGNC Symbol;Acc:HGNC:3954]	1.2086794
FRK	fyn related Src family tyrosine kinase [Source:HGNC Symbol;Acc:HGNC:3955]	1.0634891
FRMD3	FERM domain containing 3 [Source:HGNC Symbol;Acc:HGNC:24125]	2.1928285
FRMD4B	FERM domain containing 4B [Source:HGNC Symbol;Acc:HGNC:24886]	1.4652362
FRMD5	FERM domain containing 5 [Source:HGNC Symbol;Acc:HGNC:28214]	1.1846285
FRMD6	FERM domain containing 6 [Source:HGNC Symbol;Acc:HGNC:19839]	-1.2973115
FRMD8	FERM domain containing 8 [Source:HGNC Symbol;Acc:HGNC:25462]	-1.2110289
FRMPD1	FERM and PDZ domain containing 1 [Source:HGNC Symbol;Acc:HGNC:29159]	-1.0941118
FRY	FRY microtubule binding protein [Source:HGNC Symbol;Acc:HGNC:20367]	1.457712275
FSHB	follicle stimulating hormone beta subunit [Source:HGNC Symbol;Acc:HGNC:3964]	1.1080532
FSHR	follicle stimulating hormone receptor [Source:HGNC Symbol;Acc:HGNC:3969]	-1.0668025
FST	follistatin [Source:HGNC Symbol;Acc:HGNC:3971]	1.1812028
FSTL3	follistatin like 3 [Source:HGNC Symbol;Acc:HGNC:3973]	-2.3305798
FTHL17	ferritin heavy chain like 17 [Source:HGNC Symbol;Acc:HGNC:3987]	-1.1932043
FTLP10	ferritin light chain pseudogene 10 [Source:HGNC Symbol;Acc:HGNC:37959]	1.1159812
FTSJ1	FtsJ RNA methyltransferase homolog 1 (E. coli) [Source:HGNC Symbol;Acc:HGNC:13254]	-1.2087901
FTSJD2	cap methyltransferase 1	-1.1590037
FUCA1	fucosidase, alpha-L- 1, tissue [Source:HGNC Symbol;Acc:HGNC:4006]	-1.0915387
FUNDC2	FUN14 domain containing 2 [Source:HGNC Symbol;Acc:HGNC:24925]	-1.1151011
FURIN	furin, paired basic amino acid cleaving enzyme [Source:HGNC Symbol;Acc:HGNC:8568]	1.1975517

FUT10	fucosyltransferase 10 [Source:HGNC Symbol;Acc:HGNC:19234]	1.1061447
FUT4	fucosyltransferase 4 [Source:HGNC Symbol;Acc:HGNC:4015]	-1.1834342
FUT8	fucosyltransferase 8 [Source:HGNC Symbol;Acc:HGNC:4019]	1.2568308
FXN	frataxin [Source:HGNC Symbol;Acc:HGNC:3951]	-1.111749
FXR1	FMR1 autosomal homolog 1 [Source:HGNC Symbol;Acc:HGNC:4023]	1.3944286
FXR2	FMR1 autosomal homolog 2 [Source:HGNC Symbol;Acc:HGNC:4024]	1.3178192
FXYD3	FXYD domain containing ion transport regulator 3 [Source:HGNC Symbol;Acc:HGNC:4027]	-1.0584437
FXYD6	FXYD domain containing ion transport regulator 6 [Source:HGNC Symbol;Acc:HGNC:4030]	-1.2307544
FYTTD1	forty-two-three domain containing 1 [Source:HGNC Symbol;Acc:HGNC:25407]	1.2925241
FZD2	frizzled class receptor 2 [Source:HGNC Symbol;Acc:HGNC:4040]	1.298762
FZD3	frizzled class receptor 3 [Source:HGNC Symbol;Acc:HGNC:4041]	1.1348747
FZD8	frizzled class receptor 8 [Source:HGNC Symbol;Acc:HGNC:4046]	1.3511535
FZR1	fizzy/cell division cycle 20 related 1 [Source:HGNC Symbol;Acc:HGNC:24824]	-1.4614496
G0S2	G0/G1 switch 2 [Source:HGNC Symbol;Acc:HGNC:30229]	-2.1152213
G2E3	G2/M-phase specific E3 ubiquitin protein ligase [Source:HGNC Symbol;Acc:HGNC:20338]	1.33674705
G3BP2	G3BP stress granule assembly factor 2 [Source:HGNC Symbol;Acc:HGNC:30291]	1.319036
G6PC3	glucose-6-phosphatase catalytic subunit 3 [Source:HGNC Symbol;Acc:HGNC:24861]	1.3342971
G6PD	glucose-6-phosphate dehydrogenase [Source:HGNC Symbol;Acc:HGNC:4057]	1.6271123
GAA	glucosidase alpha, acid [Source:HGNC Symbol;Acc:HGNC:4065]	1.1725811
GABBR1	gamma-aminobutyric acid type B receptor subunit 1 [Source:HGNC Symbol;Acc:HGNC:4070]	-1.23852145
GABPB1	GA binding protein transcription factor beta subunit 1 [Source:HGNC Symbol;Acc:HGNC:4074]	-1.5324199
GABRA2	gamma-aminobutyric acid type A receptor alpha2 subunit [Source:HGNC Symbol;Acc:HGNC:4076]	1.1060115
GABRA3	gamma-aminobutyric acid type A receptor alpha3 subunit [Source:HGNC Symbol;Acc:HGNC:4077]	-1.07005
GABRA4	gamma-aminobutyric acid type A receptor alpha4 subunit [Source:HGNC Symbol;Acc:HGNC:4078]	1.0839996
GABRB1	gamma-aminobutyric acid type A receptor beta1 subunit [Source:HGNC Symbol;Acc:HGNC:4081]	-1.178683
GABRB2	gamma-aminobutyric acid type A receptor beta2 subunit [Source:HGNC Symbol;Acc:HGNC:4082]	1.1179624
GABRD	gamma-aminobutyric acid type A receptor delta subunit [Source:HGNC Symbol;Acc:HGNC:4084]	-1.0941114
GABRR1	gamma-aminobutyric acid type A receptor rho1 subunit [Source:HGNC Symbol;Acc:HGNC:4090]	-1.0620341
GAD2	glutamate decarboxylase 2 [Source:HGNC Symbol;Acc:HGNC:4093]	1.1215197
GADD45GIP1	GADD45G interacting protein 1 [Source:HGNC Symbol;Acc:HGNC:29996]	-1.129729
GAFA2	FGF-2 activity-associated protein 2	-1.142057
GAK	cyclin G associated kinase [Source:HGNC Symbol;Acc:HGNC:4113]	-1.1412835
GAL3ST2	galactose-3-O-sulfotransferase 2 [Source:HGNC Symbol;Acc:HGNC:24869]	-1.0833114
GALNT2	polypeptide N-acetylgalactosaminyltransferase 2 [Source:HGNC Symbol;Acc:HGNC:4124]	1.1204106
GALNT6	polypeptide N-acetylgalactosaminyltransferase 6 [Source:HGNC Symbol;Acc:HGNC:4128]	-1.2058042
GALNTL6	polypeptide N-acetylgalactosaminyltransferase-like 6 [Source:HGNC Symbol;Acc:HGNC:33844]	1.0553868
GALR2	galanin receptor 2 [Source:HGNC Symbol;Acc:HGNC:4133]	-1.1024992
GANAB	glucosidase II alpha subunit [Source:HGNC Symbol;Acc:HGNC:4138]	1.0882472
GANC	glucosidase alpha, neutral C [Source:HGNC Symbol;Acc:HGNC:4139]	-1.1853673
GAPVD1	GTPase activating protein and VPS9 domains 1 [Source:HGNC Symbol;Acc:HGNC:23375]	1.1831497
GAS2	growth arrest specific 2 [Source:HGNC Symbol;Acc:HGNC:4167]	1.0821108
GAS2L1	growth arrest specific 2 like 1 [Source:HGNC Symbol;Acc:HGNC:16955]	1.243758
GAS2L2	growth arrest specific 2 like 2 [Source:HGNC Symbol;Acc:HGNC:24846]	1.3586638
GAS6	growth arrest specific 6 [Source:HGNC Symbol;Acc:HGNC:4168]	-1.2520485
GATA2	GATA binding protein 2 [Source:HGNC Symbol;Acc:HGNC:4171]	1.6805689
GATA3	GATA binding protein 3 [Source:HGNC Symbol;Acc:HGNC:4172]	1.3656744
GATA6	GATA binding protein 6 [Source:HGNC Symbol;Acc:HGNC:4174]	-1.6315202
GATS	GATS, stromal antigen 3 opposite strand [Source:HGNC Symbol;Acc:HGNC:29954]	-1.1984142
GATSL3	GATS protein like 3 [Source:HGNC Symbol;Acc:HGNC:34423]	-1.6028535
GBA2	glucosylceramidase beta 2 [Source:HGNC Symbol;Acc:HGNC:18986]	1.9981656
GBP1	guanylate binding protein 1 [Source:HGNC Symbol;Acc:HGNC:4182]	-1.4913276
GBP2	guanylate binding protein 2 [Source:HGNC Symbol;Acc:HGNC:4183]	-1.3408135
GBP4	guanylate binding protein 4 [Source:HGNC Symbol;Acc:HGNC:20480]	-1.6045449
GCC1	GRIP and coiled-coil domain containing 1 [Source:HGNC Symbol;Acc:HGNC:19095]	-1.1462204
GCG	glucagon [Source:HGNC Symbol;Acc:HGNC:4191]	1.0756333
GCGR	glucagon receptor [Source:HGNC Symbol;Acc:HGNC:4192]	-1.0502907
GCH1	GTP cyclohydrolase 1 [Source:HGNC Symbol;Acc:HGNC:4193]	-1.33036155
GCKR	glucokinase regulator [Source:HGNC Symbol;Acc:HGNC:4196]	-1.2296566
GCLC	glutamate-cysteine ligase catalytic subunit [Source:HGNC Symbol;Acc:HGNC:4311]	1.3372725
GCN1L1	GCN1, eIF2 alpha kinase activator homolog	1.1143482
GCNT2	saminyl (N-acetyl) transferase 2, I-branching enzyme (I blood group) [Source:HGNC Symbol;Acc:HGNC:	-1.2319936
GCNT3	glucosaminyl (N-acetyl) transferase 3, mucin type [Source:HGNC Symbol;Acc:HGNC:4205]	1.1975874
GCOM1	GRINL1A complex locus 1 [Source:HGNC Symbol;Acc:HGNC:26424]	-1.5312004
GCRG224	gastric cancer-related gene GCRG224	-1.0801486
GDA	guanine deaminase [Source:HGNC Symbol;Acc:HGNC:4212]	1.0577004
GDF6	growth differentiation factor 6 [Source:HGNC Symbol;Acc:HGNC:4221]	1.3338702
GDI1	GDP dissociation inhibitor 1 [Source:HGNC Symbol;Acc:HGNC:4226]	1.2056956
GDNF	glial cell derived neurotrophic factor [Source:HGNC Symbol;Acc:HGNC:4232]	1.09753355
GEMC1	geminin coiled-coil domain containing	-1.0938087
GEMIN6	gem nuclear organelle associated protein 6 [Source:HGNC Symbol;Acc:HGNC:20044]	1.2190032
GEMIN8P4	gem nuclear organelle associated protein 8 pseudogene 4 [Source:HGNC Symbol;Acc:HGNC:37979]	-1.0719826
GET4	golgi to ER traffic protein 4 [Source:HGNC Symbol;Acc:HGNC:21690]	-1.2364275
GFM1	G elongation factor mitochondrial 1 [Source:HGNC Symbol;Acc:HGNC:13780]	1.2284544
GFOD1	glucose-fructose oxidoreductase domain containing 1 [Source:HGNC Symbol;Acc:HGNC:21096]	1.7589302
GFPT2	glutamine-fructose-6-phosphate transaminase 2 [Source:HGNC Symbol;Acc:HGNC:4242]	-2.2467678
GFRA1	GDNF family receptor alpha 1 [Source:HGNC Symbol;Acc:HGNC:4243]	-1.20831875
GFRA3	GDNF family receptor alpha 3 [Source:HGNC Symbol;Acc:HGNC:4245]	-1.1580638
GGA1	i associated, gamma adaptin ear containing, ARF binding protein 1 [Source:HGNC Symbol;Acc:HGNC:17	-1.1540504
GGA2	i associated, gamma adaptin ear containing, ARF binding protein 2 [Source:HGNC Symbol;Acc:HGNC:16	1.1873485
GGCT	gamma-glutamylcyclotransferase [Source:HGNC Symbol;Acc:HGNC:21705]	1.2692438
GGT3P	gamma-glutamyltransferase 3 pseudogene [Source:HGNC Symbol;Acc:HGNC:4252]	-1.1473093
GGT5	gamma-glutamyltransferase 5 [Source:HGNC Symbol;Acc:HGNC:4260]	1.139714
GGT7	gamma-glutamyltransferase 7 [Source:HGNC Symbol;Acc:HGNC:4259]	-1.141678
GGTA1P	glycoprotein, alpha-galactosyltransferase 1 pseudogene [Source:HGNC Symbol;Acc:HGNC:4253]	-1.0713525
GGTLC2	gamma-glutamyltransferase light chain 2 [Source:HGNC Symbol;Acc:HGNC:18596]	-1.1979313
GHRH	growth hormone releasing hormone [Source:HGNC Symbol;Acc:HGNC:4265]	-1.0990652

GHRLOS	ghrelin opposite strand/antisense RNA [Source:HGNC Symbol;Acc:HGNC:33885]	-1.3410299
GIMAP1	GTPase, IMAP family member 1 [Source:HGNC Symbol;Acc:HGNC:23237]	1.3049476
GIMAP7	GTPase, IMAP family member 7 [Source:HGNC Symbol;Acc:HGNC:22404]	1.7194321
GIN1	gypsy retrotransposon integrase 1 [Source:HGNC Symbol;Acc:HGNC:25959]	1.1487808
GIPR	gastric inhibitory polypeptide receptor [Source:HGNC Symbol;Acc:HGNC:4271]	-1.5035352
GIT1	GIT ArfGAP 1 [Source:HGNC Symbol;Acc:HGNC:4272]	1.1951913
GJA1	gap junction protein alpha 1 [Source:HGNC Symbol;Acc:HGNC:4274]	1.5761799
GJA8	gap junction protein alpha 8 [Source:HGNC Symbol;Acc:HGNC:4281]	-1.0595661
GJA9	gap junction protein alpha 9 [Source:HGNC Symbol;Acc:HGNC:19155]	1.2159333
GJB4	gap junction protein beta 4 [Source:HGNC Symbol;Acc:HGNC:4286]	1.0828336
GJD3	gap junction protein delta 3 [Source:HGNC Symbol;Acc:HGNC:19147]	1.1836607
GKAP1	G kinase anchoring protein 1 [Source:HGNC Symbol;Acc:HGNC:17496]	1.1877992
GKN2	gastrokine 2 [Source:HGNC Symbol;Acc:HGNC:24588]	1.0664618
GLB1L	galactosidase beta 1 like [Source:HGNC Symbol;Acc:HGNC:28129]	-1.2052326
GLCE	glucuronic acid epimerase [Source:HGNC Symbol;Acc:HGNC:17855]	1.4581761
GLI1	GLI family zinc finger 1 [Source:HGNC Symbol;Acc:HGNC:4317]	-1.0931576
GLI2	GLI family zinc finger 2 [Source:HGNC Symbol;Acc:HGNC:4318]	-1.1622512
GLIPR1L1	GLI pathogenesis related 1 like 1 [Source:HGNC Symbol;Acc:HGNC:28392]	1.08615
GLIPR2	GLI pathogenesis related 2 [Source:HGNC Symbol;Acc:HGNC:18007]	-1.1371504
GLIS2	GLIS family zinc finger 2 [Source:HGNC Symbol;Acc:HGNC:29450]	-2.2590895
GLOD5	glyoxalase domain containing 5 [Source:HGNC Symbol;Acc:HGNC:33358]	-1.0766553
GLRA2	glycine receptor alpha 2 [Source:HGNC Symbol;Acc:HGNC:4327]	1.1100254
GLRB	glycine receptor beta [Source:HGNC Symbol;Acc:HGNC:4329]	1.1112983
GLT25D1	collagen beta(1-O)galactosyltransferase 1	-1.5147244
GLTP	glycolipid transfer protein [Source:HGNC Symbol;Acc:HGNC:24867]	-1.2272491
GLYAT	glycine-N-acyltransferase [Source:HGNC Symbol;Acc:HGNC:13734]	-1.1158724
GLYATL3	glycine-N-acyltransferase like 3 [Source:HGNC Symbol;Acc:HGNC:21349]	-1.1042626
GLYCAM1	lycosylation dependent cell adhesion molecule 1 (pseudogene) [Source:HGNC Symbol;Acc:HGNC:1802	1.0966995
GLYR1	glyoxylate reductase 1 homolog [Source:HGNC Symbol;Acc:HGNC:24434]	1.0818541
GM2A	GM2 ganglioside activator [Source:HGNC Symbol;Acc:HGNC:4367]	1.102789
GMCL1	germ cell-less, spermatogenesis associated 1 [Source:HGNC Symbol;Acc:HGNC:23843]	1.209213
GMDS	GDP-mannose 4,6-dehydratase [Source:HGNC Symbol;Acc:HGNC:4369]	-1.2319537
GMFB	glia maturation factor beta [Source:HGNC Symbol;Acc:HGNC:4373]	1.7714572
GMFG	glia maturation factor gamma [Source:HGNC Symbol;Acc:HGNC:4374]	1.1179016
GMNN	geminin, DNA replication inhibitor [Source:HGNC Symbol;Acc:HGNC:17493]	1.3368529
GMPR2	guanosine monophosphate reductase 2 [Source:HGNC Symbol;Acc:HGNC:4377]	1.091237
GNA12	G protein subunit alpha 12 [Source:HGNC Symbol;Acc:HGNC:4380]	-1.1783155
GNA13	G protein subunit alpha 13 [Source:HGNC Symbol;Acc:HGNC:4381]	1.1752976
GNAI1	G protein subunit alpha i1 [Source:HGNC Symbol;Acc:HGNC:4384]	1.294164
GNAT3	G protein subunit alpha transducin 3 [Source:HGNC Symbol;Acc:HGNC:22800]	1.0792123
GNAZ	G protein subunit alpha z [Source:HGNC Symbol;Acc:HGNC:4395]	1.2765317
GNB3	G protein subunit beta 3 [Source:HGNC Symbol;Acc:HGNC:4400]	1.099727
GNG10	G protein subunit gamma 10 [Source:HGNC Symbol;Acc:HGNC:4402]	1.3218697
GNG13	G protein subunit gamma 13 [Source:HGNC Symbol;Acc:HGNC:14131]	-1.2102461
GNGT1	G protein subunit gamma transducin 1 [Source:HGNC Symbol;Acc:HGNC:4411]	1.0531642
GNGT2	G protein subunit gamma transducin 2 [Source:HGNC Symbol;Acc:HGNC:4412]	-1.1883078
GNL1	G protein nucleolar 1 (putative) [Source:HGNC Symbol;Acc:HGNC:4413]	-1.0955336
GNL3L	G protein nucleolar 3 like [Source:HGNC Symbol;Acc:HGNC:25553]	-1.2965157
GNL3LP1	G protein nucleolar 3 like pseudogene 1 [Source:HGNC Symbol;Acc:HGNC:25733]	-1.3284632
GNPDA1	glucosamine-6-phosphate deaminase 1 [Source:HGNC Symbol;Acc:HGNC:4417]	1.178659
GNPDA2	glucosamine-6-phosphate deaminase 2 [Source:HGNC Symbol;Acc:HGNC:21526]	1.2863
GOLGA2	golgin A2 [Source:HGNC Symbol;Acc:HGNC:4425]	1.156772
GOLGA6A	golgin A6 family member A [Source:HGNC Symbol;Acc:HGNC:13567]	-1.0768428
GOLGA6L6	golgin A6 family-like 6 [Source:HGNC Symbol;Acc:HGNC:37225]	-1.428263733
GOLGA7	golgin A7 [Source:HGNC Symbol;Acc:HGNC:24876]	-1.1403636
GOLGA8A	golgin A8 family member A [Source:HGNC Symbol;Acc:HGNC:31972]	-1.1058573
GOLGA8F	golgin A8 family member F [Source:HGNC Symbol;Acc:HGNC:32378]	1.0791911
GOLPH3	golgi phosphoprotein 3 [Source:HGNC Symbol;Acc:HGNC:15452]	-1.3405111
GOLPH3L	golgi phosphoprotein 3 like [Source:HGNC Symbol;Acc:HGNC:24882]	-1.5583141
GORASP1	golgi reassembly stacking protein 1 [Source:HGNC Symbol;Acc:HGNC:16769]	-1.2060636
GORASP2	golgi reassembly stacking protein 2 [Source:HGNC Symbol;Acc:HGNC:17500]	-1.2973789
GOSR2	golgi SNAP receptor complex member 2 [Source:HGNC Symbol;Acc:HGNC:4431]	-1.1556668
GOT1	glutamic-oxaloacetic transaminase 1 [Source:HGNC Symbol;Acc:HGNC:4432]	-1.0936532
GP1BA	glycoprotein Ib platelet alpha subunit [Source:HGNC Symbol;Acc:HGNC:4439]	-1.148358
GPA33	glycoprotein A33 [Source:HGNC Symbol;Acc:HGNC:4445]	-1.0871614
GPC2	glypican 2 [Source:HGNC Symbol;Acc:HGNC:4450]	1.283777
GPC3	glypican 3 [Source:HGNC Symbol;Acc:HGNC:4451]	-1.1009786
GPC4	glypican 4 [Source:HGNC Symbol;Acc:HGNC:4452]	-1.1483101
GPC5	glypican 5 [Source:HGNC Symbol;Acc:HGNC:4453]	1.1147877
GPHA2	glycoprotein hormone alpha 2 [Source:HGNC Symbol;Acc:HGNC:18054]	-1.15187
GPHB5	glycoprotein hormone beta 5 [Source:HGNC Symbol;Acc:HGNC:18055]	-1.2113158
GPI	glucose-6-phosphate isomerase [Source:HGNC Symbol;Acc:HGNC:4458]	-1.1139575
GPM6A	glycoprotein M6A [Source:HGNC Symbol;Acc:HGNC:4460]	1.0950427
GPN3	GPN-loop GTPase 3 [Source:HGNC Symbol;Acc:HGNC:30186]	1.2661116
GPR101	G protein-coupled receptor 101 [Source:HGNC Symbol;Acc:HGNC:14963]	1.1341285
GPR107	G protein-coupled receptor 107 [Source:HGNC Symbol;Acc:HGNC:17830]	-1.2828815
GPR108	G protein-coupled receptor 108 [Source:HGNC Symbol;Acc:HGNC:17829]	-1.2661247
GPR110	adhesion G protein-coupled receptor F1	1.1282552
GPR116	adhesion G protein-coupled receptor F5	1.0807412
GPR12	G protein-coupled receptor 12 [Source:HGNC Symbol;Acc:HGNC:4466]	1.0790191
GPR123	adhesion G protein-coupled receptor A1	-1.1358241
GPR126	adhesion G protein-coupled receptor G6	1.7564493
GPR135	G protein-coupled receptor 135 [Source:HGNC Symbol;Acc:HGNC:19991]	1.3816612
GPR137B	G protein-coupled receptor 137B [Source:HGNC Symbol;Acc:HGNC:11862]	-1.1565648
GPR148	G protein-coupled receptor 148 [Source:HGNC Symbol;Acc:HGNC:23623]	1.0794839
GPR151	G protein-coupled receptor 151 [Source:HGNC Symbol;Acc:HGNC:23624]	1.0836984

GPR153	G protein-coupled receptor 153 [Source:HGNC Symbol;Acc:HGNC:23618]	1.1023201
GPR156	G protein-coupled receptor 156 [Source:HGNC Symbol;Acc:HGNC:20844]	-1.108239
GPR161	G protein-coupled receptor 161 [Source:HGNC Symbol;Acc:HGNC:23694]	-1.1451824
GPR172A	solute carrier family 52 member 2	-1.2120808
GPR21	G protein-coupled receptor 21 [Source:HGNC Symbol;Acc:HGNC:4476]	-1.1219139
GPR26	G protein-coupled receptor 26 [Source:HGNC Symbol;Acc:HGNC:4481]	1.0973374
GPR32	G protein-coupled receptor 32 [Source:HGNC Symbol;Acc:HGNC:4487]	-1.1807947
GPR37L1	G protein-coupled receptor 37 like 1 [Source:HGNC Symbol;Acc:HGNC:14923]	-1.4936528
GPR4	G protein-coupled receptor 4 [Source:HGNC Symbol;Acc:HGNC:4497]	1.570813
GPR56	adhesion G protein-coupled receptor G1	-1.3973953
GPR68	G protein-coupled receptor 68 [Source:HGNC Symbol;Acc:HGNC:4519]	-7.8000216
GPR88	G protein-coupled receptor 88 [Source:HGNC Symbol;Acc:HGNC:4539]	-1.1286671
GPR97	adhesion G protein-coupled receptor G3	-1.1021633
GPRC5A	G protein-coupled receptor class C group 5 member A [Source:HGNC Symbol;Acc:HGNC:9836]	-1.17196
GPRC5B	G protein-coupled receptor class C group 5 member B [Source:HGNC Symbol;Acc:HGNC:13308]	-1.5166233
GPS2	G protein pathway suppressor 2 [Source:HGNC Symbol;Acc:HGNC:4550]	-1.1679187
GPX2	glutathione peroxidase 2 [Source:HGNC Symbol;Acc:HGNC:4554]	-1.0988263
GPX5	glutathione peroxidase 5 [Source:HGNC Symbol;Acc:HGNC:4557]	-1.1662979
GPX7	glutathione peroxidase 7 [Source:HGNC Symbol;Acc:HGNC:4559]	-1.1851418
GPX8	glutathione peroxidase 8 (putative) [Source:HGNC Symbol;Acc:HGNC:33100]	1.3637805
GRAMD1B	GRAM domain containing 1B [Source:HGNC Symbol;Acc:HGNC:29214]	1.1372566
GRAMD3	GRAM domain containing 3 [Source:HGNC Symbol;Acc:HGNC:24911]	-1.5446496
GRAP	GRB2-related adaptor protein [Source:HGNC Symbol;Acc:HGNC:4562]	1.3193944
GRASP	ıeral receptor for phosphoinositides 1 associated scaffold protein [Source:HGNC Symbol;Acc:HGNC:187	1.2411804
GRB10	growth factor receptor bound protein 10 [Source:HGNC Symbol;Acc:HGNC:4564]	-1.2222267
GRB14	growth factor receptor bound protein 14 [Source:HGNC Symbol;Acc:HGNC:4565]	1.2925
GRB7	growth factor receptor bound protein 7 [Source:HGNC Symbol;Acc:HGNC:4567]	-1.1898693
GRHL1	grainyhead like transcription factor 1 [Source:HGNC Symbol;Acc:HGNC:17923]	1.2377442
GRIA1	glutamate ionotropic receptor AMPA type subunit 1 [Source:HGNC Symbol;Acc:HGNC:4571]	1.2061715
GRIA2	glutamate ionotropic receptor AMPA type subunit 2 [Source:HGNC Symbol;Acc:HGNC:4572]	1.0727841
GRIA4	glutamate ionotropic receptor AMPA type subunit 4 [Source:HGNC Symbol;Acc:HGNC:4574]	1.083477
GRID1	glutamate ionotropic receptor delta type subunit 1 [Source:HGNC Symbol;Acc:HGNC:4575]	1.1553308
GRID2	glutamate ionotropic receptor delta type subunit 2 [Source:HGNC Symbol;Acc:HGNC:4576]	-1.1990379
GRID2IP	Grid2 interacting protein [Source:HGNC Symbol;Acc:HGNC:18464]	-1.1161969
GRIK1-AS1	GRIK1 antisense RNA 1 [Source:HGNC Symbol;Acc:HGNC:16458]	-1.0956793
GRIK2	glutamate ionotropic receptor kainate type subunit 2 [Source:HGNC Symbol;Acc:HGNC:4580]	-1.1011533
GRIN2B	glutamate ionotropic receptor NMDA type subunit 2B [Source:HGNC Symbol;Acc:HGNC:4586]	1.1540288
GRIN2C	glutamate ionotropic receptor NMDA type subunit 2C [Source:HGNC Symbol;Acc:HGNC:4587]	-1.2408013
GRIN3B	glutamate ionotropic receptor NMDA type subunit 3B [Source:HGNC Symbol;Acc:HGNC:16768]	-1.119777
GRINA	ımate ionotropic receptor NMDA type subunit associated protein 1 [Source:HGNC Symbol;Acc:HGNC:4	-1.5606254
GRIP1	glutamate receptor interacting protein 1 [Source:HGNC Symbol;Acc:HGNC:18708]	1.0646765
GRIP2	glutamate receptor interacting protein 2 [Source:HGNC Symbol;Acc:HGNC:23841]	-1.130115
GRK5	G protein-coupled receptor kinase 5 [Source:HGNC Symbol;Acc:HGNC:4544]	-1.17268495
GRM1	glutamate metabotropic receptor 1 [Source:HGNC Symbol;Acc:HGNC:4593]	-1.1147814
GRPEL2	GrpE like 2, mitochondrial [Source:HGNC Symbol;Acc:HGNC:21060]	1.2868178
GRRP1	family with sequence similarity 110 member D	1.8366385
GSDMA	gasdermin A [Source:HGNC Symbol;Acc:HGNC:13311]	-1.1433504
GSDMC	gasdermin C [Source:HGNC Symbol;Acc:HGNC:7151]	-1.2073267
GSK3B	glycogen synthase kinase 3 beta [Source:HGNC Symbol;Acc:HGNC:4617]	-1.1495901
GSTA2	glutathione S-transferase alpha 2 [Source:HGNC Symbol;Acc:HGNC:4627]	-1.1239275
GSTM2	glutathione S-transferase mu 2 [Source:HGNC Symbol;Acc:HGNC:4634]	1.1127639
GTDC1	glycosyltransferase like domain containing 1 [Source:HGNC Symbol;Acc:HGNC:20887]	-1.1724172
GTF2H1	general transcription factor IIH subunit 1 [Source:HGNC Symbol;Acc:HGNC:4655]	1.2079952
GTF2IRD2B	GTF2I repeat domain containing 2B [Source:HGNC Symbol;Acc:HGNC:33125]	-1.2213163
GTF3C3	general transcription factor IIIC subunit 3 [Source:HGNC Symbol;Acc:HGNC:4666]	1.1797199
GTF3C4	general transcription factor IIIC subunit 4 [Source:HGNC Symbol;Acc:HGNC:4667]	-1.3418599
GTPBP2	GTP binding protein 2 [Source:HGNC Symbol;Acc:HGNC:4670]	-1.16848465
GTSE1	G2 and S-phase expressed 1 [Source:HGNC Symbol;Acc:HGNC:13698]	-1.1216478
GUCY1A3	guanylate cyclase 1 soluble subunit alpha [Source:HGNC Symbol;Acc:HGNC:4685]	1.1187993
GUCY1B3	guanylate cyclase 1 soluble subunit beta [Source:HGNC Symbol;Acc:HGNC:4687]	1.1112224
GUSBP1	glucuronidase, beta pseudogene 1 [Source:HGNC Symbol;Acc:HGNC:13670]	-1.1376362
GUSBP11	glucuronidase, beta pseudogene 11 [Source:HGNC Symbol;Acc:HGNC:42325]	1.1656725
GYG1	glycogenin 1 [Source:HGNC Symbol;Acc:HGNC:4699]	-1.3225945
GYPE	glycophorin E (MNS blood group) [Source:HGNC Symbol;Acc:HGNC:4705]	-1.1015376
H1F0	H1 histone family member 0 [Source:HGNC Symbol;Acc:HGNC:4714]	1.2161868
H1FNT	H1 histone family member N, testis specific [Source:HGNC Symbol;Acc:HGNC:24893]	-1.216024
H2AFB2	H2A histone family member B2 [Source:HGNC Symbol;Acc:HGNC:18298]	-1.102913
H2AFX	H2A histone family member X [Source:HGNC Symbol;Acc:HGNC:4739]	1.213229
HABP4	hyaluronan binding protein 4 [Source:HGNC Symbol;Acc:HGNC:17062]	1.1008584
HAP1	huntingtin associated protein 1 [Source:HGNC Symbol;Acc:HGNC:4812]	-1.264689
HAPLN3	hyaluronan and proteoglycan link protein 3 [Source:HGNC Symbol;Acc:HGNC:21446]	-1.5585191
HAS1	hyaluronan synthase 1 [Source:HGNC Symbol;Acc:HGNC:4818]	-1.0814188
HAS2-AS1	HAS2 antisense RNA 1 [Source:HGNC Symbol;Acc:HGNC:34340]	1.0910698
HAT1	histone acetyltransferase 1 [Source:HGNC Symbol;Acc:HGNC:4821]	1.58188
HAUS1	HAUS augmin like complex subunit 1 [Source:HGNC Symbol;Acc:HGNC:25174]	1.2777169
HAUS5	HAUS augmin like complex subunit 5 [Source:HGNC Symbol;Acc:HGNC:29130]	-1.068836
HAUS8	HAUS augmin like complex subunit 8 [Source:HGNC Symbol;Acc:HGNC:30532]	1.1316115
HBA2	hemoglobin subunit alpha 2 [Source:HGNC Symbol;Acc:HGNC:4824]	-1.2018528
HBBP1	hemoglobin subunit beta pseudogene 1 [Source:HGNC Symbol;Acc:HGNC:4828]	1.127271
HBD	hemoglobin subunit delta [Source:HGNC Symbol;Acc:HGNC:4829]	-1.115907
HBEGF	heparin binding EGF like growth factor [Source:HGNC Symbol;Acc:HGNC:3059]	-1.9606428
HBS1L	HBS1 like translational GTPase [Source:HGNC Symbol;Acc:HGNC:4834]	1.2680584
HCAR1	hydroxycarboxylic acid receptor 1 [Source:HGNC Symbol;Acc:HGNC:4532]	1.0843768
HCCS	holocytochrome c synthase [Source:HGNC Symbol;Acc:HGNC:4837]	-1.1365395
HCN4	rpolarization activated cyclic nucleotide gated potassium channel 4 [Source:HGNC Symbol;Acc:HGNC:1(	1.1238633
HCP5	HLA complex P5 (non-protein coding) [Source:HGNC Symbol;Acc:HGNC:21659]	-1.2405751

HCST	hematopoietic cell signal transducer [Source:HGNC Symbol;Acc:HGNC:16977]	-1.0997273
HDAC3	histone deacetylase 3 [Source:HGNC Symbol;Acc:HGNC:4854]	1.094488
HDAC4	histone deacetylase 4 [Source:HGNC Symbol;Acc:HGNC:14063]	1.1549897
HDAC6	histone deacetylase 6 [Source:HGNC Symbol;Acc:HGNC:14064]	-1.1276944
HDAC7	histone deacetylase 7 [Source:HGNC Symbol;Acc:HGNC:14067]	1.1660109
HDAC8	histone deacetylase 8 [Source:HGNC Symbol;Acc:HGNC:13315]	-1.1609188
HDGF	hepatoma-derived growth factor [Source:HGNC Symbol;Acc:HGNC:4856]	-1.1828539
HDHD2	haloacid dehalogenase like hydrolase domain containing 2 [Source:HGNC Symbol;Acc:HGNC:25364]	1.3314756
HDX	highly divergent homeobox [Source:HGNC Symbol;Acc:HGNC:26411]	-1.1881844
HEATR1	HEAT repeat containing 1 [Source:HGNC Symbol;Acc:HGNC:25517]	1.3483844
HEATR2	dynein axonemal assembly factor 5	-1.124449
HEATR5A	HEAT repeat containing 5A [Source:HGNC Symbol;Acc:HGNC:20276]	-1.5150114
HEATR8	maestro heat like repeat family member 7	-1.2461494
HEBP1	heme binding protein 1 [Source:HGNC Symbol;Acc:HGNC:17176]	1.1240677
HERC3	HECT and RLD domain containing E3 ubiquitin protein ligase 3 [Source:HGNC Symbol;Acc:HGNC:4876]	1.1079526
HERC5	HECT and RLD domain containing E3 ubiquitin protein ligase 5 [Source:HGNC Symbol;Acc:HGNC:24368]	1.0655811
HERPUD1	ıomocysteine inducible ER protein with ubiquitin like domain 1 [Source:HGNC Symbol;Acc:HGNC:13744	-1.1320776
HES1	hes family bHLH transcription factor 1 [Source:HGNC Symbol;Acc:HGNC:5192]	1.7282095
HES2	hes family bHLH transcription factor 2 [Source:HGNC Symbol;Acc:HGNC:16005]	1.2991556
HES4	hes family bHLH transcription factor 4 [Source:HGNC Symbol;Acc:HGNC:24149]	-1.7153193
HES6	hes family bHLH transcription factor 6 [Source:HGNC Symbol;Acc:HGNC:18254]	-1.2067211
HEXA	hexosaminidase subunit alpha [Source:HGNC Symbol;Acc:HGNC:4878]	-1.1418691
HEY1	hes related family bHLH transcription factor with YRPW motif 1 [Source:HGNC Symbol;Acc:HGNC:4880]	-1.7837278
HEY2	hes related family bHLH transcription factor with YRPW motif 2 [Source:HGNC Symbol;Acc:HGNC:4881]	1.0871742
HEYL	es related family bHLH transcription factor with YRPW motif-like [Source:HGNC Symbol;Acc:HGNC:488	-1.1094779
HFE	hemochromatosis [Source:HGNC Symbol;Acc:HGNC:4886]	-1.1205891
HGD	homogentisate 1,2-dioxygenase [Source:HGNC Symbol;Acc:HGNC:4892]	-1.1496454
HGSNAT	heparan-alpha-glucosaminide N-acetyltransferase [Source:HGNC Symbol;Acc:HGNC:26527]	-1.2157128
HHAT	hedgehog acyltransferase [Source:HGNC Symbol;Acc:HGNC:18270]	1.0805167
HIBCH	3-hydroxyisobutyryl-CoA hydrolase [Source:HGNC Symbol;Acc:HGNC:4908]	1.226886
HINT3	histidine triad nucleotide binding protein 3 [Source:HGNC Symbol;Acc:HGNC:18468]	-1.469548
HIP1	huntingtin interacting protein 1 [Source:HGNC Symbol;Acc:HGNC:4913]	-1.643487
HIP1R	huntingtin interacting protein 1 related [Source:HGNC Symbol;Acc:HGNC:18415]	1.1324588
HIPK2	homeodomain interacting protein kinase 2 [Source:HGNC Symbol;Acc:HGNC:14402]	-1.280938733
HIRA	histone cell cycle regulator [Source:HGNC Symbol;Acc:HGNC:4916]	1.154029
HIST2H2BF	histone cluster 2, H2bf [Source:HGNC Symbol;Acc:HGNC:24700]	1.1890186
HIVEP2	uman immunodeficiency virus type I enhancer binding protein 2 [Source:HGNC Symbol;Acc:HGNC:4921	-4.043127
HIVEP3	ıman immunodeficiency virus type I enhancer binding protein 3 [Source:HGNC Symbol;Acc:HGNC:1356	-1.22298115
HK1	hexokinase 1 [Source:HGNC Symbol;Acc:HGNC:4922]	-1.2101345
HKR1	HKR1, GLI-Kruppel zinc finger family member [Source:HGNC Symbol;Acc:HGNC:4928]	-1.235926
HLA-A	major histocompatibility complex, class I, A [Source:HGNC Symbol;Acc:HGNC:4931]	-1.6363772
HLA-B	major histocompatibility complex, class I, B [Source:HGNC Symbol;Acc:HGNC:4932]	-1.8488251
HLA-C	major histocompatibility complex, class I, C [Source:HGNC Symbol;Acc:HGNC:4933]	-1.84950565
HLA-DMA	major histocompatibility complex, class II, DM alpha [Source:HGNC Symbol;Acc:HGNC:4934]	-1.1644521
HLA-DPB1	major histocompatibility complex, class II, DP beta 1 [Source:HGNC Symbol;Acc:HGNC:4940]	-1.1057085
HLA-DQA1	major histocompatibility complex, class II, DQ alpha 1 [Source:HGNC Symbol;Acc:HGNC:4942]	-1.078359
HLA-DQB1	major histocompatibility complex, class II, DQ beta 1 [Source:HGNC Symbol;Acc:HGNC:4944]	-1.0903803
HLA-DRB6	ıajor histocompatibility complex, class II, DR beta 6 (pseudogene) [Source:HGNC Symbol;Acc:HGNC:495	1.2222303
HLA-E	major histocompatibility complex, class I, E [Source:HGNC Symbol;Acc:HGNC:4962]	-1.5749073
HLA-F	major histocompatibility complex, class I, F [Source:HGNC Symbol;Acc:HGNC:4963]	-1.7106955
HLA-J	major histocompatibility complex, class I, J (pseudogene) [Source:HGNC Symbol;Acc:HGNC:4967]	-1.9360287
HLA-L	major histocompatibility complex, class I, L (pseudogene) [Source:HGNC Symbol;Acc:HGNC:4970]	-1.1717579
HM13	histocompatibility minor 13 [Source:HGNC Symbol;Acc:HGNC:16435]	-1.2656605
HMBOX1	homeobox containing 1 [Source:HGNC Symbol;Acc:HGNC:26137]	-1.1849896
HMBS	hydroxymethylbilane synthase [Source:HGNC Symbol;Acc:HGNC:4982]	1.2667432
HMGCLL1	3-hydroxymethyl-3-methylglutaryl-CoA lyase like 1 [Source:HGNC Symbol;Acc:HGNC:21359]	-1.169939
HMGCR	3-hydroxy-3-methylglutaryl-CoA reductase [Source:HGNC Symbol;Acc:HGNC:5006]	1.4245794
HMGCS2	3-hydroxy-3-methylglutaryl-CoA synthase 2 [Source:HGNC Symbol;Acc:HGNC:5008]	-1.0851665
HMGN3	high mobility group nucleosomal binding domain 3 [Source:HGNC Symbol;Acc:HGNC:12312]	1.2397618
HMOX1	heme oxygenase 1 [Source:HGNC Symbol;Acc:HGNC:5013]	1.2942662
HMOX2	heme oxygenase 2 [Source:HGNC Symbol;Acc:HGNC:5014]	-1.0690899
HN1	hematological and neurological expressed 1 [Source:HGNC Symbol;Acc:HGNC:14569]	1.0836526
HN1L	hematological and neurological expressed 1 like [Source:HGNC Symbol;Acc:HGNC:14137]	-1.258135
HNF4A	hepatocyte nuclear factor 4 alpha [Source:HGNC Symbol;Acc:HGNC:5024]	1.1194208
HNRNPA1	heterogeneous nuclear ribonucleoprotein A1 [Source:HGNC Symbol;Acc:HGNC:5031]	1.3027767
HOMER2	homer scaffolding protein 2 [Source:HGNC Symbol;Acc:HGNC:17513]	1.1064998
HOMEZ	homeobox and leucine zipper encoding [Source:HGNC Symbol;Acc:HGNC:20164]	-1.329111
HOTAIR	HOX transcript antisense RNA [Source:HGNC Symbol;Acc:HGNC:33510]	1.0767479
HOXA10	homeobox A10 [Source:HGNC Symbol;Acc:HGNC:5100]	1.4817383
HOXA11	homeobox A11 [Source:HGNC Symbol;Acc:HGNC:5101]	1.314562
HOXA11-AS1	HOXA11 antisense RNA	1.4127041
HOXA3	homeobox A3 [Source:HGNC Symbol;Acc:HGNC:5104]	-1.2127541
HOXA4	homeobox A4 [Source:HGNC Symbol;Acc:HGNC:5105]	-2.3998737
HOXA7	homeobox A7 [Source:HGNC Symbol;Acc:HGNC:5108]	1.1888288
HOXB2	homeobox B2 [Source:HGNC Symbol;Acc:HGNC:5113]	1.51635425
HOXB5	homeobox B5 [Source:HGNC Symbol;Acc:HGNC:5116]	1.3696412
HOXB7	homeobox B7 [Source:HGNC Symbol;Acc:HGNC:5118]	1.1884856
HOXB8	homeobox B8 [Source:HGNC Symbol;Acc:HGNC:5119]	1.2506728
HOXB9	homeobox B9 [Source:HGNC Symbol;Acc:HGNC:5120]	-1.6384615
HOXD11	homeobox D11 [Source:HGNC Symbol;Acc:HGNC:5134]	1.1339025
HOXD3	homeobox D3 [Source:HGNC Symbol;Acc:HGNC:5137]	1.1087532
HOXD8	homeobox D8 [Source:HGNC Symbol;Acc:HGNC:5139]	1.5310539
HP	haptoglobin [Source:HGNC Symbol;Acc:HGNC:5141]	-1.24853
HPS3	HPS3, biogenesis of lysosomal organelles complex 2 subunit 1 [Source:HGNC Symbol;Acc:HGNC:15597]	1.3031766
HPS4	HPS4, biogenesis of lysosomal organelles complex 3 subunit 2 [Source:HGNC Symbol;Acc:HGNC:15844]	-1.1146306
HPS6	HPS6, biogenesis of lysosomal organelles complex 2 subunit 3 [Source:HGNC Symbol;Acc:HGNC:18817]	1.1038908

HRAS	HRas proto-oncogene, GTPase [Source:HGNC Symbol;Acc:HGNC:5173]	-1.1408103
HRASLS5	HRAS like suppressor family member 5 [Source:HGNC Symbol;Acc:HGNC:24978]	-1.4020449
HRCT1	histidine rich carboxyl terminus 1 [Source:HGNC Symbol;Acc:HGNC:33872]	1.9456505
HRH1	histamine receptor H1 [Source:HGNC Symbol;Acc:HGNC:5182]	-1.4982616
HRH3	histamine receptor H3 [Source:HGNC Symbol;Acc:HGNC:5184]	1.0619979
HS2ST1	heparan sulfate 2-O-sulfotransferase 1 [Source:HGNC Symbol;Acc:HGNC:5193]	1.3886265
HS3ST1	heparan sulfate-glucosamine 3-sulfotransferase 1 [Source:HGNC Symbol;Acc:HGNC:5194]	1.4251945
HS3ST5	heparan sulfate-glucosamine 3-sulfotransferase 5 [Source:HGNC Symbol;Acc:HGNC:19419]	1.13301505
HS6ST1	heparan sulfate 6-O-sulfotransferase 1 [Source:HGNC Symbol;Acc:HGNC:5201]	-1.2474322
HS6ST3	heparan sulfate 6-O-sulfotransferase 3 [Source:HGNC Symbol;Acc:HGNC:19134]	1.1348127
HSD17B2	hydroxysteroid 17-beta dehydrogenase 2 [Source:HGNC Symbol;Acc:HGNC:5211]	1.565866
HSD17B4	hydroxysteroid 17-beta dehydrogenase 4 [Source:HGNC Symbol;Acc:HGNC:5213]	1.1892312
HSD17B7	hydroxysteroid 17-beta dehydrogenase 7 [Source:HGNC Symbol;Acc:HGNC:5215]	1.12424025
HSDL1	hydroxysteroid dehydrogenase like 1 [Source:HGNC Symbol;Acc:HGNC:16475]	1.097019
HSF2	heat shock transcription factor 2 [Source:HGNC Symbol;Acc:HGNC:5225]	1.418836
HSFY1P1	heat shock transcription factor, Y-linked 1 pseudogene 1 [Source:HGNC Symbol;Acc:HGNC:1846]	1.0765933
HSH2D	hematopoietic SH2 domain containing [Source:HGNC Symbol;Acc:HGNC:24920]	-1.1177905
HSPA1A	heat shock protein family A (Hsp70) member 1A [Source:HGNC Symbol;Acc:HGNC:5232]	-1.1816055
HSPA2	heat shock protein family A (Hsp70) member 2 [Source:HGNC Symbol;Acc:HGNC:5235]	-1.4627583
HSPA4	heat shock protein family A (Hsp70) member 4 [Source:HGNC Symbol;Acc:HGNC:5237]	1.25257205
HSPB7	heat shock protein family B (small) member 7 [Source:HGNC Symbol;Acc:HGNC:5249]	1.0952973
HSPBAP1	HSPB1 associated protein 1 [Source:HGNC Symbol;Acc:HGNC:16389]	1.1326001
HTN1	histatin 1 [Source:HGNC Symbol;Acc:HGNC:5283]	1.1245089
HTN3	histatin 3 [Source:HGNC Symbol;Acc:HGNC:5284]	1.1108384
HTR1D	5-hydroxytryptamine receptor 1D [Source:HGNC Symbol;Acc:HGNC:5289]	1.1412073
HTR1F	5-hydroxytryptamine receptor 1F [Source:HGNC Symbol;Acc:HGNC:5292]	1.1200968
HTR2C	5-hydroxytryptamine receptor 2C [Source:HGNC Symbol;Acc:HGNC:5295]	1.0986276
HTR3A	5-hydroxytryptamine receptor 3A [Source:HGNC Symbol;Acc:HGNC:5297]	-1.1769689
HTR4	5-hydroxytryptamine receptor 4 [Source:HGNC Symbol;Acc:HGNC:5299]	1.0817528
HTR6	5-hydroxytryptamine receptor 6 [Source:HGNC Symbol;Acc:HGNC:5301]	-1.1426796
HTR7	5-hydroxytryptamine receptor 7 [Source:HGNC Symbol;Acc:HGNC:5302]	1.3485706
HTR7P1	5-hydroxytryptamine receptor 7 pseudogene 1 [Source:HGNC Symbol;Acc:HGNC:30411]	1.3741966
HTRA2	HtrA serine peptidase 2 [Source:HGNC Symbol;Acc:HGNC:14348]	-1.1839318
HTT	huntingtin [Source:HGNC Symbol;Acc:HGNC:4851]	-1.1539761
HULC	hepatocellular carcinoma up-regulated long non-coding RNA [Source:HGNC Symbol;Acc:HGNC:34232]	1.095667
HUS1B	HUS1 checkpoint clamp component B [Source:HGNC Symbol;Acc:HGNC:16485]	-1.0555236
HUWE1	T, UBA and WWE domain containing 1, E3 ubiquitin protein ligase [Source:HGNC Symbol;Acc:HGNC:30	-1.4209539
HYAL1	hyaluronoglucosaminidase 1 [Source:HGNC Symbol;Acc:HGNC:5320]	-1.1860045
HYAL3	hyaluronoglucosaminidase 3 [Source:HGNC Symbol;Acc:HGNC:5322]	-1.2404289
ICAM1	intercellular adhesion molecule 1 [Source:HGNC Symbol;Acc:HGNC:5344]	-54.208492
ICAM4	ercellular adhesion molecule 4 (Landsteiner-Wiener blood group) [Source:HGNC Symbol;Acc:HGNC:53	-6.44848
ICAM5	intercellular adhesion molecule 5 [Source:HGNC Symbol;Acc:HGNC:5348]	-1.2500415
ICOS	inducible T-cell costimulator [Source:HGNC Symbol;Acc:HGNC:5351]	-1.1336795
ICOSLG	inducible T-cell costimulator ligand [Source:HGNC Symbol;Acc:HGNC:17087]	-5.4298136
ICT1	mitochondrial ribosomal protein L58	-1.1742265
ID1	inhibitor of DNA binding 1, HLH protein [Source:HGNC Symbol;Acc:HGNC:5360]	2.9173017
ID2	inhibitor of DNA binding 2, HLH protein [Source:HGNC Symbol;Acc:HGNC:5361]	1.92504425
IDAS	multiciliate differentiation and DNA synthesis associated cell cycle protein	1.1195458
IDE	insulin degrading enzyme [Source:HGNC Symbol;Acc:HGNC:5381]	1.2076976
IDH3A	isocitrate dehydrogenase 3 (NAD(+)) alpha [Source:HGNC Symbol;Acc:HGNC:5384]	1.365312
IDI1	isopentenyl-diphosphate delta isomerase 1 [Source:HGNC Symbol;Acc:HGNC:5387]	1.5269727
IER2	immediate early response 2 [Source:HGNC Symbol;Acc:HGNC:28871]	1.1591237
IER3	immediate early response 3 [Source:HGNC Symbol;Acc:HGNC:5392]	-1.3029556
IER3IP1	immediate early response 3 interacting protein 1 [Source:HGNC Symbol;Acc:HGNC:18550]	1.3192735
IER5	immediate early response 5 [Source:HGNC Symbol;Acc:HGNC:5393]	-2.3785453
IER5L	immediate early response 5 like [Source:HGNC Symbol;Acc:HGNC:23679]	1.5421685
IFFO1	intermediate filament family orphan 1 [Source:HGNC Symbol;Acc:HGNC:24970]	1.3684595
IFFO2	intermediate filament family orphan 2 [Source:HGNC Symbol;Acc:HGNC:27006]	1.1812522
IFI16	interferon gamma inducible protein 16 [Source:HGNC Symbol;Acc:HGNC:5395]	1.450654
IFI30	IFI30, lysosomal thiol reductase [Source:HGNC Symbol;Acc:HGNC:5398]	-1.8574595
IFIH1	interferon induced with helicase C domain 1 [Source:HGNC Symbol;Acc:HGNC:18873]	-1.9001925
IFITM1	interferon induced transmembrane protein 1 [Source:HGNC Symbol;Acc:HGNC:5412]	-1.1605284
IFLTD1	lamin tail domain containing 1	1.0886848
IFNA14	interferon alpha 14 [Source:HGNC Symbol;Acc:HGNC:5420]	1.1493437
IFNAR1	interferon alpha and beta receptor subunit 1 [Source:HGNC Symbol;Acc:HGNC:5432]	-1.2214895
IFNAR2	interferon alpha and beta receptor subunit 2 [Source:HGNC Symbol;Acc:HGNC:5433]	-1.488919833
IFNG	interferon gamma [Source:HGNC Symbol;Acc:HGNC:5438]	1.054601
IFNGR1	interferon gamma receptor 1 [Source:HGNC Symbol;Acc:HGNC:5439]	-1.8999877
IFNGR2	interferon gamma receptor 2 (interferon gamma transducer 1) [Source:HGNC Symbol;Acc:HGNC:5440]	-2.0907316
IFT122	intraflagellar transport 122 [Source:HGNC Symbol;Acc:HGNC:13556]	-1.1377552
IFT20	intraflagellar transport 20 [Source:HGNC Symbol;Acc:HGNC:30989]	-1.2719423
IFT74	intraflagellar transport 74 [Source:HGNC Symbol;Acc:HGNC:21424]	1.2885128
IGBP1	immunoglobulin (CD79A) binding protein 1 [Source:HGNC Symbol;Acc:HGNC:5461]	-1.1667752
IGF2AS	IGF2 antisense RNA	-1.193187
IGF2BP3	insulin like growth factor 2 mRNA binding protein 3 [Source:HGNC Symbol;Acc:HGNC:28868]	1.7994442
IGFBP1	insulin like growth factor binding protein 1 [Source:HGNC Symbol;Acc:HGNC:5469]	1.6492078
IGFBP4	insulin like growth factor binding protein 4 [Source:HGNC Symbol;Acc:HGNC:5473]	1.2349166
IGFL2	IGF like family member 2 [Source:HGNC Symbol;Acc:HGNC:32929]	-1.0987693
IGFL3	IGF like family member 3 [Source:HGNC Symbol;Acc:HGNC:32930]	1.1372129
IGFN1	nmunoglobulin-like and fibronectin type III domain containing 1 [Source:HGNC Symbol;Acc:HGNC:2460	-1.1203761
IGJ	joining chain of multimeric IgA and IgM	-1.1395674
IGSF1	immunoglobulin superfamily member 1 [Source:HGNC Symbol;Acc:HGNC:5948]	-1.1130397
IGSF21	immunoglobin superfamily member 21 [Source:HGNC Symbol;Acc:HGNC:28246]	-1.1412876
IGSF6	immunoglobulin superfamily member 6 [Source:HGNC Symbol;Acc:HGNC:5953]	1.0536579
IGSF9B	immunoglobulin superfamily member 9B [Source:HGNC Symbol;Acc:HGNC:32326]	-1.3680544
IHH	indian hedgehog [Source:HGNC Symbol;Acc:HGNC:5956]	1.0591375

IKBKAP	a light polypeptide gene enhancer in B-cells, kinase complex-associated protein [Source:HGNC Symbol;	1.1545026
IKBKE	or of kappa light polypeptide gene enhancer in B-cells, kinase epsilon [Source:HGNC Symbol;Acc:HGNC:	-1.9291891
IKZF2	IKAROS family zinc finger 2 [Source:HGNC Symbol;Acc:HGNC:13177]	1.1250074
IKZF3	IKAROS family zinc finger 3 [Source:HGNC Symbol;Acc:HGNC:13178]	-1.0699296
IKZF4	IKAROS family zinc finger 4 [Source:HGNC Symbol;Acc:HGNC:13179]	-1.277173
IL10	interleukin 10 [Source:HGNC Symbol;Acc:HGNC:5962]	-1.0685151
IL10RA	interleukin 10 receptor subunit alpha [Source:HGNC Symbol;Acc:HGNC:5964]	-1.4823235
IL10RB	interleukin 10 receptor subunit beta [Source:HGNC Symbol;Acc:HGNC:5965]	-1.1694837
IL11	interleukin 11 [Source:HGNC Symbol;Acc:HGNC:5966]	1.3062071
IL12A	interleukin 12A [Source:HGNC Symbol;Acc:HGNC:5969]	1.119767
IL13	interleukin 13 [Source:HGNC Symbol;Acc:HGNC:5973]	1.0838113
IL13RA1	interleukin 13 receptor subunit alpha 1 [Source:HGNC Symbol;Acc:HGNC:5974]	-1.1157675
IL13RA2	interleukin 13 receptor subunit alpha 2 [Source:HGNC Symbol;Acc:HGNC:5975]	1.1172427
IL15	interleukin 15 [Source:HGNC Symbol;Acc:HGNC:5977]	-4.2042212
IL15RA	interleukin 15 receptor subunit alpha [Source:HGNC Symbol;Acc:HGNC:5978]	-1.69626815
IL16	interleukin 16 [Source:HGNC Symbol;Acc:HGNC:5980]	1.0886935
IL17A	interleukin 17A [Source:HGNC Symbol;Acc:HGNC:5981]	-1.1687678
IL17RB	interleukin 17 receptor B [Source:HGNC Symbol;Acc:HGNC:18015]	1.0950464
IL17RD	interleukin 17 receptor D [Source:HGNC Symbol;Acc:HGNC:17616]	-1.1061695
IL18BP	interleukin 18 binding protein [Source:HGNC Symbol;Acc:HGNC:5987]	-1.2322948
IL18R1	interleukin 18 receptor 1 [Source:HGNC Symbol;Acc:HGNC:5988]	-1.543199033
IL18RAP	interleukin 18 receptor accessory protein [Source:HGNC Symbol;Acc:HGNC:5989]	-1.32891765
IL19	interleukin 19 [Source:HGNC Symbol;Acc:HGNC:5990]	-1.1487836
IL1A	interleukin 1 alpha [Source:HGNC Symbol;Acc:HGNC:5991]	-1.6709274
IL1B	interleukin 1 beta [Source:HGNC Symbol;Acc:HGNC:5992]	-1.8520757
IL1F10	interleukin 1 family member 10 (theta) [Source:HGNC Symbol;Acc:HGNC:15552]	-1.1481426
IL1R2	interleukin 1 receptor type 2 [Source:HGNC Symbol;Acc:HGNC:5994]	1.2024618
IL1RL2	interleukin 1 receptor like 2 [Source:HGNC Symbol;Acc:HGNC:5999]	-1.0876387
IL1RN	interleukin 1 receptor antagonist [Source:HGNC Symbol;Acc:HGNC:6000]	-1.0839552
IL20	interleukin 20 [Source:HGNC Symbol;Acc:HGNC:6002]	1.064652
IL20RA	interleukin 20 receptor subunit alpha [Source:HGNC Symbol;Acc:HGNC:6003]	1.0829166
IL20RB	interleukin 20 receptor subunit beta [Source:HGNC Symbol;Acc:HGNC:6004]	-1.3602723
IL21R	interleukin 21 receptor [Source:HGNC Symbol;Acc:HGNC:6006]	-1.1329529
IL22RA2	interleukin 22 receptor subunit alpha 2 [Source:HGNC Symbol;Acc:HGNC:14901]	1.2649815
IL23A	interleukin 23 subunit alpha [Source:HGNC Symbol;Acc:HGNC:15488]	-2.6102004
IL27RA	interleukin 27 receptor subunit alpha [Source:HGNC Symbol;Acc:HGNC:17290]	-1.3609478
IL28A	interferon lambda 2	-1.1997145
IL3	interleukin 3 [Source:HGNC Symbol;Acc:HGNC:6011]	1.0590298
IL32	interleukin 32 [Source:HGNC Symbol;Acc:HGNC:16830]	-2.7948629
IL34	interleukin 34 [Source:HGNC Symbol;Acc:HGNC:28529]	-1.2410488
IL36RN	interleukin 36 receptor antagonist [Source:HGNC Symbol;Acc:HGNC:15561]	1.1045929
IL37	interleukin 37 [Source:HGNC Symbol;Acc:HGNC:15563]	-1.2863138
IL3RA	interleukin 3 receptor subunit alpha [Source:HGNC Symbol;Acc:HGNC:6012]	-2.2783055
IL4I1	interleukin 4 induced 1 [Source:HGNC Symbol;Acc:HGNC:19094]	-6.7667794
IL4R	interleukin 4 receptor [Source:HGNC Symbol;Acc:HGNC:6015]	-1.440208767
IL6	interleukin 6 [Source:HGNC Symbol;Acc:HGNC:6018]	-1.672027
IL7R	interleukin 7 receptor [Source:HGNC Symbol;Acc:HGNC:6024]	-2.9823403
IL8	C-X-C motif chemokine ligand 8	-8.16275255
ILF2	interleukin enhancer binding factor 2 [Source:HGNC Symbol;Acc:HGNC:6037]	1.1290766
ILF3	interleukin enhancer binding factor 3 [Source:HGNC Symbol;Acc:HGNC:6038]	-1.1732329
ILK	integrin linked kinase [Source:HGNC Symbol;Acc:HGNC:6040]	-1.112325
ILKAP	ILK associated serine/threonine phosphatase [Source:HGNC Symbol;Acc:HGNC:15566]	-1.0899183
IMMT	inner membrane mitochondrial protein [Source:HGNC Symbol;Acc:HGNC:6047]	-1.2021757
IMP3	IMP3, U3 small nucleolar ribonucleoprotein [Source:HGNC Symbol;Acc:HGNC:14497]	1.1432078
IMPAD1	inositol monophosphatase domain containing 1 [Source:HGNC Symbol;Acc:HGNC:26019]	1.3196304
IMPDH2	inosine monophosphate dehydrogenase 2 [Source:HGNC Symbol;Acc:HGNC:6053]	1.0966448
IMPG2	interphotoreceptor matrix proteoglycan 2 [Source:HGNC Symbol;Acc:HGNC:18362]	-1.1148427
INADL	PATJ, crumbs cell polarity complex component	-1.338655
INE1	inactivation escape 1 (non-protein coding) [Source:HGNC Symbol;Acc:HGNC:6060]	-1.2958481
INF2	inverted formin, FH2 and WH2 domain containing [Source:HGNC Symbol;Acc:HGNC:23791]	-1.679498
ING5	inhibitor of growth family member 5 [Source:HGNC Symbol;Acc:HGNC:19421]	1.1050963
INHBB	inhibin beta B subunit [Source:HGNC Symbol;Acc:HGNC:6067]	1.1901286
INMT	indolethylamine N-methyltransferase [Source:HGNC Symbol;Acc:HGNC:6069]	-1.0938246
INO80B	INO80 complex subunit B [Source:HGNC Symbol;Acc:HGNC:13324]	1.2384149
INO80D	INO80 complex subunit D [Source:HGNC Symbol;Acc:HGNC:25997]	-1.1614156
INPP1	inositol polyphosphate-1-phosphatase [Source:HGNC Symbol;Acc:HGNC:6071]	1.3880361
INPP4A	inositol polyphosphate-4-phosphatase type I A [Source:HGNC Symbol;Acc:HGNC:6074]	-1.1496724
INPP5A	inositol polyphosphate-5-phosphatase A [Source:HGNC Symbol;Acc:HGNC:6076]	-1.24781105
INPP5D	inositol polyphosphate-5-phosphatase D [Source:HGNC Symbol;Acc:HGNC:6079]	1.3394874
INPP5E	inositol polyphosphate-5-phosphatase E [Source:HGNC Symbol;Acc:HGNC:21474]	-1.1468072
INPPL1	inositol polyphosphate phosphatase like 1 [Source:HGNC Symbol;Acc:HGNC:6080]	1.1466528
INSIG2	insulin induced gene 2 [Source:HGNC Symbol;Acc:HGNC:20452]	-1.1946335
INSL5	insulin like 5 [Source:HGNC Symbol;Acc:HGNC:6088]	1.1545088
INSM2	INSM transcriptional repressor 2 [Source:HGNC Symbol;Acc:HGNC:17539]	-1.1003453
INTS1	integrator complex subunit 1 [Source:HGNC Symbol;Acc:HGNC:24555]	1.1585999
INTS10	integrator complex subunit 10 [Source:HGNC Symbol;Acc:HGNC:25548]	1.2543128
INTS8	integrator complex subunit 8 [Source:HGNC Symbol;Acc:HGNC:26048]	1.2659352
IPO11	importin 11 [Source:HGNC Symbol;Acc:HGNC:20628]	1.166967
IPO7	importin 7 [Source:HGNC Symbol;Acc:HGNC:9852]	1.35156555
IPO8	importin 8 [Source:HGNC Symbol;Acc:HGNC:9853]	1.3170022
IPPK	inositol-pentakisphosphate 2-kinase [Source:HGNC Symbol;Acc:HGNC:14645]	-1.2809155
IQCA1	IQ motif containing with AAA domain 1 [Source:HGNC Symbol;Acc:HGNC:26195]	-1.1636845
IQCE	IQ motif containing E [Source:HGNC Symbol;Acc:HGNC:29171]	1.1891303
IQCF1	IQ motif containing F1 [Source:HGNC Symbol;Acc:HGNC:28607]	-1.1351235
IQCK	IQ motif containing K [Source:HGNC Symbol;Acc:HGNC:28556]	-1.1266048
IQGAP3	IQ motif containing GTPase activating protein 3 [Source:HGNC Symbol;Acc:HGNC:20669]	-1.1946466

IQSEC1	IQ motif and Sec7 domain 1 [Source:HGNC Symbol;Acc:HGNC:29112]	1.1182463
IRAK2	interleukin 1 receptor associated kinase 2 [Source:HGNC Symbol;Acc:HGNC:6113]	-6.068329
IRF1	interferon regulatory factor 1 [Source:HGNC Symbol;Acc:HGNC:6116]	-4.574583
IRF2	interferon regulatory factor 2 [Source:HGNC Symbol;Acc:HGNC:6117]	-1.2343482
IRF3	interferon regulatory factor 3 [Source:HGNC Symbol;Acc:HGNC:6118]	1.1684548
IRF5	interferon regulatory factor 5 [Source:HGNC Symbol;Acc:HGNC:6120]	-1.6055096
IRF6	interferon regulatory factor 6 [Source:HGNC Symbol;Acc:HGNC:6121]	-1.4407084
IRF7	interferon regulatory factor 7 [Source:HGNC Symbol;Acc:HGNC:6122]	-1.3743957
IRF9	interferon regulatory factor 9 [Source:HGNC Symbol;Acc:HGNC:6131]	-1.4288356
IRS1	insulin receptor substrate 1 [Source:HGNC Symbol;Acc:HGNC:6125]	1.1737791
IRS2	insulin receptor substrate 2 [Source:HGNC Symbol;Acc:HGNC:6126]	-1.2032815
ISCA1	iron-sulfur cluster assembly 1 [Source:HGNC Symbol;Acc:HGNC:28660]	-1.1577586
ISCU	iron-sulfur cluster assembly enzyme [Source:HGNC Symbol;Acc:HGNC:29882]	1.1609955
ISG20	interferon stimulated exonuclease gene 20 [Source:HGNC Symbol;Acc:HGNC:6130]	-2.3666964
ISM1	isthmin 1, angiogenesis inhibitor [Source:HGNC Symbol;Acc:HGNC:16213]	-1.0966566
ITGA5	integrin subunit alpha 5 [Source:HGNC Symbol;Acc:HGNC:6141]	1.30258
ITGAL	integrin subunit alpha L [Source:HGNC Symbol;Acc:HGNC:6148]	1.1334461
ITGB1BP1	integrin subunit beta 1 binding protein 1 [Source:HGNC Symbol;Acc:HGNC:23927]	1.1433567
ITGB3BP	integrin subunit beta 3 binding protein [Source:HGNC Symbol;Acc:HGNC:6157]	1.163818
ITGB7	integrin subunit beta 7 [Source:HGNC Symbol;Acc:HGNC:6162]	-1.2926884
ITIH1	inter-alpha-trypsin inhibitor heavy chain 1 [Source:HGNC Symbol;Acc:HGNC:6166]	-1.1413702
ITIH5	inter-alpha-trypsin inhibitor heavy chain family member 5 [Source:HGNC Symbol;Acc:HGNC:21449]	1.1360995
ITM2B	integral membrane protein 2B [Source:HGNC Symbol;Acc:HGNC:6174]	-1.1317462
ITPKC	inositol-trisphosphate 3-kinase C [Source:HGNC Symbol;Acc:HGNC:14897]	-1.2544945
IVNS1ABP	influenza virus NS1A binding protein [Source:HGNC Symbol;Acc:HGNC:16951]	1.28182125
IWS1	IWS1, SUPT6H interacting protein [Source:HGNC Symbol;Acc:HGNC:25467]	1.120061
JAG1	jagged 1 [Source:HGNC Symbol;Acc:HGNC:6188]	-2.8004541
JAG2	jagged 2 [Source:HGNC Symbol;Acc:HGNC:6189]	1.2217559
JAK3	Janus kinase 3 [Source:HGNC Symbol;Acc:HGNC:6193]	-2.9648972
JAM2	junctional adhesion molecule 2 [Source:HGNC Symbol;Acc:HGNC:14686]	-2.370465267
JAZF1	JAZF zinc finger 1 [Source:HGNC Symbol;Acc:HGNC:28917]	-1.2585773
JDP2	Jun dimerization protein 2 [Source:HGNC Symbol;Acc:HGNC:17546]	-1.3221147
JKAMP	JNK1/MAPK8-associated membrane protein [Source:HGNC Symbol;Acc:HGNC:20184]	1.2957004
JMJD6	arginine demethylase and lysine hydroxylase [Source:HGNC Symbol;Acc:HGNC:19355]	-1.2402571
JOSD1	Josephin domain containing 1 [Source:HGNC Symbol;Acc:HGNC:28953]	1.0955805
JPH4	junctophilin 4 [Source:HGNC Symbol;Acc:HGNC:20156]	1.1694688
JUNB	JunB proto-oncogene, AP-1 transcription factor subunit [Source:HGNC Symbol;Acc:HGNC:6205]	-2.7148168
KAL1	anosmin 1	1.2410437
KANK2	KN motif and ankyrin repeat domains 2 [Source:HGNC Symbol;Acc:HGNC:29300]	1.3396652
KANK3	KN motif and ankyrin repeat domains 3 [Source:HGNC Symbol;Acc:HGNC:24796]	-1.1484523
KAT2A	lysine acetyltransferase 2A [Source:HGNC Symbol;Acc:HGNC:4201]	1.4546719
KAT5	lysine acetyltransferase 5 [Source:HGNC Symbol;Acc:HGNC:5275]	-1.2389823
KAT6B	lysine acetyltransferase 6B [Source:HGNC Symbol;Acc:HGNC:17582]	-1.0665951
KAZALD1	Kazal type serine peptidase inhibitor domain 1 [Source:HGNC Symbol;Acc:HGNC:25460]	1.3934486
KAZN	kazrin, periplakin interacting protein [Source:HGNC Symbol;Acc:HGNC:29173]	-1.1227167
KBTBD10	kelch like family member 41	1.1044997
KCND3	potassium voltage-gated channel subfamily D member 3 [Source:HGNC Symbol;Acc:HGNC:6239]	1.069064
KCNE3	otassium voltage-gated channel subfamily E regulatory subunit 3 [Source:HGNC Symbol;Acc:HGNC:624	-1.1952144
KCNH7	potassium voltage-gated channel subfamily H member 7 [Source:HGNC Symbol;Acc:HGNC:18863]	1.0856394
KCNJ12	potassium voltage-gated channel subfamily J member 12 [Source:HGNC Symbol;Acc:HGNC:6258]	1.3526348
KCNJ15	potassium voltage-gated channel subfamily J member 15 [Source:HGNC Symbol;Acc:HGNC:6261]	1.1332278
KCNK1	potassium two pore domain channel subfamily K member 1 [Source:HGNC Symbol;Acc:HGNC:6272]	-1.6628482
KCNK4	potassium two pore domain channel subfamily K member 4 [Source:HGNC Symbol;Acc:HGNC:6279]	-1.1365039
KCNK5	potassium two pore domain channel subfamily K member 5 [Source:HGNC Symbol;Acc:HGNC:6280]	-1.2193043
KCNK6	potassium two pore domain channel subfamily K member 6 [Source:HGNC Symbol;Acc:HGNC:6281]	1.2970812
KCNK7	potassium two pore domain channel subfamily K member 7 [Source:HGNC Symbol;Acc:HGNC:6282]	-1.2175599
KCNMB2	ium calcium-activated channel subfamily M regulatory beta subunit 2 [Source:HGNC Symbol;Acc:HGNC	1.1031604
KCNMB4	ium calcium-activated channel subfamily M regulatory beta subunit 4 [Source:HGNC Symbol;Acc:HGNC	1.2300395
KCNN2	potassium calcium-activated channel subfamily N member 2 [Source:HGNC Symbol;Acc:HGNC:6291]	-2.946887
KCNQ1	potassium voltage-gated channel subfamily Q member 1 [Source:HGNC Symbol;Acc:HGNC:6294]	-1.1221502
KCNQ2	potassium voltage-gated channel subfamily Q member 2 [Source:HGNC Symbol;Acc:HGNC:6296]	-1.14196305
KCNS3	otassium voltage-gated channel modifier subfamily S member 3 [Source:HGNC Symbol;Acc:HGNC:6302	-1.6835319
KCP	kielin/chordin-like protein [Source:HGNC Symbol;Acc:HGNC:17585]	-1.1736016
KCTD1	potassium channel tetramerization domain containing 1 [Source:HGNC Symbol;Acc:HGNC:18249]	1.3043356
KCTD11	potassium channel tetramerization domain containing 11 [Source:HGNC Symbol;Acc:HGNC:21302]	-1.7550062
KCTD12	potassium channel tetramerization domain containing 12 [Source:HGNC Symbol;Acc:HGNC:14678]	2.00215525
KCTD13	potassium channel tetramerization domain containing 13 [Source:HGNC Symbol;Acc:HGNC:22234]	-1.1941956
KCTD14	potassium channel tetramerization domain containing 14 [Source:HGNC Symbol;Acc:HGNC:23295]	1.1203846
KCTD15	potassium channel tetramerization domain containing 15 [Source:HGNC Symbol;Acc:HGNC:23297]	1.1134436
KCTD21	potassium channel tetramerization domain containing 21 [Source:HGNC Symbol;Acc:HGNC:27452]	-1.1448433
KCTD3	potassium channel tetramerization domain containing 3 [Source:HGNC Symbol;Acc:HGNC:21305]	1.2762947
KCTD5	potassium channel tetramerization domain containing 5 [Source:HGNC Symbol;Acc:HGNC:21423]	-1.1867813
KDELC1	KDEL motif containing 1 [Source:HGNC Symbol;Acc:HGNC:19350]	-1.2030149
KDELR3	KDEL endoplasmic reticulum protein retention receptor 3 [Source:HGNC Symbol;Acc:HGNC:6306]	-1.3354626
KDM1B	lysine demethylase 1B [Source:HGNC Symbol;Acc:HGNC:21577]	-1.0974213
KDM2A	lysine demethylase 2A [Source:HGNC Symbol;Acc:HGNC:13606]	-1.5063708
KDM3A	lysine demethylase 3A [Source:HGNC Symbol;Acc:HGNC:20815]	1.2498034
KDM4A	lysine demethylase 4A [Source:HGNC Symbol;Acc:HGNC:22978]	-1.1761543
KDM4D	lysine demethylase 4D [Source:HGNC Symbol;Acc:HGNC:25498]	-1.2728119
KDM6B	lysine demethylase 6B [Source:HGNC Symbol;Acc:HGNC:29012]	-1.2253329
KDSR	3-ketodihydrosphingosine reductase [Source:HGNC Symbol;Acc:HGNC:4021]	1.1298662
KEL	Kell blood group, metallo-endopeptidase [Source:HGNC Symbol;Acc:HGNC:6308]	1.1421485
KHSRP	KH-type splicing regulatory protein [Source:HGNC Symbol;Acc:HGNC:6316]	1.2597895
KIAA0090	ER membrane protein complex subunit 1	1.0713794
KIAA0174	IST1, ESCRT-III associated factor	-1.2136495
KIAA0196	KIAA0196 [Source:HGNC Symbol;Acc:HGNC:28984]	1.2771231

KIAA0247	sushi domain containing 6	-1.3338534
KIAA0284	centrosomal protein 170B	1.1373115
KIAA0317	apoptosis resistant E3 ubiquitin protein ligase 1	-1.2232885
KIAA0391	KIAA0391 [Source:HGNC Symbol;Acc:HGNC:19958]	-1.1419615
KIAA0415	adaptor related protein complex 5 zeta 1 subunit	-1.4796715
KIAA0528	C2 calcium dependent domain containing 5	1.4897679
KIAA0664L3	clustered mitochondria homolog pseudogene 3	-1.2562492
KIAA0753	KIAA0753 [Source:HGNC Symbol;Acc:HGNC:29110]	1.2224723
KIAA0907	KIAA0907 [Source:HGNC Symbol;Acc:HGNC:29145]	-1.1477985
KIAA1147	KIAA1147 [Source:HGNC Symbol;Acc:HGNC:29472]	-1.31527265
KIAA1191	KIAA1191 [Source:HGNC Symbol;Acc:HGNC:29209]	-1.1766967
KIAA1217	KIAA1217 [Source:HGNC Symbol;Acc:HGNC:25428]	-1.21932455
KIAA1239	NACHT and WD repeat domain containing 2	1.1688716
KIAA1310	KAT8 regulatory NSL complex subunit 3	-1.2986103
KIAA1429	KIAA1429 [Source:HGNC Symbol;Acc:HGNC:24500]	1.3513559
KIAA1430	cilia and flagella associated protein 97	1.498723
KIAA1432	RIC1 homolog, RAB6A GEF complex partner 1	1.2558789
KIAA1467	family with sequence similarity 234 member B	1.2544901
KIAA1522	KIAA1522 [Source:HGNC Symbol;Acc:HGNC:29301]	-1.3404709
KIAA1539	family with sequence similarity 214 member B	1.3029575
KIAA1609	TBC/LysM-associated domain containing 1	-1.70323875
KIAA1644	KIAA1644 [Source:HGNC Symbol;Acc:HGNC:29335]	-1.2054121
KIAA1656	KIAA1656 protein	1.1104379
KIAA1671	KIAA1671 [Source:HGNC Symbol;Acc:HGNC:29345]	-1.108342
KIAA1683	KIAA1683 [Source:HGNC Symbol;Acc:HGNC:29350]	-1.1410635
KIAA1715	lunapark, ER junction formation factor	1.1323284
KIAA1804	mitogen-activated protein kinase kinase kinase 21	-1.2970525
KIAA1949	protein phosphatase 1 regulatory subunit 18	-1.3295424
KIF13A	kinesin family member 13A [Source:HGNC Symbol;Acc:HGNC:14566]	-1.3354458
KIF17	kinesin family member 17 [Source:HGNC Symbol;Acc:HGNC:19167]	-1.142011
KIF19	kinesin family member 19 [Source:HGNC Symbol;Acc:HGNC:26735]	1.2089506
KIF1B	kinesin family member 1B [Source:HGNC Symbol;Acc:HGNC:16636]	-1.0699205
KIF21A	kinesin family member 21A [Source:HGNC Symbol;Acc:HGNC:19349]	1.0866286
KIF21B	kinesin family member 21B [Source:HGNC Symbol;Acc:HGNC:29442]	-1.3706878
KIF26A	kinesin family member 26A [Source:HGNC Symbol;Acc:HGNC:20226]	-1.393289
KIF2A	kinesin family member 2A [Source:HGNC Symbol;Acc:HGNC:6318]	1.2320776
KIF6	kinesin family member 6 [Source:HGNC Symbol;Acc:HGNC:21202]	1.1285
KIFC2	kinesin family member C2 [Source:HGNC Symbol;Acc:HGNC:29530]	1.0632762
KIRREL2	kin of IRRE like 2 (Drosophila) [Source:HGNC Symbol;Acc:HGNC:18816]	-1.1878211
KIRREL3	kin of IRRE like 3 (Drosophila) [Source:HGNC Symbol;Acc:HGNC:23204]	-1.0816516
KIT	KIT proto-oncogene receptor tyrosine kinase [Source:HGNC Symbol;Acc:HGNC:6342]	1.149086
KLC1	kinesin light chain 1 [Source:HGNC Symbol;Acc:HGNC:6387]	-1.211962
KLC2	kinesin light chain 2 [Source:HGNC Symbol;Acc:HGNC:20716]	1.1763554
KLF11	Kruppel like factor 11 [Source:HGNC Symbol;Acc:HGNC:11811]	-1.2972825
KLF13	Kruppel like factor 13 [Source:HGNC Symbol;Acc:HGNC:13672]	1.053679
KLF15	Kruppel like factor 15 [Source:HGNC Symbol;Acc:HGNC:14536]	-1.168275
KLF2	Kruppel like factor 2 [Source:HGNC Symbol;Acc:HGNC:6347]	1.8338419
KLF3	Kruppel like factor 3 [Source:HGNC Symbol;Acc:HGNC:16516]	-1.3434803
KLF6	Kruppel like factor 6 [Source:HGNC Symbol;Acc:HGNC:2235]	-1.1862088
KLF7	Kruppel like factor 7 [Source:HGNC Symbol;Acc:HGNC:6350]	-1.7217264
KLHDC10	kelch domain containing 10 [Source:HGNC Symbol;Acc:HGNC:22194]	1.16742
KLHDC3	kelch domain containing 3 [Source:HGNC Symbol;Acc:HGNC:20704]	1.3041364
KLHDC4	kelch domain containing 4 [Source:HGNC Symbol;Acc:HGNC:25272]	-1.2552928
KLHDC8A	kelch domain containing 8A [Source:HGNC Symbol;Acc:HGNC:25573]	-1.1591922
KLHL21	kelch like family member 21 [Source:HGNC Symbol;Acc:HGNC:29041]	-1.3415769
KLHL22	kelch like family member 22 [Source:HGNC Symbol;Acc:HGNC:25888]	-1.0968361
KLHL29	kelch like family member 29 [Source:HGNC Symbol;Acc:HGNC:29404]	1.124055
KLHL3	kelch like family member 3 [Source:HGNC Symbol;Acc:HGNC:6354]	1.705701
KLHL33	kelch like family member 33 [Source:HGNC Symbol;Acc:HGNC:31952]	-1.1775262
KLHL35	kelch like family member 35 [Source:HGNC Symbol;Acc:HGNC:26597]	1.3110178
KLHL36	kelch like family member 36 [Source:HGNC Symbol;Acc:HGNC:17844]	-2.07655585
KLHL5	kelch like family member 5 [Source:HGNC Symbol;Acc:HGNC:6356]	-1.2760949
KLHL6	kelch like family member 6 [Source:HGNC Symbol;Acc:HGNC:18653]	1.34621075
KLHL7	kelch like family member 7 [Source:HGNC Symbol;Acc:HGNC:15646]	-1.2467619
KLK14	kallikrein related peptidase 14 [Source:HGNC Symbol;Acc:HGNC:6362]	1.111934
KLK2	kallikrein related peptidase 2 [Source:HGNC Symbol;Acc:HGNC:6363]	-1.2017409
KLK3	kallikrein related peptidase 3 [Source:HGNC Symbol;Acc:HGNC:6364]	-1.1380554
KLK7	kallikrein related peptidase 7 [Source:HGNC Symbol;Acc:HGNC:6368]	1.0780284
KLK9	kallikrein related peptidase 9 [Source:HGNC Symbol;Acc:HGNC:6370]	-1.1499418
KLRD1	killer cell lectin like receptor D1 [Source:HGNC Symbol;Acc:HGNC:6378]	1.118779
KLRF1	killer cell lectin like receptor F1 [Source:HGNC Symbol;Acc:HGNC:13342]	1.1372107
KPNA3	karyopherin subunit alpha 3 [Source:HGNC Symbol;Acc:HGNC:6396]	1.4870386
KPNA5	karyopherin subunit alpha 5 [Source:HGNC Symbol;Acc:HGNC:6398]	1.20115465
KREMEN1	kringle containing transmembrane protein 1 [Source:HGNC Symbol;Acc:HGNC:17550]	-1.31337725
KRT12	keratin 12 [Source:HGNC Symbol;Acc:HGNC:6414]	1.0628365
KRT16	keratin 16 [Source:HGNC Symbol;Acc:HGNC:6423]	1.0984526
KRT18	keratin 18 [Source:HGNC Symbol;Acc:HGNC:6430]	1.1631695
KRT18P55	keratin 18 pseudogene 55 [Source:HGNC Symbol;Acc:HGNC:26874]	1.2552879
KRT19	keratin 19 [Source:HGNC Symbol;Acc:HGNC:6436]	1.343165
KRT20	keratin 20 [Source:HGNC Symbol;Acc:HGNC:20412]	1.0814995
KRT32	keratin 32 [Source:HGNC Symbol;Acc:HGNC:6449]	-1.214745
KRT7	keratin 7 [Source:HGNC Symbol;Acc:HGNC:6445]	1.1977432
KRT74	keratin 74 [Source:HGNC Symbol;Acc:HGNC:28929]	-1.1463302
KRT79	keratin 79 [Source:HGNC Symbol;Acc:HGNC:28930]	1.1214811
KRT80	keratin 80 [Source:HGNC Symbol;Acc:HGNC:27056]	2.3721228
KRTAP10-10	keratin associated protein 10-10 [Source:HGNC Symbol;Acc:HGNC:22972]	-1.2159207

KRTAP10-11	keratin associated protein 10-11 [Source:HGNC Symbol;Acc:HGNC:20528]	1.0876746
KRTAP10-4	keratin associated protein 10-4 [Source:HGNC Symbol;Acc:HGNC:20521]	-1.1042213
KRTAP10-9	keratin associated protein 10-9 [Source:HGNC Symbol;Acc:HGNC:22971]	-1.1157311
KRTAP13-2	keratin associated protein 13-2 [Source:HGNC Symbol;Acc:HGNC:18923]	-1.0916367
KRTAP19-1	keratin associated protein 19-1 [Source:HGNC Symbol;Acc:HGNC:18936]	-1.0934671
KRTAP19-2	keratin associated protein 19-2 [Source:HGNC Symbol;Acc:HGNC:18937]	-1.1741942
KRTAP19-4	keratin associated protein 19-4 [Source:HGNC Symbol;Acc:HGNC:18939]	1.1121563
KRTAP20-3	keratin associated protein 20-3 [Source:HGNC Symbol;Acc:HGNC:34001]	-1.0737506
KRTAP21-1	keratin associated protein 21-1 [Source:HGNC Symbol;Acc:HGNC:18945]	-1.2780074
KRTAP21-2	keratin associated protein 21-2 [Source:HGNC Symbol;Acc:HGNC:18946]	-1.0685396
KRTAP27-1	keratin associated protein 27-1 [Source:HGNC Symbol;Acc:HGNC:33864]	1.0952574
KRTAP3-1	keratin associated protein 3-1 [Source:HGNC Symbol;Acc:HGNC:16778]	-1.0867842
KRTAP4-12	keratin associated protein 4-12 [Source:HGNC Symbol;Acc:HGNC:16776]	-1.152522
KRTAP4-4	keratin associated protein 4-4 [Source:HGNC Symbol;Acc:HGNC:16928]	1.1248299
KRTAP4-9	keratin associated protein 4-9 [Source:HGNC Symbol;Acc:HGNC:18910]	-1.1043428
KRTAP5-4	keratin associated protein 5-4 [Source:HGNC Symbol;Acc:HGNC:23599]	-1.1343261
KRTAP5-5	keratin associated protein 5-5 [Source:HGNC Symbol;Acc:HGNC:23601]	-1.1592612
KRTAP5-6	keratin associated protein 5-6 [Source:HGNC Symbol;Acc:HGNC:23600]	-1.095934
KRTAP9-1	keratin associated protein 9-1 [Source:HGNC Symbol;Acc:HGNC:18912]	-1.1233598
KRTCAP2	keratinocyte associated protein 2 [Source:HGNC Symbol;Acc:HGNC:28942]	-1.1462412
KSR1	kinase suppressor of ras 1 [Source:HGNC Symbol;Acc:HGNC:6465]	-1.26323
KSR2	kinase suppressor of ras 2 [Source:HGNC Symbol;Acc:HGNC:18610]	1.5434886
LACTB	lactamase beta [Source:HGNC Symbol;Acc:HGNC:16468]	-1.3462895
LACTB2	lactamase beta 2 [Source:HGNC Symbol;Acc:HGNC:18512]	1.286678
LAIR1	leukocyte associated immunoglobulin like receptor 1 [Source:HGNC Symbol;Acc:HGNC:6477]	1.0558828
LAIR2	leukocyte associated immunoglobulin like receptor 2 [Source:HGNC Symbol;Acc:HGNC:6478]	1.1539493
LAMA1	laminin subunit alpha 1 [Source:HGNC Symbol;Acc:HGNC:6481]	1.0761662
LAMA4	laminin subunit alpha 4 [Source:HGNC Symbol;Acc:HGNC:6484]	-1.1608424
LAMA5	laminin subunit alpha 5 [Source:HGNC Symbol;Acc:HGNC:6485]	1.1759576
LAMB1	laminin subunit beta 1 [Source:HGNC Symbol;Acc:HGNC:6486]	-1.3763217
LAMB2	laminin subunit beta 2 [Source:HGNC Symbol;Acc:HGNC:6487]	-1.1154493
LAMB3	laminin subunit beta 3 [Source:HGNC Symbol;Acc:HGNC:6490]	-2.6593647
LAMC2	laminin subunit gamma 2 [Source:HGNC Symbol;Acc:HGNC:6493]	-3.36951355
LAMP2	lysosomal associated membrane protein 2 [Source:HGNC Symbol;Acc:HGNC:6501]	1.3237523
LAMP3	lysosomal associated membrane protein 3 [Source:HGNC Symbol;Acc:HGNC:14582]	-1.3825214
LAMTOR2	ıte endosomal/lysosomal adaptor, MAPK and MTOR activator 2 [Source:HGNC Symbol;Acc:HGNC:2979(	1.176119
LANCL1	LanC like 1 [Source:HGNC Symbol;Acc:HGNC:6508]	1.2090051
LANCL3	LanC like 3 [Source:HGNC Symbol;Acc:HGNC:24767]	-1.5825136
LARP6	La ribonucleoprotein domain family member 6 [Source:HGNC Symbol;Acc:HGNC:24012]	-1.77167215
LAT2	linker for activation of T-cells family member 2 [Source:HGNC Symbol;Acc:HGNC:12749]	-1.1941824
LAX1	lymphocyte transmembrane adaptor 1 [Source:HGNC Symbol;Acc:HGNC:26005]	1.1328876
LBR	lamin B receptor [Source:HGNC Symbol;Acc:HGNC:6518]	1.35621
LCAT	lecithin-cholesterol acyltransferase [Source:HGNC Symbol;Acc:HGNC:6522]	-1.2431127
LCMT1	leucine carboxyl methyltransferase 1 [Source:HGNC Symbol;Acc:HGNC:17557]	-1.1402801
LCMT2	leucine carboxyl methyltransferase 2 [Source:HGNC Symbol;Acc:HGNC:17558]	-1.1926348
LCN8	lipocalin 8 [Source:HGNC Symbol;Acc:HGNC:27038]	1.1072338
LDB2	LIM domain binding 2 [Source:HGNC Symbol;Acc:HGNC:6533]	1.3065525
LDHC	lactate dehydrogenase C [Source:HGNC Symbol;Acc:HGNC:6544]	-1.28758
LDLR	low density lipoprotein receptor [Source:HGNC Symbol;Acc:HGNC:6547]	1.1372726
LDOC1	leucine zipper down-regulated in cancer 1 [Source:HGNC Symbol;Acc:HGNC:6548]	1.1703067
LECT1	leukocyte cell derived chemotaxin 1 [Source:HGNC Symbol;Acc:HGNC:17005]	-1.0792246
LEP	leptin [Source:HGNC Symbol;Acc:HGNC:6553]	1.052517
LETM1	eucine zipper and EF-hand containing transmembrane protein 1 [Source:HGNC Symbol;Acc:HGNC:6556	-1.1649483
LETM2	eucine zipper and EF-hand containing transmembrane protein 2 [Source:HGNC Symbol;Acc:HGNC:1464(	-1.0639598
LETMD1	LETM1 domain containing 1 [Source:HGNC Symbol;Acc:HGNC:24241]	1.0909616
LFNG	LFNG O-fucosylpeptide 3-beta-N-acetylglucosaminyltransferase [Source:HGNC Symbol;Acc:HGNC:6560]	1.6682773
LGALS14	galectin 14 [Source:HGNC Symbol;Acc:HGNC:30054]	1.1164047
LGALS2	galectin 2 [Source:HGNC Symbol;Acc:HGNC:6562]	1.0507044
LGALS3	galectin 3 [Source:HGNC Symbol;Acc:HGNC:6563]	-1.1592638
LGALS8	galectin 8 [Source:HGNC Symbol;Acc:HGNC:6569]	-1.221667
LGALS9C	galectin 9C [Source:HGNC Symbol;Acc:HGNC:33874]	-1.610029
LGMN	legumain [Source:HGNC Symbol;Acc:HGNC:9472]	-1.3354099
LGR4	leucine rich repeat containing G protein-coupled receptor 4 [Source:HGNC Symbol;Acc:HGNC:13299]	1.3657573
LGR6	leucine rich repeat containing G protein-coupled receptor 6 [Source:HGNC Symbol;Acc:HGNC:19719]	1.128515
LHFP	lipoma HMGIC fusion partner [Source:HGNC Symbol;Acc:HGNC:6586]	-1.3025622
LHFPL4	lipoma HMGIC fusion partner-like 4 [Source:HGNC Symbol;Acc:HGNC:29568]	-1.1669483
LHX6	LIM homeobox 6 [Source:HGNC Symbol;Acc:HGNC:21735]	1.7909405
LIF	leukemia inhibitory factor [Source:HGNC Symbol;Acc:HGNC:6596]	-1.442118
LIG3	DNA ligase 3 [Source:HGNC Symbol;Acc:HGNC:6600]	-1.2142191
LIMA1	LIM domain and actin binding 1 [Source:HGNC Symbol;Acc:HGNC:24636]	1.3701553
LIMCH1	LIM and calponin homology domains 1 [Source:HGNC Symbol;Acc:HGNC:29191]	1.7698032
LIMD1	LIM domains containing 1 [Source:HGNC Symbol;Acc:HGNC:6612]	1.129974
LIMD2	LIM domain containing 2 [Source:HGNC Symbol;Acc:HGNC:28142]	-1.5295537
LIMK2	LIM domain kinase 2 [Source:HGNC Symbol;Acc:HGNC:6614]	-1.49604595
LIMS2	LIM zinc finger domain containing 2 [Source:HGNC Symbol;Acc:HGNC:16084]	1.3352811
LIMS3L	LIM zinc finger domain containing 4	-1.0931861
LIN28A	lin-28 homolog A [Source:HGNC Symbol;Acc:HGNC:15986]	1.096894
LIN37	lin-37 DREAM MuvB core complex component [Source:HGNC Symbol;Acc:HGNC:33234]	-1.2702465
LIN54	lin-54 DREAM MuvB core complex component [Source:HGNC Symbol;Acc:HGNC:25397]	1.6556774
LINGO1	leucine rich repeat and Ig domain containing 1 [Source:HGNC Symbol;Acc:HGNC:21205]	1.1186749
LIPE	lipase E, hormone sensitive type [Source:HGNC Symbol;Acc:HGNC:6621]	1.4499587
LIPK	lipase family member K [Source:HGNC Symbol;Acc:HGNC:23444]	1.1416081
LITAF	lipopolysaccharide induced TNF factor [Source:HGNC Symbol;Acc:HGNC:16841]	-1.3813869
LMBR1	limb development membrane protein 1 [Source:HGNC Symbol;Acc:HGNC:13243]	1.1402656
LMBR1L	limb development membrane protein 1 like [Source:HGNC Symbol;Acc:HGNC:18268]	-1.2192345
LMCD1	LIM and cysteine rich domains 1 [Source:HGNC Symbol;Acc:HGNC:6633]	1.8152966

LMF2	lipase maturation factor 2 [Source:HGNC Symbol;Acc:HGNC:25096]	1.4821904
LMNA	lamin A/C [Source:HGNC Symbol;Acc:HGNC:6636]	1.1671559
LMNB1	lamin B1 [Source:HGNC Symbol;Acc:HGNC:6637]	1.4240129
LMO2	LIM domain only 2 [Source:HGNC Symbol;Acc:HGNC:6642]	1.3269219
LMO3	LIM domain only 3 [Source:HGNC Symbol;Acc:HGNC:6643]	1.0644913
LMO4	LIM domain only 4 [Source:HGNC Symbol;Acc:HGNC:6644]	1.4164028
LMO7	LIM domain 7 [Source:HGNC Symbol;Acc:HGNC:6646]	1.1182084
LMTK2	lemur tyrosine kinase 2 [Source:HGNC Symbol;Acc:HGNC:17880]	-1.1656594
LNX1	ligand of numb-protein X 1 [Source:HGNC Symbol;Acc:HGNC:6657]	1.1680574
LONRF3	LON peptidase N-terminal domain and ring finger 3 [Source:HGNC Symbol;Acc:HGNC:21152]	1.59733185
LOX	lysyl oxidase [Source:HGNC Symbol;Acc:HGNC:6664]	-1.1914655
LOXL2	lysyl oxidase like 2 [Source:HGNC Symbol;Acc:HGNC:6666]	-1.1572932
LOXL3	lysyl oxidase like 3 [Source:HGNC Symbol;Acc:HGNC:13869]	-1.1476412
LOXL4	lysyl oxidase like 4 [Source:HGNC Symbol;Acc:HGNC:17171]	-1.1571759
LPAR3	lysophosphatidic acid receptor 3 [Source:HGNC Symbol;Acc:HGNC:14298]	-1.0641958
LPCAT4	lysophosphatidylcholine acyltransferase 4 [Source:HGNC Symbol;Acc:HGNC:30059]	1.244596
LPHN2	adhesion G protein-coupled receptor L2	1.2963302
LRCH1	eucine rich repeats and calponin homology domain containing 1 [Source:HGNC Symbol;Acc:HGNC:2030	-1.1665282
LRG1	leucine rich alpha-2-glycoprotein 1 [Source:HGNC Symbol;Acc:HGNC:29480]	-1.4057187
LRIG1	leucine rich repeats and immunoglobulin like domains 1 [Source:HGNC Symbol;Acc:HGNC:17360]	-1.5004224
LRIG2	leucine rich repeats and immunoglobulin like domains 2 [Source:HGNC Symbol;Acc:HGNC:20889]	1.2528557
LRIG3	leucine rich repeats and immunoglobulin like domains 3 [Source:HGNC Symbol;Acc:HGNC:30991]	-2.158922
LRIT1	leucine rich repeat, Ig-like and transmembrane domains 1 [Source:HGNC Symbol;Acc:HGNC:23404]	1.0577539
LRMP	lymphoid restricted membrane protein [Source:HGNC Symbol;Acc:HGNC:6690]	1.1006272
LRP10	LDL receptor related protein 10 [Source:HGNC Symbol;Acc:HGNC:14553]	1.2956955
LRP12	LDL receptor related protein 12 [Source:HGNC Symbol;Acc:HGNC:31708]	-1.2618241
LRP4	LDL receptor related protein 4 [Source:HGNC Symbol;Acc:HGNC:6696]	-1.0742399
LRP5	LDL receptor related protein 5 [Source:HGNC Symbol;Acc:HGNC:6697]	1.3727422
LRP8	LDL receptor related protein 8 [Source:HGNC Symbol;Acc:HGNC:6700]	1.3845886
LRPPRC	leucine rich pentatricopeptide repeat containing [Source:HGNC Symbol;Acc:HGNC:15714]	1.3553634
LRRC10B	leucine rich repeat containing 10B [Source:HGNC Symbol;Acc:HGNC:37215]	1.1158272
LRRC17	leucine rich repeat containing 17 [Source:HGNC Symbol;Acc:HGNC:16895]	1.28771115
LRRC20	leucine rich repeat containing 20 [Source:HGNC Symbol;Acc:HGNC:23421]	1.700489
LRRC23	leucine rich repeat containing 23 [Source:HGNC Symbol;Acc:HGNC:19138]	-1.100099
LRRC27	leucine rich repeat containing 27 [Source:HGNC Symbol;Acc:HGNC:29346]	1.0714446
LRRC3	leucine rich repeat containing 3 [Source:HGNC Symbol;Acc:HGNC:14965]	-1.7772814
LRRC32	leucine rich repeat containing 32 [Source:HGNC Symbol;Acc:HGNC:4161]	-1.8764056
LRRC33	negative regulator of reactive oxygen species	1.351554
LRRC36	leucine rich repeat containing 36 [Source:HGNC Symbol;Acc:HGNC:25615]	-1.2503124
LRRC37BP1	leucine rich repeat containing 37B pseudogene 1 [Source:HGNC Symbol;Acc:HGNC:25390]	-1.162778
LRRC3C	leucine rich repeat containing 3C [Source:HGNC Symbol;Acc:HGNC:40034]	-1.2628129
LRRC4	leucine rich repeat containing 4 [Source:HGNC Symbol;Acc:HGNC:15586]	1.3209989
LRRC42	leucine rich repeat containing 42 [Source:HGNC Symbol;Acc:HGNC:28792]	-1.112394
LRRC47	leucine rich repeat containing 47 [Source:HGNC Symbol;Acc:HGNC:29207]	-1.0724989
LRRC57	leucine rich repeat containing 57 [Source:HGNC Symbol;Acc:HGNC:26719]	-1.3118026
LRRC66	leucine rich repeat containing 66 [Source:HGNC Symbol;Acc:HGNC:34299]	1.1906478
LRRC70	leucine rich repeat containing 70 [Source:HGNC Symbol;Acc:HGNC:35155]	1.6328385
LRRC8A	leucine rich repeat containing 8 family member A [Source:HGNC Symbol;Acc:HGNC:19027]	1.2774278
LRRC8D	leucine rich repeat containing 8 family member D [Source:HGNC Symbol;Acc:HGNC:16992]	1.3685906
LRRCC1	leucine rich repeat and coiled-coil centrosomal protein 1 [Source:HGNC Symbol;Acc:HGNC:29373]	1.1786047
LRRFIP2	LRR binding FLII interacting protein 2 [Source:HGNC Symbol;Acc:HGNC:6703]	-1.1465592
LRRN2	leucine rich repeat neuronal 2 [Source:HGNC Symbol;Acc:HGNC:16914]	-1.2214459
LRRTM1	leucine rich repeat transmembrane neuronal 1 [Source:HGNC Symbol;Acc:HGNC:19408]	-1.1170201
LRRTM2	leucine rich repeat transmembrane neuronal 2 [Source:HGNC Symbol;Acc:HGNC:19409]	1.0802752
LRRTM3	leucine rich repeat transmembrane neuronal 3 [Source:HGNC Symbol;Acc:HGNC:19410]	1.147034
LRTOMT	e rich transmembrane and O-methyltransferase domain containing [Source:HGNC Symbol;Acc:HGNC:2	-1.0664679
LSM10	LSM10, U7 small nuclear RNA associated [Source:HGNC Symbol;Acc:HGNC:17562]	1.1901011
LSM11	LSM11, U7 small nuclear RNA associated [Source:HGNC Symbol;Acc:HGNC:30860]	1.0894629
LSM12	LSM12 homolog [Source:HGNC Symbol;Acc:HGNC:26407]	1.3058231
LST1	leukocyte specific transcript 1 [Source:HGNC Symbol;Acc:HGNC:14189]	-1.276297
LTB	lymphotoxin beta [Source:HGNC Symbol;Acc:HGNC:6711]	-18.932013
LTBP2	latent transforming growth factor beta binding protein 2 [Source:HGNC Symbol;Acc:HGNC:6715]	1.2837393
LTC4S	leukotriene C4 synthase [Source:HGNC Symbol;Acc:HGNC:6719]	-1.0717883
LTV1	LTV1 ribosome biogenesis factor [Source:HGNC Symbol;Acc:HGNC:21173]	1.5690323
LUZP2	leucine zipper protein 2 [Source:HGNC Symbol;Acc:HGNC:23206]	1.0500579
LXN	latexin [Source:HGNC Symbol;Acc:HGNC:13347]	1.3052856
LY6E	lymphocyte antigen 6 complex, locus E [Source:HGNC Symbol;Acc:HGNC:6727]	1.4366633
LY6G6E	lymphocyte antigen 6 complex, locus G6E [Source:HGNC Symbol;Acc:HGNC:13934]	-1.1221377
LY86	lymphocyte antigen 86 [Source:HGNC Symbol;Acc:HGNC:16837]	-1.1978259
LYG1	lysozyme g1 [Source:HGNC Symbol;Acc:HGNC:27014]	1.0597007
LYL1	LYL1, basic helix-loop-helix family member [Source:HGNC Symbol;Acc:HGNC:6734]	1.9544288
LYN	LYN proto-oncogene, Src family tyrosine kinase [Source:HGNC Symbol;Acc:HGNC:6735]	1.4003502
LYNX1	Ly6/neurotoxin 1 [Source:HGNC Symbol;Acc:HGNC:29604]	-1.08229535
LYPD1	LY6/PLAUR domain containing 1 [Source:HGNC Symbol;Acc:HGNC:28431]	1.6627007
LYPD6	LY6/PLAUR domain containing 6 [Source:HGNC Symbol;Acc:HGNC:28751]	-1.8305895
LYPLA1	lysophospholipase I [Source:HGNC Symbol;Acc:HGNC:6737]	1.2579392
LYPLA2	lysophospholipase II [Source:HGNC Symbol;Acc:HGNC:6738]	1.3950955
LYRM4	LYR motif containing 4 [Source:HGNC Symbol;Acc:HGNC:21365]	-1.267826
LYSMD2	LysM domain containing 2 [Source:HGNC Symbol;Acc:HGNC:28571]	1.2302854
LYSMD4	LysM domain containing 4 [Source:HGNC Symbol;Acc:HGNC:26571]	-1.2102098
LYST	lysosomal trafficking regulator [Source:HGNC Symbol;Acc:HGNC:1968]	1.1439046
LYZL6	lysozyme like 6 [Source:HGNC Symbol;Acc:HGNC:29614]	1.0975963
LZIC	leucine zipper and CTNNBIP1 domain containing [Source:HGNC Symbol;Acc:HGNC:17497]	1.1074947
LZTS2	leucine zipper tumor suppressor 2 [Source:HGNC Symbol;Acc:HGNC:29381]	1.0875145
MAD2L1	MAD2 mitotic arrest deficient-like 1 (yeast) [Source:HGNC Symbol;Acc:HGNC:6763]	1.4784863
MAD2L1BP	MAD2L1 binding protein [Source:HGNC Symbol;Acc:HGNC:21059]	-1.1888171

MAD2L2	MAD2 mitotic arrest deficient-like 2 (yeast) [Source:HGNC Symbol;Acc:HGNC:6764]	-1.0844967
MAEA	macrophage erythroblast attacher [Source:HGNC Symbol;Acc:HGNC:13731]	-1.1533428
MAF1	MAF1 homolog, negative regulator of RNA polymerase III [Source:HGNC Symbol;Acc:HGNC:24966]	1.2340903
MAFB	MAF bZIP transcription factor B [Source:HGNC Symbol;Acc:HGNC:6408]	1.40811905
MAFF	MAF bZIP transcription factor F [Source:HGNC Symbol;Acc:HGNC:6780]	-1.7996502
MAFK	MAF bZIP transcription factor K [Source:HGNC Symbol;Acc:HGNC:6782]	1.1679516
MAGEA12	MAGE family member A12 [Source:HGNC Symbol;Acc:HGNC:6799]	1.0825344
MAGEB16	MAGE family member B16 [Source:HGNC Symbol;Acc:HGNC:21188]	1.0903957
MAGEB17	MAGE family member B17 [Source:HGNC Symbol;Acc:HGNC:17418]	1.1255401
MAGEB18	MAGE family member B18 [Source:HGNC Symbol;Acc:HGNC:28515]	1.1000487
MAGEC1	MAGE family member C1 [Source:HGNC Symbol;Acc:HGNC:6812]	-1.0932069
MAGED2	MAGE family member D2 [Source:HGNC Symbol;Acc:HGNC:16353]	-1.1548064
MAGEE1	MAGE family member E1 [Source:HGNC Symbol;Acc:HGNC:24934]	1.3016329
MAGI1	brane associated guanylate kinase, WW and PDZ domain containing 1 [Source:HGNC Symbol;Acc:HGNC	-1.1452323
MAK16	MAK16 homolog [Source:HGNC Symbol;Acc:HGNC:13703]	1.3633536
MALL	mal, T-cell differentiation protein like [Source:HGNC Symbol;Acc:HGNC:6818]	1.2325159
MAMDC4	MAM domain containing 4 [Source:HGNC Symbol;Acc:HGNC:24083]	-1.1120011
MAN2A2	mannosidase alpha class 2A member 2 [Source:HGNC Symbol;Acc:HGNC:6825]	-1.0812061
MAN2B1	mannosidase alpha class 2B member 1 [Source:HGNC Symbol;Acc:HGNC:6826]	-1.2858471
MAN2B2	mannosidase alpha class 2B member 2 [Source:HGNC Symbol;Acc:HGNC:29623]	-1.1802673
MAP2	microtubule associated protein 2 [Source:HGNC Symbol;Acc:HGNC:6839]	1.3737687
MAP2K1	mitogen-activated protein kinase kinase 1 [Source:HGNC Symbol;Acc:HGNC:6840]	-1.3636717
MAP2K3	mitogen-activated protein kinase kinase 3 [Source:HGNC Symbol;Acc:HGNC:6843]	-2.04736985
MAP2K6	mitogen-activated protein kinase kinase 6 [Source:HGNC Symbol;Acc:HGNC:6846]	1.3487295
MAP3K1	mitogen-activated protein kinase kinase kinase 1 [Source:HGNC Symbol;Acc:HGNC:6848]	-1.6828507
MAP3K11	mitogen-activated protein kinase kinase kinase 11 [Source:HGNC Symbol;Acc:HGNC:6850]	-1.4856573
MAP3K12	mitogen-activated protein kinase kinase kinase 12 [Source:HGNC Symbol;Acc:HGNC:6851]	1.2681408
MAP3K5	mitogen-activated protein kinase kinase kinase 5 [Source:HGNC Symbol;Acc:HGNC:6857]	-1.2381017
MAP3K8	mitogen-activated protein kinase kinase kinase 8 [Source:HGNC Symbol;Acc:HGNC:6860]	-2.29677995
MAP6D1	MAP6 domain containing 1 [Source:HGNC Symbol;Acc:HGNC:25753]	1.2164681
MAP7D1	MAP7 domain containing 1 [Source:HGNC Symbol;Acc:HGNC:25514]	-1.18308955
MAP7D3	MAP7 domain containing 3 [Source:HGNC Symbol;Acc:HGNC:25742]	1.2764419
MAP9	microtubule associated protein 9 [Source:HGNC Symbol;Acc:HGNC:26118]	-1.1121889
MAPK10	mitogen-activated protein kinase 10 [Source:HGNC Symbol;Acc:HGNC:6872]	1.0967678
MAPK1IP1L	mitogen-activated protein kinase 1 interacting protein 1 like [Source:HGNC Symbol;Acc:HGNC:19840]	-1.0867488
MAPKBP1	mitogen-activated protein kinase binding protein 1 [Source:HGNC Symbol;Acc:HGNC:29536]	-1.4924357
MAPRE1	microtubule associated protein RP/EB family member 1 [Source:HGNC Symbol;Acc:HGNC:6890]	-1.1757857
MARCH1	membrane associated ring-CH-type finger 1 [Source:HGNC Symbol;Acc:HGNC:26077]	1.0513215
MARCH8	membrane associated ring-CH-type finger 8 [Source:HGNC Symbol;Acc:HGNC:23356]	-1.1295424
MARK4	microtubule affinity regulating kinase 4 [Source:HGNC Symbol;Acc:HGNC:13538]	1.3102645
MARVELD2	MARVEL domain containing 2 [Source:HGNC Symbol;Acc:HGNC:26401]	1.0867399
MAST3	microtubule associated serine/threonine kinase 3 [Source:HGNC Symbol;Acc:HGNC:19036]	1.3020526
MAT2A	methionine adenosyltransferase 2A [Source:HGNC Symbol;Acc:HGNC:6904]	1.3899198
MATN1	matrilin 1, cartilage matrix protein [Source:HGNC Symbol;Acc:HGNC:6907]	-1.0770924
MAVS	mitochondrial antiviral signaling protein [Source:HGNC Symbol;Acc:HGNC:29233]	-1.2003858
MAX	MYC associated factor X [Source:HGNC Symbol;Acc:HGNC:6913]	-1.1277468
MAZ	MYC associated zinc finger protein [Source:HGNC Symbol;Acc:HGNC:6914]	1.2998579
MB21D1	Mab-21 domain containing 1 [Source:HGNC Symbol;Acc:HGNC:21367]	-1.8753603
MBD2	methyl-CpG binding domain protein 2 [Source:HGNC Symbol;Acc:HGNC:6917]	1.0632762
MBD4	methyl-CpG binding domain 4, DNA glycosylase [Source:HGNC Symbol;Acc:HGNC:6919]	1.3388196
MBD6	methyl-CpG binding domain protein 6 [Source:HGNC Symbol;Acc:HGNC:20445]	1.1919225
MBL1P	mannose binding lectin 1, pseudogene [Source:HGNC Symbol;Acc:HGNC:6921]	1.2029426
MBLAC2	metallo-beta-lactamase domain containing 2 [Source:HGNC Symbol;Acc:HGNC:33711]	1.5626351
MBNL1	muscleblind like splicing regulator 1 [Source:HGNC Symbol;Acc:HGNC:6923]	1.9377193
MBOAT7	membrane bound O-acyltransferase domain containing 7 [Source:HGNC Symbol;Acc:HGNC:15505]	1.1796237
MBP	myelin basic protein [Source:HGNC Symbol;Acc:HGNC:6925]	-1.5389285
MC5R	melanocortin 5 receptor [Source:HGNC Symbol;Acc:HGNC:6933]	-1.167776
MCEE	methylmalonyl-CoA epimerase [Source:HGNC Symbol;Acc:HGNC:16732]	1.1203659
MCM10	minichromosome maintenance 10 replication initiation factor [Source:HGNC Symbol;Acc:HGNC:18043]	1.3772166
MCM8	ıromosome maintenance 8 homologous recombination repair factor [Source:HGNC Symbol;Acc:HGNC::	1.2662569
MCTP1	multiple C2 and transmembrane domain containing 1 [Source:HGNC Symbol;Acc:HGNC:26183]	-1.3161192
MDFI	MyoD family inhibitor [Source:HGNC Symbol;Acc:HGNC:6967]	2.0532594
MDFIC	MyoD family inhibitor domain containing [Source:HGNC Symbol;Acc:HGNC:28870]	1.2545575
MDGA1	MAM domain containing glycosylphosphatidylinositol anchor 1 [Source:HGNC Symbol;Acc:HGNC:19267	-1.2395235
MDK	midkine (neurite growth-promoting factor 2) [Source:HGNC Symbol;Acc:HGNC:6972]	1.1536686
MECOM	MDS1 and EVI1 complex locus [Source:HGNC Symbol;Acc:HGNC:3498]	1.5965287
MECP2	methyl-CpG binding protein 2 [Source:HGNC Symbol;Acc:HGNC:6990]	-1.1727586
MED12L	mediator complex subunit 12 like [Source:HGNC Symbol;Acc:HGNC:16050]	1.0820377
MED15	mediator complex subunit 15 [Source:HGNC Symbol;Acc:HGNC:14248]	-1.1365343
MED18	mediator complex subunit 18 [Source:HGNC Symbol;Acc:HGNC:25944]	1.3001593
MED22	mediator complex subunit 22 [Source:HGNC Symbol;Acc:HGNC:11477]	-1.1676494
MED24	mediator complex subunit 24 [Source:HGNC Symbol;Acc:HGNC:22963]	-1.2291209
MED30	mediator complex subunit 30 [Source:HGNC Symbol;Acc:HGNC:23032]	1.1423309
MED7	mediator complex subunit 7 [Source:HGNC Symbol;Acc:HGNC:2378]	1.1232998
MED8	mediator complex subunit 8 [Source:HGNC Symbol;Acc:HGNC:19971]	1.1588145
MEF2C	myocyte enhancer factor 2C [Source:HGNC Symbol;Acc:HGNC:6996]	1.732577
MEG3	maternally expressed 3 (non-protein coding) [Source:HGNC Symbol;Acc:HGNC:14575]	1.1385375
MEGF11	multiple EGF like domains 11 [Source:HGNC Symbol;Acc:HGNC:29635]	-1.259317
MEIS1	Meis homeobox 1 [Source:HGNC Symbol;Acc:HGNC:7000]	1.2862391
MEIS3	Meis homeobox 3 [Source:HGNC Symbol;Acc:HGNC:29537]	-1.1067299
MEMO1	mediator of cell motility 1 [Source:HGNC Symbol;Acc:HGNC:14014]	-1.2563117
MEOX1	mesenchyme homeobox 1 [Source:HGNC Symbol;Acc:HGNC:7013]	-1.0681096
MEOX2	mesenchyme homeobox 2 [Source:HGNC Symbol;Acc:HGNC:7014]	1.2465036
MERTK	MER proto-oncogene, tyrosine kinase [Source:HGNC Symbol;Acc:HGNC:7027]	1.7708983
MESDC1	mesoderm development candidate 1 [Source:HGNC Symbol;Acc:HGNC:13519]	-1.2323112
MESP2	mesoderm posterior bHLH transcription factor 2 [Source:HGNC Symbol;Acc:HGNC:29659]	-1.3541715

MET	MET proto-oncogene, receptor tyrosine kinase [Source:HGNC Symbol;Acc:HGNC:7029]	1.4105467
METAP2	methionyl aminopeptidase 2 [Source:HGNC Symbol;Acc:HGNC:16672]	1.3503747
METRNL	meteorin like, glial cell differentiation regulator [Source:HGNC Symbol;Acc:HGNC:27584]	1.2885134
METTL16	methyltransferase like 16 [Source:HGNC Symbol;Acc:HGNC:28484]	-1.1863065
METTL19	tRNA methyltransferase 44 homolog (S. cerevisiae)	-1.2194794
METTL20	electron transfer flavoprotein beta subunit lysine methyltransferase	1.1098088
METTL21A	methyltransferase like 21A [Source:HGNC Symbol;Acc:HGNC:30476]	-1.1722832
METTL21D	valosin containing protein lysine methyltransferase	1.3069048
METTL23	methyltransferase like 23 [Source:HGNC Symbol;Acc:HGNC:26988]	1.0751764
METTL2A	methyltransferase like 2A [Source:HGNC Symbol;Acc:HGNC:25755]	-1.2067413
METTL5	methyltransferase like 5 [Source:HGNC Symbol;Acc:HGNC:25006]	1.1911906
METTL7A	methyltransferase like 7A [Source:HGNC Symbol;Acc:HGNC:24550]	1.2778786
METTL8	methyltransferase like 8 [Source:HGNC Symbol;Acc:HGNC:25856]	1.1501254
MEX3A	mex-3 RNA binding family member A [Source:HGNC Symbol;Acc:HGNC:33482]	1.0990336
MEX3D	mex-3 RNA binding family member D [Source:HGNC Symbol;Acc:HGNC:16734]	-1.1856425
MFAP1	microfibrillar associated protein 1 [Source:HGNC Symbol;Acc:HGNC:7032]	1.2509974
MFF	mitochondrial fission factor [Source:HGNC Symbol;Acc:HGNC:24858]	1.05885925
MFGE8	milk fat globule-EGF factor 8 protein [Source:HGNC Symbol;Acc:HGNC:7036]	1.2469249
MFHAS1	malignant fibrous histiocytoma amplified sequence 1 [Source:HGNC Symbol;Acc:HGNC:16982]	-1.2710286
MFNG	MFNG O-fucosylpeptide 3-beta-N-acetylglucosaminyltransferase [Source:HGNC Symbol;Acc:HGNC:7038	1.1598064
MFSD10	major facilitator superfamily domain containing 10 [Source:HGNC Symbol;Acc:HGNC:16894]	1.2072862
MFSD2B	major facilitator superfamily domain containing 2B [Source:HGNC Symbol;Acc:HGNC:37207]	-1.0713822
MFSD9	major facilitator superfamily domain containing 9 [Source:HGNC Symbol;Acc:HGNC:28158]	-1.1633606
MGAT1	syl (alpha-1,3-)-glycoprotein beta-1,2-N-acetylglucosaminyltransferase [Source:HGNC Symbol;Acc:HGN	1.1674305
MGAT3	osyl (beta-1,4-)-glycoprotein beta-1,4-N-acetylglucosaminyltransferase [Source:HGNC Symbol;Acc:HGNC	1.135487
MGAT4A	pha-1,3-)-glycoprotein beta-1,4-N-acetylglucosaminyltransferase, isozyme A [Source:HGNC Symbol;Acc	1.4266751
MGAT5B	ha-1,6-)-glycoprotein beta-1,6-N-acetyl-glucosaminyltransferase, isozyme B [Source:HGNC Symbol;Acc:	1.3078734
MGC11082	DLGAP1 antisense RNA 2	-1.6289334
MGC16121	MIR503 host gene	1.3811054
MGC16703	tubulin alpha 3f pseudogene	-1.2418202
MGC21881	glioblastoma down-regulated RNA	-1.2184538
MGC2752	CENPB DNA-binding domains containing 1 pseudogene 1	-1.1507071
MGC2889	uncharacterized protein MGC2889	1.0799145
MGC39372	serpin family B member 9 pseudogene 1	-1.1760616
MGC4294	long intergenic non-protein coding RNA 1711	1.2049396
MGC45800	uncharacterized LOC90768	-1.0570949
MGC4859	uncharacterized LOC79150	-1.1151105
MGLL	monoglyceride lipase [Source:HGNC Symbol;Acc:HGNC:17038]	-1.4361192
MGP	matrix Gla protein [Source:HGNC Symbol;Acc:HGNC:7060]	-1.2073275
MGRN1	mahogunin ring finger 1 [Source:HGNC Symbol;Acc:HGNC:20254]	-1.2108285
MGST2	microsomal glutathione S-transferase 2 [Source:HGNC Symbol;Acc:HGNC:7063]	1.105127
MGST3	microsomal glutathione S-transferase 3 [Source:HGNC Symbol;Acc:HGNC:7064]	1.1515713
MICAL3	ule associated monooxygenase, calponin and LIM domain containing 3 [Source:HGNC Symbol;Acc:HGN	-1.643714967
MICB	MHC class I polypeptide-related sequence B [Source:HGNC Symbol;Acc:HGNC:7091]	-1.27163005
MID1IP1	MID1 interacting protein 1 [Source:HGNC Symbol;Acc:HGNC:20715]	1.0643475
MID2	midline 2 [Source:HGNC Symbol;Acc:HGNC:7096]	-1.0976106
MIDN	midnolin [Source:HGNC Symbol;Acc:HGNC:16298]	1.2447886
MIER1	MIER1 transcriptional regulator [Source:HGNC Symbol;Acc:HGNC:29657]	1.3780077
MINK1	misshapen like kinase 1 [Source:HGNC Symbol;Acc:HGNC:17565]	1.1205242
MIOS	meiosis regulator for oocyte development [Source:HGNC Symbol;Acc:HGNC:21905]	-1.0545697
MIOX	myo-inositol oxygenase [Source:HGNC Symbol;Acc:HGNC:14522]	-1.2139149
MIP	major intrinsic protein of lens fiber [Source:HGNC Symbol;Acc:HGNC:7103]	1.4521441
MIR155HG	MIR155 host gene [Source:HGNC Symbol;Acc:HGNC:35460]	-2.5394192
MIRLET7BHG	MIRLET7B host gene [Source:HGNC Symbol;Acc:HGNC:37189]	1.3963397
MKI67IP	nucleolar protein interacting with the FHA domain of MKI67	1.38307525
MKKS	McKusick-Kaufman syndrome [Source:HGNC Symbol;Acc:HGNC:7108]	1.1649472
MKL1	megakaryoblastic leukemia (translocation) 1 [Source:HGNC Symbol;Acc:HGNC:14334]	-1.4735155
MKNK2	MAP kinase interacting serine/threonine kinase 2 [Source:HGNC Symbol;Acc:HGNC:7111]	1.225927
MLF1	myeloid leukemia factor 1 [Source:HGNC Symbol;Acc:HGNC:7125]	1.096421
MLF2	myeloid leukemia factor 2 [Source:HGNC Symbol;Acc:HGNC:7126]	1.1868397
MLL	lysine methyltransferase 2A	-1.102478
MLL2	lysine methyltransferase 2B	1.0893265
MLLT1	MLLT1, super elongation complex subunit [Source:HGNC Symbol;Acc:HGNC:7134]	1.31923705
MLLT4	afadin, adherens junction formation factor	-1.1177746
MLNR	motilin receptor [Source:HGNC Symbol;Acc:HGNC:4495]	-1.0594629
MLPH	melanophilin [Source:HGNC Symbol;Acc:HGNC:29643]	-1.3020072
MLX	MLX, MAX dimerization protein [Source:HGNC Symbol;Acc:HGNC:11645]	1.0578768
MLXIP	MLX interacting protein [Source:HGNC Symbol;Acc:HGNC:17055]	1.2191186
MLYCD	malonyl-CoA decarboxylase [Source:HGNC Symbol;Acc:HGNC:7150]	1.214921
MMD	monocyte to macrophage differentiation associated [Source:HGNC Symbol;Acc:HGNC:7153]	-1.885646
MMP10	matrix metallopeptidase 10 [Source:HGNC Symbol;Acc:HGNC:7156]	-1.6477629
MMP14	matrix metallopeptidase 14 [Source:HGNC Symbol;Acc:HGNC:7160]	1.1654681
MMP16	matrix metallopeptidase 16 [Source:HGNC Symbol;Acc:HGNC:7162]	1.1834044
MMP19	matrix metallopeptidase 19 [Source:HGNC Symbol;Acc:HGNC:7165]	-1.4415941
MMP23B	matrix metallopeptidase 23B [Source:HGNC Symbol;Acc:HGNC:7171]	-1.1704142
MMP25	matrix metallopeptidase 25 [Source:HGNC Symbol;Acc:HGNC:14246]	-1.1620309
MMP27	matrix metallopeptidase 27 [Source:HGNC Symbol;Acc:HGNC:14250]	1.0879872
MMP9	matrix metallopeptidase 9 [Source:HGNC Symbol;Acc:HGNC:7176]	-1.1594485
MMRN2	multimerin 2 [Source:HGNC Symbol;Acc:HGNC:19888]	-1.1682253
MND1	meiotic nuclear divisions 1 [Source:HGNC Symbol;Acc:HGNC:24839]	1.204853
MNT	MAX network transcriptional repressor [Source:HGNC Symbol;Acc:HGNC:7188]	1.1576973
MOB2	MOB kinase activator 2 [Source:HGNC Symbol;Acc:HGNC:24904]	-1.1598198
MOBKL1B	MOB kinase activator 1A	1.1134045
MOBKL2C	MOB kinase activator 3C	-2.2491398
MOBKL3	MOB family member 4, phocein	1.24318365
MOBP	myelin-associated oligodendrocyte basic protein [Source:HGNC Symbol;Acc:HGNC:7189]	-1.1642436

MOCS1	molybdenum cofactor synthesis 1 [Source:HGNC Symbol;Acc:HGNC:7190]	1.1009277
MOGAT1	monoacylglycerol O-acyltransferase 1 [Source:HGNC Symbol;Acc:HGNC:18210]	-1.2528812
MORC2-AS1	MORC2 antisense RNA 1 [Source:HGNC Symbol;Acc:HGNC:26662]	1.1463794
MORN1	MORN repeat containing 1 [Source:HGNC Symbol;Acc:HGNC:25852]	-1.1297684
MORN3	MORN repeat containing 3 [Source:HGNC Symbol;Acc:HGNC:29807]	-1.13824
MORN4	MORN repeat containing 4 [Source:HGNC Symbol;Acc:HGNC:24001]	1.1473721
MOSC2	mitochondrial amidoxime reducing component 2	-1.1242934
MOSPD1	motile sperm domain containing 1 [Source:HGNC Symbol;Acc:HGNC:25235]	1.1763078
MOV10L1	Mov10 RISC complex RNA helicase like 1 [Source:HGNC Symbol;Acc:HGNC:7201]	-1.0674996
MPHOSPH6	M-phase phosphoprotein 6 [Source:HGNC Symbol;Acc:HGNC:7214]	1.1629133
MPO	myeloperoxidase [Source:HGNC Symbol;Acc:HGNC:7218]	1.0691131
MPP7	membrane palmitoylated protein 7 [Source:HGNC Symbol;Acc:HGNC:26542]	1.1265938
MPPE1	metallophosphoesterase 1 [Source:HGNC Symbol;Acc:HGNC:15988]	1.1038941
MPPED1	metallophosphoesterase domain containing 1 [Source:HGNC Symbol;Acc:HGNC:1306]	-1.0636511
MPRIP	myosin phosphatase Rho interacting protein [Source:HGNC Symbol;Acc:HGNC:30321]	-1.3379905
MPZ	myelin protein zero [Source:HGNC Symbol;Acc:HGNC:7225]	1.0962515
MPZL1	myelin protein zero like 1 [Source:HGNC Symbol;Acc:HGNC:7226]	-1.4519815
MPZL2	myelin protein zero like 2 [Source:HGNC Symbol;Acc:HGNC:3496]	1.323316
MPZL3	myelin protein zero like 3 [Source:HGNC Symbol;Acc:HGNC:27279]	1.0789009
MRC1	mannose receptor, C type 1 [Source:HGNC Symbol;Acc:HGNC:7228]	-1.0991772
MREG	melanoregulin [Source:HGNC Symbol;Acc:HGNC:25478]	-1.3085295
MRFAP1L1	Morf4 family associated protein 1 like 1 [Source:HGNC Symbol;Acc:HGNC:28796]	1.1562331
MRGPRE	MAS related GPR family member E [Source:HGNC Symbol;Acc:HGNC:30694]	-1.1289794
MRGPRX1	MAS related GPR family member X1 [Source:HGNC Symbol;Acc:HGNC:17962]	-1.0760711
MRGPRX2	MAS related GPR family member X2 [Source:HGNC Symbol;Acc:HGNC:17983]	1.0708988
MRI1	methylthioribose-1-phosphate isomerase 1 [Source:HGNC Symbol;Acc:HGNC:28469]	-1.2267312
MRPL14	mitochondrial ribosomal protein L14 [Source:HGNC Symbol;Acc:HGNC:14279]	-1.1930573
MRPL15	mitochondrial ribosomal protein L15 [Source:HGNC Symbol;Acc:HGNC:14054]	1.1982453
MRPL16	mitochondrial ribosomal protein L16 [Source:HGNC Symbol;Acc:HGNC:14476]	1.0670502
MRPL17	mitochondrial ribosomal protein L17 [Source:HGNC Symbol;Acc:HGNC:14053]	1.2097844
MRPL19	mitochondrial ribosomal protein L19 [Source:HGNC Symbol;Acc:HGNC:14052]	1.3548182
MRPL20	mitochondrial ribosomal protein L20 [Source:HGNC Symbol;Acc:HGNC:14478]	1.1055131
MRPL22	mitochondrial ribosomal protein L22 [Source:HGNC Symbol;Acc:HGNC:14480]	1.1027917
MRPL34	mitochondrial ribosomal protein L34 [Source:HGNC Symbol;Acc:HGNC:14488]	1.0947809
MRPL36	mitochondrial ribosomal protein L36 [Source:HGNC Symbol;Acc:HGNC:14490]	1.0553206
MRPL42	mitochondrial ribosomal protein L42 [Source:HGNC Symbol;Acc:HGNC:14493]	1.4151822
MRPL43	mitochondrial ribosomal protein L43 [Source:HGNC Symbol;Acc:HGNC:14517]	-1.0981148
MRPL45	mitochondrial ribosomal protein L45 [Source:HGNC Symbol;Acc:HGNC:16651]	-1.1249841
MRPL46	mitochondrial ribosomal protein L46 [Source:HGNC Symbol;Acc:HGNC:1192]	-1.1880796
MRPL48	mitochondrial ribosomal protein L48 [Source:HGNC Symbol;Acc:HGNC:16653]	-1.1897266
MRPL50	mitochondrial ribosomal protein L50 [Source:HGNC Symbol;Acc:HGNC:16654]	1.4921547
MRPL51	mitochondrial ribosomal protein L51 [Source:HGNC Symbol;Acc:HGNC:14044]	1.208421
MRPS17	mitochondrial ribosomal protein S17 [Source:HGNC Symbol;Acc:HGNC:14047]	1.0989085
MRPS18A	mitochondrial ribosomal protein S18A [Source:HGNC Symbol;Acc:HGNC:14515]	-1.2290243
MRPS21	mitochondrial ribosomal protein S21 [Source:HGNC Symbol;Acc:HGNC:14046]	1.0657355
MRPS26	mitochondrial ribosomal protein S26 [Source:HGNC Symbol;Acc:HGNC:14045]	1.1707754
MRPS31	mitochondrial ribosomal protein S31 [Source:HGNC Symbol;Acc:HGNC:16632]	1.2290998
MRPS7	mitochondrial ribosomal protein S7 [Source:HGNC Symbol;Acc:HGNC:14499]	-1.1262017
MRTO4	MRT4 homolog, ribosome maturation factor [Source:HGNC Symbol;Acc:HGNC:18477]	-1.1262721
MS4A14	membrane spanning 4-domains A14 [Source:HGNC Symbol;Acc:HGNC:30706]	-1.129945
MSH2	mutS homolog 2 [Source:HGNC Symbol;Acc:HGNC:7325]	1.4469556
MSH5	mutS homolog 5 [Source:HGNC Symbol;Acc:HGNC:7328]	1.0866443
MSH6	mutS homolog 6 [Source:HGNC Symbol;Acc:HGNC:7329]	1.527479
MSI2	musashi RNA binding protein 2 [Source:HGNC Symbol;Acc:HGNC:18585]	-1.21237435
MSL3	male-specific lethal 3 homolog (Drosophila) [Source:HGNC Symbol;Acc:HGNC:7370]	-1.2064348
MSTN	myostatin [Source:HGNC Symbol;Acc:HGNC:4223]	1.0734158
MSX1	msh homeobox 1 [Source:HGNC Symbol;Acc:HGNC:7391]	-3.1839964
MSX2	msh homeobox 2 [Source:HGNC Symbol;Acc:HGNC:7392]	-1.2300427
MT1G	metallothionein 1G [Source:HGNC Symbol;Acc:HGNC:7399]	-1.3278118
MT1H	metallothionein 1H [Source:HGNC Symbol;Acc:HGNC:7400]	-1.1419755
MT1IP	metallothionein 1I, pseudogene	-1.0530771
MT4	metallothionein 4 [Source:HGNC Symbol;Acc:HGNC:18705]	1.1051198
MTA1	metastasis associated 1 [Source:HGNC Symbol;Acc:HGNC:7410]	1.179742
MTA2	metastasis associated 1 family member 2 [Source:HGNC Symbol;Acc:HGNC:7411]	1.1813836
MTERFD1	mitochondrial transcription termination factor 3	1.2724098
MTERFD2	mitochondrial transcription termination factor 4	1.1652098
MTERFD3	mitochondrial transcription termination factor 2	1.3274268
MTF2	metal response element binding transcription factor 2 [Source:HGNC Symbol;Acc:HGNC:29535]	1.3621438
MTFP1	mitochondrial fission process 1 [Source:HGNC Symbol;Acc:HGNC:26945]	-1.1728021
MTHFR	methylenetetrahydrofolate reductase (NAD(P)H) [Source:HGNC Symbol;Acc:HGNC:7436]	1.2096134
MTIF2	mitochondrial translational initiation factor 2 [Source:HGNC Symbol;Acc:HGNC:7441]	1.0948825
MTIF3	mitochondrial translational initiation factor 3 [Source:HGNC Symbol;Acc:HGNC:29788]	1.335104
MTMR10	myotubularin related protein 10 [Source:HGNC Symbol;Acc:HGNC:25999]	-1.0820256
MTMR11	myotubularin related protein 11 [Source:HGNC Symbol;Acc:HGNC:24307]	-1.2663863
MTMR14	myotubularin related protein 14 [Source:HGNC Symbol;Acc:HGNC:26190]	-1.34764825
MTMR9LP	myotubularin related protein 9-like, pseudogene [Source:HGNC Symbol;Acc:HGNC:27920]	-1.37028625
MTPAP	mitochondrial poly(A) polymerase [Source:HGNC Symbol;Acc:HGNC:25532]	1.2529621
MTSS1	metastasis suppressor 1 [Source:HGNC Symbol;Acc:HGNC:20443]	-1.922131
MTX1	metaxin 1 [Source:HGNC Symbol;Acc:HGNC:7504]	-1.2283148
MUC1	mucin 1, cell surface associated [Source:HGNC Symbol;Acc:HGNC:7508]	-1.4117757
MUC12	mucin 12, cell surface associated [Source:HGNC Symbol;Acc:HGNC:7510]	-1.0558068
MUC15	mucin 15, cell surface associated [Source:HGNC Symbol;Acc:HGNC:14956]	1.0891427
MUC2	mucin 2, oligomeric mucus/gel-forming [Source:HGNC Symbol;Acc:HGNC:7512]	-1.1619539
MUC4	mucin 4, cell surface associated [Source:HGNC Symbol;Acc:HGNC:7514]	-1.1270977
MUC5AC	mucin 5AC, oligomeric mucus/gel-forming [Source:HGNC Symbol;Acc:HGNC:7515]	-1.1231213
MUM1	melanoma associated antigen (mutated) 1 [Source:HGNC Symbol;Acc:HGNC:29641]	1.18484055

MUS81	MUS81 structure-specific endonuclease subunit [Source:HGNC Symbol;Acc:HGNC:29814]	-1.1942885
MUT	methylmalonyl-CoA mutase [Source:HGNC Symbol;Acc:HGNC:7526]	1.1682402
MUTED	biogenesis of lysosomal organelles complex 1 subunit 5	1.2328994
MVK	mevalonate kinase [Source:HGNC Symbol;Acc:HGNC:7530]	1.1684704
MVP	major vault protein [Source:HGNC Symbol;Acc:HGNC:7531]	-1.1868476
MX1	MX dynamin like GTPase 1 [Source:HGNC Symbol;Acc:HGNC:7532]	-1.8759524
MXD1	MAX dimerization protein 1 [Source:HGNC Symbol;Acc:HGNC:6761]	-1.2648048
MYADM	myeloid associated differentiation marker [Source:HGNC Symbol;Acc:HGNC:7544]	-1.1319844
MYCL1	v-myc avian myelocytomatosis viral oncogene lung carcinoma derived homolog	-1.0625073
MYCT1	myc target 1 [Source:HGNC Symbol;Acc:HGNC:23172]	1.4600176
MYD88	myeloid differentiation primary response 88 [Source:HGNC Symbol;Acc:HGNC:7562]	-1.1479787
MYEOV	myeloma overexpressed [Source:HGNC Symbol;Acc:HGNC:7563]	-1.3450613
MYH14	myosin heavy chain 14 [Source:HGNC Symbol;Acc:HGNC:23212]	-1.1081489
MYH7B	myosin heavy chain 7B [Source:HGNC Symbol;Acc:HGNC:15906]	-1.0897846
MYH9	myosin heavy chain 9 [Source:HGNC Symbol;Acc:HGNC:7579]	-1.1162379
MYLK	myosin light chain kinase [Source:HGNC Symbol;Acc:HGNC:7590]	-1.2029926
MYLK2	myosin light chain kinase 2 [Source:HGNC Symbol;Acc:HGNC:16243]	1.3992043
MYNN	myoneurin [Source:HGNC Symbol;Acc:HGNC:14955]	1.1969945
MYO10	myosin X [Source:HGNC Symbol;Acc:HGNC:7593]	-1.2484334
MYO16	myosin XVI [Source:HGNC Symbol;Acc:HGNC:29822]	1.113762
MYO19	myosin XIX [Source:HGNC Symbol;Acc:HGNC:26234]	1.3355765
MYO1B	myosin IB [Source:HGNC Symbol;Acc:HGNC:7596]	1.1828009
MYO1D	myosin ID [Source:HGNC Symbol;Acc:HGNC:7598]	-1.1997279
MYO3B	myosin IIIB [Source:HGNC Symbol;Acc:HGNC:15576]	1.0970323
MYO5B	myosin VB [Source:HGNC Symbol;Acc:HGNC:7603]	1.1730849
MYO9B	myosin IXB [Source:HGNC Symbol;Acc:HGNC:7609]	-1.1884043
MYOZ3	myozenin 3 [Source:HGNC Symbol;Acc:HGNC:18565]	1.1289408
MYRIP	myosin VIIA and Rab interacting protein [Source:HGNC Symbol;Acc:HGNC:19156]	1.3019615
MZT1	mitotic spindle organizing protein 1 [Source:HGNC Symbol;Acc:HGNC:33830]	1.7463579
N4BP2L1	NEDD4 binding protein 2 like 1 [Source:HGNC Symbol;Acc:HGNC:25037]	-1.133873
N4BP3	NEDD4 binding protein 3 [Source:HGNC Symbol;Acc:HGNC:29852]	-1.2264096
NAA16	N(alpha)-acetyltransferase 16, NatA auxiliary subunit [Source:HGNC Symbol;Acc:HGNC:26164]	1.3773995
NAA30	N(alpha)-acetyltransferase 30, NatC catalytic subunit [Source:HGNC Symbol;Acc:HGNC:19844]	1.267436
NAA35	N(alpha)-acetyltransferase 35, NatC auxiliary subunit [Source:HGNC Symbol;Acc:HGNC:24340]	1.1721123
NAAA	N-acylethanolamine acid amidase [Source:HGNC Symbol;Acc:HGNC:736]	-1.089135
NAALAD2	N-acetylated alpha-linked acidic dipeptidase 2 [Source:HGNC Symbol;Acc:HGNC:14526]	1.0945653
NAALADL1	N-acetylated alpha-linked acidic dipeptidase-like 1 [Source:HGNC Symbol;Acc:HGNC:23536]	1.1361859
NAALADL2	N-acetylated alpha-linked acidic dipeptidase-like 2 [Source:HGNC Symbol;Acc:HGNC:23219]	-1.354432
NAB1	NGFI-A binding protein 1 [Source:HGNC Symbol;Acc:HGNC:7626]	-1.2834451
NACAD	NAC alpha domain containing [Source:HGNC Symbol;Acc:HGNC:22196]	-1.3009907
NADKD1	NAD kinase 2, mitochondrial	1.1158773
NANOS2	nanos C2HC-type zinc finger 2 [Source:HGNC Symbol;Acc:HGNC:23292]	1.0899192
NANS	N-acetylneuraminate synthase [Source:HGNC Symbol;Acc:HGNC:19237]	-1.0974611
NAP1L1	nucleosome assembly protein 1 like 1 [Source:HGNC Symbol;Acc:HGNC:7637]	1.4667228
NAP1L6	nucleosome assembly protein 1 like 6 [Source:HGNC Symbol;Acc:HGNC:31706]	1.1447695
NAPA	NSF attachment protein alpha [Source:HGNC Symbol;Acc:HGNC:7641]	1.3081883
NARS	asparaginyl-tRNA synthetase [Source:HGNC Symbol;Acc:HGNC:7643]	1.3537246
NAT8	N-acetyltransferase 8 (putative) [Source:HGNC Symbol;Acc:HGNC:18069]	1.1370331
NAT8L	N-acetyltransferase 8 like [Source:HGNC Symbol;Acc:HGNC:26742]	-1.574615
NAT9	N-acetyltransferase 9 (putative) [Source:HGNC Symbol;Acc:HGNC:23133]	-1.0752435
NAV2	neuron navigator 2 [Source:HGNC Symbol;Acc:HGNC:15997]	-1.7710113
NAV3	neuron navigator 3 [Source:HGNC Symbol;Acc:HGNC:15998]	-1.1614208
NCAM2	neural cell adhesion molecule 2 [Source:HGNC Symbol;Acc:HGNC:7657]	1.0656855
NCDN	neurochondrin [Source:HGNC Symbol;Acc:HGNC:17597]	1.3735135
NCF4	neutrophil cytosolic factor 4 [Source:HGNC Symbol;Acc:HGNC:7662]	-1.5492157
NCK2	NCK adaptor protein 2 [Source:HGNC Symbol;Acc:HGNC:7665]	-1.1271875
NCKAP1L	NCK associated protein 1 like [Source:HGNC Symbol;Acc:HGNC:4862]	-1.1438162
NCOA7	nuclear receptor coactivator 7 [Source:HGNC Symbol;Acc:HGNC:21081]	-4.234848
NCOR2	nuclear receptor corepressor 2 [Source:HGNC Symbol;Acc:HGNC:7673]	1.1294395
NCR1	natural cytotoxicity triggering receptor 1 [Source:HGNC Symbol;Acc:HGNC:6731]	1.1030093
NCR2	natural cytotoxicity triggering receptor 2 [Source:HGNC Symbol;Acc:HGNC:6732]	-1.1105155
NCR3	natural cytotoxicity triggering receptor 3 [Source:HGNC Symbol;Acc:HGNC:19077]	-1.0804781
NCR3LG1	natural killer cell cytotoxicity receptor 3 ligand 1	-1.0660846
NCRNA00029	long intergenic non-protein coding RNA 29	-1.1239451
NCRNA00087	small integral membrane protein 10 like 2B	-1.1497282
NCRNA00152	cytoskeleton regulator RNA	1.1411177
NCRNA00157	CHODL antisense RNA 1	1.0504012
NCRNA00158	long intergenic non-protein coding RNA 158	1.1174748
NCRNA00160	long intergenic non-protein coding RNA 160	-1.1556267
NCRNA00167	long intergenic non-protein coding RNA 167	1.1194732
NCRNA00219	EPB41L4A antisense RNA 1	1.0680128
NCRNA00238	long intergenic non-protein coding RNA 238	1.1053103
NCRNA00241	AGPAT4 intronic transcript 1	-1.3924394
NCRNA00244	long intergenic non-protein coding RNA 244	1.092499
NCRNA00251	long intergenic non-protein coding RNA 251	1.1129433
NCRNA00258	GRIK1 antisense RNA 2	1.1015751
NCRNA00266-1	long intergenic non-protein coding RNA 266-1	1.0630977
NCRNA00273	long intergenic non-protein coding RNA 273	-1.1872648
NCRNA00277	Ewing sarcoma associated transcript 1	1.1369424
NCRNA00282	long intergenic non-protein coding RNA 282	1.1505791
NCRNA00288	KIRREL3 antisense RNA 3	-1.1247498
NCRNA00304	long intergenic non-protein coding RNA 304	1.1437521
NCRNA00306	MIR7-3 host gene	1.1337112
NCRNA00311	long intergenic non-protein coding RNA 311	-1.0924194
NCRNA00314	long intergenic non-protein coding RNA 314	1.089246
NCRNA00317	long intergenic non-protein coding RNA 317	-1.2388884

NCRNA00324	long intergenic non-protein coding RNA 324	1.1205256
NCRNA00338	small nucleolar RNA host gene 20	1.1738718
NCRNA00339	long intergenic non-protein coding RNA 339	1.2126217
NCSTN	nicastrin [Source:HGNC Symbol;Acc:HGNC:17091]	-1.2289566
ND3	mitochondrially encoded NADH:ubiquinone oxidoreductase core subunit 3	-1.1356391
NDC80	NDC80, kinetochore complex component [Source:HGNC Symbol;Acc:HGNC:16909]	1.433804
NDEL1	nudE neurodevelopment protein 1 like 1 [Source:HGNC Symbol;Acc:HGNC:17620]	-1.2389675
NDNF	neuron derived neurotrophic factor [Source:HGNC Symbol;Acc:HGNC:26256]	1.0804515
NDST1	N-deacetylase and N-sulfotransferase 1 [Source:HGNC Symbol;Acc:HGNC:7680]	-1.300938
NDUFA10	NADH:ubiquinone oxidoreductase subunit A10 [Source:HGNC Symbol;Acc:HGNC:7684]	-1.1831264
NDUFA12	NADH:ubiquinone oxidoreductase subunit A12 [Source:HGNC Symbol;Acc:HGNC:23987]	-1.1322144
NDUFA4L2	NDUFA4, mitochondrial complex associated like 2 [Source:HGNC Symbol;Acc:HGNC:29836]	1.1521494
NDUFA7	NADH:ubiquinone oxidoreductase subunit A7 [Source:HGNC Symbol;Acc:HGNC:7691]	-1.1163362
NDUFA8	NADH:ubiquinone oxidoreductase subunit A8 [Source:HGNC Symbol;Acc:HGNC:7692]	1.069227
NDUFA9	NADH:ubiquinone oxidoreductase subunit A9 [Source:HGNC Symbol;Acc:HGNC:7693]	-1.1948994
NDUFAB1	NADH:ubiquinone oxidoreductase subunit AB1 [Source:HGNC Symbol;Acc:HGNC:7694]	1.0880563
NDUFAF2	NADH:ubiquinone oxidoreductase complex assembly factor 2 [Source:HGNC Symbol;Acc:HGNC:28086]	-1.1901318
NDUFAF4	NADH:ubiquinone oxidoreductase complex assembly factor 4 [Source:HGNC Symbol;Acc:HGNC:21034]	1.1776307
NDUFB8	NADH:ubiquinone oxidoreductase subunit B8 [Source:HGNC Symbol;Acc:HGNC:7703]	-1.1195259
NDUFC1	NADH:ubiquinone oxidoreductase subunit C1 [Source:HGNC Symbol;Acc:HGNC:7705]	-1.2224678
NDUFC2	NADH:ubiquinone oxidoreductase subunit C2 [Source:HGNC Symbol;Acc:HGNC:7706]	1.0590535
NEBL	nebulette [Source:HGNC Symbol;Acc:HGNC:16932]	1.1123013
NECAB2	N-terminal EF-hand calcium binding protein 2 [Source:HGNC Symbol;Acc:HGNC:23746]	-1.0600048
NECAP2	NECAP endocytosis associated 2 [Source:HGNC Symbol;Acc:HGNC:25528]	-1.5175261
NEDD4L	:ell expressed, developmentally down-regulated 4-like, E3 ubiquitin protein ligase [Source:HGNC Symbc	-1.2493476
NEDD9	:ural precursor cell expressed, developmentally down-regulated 9 [Source:HGNC Symbol;Acc:HGNC:773	-1.57465925
NEK6	NIMA related kinase 6 [Source:HGNC Symbol;Acc:HGNC:7749]	1.13452955
NEK7	NIMA related kinase 7 [Source:HGNC Symbol;Acc:HGNC:13386]	1.1503863
NES	nestin [Source:HGNC Symbol;Acc:HGNC:7756]	1.3966322
NET1	neuroepithelial cell transforming 1 [Source:HGNC Symbol;Acc:HGNC:14592]	1.3341147
NEURL	neuralized E3 ubiquitin protein ligase 1	-1.093151
NEURL1B	neuralized E3 ubiquitin protein ligase 1B [Source:HGNC Symbol;Acc:HGNC:35422]	1.1753443
NEURL3	neuralized E3 ubiquitin protein ligase 3 [Source:HGNC Symbol;Acc:HGNC:25162]	-2.2733395
NEUROG3	neurogenin 3 [Source:HGNC Symbol;Acc:HGNC:13806]	-1.0974045
NF2	neurofibromin 2 [Source:HGNC Symbol;Acc:HGNC:7773]	1.2619874
NFATC1	nuclear factor of activated T-cells 1 [Source:HGNC Symbol;Acc:HGNC:7775]	-1.5271399
NFATC2IP	nuclear factor of activated T-cells 2 interacting protein [Source:HGNC Symbol;Acc:HGNC:25906]	-1.2694521
NFE2L1	nuclear factor, erythroid 2 like 1 [Source:HGNC Symbol;Acc:HGNC:7781]	-1.3561966
NFIA	nuclear factor I A [Source:HGNC Symbol;Acc:HGNC:7784]	1.7764044
NFIB	nuclear factor I B [Source:HGNC Symbol;Acc:HGNC:7785]	1.6210234
NFIC	nuclear factor I C [Source:HGNC Symbol;Acc:HGNC:7786]	1.1761032
NFIL3	nuclear factor, interleukin 3 regulated [Source:HGNC Symbol;Acc:HGNC:7787]	-1.2569853
NFIX	nuclear factor I X [Source:HGNC Symbol;Acc:HGNC:7788]	-1.2861302
NFKB1	nuclear factor kappa B subunit 1 [Source:HGNC Symbol;Acc:HGNC:7794]	-2.8601785
NFKB2	nuclear factor kappa B subunit 2 [Source:HGNC Symbol;Acc:HGNC:7795]	-3.6775794
NFKBIA	NFKB inhibitor alpha [Source:HGNC Symbol;Acc:HGNC:7797]	-10.365542
NFKBIB	NFKB inhibitor beta [Source:HGNC Symbol;Acc:HGNC:7798]	-1.8789163
NFKBIE	NFKB inhibitor epsilon [Source:HGNC Symbol;Acc:HGNC:7799]	-4.569497
NFS1	NFS1 cysteine desulfurase [Source:HGNC Symbol;Acc:HGNC:15910]	1.0615354
NFXL1	nuclear transcription factor, X-box binding like 1 [Source:HGNC Symbol;Acc:HGNC:18726]	-1.2884429
NFYA	nuclear transcription factor Y subunit alpha [Source:HGNC Symbol;Acc:HGNC:7804]	1.2024946
NFYC	nuclear transcription factor Y subunit gamma [Source:HGNC Symbol;Acc:HGNC:7806]	-1.183952667
NHLH2	nescient helix-loop-helix 2 [Source:HGNC Symbol;Acc:HGNC:7818]	-1.1875988
NHS	NHS actin remodeling regulator [Source:HGNC Symbol;Acc:HGNC:7820]	1.1015651
NHSL1	NHS like 1 [Source:HGNC Symbol;Acc:HGNC:21021]	-1.1445942
NID2	nidogen 2 [Source:HGNC Symbol;Acc:HGNC:13389]	-1.3058165
NIF3L1	NGG1 interacting factor 3 like 1 [Source:HGNC Symbol;Acc:HGNC:13390]	1.1997259
NINJ1	ninjurin 1 [Source:HGNC Symbol;Acc:HGNC:7824]	-2.1408255
NINL	ninein like [Source:HGNC Symbol;Acc:HGNC:29163]	1.205701
NIPA1	non imprinted in Prader-Willi/Angelman syndrome 1 [Source:HGNC Symbol;Acc:HGNC:17043]	-1.3232436
NIPAL3	NIPA like domain containing 3 [Source:HGNC Symbol;Acc:HGNC:25233]	-1.1353283
NIPAL4	NIPA like domain containing 4 [Source:HGNC Symbol;Acc:HGNC:28018]	-1.2027171
NIPSNAP3A	nipsnap homolog 3A [Source:HGNC Symbol;Acc:HGNC:23619]	-1.2086691
NKD2	naked cuticle homolog 2 [Source:HGNC Symbol;Acc:HGNC:17046]	-2.2502365
NKIRAS1	NFKB inhibitor interacting Ras like 1 [Source:HGNC Symbol;Acc:HGNC:17899]	1.1220745
NKRF	NFKB repressing factor [Source:HGNC Symbol;Acc:HGNC:19374]	1.2072194
NKX3-1	NK3 homeobox 1 [Source:HGNC Symbol;Acc:HGNC:7838]	-1.7671115
NLGN2	neuroligin 2 [Source:HGNC Symbol;Acc:HGNC:14290]	1.2699687
NLGN4X	neuroligin 4, X-linked [Source:HGNC Symbol;Acc:HGNC:14287]	-1.0752696
NLRC3	NLR family CARD domain containing 3 [Source:HGNC Symbol;Acc:HGNC:29889]	1.6299596
NLRC4	NLR family CARD domain containing 4 [Source:HGNC Symbol;Acc:HGNC:16412]	1.0833808
NLRC5	NLR family CARD domain containing 5 [Source:HGNC Symbol;Acc:HGNC:29933]	-1.208081
NLRP1	NLR family pyrin domain containing 1 [Source:HGNC Symbol;Acc:HGNC:14374]	-1.1395012
NLRP12	NLR family pyrin domain containing 12 [Source:HGNC Symbol;Acc:HGNC:22938]	1.3526211
NLRP13	NLR family pyrin domain containing 13 [Source:HGNC Symbol;Acc:HGNC:22937]	1.0932368
NLRP3	NLR family pyrin domain containing 3 [Source:HGNC Symbol;Acc:HGNC:16400]	-1.5980512
NLRP5	NLR family pyrin domain containing 5 [Source:HGNC Symbol;Acc:HGNC:21269]	-1.2770007
NME4	NME/NM23 nucleoside diphosphate kinase 4 [Source:HGNC Symbol;Acc:HGNC:7852]	1.1492362
NME6	NME/NM23 nucleoside diphosphate kinase 6 [Source:HGNC Symbol;Acc:HGNC:20567]	-1.224104
NMNAT1	nicotinamide nucleotide adenylyltransferase 1 [Source:HGNC Symbol;Acc:HGNC:17877]	-1.0642712
NMUR2	neuromedin U receptor 2 [Source:HGNC Symbol;Acc:HGNC:16454]	-1.0657262
NOD1	nucleotide binding oligomerization domain containing 1 [Source:HGNC Symbol;Acc:HGNC:16390]	-1.1250986
NOD2	nucleotide binding oligomerization domain containing 2 [Source:HGNC Symbol;Acc:HGNC:5331]	-1.5108893
NOG	noggin [Source:HGNC Symbol;Acc:HGNC:7866]	1.353384
NOL10	nucleolar protein 10 [Source:HGNC Symbol;Acc:HGNC:25862]	1.1873175
NOL11	nucleolar protein 11 [Source:HGNC Symbol;Acc:HGNC:24557]	1.2506723

NOMO1	NODAL modulator 1 [Source:HGNC Symbol;Acc:HGNC:30060]	-1.1300565
NOP10	NOP10 ribonucleoprotein [Source:HGNC Symbol;Acc:HGNC:14378]	-1.0557715
NOP56	NOP56 ribonucleoprotein [Source:HGNC Symbol;Acc:HGNC:15911]	1.1831043
NOS3	nitric oxide synthase 3 [Source:HGNC Symbol;Acc:HGNC:7876]	1.6671364
NOTCH2	notch 2 [Source:HGNC Symbol;Acc:HGNC:7882]	-1.6685209
NOTCH2NL	notch 2 N-terminal like [Source:HGNC Symbol;Acc:HGNC:31862]	-1.3911723
NOV	nephroblastoma overexpressed [Source:HGNC Symbol;Acc:HGNC:7885]	-1.3349334
NOX4	NADPH oxidase 4 [Source:HGNC Symbol;Acc:HGNC:7891]	1.5578209
NPAS2	neuronal PAS domain protein 2 [Source:HGNC Symbol;Acc:HGNC:7895]	1.2163064
NPAT	nuclear protein, coactivator of histone transcription [Source:HGNC Symbol;Acc:HGNC:7896]	1.294044
NPBWR1	neuropeptides B/W receptor 1 [Source:HGNC Symbol;Acc:HGNC:4522]	-1.1682034
NPC2	NPC intracellular cholesterol transporter 2 [Source:HGNC Symbol;Acc:HGNC:14537]	-1.1062347
NPDC1	neural proliferation, differentiation and control 1 [Source:HGNC Symbol;Acc:HGNC:7899]	1.2665823
NPFFR2	neuropeptide FF receptor 2 [Source:HGNC Symbol;Acc:HGNC:4525]	-1.0648376
NPIPL3	nuclear pore complex interacting protein family member B3	1.0685937
NPL	N-acetylneuraminate pyruvate lyase [Source:HGNC Symbol;Acc:HGNC:16781]	-1.1143938
NPLOC4	NPL4 homolog, ubiquitin recognition factor [Source:HGNC Symbol;Acc:HGNC:18261]	-1.3084962
NPM1	nucleophosmin [Source:HGNC Symbol;Acc:HGNC:7910]	1.3983469
NPR2	natriuretic peptide receptor 2 [Source:HGNC Symbol;Acc:HGNC:7944]	1.1408908
NPY1R	neuropeptide Y receptor Y1 [Source:HGNC Symbol;Acc:HGNC:7956]	1.0789124
NR1D2	nuclear receptor subfamily 1 group D member 2 [Source:HGNC Symbol;Acc:HGNC:7963]	1.1156998
NR1I2	nuclear receptor subfamily 1 group I member 2 [Source:HGNC Symbol;Acc:HGNC:7968]	1.0594279
NR1I3	nuclear receptor subfamily 1 group I member 3 [Source:HGNC Symbol;Acc:HGNC:7969]	1.0902568
NR2F2	nuclear receptor subfamily 2 group F member 2 [Source:HGNC Symbol;Acc:HGNC:7976]	1.9012372
NR2F6	nuclear receptor subfamily 2 group F member 6 [Source:HGNC Symbol;Acc:HGNC:7977]	1.3242253
NR3C1	nuclear receptor subfamily 3 group C member 1 [Source:HGNC Symbol;Acc:HGNC:7978]	1.408674
NR3C2	nuclear receptor subfamily 3 group C member 2 [Source:HGNC Symbol;Acc:HGNC:7979]	1.5998374
NR4A3	nuclear receptor subfamily 4 group A member 3 [Source:HGNC Symbol;Acc:HGNC:7982]	-2.3759637
NR5A2	nuclear receptor subfamily 5 group A member 2 [Source:HGNC Symbol;Acc:HGNC:7984]	1.6139132
NRG1	neuregulin 1 [Source:HGNC Symbol;Acc:HGNC:7997]	-1.27745355
NRIP2	nuclear receptor interacting protein 2 [Source:HGNC Symbol;Acc:HGNC:23078]	-1.1722987
NRK	Nik related kinase [Source:HGNC Symbol;Acc:HGNC:25391]	1.0912156
NRN1	neuritin 1 [Source:HGNC Symbol;Acc:HGNC:17972]	1.1884468
NRP1	neuropilin 1 [Source:HGNC Symbol;Acc:HGNC:8004]	1.5257566
NRP2	neuropilin 2 [Source:HGNC Symbol;Acc:HGNC:8005]	-1.275035
NRXN1	neurexin 1 [Source:HGNC Symbol;Acc:HGNC:8008]	1.0697949
NSA2	NSA2, ribosome biogenesis homolog [Source:HGNC Symbol;Acc:HGNC:30728]	1.3082967
NSD1	nuclear receptor binding SET domain protein 1 [Source:HGNC Symbol;Acc:HGNC:14234]	1.1261767
NSDHL	NAD(P) dependent steroid dehydrogenase-like [Source:HGNC Symbol;Acc:HGNC:13398]	1.1661164
NSUN3	NOP2/Sun RNA methyltransferase family member 3 [Source:HGNC Symbol;Acc:HGNC:26208]	1.1336774
NSUN5	NOP2/Sun RNA methyltransferase family member 5 [Source:HGNC Symbol;Acc:HGNC:16385]	1.1397355
NT5DC4	5'-nucleotidase domain containing 4 [Source:HGNC Symbol;Acc:HGNC:27678]	1.1212589
NTHL1	nth like DNA glycosylase 1 [Source:HGNC Symbol;Acc:HGNC:8028]	1.1211994
NTN1	netrin 1 [Source:HGNC Symbol;Acc:HGNC:8029]	-1.4923034
NTN4	netrin 4 [Source:HGNC Symbol;Acc:HGNC:13658]	1.381472
NTNG2	netrin G2 [Source:HGNC Symbol;Acc:HGNC:14288]	-1.0913815
NTSR1	neurotensin receptor 1 (high affinity) [Source:HGNC Symbol;Acc:HGNC:8039]	1.1732681
NUAK1	NUAK family kinase 1 [Source:HGNC Symbol;Acc:HGNC:14311]	1.4934733
NUAK2	NUAK family kinase 2 [Source:HGNC Symbol;Acc:HGNC:29558]	-9.072099
NUCKS1	nuclear casein kinase and cyclin dependent kinase substrate 1 [Source:HGNC Symbol;Acc:HGNC:29923]	1.1896721
NUDCD2	NudC domain containing 2 [Source:HGNC Symbol;Acc:HGNC:30535]	1.1813283
NUDT10	nudix hydrolase 10 [Source:HGNC Symbol;Acc:HGNC:17621]	-1.1273559
NUDT16	nudix hydrolase 16 [Source:HGNC Symbol;Acc:HGNC:26442]	-1.1129522
NUDT21	nudix hydrolase 21 [Source:HGNC Symbol;Acc:HGNC:13870]	1.5302132
NUDT3	nudix hydrolase 3 [Source:HGNC Symbol;Acc:HGNC:8050]	-1.2032771
NUDT6	nudix hydrolase 6 [Source:HGNC Symbol;Acc:HGNC:8053]	1.4160154
NUMA1	nuclear mitotic apparatus protein 1 [Source:HGNC Symbol;Acc:HGNC:8059]	1.1658032
NUMBL	NUMB like, endocytic adaptor protein [Source:HGNC Symbol;Acc:HGNC:8061]	-1.1770153
NUP205	nucleoporin 205 [Source:HGNC Symbol;Acc:HGNC:18658]	1.3083394
NUP62	nucleoporin 62 [Source:HGNC Symbol;Acc:HGNC:8066]	-1.46466225
NUTF2	nuclear transport factor 2 [Source:HGNC Symbol;Acc:HGNC:13722]	1.1316485
NXF1	nuclear RNA export factor 1 [Source:HGNC Symbol;Acc:HGNC:8071]	-1.2153399
NXPH4	neurexophilin 4 [Source:HGNC Symbol;Acc:HGNC:8078]	-1.1428909
NYNRIN	NYN domain and retroviral integrase containing [Source:HGNC Symbol;Acc:HGNC:20165]	1.18795335
O3FAR1	free fatty acid receptor 4	-1.1809589
OAF	out at first homolog [Source:HGNC Symbol;Acc:HGNC:28752]	1.177142
OAS2	2'-5'-oligoadenylate synthetase 2 [Source:HGNC Symbol;Acc:HGNC:8087]	-1.15461685
OAS3	2'-5'-oligoadenylate synthetase 3 [Source:HGNC Symbol;Acc:HGNC:8088]	-1.29767705
OASL	2'-5'-oligoadenylate synthetase like [Source:HGNC Symbol;Acc:HGNC:8090]	-1.2026701
OBFC1	oligonucleotide/oligosaccharide binding fold containing 1 [Source:HGNC Symbol;Acc:HGNC:26200]	-1.2197133
OBFC2A	nucleic acid binding protein 1	-1.3853759
OBFC2B	nucleic acid binding protein 2	1.2630414
OCLN	occludin [Source:HGNC Symbol;Acc:HGNC:8104]	1.0882439
OCM2	oncomodulin 2 [Source:HGNC Symbol;Acc:HGNC:34396]	-1.1395899
ODF2	outer dense fiber of sperm tails 2 [Source:HGNC Symbol;Acc:HGNC:8114]	-1.2228826
ODF2L	outer dense fiber of sperm tails 2 like [Source:HGNC Symbol;Acc:HGNC:29225]	1.0953338
ODF3	outer dense fiber of sperm tails 3 [Source:HGNC Symbol;Acc:HGNC:19905]	-1.1364433
ODF3L2	outer dense fiber of sperm tails 3 like 2 [Source:HGNC Symbol;Acc:HGNC:26841]	-1.3302294
ODF4	outer dense fiber of sperm tails 4 [Source:HGNC Symbol;Acc:HGNC:19056]	-1.1083764
ODZ3	teneurin transmembrane protein 3	-1.1195648
OGFR	opioid growth factor receptor [Source:HGNC Symbol;Acc:HGNC:15768]	-1.4049805
OGFRL1	opioid growth factor receptor like 1 [Source:HGNC Symbol;Acc:HGNC:21378]	-1.151595
OLFML2A	olfactomedin like 2A [Source:HGNC Symbol;Acc:HGNC:27270]	-1.168296
OLFML2B	olfactomedin like 2B [Source:HGNC Symbol;Acc:HGNC:24558]	-1.1166612
OLIG1	oligodendrocyte transcription factor 1 [Source:HGNC Symbol;Acc:HGNC:16983]	1.1477231
OLIG3	oligodendrocyte transcription factor 3 [Source:HGNC Symbol;Acc:HGNC:18003]	1.0989375

ONECUT2	one cut homeobox 2 [Source:HGNC Symbol;Acc:HGNC:8139]	-1.131913
OOSP1	oocyte secreted protein 1, pseudogene [Source:HGNC Symbol;Acc:HGNC:49233]	-1.0635871
OPCML	opioid binding protein/cell adhesion molecule like [Source:HGNC Symbol;Acc:HGNC:8143]	1.0586793
OPN1SW	opsin 1 (cone pigments), short-wave-sensitive [Source:HGNC Symbol;Acc:HGNC:1012]	-1.1097462
OPRL1	opioid related nociceptin receptor 1 [Source:HGNC Symbol;Acc:HGNC:8155]	-1.125468
OPTN	optineurin [Source:HGNC Symbol;Acc:HGNC:17142]	-1.7759129
OR10G2	olfactory receptor family 10 subfamily G member 2 [Source:HGNC Symbol;Acc:HGNC:8170]	-1.4409568
OR10H1	olfactory receptor family 10 subfamily H member 1 [Source:HGNC Symbol;Acc:HGNC:8172]	-1.2127231
OR10H4	olfactory receptor family 10 subfamily H member 4 [Source:HGNC Symbol;Acc:HGNC:15388]	-1.1180928
OR10H5	olfactory receptor family 10 subfamily H member 5 [Source:HGNC Symbol;Acc:HGNC:15389]	1.0999346
OR10J5	olfactory receptor family 10 subfamily J member 5 [Source:HGNC Symbol;Acc:HGNC:14993]	-1.099858
OR11H12	olfactory receptor family 11 subfamily H member 12 [Source:HGNC Symbol;Acc:HGNC:30738]	-1.0969833
OR13C4	olfactory receptor family 13 subfamily C member 4 [Source:HGNC Symbol;Acc:HGNC:14722]	1.095171
OR13C8	olfactory receptor family 13 subfamily C member 8 [Source:HGNC Symbol;Acc:HGNC:15103]	1.1107129
OR13D1	olfactory receptor family 13 subfamily D member 1 [Source:HGNC Symbol;Acc:HGNC:14695]	-1.1652844
OR13H1	olfactory receptor family 13 subfamily H member 1 [Source:HGNC Symbol;Acc:HGNC:14755]	-1.3002077
OR14I1	olfactory receptor family 14 subfamily I member 1 [Source:HGNC Symbol;Acc:HGNC:19575]	1.0775034
OR14J1	olfactory receptor family 14 subfamily J member 1 [Source:HGNC Symbol;Acc:HGNC:13971]	1.1773582
OR1A1	olfactory receptor family 1 subfamily A member 1 [Source:HGNC Symbol;Acc:HGNC:8179]	1.0743848
OR1F2P	olfactory receptor family 1 subfamily F member 2 pseudogene [Source:HGNC Symbol;Acc:HGNC:8196]	-1.0862782
OR1J4	olfactory receptor family 1 subfamily J member 4 [Source:HGNC Symbol;Acc:HGNC:8211]	-1.2237248
OR1N2	olfactory receptor family 1 subfamily N member 2 [Source:HGNC Symbol;Acc:HGNC:15111]	-1.0834854
OR2A12	olfactory receptor family 2 subfamily A member 12 [Source:HGNC Symbol;Acc:HGNC:15082]	1.125259
OR2A20P	ılfactory receptor family 2 subfamily A member 20 pseudogene [Source:HGNC Symbol;Acc:HGNC:15413	-1.1669725
OR2A25	olfactory receptor family 2 subfamily A member 25 [Source:HGNC Symbol;Acc:HGNC:19562]	-1.1627123
OR2A9P	olfactory receptor family 2 subfamily A member 9 pseudogene [Source:HGNC Symbol;Acc:HGNC:8236]	-1.0777371
OR2AG2	olfactory receptor family 2 subfamily AG member 2 [Source:HGNC Symbol;Acc:HGNC:15143]	-1.1758953
OR2C3	olfactory receptor family 2 subfamily C member 3 [Source:HGNC Symbol;Acc:HGNC:15005]	-1.075338
OR2H1	olfactory receptor family 2 subfamily H member 1 [Source:HGNC Symbol;Acc:HGNC:8252]	1.0919843
OR2J3	olfactory receptor family 2 subfamily J member 3 [Source:HGNC Symbol;Acc:HGNC:8261]	1.1759146
OR2L8	ctory receptor family 2 subfamily L member 8 (gene/pseudogene) [Source:HGNC Symbol;Acc:HGNC:15(	-1.0578525
OR2M3	olfactory receptor family 2 subfamily M member 3 [Source:HGNC Symbol;Acc:HGNC:8269]	1.0811707
OR2T27	olfactory receptor family 2 subfamily T member 27 [Source:HGNC Symbol;Acc:HGNC:31252]	-1.1447225
OR2T4	olfactory receptor family 2 subfamily T member 4 [Source:HGNC Symbol;Acc:HGNC:15016]	1.0820304
OR2W3	olfactory receptor family 2 subfamily W member 3 [Source:HGNC Symbol;Acc:HGNC:15021]	-1.0579541
OR2Y1	olfactory receptor family 2 subfamily Y member 1 [Source:HGNC Symbol;Acc:HGNC:14837]	1.159105
OR4A15	olfactory receptor family 4 subfamily A member 15 [Source:HGNC Symbol;Acc:HGNC:15152]	-1.1634454
OR4A47	olfactory receptor family 4 subfamily A member 47 [Source:HGNC Symbol;Acc:HGNC:31266]	1.1397042
OR4D10	olfactory receptor family 4 subfamily D member 10 [Source:HGNC Symbol;Acc:HGNC:15173]	-1.0963695
OR4D11	olfactory receptor family 4 subfamily D member 11 [Source:HGNC Symbol;Acc:HGNC:15174]	-1.1159046
OR4D2	olfactory receptor family 4 subfamily D member 2 [Source:HGNC Symbol;Acc:HGNC:8294]	1.1204609
OR4D6	olfactory receptor family 4 subfamily D member 6 [Source:HGNC Symbol;Acc:HGNC:15175]	-1.1604722
OR4D9	olfactory receptor family 4 subfamily D member 9 [Source:HGNC Symbol;Acc:HGNC:15178]	1.1283741
OR4F21	olfactory receptor family 4 subfamily F member 21 [Source:HGNC Symbol;Acc:HGNC:19583]	1.082412
OR4K13	olfactory receptor family 4 subfamily K member 13 [Source:HGNC Symbol;Acc:HGNC:15351]	1.0750698
OR4K17	olfactory receptor family 4 subfamily K member 17 [Source:HGNC Symbol;Acc:HGNC:15355]	1.101651
OR4N2	olfactory receptor family 4 subfamily N member 2 [Source:HGNC Symbol;Acc:HGNC:14742]	1.0925088
OR4N3P	olfactory receptor family 4 subfamily N member 3 pseudogene [Source:HGNC Symbol;Acc:HGNC:15374	1.0756279
OR51B2	ctory receptor family 51 subfamily B member 2 (gene/pseudogene) [Source:HGNC Symbol;Acc:HGNC:14	1.075773
OR51B4	olfactory receptor family 51 subfamily B member 4 [Source:HGNC Symbol;Acc:HGNC:14708]	1.1831416
OR51B5	olfactory receptor family 51 subfamily B member 5 [Source:HGNC Symbol;Acc:HGNC:19599]	-1.0741031
OR52A1	olfactory receptor family 52 subfamily A member 1 [Source:HGNC Symbol;Acc:HGNC:8318]	-1.1370674
OR52B2	olfactory receptor family 52 subfamily B member 2 [Source:HGNC Symbol;Acc:HGNC:15207]	-1.156535
OR52L1	olfactory receptor family 52 subfamily L member 1 [Source:HGNC Symbol;Acc:HGNC:14785]	1.119468
OR56A3	olfactory receptor family 56 subfamily A member 3 [Source:HGNC Symbol;Acc:HGNC:14786]	1.0653118
OR56A4	olfactory receptor family 56 subfamily A member 4 [Source:HGNC Symbol;Acc:HGNC:14791]	-1.1008959
OR56A5	olfactory receptor family 56 subfamily A member 5 [Source:HGNC Symbol;Acc:HGNC:14792]	1.1346315
OR5A1	olfactory receptor family 5 subfamily A member 1 [Source:HGNC Symbol;Acc:HGNC:8319]	-1.2393959
OR5AS1	olfactory receptor family 5 subfamily AS member 1 [Source:HGNC Symbol;Acc:HGNC:15261]	-1.1433871
OR5AU1	olfactory receptor family 5 subfamily AU member 1 [Source:HGNC Symbol;Acc:HGNC:15362]	1.0851042
OR5C1	olfactory receptor family 5 subfamily C member 1 [Source:HGNC Symbol;Acc:HGNC:8331]	-1.1889173
OR5D14	olfactory receptor family 5 subfamily D member 14 [Source:HGNC Symbol;Acc:HGNC:15281]	1.1983411
OR5D16	olfactory receptor family 5 subfamily D member 16 [Source:HGNC Symbol;Acc:HGNC:15283]	-1.1016698
OR5D18	olfactory receptor family 5 subfamily D member 18 [Source:HGNC Symbol;Acc:HGNC:15285]	1.1530051
OR5H1	olfactory receptor family 5 subfamily H member 1 [Source:HGNC Symbol;Acc:HGNC:8346]	-1.3835421
OR5V1	olfactory receptor family 5 subfamily V member 1 [Source:HGNC Symbol;Acc:HGNC:13972]	-1.1541651
OR6B1	olfactory receptor family 6 subfamily B member 1 [Source:HGNC Symbol;Acc:HGNC:8354]	1.1885264
OR6K6	olfactory receptor family 6 subfamily K member 6 [Source:HGNC Symbol;Acc:HGNC:15033]	1.1597985
OR7C2	olfactory receptor family 7 subfamily C member 2 [Source:HGNC Symbol;Acc:HGNC:8374]	-1.1190263
OR7E13P	olfactory receptor family 7 subfamily E member 13 pseudogene [Source:HGNC Symbol;Acc:HGNC:8384]	-1.1991323
OR7E156P	lfactory receptor family 7 subfamily E member 156 pseudogene [Source:HGNC Symbol;Acc:HGNC:3131	-1.2184136
OR7E19P	olfactory receptor family 7 subfamily E member 19 pseudogene [Source:HGNC Symbol;Acc:HGNC:8390]	1.483685
OR7E5P	olfactory receptor family 7 subfamily E member 5 pseudogene [Source:HGNC Symbol;Acc:HGNC:8435]	-1.1359444
OR7E91P	olfactory receptor family 7 subfamily E member 91 pseudogene [Source:HGNC Symbol;Acc:HGNC:14747	-1.220694
OR7G2	olfactory receptor family 7 subfamily G member 2 [Source:HGNC Symbol;Acc:HGNC:8466]	-1.0777601
OR8K3	ctory receptor family 8 subfamily K member 3 (gene/pseudogene) [Source:HGNC Symbol;Acc:HGNC:15	-1.1767447
ORAOV1	oral cancer overexpressed 1 [Source:HGNC Symbol;Acc:HGNC:17589]	-1.10873945
ORC1	origin recognition complex subunit 1 [Source:HGNC Symbol;Acc:HGNC:8487]	1.2800539
ORC4	origin recognition complex subunit 4 [Source:HGNC Symbol;Acc:HGNC:8490]	1.3102055
ORC5	origin recognition complex subunit 5 [Source:HGNC Symbol;Acc:HGNC:8491]	1.2457291
ORC6	origin recognition complex subunit 6 [Source:HGNC Symbol;Acc:HGNC:17151]	1.2005365
OSBPL11	oxysterol binding protein like 11 [Source:HGNC Symbol;Acc:HGNC:16397]	1.2359545
OSBPL2	oxysterol binding protein like 2 [Source:HGNC Symbol;Acc:HGNC:15761]	-1.0756029
OSBPL3	oxysterol binding protein like 3 [Source:HGNC Symbol;Acc:HGNC:16370]	-1.4310619
OSBPL6	oxysterol binding protein like 6 [Source:HGNC Symbol;Acc:HGNC:16388]	1.2975129
OSBPL7	oxysterol binding protein like 7 [Source:HGNC Symbol;Acc:HGNC:16387]	1.4192607

OSBPL9	oxysterol binding protein like 9 [Source:HGNC Symbol;Acc:HGNC:16386]	1.2930473
OSGEPL1	O-sialoglycoprotein endopeptidase-like 1 [Source:HGNC Symbol;Acc:HGNC:23075]	1.323539
OSGIN1	oxidative stress induced growth inhibitor 1 [Source:HGNC Symbol;Acc:HGNC:30093]	-1.225699
OSTN	osteocrin [Source:HGNC Symbol;Acc:HGNC:29961]	1.0821339
OTOG	otogelin [Source:HGNC Symbol;Acc:HGNC:8516]	-1.18891365
OTUD4	OTU deubiquitinase 4 [Source:HGNC Symbol;Acc:HGNC:24949]	-1.151607
OXER1	oxoeicosanoid receptor 1 [Source:HGNC Symbol;Acc:HGNC:24884]	-1.3758495
P2RX4	purinergic receptor P2X 4 [Source:HGNC Symbol;Acc:HGNC:8535]	-1.4819273
P2RX7	purinergic receptor P2X 7 [Source:HGNC Symbol;Acc:HGNC:8537]	1.1682496
P2RY12	purinergic receptor P2Y12 [Source:HGNC Symbol;Acc:HGNC:18124]	1.0663192
P2RY6	pyrimidinergic receptor P2Y6 [Source:HGNC Symbol;Acc:HGNC:8543]	-1.5899575
P4HA1	prolyl 4-hydroxylase subunit alpha 1 [Source:HGNC Symbol;Acc:HGNC:8546]	1.1597677
P4HA2	prolyl 4-hydroxylase subunit alpha 2 [Source:HGNC Symbol;Acc:HGNC:8547]	-1.2146702
P4HB	prolyl 4-hydroxylase subunit beta [Source:HGNC Symbol;Acc:HGNC:8548]	-1.0994105
PAAF1	proteasomal ATPase associated factor 1 [Source:HGNC Symbol;Acc:HGNC:25687]	1.0917611
PACSIN2	protein kinase C and casein kinase substrate in neurons 2 [Source:HGNC Symbol;Acc:HGNC:8571]	-1.16357605
PADI2	peptidyl arginine deiminase 2 [Source:HGNC Symbol;Acc:HGNC:18341]	-1.1434999
PAK2	p21 (RAC1) activated kinase 2 [Source:HGNC Symbol;Acc:HGNC:8591]	1.2382716
PALB2	partner and localizer of BRCA2 [Source:HGNC Symbol;Acc:HGNC:26144]	1.2662883
PALLD	palladin, cytoskeletal associated protein [Source:HGNC Symbol;Acc:HGNC:17068]	-1.118894
PALMD	palmdelphin [Source:HGNC Symbol;Acc:HGNC:15846]	1.5529926
PANX1	pannexin 1 [Source:HGNC Symbol;Acc:HGNC:8599]	-2.2744923
PAOX	polyamine oxidase (exo-N4-amino) [Source:HGNC Symbol;Acc:HGNC:20837]	-1.2730788
PAPLN	papilin, proteoglycan like sulfated glycoprotein [Source:HGNC Symbol;Acc:HGNC:19262]	-2.5828102
PAPOLB	poly(A) polymerase beta [Source:HGNC Symbol;Acc:HGNC:15970]	1.0942662
PARD3B	par-3 family cell polarity regulator beta [Source:HGNC Symbol;Acc:HGNC:14446]	1.0533988
PARL	presenilin associated rhomboid like [Source:HGNC Symbol;Acc:HGNC:18253]	-1.1298854
PARP10	poly(ADP-ribose) polymerase family member 10 [Source:HGNC Symbol;Acc:HGNC:25895]	-1.65136
PARP11	poly(ADP-ribose) polymerase family member 11 [Source:HGNC Symbol;Acc:HGNC:1186]	1.0727016
PARP12	poly(ADP-ribose) polymerase family member 12 [Source:HGNC Symbol;Acc:HGNC:21919]	-1.4553648
PARP16	poly(ADP-ribose) polymerase family member 16 [Source:HGNC Symbol;Acc:HGNC:26040]	1.3805369
PARP4	poly(ADP-ribose) polymerase family member 4 [Source:HGNC Symbol;Acc:HGNC:271]	1.21597
PARP6	poly(ADP-ribose) polymerase family member 6 [Source:HGNC Symbol;Acc:HGNC:26921]	-1.1866733
PARP9	poly(ADP-ribose) polymerase family member 9 [Source:HGNC Symbol;Acc:HGNC:24118]	-1.2191552
PARVG	parvin gamma [Source:HGNC Symbol;Acc:HGNC:14654]	-1.0633644
PATL1	PAT1 homolog 1, processing body mRNA decay factor [Source:HGNC Symbol;Acc:HGNC:26721]	-1.4273281
PATZ1	POZ/BTB and AT hook containing zinc finger 1 [Source:HGNC Symbol;Acc:HGNC:13071]	1.1834778
PAX3	paired box 3 [Source:HGNC Symbol;Acc:HGNC:8617]	1.1452516
PAX6	paired box 6 [Source:HGNC Symbol;Acc:HGNC:8620]	1.09244795
PAX9	paired box 9 [Source:HGNC Symbol;Acc:HGNC:8623]	1.350595
PBK	PDZ binding kinase [Source:HGNC Symbol;Acc:HGNC:18282]	1.5030258
PBOV1	prostate and breast cancer overexpressed 1 [Source:HGNC Symbol;Acc:HGNC:21079]	-1.1321723
PBXIP1	PBX homeobox interacting protein 1 [Source:HGNC Symbol;Acc:HGNC:21199]	1.115023
PCA3	prostate cancer associated 3 (non-protein coding) [Source:HGNC Symbol;Acc:HGNC:8637]	1.0953386
PCBD2	pterin-4 alpha-carbinolamine dehydratase 2 [Source:HGNC Symbol;Acc:HGNC:24474]	-1.2245479
PCCA	propionyl-CoA carboxylase alpha subunit [Source:HGNC Symbol;Acc:HGNC:8653]	1.1915717
PCDH12	protocadherin 12 [Source:HGNC Symbol;Acc:HGNC:8657]	-1.5214883
PCDH7	protocadherin 7 [Source:HGNC Symbol;Acc:HGNC:8659]	1.4556979
PCDH9	protocadherin 9 [Source:HGNC Symbol;Acc:HGNC:8661]	-1.0827903
PCDHA1	protocadherin alpha 1 [Source:HGNC Symbol;Acc:HGNC:8663]	1.0758625
PCDHA2	protocadherin alpha 2 [Source:HGNC Symbol;Acc:HGNC:8668]	1.068006
PCDHA3	protocadherin alpha 3 [Source:HGNC Symbol;Acc:HGNC:8669]	1.0938882
PCDHA5	protocadherin alpha 5 [Source:HGNC Symbol;Acc:HGNC:8671]	-1.391379
PCDHB11	protocadherin beta 11 [Source:HGNC Symbol;Acc:HGNC:8682]	-1.1345234
PCDHB14	protocadherin beta 14 [Source:HGNC Symbol;Acc:HGNC:8685]	-1.1506243
PCDHGA12	protocadherin gamma subfamily A, 12 [Source:HGNC Symbol;Acc:HGNC:8699]	-1.1868048
PCDHGA5	protocadherin gamma subfamily A, 5 [Source:HGNC Symbol;Acc:HGNC:8703]	1.0533369
PCDHGA8	protocadherin gamma subfamily A, 8 [Source:HGNC Symbol;Acc:HGNC:8706]	-1.2446334
PCDHGB7	protocadherin gamma subfamily B, 7 [Source:HGNC Symbol;Acc:HGNC:8714]	-1.1806957
PCDHGC4	protocadherin gamma subfamily C, 4 [Source:HGNC Symbol;Acc:HGNC:8717]	-1.1518114
PCGF5	polycomb group ring finger 5 [Source:HGNC Symbol;Acc:HGNC:28264]	-1.2417965
PCLO	piccolo presynaptic cytomatrix protein [Source:HGNC Symbol;Acc:HGNC:13406]	1.116391
PCMT1	protein-L-isoaspartate (D-aspartate) O-methyltransferase [Source:HGNC Symbol;Acc:HGNC:8728]	-1.1004567
PCNP	PEST proteolytic signal containing nuclear protein [Source:HGNC Symbol;Acc:HGNC:30023]	1.3844329
PCNX	pecanex homolog 1 (Drosophila)	-1.2184458
PCP4	Purkinje cell protein 4 [Source:HGNC Symbol;Acc:HGNC:8742]	-1.0652758
PCSK5	proprotein convertase subtilisin/kexin type 5 [Source:HGNC Symbol;Acc:HGNC:8747]	1.1679882
PCTP	phosphatidylcholine transfer protein [Source:HGNC Symbol;Acc:HGNC:8752]	1.1647224
PCYT1A	phosphate cytidylyltransferase 1, choline, alpha [Source:HGNC Symbol;Acc:HGNC:8754]	-1.3517264
PDAP1	PDGFA associated protein 1 [Source:HGNC Symbol;Acc:HGNC:14634]	-1.140192
PDCD1LG2	programmed cell death 1 ligand 2 [Source:HGNC Symbol;Acc:HGNC:18731]	-1.1713042
PDCD2	programmed cell death 2 [Source:HGNC Symbol;Acc:HGNC:8762]	1.0717789
PDCD7	programmed cell death 7 [Source:HGNC Symbol;Acc:HGNC:8767]	1.1732742
PDCL	phosducin like [Source:HGNC Symbol;Acc:HGNC:8770]	1.4813218
PDDC1	Parkinson disease 7 domain containing 1 [Source:HGNC Symbol;Acc:HGNC:26616]	-1.1347449
PDE1A	phosphodiesterase 1A [Source:HGNC Symbol;Acc:HGNC:8774]	-1.111751
PDE4B	phosphodiesterase 4B [Source:HGNC Symbol;Acc:HGNC:8781]	-1.3457139
PDE5A	phosphodiesterase 5A [Source:HGNC Symbol;Acc:HGNC:8784]	-1.3351357
PDE6D	phosphodiesterase 6D [Source:HGNC Symbol;Acc:HGNC:8788]	-1.2344568
PDE7B	phosphodiesterase 7B [Source:HGNC Symbol;Acc:HGNC:8792]	1.9053061
PDE9A	phosphodiesterase 9A [Source:HGNC Symbol;Acc:HGNC:8795]	-1.2880346
PDGFB	platelet derived growth factor subunit B [Source:HGNC Symbol;Acc:HGNC:8800]	-1.393895
PDGFC	platelet derived growth factor C [Source:HGNC Symbol;Acc:HGNC:8801]	-1.2014569
PDGFRL	platelet derived growth factor receptor like [Source:HGNC Symbol;Acc:HGNC:8805]	-1.7485782
PDHA1	pyruvate dehydrogenase (lipoamide) alpha 1 [Source:HGNC Symbol;Acc:HGNC:8806]	-1.2505089
PDIA4	protein disulfide isomerase family A member 4 [Source:HGNC Symbol;Acc:HGNC:30167]	1.2604866

PDIA5	protein disulfide isomerase family A member 5 [Source:HGNC Symbol;Acc:HGNC:24811]	-1.1073412
PDK3	pyruvate dehydrogenase kinase 3 [Source:HGNC Symbol;Acc:HGNC:8811]	-1.1541294
PDK4	pyruvate dehydrogenase kinase 4 [Source:HGNC Symbol;Acc:HGNC:8812]	-1.1843957
PDLIM1	PDZ and LIM domain 1 [Source:HGNC Symbol;Acc:HGNC:2067]	-1.2064899
PDLIM4	PDZ and LIM domain 4 [Source:HGNC Symbol;Acc:HGNC:16501]	-1.9695991
PDLIM5	PDZ and LIM domain 5 [Source:HGNC Symbol;Acc:HGNC:17468]	-1.0868515
PDLIM7	PDZ and LIM domain 7 [Source:HGNC Symbol;Acc:HGNC:22958]	1.1619644
PDRG1	p53 and DNA damage regulated 1 [Source:HGNC Symbol;Acc:HGNC:16119]	-1.1708859
PDXP	pyridoxal phosphatase [Source:HGNC Symbol;Acc:HGNC:30259]	1.5862558
PDZD11	PDZ domain containing 11 [Source:HGNC Symbol;Acc:HGNC:28034]	-1.16477365
PDZD2	PDZ domain containing 2 [Source:HGNC Symbol;Acc:HGNC:18486]	-2.5348952
PEA15	phosphoprotein enriched in astrocytes 15 [Source:HGNC Symbol;Acc:HGNC:8822]	1.15223065
PEBP1	phosphatidylethanolamine binding protein 1 [Source:HGNC Symbol;Acc:HGNC:8630]	1.0957117
PEF1	penta-EF-hand domain containing 1 [Source:HGNC Symbol;Acc:HGNC:30009]	-1.0971975
PEG3	paternally expressed 3 [Source:HGNC Symbol;Acc:HGNC:8826]	1.1355479
PELI2	pellino E3 ubiquitin protein ligase family member 2 [Source:HGNC Symbol;Acc:HGNC:8828]	1.5983356
PEPD	peptidase D [Source:HGNC Symbol;Acc:HGNC:8840]	-1.1098031
PER4	period circadian clock 3 pseudogene	-1.0853449
PEX11B	peroxisomal biogenesis factor 11 beta [Source:HGNC Symbol;Acc:HGNC:8853]	-1.2818654
PEX11G	peroxisomal biogenesis factor 11 gamma [Source:HGNC Symbol;Acc:HGNC:20208]	-1.1438046
PEX14	peroxisomal biogenesis factor 14 [Source:HGNC Symbol;Acc:HGNC:8856]	-1.1476759
PEX2	peroxisomal biogenesis factor 2 [Source:HGNC Symbol;Acc:HGNC:9717]	1.0919818
PEX26	peroxisomal biogenesis factor 26 [Source:HGNC Symbol;Acc:HGNC:22965]	-1.2084986
PEX3	peroxisomal biogenesis factor 3 [Source:HGNC Symbol;Acc:HGNC:8858]	1.1109711
PFDN2	prefoldin subunit 2 [Source:HGNC Symbol;Acc:HGNC:8867]	1.1714408
PFKFB2	6-phosphofructo-2-kinase/fructose-2,6-biphosphatase 2 [Source:HGNC Symbol;Acc:HGNC:8873]	-1.4010214
PFKFB3	6-phosphofructo-2-kinase/fructose-2,6-biphosphatase 3 [Source:HGNC Symbol;Acc:HGNC:8874]	-1.6853517
PFKP	phosphofructokinase, platelet [Source:HGNC Symbol;Acc:HGNC:8878]	-1.1174766
PFN1	profilin 1 [Source:HGNC Symbol;Acc:HGNC:8881]	1.0749463
PGA3	pepsinogen 3, group I (pepsinogen A) [Source:HGNC Symbol;Acc:HGNC:8885]	-1.0551398
PGAM4	phosphoglycerate mutase family member 4 [Source:HGNC Symbol;Acc:HGNC:21731]	-1.1258359
PGAP1	post-GPI attachment to proteins 1 [Source:HGNC Symbol;Acc:HGNC:25712]	1.1895629
PGBD2	piggyBac transposable element derived 2 [Source:HGNC Symbol;Acc:HGNC:19399]	1.1030298
PGC	progastricsin [Source:HGNC Symbol;Acc:HGNC:8890]	-1.186319
PGD	phosphogluconate dehydrogenase [Source:HGNC Symbol;Acc:HGNC:8891]	-1.1507444
PGF	placental growth factor [Source:HGNC Symbol;Acc:HGNC:8893]	1.4565831
PGLYRP1	peptidoglycan recognition protein 1 [Source:HGNC Symbol;Acc:HGNC:8904]	-1.1033852
PGLYRP2	peptidoglycan recognition protein 2 [Source:HGNC Symbol;Acc:HGNC:30013]	-1.1462184
PGM1	phosphoglucomutase 1 [Source:HGNC Symbol;Acc:HGNC:8905]	-1.1448034
PGPEP1	pyroglutamyl-peptidase I [Source:HGNC Symbol;Acc:HGNC:13568]	1.489026
PGRMC2	progesterone receptor membrane component 2 [Source:HGNC Symbol;Acc:HGNC:16089]	1.3258793
PHACTR3	phosphatase and actin regulator 3 [Source:HGNC Symbol;Acc:HGNC:15833]	1.1339577
PHB	prohibitin [Source:HGNC Symbol;Acc:HGNC:8912]	-1.1385491
PHF12	PHD finger protein 12 [Source:HGNC Symbol;Acc:HGNC:20816]	-1.1400425
PHF15	jade family PHD finger 2	-1.3133161
PHF17	jade family PHD finger 1	1.5219905
PHF2	PHD finger protein 2 [Source:HGNC Symbol;Acc:HGNC:8920]	-1.078113
PHF20	PHD finger protein 20 [Source:HGNC Symbol;Acc:HGNC:16098]	-1.2209255
PHF23	PHD finger protein 23 [Source:HGNC Symbol;Acc:HGNC:28428]	1.12990695
PHF5A	PHD finger protein 5A [Source:HGNC Symbol;Acc:HGNC:18000]	1.1082019
PHF8	PHD finger protein 8 [Source:HGNC Symbol;Acc:HGNC:20672]	-1.1832398
PHKA2	phosphorylase kinase regulatory subunit alpha 2 [Source:HGNC Symbol;Acc:HGNC:8926]	1.1574057
PHKB	phosphorylase kinase regulatory subunit beta [Source:HGNC Symbol;Acc:HGNC:8927]	1.169951
PHLDA2	pleckstrin homology like domain family A member 2 [Source:HGNC Symbol;Acc:HGNC:12385]	-1.46419
PHLPP2	PH domain and leucine rich repeat protein phosphatase 2 [Source:HGNC Symbol;Acc:HGNC:29149]	-1.1291445
PHRF1	PHD and ring finger domains 1 [Source:HGNC Symbol;Acc:HGNC:24351]	1.0577971
PHTF1	putative homeodomain transcription factor 1 [Source:HGNC Symbol;Acc:HGNC:8939]	1.3572558
PHTF2	putative homeodomain transcription factor 2 [Source:HGNC Symbol;Acc:HGNC:13411]	1.5012875
PI4KB	phosphatidylinositol 4-kinase beta [Source:HGNC Symbol;Acc:HGNC:8984]	-1.21080435
PIAS3	protein inhibitor of activated STAT 3 [Source:HGNC Symbol;Acc:HGNC:16861]	1.0720308
PIGA	phosphatidylinositol glycan anchor biosynthesis class A [Source:HGNC Symbol;Acc:HGNC:8957]	1.1063608
PIGC	phosphatidylinositol glycan anchor biosynthesis class C [Source:HGNC Symbol;Acc:HGNC:8960]	1.1024767
PIGG	phosphatidylinositol glycan anchor biosynthesis class G [Source:HGNC Symbol;Acc:HGNC:25985]	-1.1840585
PIGK	phosphatidylinositol glycan anchor biosynthesis class K [Source:HGNC Symbol;Acc:HGNC:8965]	1.5230957
PIGN	phosphatidylinositol glycan anchor biosynthesis class N [Source:HGNC Symbol;Acc:HGNC:8967]	1.1643058
PIGQ	phosphatidylinositol glycan anchor biosynthesis class Q [Source:HGNC Symbol;Acc:HGNC:14135]	1.1149392
PIGS	phosphatidylinositol glycan anchor biosynthesis class S [Source:HGNC Symbol;Acc:HGNC:14937]	-1.1851413
PIGU	phosphatidylinositol glycan anchor biosynthesis class U [Source:HGNC Symbol;Acc:HGNC:15791]	-1.0932395
PIGX	phosphatidylinositol glycan anchor biosynthesis class X [Source:HGNC Symbol;Acc:HGNC:26046]	1.1303811
PIK3AP1	phosphoinositide-3-kinase adaptor protein 1 [Source:HGNC Symbol;Acc:HGNC:30034]	1.0779663
PIK3C2B	sphatidylinositol-4-phosphate 3-kinase catalytic subunit type 2 beta [Source:HGNC Symbol;Acc:HGNC:8	-1.5629599
PIK3CB	osphatidylinositol-4,5-bisphosphate 3-kinase catalytic subunit beta [Source:HGNC Symbol;Acc:HGNC:89	1.2095671
PIK3CD	osphatidylinositol-4,5-bisphosphate 3-kinase catalytic subunit delta [Source:HGNC Symbol;Acc:HGNC:89	-1.8270771
PIK3CG	phatidylinositol-4,5-bisphosphate 3-kinase catalytic subunit gamma [Source:HGNC Symbol;Acc:HGNC:8	1.5327988
PILRB	paired immunoglobin-like type 2 receptor beta [Source:HGNC Symbol;Acc:HGNC:18297]	-1.0593379
PIM1	Pim-1 proto-oncogene, serine/threonine kinase [Source:HGNC Symbol;Acc:HGNC:8986]	1.2265952
PIN1	peptidylprolyl cis/trans isomerase, NIMA-interacting 1 [Source:HGNC Symbol;Acc:HGNC:8988]	1.2424008
PIN1P1	ptidylprolyl cis/trans isomerase, NIMA-interacting 1 pseudogene 1 [Source:HGNC Symbol;Acc:HGNC:89	1.2454847
PINX1	PIN2/TERF1 interacting, telomerase inhibitor 1 [Source:HGNC Symbol;Acc:HGNC:30046]	-1.2181548
PION	gamma-secretase activating protein	-1.1931988
PIP5K1B	phosphatidylinositol-4-phosphate 5-kinase type 1 beta [Source:HGNC Symbol;Acc:HGNC:8995]	1.0794615
PIP5K1P1	hosphatidylinositol-4-phosphate 5-kinase type 1 pseudogene 1 [Source:HGNC Symbol;Acc:HGNC:28372	-1.0974145
PITPNM3	PITPNM family member 3 [Source:HGNC Symbol;Acc:HGNC:21043]	1.2326429
PITX3	paired like homeodomain 3 [Source:HGNC Symbol;Acc:HGNC:9006]	-1.154338
PJA1	praja ring finger ubiquitin ligase 1 [Source:HGNC Symbol;Acc:HGNC:16648]	-1.2743773
PKD1	polycystin 1, transient receptor potential channel interacting [Source:HGNC Symbol;Acc:HGNC:9008]	-1.2798247

PKD1L2	polycystin 1 like 2 (gene/pseudogene) [Source:HGNC Symbol;Acc:HGNC:21715]	-1.0772259
PKDCC	protein kinase domain containing, cytoplasmic [Source:HGNC Symbol;Acc:HGNC:25123]	1.39499845
PKIG	protein kinase (cAMP-dependent, catalytic) inhibitor gamma [Source:HGNC Symbol;Acc:HGNC:9019]	-1.373575
PKNOX2	PBX/knotted 1 homeobox 2 [Source:HGNC Symbol;Acc:HGNC:16714]	1.0923101
PKP2	plakophilin 2 [Source:HGNC Symbol;Acc:HGNC:9024]	1.1693565
PKP4	plakophilin 4 [Source:HGNC Symbol;Acc:HGNC:9026]	1.1752901
PLA2G12A	phospholipase A2 group XIIA [Source:HGNC Symbol;Acc:HGNC:18554]	-1.0684586
PLA2G16	phospholipase A2 group XVI [Source:HGNC Symbol;Acc:HGNC:17825]	1.2061101
PLA2G2C	phospholipase A2 group IIC [Source:HGNC Symbol;Acc:HGNC:9032]	-1.124607
PLA2G2D	phospholipase A2 group IID [Source:HGNC Symbol;Acc:HGNC:9033]	-1.1052215
PLA2G4A	phospholipase A2 group IVA [Source:HGNC Symbol;Acc:HGNC:9035]	1.2550426
PLA2G4C	phospholipase A2 group IVC [Source:HGNC Symbol;Acc:HGNC:9037]	-1.6717633
PLA2G5	phospholipase A2 group V [Source:HGNC Symbol;Acc:HGNC:9038]	-1.5316108
PLAC8	placenta specific 8 [Source:HGNC Symbol;Acc:HGNC:19254]	1.0766348
PLAC9	placenta specific 9 [Source:HGNC Symbol;Acc:HGNC:19255]	-1.2373068
PLAGL2	PLAG1 like zinc finger 2 [Source:HGNC Symbol;Acc:HGNC:9047]	-1.2629566
PLAU	plasminogen activator, urokinase [Source:HGNC Symbol;Acc:HGNC:9052]	-3.6658466
PLAUR	plasminogen activator, urokinase receptor [Source:HGNC Symbol;Acc:HGNC:9053]	-1.1335421
PLCB1	phospholipase C beta 1 [Source:HGNC Symbol;Acc:HGNC:15917]	1.0977348
PLCB2	phospholipase C beta 2 [Source:HGNC Symbol;Acc:HGNC:9055]	1.3084667
PLCB4	phospholipase C beta 4 [Source:HGNC Symbol;Acc:HGNC:9059]	-1.2671567
PLCD3	phospholipase C delta 3 [Source:HGNC Symbol;Acc:HGNC:9061]	1.3275344
PLCD4	phospholipase C delta 4 [Source:HGNC Symbol;Acc:HGNC:9062]	-1.5221984
PLCE1	phospholipase C epsilon 1 [Source:HGNC Symbol;Acc:HGNC:17175]	-1.1013318
PLCG2	phospholipase C gamma 2 [Source:HGNC Symbol;Acc:HGNC:9066]	-1.1942577
PLCL2	phospholipase C like 2 [Source:HGNC Symbol;Acc:HGNC:9064]	1.1478751
PLCXD1	osphatidylinositol specific phospholipase C X domain containing 1 [Source:HGNC Symbol;Acc:HGNC:231	1.2977169
PLCXD3	osphatidylinositol specific phospholipase C X domain containing 3 [Source:HGNC Symbol;Acc:HGNC:318	1.06927
PLD2	phospholipase D2 [Source:HGNC Symbol;Acc:HGNC:9068]	-1.4411585
PLD6	phospholipase D family member 6 [Source:HGNC Symbol;Acc:HGNC:30447]	1.2023072
PLEC	plectin [Source:HGNC Symbol;Acc:HGNC:9069]	1.2061851
PLEKHA4	pleckstrin homology domain containing A4 [Source:HGNC Symbol;Acc:HGNC:14339]	-1.3045158
PLEKHA6	pleckstrin homology domain containing A6 [Source:HGNC Symbol;Acc:HGNC:17053]	1.3253518
PLEKHB2	pleckstrin homology domain containing B2 [Source:HGNC Symbol;Acc:HGNC:19236]	-1.2048403
PLEKHF2	pleckstrin homology and FYVE domain containing 2 [Source:HGNC Symbol;Acc:HGNC:20757]	1.2496653
PLEKHG1	pleckstrin homology and RhoGEF domain containing G1 [Source:HGNC Symbol;Acc:HGNC:20884]	-1.127959
PLEKHG3	pleckstrin homology and RhoGEF domain containing G3 [Source:HGNC Symbol;Acc:HGNC:20364]	-1.2429764
PLEKHG4	pleckstrin homology and RhoGEF domain containing G4 [Source:HGNC Symbol;Acc:HGNC:24501]	1.1478274
PLEKHG4B	pleckstrin homology and RhoGEF domain containing G4B [Source:HGNC Symbol;Acc:HGNC:29399]	1.1454644
PLEKHG7	pleckstrin homology and RhoGEF domain containing G7 [Source:HGNC Symbol;Acc:HGNC:33829]	1.1128165
PLEKHM1	pleckstrin homology and RUN domain containing M1 [Source:HGNC Symbol;Acc:HGNC:29017]	-1.1965452
PLEKHO1	pleckstrin homology domain containing O1 [Source:HGNC Symbol;Acc:HGNC:24310]	1.0656023
PLEKHO2	pleckstrin homology domain containing O2 [Source:HGNC Symbol;Acc:HGNC:30026]	-1.6812955
PLG	plasminogen [Source:HGNC Symbol;Acc:HGNC:9071]	1.080244
PLIN2	perilipin 2 [Source:HGNC Symbol;Acc:HGNC:248]	1.1608387
PLIN4	perilipin 4 [Source:HGNC Symbol;Acc:HGNC:29393]	-1.1176945
PLK1	polo like kinase 1 [Source:HGNC Symbol;Acc:HGNC:9077]	1.133942
PLK2	polo like kinase 2 [Source:HGNC Symbol;Acc:HGNC:19699]	-1.5800034
PLK3	polo like kinase 3 [Source:HGNC Symbol;Acc:HGNC:2154]	-1.1642267
PLK4	polo like kinase 4 [Source:HGNC Symbol;Acc:HGNC:11397]	1.383828
PLLP	plasmolipin [Source:HGNC Symbol;Acc:HGNC:18553]	-1.8300961
PLXDC1	plexin domain containing 1 [Source:HGNC Symbol;Acc:HGNC:20945]	-1.1078465
PLXNA1	plexin A1 [Source:HGNC Symbol;Acc:HGNC:9099]	-1.3369428
PLXNA2	plexin A2 [Source:HGNC Symbol;Acc:HGNC:9100]	1.2145233
PLXNB1	plexin B1 [Source:HGNC Symbol;Acc:HGNC:9103]	-1.1343492
PLXNB2	plexin B2 [Source:HGNC Symbol;Acc:HGNC:9104]	-1.1624212
PLXNB3	plexin B3 [Source:HGNC Symbol;Acc:HGNC:9105]	1.1134405
PM20D1	peptidase M20 domain containing 1 [Source:HGNC Symbol;Acc:HGNC:26518]	1.0770282
PMAIP1	phorbol-12-myristate-13-acetate-induced protein 1 [Source:HGNC Symbol;Acc:HGNC:9108]	-1.9358847
PMEPA1	prostate transmembrane protein, androgen induced 1 [Source:HGNC Symbol;Acc:HGNC:14107]	1.3048342
PMFBP1	polyamine modulated factor 1 binding protein 1 [Source:HGNC Symbol;Acc:HGNC:17728]	-1.071204
PML	promyelocytic leukemia [Source:HGNC Symbol;Acc:HGNC:9113]	-1.576471533
PMP22	peripheral myelin protein 22 [Source:HGNC Symbol;Acc:HGNC:9118]	-1.4879855
PMS2L2	PMS1 homolog 2, mismatch repair system component pseudogene 2	1.0623374
PMS2P5	IS1 homolog 2, mismatch repair system component pseudogene 5 [Source:HGNC Symbol;Acc:HGNC:91	-1.14247
PNKD	paroxysmal nonkinesigenic dyskinesia [Source:HGNC Symbol;Acc:HGNC:9153]	-1.1933361
PNMA6A	paraneoplastic Ma antigen family member 6A [Source:HGNC Symbol;Acc:HGNC:28248]	1.1078731
PNMAL1	paraneoplastic Ma antigen family like 1 [Source:HGNC Symbol;Acc:HGNC:25578]	1.3285961
PNMAL2	paraneoplastic Ma antigen family like 2 [Source:HGNC Symbol;Acc:HGNC:29206]	-1.2039226
PNP	purine nucleoside phosphorylase [Source:HGNC Symbol;Acc:HGNC:7892]	1.2035432
PNPLA4	patatin like phospholipase domain containing 4 [Source:HGNC Symbol;Acc:HGNC:24887]	-1.0947318
PNPT1	polyribonucleotide nucleotidyltransferase 1 [Source:HGNC Symbol;Acc:HGNC:23166]	1.363815
PNRC1	proline rich nuclear receptor coactivator 1 [Source:HGNC Symbol;Acc:HGNC:17278]	-1.491026
POFUT2	protein O-fucosyltransferase 2 [Source:HGNC Symbol;Acc:HGNC:14683]	1.126868
POGK	pogo transposable element with KRAB domain [Source:HGNC Symbol;Acc:HGNC:18800]	-1.2757027
POLD3	DNA polymerase delta 3, accessory subunit [Source:HGNC Symbol;Acc:HGNC:20932]	1.2591331
POLDIP3	DNA polymerase delta interacting protein 3 [Source:HGNC Symbol;Acc:HGNC:23782]	-1.1764833
POLE	DNA polymerase epsilon, catalytic subunit [Source:HGNC Symbol;Acc:HGNC:9177]	1.1039143
POLG	DNA polymerase gamma, catalytic subunit [Source:HGNC Symbol;Acc:HGNC:9179]	-1.1557217
POLN	DNA polymerase nu [Source:HGNC Symbol;Acc:HGNC:18870]	1.1041567
POLR1C	RNA polymerase I subunit C [Source:HGNC Symbol;Acc:HGNC:20194]	-1.0941062
POLR2B	RNA polymerase II subunit B [Source:HGNC Symbol;Acc:HGNC:9188]	1.3690048
POLR2D	RNA polymerase II subunit D [Source:HGNC Symbol;Acc:HGNC:9191]	-1.1413199
POLR2G	RNA polymerase II subunit G [Source:HGNC Symbol;Acc:HGNC:9194]	-1.1731031
POLR2H	RNA polymerase II subunit H [Source:HGNC Symbol;Acc:HGNC:9195]	1.1129702
POLR2K	RNA polymerase II subunit K [Source:HGNC Symbol;Acc:HGNC:9198]	-1.1619179

POLR3E	RNA polymerase III subunit E [Source:HGNC Symbol;Acc:HGNC:30347]	-1.1106274
POLR3G	RNA polymerase III subunit G [Source:HGNC Symbol;Acc:HGNC:30075]	1.5255877
POLR3GL	RNA polymerase III subunit G like [Source:HGNC Symbol;Acc:HGNC:28466]	1.0729084
POLR3H	RNA polymerase III subunit H [Source:HGNC Symbol;Acc:HGNC:30349]	1.2078291
POLR3K	RNA polymerase III subunit K [Source:HGNC Symbol;Acc:HGNC:14121]	1.2108947
POM121L10P	POM121 transmembrane nucleoporin like 10, pseudogene [Source:HGNC Symbol;Acc:HGNC:35448]	1.1422875
POM121L1P	POM121 transmembrane nucleoporin like 1, pseudogene [Source:HGNC Symbol;Acc:HGNC:16439]	1.0987558
POM121L2	POM121 transmembrane nucleoporin like 2 [Source:HGNC Symbol;Acc:HGNC:13973]	1.0866201
POM121L4P	POM121 transmembrane nucleoporin like 4, pseudogene [Source:HGNC Symbol;Acc:HGNC:19326]	-1.1346741
POMT2	protein O-mannosyltransferase 2 [Source:HGNC Symbol;Acc:HGNC:19743]	1.0813807
POP5	POP5 homolog, ribonuclease P/MRP subunit [Source:HGNC Symbol;Acc:HGNC:17689]	-1.1359521
POR	cytochrome p450 oxidoreductase [Source:HGNC Symbol;Acc:HGNC:9208]	-1.1947742
POTEF	POTE ankyrin domain family member F [Source:HGNC Symbol;Acc:HGNC:33905]	-1.1613919
PPAN-P2RY11	PPAN-P2RY11 readthrough [Source:HGNC Symbol;Acc:HGNC:33526]	1.1644465
PPAP2A	phospholipid phosphatase 1	-1.7911497
PPAP2B	phospholipid phosphatase 3	-1.4292244
PPARGC1B	PPARG coactivator 1 beta [Source:HGNC Symbol;Acc:HGNC:30022]	1.2223347
PPAT	phosphoribosyl pyrophosphate amidotransferase [Source:HGNC Symbol;Acc:HGNC:9238]	1.6158689
PPCDC	phosphopantothenoylcysteine decarboxylase [Source:HGNC Symbol;Acc:HGNC:28107]	-1.0662992
PPFIBP2	PPFIA binding protein 2 [Source:HGNC Symbol;Acc:HGNC:9250]	1.2659147
PPIA	peptidylprolyl isomerase A [Source:HGNC Symbol;Acc:HGNC:9253]	-1.0948275
PPIAL4G	peptidylprolyl isomerase A like 4G [Source:HGNC Symbol;Acc:HGNC:33996]	-1.1838534
PPIF	peptidylprolyl isomerase F [Source:HGNC Symbol;Acc:HGNC:9259]	-1.1751583
PPIH	peptidylprolyl isomerase H [Source:HGNC Symbol;Acc:HGNC:14651]	-1.0856756
PPIL2	peptidylprolyl isomerase like 2 [Source:HGNC Symbol;Acc:HGNC:9261]	-1.1240679
PPL	periplakin [Source:HGNC Symbol;Acc:HGNC:9273]	-1.1562318
PPM1D	protein phosphatase, Mg2+/Mn2+ dependent 1D [Source:HGNC Symbol;Acc:HGNC:9277]	1.1839567
PPM1F	protein phosphatase, Mg2+/Mn2+ dependent 1F [Source:HGNC Symbol;Acc:HGNC:19388]	-1.3110054
PPM1J	protein phosphatase, Mg2+/Mn2+ dependent 1J [Source:HGNC Symbol;Acc:HGNC:20785]	-1.0835079
PPME1	protein phosphatase methylesterase 1 [Source:HGNC Symbol;Acc:HGNC:30178]	-1.1195854
PPP1R10	protein phosphatase 1 regulatory subunit 10 [Source:HGNC Symbol;Acc:HGNC:9284]	1.0740864
PPP1R13B	protein phosphatase 1 regulatory subunit 13B [Source:HGNC Symbol;Acc:HGNC:14950]	-1.7147648
PPP1R15A	protein phosphatase 1 regulatory subunit 15A [Source:HGNC Symbol;Acc:HGNC:14375]	-1.2326063
PPP1R3A	protein phosphatase 1 regulatory subunit 3A [Source:HGNC Symbol;Acc:HGNC:9291]	1.0816936
PPP1R3B	protein phosphatase 1 regulatory subunit 3B [Source:HGNC Symbol;Acc:HGNC:14942]	1.3317975
PPP1R3G	protein phosphatase 1 regulatory subunit 3G [Source:HGNC Symbol;Acc:HGNC:14945]	-1.170762
PPP1R9A	protein phosphatase 1 regulatory subunit 9A [Source:HGNC Symbol;Acc:HGNC:14946]	1.1525604
PPP2CA	protein phosphatase 2 catalytic subunit alpha [Source:HGNC Symbol;Acc:HGNC:9299]	-1.1998922
PPP2R1B	protein phosphatase 2 scaffold subunit Abeta [Source:HGNC Symbol;Acc:HGNC:9303]	-1.2915275
PPP2R3C	protein phosphatase 2 regulatory subunit B''gamma [Source:HGNC Symbol;Acc:HGNC:17485]	1.3481054
PPP2R5B	protein phosphatase 2 regulatory subunit B'beta [Source:HGNC Symbol;Acc:HGNC:9310]	-1.1320546
PPP3CB	protein phosphatase 3 catalytic subunit beta [Source:HGNC Symbol;Acc:HGNC:9315]	-1.1937457
PPP3CC	protein phosphatase 3 catalytic subunit gamma [Source:HGNC Symbol;Acc:HGNC:9316]	-1.0698309
PPP4R1L	protein phosphatase 4 regulatory subunit 1 like (pseudogene) [Source:HGNC Symbol;Acc:HGNC:15755]	-1.2356414
PPP5C	protein phosphatase 5 catalytic subunit [Source:HGNC Symbol;Acc:HGNC:9322]	1.4402192
PPT2	palmitoyl-protein thioesterase 2 [Source:HGNC Symbol;Acc:HGNC:9326]	1.2660841
PQLC1	PQ loop repeat containing 1 [Source:HGNC Symbol;Acc:HGNC:26188]	-1.3197469
PRAMEF2	PRAME family member 2 [Source:HGNC Symbol;Acc:HGNC:28841]	1.1137437
PRCD	progressive rod-cone degeneration [Source:HGNC Symbol;Acc:HGNC:32528]	-1.0792962
PRDM1	PR/SET domain 1 [Source:HGNC Symbol;Acc:HGNC:9346]	-1.5286527
PRDM12	PR/SET domain 12 [Source:HGNC Symbol;Acc:HGNC:13997]	1.1000183
PRDM14	PR/SET domain 14 [Source:HGNC Symbol;Acc:HGNC:14001]	1.0806712
PRDM8	PR/SET domain 8 [Source:HGNC Symbol;Acc:HGNC:13993]	-2.9975948
PRDM9	PR/SET domain 9 [Source:HGNC Symbol;Acc:HGNC:13994]	-1.1094548
PRDX1	peroxiredoxin 1 [Source:HGNC Symbol;Acc:HGNC:9352]	1.2535508
PRDX5	peroxiredoxin 5 [Source:HGNC Symbol;Acc:HGNC:9355]	-1.0715536
PREPL	prolyl endopeptidase-like [Source:HGNC Symbol;Acc:HGNC:30228]	1.3544335
PRG1	serglycin	1.1098455
PRIC285	helicase with zinc finger 2	-2.2810578
PRICKLE1	prickle planar cell polarity protein 1 [Source:HGNC Symbol;Acc:HGNC:17019]	2.1234584
PRIM1	primase (DNA) subunit 1 [Source:HGNC Symbol;Acc:HGNC:9369]	1.2464645
PRINS	psoriasis associated non-protein coding RNA induced by stress	-1.8291407
PRKACA	protein kinase cAMP-activated catalytic subunit alpha [Source:HGNC Symbol;Acc:HGNC:9380]	1.3031893
PRKACB	protein kinase cAMP-activated catalytic subunit beta [Source:HGNC Symbol;Acc:HGNC:9381]	2.0870671
PRKAR1A	protein kinase cAMP-dependent type I regulatory subunit alpha [Source:HGNC Symbol;Acc:HGNC:9388]	-1.0741287
PRKCD	protein kinase C delta [Source:HGNC Symbol;Acc:HGNC:9399]	-1.8098637
PRKCE	protein kinase C epsilon [Source:HGNC Symbol;Acc:HGNC:9401]	1.539213
PRKCH	protein kinase C eta [Source:HGNC Symbol;Acc:HGNC:9403]	1.2702664
PRKCI	protein kinase C iota [Source:HGNC Symbol;Acc:HGNC:9404]	1.3324362
PRKCQ	protein kinase C theta [Source:HGNC Symbol;Acc:HGNC:9410]	1.0667999
PRKCSH	protein kinase C substrate 80K-H [Source:HGNC Symbol;Acc:HGNC:9411]	1.3278368
PRKD2	protein kinase D2 [Source:HGNC Symbol;Acc:HGNC:17293]	-1.4493564
PRKG2	protein kinase, cGMP-dependent, type II [Source:HGNC Symbol;Acc:HGNC:9416]	1.1287003
PRKRIR	THAP domain containing 12	1.1619819
PRLR	prolactin receptor [Source:HGNC Symbol;Acc:HGNC:9446]	1.1165869
PRM1	protamine 1 [Source:HGNC Symbol;Acc:HGNC:9447]	-1.0934547
PRMT1	protein arginine methyltransferase 1 [Source:HGNC Symbol;Acc:HGNC:5187]	-1.1144887
PRMT2	protein arginine methyltransferase 2 [Source:HGNC Symbol;Acc:HGNC:5186]	-1.108796
PRMT3	protein arginine methyltransferase 3 [Source:HGNC Symbol;Acc:HGNC:30163]	1.2014946
PRMT5	protein arginine methyltransferase 5 [Source:HGNC Symbol;Acc:HGNC:10894]	-1.1139424
PRMT6	protein arginine methyltransferase 6 [Source:HGNC Symbol;Acc:HGNC:18241]	1.1238465
PRO0471	uncharacterized LOC28994	1.0943657
PRO0611	PRO0611 protein	1.0776019
PROCR	protein C receptor [Source:HGNC Symbol;Acc:HGNC:9452]	1.1413561
PRODH	proline dehydrogenase 1 [Source:HGNC Symbol;Acc:HGNC:9453]	-1.1985888
ProSAPiP1	leucine zipper tumor suppressor family member 3	-1.254513

PRPF18	pre-mRNA processing factor 18 [Source:HGNC Symbol;Acc:HGNC:17351]	1.3307501
PRPF6	pre-mRNA processing factor 6 [Source:HGNC Symbol;Acc:HGNC:15860]	-1.0603973
PRR18	proline rich 18 [Source:HGNC Symbol;Acc:HGNC:28574]	-1.1312983
PRR23B	proline rich 23B [Source:HGNC Symbol;Acc:HGNC:33764]	-1.085348
PRR7	proline rich 7 (synaptic) [Source:HGNC Symbol;Acc:HGNC:28130]	1.5090052
PRRC2A	proline rich coiled-coil 2A [Source:HGNC Symbol;Acc:HGNC:13918]	1.3779577
PRRG1	proline rich and Gla domain 1 [Source:HGNC Symbol;Acc:HGNC:9469]	-1.2336901
PRRG2	proline rich and Gla domain 2 [Source:HGNC Symbol;Acc:HGNC:9470]	1.1438161
PRRG3	proline rich and Gla domain 3 [Source:HGNC Symbol;Acc:HGNC:30798]	-1.1878572
PRRT1	proline rich transmembrane protein 1 [Source:HGNC Symbol;Acc:HGNC:13943]	-1.0999475
PRRT4	proline rich transmembrane protein 4 [Source:HGNC Symbol;Acc:HGNC:37280]	1.2130818
PRSS36	protease, serine 36 [Source:HGNC Symbol;Acc:HGNC:26906]	-1.1630216
PRSS8	protease, serine 8 [Source:HGNC Symbol;Acc:HGNC:9491]	-1.0591403
PRTG	protogenin [Source:HGNC Symbol;Acc:HGNC:26373]	1.1733447
PRTN3	proteinase 3 [Source:HGNC Symbol;Acc:HGNC:9495]	-1.130987
PRUNE2	prune homolog 2 [Source:HGNC Symbol;Acc:HGNC:25209]	1.2618607
PSD2	pleckstrin and Sec7 domain containing 2 [Source:HGNC Symbol;Acc:HGNC:19092]	1.1004092
PSEN1	presenilin 1 [Source:HGNC Symbol;Acc:HGNC:9508]	-1.567963
PSEN2	presenilin 2 [Source:HGNC Symbol;Acc:HGNC:9509]	-1.6942778
psiTPTE22	transmembrane phosphatase with tensin homology pseudogene 1	-1.074293
PSMA6	proteasome subunit alpha 6 [Source:HGNC Symbol;Acc:HGNC:9535]	-1.2066969
PSMB10	proteasome subunit beta 10 [Source:HGNC Symbol;Acc:HGNC:9538]	-1.2886705
PSMB8	proteasome subunit beta 8 [Source:HGNC Symbol;Acc:HGNC:9545]	-1.4257451
PSMB9	proteasome subunit beta 9 [Source:HGNC Symbol;Acc:HGNC:9546]	-1.4243968
PSMC3	proteasome 26S subunit, ATPase 3 [Source:HGNC Symbol;Acc:HGNC:9549]	1.0500098
PSMC3IP	PSMC3 interacting protein [Source:HGNC Symbol;Acc:HGNC:17928]	1.1516057
PSMD11	proteasome 26S subunit, non-ATPase 11 [Source:HGNC Symbol;Acc:HGNC:9556]	1.2300076
PSMD4	proteasome 26S subunit, non-ATPase 4 [Source:HGNC Symbol;Acc:HGNC:9561]	-1.0817127
PSMD5	proteasome 26S subunit, non-ATPase 5 [Source:HGNC Symbol;Acc:HGNC:9563]	1.0886635
PSMD9	proteasome 26S subunit, non-ATPase 9 [Source:HGNC Symbol;Acc:HGNC:9567]	1.125119
PSME1	proteasome activator subunit 1 [Source:HGNC Symbol;Acc:HGNC:9568]	-1.3515717
PSME2	proteasome activator subunit 2 [Source:HGNC Symbol;Acc:HGNC:9569]	-1.3862005
PSME4	proteasome activator subunit 4 [Source:HGNC Symbol;Acc:HGNC:20635]	1.251497
PSMG2	proteasome assembly chaperone 2 [Source:HGNC Symbol;Acc:HGNC:24929]	-1.1941135
PSPH	phosphoserine phosphatase [Source:HGNC Symbol;Acc:HGNC:9577]	1.1632761
PTAFR	platelet activating factor receptor [Source:HGNC Symbol;Acc:HGNC:9582]	-1.3644845
PTBP1	polypyrimidine tract binding protein 1 [Source:HGNC Symbol;Acc:HGNC:9583]	-1.2254856
PTCH2	patched 2 [Source:HGNC Symbol;Acc:HGNC:9586]	-1.1322902
PTCHD3	patched domain containing 3 [Source:HGNC Symbol;Acc:HGNC:24776]	1.0956552
PTEN	phosphatase and tensin homolog [Source:HGNC Symbol;Acc:HGNC:9588]	1.1696136
PTF1A	pancreas specific transcription factor, 1a [Source:HGNC Symbol;Acc:HGNC:23734]	1.1751045
PTGFR	prostaglandin F receptor [Source:HGNC Symbol;Acc:HGNC:9600]	-1.2229843
PTGFRN	prostaglandin F2 receptor inhibitor [Source:HGNC Symbol;Acc:HGNC:9601]	-1.2263702
PTGIS	prostaglandin I2 (prostacyclin) synthase [Source:HGNC Symbol;Acc:HGNC:9603]	-1.1255989
PTGS2	prostaglandin-endoperoxide synthase 2 [Source:HGNC Symbol;Acc:HGNC:9605]	-1.4346144
PTHLH	parathyroid hormone like hormone [Source:HGNC Symbol;Acc:HGNC:9607]	-1.216422
PTP4A1	protein tyrosine phosphatase type IVA, member 1 [Source:HGNC Symbol;Acc:HGNC:9634]	1.2091388
PTP4A2	protein tyrosine phosphatase type IVA, member 2 [Source:HGNC Symbol;Acc:HGNC:9635]	1.2856109
PTPLA	3-hydroxyacyl-CoA dehydratase 1	-1.0671303
PTPN20B	protein tyrosine phosphatase, non-receptor type 20	-1.07132435
PTPN3	protein tyrosine phosphatase, non-receptor type 3 [Source:HGNC Symbol;Acc:HGNC:9655]	1.1272525
PTPRF	protein tyrosine phosphatase, receptor type F [Source:HGNC Symbol;Acc:HGNC:9670]	-1.131666
PTPRK	protein tyrosine phosphatase, receptor type K [Source:HGNC Symbol;Acc:HGNC:9674]	-1.37492985
PTPRM	protein tyrosine phosphatase, receptor type M [Source:HGNC Symbol;Acc:HGNC:9675]	-1.2529433
PTPRN2	protein tyrosine phosphatase, receptor type N2 [Source:HGNC Symbol;Acc:HGNC:9677]	1.0979127
PTPRS	protein tyrosine phosphatase, receptor type S [Source:HGNC Symbol;Acc:HGNC:9681]	-1.1809596
PTPRT	protein tyrosine phosphatase, receptor type T [Source:HGNC Symbol;Acc:HGNC:9682]	1.1026493
PTPRVP	protein tyrosine phosphatase, receptor type V, pseudogene [Source:HGNC Symbol;Acc:HGNC:13421]	1.1104845
PTTG1	pituitary tumor-transforming 1 [Source:HGNC Symbol;Acc:HGNC:9690]	-1.0639142
PTTG1IP	pituitary tumor-transforming 1 interacting protein [Source:HGNC Symbol;Acc:HGNC:13524]	-1.2765256
PTTG2	pituitary tumor-transforming 2 [Source:HGNC Symbol;Acc:HGNC:9691]	1.1118758
PUS1	pseudouridylate synthase 1 [Source:HGNC Symbol;Acc:HGNC:15508]	1.1251047
PUS10	pseudouridylate synthase 10 [Source:HGNC Symbol;Acc:HGNC:26505]	1.0638803
PUS3	pseudouridylate synthase 3 [Source:HGNC Symbol;Acc:HGNC:25461]	1.2649394
PUS7L	pseudouridylate synthase 7 like [Source:HGNC Symbol;Acc:HGNC:25276]	1.1140956
PVR	poliovirus receptor [Source:HGNC Symbol;Acc:HGNC:9705]	-1.3686592
PVRL2	nectin cell adhesion molecule 2	-1.275217
PVRL3	nectin cell adhesion molecule 3	1.5390055
PVT1	Pvt1 oncogene (non-protein coding) [Source:HGNC Symbol;Acc:HGNC:9709]	-1.2852803
PWRN2	Prader-Willi region non-protein coding RNA 2 [Source:HGNC Symbol;Acc:HGNC:33236]	1.092489
PXDN	peroxidasin [Source:HGNC Symbol;Acc:HGNC:14966]	-1.24636355
PXMP4	peroxisomal membrane protein 4 [Source:HGNC Symbol;Acc:HGNC:15920]	1.1793512
PXN	paxillin [Source:HGNC Symbol;Acc:HGNC:9718]	1.4310409
PYCRL	pyrroline-5-carboxylate reductase-like [Source:HGNC Symbol;Acc:HGNC:25846]	1.2244414
PYGB	phosphorylase, glycogen; brain [Source:HGNC Symbol;Acc:HGNC:9723]	1.2334601
PYGO1	pygopus family PHD finger 1 [Source:HGNC Symbol;Acc:HGNC:30256]	1.2473793
QARS	glutaminyl-tRNA synthetase [Source:HGNC Symbol;Acc:HGNC:9751]	-1.1252935
QKI	QKI, KH domain containing RNA binding [Source:HGNC Symbol;Acc:HGNC:21100]	-1.0712098
QRSL1	glutaminyl-tRNA synthase (glutamine-hydrolyzing)-like 1 [Source:HGNC Symbol;Acc:HGNC:21020]	1.078503
R3HDM2	R3H domain containing 2 [Source:HGNC Symbol;Acc:HGNC:29167]	1.0525253
RAB10	RAB10, member RAS oncogene family [Source:HGNC Symbol;Acc:HGNC:9759]	1.2290475
RAB11FIP4	RAB11 family interacting protein 4 [Source:HGNC Symbol;Acc:HGNC:30267]	-1.1446735
RAB12	RAB12, member RAS oncogene family [Source:HGNC Symbol;Acc:HGNC:31332]	-1.63504765
RAB13	RAB13, member RAS oncogene family [Source:HGNC Symbol;Acc:HGNC:9762]	-1.1494269
RAB14	RAB14, member RAS oncogene family [Source:HGNC Symbol;Acc:HGNC:16524]	1.1026078
RAB19	RAB19, member RAS oncogene family [Source:HGNC Symbol;Acc:HGNC:19982]	-1.1146326

RAB1B	RAB1B, member RAS oncogene family [Source:HGNC Symbol;Acc:HGNC:18370]	1.801651
RAB25	RAB25, member RAS oncogene family [Source:HGNC Symbol;Acc:HGNC:18238]	1.1034021
RAB28	RAB28, member RAS oncogene family [Source:HGNC Symbol;Acc:HGNC:9768]	1.1453584
RAB2B	RAB2B, member RAS oncogene family [Source:HGNC Symbol;Acc:HGNC:20246]	-1.2159598
RAB35	RAB35, member RAS oncogene family [Source:HGNC Symbol;Acc:HGNC:9774]	-1.0605818
RAB38	RAB38, member RAS oncogene family [Source:HGNC Symbol;Acc:HGNC:9776]	-1.278485
RAB3A	RAB3A, member RAS oncogene family [Source:HGNC Symbol;Acc:HGNC:9777]	1.2566376
RAB3B	RAB3B, member RAS oncogene family [Source:HGNC Symbol;Acc:HGNC:9778]	1.0797741
RAB3C	RAB3C, member RAS oncogene family [Source:HGNC Symbol;Acc:HGNC:30269]	-1.1289219
RAB7B	RAB7B, member RAS oncogene family [Source:HGNC Symbol;Acc:HGNC:30513]	1.0883194
RAB7L1	RAB29, member RAS oncogene family	-1.2832674
RAB9BP1	RAB9B, member RAS oncogene family pseudogene 1 [Source:HGNC Symbol;Acc:HGNC:9793]	-1.2524675
RABEPK	Rab9 effector protein with kelch motifs [Source:HGNC Symbol;Acc:HGNC:16896]	-1.3201813
RABGAP1L	RAB GTPase activating protein 1 like [Source:HGNC Symbol;Acc:HGNC:24663]	1.1952218
RABL2A	RAB, member of RAS oncogene family-like 2A [Source:HGNC Symbol;Acc:HGNC:9799]	-1.112319267
RABL2B	RAB, member of RAS oncogene family-like 2B [Source:HGNC Symbol;Acc:HGNC:9800]	-1.1966829
RAD1	RAD1 checkpoint DNA exonuclease [Source:HGNC Symbol;Acc:HGNC:9806]	-1.2265265
RAD21	RAD21 cohesin complex component [Source:HGNC Symbol;Acc:HGNC:9811]	1.4785478
RAD51AP1	RAD51 associated protein 1 [Source:HGNC Symbol;Acc:HGNC:16956]	1.3344936
RAD51C	RAD51 paralog C [Source:HGNC Symbol;Acc:HGNC:9820]	1.1059057
RAD51D	RAD51 paralog D [Source:HGNC Symbol;Acc:HGNC:9823]	-1.1348433
RAD54L	RAD54-like (S. cerevisiae) [Source:HGNC Symbol;Acc:HGNC:9826]	1.1402247
RADIL	Rap associating with DIL domain [Source:HGNC Symbol;Acc:HGNC:22226]	-1.3268523
RAE1	ribonucleic acid export 1 [Source:HGNC Symbol;Acc:HGNC:9828]	-1.1300287
RAGE	MOK protein kinase	-1.2160566
RAI1	retinoic acid induced 1 [Source:HGNC Symbol;Acc:HGNC:9834]	-1.1390872
RALGPS1	Ral GEF with PH domain and SH3 binding motif 1 [Source:HGNC Symbol;Acc:HGNC:16851]	-1.1327692
RALYL	RALY RNA binding protein-like [Source:HGNC Symbol;Acc:HGNC:27036]	1.12957865
RAMP3	receptor activity modifying protein 3 [Source:HGNC Symbol;Acc:HGNC:9845]	-1.1163145
RANBP10	RAN binding protein 10 [Source:HGNC Symbol;Acc:HGNC:29285]	-1.5057667
RANBP2	RAN binding protein 2 [Source:HGNC Symbol;Acc:HGNC:9848]	1.4194016
RANBP3	RAN binding protein 3 [Source:HGNC Symbol;Acc:HGNC:9850]	1.1505704
RANBP9	RAN binding protein 9 [Source:HGNC Symbol;Acc:HGNC:13727]	-1.1893266
RAP1GAP	RAP1 GTPase activating protein [Source:HGNC Symbol;Acc:HGNC:9858]	1.1266047
RAP1GDS1	Rap1 GTPase-GDP dissociation stimulator 1 [Source:HGNC Symbol;Acc:HGNC:9859]	1.1494986
RAP2B	RAP2B, member of RAS oncogene family [Source:HGNC Symbol;Acc:HGNC:9862]	1.1527528
RAP2C	RAP2C, member of RAS oncogene family [Source:HGNC Symbol;Acc:HGNC:21165]	-1.1698175
RAPGEF1	Rap guanine nucleotide exchange factor 1 [Source:HGNC Symbol;Acc:HGNC:4568]	-1.1994383
RAPGEF3	Rap guanine nucleotide exchange factor 3 [Source:HGNC Symbol;Acc:HGNC:16629]	-1.1288518
RAPGEF5	Rap guanine nucleotide exchange factor 5 [Source:HGNC Symbol;Acc:HGNC:16862]	-1.0508581
RAPH1	ıs association (RalGDS/AF-6) and pleckstrin homology domains 1 [Source:HGNC Symbol;Acc:HGNC:1443	-1.3143218
RAPSN	receptor associated protein of the synapse [Source:HGNC Symbol;Acc:HGNC:9863]	-1.1202495
RARA	retinoic acid receptor alpha [Source:HGNC Symbol;Acc:HGNC:9864]	1.4296079
RARG	retinoic acid receptor gamma [Source:HGNC Symbol;Acc:HGNC:9866]	1.3348132
RASA4	RAS p21 protein activator 4 [Source:HGNC Symbol;Acc:HGNC:23181]	-1.24145195
RASAL2	RAS protein activator like 2 [Source:HGNC Symbol;Acc:HGNC:9874]	1.0799102
RASD1	ras related dexamethasone induced 1 [Source:HGNC Symbol;Acc:HGNC:15828]	-1.3344815
RASGEF1A	RasGEF domain family member 1A [Source:HGNC Symbol;Acc:HGNC:24246]	-1.1047115
RASGEF1C	RasGEF domain family member 1C [Source:HGNC Symbol;Acc:HGNC:27400]	-1.1015
RASL10B	RAS like family 10 member B [Source:HGNC Symbol;Acc:HGNC:30295]	1.3759699
RASL11B	RAS like family 11 member B [Source:HGNC Symbol;Acc:HGNC:23804]	1.1099755
RASSF1	Ras association domain family member 1 [Source:HGNC Symbol;Acc:HGNC:9882]	-1.3333485
RASSF2	Ras association domain family member 2 [Source:HGNC Symbol;Acc:HGNC:9883]	-1.5431599
RASSF7	Ras association domain family member 7 [Source:HGNC Symbol;Acc:HGNC:1166]	1.1906923
RBBP6	RB binding protein 6, ubiquitin ligase [Source:HGNC Symbol;Acc:HGNC:9889]	-1.2015558
RBKS	ribokinase [Source:HGNC Symbol;Acc:HGNC:30325]	-1.1442403
RBM12	RNA binding motif protein 12 [Source:HGNC Symbol;Acc:HGNC:9898]	1.2351298
RBM14	RNA binding motif protein 14 [Source:HGNC Symbol;Acc:HGNC:14219]	-1.1553562
RBM14-RBM4	RBM14-RBM4 readthrough [Source:HGNC Symbol;Acc:HGNC:38840]	1.0829846
RBM22	RNA binding motif protein 22 [Source:HGNC Symbol;Acc:HGNC:25503]	-1.3336023
RBM23	RNA binding motif protein 23 [Source:HGNC Symbol;Acc:HGNC:20155]	-1.194606
RBM27	RNA binding motif protein 27 [Source:HGNC Symbol;Acc:HGNC:29243]	1.5047793
RBM33	RNA binding motif protein 33 [Source:HGNC Symbol;Acc:HGNC:27223]	-1.118730667
RBM38	RNA binding motif protein 38 [Source:HGNC Symbol;Acc:HGNC:15818]	-1.6868588
RBM41	RNA binding motif protein 41 [Source:HGNC Symbol;Acc:HGNC:25617]	1.1215496
RBM44	RNA binding motif protein 44 [Source:HGNC Symbol;Acc:HGNC:24756]	-1.1119523
RBM45	RNA binding motif protein 45 [Source:HGNC Symbol;Acc:HGNC:24468]	1.1784016
RBM4B	RNA binding motif protein 4B [Source:HGNC Symbol;Acc:HGNC:28842]	1.172716
RBMY1B	RNA binding motif protein, Y-linked, family 1, member B [Source:HGNC Symbol;Acc:HGNC:23914]	1.0639497
RBP1	retinol binding protein 1 [Source:HGNC Symbol;Acc:HGNC:9919]	-1.2721735
RBP3	retinol binding protein 3 [Source:HGNC Symbol;Acc:HGNC:9921]	1.1597089
RBP4	retinol binding protein 4 [Source:HGNC Symbol;Acc:HGNC:9922]	1.1438098
RBPJ	mbination signal binding protein for immunoglobulin kappa J region [Source:HGNC Symbol;Acc:HGNC:5	1.4767572
RC3H2	ring finger and CCCH-type domains 2 [Source:HGNC Symbol;Acc:HGNC:21461]	1.631932
RCAN1	regulator of calcineurin 1 [Source:HGNC Symbol;Acc:HGNC:3040]	-1.6188667
RCAN2	regulator of calcineurin 2 [Source:HGNC Symbol;Acc:HGNC:3041]	-1.1152011
RCC2	regulator of chromosome condensation 2 [Source:HGNC Symbol;Acc:HGNC:30297]	-1.1822382
RCL1	RNA terminal phosphate cyclase like 1 [Source:HGNC Symbol;Acc:HGNC:17687]	1.421357
RCOR2	REST corepressor 2 [Source:HGNC Symbol;Acc:HGNC:27455]	1.2995554
RDX	radixin [Source:HGNC Symbol;Acc:HGNC:9944]	-1.2025448
RECQL4	RecQ like helicase 4 [Source:HGNC Symbol;Acc:HGNC:9949]	1.2493951
REEP3	receptor accessory protein 3 [Source:HGNC Symbol;Acc:HGNC:23711]	1.1288784
REG1A	regenerating family member 1 alpha [Source:HGNC Symbol;Acc:HGNC:9951]	-1.1179345
REG1B	regenerating family member 1 beta [Source:HGNC Symbol;Acc:HGNC:9952]	-1.1666206
REG3A	regenerating family member 3 alpha [Source:HGNC Symbol;Acc:HGNC:8601]	-1.1383637
REL	REL proto-oncogene, NF-kB subunit [Source:HGNC Symbol;Acc:HGNC:9954]	-1.4343128

RELA	RELA proto-oncogene, NF-kB subunit [Source:HGNC Symbol;Acc:HGNC:9955]	-1.4517273
RELL1	RELT like 1 [Source:HGNC Symbol;Acc:HGNC:27379]	-1.65665905
RELT	RELT tumor necrosis factor receptor [Source:HGNC Symbol;Acc:HGNC:13764]	-1.6056762
REPIN1	replication initiator 1 [Source:HGNC Symbol;Acc:HGNC:17922]	-1.146611
REPS1	RALBP1 associated Eps domain containing 1 [Source:HGNC Symbol;Acc:HGNC:15578]	1.4419588
REPS2	RALBP1 associated Eps domain containing 2 [Source:HGNC Symbol;Acc:HGNC:9963]	-1.126643
RERE	arginine-glutamic acid dipeptide repeats [Source:HGNC Symbol;Acc:HGNC:9965]	-1.2547234
RERG	RAS like estrogen regulated growth inhibitor [Source:HGNC Symbol;Acc:HGNC:15980]	-1.2908804
RETN	resistin [Source:HGNC Symbol;Acc:HGNC:20389]	1.1744215
RFC1	replication factor C subunit 1 [Source:HGNC Symbol;Acc:HGNC:9969]	1.5744472
RFC2	replication factor C subunit 2 [Source:HGNC Symbol;Acc:HGNC:9970]	-1.1823457
RFC3	replication factor C subunit 3 [Source:HGNC Symbol;Acc:HGNC:9971]	1.187361
RFC4	replication factor C subunit 4 [Source:HGNC Symbol;Acc:HGNC:9972]	1.2885237
RFFL	finger and FYVE-like domain containing E3 ubiquitin protein ligase [Source:HGNC Symbol;Acc:HGNC:24	-1.3523805
RFK	riboflavin kinase [Source:HGNC Symbol;Acc:HGNC:30324]	1.2307153
RFPL4B	ret finger protein like 4B [Source:HGNC Symbol;Acc:HGNC:33264]	1.0892528
RFT1	RFT1 homolog [Source:HGNC Symbol;Acc:HGNC:30220]	-1.0759046
RFTN1	raftlin, lipid raft linker 1 [Source:HGNC Symbol;Acc:HGNC:30278]	-1.2059972
RFX3	regulatory factor X3 [Source:HGNC Symbol;Acc:HGNC:9984]	-1.1510642
RFX5	regulatory factor X5 [Source:HGNC Symbol;Acc:HGNC:9986]	-1.5581308
RFX8	RFX family member 8, lacking RFX DNA binding domain [Source:HGNC Symbol;Acc:HGNC:37253]	-1.1445566
RG9MTD1	tRNA methyltransferase 10C, mitochondrial RNase P subunit	1.5117532
RGAG4	retrotransposon gag domain containing 4 [Source:HGNC Symbol;Acc:HGNC:29430]	-1.495805
RGMB	repulsive guidance molecule family member b [Source:HGNC Symbol;Acc:HGNC:26896]	-1.2085315
RGNEF	Rho guanine nucleotide exchange factor 28	1.2227029
RGPD1	RANBP2-like and GRIP domain containing 1 [Source:HGNC Symbol;Acc:HGNC:32414]	1.0972469
RGR	retinal G protein coupled receptor [Source:HGNC Symbol;Acc:HGNC:9990]	-1.0757536
RGS14	regulator of G-protein signaling 14 [Source:HGNC Symbol;Acc:HGNC:9996]	1.2254087
RGS16	regulator of G-protein signaling 16 [Source:HGNC Symbol;Acc:HGNC:9997]	-1.131075
RGS3	regulator of G-protein signaling 3 [Source:HGNC Symbol;Acc:HGNC:9999]	-1.8004479
RGS4	regulator of G-protein signaling 4 [Source:HGNC Symbol;Acc:HGNC:10000]	1.2698575
RGS6	regulator of G-protein signaling 6 [Source:HGNC Symbol;Acc:HGNC:10002]	1.0752645
RHBDF1	rhomboid 5 homolog 1 [Source:HGNC Symbol;Acc:HGNC:20561]	-1.3012015
RHBDF2	rhomboid 5 homolog 2 [Source:HGNC Symbol;Acc:HGNC:20788]	-2.2712052
RHBDL2	rhomboid like 2 [Source:HGNC Symbol;Acc:HGNC:16083]	-1.39831535
RHEBL1	Ras homolog enriched in brain like 1 [Source:HGNC Symbol;Acc:HGNC:21166]	-1.3419379
RHOB	ras homolog family member B [Source:HGNC Symbol;Acc:HGNC:668]	-1.52579835
RHOBTB1	Rho related BTB domain containing 1 [Source:HGNC Symbol;Acc:HGNC:18738]	1.3036652
RHOBTB2	Rho related BTB domain containing 2 [Source:HGNC Symbol;Acc:HGNC:18756]	1.724029
RHOBTB3	Rho related BTB domain containing 3 [Source:HGNC Symbol;Acc:HGNC:18757]	1.2937769
RHOF	ras homolog family member F, filopodia associated [Source:HGNC Symbol;Acc:HGNC:15703]	-1.2287095
RHOG	ras homolog family member G [Source:HGNC Symbol;Acc:HGNC:672]	-1.2134145
RHOV	ras homolog family member V [Source:HGNC Symbol;Acc:HGNC:18313]	-1.1789336
RIBC1	RIB43A domain with coiled-coils 1 [Source:HGNC Symbol;Acc:HGNC:26537]	-1.06599
RILPL2	Rab interacting lysosomal protein like 2 [Source:HGNC Symbol;Acc:HGNC:28787]	-1.1802472
RIMBP3	RIMS binding protein 3 [Source:HGNC Symbol;Acc:HGNC:29344]	1.5299807
RIMKLA	ribosomal modification protein rimK like family member A [Source:HGNC Symbol;Acc:HGNC:28725]	-1.0862191
RIMS3	regulating synaptic membrane exocytosis 3 [Source:HGNC Symbol;Acc:HGNC:21292]	-1.0589024
RIN1	Ras and Rab interactor 1 [Source:HGNC Symbol;Acc:HGNC:18749]	2.1459193
RIN2	Ras and Rab interactor 2 [Source:HGNC Symbol;Acc:HGNC:18750]	-2.1623456
RINL	Ras and Rab interactor like [Source:HGNC Symbol;Acc:HGNC:24795]	1.2944311
RIOK2	RIO kinase 2 [Source:HGNC Symbol;Acc:HGNC:18999]	1.417384
RIPK2	receptor interacting serine/threonine kinase 2 [Source:HGNC Symbol;Acc:HGNC:10020]	-4.11816
RIPK3	receptor interacting serine/threonine kinase 3 [Source:HGNC Symbol;Acc:HGNC:10021]	-1.0710562
RMI1	RecQ mediated genome instability 1 [Source:HGNC Symbol;Acc:HGNC:25764]	1.5255411
RNASE1	ribonuclease A family member 1, pancreatic [Source:HGNC Symbol;Acc:HGNC:10044]	1.1199994
RNASE7	ribonuclease A family member 7 [Source:HGNC Symbol;Acc:HGNC:19278]	-1.1191915
RNASE9	ribonuclease A family member 9 (inactive) [Source:HGNC Symbol;Acc:HGNC:20673]	1.0902382
RNASEH1	ribonuclease H1 [Source:HGNC Symbol;Acc:HGNC:18466]	-1.0920646
RND1	Rho family GTPase 1 [Source:HGNC Symbol;Acc:HGNC:18314]	-2.500136
RND3	Rho family GTPase 3 [Source:HGNC Symbol;Acc:HGNC:671]	2.1681395
RNF112	ring finger protein 112 [Source:HGNC Symbol;Acc:HGNC:12968]	1.1857562
RNF113A	ring finger protein 113A [Source:HGNC Symbol;Acc:HGNC:12974]	1.1455426
RNF113B	ring finger protein 113B [Source:HGNC Symbol;Acc:HGNC:17267]	1.1847668
RNF121	ring finger protein 121 [Source:HGNC Symbol;Acc:HGNC:21070]	-1.22433
RNF122	ring finger protein 122 [Source:HGNC Symbol;Acc:HGNC:21147]	-1.3467888
RNF126	ring finger protein 126 [Source:HGNC Symbol;Acc:HGNC:21151]	1.1591399
RNF13	ring finger protein 13 [Source:HGNC Symbol;Acc:HGNC:10057]	1.3462169
RNF130	ring finger protein 130 [Source:HGNC Symbol;Acc:HGNC:18280]	-1.1361308
RNF138	ring finger protein 138 [Source:HGNC Symbol;Acc:HGNC:17765]	1.2717316
RNF144B	ring finger protein 144B [Source:HGNC Symbol;Acc:HGNC:21578]	1.2810583
RNF145	ring finger protein 145 [Source:HGNC Symbol;Acc:HGNC:20853]	-1.0762523
RNF150	ring finger protein 150 [Source:HGNC Symbol;Acc:HGNC:23138]	1.0907253
RNF152	ring finger protein 152 [Source:HGNC Symbol;Acc:HGNC:26811]	1.7127714
RNF166	ring finger protein 166 [Source:HGNC Symbol;Acc:HGNC:28856]	1.2659713
RNF167	ring finger protein 167 [Source:HGNC Symbol;Acc:HGNC:24544]	1.25197575
RNF168	ring finger protein 168 [Source:HGNC Symbol;Acc:HGNC:26661]	1.0536687
RNF169	ring finger protein 169 [Source:HGNC Symbol;Acc:HGNC:26961]	1.1386145
RNF17	ring finger protein 17 [Source:HGNC Symbol;Acc:HGNC:10060]	1.1399482
RNF19B	ring finger protein 19B [Source:HGNC Symbol;Acc:HGNC:26886]	-1.4921013
RNF2	ring finger protein 2 [Source:HGNC Symbol;Acc:HGNC:10061]	1.2109352
RNF207	ring finger protein 207 [Source:HGNC Symbol;Acc:HGNC:32947]	-1.679059433
RNF212	ring finger protein 212 [Source:HGNC Symbol;Acc:HGNC:27729]	-1.1356851
RNF214	ring finger protein 214 [Source:HGNC Symbol;Acc:HGNC:25335]	-1.1261632
RNF216L	ring finger protein 216 pseudogene 1	-1.1690171
RNF219	ring finger protein 219 [Source:HGNC Symbol;Acc:HGNC:20308]	1.8364041

RNF220	ring finger protein 220 [Source:HGNC Symbol;Acc:HGNC:25552]	-1.1685171
RNF24	ring finger protein 24 [Source:HGNC Symbol;Acc:HGNC:13779]	-1.5495422
RNF40	ring finger protein 40 [Source:HGNC Symbol;Acc:HGNC:16867]	1.2349421
RNF44	ring finger protein 44 [Source:HGNC Symbol;Acc:HGNC:19180]	1.1546203
RNGTT	RNA guanylyltransferase and 5'-phosphatase [Source:HGNC Symbol;Acc:HGNC:10073]	1.2414063
RNH1	ribonuclease/angiogenin inhibitor 1 [Source:HGNC Symbol;Acc:HGNC:10074]	-1.1248939
RNPC3	RNA binding region (RNP1, RRM) containing 3 [Source:HGNC Symbol;Acc:HGNC:18666]	1.1122326
RNPEP	arginyl aminopeptidase [Source:HGNC Symbol;Acc:HGNC:10078]	1.0825993
ROBO2	roundabout guidance receptor 2 [Source:HGNC Symbol;Acc:HGNC:10250]	-1.0873826
ROR2	receptor tyrosine kinase like orphan receptor 2 [Source:HGNC Symbol;Acc:HGNC:10257]	-1.0713011
RP1L1	retinitis pigmentosa 1-like 1 [Source:HGNC Symbol;Acc:HGNC:15946]	-1.1441281
RPAIN	RPA interacting protein [Source:HGNC Symbol;Acc:HGNC:28641]	1.0552642
RPF1	ribosome production factor 1 homolog [Source:HGNC Symbol;Acc:HGNC:30350]	1.1810331
RPF2	ribosome production factor 2 homolog [Source:HGNC Symbol;Acc:HGNC:20870]	1.2993562
RPIA	ribose 5-phosphate isomerase A [Source:HGNC Symbol;Acc:HGNC:10297]	1.2135935
RPL23	ribosomal protein L23 [Source:HGNC Symbol;Acc:HGNC:10316]	-1.2232064
RPL23AP7	ribosomal protein L23a pseudogene 7 [Source:HGNC Symbol;Acc:HGNC:17336]	-1.1754092
RPL29	ribosomal protein L29 [Source:HGNC Symbol;Acc:HGNC:10331]	1.07397595
RPL29P2	ribosomal protein L29 pseudogene 2 [Source:HGNC Symbol;Acc:HGNC:17334]	1.1060773
RPL32P3	ribosomal protein L32 pseudogene 3 [Source:HGNC Symbol;Acc:HGNC:27024]	-1.1229272
RPL7AP37	ribosomal protein L7a pseudogene 37	1.1404786
RPP30	ribonuclease P/MRP subunit p30 [Source:HGNC Symbol;Acc:HGNC:17688]	-1.1435267
RPP38	ribonuclease P/MRP subunit p38 [Source:HGNC Symbol;Acc:HGNC:30329]	-1.1579903
RPRD1A	regulation of nuclear pre-mRNA domain containing 1A [Source:HGNC Symbol;Acc:HGNC:25560]	1.3453406
RPRD2	regulation of nuclear pre-mRNA domain containing 2 [Source:HGNC Symbol;Acc:HGNC:29039]	1.28579215
RPRML	reprimo like [Source:HGNC Symbol;Acc:HGNC:32422]	1.06371795
RPS14	ribosomal protein S14 [Source:HGNC Symbol;Acc:HGNC:10387]	-1.1270045
RPS2	ribosomal protein S2 [Source:HGNC Symbol;Acc:HGNC:10404]	1.1649574
RPS6KA2	ribosomal protein S6 kinase A2 [Source:HGNC Symbol;Acc:HGNC:10431]	-1.410639667
RPS6KA3	ribosomal protein S6 kinase A3 [Source:HGNC Symbol;Acc:HGNC:10432]	-1.0556641
RPS6KA5	ribosomal protein S6 kinase A5 [Source:HGNC Symbol;Acc:HGNC:10434]	1.4956322
RPS6KL1	ribosomal protein S6 kinase like 1 [Source:HGNC Symbol;Acc:HGNC:20222]	-1.3123137
RPSAP52	ribosomal protein SA pseudogene 52 [Source:HGNC Symbol;Acc:HGNC:35752]	-1.1133547
RPUSD4	RNA pseudouridylate synthase domain containing 4 [Source:HGNC Symbol;Acc:HGNC:25898]	-1.1648445
RQCD1	CCR4-NOT transcription complex subunit 9	-1.2591312
RRAD	AD, Ras related glycolysis inhibitor and calcium channel regulator [Source:HGNC Symbol;Acc:HGNC:104	-2.3344486
RRAGA	Ras related GTP binding A [Source:HGNC Symbol;Acc:HGNC:16963]	-1.3039336
RRAGB	Ras related GTP binding B [Source:HGNC Symbol;Acc:HGNC:19901]	1.1105382
RRAS2	related RAS viral (r-ras) oncogene homolog 2 [Source:HGNC Symbol;Acc:HGNC:17271]	1.2920831
RRBP1	ribosome binding protein 1 [Source:HGNC Symbol;Acc:HGNC:10448]	-1.3017266
RRM1	ribonucleotide reductase catalytic subunit M1 [Source:HGNC Symbol;Acc:HGNC:10451]	1.2725685
RRN3	RRN3 homolog, RNA polymerase I transcription factor [Source:HGNC Symbol;Acc:HGNC:30346]	-1.1194341
RRP8	bosomal RNA processing 8, methyltransferase, homolog (yeast) [Source:HGNC Symbol;Acc:HGNC:2903(	1.1276213
RRS1	ribosome biogenesis regulator homolog [Source:HGNC Symbol;Acc:HGNC:17083]	1.281741
RSAD1	radical S-adenosyl methionine domain containing 1 [Source:HGNC Symbol;Acc:HGNC:25634]	1.3272914
RSBN1L	round spermatid basic protein 1 like [Source:HGNC Symbol;Acc:HGNC:24765]	1.3462671
RSPH1	radial spoke head 1 homolog [Source:HGNC Symbol;Acc:HGNC:12371]	-1.0606655
RSPH4A	radial spoke head 4 homolog A [Source:HGNC Symbol;Acc:HGNC:21558]	1.1570005
RSPO3	R-spondin 3 [Source:HGNC Symbol;Acc:HGNC:20866]	-1.2142001
RTBDN	retbindin [Source:HGNC Symbol;Acc:HGNC:30310]	-1.1535487
RTDR1	radial spoke head 14 homolog	-1.1295856
RTKN	rhotekin [Source:HGNC Symbol;Acc:HGNC:10466]	1.174732
RTKN2	rhotekin 2 [Source:HGNC Symbol;Acc:HGNC:19364]	-1.0647374
RTN3	reticulon 3 [Source:HGNC Symbol;Acc:HGNC:10469]	-1.2339903
RTN4IP1	reticulon 4 interacting protein 1 [Source:HGNC Symbol;Acc:HGNC:18647]	1.1477271
RTN4RL2	reticulon 4 receptor-like 2 [Source:HGNC Symbol;Acc:HGNC:23053]	-1.1881436
RTP4	receptor transporter protein 4 [Source:HGNC Symbol;Acc:HGNC:23992]	-1.7279081
RUFY4	RUN and FYVE domain containing 4 [Source:HGNC Symbol;Acc:HGNC:24804]	-1.1594365
RUNDC3B	RUN domain containing 3B [Source:HGNC Symbol;Acc:HGNC:30286]	1.1152334
RUNX1	runt related transcription factor 1 [Source:HGNC Symbol;Acc:HGNC:10471]	-1.22274345
RUNX1T1	RUNX1 translocation partner 1 [Source:HGNC Symbol;Acc:HGNC:1535]	2.3893292
RUVBL2	RuvB like AAA ATPase 2 [Source:HGNC Symbol;Acc:HGNC:10475]	-1.1188267
RWDD1	RWD domain containing 1 [Source:HGNC Symbol;Acc:HGNC:20993]	1.2436431
RWDD2A	RWD domain containing 2A [Source:HGNC Symbol;Acc:HGNC:21385]	-1.1700553
RXRA	retinoid X receptor alpha [Source:HGNC Symbol;Acc:HGNC:10477]	1.088242
RXRB	retinoid X receptor beta [Source:HGNC Symbol;Acc:HGNC:10478]	1.1835409
RYR3	ryanodine receptor 3 [Source:HGNC Symbol;Acc:HGNC:10485]	-1.1246238
S100A12	S100 calcium binding protein A12 [Source:HGNC Symbol;Acc:HGNC:10489]	-1.0570798
S100A3	S100 calcium binding protein A3 [Source:HGNC Symbol;Acc:HGNC:10493]	-5.924905
S100G	S100 calcium binding protein G [Source:HGNC Symbol;Acc:HGNC:1436]	1.099018
S100P	S100 calcium binding protein P [Source:HGNC Symbol;Acc:HGNC:10504]	-1.1783785
S1PR1	sphingosine-1-phosphate receptor 1 [Source:HGNC Symbol;Acc:HGNC:3165]	-1.8234551
S1PR2	sphingosine-1-phosphate receptor 2 [Source:HGNC Symbol;Acc:HGNC:3169]	-1.8879114
SAA1	serum amyloid A1 [Source:HGNC Symbol;Acc:HGNC:10513]	-1.0723135
SAMD12	sterile alpha motif domain containing 12 [Source:HGNC Symbol;Acc:HGNC:31750]	1.1758848
SAMD14	sterile alpha motif domain containing 14 [Source:HGNC Symbol;Acc:HGNC:27312]	-1.8836696
SAMD4A	sterile alpha motif domain containing 4A [Source:HGNC Symbol;Acc:HGNC:23023]	-2.029286833
SAMD4B	sterile alpha motif domain containing 4B [Source:HGNC Symbol;Acc:HGNC:25492]	1.3907033
SAMD8	sterile alpha motif domain containing 8 [Source:HGNC Symbol;Acc:HGNC:26320]	-1.2904165
SAMD9	sterile alpha motif domain containing 9 [Source:HGNC Symbol;Acc:HGNC:1348]	1.1131039
SAMM50	SAMM50 sorting and assembly machinery component [Source:HGNC Symbol;Acc:HGNC:24276]	1.09371
SAP30BP	SAP30 binding protein [Source:HGNC Symbol;Acc:HGNC:30785]	-1.153906
SARDH	sarcosine dehydrogenase [Source:HGNC Symbol;Acc:HGNC:10536]	-1.194814
SARS	seryl-tRNA synthetase [Source:HGNC Symbol;Acc:HGNC:10537]	1.0871358
SASH1	SAM and SH3 domain containing 1 [Source:HGNC Symbol;Acc:HGNC:19182]	1.6712207
SASS6	SAS-6 centriolar assembly protein [Source:HGNC Symbol;Acc:HGNC:25403]	1.3084991

SAT1	spermidine/spermine N1-acetyltransferase 1 [Source:HGNC Symbol;Acc:HGNC:10540]	-1.714093733
SAT2	spermidine/spermine N1-acetyltransferase family member 2 [Source:HGNC Symbol;Acc:HGNC:23160]	1.2109832
SATB1	SATB homeobox 1 [Source:HGNC Symbol;Acc:HGNC:10541]	1.096392
SAV1	salvador family WW domain containing protein 1 [Source:HGNC Symbol;Acc:HGNC:17795]	-2.4822557
SBK1	SH3 domain binding kinase 1 [Source:HGNC Symbol;Acc:HGNC:17699]	1.0506829
SBNO2	strawberry notch homolog 2 [Source:HGNC Symbol;Acc:HGNC:29158]	-1.8019269
SBSN	suprabasin [Source:HGNC Symbol;Acc:HGNC:24950]	1.0709168
SCAF1	SR-related CTD associated factor 1 [Source:HGNC Symbol;Acc:HGNC:30403]	1.3098054
SCAMP2	secretory carrier membrane protein 2 [Source:HGNC Symbol;Acc:HGNC:10564]	-1.1602647
SCAMP3	secretory carrier membrane protein 3 [Source:HGNC Symbol;Acc:HGNC:10565]	-1.1221188
SCAND2	SCAN domain containing 2 pseudogene	-1.1989627
SCARA3	scavenger receptor class A member 3 [Source:HGNC Symbol;Acc:HGNC:19000]	-1.0940439
SCARB1	scavenger receptor class B member 1 [Source:HGNC Symbol;Acc:HGNC:1664]	1.2331651
SCARB2	scavenger receptor class B member 2 [Source:HGNC Symbol;Acc:HGNC:1665]	1.3289002
SCARF1	scavenger receptor class F member 1 [Source:HGNC Symbol;Acc:HGNC:16820]	-1.4201542
SCARNA9L	small Cajal body-specific RNA 9-like	-1.1707532
SCD5	stearoyl-CoA desaturase 5 [Source:HGNC Symbol;Acc:HGNC:21088]	-1.1308806
SCG2	secretogranin II [Source:HGNC Symbol;Acc:HGNC:10575]	1.0515409
SCGB2A2	secretoglobin family 2A member 2 [Source:HGNC Symbol;Acc:HGNC:7050]	1.1288807
SCLY	selenocysteine lyase [Source:HGNC Symbol;Acc:HGNC:18161]	1.1541694
SCML1	sex comb on midleg-like 1 (Drosophila) [Source:HGNC Symbol;Acc:HGNC:10580]	1.1293019
SCML4	sex comb on midleg-like 4 (Drosophila) [Source:HGNC Symbol;Acc:HGNC:21397]	1.0655712
SCN1B	sodium voltage-gated channel beta subunit 1 [Source:HGNC Symbol;Acc:HGNC:10586]	1.4391778
SCN2B	sodium voltage-gated channel beta subunit 2 [Source:HGNC Symbol;Acc:HGNC:10589]	-1.4855578
SCN4A	sodium voltage-gated channel alpha subunit 4 [Source:HGNC Symbol;Acc:HGNC:10591]	-1.1423026
SCN4B	sodium voltage-gated channel beta subunit 4 [Source:HGNC Symbol;Acc:HGNC:10592]	1.0738896
SCN5A	sodium voltage-gated channel alpha subunit 5 [Source:HGNC Symbol;Acc:HGNC:10593]	1.2273055
SCN8A	sodium voltage-gated channel alpha subunit 8 [Source:HGNC Symbol;Acc:HGNC:10596]	-1.156236
SCNN1B	sodium channel epithelial 1 beta subunit [Source:HGNC Symbol;Acc:HGNC:10600]	1.1492671
SCO1	SCO1 cytochrome c oxidase assembly protein [Source:HGNC Symbol;Acc:HGNC:10603]	1.1552103
SCRN3	secernin 3 [Source:HGNC Symbol;Acc:HGNC:30382]	1.086931
SCRT1	scratch family transcriptional repressor 1 [Source:HGNC Symbol;Acc:HGNC:15950]	1.0859209
SCYL1	SCY1 like pseudokinase 1 [Source:HGNC Symbol;Acc:HGNC:14372]	-1.1542336
SCYL2	SCY1 like pseudokinase 2 [Source:HGNC Symbol;Acc:HGNC:19286]	1.1025493
SDC1	syndecan 1 [Source:HGNC Symbol;Acc:HGNC:10658]	1.2037464
SDC4	syndecan 4 [Source:HGNC Symbol;Acc:HGNC:10661]	-3.5583906
SDCCAG8	serologically defined colon cancer antigen 8 [Source:HGNC Symbol;Acc:HGNC:10671]	1.18331875
SDHA	succinate dehydrogenase complex flavoprotein subunit A [Source:HGNC Symbol;Acc:HGNC:10680]	-1.0608532
SDHAF2	succinate dehydrogenase complex assembly factor 2 [Source:HGNC Symbol;Acc:HGNC:26034]	-1.0815994
SDHAF3	succinate dehydrogenase complex assembly factor 3	1.2237418
SDHD	succinate dehydrogenase complex subunit D [Source:HGNC Symbol;Acc:HGNC:10683]	1.1616831
SDK2	sidekick cell adhesion molecule 2 [Source:HGNC Symbol;Acc:HGNC:19308]	1.087193
SEBOX	SEBOX homeobox [Source:HGNC Symbol;Acc:HGNC:32942]	-1.1378819
SEC11C	SEC11 homolog C, signal peptidase complex subunit [Source:HGNC Symbol;Acc:HGNC:23400]	-1.1608229
SEC14L2	SEC14 like lipid binding 2 [Source:HGNC Symbol;Acc:HGNC:10699]	-1.3377573
SEC16B	SEC16 homolog B, endoplasmic reticulum export factor [Source:HGNC Symbol;Acc:HGNC:30301]	1.0731544
SEC22C	SEC22 homolog C, vesicle trafficking protein [Source:HGNC Symbol;Acc:HGNC:16828]	-1.1578892
SEC23B	Sec23 homolog B, coat complex II component [Source:HGNC Symbol;Acc:HGNC:10702]	-1.32401675
SEC24A	SEC24 homolog A, COPII coat complex component [Source:HGNC Symbol;Acc:HGNC:10703]	-1.4126457
SEC31B	SEC31 homolog B, COPII coat complex component [Source:HGNC Symbol;Acc:HGNC:23197]	-1.1160028
SEC62	SEC62 homolog, preprotein translocation factor [Source:HGNC Symbol;Acc:HGNC:11846]	1.2161474
SECISBP2L	SECIS binding protein 2 like [Source:HGNC Symbol;Acc:HGNC:28997]	1.0905099
SEL1L	SEL1L ERAD E3 ligase adaptor subunit [Source:HGNC Symbol;Acc:HGNC:10717]	1.18132965
SELE	selectin E [Source:HGNC Symbol;Acc:HGNC:10718]	-50.88709
SELENBP1	selenium binding protein 1 [Source:HGNC Symbol;Acc:HGNC:10719]	-1.1793039
SELM	selenoprotein M	-1.6307195
SELT	selenoprotein T	1.3112134
SEMA3F	semaphorin 3F [Source:HGNC Symbol;Acc:HGNC:10728]	1.4922091
SEMA3G	semaphorin 3G [Source:HGNC Symbol;Acc:HGNC:30400]	1.2153898
SEMA4A	semaphorin 4A [Source:HGNC Symbol;Acc:HGNC:10729]	-1.2179706
SEMA4B	semaphorin 4B [Source:HGNC Symbol;Acc:HGNC:10730]	-1.3499597
SEMA4C	semaphorin 4C [Source:HGNC Symbol;Acc:HGNC:10731]	-1.5851582
SEMA4D	semaphorin 4D [Source:HGNC Symbol;Acc:HGNC:10732]	1.1410433
SEMA6B	semaphorin 6B [Source:HGNC Symbol;Acc:HGNC:10739]	1.327658
SEMA6C	semaphorin 6C [Source:HGNC Symbol;Acc:HGNC:10740]	1.3072208
SEMA6D	semaphorin 6D [Source:HGNC Symbol;Acc:HGNC:16770]	1.1215733
SENP1	SUMO1/sentrin specific peptidase 1 [Source:HGNC Symbol;Acc:HGNC:17927]	1.2220706
SEPHS2	selenophosphate synthetase 2 [Source:HGNC Symbol;Acc:HGNC:19686]	1.2815186
SEPN1	selenoprotein N, 1 [Source:HGNC Symbol;Acc:HGNC:15999]	1.1940587
SEPP1	selenoprotein P, plasma, 1 [Source:HGNC Symbol;Acc:HGNC:10751]	-1.1888655
SEPSECS	ep (O-phosphoserine) tRNA:Sec (selenocysteine) tRNA synthase [Source:HGNC Symbol;Acc:HGNC:3060!	-1.0557537
SEPW1	selenoprotein W, 1 [Source:HGNC Symbol;Acc:HGNC:10752]	1.2385434
SERF1A	small EDRK-rich factor 1A [Source:HGNC Symbol;Acc:HGNC:10755]	-1.353089
SERINC5	serine incorporator 5 [Source:HGNC Symbol;Acc:HGNC:18825]	-1.1235372
SERPINA1	serpin family A member 1 [Source:HGNC Symbol;Acc:HGNC:8941]	-1.222883
SERPINA3	serpin family A member 3 [Source:HGNC Symbol;Acc:HGNC:16]	-1.1721622
SERPINB13	serpin family B member 13 [Source:HGNC Symbol;Acc:HGNC:8944]	-1.2347697
SERPINB2	serpin family B member 2 [Source:HGNC Symbol;Acc:HGNC:8584]	-1.6792448
SERPINB8	serpin family B member 8 [Source:HGNC Symbol;Acc:HGNC:8952]	-1.6072087
SERPIND1	serpin family D member 1 [Source:HGNC Symbol;Acc:HGNC:4838]	-1.6149043
SERPINE1	serpin family E member 1 [Source:HGNC Symbol;Acc:HGNC:8583]	-1.8878003
SERPINH1	serpin family H member 1 [Source:HGNC Symbol;Acc:HGNC:1546]	1.0907226
SERTAD3	SERTA domain containing 3 [Source:HGNC Symbol;Acc:HGNC:17931]	1.233979
SERTAD4	SERTA domain containing 4 [Source:HGNC Symbol;Acc:HGNC:25236]	1.382674
SESN2	sestrin 2 [Source:HGNC Symbol;Acc:HGNC:20746]	-1.1368705
SETD5	SET domain containing 5 [Source:HGNC Symbol;Acc:HGNC:25566]	-1.0661584

SETD7	SET domain containing lysine methyltransferase 7 [Source:HGNC Symbol;Acc:HGNC:30412]	1.2369637
SETDB2	SET domain bifurcated 2 [Source:HGNC Symbol;Acc:HGNC:20263]	1.644999
SF1	splicing factor 1 [Source:HGNC Symbol;Acc:HGNC:12950]	1.087913
SF3A1	splicing factor 3a subunit 1 [Source:HGNC Symbol;Acc:HGNC:10765]	-1.2357739
SF3B4	splicing factor 3b subunit 4 [Source:HGNC Symbol;Acc:HGNC:10771]	1.6588527
SFI1	SFI1 centrin binding protein [Source:HGNC Symbol;Acc:HGNC:29064]	1.1352545
SGK1	serum/glucocorticoid regulated kinase 1 [Source:HGNC Symbol;Acc:HGNC:10810]	-1.4778552
SGK223	PEAK1 related kinase activating pseudokinase 1	1.915186
SGPL1	sphingosine-1-phosphate lyase 1 [Source:HGNC Symbol;Acc:HGNC:10817]	-1.8910965
SH2B2	SH2B adaptor protein 2 [Source:HGNC Symbol;Acc:HGNC:17381]	1.3455522
SH2D7	SH2 domain containing 7 [Source:HGNC Symbol;Acc:HGNC:34549]	1.1383865
SH3D21	SH3 domain containing 21 [Source:HGNC Symbol;Acc:HGNC:26236]	-1.1510593
SH3GL1	SH3 domain containing GRB2 like 1, endophilin A2 [Source:HGNC Symbol;Acc:HGNC:10830]	1.2145821
SH3GL1P2	H3 domain containing GRB2 like 1, endophilin A2 pseudogene 2 [Source:HGNC Symbol;Acc:HGNC:1083	1.34683085
SH3GL1P3	H3 domain containing GRB2 like 1, endophilin A2 pseudogene 3 [Source:HGNC Symbol;Acc:HGNC:1083	1.4997866
SH3PXD2B	SH3 and PX domains 2B [Source:HGNC Symbol;Acc:HGNC:29242]	1.2531106
SH3RF3	SH3 domain containing ring finger 3 [Source:HGNC Symbol;Acc:HGNC:24699]	-1.42392935
SH3TC1	SH3 domain and tetratricopeptide repeats 1 [Source:HGNC Symbol;Acc:HGNC:26009]	-1.651052033
SH3TC2	SH3 domain and tetratricopeptide repeats 2 [Source:HGNC Symbol;Acc:HGNC:29427]	1.223008
SHANK3	SH3 and multiple ankyrin repeat domains 3 [Source:HGNC Symbol;Acc:HGNC:14294]	1.4359393
SHARPIN	SHANK associated RH domain interactor [Source:HGNC Symbol;Acc:HGNC:25321]	1.2940657
SHB	SH2 domain containing adaptor protein B [Source:HGNC Symbol;Acc:HGNC:10838]	-2.0696073
SHBG	sex hormone binding globulin [Source:HGNC Symbol;Acc:HGNC:10839]	-1.0776646
SHC3	SHC adaptor protein 3 [Source:HGNC Symbol;Acc:HGNC:18181]	1.1981919
SHE	Src homology 2 domain containing E [Source:HGNC Symbol;Acc:HGNC:27004]	1.6982174
SHISA2	shisa family member 2 [Source:HGNC Symbol;Acc:HGNC:20366]	1.3395536
SHISA3	shisa family member 3 [Source:HGNC Symbol;Acc:HGNC:25159]	1.4962261
SHKBP1	SH3KBP1 binding protein 1 [Source:HGNC Symbol;Acc:HGNC:19214]	1.2659011
SHMT1	serine hydroxymethyltransferase 1 [Source:HGNC Symbol;Acc:HGNC:10850]	-1.0853193
SHPRH	SNF2 histone linker PHD RING helicase [Source:HGNC Symbol;Acc:HGNC:19336]	1.5827359
SHQ1	SHQ1, H/ACA ribonucleoprotein assembly factor [Source:HGNC Symbol;Acc:HGNC:25543]	-1.1409504
SHROOM2	shroom family member 2 [Source:HGNC Symbol;Acc:HGNC:630]	-1.5547401
SHROOM3	shroom family member 3 [Source:HGNC Symbol;Acc:HGNC:30422]	1.1722264
SIAH1	siah E3 ubiquitin protein ligase 1 [Source:HGNC Symbol;Acc:HGNC:10857]	-1.2271664
SIAH2	siah E3 ubiquitin protein ligase 2 [Source:HGNC Symbol;Acc:HGNC:10858]	-1.0817205
SIGLEC1	sialic acid binding Ig like lectin 1 [Source:HGNC Symbol;Acc:HGNC:11127]	1.088942
SIGLEC16	sialic acid binding Ig like lectin 16 (gene/pseudogene) [Source:HGNC Symbol;Acc:HGNC:24851]	-1.2795612
SIGLEC5	sialic acid binding Ig like lectin 5 [Source:HGNC Symbol;Acc:HGNC:10874]	-1.0702451
SIGMAR1	sigma non-opioid intracellular receptor 1 [Source:HGNC Symbol;Acc:HGNC:8157]	1.1126697
SIK1	salt inducible kinase 1 [Source:HGNC Symbol;Acc:HGNC:11142]	-1.4303672
SIK2	salt inducible kinase 2 [Source:HGNC Symbol;Acc:HGNC:21680]	-1.5103676
SIM1	single-minded family bHLH transcription factor 1 [Source:HGNC Symbol;Acc:HGNC:10882]	-1.1851674
SIN3A	SIN3 transcription regulator family member A [Source:HGNC Symbol;Acc:HGNC:19353]	-1.3092691
SIP1	SR-related CTD associated factor 11	1.1136341
SIRPA	signal regulatory protein alpha [Source:HGNC Symbol;Acc:HGNC:9662]	1.1174405
SIRPB1	signal regulatory protein beta 1 [Source:HGNC Symbol;Acc:HGNC:15928]	-1.1935005
SIRT2	sirtuin 2 [Source:HGNC Symbol;Acc:HGNC:10886]	1.4460858
SIX5	SIX homeobox 5 [Source:HGNC Symbol;Acc:HGNC:10891]	-1.3978463
SKA1	spindle and kinetochore associated complex subunit 1 [Source:HGNC Symbol;Acc:HGNC:28109]	-1.3619052
SKA3	spindle and kinetochore associated complex subunit 3 [Source:HGNC Symbol;Acc:HGNC:20262]	1.2582046
SKIV2L2	Ski2 like RNA helicase 2 [Source:HGNC Symbol;Acc:HGNC:18734]	1.5425587
SKP1	S-phase kinase-associated protein 1 [Source:HGNC Symbol;Acc:HGNC:10899]	1.2975101
SLAMF1	signaling lymphocytic activation molecule family member 1 [Source:HGNC Symbol;Acc:HGNC:10903]	-1.4317615
SLAMF8	SLAM family member 8 [Source:HGNC Symbol;Acc:HGNC:21391]	-1.1951581
SLC10A4	solute carrier family 10 member 4 [Source:HGNC Symbol;Acc:HGNC:22980]	1.0998561
SLC11A2	solute carrier family 11 member 2 [Source:HGNC Symbol;Acc:HGNC:10908]	-1.2158426
SLC12A7	solute carrier family 12 member 7 [Source:HGNC Symbol;Acc:HGNC:10915]	-3.9705856
SLC12A9	solute carrier family 12 member 9 [Source:HGNC Symbol;Acc:HGNC:17435]	1.3560034
SLC13A2	solute carrier family 13 member 2 [Source:HGNC Symbol;Acc:HGNC:10917]	-1.1065593
SLC14A1	solute carrier family 14 member 1 (Kidd blood group) [Source:HGNC Symbol;Acc:HGNC:10918]	1.128409
SLC14A2	solute carrier family 14 member 2 [Source:HGNC Symbol;Acc:HGNC:10919]	-1.2378604
SLC15A3	solute carrier family 15 member 3 [Source:HGNC Symbol;Acc:HGNC:18068]	-2.4775558
SLC15A4	solute carrier family 15 member 4 [Source:HGNC Symbol;Acc:HGNC:23090]	-1.6239801
SLC16A4	solute carrier family 16 member 4 [Source:HGNC Symbol;Acc:HGNC:10925]	-1.117493
SLC16A9	solute carrier family 16 member 9 [Source:HGNC Symbol;Acc:HGNC:23520]	-1.2664418
SLC17A2	solute carrier family 17 member 2 [Source:HGNC Symbol;Acc:HGNC:10930]	1.0911045
SLC17A3	solute carrier family 17 member 3 [Source:HGNC Symbol;Acc:HGNC:10931]	-1.087898
SLC17A5	solute carrier family 17 member 5 [Source:HGNC Symbol;Acc:HGNC:10933]	-1.3112874
SLC18A3	solute carrier family 18 member A3 [Source:HGNC Symbol;Acc:HGNC:10936]	-1.2391266
SLC1A1	solute carrier family 1 member 1 [Source:HGNC Symbol;Acc:HGNC:10939]	1.4851477
SLC1A2	solute carrier family 1 member 2 [Source:HGNC Symbol;Acc:HGNC:10940]	1.159159
SLC1A3	solute carrier family 1 member 3 [Source:HGNC Symbol;Acc:HGNC:10941]	-1.1486062
SLC20A1	solute carrier family 20 member 1 [Source:HGNC Symbol;Acc:HGNC:10946]	1.8247638
SLC20A2	solute carrier family 20 member 2 [Source:HGNC Symbol;Acc:HGNC:10947]	1.1605104
SLC22A18AS	solute carrier family 22 member 18 antisense [Source:HGNC Symbol;Acc:HGNC:10965]	1.1776129
SLC22A2	solute carrier family 22 member 2 [Source:HGNC Symbol;Acc:HGNC:10966]	1.1039569
SLC22A20	solute carrier family 22 member 20 [Source:HGNC Symbol;Acc:HGNC:29867]	-1.19168975
SLC22A23	solute carrier family 22 member 23 [Source:HGNC Symbol;Acc:HGNC:21106]	-1.3922028
SLC22A31	solute carrier family 22 member 31 [Source:HGNC Symbol;Acc:HGNC:27091]	-1.1529076
SLC22A4	solute carrier family 22 member 4 [Source:HGNC Symbol;Acc:HGNC:10968]	-1.0861019
SLC23A3	solute carrier family 23 member 3 [Source:HGNC Symbol;Acc:HGNC:20601]	-1.13349775
SLC24A1	solute carrier family 24 member 1 [Source:HGNC Symbol;Acc:HGNC:10975]	-1.0706054
SLC24A4	solute carrier family 24 member 4 [Source:HGNC Symbol;Acc:HGNC:10978]	-1.1145053
SLC24A6	solute carrier family 8 member B1	-1.2741559
SLC25A19	solute carrier family 25 member 19 [Source:HGNC Symbol;Acc:HGNC:14409]	-1.1865109
SLC25A23	solute carrier family 25 member 23 [Source:HGNC Symbol;Acc:HGNC:19375]	1.1812223

SLC25A26	solute carrier family 25 member 26 [Source:HGNC Symbol;Acc:HGNC:20661]	-1.0882463
SLC25A28	solute carrier family 25 member 28 [Source:HGNC Symbol;Acc:HGNC:23472]	-1.3696846
SLC25A30	solute carrier family 25 member 30 [Source:HGNC Symbol;Acc:HGNC:27371]	1.6308022
SLC25A32	solute carrier family 25 member 32 [Source:HGNC Symbol;Acc:HGNC:29683]	-1.3701208
SLC25A34	solute carrier family 25 member 34 [Source:HGNC Symbol;Acc:HGNC:27653]	-1.3937715
SLC25A37	solute carrier family 25 member 37 [Source:HGNC Symbol;Acc:HGNC:29786]	-1.370395
SLC25A43	solute carrier family 25 member 43 [Source:HGNC Symbol;Acc:HGNC:30557]	-1.2309555
SLC25A47	solute carrier family 25 member 47 [Source:HGNC Symbol;Acc:HGNC:20115]	-1.1311978
SLC26A11	solute carrier family 26 member 11 [Source:HGNC Symbol;Acc:HGNC:14471]	-1.3529459
SLC26A2	solute carrier family 26 member 2 [Source:HGNC Symbol;Acc:HGNC:10994]	1.273453
SLC26A5	solute carrier family 26 member 5 [Source:HGNC Symbol;Acc:HGNC:9359]	1.0908985
SLC26A7	solute carrier family 26 member 7 [Source:HGNC Symbol;Acc:HGNC:14467]	1.1172166
SLC26A9	solute carrier family 26 member 9 [Source:HGNC Symbol;Acc:HGNC:14469]	-1.2052923
SLC28A1	solute carrier family 28 member 1 [Source:HGNC Symbol;Acc:HGNC:11001]	1.0532773
SLC29A1	solute carrier family 29 member 1 (Augustine blood group) [Source:HGNC Symbol;Acc:HGNC:11003]	1.1237997
SLC29A2	solute carrier family 29 member 2 [Source:HGNC Symbol;Acc:HGNC:11004]	1.0677754
SLC29A4	solute carrier family 29 member 4 [Source:HGNC Symbol;Acc:HGNC:23097]	1.1062043
SLC2A13	solute carrier family 2 member 13 [Source:HGNC Symbol;Acc:HGNC:15956]	1.0709485
SLC2A5	solute carrier family 2 member 5 [Source:HGNC Symbol;Acc:HGNC:11010]	-1.2770567
SLC2A6	solute carrier family 2 member 6 [Source:HGNC Symbol;Acc:HGNC:11011]	-2.38111955
SLC30A1	solute carrier family 30 member 1 [Source:HGNC Symbol;Acc:HGNC:11012]	1.1185017
SLC30A2	solute carrier family 30 member 2 [Source:HGNC Symbol;Acc:HGNC:11013]	-1.2445333
SLC30A7	solute carrier family 30 member 7 [Source:HGNC Symbol;Acc:HGNC:19306]	-1.1713142
SLC31A2	solute carrier family 31 member 2 [Source:HGNC Symbol;Acc:HGNC:11017]	-3.8152761
SLC34A1	solute carrier family 34 member 1 [Source:HGNC Symbol;Acc:HGNC:11019]	-1.0865018
SLC35D2	solute carrier family 35 member D2 [Source:HGNC Symbol;Acc:HGNC:20799]	-1.1075573
SLC35E1	solute carrier family 35 member E1 [Source:HGNC Symbol;Acc:HGNC:20803]	-1.16162115
SLC35E2	solute carrier family 35 member E2 [Source:HGNC Symbol;Acc:HGNC:20863]	1.1610974
SLC35E3	solute carrier family 35 member E3 [Source:HGNC Symbol;Acc:HGNC:20864]	-1.1705283
SLC35F5	solute carrier family 35 member F5 [Source:HGNC Symbol;Acc:HGNC:23617]	1.0794834
SLC36A3	solute carrier family 36 member 3 [Source:HGNC Symbol;Acc:HGNC:19659]	1.1321479
SLC37A1	solute carrier family 37 member 1 [Source:HGNC Symbol;Acc:HGNC:11024]	-1.6429214
SLC38A10	solute carrier family 38 member 10 [Source:HGNC Symbol;Acc:HGNC:28237]	-1.1541848
SLC38A2	solute carrier family 38 member 2 [Source:HGNC Symbol;Acc:HGNC:13448]	1.81909475
SLC39A1	solute carrier family 39 member 1 [Source:HGNC Symbol;Acc:HGNC:12876]	-1.283497
SLC39A11	solute carrier family 39 member 11 [Source:HGNC Symbol;Acc:HGNC:14463]	-1.1256454
SLC39A2	solute carrier family 39 member 2 [Source:HGNC Symbol;Acc:HGNC:17127]	-1.1966377
SLC39A7	solute carrier family 39 member 7 [Source:HGNC Symbol;Acc:HGNC:4927]	1.21949
SLC3A1	solute carrier family 3 member 1 [Source:HGNC Symbol;Acc:HGNC:11025]	1.2051109
SLC3A2	solute carrier family 3 member 2 [Source:HGNC Symbol;Acc:HGNC:11026]	-1.1652458
SLC41A1	solute carrier family 41 member 1 [Source:HGNC Symbol;Acc:HGNC:19429]	-1.8855487
SLC41A2	solute carrier family 41 member 2 [Source:HGNC Symbol;Acc:HGNC:31045]	-1.5176158
SLC43A1	solute carrier family 43 member 1 [Source:HGNC Symbol;Acc:HGNC:9225]	1.3081092
SLC43A2	solute carrier family 43 member 2 [Source:HGNC Symbol;Acc:HGNC:23087]	-1.2620823
SLC44A1	solute carrier family 44 member 1 [Source:HGNC Symbol;Acc:HGNC:18798]	1.3095196
SLC44A3	solute carrier family 44 member 3 [Source:HGNC Symbol;Acc:HGNC:28689]	-1.0501009
SLC45A1	solute carrier family 45 member 1 [Source:HGNC Symbol;Acc:HGNC:17939]	1.0982971
SLC45A3	solute carrier family 45 member 3 [Source:HGNC Symbol;Acc:HGNC:8642]	2.0233
SLC46A1	solute carrier family 46 member 1 [Source:HGNC Symbol;Acc:HGNC:30521]	1.2280153
SLC47A1	solute carrier family 47 member 1 [Source:HGNC Symbol;Acc:HGNC:25588]	-1.077101
SLC47A2	solute carrier family 47 member 2 [Source:HGNC Symbol;Acc:HGNC:26439]	1.1229268
SLC48A1	solute carrier family 48 member 1 [Source:HGNC Symbol;Acc:HGNC:26035]	-1.4926559
SLC4A2	solute carrier family 4 member 2 [Source:HGNC Symbol;Acc:HGNC:11028]	1.2275552
SLC4A8	solute carrier family 4 member 8 [Source:HGNC Symbol;Acc:HGNC:11034]	-1.1032156
SLC50A1	solute carrier family 50 member 1 [Source:HGNC Symbol;Acc:HGNC:30657]	-1.6497064
SLC5A1	solute carrier family 5 member 1 [Source:HGNC Symbol;Acc:HGNC:11036]	-1.0931611
SLC5A2	solute carrier family 5 member 2 [Source:HGNC Symbol;Acc:HGNC:11037]	-1.0688126
SLC6A2	solute carrier family 6 member 2 [Source:HGNC Symbol;Acc:HGNC:11048]	-1.1199039
SLC6A20	solute carrier family 6 member 20 [Source:HGNC Symbol;Acc:HGNC:30927]	1.0628656
SLC6A4	solute carrier family 6 member 4 [Source:HGNC Symbol;Acc:HGNC:11050]	-1.3332648
SLC6A7	solute carrier family 6 member 7 [Source:HGNC Symbol;Acc:HGNC:11054]	-1.055598
SLC7A14	solute carrier family 7 member 14 [Source:HGNC Symbol;Acc:HGNC:29326]	-1.400286
SLC7A2	solute carrier family 7 member 2 [Source:HGNC Symbol;Acc:HGNC:11060]	-9.099952
SLC7A8	solute carrier family 7 member 8 [Source:HGNC Symbol;Acc:HGNC:11066]	1.2073721
SLC8A1	solute carrier family 8 member A1 [Source:HGNC Symbol;Acc:HGNC:11068]	-1.0831733
SLC8A3	solute carrier family 8 member A3 [Source:HGNC Symbol;Acc:HGNC:11070]	-1.1240797
SLC9A1	solute carrier family 9 member A1 [Source:HGNC Symbol;Acc:HGNC:11071]	-1.2156603
SLC9A7P1	solute carrier family 9 member 7 pseudogene 1 [Source:HGNC Symbol;Acc:HGNC:32679]	-1.087863
SLC9A8	solute carrier family 9 member A8 [Source:HGNC Symbol;Acc:HGNC:20728]	-1.3041918
SLCO1C1	solute carrier organic anion transporter family member 1C1 [Source:HGNC Symbol;Acc:HGNC:13819]	1.0606058
SLCO3A1	solute carrier organic anion transporter family member 3A1 [Source:HGNC Symbol;Acc:HGNC:10952]	-1.1044437
SLCO4A1	solute carrier organic anion transporter family member 4A1 [Source:HGNC Symbol;Acc:HGNC:10953]	-1.4484872
SLIT2	slit guidance ligand 2 [Source:HGNC Symbol;Acc:HGNC:11086]	-1.1446851
SMAD1	SMAD family member 1 [Source:HGNC Symbol;Acc:HGNC:6767]	-1.2996587
SMAD3	SMAD family member 3 [Source:HGNC Symbol;Acc:HGNC:6769]	-2.8720343
SMAD6	SMAD family member 6 [Source:HGNC Symbol;Acc:HGNC:6772]	2.070265
SMAD7	SMAD family member 7 [Source:HGNC Symbol;Acc:HGNC:6773]	1.8674072
SMAD9	SMAD family member 9 [Source:HGNC Symbol;Acc:HGNC:6774]	1.3086945
SMAGP	small cell adhesion glycoprotein [Source:HGNC Symbol;Acc:HGNC:26918]	1.1212053
SMAP1	small ArfGAP 1 [Source:HGNC Symbol;Acc:HGNC:19651]	-1.1217533
SMARCA4	ıatrix associated, actin dependent regulator of chromatin, subfamily a, member 4 [Source:HGNC Symbc	-1.0932351
SMARCB1	ıatrix associated, actin dependent regulator of chromatin, subfamily b, member 1 [Source:HGNC Symbc	1.2377583
SMARCE1	ıatrix associated, actin dependent regulator of chromatin, subfamily e, member 1 [Source:HGNC Symbc	1.2623575
SMEK1	protein phosphatase 4 regulatory subunit 3A	1.3475604
SMEK3P	protein phosphatase 4 regulatory subunit 3C, pseudogene	1.1270995
SMG5	SMG5, nonsense mediated mRNA decay factor [Source:HGNC Symbol;Acc:HGNC:24644]	-1.1425986

SMG6	SMG6, nonsense mediated mRNA decay factor [Source:HGNC Symbol;Acc:HGNC:17809]	1.1846209
SMG9	SMG9, nonsense mediated mRNA decay factor [Source:HGNC Symbol;Acc:HGNC:25763]	-1.186066
SMO	smoothened, frizzled class receptor [Source:HGNC Symbol;Acc:HGNC:11119]	1.2351292
SMOC1	SPARC related modular calcium binding 1 [Source:HGNC Symbol;Acc:HGNC:20318]	-1.1692297
SMOC2	SPARC related modular calcium binding 2 [Source:HGNC Symbol;Acc:HGNC:20323]	1.079961
SMOX	spermine oxidase [Source:HGNC Symbol;Acc:HGNC:15862]	-1.4794824
SMURF1	SMAD specific E3 ubiquitin protein ligase 1 [Source:HGNC Symbol;Acc:HGNC:16807]	-1.9796493
SNAI1	snail family transcriptional repressor 1 [Source:HGNC Symbol;Acc:HGNC:11128]	1.7465347
SNAI2	snail family transcriptional repressor 2 [Source:HGNC Symbol;Acc:HGNC:11094]	1.1152751
SNAI3	snail family transcriptional repressor 3 [Source:HGNC Symbol;Acc:HGNC:18411]	1.1613162
SNAP25	synaptosome associated protein 25 [Source:HGNC Symbol;Acc:HGNC:11132]	-1.077266
SNAP29	synaptosome associated protein 29 [Source:HGNC Symbol;Acc:HGNC:11133]	1.1797107
SNAPC1	small nuclear RNA activating complex polypeptide 1 [Source:HGNC Symbol;Acc:HGNC:11134]	1.2312425
SNAPC4	small nuclear RNA activating complex polypeptide 4 [Source:HGNC Symbol;Acc:HGNC:11137]	-1.755772
SNAR-E	small ILF3/NF90-associated RNA E	-1.0705185
SND1	staphylococcal nuclease and tudor domain containing 1 [Source:HGNC Symbol;Acc:HGNC:30646]	-1.0733922
SNHG12	small nucleolar RNA host gene 12 [Source:HGNC Symbol;Acc:HGNC:30062]	1.2176762
SNHG4	small nucleolar RNA host gene 4 [Source:HGNC Symbol;Acc:HGNC:32964]	1.1724967
SNHG8	small nucleolar RNA host gene 8 [Source:HGNC Symbol;Acc:HGNC:33098]	1.173885
SNN	stannin [Source:HGNC Symbol;Acc:HGNC:11149]	-2.515417
SNORA22	small nucleolar RNA, H/ACA box 22 [Source:HGNC Symbol;Acc:HGNC:32612]	-1.1060855
SNORA58	small nucleolar RNA, H/ACA box 58 [Source:HGNC Symbol;Acc:HGNC:32652]	1.0604714
SNORA78	small nucleolar RNA, H/ACA box 78 [Source:HGNC Symbol;Acc:HGNC:32664]	-1.2393686
SNORD12B	small nucleolar RNA, C/D box 12B [Source:HGNC Symbol;Acc:HGNC:33573]	-1.1459838
SNPH	syntaphilin [Source:HGNC Symbol;Acc:HGNC:15931]	1.2998594
SNRK	SNF related kinase [Source:HGNC Symbol;Acc:HGNC:30598]	1.920905
SNRNP27	small nuclear ribonucleoprotein U4/U6.U5 subunit 27 [Source:HGNC Symbol;Acc:HGNC:30240]	-1.1821932
SNRNP40	small nuclear ribonucleoprotein U5 subunit 40 [Source:HGNC Symbol;Acc:HGNC:30857]	1.1840714
SNRPA	small nuclear ribonucleoprotein polypeptide A [Source:HGNC Symbol;Acc:HGNC:11151]	1.1525942
SNRPA1	small nuclear ribonucleoprotein polypeptide A' [Source:HGNC Symbol;Acc:HGNC:11152]	-1.2197748
SNRPB	small nuclear ribonucleoprotein polypeptides B and B1 [Source:HGNC Symbol;Acc:HGNC:11153]	1.2685963
SNRPC	small nuclear ribonucleoprotein polypeptide C [Source:HGNC Symbol;Acc:HGNC:11157]	1.0592827
SNRPD1	small nuclear ribonucleoprotein D1 polypeptide [Source:HGNC Symbol;Acc:HGNC:11158]	1.1993759
SNRPD3	small nuclear ribonucleoprotein D3 polypeptide [Source:HGNC Symbol;Acc:HGNC:11160]	1.2016234
SNTB2	syntrophin beta 2 [Source:HGNC Symbol;Acc:HGNC:11169]	-1.4100816
SNUPN	snurportin 1 [Source:HGNC Symbol;Acc:HGNC:14245]	-1.2416916
SNX2	sorting nexin 2 [Source:HGNC Symbol;Acc:HGNC:11173]	1.4450452
SNX24	sorting nexin 24 [Source:HGNC Symbol;Acc:HGNC:21533]	1.0954369
SNX30	sorting nexin family member 30 [Source:HGNC Symbol;Acc:HGNC:23685]	1.1995053
SNX6	sorting nexin 6 [Source:HGNC Symbol;Acc:HGNC:14970]	1.2200302
SNX9	sorting nexin 9 [Source:HGNC Symbol;Acc:HGNC:14973]	-1.3864666
SOBP	sine oculis binding protein homolog [Source:HGNC Symbol;Acc:HGNC:29256]	1.4289916
SOCS1	suppressor of cytokine signaling 1 [Source:HGNC Symbol;Acc:HGNC:19383]	-1.4221374
SOCS2	suppressor of cytokine signaling 2 [Source:HGNC Symbol;Acc:HGNC:19382]	-1.1783967
SOCS3	suppressor of cytokine signaling 3 [Source:HGNC Symbol;Acc:HGNC:19391]	1.4182186
SOCS6	suppressor of cytokine signaling 6 [Source:HGNC Symbol;Acc:HGNC:16833]	1.1634434
SOD2	superoxide dismutase 2, mitochondrial [Source:HGNC Symbol;Acc:HGNC:11180]	-2.7629724
SOHLH2	spermatogenesis and oogenesis specific basic helix-loop-helix 2 [Source:HGNC Symbol;Acc:HGNC:26026	1.0950915
SON	SON DNA binding protein [Source:HGNC Symbol;Acc:HGNC:11183]	-1.2067156
SORBS2	sorbin and SH3 domain containing 2 [Source:HGNC Symbol;Acc:HGNC:24098]	1.3960792
SORCS3	sortilin related VPS10 domain containing receptor 3 [Source:HGNC Symbol;Acc:HGNC:16699]	1.1625923
SORD	sorbitol dehydrogenase [Source:HGNC Symbol;Acc:HGNC:11184]	1.1756208
SOX1	SRY-box 1 [Source:HGNC Symbol;Acc:HGNC:11189]	1.1297421
SOX12	SRY-box 12 [Source:HGNC Symbol;Acc:HGNC:11198]	1.1355515
SOX13	SRY-box 13 [Source:HGNC Symbol;Acc:HGNC:11192]	-1.2355682
SOX15	SRY-box 15 [Source:HGNC Symbol;Acc:HGNC:11196]	1.2521343
SOX17	SRY-box 17 [Source:HGNC Symbol;Acc:HGNC:18122]	1.2470721
SOX18	SRY-box 18 [Source:HGNC Symbol;Acc:HGNC:11194]	1.5754496
SOX2OT	SOX2 overlapping transcript	1.1706005
SOX30	SRY-box 30 [Source:HGNC Symbol;Acc:HGNC:30635]	1.1593004
SOX7	SRY-box 7 [Source:HGNC Symbol;Acc:HGNC:18196]	-2.4292214
SP1	Sp1 transcription factor [Source:HGNC Symbol;Acc:HGNC:11205]	-1.1988295
SP110	SP110 nuclear body protein [Source:HGNC Symbol;Acc:HGNC:5401]	-1.1568704
SP140	SP140 nuclear body protein [Source:HGNC Symbol;Acc:HGNC:17133]	-1.13882395
SP2	Sp2 transcription factor [Source:HGNC Symbol;Acc:HGNC:11207]	1.1116369
SP6	Sp6 transcription factor [Source:HGNC Symbol;Acc:HGNC:14530]	-1.7814846
SP8	Sp8 transcription factor [Source:HGNC Symbol;Acc:HGNC:19196]	1.1489772
SPAG17	sperm associated antigen 17 [Source:HGNC Symbol;Acc:HGNC:26620]	1.1051317
SPANXN1	SPANX family member N1 [Source:HGNC Symbol;Acc:HGNC:33174]	1.1521657
SPATA1	spermatogenesis associated 1 [Source:HGNC Symbol;Acc:HGNC:14682]	-1.0945317
SPATA2	spermatogenesis associated 2 [Source:HGNC Symbol;Acc:HGNC:14681]	-1.3791881
SPATA2L	spermatogenesis associated 2 like [Source:HGNC Symbol;Acc:HGNC:28393]	-1.3247792
SPATA5L1	spermatogenesis associated 5 like 1 [Source:HGNC Symbol;Acc:HGNC:28762]	-1.118301
SPATA6	spermatogenesis associated 6 [Source:HGNC Symbol;Acc:HGNC:18309]	1.1510923
SPATA7	spermatogenesis associated 7 [Source:HGNC Symbol;Acc:HGNC:20423]	1.3338883
SPATA9	spermatogenesis associated 9 [Source:HGNC Symbol;Acc:HGNC:22988]	-1.0932338
SPATC1	spermatogenesis and centriole associated 1 [Source:HGNC Symbol;Acc:HGNC:30510]	-1.1541201
SPC25	SPC25, NDC80 kinetochore complex component [Source:HGNC Symbol;Acc:HGNC:24031]	1.1633643
SPECC1	sperm antigen with calponin homology and coiled-coil domains 1 [Source:HGNC Symbol;Acc:HGNC:3061	-1.2656026
SPEG	SPEG complex locus [Source:HGNC Symbol;Acc:HGNC:16901]	-1.1496651
SPG21	spastic paraplegia 21 (autosomal recessive, Mast syndrome) [Source:HGNC Symbol;Acc:HGNC:20373]	-1.0597708
SPG7	SPG7, paraplegin matrix AAA peptidase subunit [Source:HGNC Symbol;Acc:HGNC:11237]	-1.1414359
SPHK1	sphingosine kinase 1 [Source:HGNC Symbol;Acc:HGNC:11240]	-1.5180042
SPI1	Spi-1 proto-oncogene [Source:HGNC Symbol;Acc:HGNC:11241]	-1.3360361
SPIN3	spindlin family member 3 [Source:HGNC Symbol;Acc:HGNC:27272]	1.0840518
SPIN4	spindlin family member 4 [Source:HGNC Symbol;Acc:HGNC:27040]	1.4123063

SPINK1	serine peptidase inhibitor, Kazal type 1 [Source:HGNC Symbol;Acc:HGNC:11244]	-1.1005225
SPINK14	serine peptidase inhibitor, Kazal type 14 (putative) [Source:HGNC Symbol;Acc:HGNC:33825]	-1.0626346
SPINK2	serine peptidase inhibitor, Kazal type 2 [Source:HGNC Symbol;Acc:HGNC:11245]	1.1215383
SPINK8	serine peptidase inhibitor, Kazal type 8 (putative) [Source:HGNC Symbol;Acc:HGNC:33160]	-1.1167119
SPINT4	serine peptidase inhibitor, Kunitz type 4 [Source:HGNC Symbol;Acc:HGNC:16130]	1.103353
SPNS2	sphingolipid transporter 2 [Source:HGNC Symbol;Acc:HGNC:26992]	-1.7125467
SPNS3	sphingolipid transporter 3 (putative) [Source:HGNC Symbol;Acc:HGNC:28433]	-1.3699741
SPOPL	speckle type BTB/POZ protein like [Source:HGNC Symbol;Acc:HGNC:27934]	1.3731387
SPPL2B	signal peptide peptidase like 2B [Source:HGNC Symbol;Acc:HGNC:30627]	1.098127
SPPL3	signal peptide peptidase like 3 [Source:HGNC Symbol;Acc:HGNC:30424]	-1.0523384
SPRED1	sprouty related EVH1 domain containing 1 [Source:HGNC Symbol;Acc:HGNC:20249]	1.3902912
SPRED2	sprouty related EVH1 domain containing 2 [Source:HGNC Symbol;Acc:HGNC:17722]	-1.1629206
SPRR2C	small proline rich protein 2C (pseudogene) [Source:HGNC Symbol;Acc:HGNC:11263]	-1.1278527
SPRR2D	small proline rich protein 2D [Source:HGNC Symbol;Acc:HGNC:11264]	-1.0577679
SPRR2E	small proline rich protein 2E [Source:HGNC Symbol;Acc:HGNC:11265]	-1.1943921
SPRY1	sprouty RTK signaling antagonist 1 [Source:HGNC Symbol;Acc:HGNC:11269]	1.3469466
SPRY2	sprouty RTK signaling antagonist 2 [Source:HGNC Symbol;Acc:HGNC:11270]	1.1147985
SPRY3	sprouty RTK signaling antagonist 3 [Source:HGNC Symbol;Acc:HGNC:11271]	-1.1297424
SPRY4	sprouty RTK signaling antagonist 4 [Source:HGNC Symbol;Acc:HGNC:15533]	-1.295955
SPRYD4	SPRY domain containing 4 [Source:HGNC Symbol;Acc:HGNC:27468]	-1.1377538
SPTA1	spectrin alpha, erythrocytic 1 [Source:HGNC Symbol;Acc:HGNC:11272]	1.1368048
SPTB	spectrin beta, erythrocytic [Source:HGNC Symbol;Acc:HGNC:11274]	1.0735816
SPTBN1	spectrin beta, non-erythrocytic 1 [Source:HGNC Symbol;Acc:HGNC:11275]	-1.2001902
SPTBN5	spectrin beta, non-erythrocytic 5 [Source:HGNC Symbol;Acc:HGNC:15680]	-1.2231363
SQRDL	sulfide quinone reductase-like (yeast) [Source:HGNC Symbol;Acc:HGNC:20390]	-1.9914875
SQSTM1	sequestosome 1 [Source:HGNC Symbol;Acc:HGNC:11280]	-2.8412058
SRCAP	Snf2-related CREBBP activator protein [Source:HGNC Symbol;Acc:HGNC:16974]	1.123425
SREBF2	sterol regulatory element binding transcription factor 2 [Source:HGNC Symbol;Acc:HGNC:11290]	1.1755402
SRG7	spermatogenesis associated 42 (non-protein coding)	1.0715241
SRGAP1	SLIT-ROBO Rho GTPase activating protein 1 [Source:HGNC Symbol;Acc:HGNC:17382]	-1.6967522
SRGAP2	SLIT-ROBO Rho GTPase activating protein 2 [Source:HGNC Symbol;Acc:HGNC:19751]	1.11022265
SRP19	signal recognition particle 19 [Source:HGNC Symbol;Acc:HGNC:11300]	1.1605332
SRP9	signal recognition particle 9 [Source:HGNC Symbol;Acc:HGNC:11304]	1.4022194
SRPK2	SRSF protein kinase 2 [Source:HGNC Symbol;Acc:HGNC:11306]	1.2924603
SRPRB	SRP receptor beta subunit [Source:HGNC Symbol;Acc:HGNC:24085]	1.0793916
SRPX2	sushi repeat containing protein, X-linked 2 [Source:HGNC Symbol;Acc:HGNC:30668]	1.1076976
SRRM2	serine/arginine repetitive matrix 2 [Source:HGNC Symbol;Acc:HGNC:16639]	-1.2876629
SRRM3	serine/arginine repetitive matrix 3 [Source:HGNC Symbol;Acc:HGNC:26729]	1.3769407
SRRT	serrate, RNA effector molecule [Source:HGNC Symbol;Acc:HGNC:24101]	-1.1109728
SRSF12	serine and arginine rich splicing factor 12 [Source:HGNC Symbol;Acc:HGNC:21220]	-1.0577995
SRSF8	serine and arginine rich splicing factor 8 [Source:HGNC Symbol;Acc:HGNC:16988]	1.2972506
SRXN1	sulfiredoxin 1 [Source:HGNC Symbol;Acc:HGNC:16132]	1.1963407
SSBP3	single stranded DNA binding protein 3 [Source:HGNC Symbol;Acc:HGNC:15674]	-1.1203994
SSBP4	single stranded DNA binding protein 4 [Source:HGNC Symbol;Acc:HGNC:15676]	1.3567343
SSFA2	sperm specific antigen 2 [Source:HGNC Symbol;Acc:HGNC:11319]	2.0738115
SSH1	slingshot protein phosphatase 1 [Source:HGNC Symbol;Acc:HGNC:30579]	-2.0153873
SSH2	slingshot protein phosphatase 2 [Source:HGNC Symbol;Acc:HGNC:30580]	-1.3045303
SSH3	slingshot protein phosphatase 3 [Source:HGNC Symbol;Acc:HGNC:30581]	1.06624
SSTR1	somatostatin receptor 1 [Source:HGNC Symbol;Acc:HGNC:11330]	1.4423593
SSTR2	somatostatin receptor 2 [Source:HGNC Symbol;Acc:HGNC:11331]	-1.245914
SSU72	SSU72 homolog, RNA polymerase II CTD phosphatase [Source:HGNC Symbol;Acc:HGNC:25016]	-1.1449313
SSX2	SSX family member 2 [Source:HGNC Symbol;Acc:HGNC:11336]	1.1037519
SSX7	SSX family member 7 [Source:HGNC Symbol;Acc:HGNC:19653]	1.0840541
ST3GAL1	ST3 beta-galactoside alpha-2,3-sialyltransferase 1 [Source:HGNC Symbol;Acc:HGNC:10862]	1.1872938
ST3GAL2	ST3 beta-galactoside alpha-2,3-sialyltransferase 2 [Source:HGNC Symbol;Acc:HGNC:10863]	-1.1934189
ST3GAL6	ST3 beta-galactoside alpha-2,3-sialyltransferase 6 [Source:HGNC Symbol;Acc:HGNC:18080]	1.3964665
ST6GAL1	ST6 beta-galactoside alpha-2,6-sialyltransferase 1 [Source:HGNC Symbol;Acc:HGNC:10860]	-1.754508
ST6GALNAC3	ST6 N-acetylgalactosaminide alpha-2,6-sialyltransferase 3 [Source:HGNC Symbol;Acc:HGNC:19343]	1.3754711
ST7	suppression of tumorigenicity 7 [Source:HGNC Symbol;Acc:HGNC:11351]	-1.1680267
ST7-AS1	ST7 antisense RNA 1 [Source:HGNC Symbol;Acc:HGNC:16000]	-1.504437
ST7OT4	ST7 overlapping transcript 4	1.0794348
ST8SIA4	ST8 alpha-N-acetyl-neuraminide alpha-2,8-sialyltransferase 4 [Source:HGNC Symbol;Acc:HGNC:10871]	1.1466286
STAC2	SH3 and cysteine rich domain 2 [Source:HGNC Symbol;Acc:HGNC:23990]	-1.1196139
STAP2	signal transducing adaptor family member 2 [Source:HGNC Symbol;Acc:HGNC:30430]	-2.1225429
STARD10	StAR related lipid transfer domain containing 10 [Source:HGNC Symbol;Acc:HGNC:10666]	-2.8078258
STARD8	StAR related lipid transfer domain containing 8 [Source:HGNC Symbol;Acc:HGNC:19161]	-1.4184645
STAT2	signal transducer and activator of transcription 2 [Source:HGNC Symbol;Acc:HGNC:11363]	1.1347741
STAT5A	signal transducer and activator of transcription 5A [Source:HGNC Symbol;Acc:HGNC:11366]	-3.1095648
STAT5B	signal transducer and activator of transcription 5B [Source:HGNC Symbol;Acc:HGNC:11367]	-1.1172311
STAT6	signal transducer and activator of transcription 6 [Source:HGNC Symbol;Acc:HGNC:11368]	-2.2853472
STBD1	starch binding domain 1 [Source:HGNC Symbol;Acc:HGNC:24854]	1.1040965
STK10	serine/threonine kinase 10 [Source:HGNC Symbol;Acc:HGNC:11388]	-1.5018309
STK4	serine/threonine kinase 4 [Source:HGNC Symbol;Acc:HGNC:11408]	-1.0922916
STK40	serine/threonine kinase 40 [Source:HGNC Symbol;Acc:HGNC:21373]	-1.2458426
STOML2	stomatin like 2 [Source:HGNC Symbol;Acc:HGNC:14559]	-1.0513841
STOML3	stomatin like 3 [Source:HGNC Symbol;Acc:HGNC:19420]	1.1237805
STRADA	STE20-related kinase adaptor alpha [Source:HGNC Symbol;Acc:HGNC:30172]	1.1524712
STRC	stereocilin [Source:HGNC Symbol;Acc:HGNC:16035]	-1.2619677
STRN3	striatin 3 [Source:HGNC Symbol;Acc:HGNC:15720]	1.22326
STX11	syntaxin 11 [Source:HGNC Symbol;Acc:HGNC:11429]	-1.7400285
STX16	syntaxin 16 [Source:HGNC Symbol;Acc:HGNC:11431]	1.1989602
STX18	syntaxin 18 [Source:HGNC Symbol;Acc:HGNC:15942]	-1.0655811
STX1A	syntaxin 1A [Source:HGNC Symbol;Acc:HGNC:11433]	1.4042501
STX3	syntaxin 3 [Source:HGNC Symbol;Acc:HGNC:11438]	1.4778314
STX4	syntaxin 4 [Source:HGNC Symbol;Acc:HGNC:11439]	-1.216301
STX6	syntaxin 6 [Source:HGNC Symbol;Acc:HGNC:11441]	-1.3965899

STXBP5	syntaxin binding protein 5 [Source:HGNC Symbol;Acc:HGNC:19665]	1.1357594
STXBP6	syntaxin binding protein 6 [Source:HGNC Symbol;Acc:HGNC:19666]	1.1285287
STYK1	serine/threonine/tyrosine kinase 1 [Source:HGNC Symbol;Acc:HGNC:18889]	1.5221668
SUCNR1	succinate receptor 1 [Source:HGNC Symbol;Acc:HGNC:4542]	1.1384605
SUFU	SUFU negative regulator of hedgehog signaling [Source:HGNC Symbol;Acc:HGNC:16466]	-1.0691607
SULT1A4	sulfotransferase family 1A member 4 [Source:HGNC Symbol;Acc:HGNC:30004]	-1.30498895
SULT1B1	sulfotransferase family 1B member 1 [Source:HGNC Symbol;Acc:HGNC:17845]	1.222456
SULT1C3	sulfotransferase family 1C member 3 [Source:HGNC Symbol;Acc:HGNC:33543]	1.0664893
SULT1E1	sulfotransferase family 1E member 1 [Source:HGNC Symbol;Acc:HGNC:11377]	1.1907842
SULT2A1	sulfotransferase family 2A member 1 [Source:HGNC Symbol;Acc:HGNC:11458]	-1.1195066
SUMO1	small ubiquitin-like modifier 1 [Source:HGNC Symbol;Acc:HGNC:12502]	1.1942035
SUMO1P3	SUMO1 pseudogene 3 [Source:HGNC Symbol;Acc:HGNC:33150]	1.2398506
SUMO3	small ubiquitin-like modifier 3 [Source:HGNC Symbol;Acc:HGNC:11124]	-1.1117625
SUN1	Sad1 and UNC84 domain containing 1 [Source:HGNC Symbol;Acc:HGNC:18587]	-1.1049213
SUPT3H	SPT3 homolog, SAGA and STAGA complex component [Source:HGNC Symbol;Acc:HGNC:11466]	-1.092337
SUSD5	sushi domain containing 5 [Source:HGNC Symbol;Acc:HGNC:29061]	-1.245893
SUV39H1	suppressor of variegation 3-9 homolog 1 [Source:HGNC Symbol;Acc:HGNC:11479]	1.372106
SUV39H2	suppressor of variegation 3-9 homolog 2 [Source:HGNC Symbol;Acc:HGNC:17287]	1.412716
SUV420H1	lysine methyltransferase 5B	1.1434336
SUV420H2	lysine methyltransferase 5C	1.3840066
SVIL	supervillin [Source:HGNC Symbol;Acc:HGNC:11480]	-1.3011549
SVIP	small VCP interacting protein [Source:HGNC Symbol;Acc:HGNC:25238]	1.1497164
SVOP	SV2 related protein [Source:HGNC Symbol;Acc:HGNC:25417]	1.07458
SVOPL	SVOP like [Source:HGNC Symbol;Acc:HGNC:27034]	1.1703988
SYAP1	synapse associated protein 1 [Source:HGNC Symbol;Acc:HGNC:16273]	1.1688595
SYBU	syntabulin [Source:HGNC Symbol;Acc:HGNC:26011]	1.1641141
SYN2	synapsin II [Source:HGNC Symbol;Acc:HGNC:11495]	-1.1460617
SYNDIG1	synapse differentiation inducing 1 [Source:HGNC Symbol;Acc:HGNC:15885]	1.1307203
SYNE1	spectrin repeat containing nuclear envelope protein 1 [Source:HGNC Symbol;Acc:HGNC:17089]	1.2101638
SYNGR3	synaptogyrin 3 [Source:HGNC Symbol;Acc:HGNC:11501]	-1.33068305
SYNJ2	synaptojanin 2 [Source:HGNC Symbol;Acc:HGNC:11504]	-1.2392128
SYNPO	synaptopodin [Source:HGNC Symbol;Acc:HGNC:30672]	-1.3194586
SYNRG	synergin, gamma [Source:HGNC Symbol;Acc:HGNC:557]	-1.1342684
SYS1	Sys1 golgi trafficking protein [Source:HGNC Symbol;Acc:HGNC:16162]	-1.17128965
SYT11	synaptotagmin 11 [Source:HGNC Symbol;Acc:HGNC:19239]	-1.2013193
SYT12	synaptotagmin 12 [Source:HGNC Symbol;Acc:HGNC:18381]	1.0516901
SYT14	synaptotagmin 14 [Source:HGNC Symbol;Acc:HGNC:23143]	-1.1756594
SYT2	synaptotagmin 2 [Source:HGNC Symbol;Acc:HGNC:11510]	-1.11471305
SYT6	synaptotagmin 6 [Source:HGNC Symbol;Acc:HGNC:18638]	-1.0813587
SYTL2	synaptotagmin like 2 [Source:HGNC Symbol;Acc:HGNC:15585]	1.0857561
SYVN1	synoviolin 1 [Source:HGNC Symbol;Acc:HGNC:20738]	1.1483997
SZT2	seizure threshold 2 homolog (mouse) [Source:HGNC Symbol;Acc:HGNC:29040]	-1.1587
TAAR1	trace amine associated receptor 1 [Source:HGNC Symbol;Acc:HGNC:17734]	1.1227604
TAAR8	trace amine associated receptor 8 [Source:HGNC Symbol;Acc:HGNC:14964]	-1.1320313
TAB2	TGF-beta activated kinase 1/MAP3K7 binding protein 2 [Source:HGNC Symbol;Acc:HGNC:17075]	-1.38926615
TAC1	tachykinin precursor 1 [Source:HGNC Symbol;Acc:HGNC:11517]	1.1266389
TACR1	tachykinin receptor 1 [Source:HGNC Symbol;Acc:HGNC:11526]	-1.0792997
TACSTD2	tumor-associated calcium signal transducer 2 [Source:HGNC Symbol;Acc:HGNC:11530]	1.2108705
TAF12	TATA-box binding protein associated factor 12 [Source:HGNC Symbol;Acc:HGNC:11545]	1.1606117
TAF1A	-box binding protein associated factor, RNA polymerase I subunit A [Source:HGNC Symbol;Acc:HGNC:1	1.0814369
TAF1B	-box binding protein associated factor, RNA polymerase I subunit B [Source:HGNC Symbol;Acc:HGNC:1	1.2733359
TAF1C	-box binding protein associated factor, RNA polymerase I subunit C [Source:HGNC Symbol;Acc:HGNC:1	-1.2037797
TAF5	TATA-box binding protein associated factor 5 [Source:HGNC Symbol;Acc:HGNC:11539]	1.2037756
TAF9B	TATA-box binding protein associated factor 9b [Source:HGNC Symbol;Acc:HGNC:17306]	1.2268778
TAL2	TAL bHLH transcription factor 2 [Source:HGNC Symbol;Acc:HGNC:11557]	-1.1466997
TANK	TRAF family member associated NFKB activator [Source:HGNC Symbol;Acc:HGNC:11562]	-1.2746692
TAP1	transporter 1, ATP binding cassette subfamily B member [Source:HGNC Symbol;Acc:HGNC:43]	-5.840709
TAP2	transporter 2, ATP binding cassette subfamily B member [Source:HGNC Symbol;Acc:HGNC:44]	-1.3878918
TAPBP	TAP binding protein (tapasin) [Source:HGNC Symbol;Acc:HGNC:11566]	-1.659206425
TAPBPL	TAP binding protein like [Source:HGNC Symbol;Acc:HGNC:30683]	-1.2435796
TAPT1	transmembrane anterior posterior transformation 1 [Source:HGNC Symbol;Acc:HGNC:26887]	1.2648808
TARBP1	TAR (HIV-1) RNA binding protein 1 [Source:HGNC Symbol;Acc:HGNC:11568]	1.1722906
TAS2R1	taste 2 receptor member 1 [Source:HGNC Symbol;Acc:HGNC:14909]	1.1054171
TAS2R38	taste 2 receptor member 38 [Source:HGNC Symbol;Acc:HGNC:9584]	1.0671
TAS2R42	taste 2 receptor member 42 [Source:HGNC Symbol;Acc:HGNC:18888]	1.1000696
TAS2R8	taste 2 receptor member 8 [Source:HGNC Symbol;Acc:HGNC:14915]	-1.1002846
TAT	tyrosine aminotransferase [Source:HGNC Symbol;Acc:HGNC:11573]	1.4149128
TATDN2	TatD DNase domain containing 2 [Source:HGNC Symbol;Acc:HGNC:28988]	-1.2221595
TBC1D1	TBC1 domain family member 1 [Source:HGNC Symbol;Acc:HGNC:11578]	-1.16956065
TBC1D10A	TBC1 domain family member 10A [Source:HGNC Symbol;Acc:HGNC:23609]	-1.6626483
TBC1D10B	TBC1 domain family member 10B [Source:HGNC Symbol;Acc:HGNC:24510]	1.4120173
TBC1D10C	TBC1 domain family member 10C [Source:HGNC Symbol;Acc:HGNC:24702]	1.0942843
TBC1D20	TBC1 domain family member 20 [Source:HGNC Symbol;Acc:HGNC:16133]	-1.1258305
TBC1D22A	TBC1 domain family member 22A [Source:HGNC Symbol;Acc:HGNC:1309]	-1.2354038
TBC1D22B	TBC1 domain family member 22B [Source:HGNC Symbol;Acc:HGNC:21602]	-1.6846085
TBC1D24	TBC1 domain family member 24 [Source:HGNC Symbol;Acc:HGNC:29203]	-1.1942153
TBC1D25	TBC1 domain family member 25 [Source:HGNC Symbol;Acc:HGNC:8092]	-1.1994458
TBC1D27	TBC1 domain family member 27 [Source:HGNC Symbol;Acc:HGNC:28104]	1.0916164
TBC1D8	TBC1 domain family member 8 [Source:HGNC Symbol;Acc:HGNC:17791]	1.7108886
TBCD	tubulin folding cofactor D [Source:HGNC Symbol;Acc:HGNC:11581]	-1.2003107
TBCEL	tubulin folding cofactor E like [Source:HGNC Symbol;Acc:HGNC:28115]	1.2188548
TBL1X	transducin beta like 1X-linked [Source:HGNC Symbol;Acc:HGNC:11585]	-1.205578
TBL1Y	transducin beta like 1, Y-linked [Source:HGNC Symbol;Acc:HGNC:18502]	-1.3603249
TBL2	transducin beta like 2 [Source:HGNC Symbol;Acc:HGNC:11586]	1.2404001
TBP	TATA-box binding protein [Source:HGNC Symbol;Acc:HGNC:11588]	1.1188513
TBRG1	transforming growth factor beta regulator 1 [Source:HGNC Symbol;Acc:HGNC:29551]	-1.1646354

TBX1	T-box 1 [Source:HGNC Symbol;Acc:HGNC:11592]	1.72025485
TBX19	T-box 19 [Source:HGNC Symbol;Acc:HGNC:11596]	-1.3837837
TBX20	T-box 20 [Source:HGNC Symbol;Acc:HGNC:11598]	1.1061829
TBX22	T-box 22 [Source:HGNC Symbol;Acc:HGNC:11600]	1.143525
TBXA2R	thromboxane A2 receptor [Source:HGNC Symbol;Acc:HGNC:11608]	-1.3096033
TCEA1	transcription elongation factor A1 [Source:HGNC Symbol;Acc:HGNC:11612]	1.2140621
TCEA2	transcription elongation factor A2 [Source:HGNC Symbol;Acc:HGNC:11614]	1.2129707
TCEAL5	transcription elongation factor A like 5 [Source:HGNC Symbol;Acc:HGNC:22282]	-1.1620266
TCEAL8	transcription elongation factor A like 8 [Source:HGNC Symbol;Acc:HGNC:28683]	1.2279946
TCF15	transcription factor 15 (basic helix-loop-helix) [Source:HGNC Symbol;Acc:HGNC:11627]	-2.0575776
TCF21	transcription factor 21 [Source:HGNC Symbol;Acc:HGNC:11632]	1.0974195
TCF25	transcription factor 25 [Source:HGNC Symbol;Acc:HGNC:29181]	-1.1429046
TCF7	transcription factor 7 (T-cell specific, HMG-box) [Source:HGNC Symbol;Acc:HGNC:11639]	-1.544704
TCF7L1	transcription factor 7 like 1 [Source:HGNC Symbol;Acc:HGNC:11640]	-1.552054
TCF7L2	transcription factor 7 like 2 [Source:HGNC Symbol;Acc:HGNC:11641]	-1.1125649
TCL1A	T-cell leukemia/lymphoma 1A [Source:HGNC Symbol;Acc:HGNC:11648]	-1.0657189
TCL6	T-cell leukemia/lymphoma 6 (non-protein coding) [Source:HGNC Symbol;Acc:HGNC:13463]	1.1174482
TCOF1	treacle ribosome biogenesis factor 1 [Source:HGNC Symbol;Acc:HGNC:11654]	1.1835889
TCP11L1	t-complex 11 like 1 [Source:HGNC Symbol;Acc:HGNC:25655]	-1.3249453
TCTE1	t-complex-associated-testis-expressed 1 [Source:HGNC Symbol;Acc:HGNC:11693]	-1.1561464
TCTEX1D4	Tctex1 domain containing 4 [Source:HGNC Symbol;Acc:HGNC:32315]	-1.1280748
TCTN2	tectonic family member 2 [Source:HGNC Symbol;Acc:HGNC:25774]	-1.1062703
TDG	thymine DNA glycosylase [Source:HGNC Symbol;Acc:HGNC:11700]	1.3547912
TDP2	tyrosyl-DNA phosphodiesterase 2 [Source:HGNC Symbol;Acc:HGNC:17768]	1.3462905
TDRD1	tudor domain containing 1 [Source:HGNC Symbol;Acc:HGNC:11712]	-1.1629562
TDRD5	tudor domain containing 5 [Source:HGNC Symbol;Acc:HGNC:20614]	1.1321472
TDRD7	tudor domain containing 7 [Source:HGNC Symbol;Acc:HGNC:30831]	-1.2014122
TECPR1	tectonin beta-propeller repeat containing 1 [Source:HGNC Symbol;Acc:HGNC:22214]	-1.0677514
TECPR2	tectonin beta-propeller repeat containing 2 [Source:HGNC Symbol;Acc:HGNC:19957]	-1.1434623
TECTA	tectorin alpha [Source:HGNC Symbol;Acc:HGNC:11720]	-1.0953746
TEF	TEF, PAR bZIP transcription factor [Source:HGNC Symbol;Acc:HGNC:11722]	-1.2507441
TEKT3	tektin 3 [Source:HGNC Symbol;Acc:HGNC:14293]	-1.0586303
TENC1	tensin 2	1.7355207
TEPP	testis, prostate and placenta expressed [Source:HGNC Symbol;Acc:HGNC:33745]	-1.294156
TES	testin LIM domain protein [Source:HGNC Symbol;Acc:HGNC:14620]	-1.242406
TESC	tescalcin [Source:HGNC Symbol;Acc:HGNC:26065]	-1.229247
TET3	tet methylcytosine dioxygenase 3 [Source:HGNC Symbol;Acc:HGNC:28313]	1.0833104
TEX12	testis expressed 12 [Source:HGNC Symbol;Acc:HGNC:11734]	1.0969146
TEX15	testis expressed 15 [Source:HGNC Symbol;Acc:HGNC:11738]	1.0760782
TEX9	testis expressed 9 [Source:HGNC Symbol;Acc:HGNC:29585]	1.1261269
TFB1M	transcription factor B1, mitochondrial [Source:HGNC Symbol;Acc:HGNC:17037]	-1.1552731
TFE3	transcription factor binding to IGHM enhancer 3 [Source:HGNC Symbol;Acc:HGNC:11752]	-1.3735228
TFEB	transcription factor EB [Source:HGNC Symbol;Acc:HGNC:11753]	-1.215368
TFF2	trefoil factor 2 [Source:HGNC Symbol;Acc:HGNC:11756]	-1.1593903
TFG	TRK-fused gene [Source:HGNC Symbol;Acc:HGNC:11758]	-1.2120285
TFIP11	tuftelin interacting protein 11 [Source:HGNC Symbol;Acc:HGNC:17165]	-1.1499203
TFPI2	tissue factor pathway inhibitor 2 [Source:HGNC Symbol;Acc:HGNC:11761]	-1.2973212
TFRC	transferrin receptor [Source:HGNC Symbol;Acc:HGNC:11763]	1.3148665
TGFA	transforming growth factor alpha [Source:HGNC Symbol;Acc:HGNC:11765]	-1.2942945
TGFB1	transforming growth factor beta 1 [Source:HGNC Symbol;Acc:HGNC:11766]	1.2400473
TGFBR2	transforming growth factor beta receptor 2 [Source:HGNC Symbol;Acc:HGNC:11773]	-1.6929325
TGIF2LY	TGFB induced factor homeobox 2 like, Y-linked [Source:HGNC Symbol;Acc:HGNC:18569]	-1.0957899
TGS1	trimethylguanosine synthase 1 [Source:HGNC Symbol;Acc:HGNC:17843]	1.644198
THAP1	THAP domain containing 1 [Source:HGNC Symbol;Acc:HGNC:20856]	1.2663431
THAP5	THAP domain containing 5 [Source:HGNC Symbol;Acc:HGNC:23188]	1.1731004
THAP8	THAP domain containing 8 [Source:HGNC Symbol;Acc:HGNC:23191]	1.1259615
THBD	thrombomodulin [Source:HGNC Symbol;Acc:HGNC:11784]	2.5723958
THEM4	thioesterase superfamily member 4 [Source:HGNC Symbol;Acc:HGNC:17947]	-1.0741367
THOC3	THO complex 3 [Source:HGNC Symbol;Acc:HGNC:19072]	1.1531777
THOC7	THO complex 7 [Source:HGNC Symbol;Acc:HGNC:29874]	1.2816858
THSD1	thrombospondin type 1 domain containing 1 [Source:HGNC Symbol;Acc:HGNC:17754]	1.1414342
THUMPD3	THUMP domain containing 3 [Source:HGNC Symbol;Acc:HGNC:24493]	1.2828622
THY1	Thy-1 cell surface antigen [Source:HGNC Symbol;Acc:HGNC:11801]	-1.495947
TICAM1	toll like receptor adaptor molecule 1 [Source:HGNC Symbol;Acc:HGNC:18348]	-1.68680635
TIE1	yrosine kinase with immunoglobulin like and EGF like domains 1 [Source:HGNC Symbol;Acc:HGNC:1180	1.2269287
TIFA	TRAF interacting protein with forkhead associated domain [Source:HGNC Symbol;Acc:HGNC:19075]	-1.3406183
TIGD2	tigger transposable element derived 2 [Source:HGNC Symbol;Acc:HGNC:18333]	1.2720616
TIGD4	tigger transposable element derived 4 [Source:HGNC Symbol;Acc:HGNC:18335]	1.0937688
TIMM17B	nslocase of inner mitochondrial membrane 17 homolog B (yeast) [Source:HGNC Symbol;Acc:HGNC:173	-1.3398985
TIMM23	translocase of inner mitochondrial membrane 23 [Source:HGNC Symbol;Acc:HGNC:17312]	-1.1232618
TIMM8A	anslocase of inner mitochondrial membrane 8 homolog A (yeast) [Source:HGNC Symbol;Acc:HGNC:118	1.1209888
TIMM8B	translocase of inner mitochondrial membrane 8 homolog B [Source:HGNC Symbol;Acc:HGNC:11818]	-1.1581358
TIMM9	translocase of inner mitochondrial membrane 9 [Source:HGNC Symbol;Acc:HGNC:11819]	1.2661512
TIMP3	TIMP metallopeptidase inhibitor 3 [Source:HGNC Symbol;Acc:HGNC:11822]	-1.5256875
TINF2	TERF1 interacting nuclear factor 2 [Source:HGNC Symbol;Acc:HGNC:11824]	-1.1987168
TIPARP	TCDD inducible poly(ADP-ribose) polymerase [Source:HGNC Symbol;Acc:HGNC:23696]	1.3308982
TJAP1	tight junction associated protein 1 [Source:HGNC Symbol;Acc:HGNC:17949]	-1.3311831
TKTL1	transketolase like 1 [Source:HGNC Symbol;Acc:HGNC:11835]	-1.1161263
TKTL2	transketolase like 2 [Source:HGNC Symbol;Acc:HGNC:25313]	1.1063786
TLE3	transducin like enhancer of split 3 [Source:HGNC Symbol;Acc:HGNC:11839]	-1.2865205
TLE4	transducin like enhancer of split 4 [Source:HGNC Symbol;Acc:HGNC:11840]	-1.21103525
TLN1	talin 1 [Source:HGNC Symbol;Acc:HGNC:11845]	1.2138771
TLR2	toll like receptor 2 [Source:HGNC Symbol;Acc:HGNC:11848]	-1.508518
TLR4	toll like receptor 4 [Source:HGNC Symbol;Acc:HGNC:11850]	1.7092305
TM4SF19	transmembrane 4 L six family member 19 [Source:HGNC Symbol;Acc:HGNC:25167]	-1.1430283
TM4SF20	transmembrane 4 L six family member 20 [Source:HGNC Symbol;Acc:HGNC:26230]	1.0508955

TM4SF5	transmembrane 4 L six family member 5 [Source:HGNC Symbol;Acc:HGNC:11857]	-1.131919
TM9SF4	transmembrane 9 superfamily member 4 [Source:HGNC Symbol;Acc:HGNC:30797]	1.1924593
TMC7	transmembrane channel like 7 [Source:HGNC Symbol;Acc:HGNC:23000]	1.2306852
TMCC2	transmembrane and coiled-coil domain family 2 [Source:HGNC Symbol;Acc:HGNC:24239]	-2.2500358
TMCC3	transmembrane and coiled-coil domain family 3 [Source:HGNC Symbol;Acc:HGNC:29199]	1.3422843
TMCO2	transmembrane and coiled-coil domains 2 [Source:HGNC Symbol;Acc:HGNC:23312]	-1.2528173
TMCO5A	transmembrane and coiled-coil domains 5A [Source:HGNC Symbol;Acc:HGNC:28558]	-1.1507288
TMED10	transmembrane p24 trafficking protein 10 [Source:HGNC Symbol;Acc:HGNC:16998]	1.3314298
TMED5	transmembrane p24 trafficking protein 5 [Source:HGNC Symbol;Acc:HGNC:24251]	1.5333451
TMED7-TICAM2	TMED7-TICAM2 readthrough [Source:HGNC Symbol;Acc:HGNC:33945]	-1.3315955
TMEFF2	smembrane protein with EGF like and two follistatin like domains 2 [Source:HGNC Symbol;Acc:HGNC:11	1.0801088
TMEM100	transmembrane protein 100 [Source:HGNC Symbol;Acc:HGNC:25607]	1.1063317
TMEM106A	transmembrane protein 106A [Source:HGNC Symbol;Acc:HGNC:28288]	-1.2923992
TMEM106B	transmembrane protein 106B [Source:HGNC Symbol;Acc:HGNC:22407]	1.2412679
TMEM106C	transmembrane protein 106C [Source:HGNC Symbol;Acc:HGNC:28775]	1.2137396
TMEM11	transmembrane protein 11 [Source:HGNC Symbol;Acc:HGNC:16823]	1.1345352
TMEM111	ER membrane protein complex subunit 3	-1.1016078
TMEM120A	transmembrane protein 120A [Source:HGNC Symbol;Acc:HGNC:21697]	-1.3857841
TMEM121	transmembrane protein 121 [Source:HGNC Symbol;Acc:HGNC:20511]	1.663474
TMEM126B	transmembrane protein 126B [Source:HGNC Symbol;Acc:HGNC:30883]	1.186125
TMEM135	transmembrane protein 135 [Source:HGNC Symbol;Acc:HGNC:26167]	1.2945952
TMEM14A	transmembrane protein 14A [Source:HGNC Symbol;Acc:HGNC:21076]	1.132862
TMEM14B	transmembrane protein 14B [Source:HGNC Symbol;Acc:HGNC:21384]	-1.1426967
TMEM14C	transmembrane protein 14C [Source:HGNC Symbol;Acc:HGNC:20952]	-1.1689383
TMEM156	transmembrane protein 156 [Source:HGNC Symbol;Acc:HGNC:26260]	1.1164584
TMEM158	transmembrane protein 158 (gene/pseudogene) [Source:HGNC Symbol;Acc:HGNC:30293]	-1.2943968
TMEM167A	transmembrane protein 167A [Source:HGNC Symbol;Acc:HGNC:28330]	-1.1429507
TMEM167B	transmembrane protein 167B [Source:HGNC Symbol;Acc:HGNC:30187]	-1.4892629
TMEM169	transmembrane protein 169 [Source:HGNC Symbol;Acc:HGNC:25130]	1.0994143
TMEM171	transmembrane protein 171 [Source:HGNC Symbol;Acc:HGNC:27031]	-1.4058913
TMEM173	transmembrane protein 173 [Source:HGNC Symbol;Acc:HGNC:27962]	-1.2409935
TMEM177	transmembrane protein 177 [Source:HGNC Symbol;Acc:HGNC:28143]	1.3067323
TMEM185A	transmembrane protein 185A [Source:HGNC Symbol;Acc:HGNC:17125]	-1.3029523
TMEM185B	transmembrane protein 185B [Source:HGNC Symbol;Acc:HGNC:18896]	-1.1686187
TMEM189	transmembrane protein 189 [Source:HGNC Symbol;Acc:HGNC:16735]	1.2265846
TMEM191C	transmembrane protein 191C [Source:HGNC Symbol;Acc:HGNC:33601]	-1.099501
TMEM200B	transmembrane protein 200B [Source:HGNC Symbol;Acc:HGNC:33785]	-1.0794677
TMEM207	transmembrane protein 207 [Source:HGNC Symbol;Acc:HGNC:33705]	1.1245984
TMEM215	transmembrane protein 215 [Source:HGNC Symbol;Acc:HGNC:33816]	1.1655358
TMEM217	transmembrane protein 217 [Source:HGNC Symbol;Acc:HGNC:21238]	-4.6164846
TMEM22	solute carrier family 35 member G2	-1.3441243
TMEM223	transmembrane protein 223 [Source:HGNC Symbol;Acc:HGNC:28464]	1.2169384
TMEM225	transmembrane protein 225 [Source:HGNC Symbol;Acc:HGNC:32390]	1.1413114
TMEM229B	transmembrane protein 229B [Source:HGNC Symbol;Acc:HGNC:20130]	-1.1118013
TMEM237	transmembrane protein 237 [Source:HGNC Symbol;Acc:HGNC:14432]	1.1738029
TMEM25	transmembrane protein 25 [Source:HGNC Symbol;Acc:HGNC:25890]	1.2146704
TMEM35	transmembrane protein 35A	1.2645749
TMEM38B	transmembrane protein 38B [Source:HGNC Symbol;Acc:HGNC:25535]	-1.2232078
TMEM39B	transmembrane protein 39B [Source:HGNC Symbol;Acc:HGNC:25510]	1.2717906
TMEM40	transmembrane protein 40 [Source:HGNC Symbol;Acc:HGNC:25620]	-1.3055817
TMEM41A	transmembrane protein 41A [Source:HGNC Symbol;Acc:HGNC:30544]	-1.1097478
TMEM43	transmembrane protein 43 [Source:HGNC Symbol;Acc:HGNC:28472]	-1.1273928
TMEM44	transmembrane protein 44 [Source:HGNC Symbol;Acc:HGNC:25120]	-1.2716272
TMEM50B	transmembrane protein 50B [Source:HGNC Symbol;Acc:HGNC:1280]	1.2522484
TMEM55A	transmembrane protein 55A [Source:HGNC Symbol;Acc:HGNC:25452]	1.4456233
TMEM61	transmembrane protein 61 [Source:HGNC Symbol;Acc:HGNC:27296]	-1.0690391
TMEM64	transmembrane protein 64 [Source:HGNC Symbol;Acc:HGNC:25441]	1.2531525
TMEM65	transmembrane protein 65 [Source:HGNC Symbol;Acc:HGNC:25203]	1.3447341
TMEM67	transmembrane protein 67 [Source:HGNC Symbol;Acc:HGNC:28396]	1.1461643
TMEM68	transmembrane protein 68 [Source:HGNC Symbol;Acc:HGNC:26510]	1.2308097
TMEM69	transmembrane protein 69 [Source:HGNC Symbol;Acc:HGNC:28035]	-1.1867077
TMEM71	transmembrane protein 71 [Source:HGNC Symbol;Acc:HGNC:26572]	-1.3289893
TMEM86A	transmembrane protein 86A [Source:HGNC Symbol;Acc:HGNC:26890]	1.0948877
TMEM8C	transmembrane protein 8C [Source:HGNC Symbol;Acc:HGNC:33778]	-1.2153137
TMEM93	ER membrane protein complex subunit 6	1.1098008
TMIE	transmembrane inner ear [Source:HGNC Symbol;Acc:HGNC:30800]	-1.5493641
TMIGD1	transmembrane and immunoglobulin domain containing 1 [Source:HGNC Symbol;Acc:HGNC:32431]	1.0664574
TMOD1	tropomodulin 1 [Source:HGNC Symbol;Acc:HGNC:11871]	-1.2055215
TMPPE	transmembrane protein with metallophosphoesterase domain [Source:HGNC Symbol;Acc:HGNC:33865	-1.2083989
TMPRSS11E	transmembrane protease, serine 11E [Source:HGNC Symbol;Acc:HGNC:24465]	1.0765096
TMPRSS11F	transmembrane protease, serine 11F [Source:HGNC Symbol;Acc:HGNC:29994]	1.0951322
TMPRSS12	transmembrane protease, serine 12 [Source:HGNC Symbol;Acc:HGNC:28779]	1.1629393
TMPRSS15	transmembrane protease, serine 15 [Source:HGNC Symbol;Acc:HGNC:9490]	1.0570371
TMPRSS2	transmembrane protease, serine 2 [Source:HGNC Symbol;Acc:HGNC:11876]	-1.1248425
TMX3	thioredoxin related transmembrane protein 3 [Source:HGNC Symbol;Acc:HGNC:24718]	1.7814785
TNAP	alkaline phosphatase	-1.2356347
TNC	tenascin C [Source:HGNC Symbol;Acc:HGNC:5318]	-1.123085
TNF	tumor necrosis factor [Source:HGNC Symbol;Acc:HGNC:11892]	-1.5600228
TNFAIP1	TNF alpha induced protein 1 [Source:HGNC Symbol;Acc:HGNC:11894]	-3.285186
TNFAIP3	TNF alpha induced protein 3 [Source:HGNC Symbol;Acc:HGNC:11896]	-10.461266
TNFAIP8	TNF alpha induced protein 8 [Source:HGNC Symbol;Acc:HGNC:17260]	-1.6170085
TNFRSF10B	TNF receptor superfamily member 10b [Source:HGNC Symbol;Acc:HGNC:11905]	-2.246114
TNFRSF11A	TNF receptor superfamily member 11a [Source:HGNC Symbol;Acc:HGNC:11908]	-1.207558
TNFRSF12A	TNF receptor superfamily member 12A [Source:HGNC Symbol;Acc:HGNC:18152]	1.4738115
TNFRSF14	TNF receptor superfamily member 14 [Source:HGNC Symbol;Acc:HGNC:11912]	-1.350024
TNFRSF25	TNF receptor superfamily member 25 [Source:HGNC Symbol;Acc:HGNC:11910]	1.1280475

TNFRSF4	TNF receptor superfamily member 4 [Source:HGNC Symbol;Acc:HGNC:11918]	-2.6552737
TNFRSF6B	TNF receptor superfamily member 6b [Source:HGNC Symbol;Acc:HGNC:11921]	-1.38228975
TNFRSF9	TNF receptor superfamily member 9 [Source:HGNC Symbol;Acc:HGNC:11924]	-3.1794212
TNFSF10	tumor necrosis factor superfamily member 10 [Source:HGNC Symbol;Acc:HGNC:11925]	-2.179855
TNFSF12	tumor necrosis factor superfamily member 12 [Source:HGNC Symbol;Acc:HGNC:11927]	1.0728955
TNFSF15	tumor necrosis factor superfamily member 15 [Source:HGNC Symbol;Acc:HGNC:11931]	-1.6641451
TNFSF18	tumor necrosis factor superfamily member 18 [Source:HGNC Symbol;Acc:HGNC:11932]	-1.3544971
TNFSF4	tumor necrosis factor superfamily member 4 [Source:HGNC Symbol;Acc:HGNC:11934]	-1.2406721
TNFSF9	tumor necrosis factor superfamily member 9 [Source:HGNC Symbol;Acc:HGNC:11939]	-1.3042388
TNIK	TRAF2 and NCK interacting kinase [Source:HGNC Symbol;Acc:HGNC:30765]	1.2649497
TNIP1	TNFAIP3 interacting protein 1 [Source:HGNC Symbol;Acc:HGNC:16903]	-4.809692
TNIP2	TNFAIP3 interacting protein 2 [Source:HGNC Symbol;Acc:HGNC:19118]	-1.8379979
TNKS1BP1	tankyrase 1 binding protein 1 [Source:HGNC Symbol;Acc:HGNC:19081]	-1.4680911
TNNI3K	TNNI3 interacting kinase [Source:HGNC Symbol;Acc:HGNC:19661]	1.1072202
TNPO2	transportin 2 [Source:HGNC Symbol;Acc:HGNC:19998]	-1.2568765
TNRC6C	trinucleotide repeat containing 6C [Source:HGNC Symbol;Acc:HGNC:29318]	-1.3360386
TNS1	tensin 1 [Source:HGNC Symbol;Acc:HGNC:11973]	-1.1917463
TNS3	tensin 3 [Source:HGNC Symbol;Acc:HGNC:21616]	-1.4141543
TNXB	tenascin XB [Source:HGNC Symbol;Acc:HGNC:11976]	1.2142673
TOB2	transducer of ERBB2, 2 [Source:HGNC Symbol;Acc:HGNC:11980]	-1.301671
TOB2P1	transducer of ERBB2, 2 pseudogene 1 [Source:HGNC Symbol;Acc:HGNC:13986]	-1.1989537
TOMM20	translocase of outer mitochondrial membrane 20 [Source:HGNC Symbol;Acc:HGNC:20947]	1.2529776
TOMM22	translocase of outer mitochondrial membrane 22 [Source:HGNC Symbol;Acc:HGNC:18002]	1.1044419
TOMM34	translocase of outer mitochondrial membrane 34 [Source:HGNC Symbol;Acc:HGNC:15746]	-1.1401911
TOMM40	translocase of outer mitochondrial membrane 40 [Source:HGNC Symbol;Acc:HGNC:18001]	1.51621095
TOMM6	translocase of outer mitochondrial membrane 6 [Source:HGNC Symbol;Acc:HGNC:34528]	1.1691308
TOMM7	translocase of outer mitochondrial membrane 7 [Source:HGNC Symbol;Acc:HGNC:21648]	1.1434207
TONSL	tonsoku-like, DNA repair protein [Source:HGNC Symbol;Acc:HGNC:7801]	1.2066493
TOP1P2	topoisomerase (DNA) I pseudogene 2	1.4177773
TOPBP1	topoisomerase (DNA) II binding protein 1 [Source:HGNC Symbol;Acc:HGNC:17008]	1.4350045
TOR1AIP1	torsin 1A interacting protein 1 [Source:HGNC Symbol;Acc:HGNC:29456]	1.1124265
TOR1AIP2	torsin 1A interacting protein 2 [Source:HGNC Symbol;Acc:HGNC:24055]	1.0945134
TOR1B	torsin family 1 member B [Source:HGNC Symbol;Acc:HGNC:11995]	-1.2010314
TOR3A	torsin family 3 member A [Source:HGNC Symbol;Acc:HGNC:11997]	1.2394414
TOX2	TOX high mobility group box family member 2 [Source:HGNC Symbol;Acc:HGNC:16095]	1.3213398
TP53	tumor protein p53 [Source:HGNC Symbol;Acc:HGNC:11998]	-1.27585
TP53BP2	tumor protein p53 binding protein 2 [Source:HGNC Symbol;Acc:HGNC:12000]	-1.2665701
TP53INP1	tumor protein p53 inducible nuclear protein 1 [Source:HGNC Symbol;Acc:HGNC:18022]	1.2099884
TP53INP2	tumor protein p53 inducible nuclear protein 2 [Source:HGNC Symbol;Acc:HGNC:16104]	-1.1525676
TP73-AS1	TP73 antisense RNA 1 [Source:HGNC Symbol;Acc:HGNC:29052]	1.1244515
TPBG	trophoblast glycoprotein [Source:HGNC Symbol;Acc:HGNC:12004]	-1.3818378
TPD52	tumor protein D52 [Source:HGNC Symbol;Acc:HGNC:12005]	1.0762159
TPH1	tryptophan hydroxylase 1 [Source:HGNC Symbol;Acc:HGNC:12008]	1.1287633
TPH2	tryptophan hydroxylase 2 [Source:HGNC Symbol;Acc:HGNC:20692]	1.0575708
TPI1P2	triosephosphate isomerase 1 pseudogene 2 [Source:HGNC Symbol;Acc:HGNC:38069]	-1.1306612
TPM3	tropomyosin 3 [Source:HGNC Symbol;Acc:HGNC:12012]	-1.2672457
TPO	thyroid peroxidase [Source:HGNC Symbol;Acc:HGNC:12015]	-1.1701678
TPRG1	tumor protein p63 regulated 1 [Source:HGNC Symbol;Acc:HGNC:24759]	-1.056538
TPRKB	TP53RK binding protein [Source:HGNC Symbol;Acc:HGNC:24259]	1.1895676
TPSD1	tryptase delta 1 [Source:HGNC Symbol;Acc:HGNC:14118]	1.0654223
TPST1	tyrosylprotein sulfotransferase 1 [Source:HGNC Symbol;Acc:HGNC:12020]	-1.2655418
TPTE2P1	ane phosphoinositide 3-phosphatase and tensin homolog 2 pseudogene 1 [Source:HGNC Symbol;Acc:H	1.0764427
TRAF1	TNF receptor associated factor 1 [Source:HGNC Symbol;Acc:HGNC:12031]	-14.809285
TRAF2	TNF receptor associated factor 2 [Source:HGNC Symbol;Acc:HGNC:12032]	-1.43029035
TRAF3	TNF receptor associated factor 3 [Source:HGNC Symbol;Acc:HGNC:12033]	-2.0109203
TRAF3IP2-AS1	TRAF3IP2 antisense RNA 1 [Source:HGNC Symbol;Acc:HGNC:40005]	-1.1228024
TRAF6	TNF receptor associated factor 6 [Source:HGNC Symbol;Acc:HGNC:12036]	-1.3785845
TRAF7	TNF receptor associated factor 7 [Source:HGNC Symbol;Acc:HGNC:20456]	1.1727204
TRAFD1	TRAF-type zinc finger domain containing 1 [Source:HGNC Symbol;Acc:HGNC:24808]	-1.1418102
TRAK1	trafficking kinesin protein 1 [Source:HGNC Symbol;Acc:HGNC:29947]	-1.163809
TRAK2	trafficking kinesin protein 2 [Source:HGNC Symbol;Acc:HGNC:13206]	1.3494784
TRAM2	translocation associated membrane protein 2 [Source:HGNC Symbol;Acc:HGNC:16855]	1.5059513
TRAPPC10	trafficking protein particle complex 10 [Source:HGNC Symbol;Acc:HGNC:11868]	-1.3068522
TRAPPC2P1	trafficking protein particle complex 2B	-1.1783549
TRAPPC6B	trafficking protein particle complex 6B [Source:HGNC Symbol;Acc:HGNC:23066]	1.1573316
TREM1	triggering receptor expressed on myeloid cells 1 [Source:HGNC Symbol;Acc:HGNC:17760]	1.2303058
TRERF1	transcriptional regulating factor 1 [Source:HGNC Symbol;Acc:HGNC:18273]	1.35269
TRIB1	tribbles pseudokinase 1 [Source:HGNC Symbol;Acc:HGNC:16891]	-2.284549
TRIL	TLR4 interactor with leucine rich repeats [Source:HGNC Symbol;Acc:HGNC:22200]	1.5817469
TRIM10	tripartite motif containing 10 [Source:HGNC Symbol;Acc:HGNC:10072]	-1.0960135
TRIM11	tripartite motif containing 11 [Source:HGNC Symbol;Acc:HGNC:16281]	-1.2189177
TRIM13	tripartite motif containing 13 [Source:HGNC Symbol;Acc:HGNC:9976]	1.27498165
TRIM14	tripartite motif containing 14 [Source:HGNC Symbol;Acc:HGNC:16283]	-1.2784243
TRIM16L	tripartite motif containing 16-like [Source:HGNC Symbol;Acc:HGNC:32670]	-2.223522
TRIM21	tripartite motif containing 21 [Source:HGNC Symbol;Acc:HGNC:11312]	-1.5831492
TRIM25	tripartite motif containing 25 [Source:HGNC Symbol;Acc:HGNC:12932]	-1.4707078
TRIM27	tripartite motif containing 27 [Source:HGNC Symbol;Acc:HGNC:9975]	-1.1455419
TRIM32	tripartite motif containing 32 [Source:HGNC Symbol;Acc:HGNC:16380]	-1.142216
TRIM35	tripartite motif containing 35 [Source:HGNC Symbol;Acc:HGNC:16285]	-1.3243946
TRIM36	tripartite motif containing 36 [Source:HGNC Symbol;Acc:HGNC:16280]	1.0607179
TRIM39	tripartite motif containing 39 [Source:HGNC Symbol;Acc:HGNC:10065]	-1.1761115
TRIM42	tripartite motif containing 42 [Source:HGNC Symbol;Acc:HGNC:19014]	-1.0968263
TRIM45	tripartite motif containing 45 [Source:HGNC Symbol;Acc:HGNC:19018]	1.335756
TRIM47	tripartite motif containing 47 [Source:HGNC Symbol;Acc:HGNC:19020]	-2.7629037
TRIM49	tripartite motif containing 49 [Source:HGNC Symbol;Acc:HGNC:13431]	-1.1847588
TRIM56	tripartite motif containing 56 [Source:HGNC Symbol;Acc:HGNC:19028]	-1.7186335

TRIM59	tripartite motif containing 59 [Source:HGNC Symbol;Acc:HGNC:30834]	1.3179337
TRIM6	tripartite motif containing 6 [Source:HGNC Symbol;Acc:HGNC:16277]	1.2045777
TRIM63	tripartite motif containing 63 [Source:HGNC Symbol;Acc:HGNC:16007]	1.0726925
TRIM66	tripartite motif containing 66 [Source:HGNC Symbol;Acc:HGNC:29005]	1.1645573
TRIO	trio Rho guanine nucleotide exchange factor [Source:HGNC Symbol;Acc:HGNC:12303]	-1.3608733
TRIOBP	TRIO and F-actin binding protein [Source:HGNC Symbol;Acc:HGNC:17009]	-1.3349626
TRIP10	thyroid hormone receptor interactor 10 [Source:HGNC Symbol;Acc:HGNC:12304]	-1.4320321
TRMT112	tRNA methyltransferase 11-2 homolog (S. cerevisiae) [Source:HGNC Symbol;Acc:HGNC:26940]	-1.1423287
TRMT5	tRNA methyltransferase 5 [Source:HGNC Symbol;Acc:HGNC:23141]	1.1948779
TRNT1	tRNA nucleotidyl transferase 1 [Source:HGNC Symbol;Acc:HGNC:17341]	-1.0948597
TRO	trophinin [Source:HGNC Symbol;Acc:HGNC:12326]	-1.1236232
TRPC6	ansient receptor potential cation channel subfamily C member 6 [Source:HGNC Symbol;Acc:HGNC:1233	-1.3125032
TRPM2	insient receptor potential cation channel subfamily M member 2 [Source:HGNC Symbol;Acc:HGNC:123	1.1360066
TRPM8	insient receptor potential cation channel subfamily M member 8 [Source:HGNC Symbol;Acc:HGNC:179	-1.104008
TRPV6	ansient receptor potential cation channel subfamily V member 6 [Source:HGNC Symbol;Acc:HGNC:140	-1.1317482
TRUB1	TruB pseudouridine synthase family member 1 [Source:HGNC Symbol;Acc:HGNC:16060]	1.4186193
TSC22D4	TSC22 domain family member 4 [Source:HGNC Symbol;Acc:HGNC:21696]	1.2351038
TSFM	Ts translation elongation factor, mitochondrial [Source:HGNC Symbol;Acc:HGNC:12367]	1.0869668
TSG101	tumor susceptibility 101 [Source:HGNC Symbol;Acc:HGNC:15971]	-1.2770367
TSGA10	testis specific 10 [Source:HGNC Symbol;Acc:HGNC:14927]	1.0654902
TSHZ1	teashirt zinc finger homeobox 1 [Source:HGNC Symbol;Acc:HGNC:10669]	1.3527504
TSKS	testis specific serine kinase substrate [Source:HGNC Symbol;Acc:HGNC:30719]	1.0732895
TSKU	tsukushi, small leucine rich proteoglycan [Source:HGNC Symbol;Acc:HGNC:28850]	-1.186316
TSL	testis-expressed, seven-twelve, leukemia	-1.1505249
TSLP	thymic stromal lymphopoietin [Source:HGNC Symbol;Acc:HGNC:30743]	1.1000466
TSNAX	translin associated factor X [Source:HGNC Symbol;Acc:HGNC:12380]	1.2380335
TSPAN1	tetraspanin 1 [Source:HGNC Symbol;Acc:HGNC:20657]	-1.1788192
TSPAN12	tetraspanin 12 [Source:HGNC Symbol;Acc:HGNC:21641]	1.5059534
TSPAN14	tetraspanin 14 [Source:HGNC Symbol;Acc:HGNC:23303]	1.358315233
TSPAN18	tetraspanin 18 [Source:HGNC Symbol;Acc:HGNC:20660]	-1.1542375
TSPYL2	TSPY like 2 [Source:HGNC Symbol;Acc:HGNC:24358]	1.335354
TSPYL5	TSPY like 5 [Source:HGNC Symbol;Acc:HGNC:29367]	-1.1418211
TSPYL6	TSPY like 6 [Source:HGNC Symbol;Acc:HGNC:14521]	1.0548588
TTC24	tetratricopeptide repeat domain 24 [Source:HGNC Symbol;Acc:HGNC:32348]	-1.1401285
TTC28	tetratricopeptide repeat domain 28 [Source:HGNC Symbol;Acc:HGNC:29179]	-1.0991809
TTC30B	tetratricopeptide repeat domain 30B [Source:HGNC Symbol;Acc:HGNC:26425]	1.099937
TTC34	tetratricopeptide repeat domain 34 [Source:HGNC Symbol;Acc:HGNC:34297]	-1.0974952
TTC39A	tetratricopeptide repeat domain 39A [Source:HGNC Symbol;Acc:HGNC:18657]	-1.4693576
TTC39C	tetratricopeptide repeat domain 39C [Source:HGNC Symbol;Acc:HGNC:26595]	-1.23741475
TTC5	tetratricopeptide repeat domain 5 [Source:HGNC Symbol;Acc:HGNC:19274]	1.3890661
TTC9C	tetratricopeptide repeat domain 9C [Source:HGNC Symbol;Acc:HGNC:28432]	-1.1699338
TTL	tubulin tyrosine ligase [Source:HGNC Symbol;Acc:HGNC:21586]	-1.1386338
TTLL4	tubulin tyrosine ligase like 4 [Source:HGNC Symbol;Acc:HGNC:28976]	-1.2866039
TTLL9	tubulin tyrosine ligase like 9 [Source:HGNC Symbol;Acc:HGNC:16118]	-1.0868782
TTTY13	testis-specific transcript, Y-linked 13 (non-protein coding) [Source:HGNC Symbol;Acc:HGNC:18494]	-1.0855936
TTTY14	testis-specific transcript, Y-linked 14 (non-protein coding) [Source:HGNC Symbol;Acc:HGNC:18495]	-1.3316395
TTTY3	testis-specific transcript, Y-linked 3 (non-protein coding) [Source:HGNC Symbol;Acc:HGNC:16480]	1.1542276
TTTY4C	testis-specific transcript, Y-linked 4C (non-protein coding) [Source:HGNC Symbol;Acc:HGNC:31892]	1.0599645
TUB	tubby bipartite transcription factor [Source:HGNC Symbol;Acc:HGNC:12406]	1.1466547
TUBA4A	tubulin alpha 4a [Source:HGNC Symbol;Acc:HGNC:12407]	1.180965
TUBA8	tubulin alpha 8 [Source:HGNC Symbol;Acc:HGNC:12410]	1.3726145
TUBAL3	tubulin alpha like 3 [Source:HGNC Symbol;Acc:HGNC:23534]	1.0916649
TUBB2A	tubulin beta 2A class IIa [Source:HGNC Symbol;Acc:HGNC:12412]	-1.3976978
TUBB2B	tubulin beta 2B class IIb [Source:HGNC Symbol;Acc:HGNC:30829]	-2.0936594
TUBB8	tubulin beta 8 class VIII [Source:HGNC Symbol;Acc:HGNC:20773]	-1.2128648
TUBBP5	tubulin beta pseudogene 5 [Source:HGNC Symbol;Acc:HGNC:23674]	-1.1514122
TUBG2	tubulin gamma 2 [Source:HGNC Symbol;Acc:HGNC:12419]	-1.1193223
TUBGCP3	tubulin gamma complex associated protein 3 [Source:HGNC Symbol;Acc:HGNC:18598]	1.0931134
TUBGCP5	tubulin gamma complex associated protein 5 [Source:HGNC Symbol;Acc:HGNC:18600]	1.2224793
TULP2	tubby like protein 2 [Source:HGNC Symbol;Acc:HGNC:12424]	1.1218727
TWIST2	twist family bHLH transcription factor 2 [Source:HGNC Symbol;Acc:HGNC:20670]	-1.421087
TWISTNB	TWIST neighbor [Source:HGNC Symbol;Acc:HGNC:18027]	1.4070615
TWSG1	twisted gastrulation BMP signaling modulator 1 [Source:HGNC Symbol;Acc:HGNC:12429]	1.4226744
TXK	TXK tyrosine kinase [Source:HGNC Symbol;Acc:HGNC:12434]	1.2076851
TXLNB	taxilin beta [Source:HGNC Symbol;Acc:HGNC:21617]	1.0959263
TXNDC15	thioredoxin domain containing 15 [Source:HGNC Symbol;Acc:HGNC:20652]	-1.2350338
TXNDC9	thioredoxin domain containing 9 [Source:HGNC Symbol;Acc:HGNC:24110]	1.6127372
TXNL4A	thioredoxin like 4A [Source:HGNC Symbol;Acc:HGNC:30551]	1.1648793
TYK2	tyrosine kinase 2 [Source:HGNC Symbol;Acc:HGNC:12440]	-1.2067227
TYSND1	trypsin domain containing 1 [Source:HGNC Symbol;Acc:HGNC:28531]	1.2387348
UBA2	ubiquitin like modifier activating enzyme 2 [Source:HGNC Symbol;Acc:HGNC:30661]	1.27021895
UBA7	ubiquitin like modifier activating enzyme 7 [Source:HGNC Symbol;Acc:HGNC:12471]	-1.2822809
UBAC1	UBA domain containing 1 [Source:HGNC Symbol;Acc:HGNC:30221]	-1.212058
UBAP1	ubiquitin associated protein 1 [Source:HGNC Symbol;Acc:HGNC:12461]	-1.1657261
UBAP2	ubiquitin associated protein 2 [Source:HGNC Symbol;Acc:HGNC:14185]	-1.0620881
UBAP2L	ubiquitin associated protein 2 like [Source:HGNC Symbol;Acc:HGNC:29877]	-1.17111275
UBD	ubiquitin D [Source:HGNC Symbol;Acc:HGNC:18795]	-13.445234
UBE2A	ubiquitin conjugating enzyme E2 A [Source:HGNC Symbol;Acc:HGNC:12472]	-1.1870227
UBE2CBP	ubiquitin protein ligase E3D	-1.0794846
UBE2D2	ubiquitin conjugating enzyme E2 D2 [Source:HGNC Symbol;Acc:HGNC:12475]	-1.1681869
UBE2D4	ubiquitin conjugating enzyme E2 D4 (putative) [Source:HGNC Symbol;Acc:HGNC:21647]	-1.1512454
UBE2E2	ubiquitin conjugating enzyme E2 E2 [Source:HGNC Symbol;Acc:HGNC:12478]	-1.2058421
UBE2H	ubiquitin conjugating enzyme E2 H [Source:HGNC Symbol;Acc:HGNC:12484]	-1.2265989
UBE2J2	ubiquitin conjugating enzyme E2 J2 [Source:HGNC Symbol;Acc:HGNC:19268]	-1.092073
UBE2L6	ubiquitin conjugating enzyme E2 L6 [Source:HGNC Symbol;Acc:HGNC:12490]	-1.2425293
UBE2O	ubiquitin conjugating enzyme E2 O [Source:HGNC Symbol;Acc:HGNC:29554]	-1.2188761

UBE2Q2	ubiquitin conjugating enzyme E2 Q2 [Source:HGNC Symbol;Acc:HGNC:19248]	1.301491
UBE2S	ubiquitin conjugating enzyme E2 S [Source:HGNC Symbol;Acc:HGNC:17895]	1.1708115
UBE2T	ubiquitin conjugating enzyme E2 T [Source:HGNC Symbol;Acc:HGNC:25009]	1.3093588
UBE2Z	ubiquitin conjugating enzyme E2 Z [Source:HGNC Symbol;Acc:HGNC:25847]	-1.199173
UBE3B	ubiquitin protein ligase E3B [Source:HGNC Symbol;Acc:HGNC:13478]	-1.12858895
UBFD1	ubiquitin family domain containing 1 [Source:HGNC Symbol;Acc:HGNC:30565]	-1.2599049
UBQLN2	ubiquilin 2 [Source:HGNC Symbol;Acc:HGNC:12509]	-1.3322608
UBQLN3	ubiquilin 3 [Source:HGNC Symbol;Acc:HGNC:12510]	-1.1765635
UBR4	ubiquitin protein ligase E3 component n-recognin 4 [Source:HGNC Symbol;Acc:HGNC:30313]	-1.2233404
UBR7	ubiquitin protein ligase E3 component n-recognin 7 (putative) [Source:HGNC Symbol;Acc:HGNC:20344]	1.1899208
UBTF	upstream binding transcription factor, RNA polymerase I [Source:HGNC Symbol;Acc:HGNC:12511]	1.3408439
UBXN8	UBX domain protein 8 [Source:HGNC Symbol;Acc:HGNC:30307]	1.0647758
UCHL5	ubiquitin C-terminal hydrolase L5 [Source:HGNC Symbol;Acc:HGNC:19678]	1.34053575
UCMA	upper zone of growth plate and cartilage matrix associated [Source:HGNC Symbol;Acc:HGNC:25205]	-1.0508285
UFD1L	ubiquitin fusion degradation 1 like (yeast) [Source:HGNC Symbol;Acc:HGNC:12520]	-1.1013578
UGGT2	UDP-glucose glycoprotein glucosyltransferase 2 [Source:HGNC Symbol;Acc:HGNC:15664]	1.309824
UHRF1BP1	UHRF1 binding protein 1 [Source:HGNC Symbol;Acc:HGNC:21216]	-1.2545043
ULBP2	UL16 binding protein 2 [Source:HGNC Symbol;Acc:HGNC:14894]	1.1818653
ULK4	unc-51 like kinase 4 [Source:HGNC Symbol;Acc:HGNC:15784]	-1.0974432
UMODL1	uromodulin like 1 [Source:HGNC Symbol;Acc:HGNC:12560]	-1.227653333
UNC13B	unc-13 homolog B [Source:HGNC Symbol;Acc:HGNC:12566]	-1.1886598
UNC5D	unc-5 netrin receptor D [Source:HGNC Symbol;Acc:HGNC:18634]	1.0867689
UNG	uracil DNA glycosylase [Source:HGNC Symbol;Acc:HGNC:12572]	1.2404886
UNKL	unkempt family like zinc finger [Source:HGNC Symbol;Acc:HGNC:14184]	-1.2342783
UPB1	beta-ureidopropionase 1 [Source:HGNC Symbol;Acc:HGNC:16297]	-1.0942532
UPK3B	uroplakin 3B [Source:HGNC Symbol;Acc:HGNC:21444]	-1.1277776
UPP1	uridine phosphorylase 1 [Source:HGNC Symbol;Acc:HGNC:12576]	-1.43313055
UPRT	uracil phosphoribosyltransferase homolog [Source:HGNC Symbol;Acc:HGNC:28334]	1.13826065
UQCC	ubiquinol-cytochrome c reductase complex assembly factor 1	-1.064101
URB1	URB1 ribosome biogenesis 1 homolog (S. cerevisiae) [Source:HGNC Symbol;Acc:HGNC:17344]	-1.146531
URGCP	upregulator of cell proliferation [Source:HGNC Symbol;Acc:HGNC:30890]	-1.2461512
USH2A	usherin [Source:HGNC Symbol;Acc:HGNC:12601]	1.1208235
USP11	ubiquitin specific peptidase 11 [Source:HGNC Symbol;Acc:HGNC:12609]	-1.1985438
USP17	ubiquitin specific peptidase 17-like family member 9, pseudogene	1.0650381
USP31	ubiquitin specific peptidase 31 [Source:HGNC Symbol;Acc:HGNC:20060]	-1.5834881
USP33	ubiquitin specific peptidase 33 [Source:HGNC Symbol;Acc:HGNC:20059]	1.1261317
USP36	ubiquitin specific peptidase 36 [Source:HGNC Symbol;Acc:HGNC:20062]	-1.4490665
USP38	ubiquitin specific peptidase 38 [Source:HGNC Symbol;Acc:HGNC:20067]	1.2359266
USP45	ubiquitin specific peptidase 45 [Source:HGNC Symbol;Acc:HGNC:20080]	1.1660931
USP49	ubiquitin specific peptidase 49 [Source:HGNC Symbol;Acc:HGNC:20078]	-1.1702685
USP5	ubiquitin specific peptidase 5 [Source:HGNC Symbol;Acc:HGNC:12628]	1.2452204
USP51	ubiquitin specific peptidase 51 [Source:HGNC Symbol;Acc:HGNC:23086]	1.1184583
USP54	ubiquitin specific peptidase 54 [Source:HGNC Symbol;Acc:HGNC:23513]	-1.1127272
UVRAG	UV radiation resistance associated [Source:HGNC Symbol;Acc:HGNC:12640]	-1.4342183
VAC14	Vac14, PIKFYVE complex component [Source:HGNC Symbol;Acc:HGNC:25507]	-1.1425991
VAMP7	vesicle associated membrane protein 7 [Source:HGNC Symbol;Acc:HGNC:11486]	1.380774
VARS2	valyl-tRNA synthetase 2, mitochondrial [Source:HGNC Symbol;Acc:HGNC:21642]	1.209421
VASH1	vasohibin 1 [Source:HGNC Symbol;Acc:HGNC:19964]	1.2086797
VAT1	vesicle amine transport 1 [Source:HGNC Symbol;Acc:HGNC:16919]	1.48712755
VAV2	vav guanine nucleotide exchange factor 2 [Source:HGNC Symbol;Acc:HGNC:12658]	-1.5332677
VAX2	ventral anterior homeobox 2 [Source:HGNC Symbol;Acc:HGNC:12661]	-1.266751
VCAM1	vascular cell adhesion molecule 1 [Source:HGNC Symbol;Acc:HGNC:12663]	-10.085852
VCY	variable charge, Y-linked [Source:HGNC Symbol;Acc:HGNC:12668]	-1.1203098
VDAC2	voltage dependent anion channel 2 [Source:HGNC Symbol;Acc:HGNC:12672]	-1.2801745
VDR	vitamin D (1,25- dihydroxyvitamin D3) receptor [Source:HGNC Symbol;Acc:HGNC:12679]	-1.1874926
VEGFA	vascular endothelial growth factor A [Source:HGNC Symbol;Acc:HGNC:12680]	-1.39839035
VGLL4	vestigial like family member 4 [Source:HGNC Symbol;Acc:HGNC:28966]	-1.4039645
VIT	vitrin [Source:HGNC Symbol;Acc:HGNC:12697]	-1.2075036
VMA21	VMA21 vacuolar H+-ATPase homolog (S. cerevisiae) [Source:HGNC Symbol;Acc:HGNC:22082]	1.4221191
VNN2	vanin 2 [Source:HGNC Symbol;Acc:HGNC:12706]	-1.1859345
VPRBP	DDB1 and CUL4 associated factor 1	-1.1331074
VPS24	charged multivesicular body protein 3	1.1542709
VPS26A	VPS26, retromer complex component A [Source:HGNC Symbol;Acc:HGNC:12711]	1.2515216
VPS35	VPS35, retromer complex component [Source:HGNC Symbol;Acc:HGNC:13487]	1.1885247
VPS37A	VPS37A, ESCRT-I subunit [Source:HGNC Symbol;Acc:HGNC:24928]	-1.2342338
VPS37C	VPS37C, ESCRT-I subunit [Source:HGNC Symbol;Acc:HGNC:26097]	-1.3744162
VPS37D	VPS37D, ESCRT-I subunit [Source:HGNC Symbol;Acc:HGNC:18287]	1.467897
VPS8	VPS8, CORVET complex subunit [Source:HGNC Symbol;Acc:HGNC:29122]	1.0975047
VRTN	vertebrae development associated [Source:HGNC Symbol;Acc:HGNC:20223]	1.0938108
VSIG10	V-set and immunoglobulin domain containing 10 [Source:HGNC Symbol;Acc:HGNC:26078]	1.2436857
VSTM1	V-set and transmembrane domain containing 1 [Source:HGNC Symbol;Acc:HGNC:29455]	-2.0998054
VSTM2A	V-set and transmembrane domain containing 2A [Source:HGNC Symbol;Acc:HGNC:28499]	1.0857421
VTI1A	vesicle transport through interaction with t-SNAREs 1A [Source:HGNC Symbol;Acc:HGNC:17792]	-1.1884581
WARS	tryptophanyl-tRNA synthetase [Source:HGNC Symbol;Acc:HGNC:12729]	-1.3721117
WASF2	WAS protein family member 2 [Source:HGNC Symbol;Acc:HGNC:12733]	-1.462956
WASH1	WAS protein family homolog 1 [Source:HGNC Symbol;Acc:HGNC:24361]	1.1659089
WDFY4	WDFY family member 4 [Source:HGNC Symbol;Acc:HGNC:29323]	1.26858025
WDHD1	WD repeat and HMG-box DNA binding protein 1 [Source:HGNC Symbol;Acc:HGNC:23170]	1.5514395
WDR1	WD repeat domain 1 [Source:HGNC Symbol;Acc:HGNC:12754]	-1.2626114
WDR17	WD repeat domain 17 [Source:HGNC Symbol;Acc:HGNC:16661]	1.0526505
WDR19	WD repeat domain 19 [Source:HGNC Symbol;Acc:HGNC:18340]	1.1199018
WDR33	WD repeat domain 33 [Source:HGNC Symbol;Acc:HGNC:25651]	-1.164613
WDR43	WD repeat domain 43 [Source:HGNC Symbol;Acc:HGNC:28945]	1.3135468
WDR45	WD repeat domain 45 [Source:HGNC Symbol;Acc:HGNC:28912]	-1.1269876
WDR45L	WD repeat domain 45B	-1.2680389
WDR48	WD repeat domain 48 [Source:HGNC Symbol;Acc:HGNC:30914]	-1.2804276

WDR52	cilia and flagella associated protein 44	1.0673912
WDR55	WD repeat domain 55 [Source:HGNC Symbol;Acc:HGNC:25971]	-1.1077034
WDR59	WD repeat domain 59 [Source:HGNC Symbol;Acc:HGNC:25706]	-1.1465641
WDR72	WD repeat domain 72 [Source:HGNC Symbol;Acc:HGNC:26790]	1.1288078
WDR82	WD repeat domain 82 [Source:HGNC Symbol;Acc:HGNC:28826]	-1.2486362
WDR83	WD repeat domain 83 [Source:HGNC Symbol;Acc:HGNC:32672]	-1.1777533
WDR86	WD repeat domain 86 [Source:HGNC Symbol;Acc:HGNC:28020]	1.0679293
WDR89	WD repeat domain 89 [Source:HGNC Symbol;Acc:HGNC:20489]	1.1597314
WEE2	WEE1 homolog 2 (S. pombe) [Source:HGNC Symbol;Acc:HGNC:19684]	-1.0791117
WFDC2	WAP four-disulfide core domain 2 [Source:HGNC Symbol;Acc:HGNC:15939]	-1.1406696
WFDC8	WAP four-disulfide core domain 8 [Source:HGNC Symbol;Acc:HGNC:16163]	-1.1687293
WHAMM	:ein homolog associated with actin, golgi membranes and microtubules [Source:HGNC Symbol;Acc:HGN	-1.2539296
WHSC1L1	Wolf-Hirschhorn syndrome candidate 1-like 1 [Source:HGNC Symbol;Acc:HGNC:12767]	1.1519984
WIPF1	WAS/WASL interacting protein family member 1 [Source:HGNC Symbol;Acc:HGNC:12736]	1.1068803
WIPI2	WD repeat domain, phosphoinositide interacting 2 [Source:HGNC Symbol;Acc:HGNC:32225]	-1.163766
WISP2	WNT1 inducible signaling pathway protein 2 [Source:HGNC Symbol;Acc:HGNC:12770]	1.0572568
WISP3	WNT1 inducible signaling pathway protein 3 [Source:HGNC Symbol;Acc:HGNC:12771]	-1.0808573
WLS	wntless Wnt ligand secretion mediator [Source:HGNC Symbol;Acc:HGNC:30238]	-1.1506457
WNT1	Wnt family member 1 [Source:HGNC Symbol;Acc:HGNC:12774]	-1.09567875
WNT3A	Wnt family member 3A [Source:HGNC Symbol;Acc:HGNC:15983]	-1.0637966
WNT5A	Wnt family member 5A [Source:HGNC Symbol;Acc:HGNC:12784]	-1.6691802
WTAP	Wilms tumor 1 associated protein [Source:HGNC Symbol;Acc:HGNC:16846]	-1.129792
WTIP	Wilms tumor 1 interacting protein [Source:HGNC Symbol;Acc:HGNC:20964]	1.2016112
WWC1	WW and C2 domain containing 1 [Source:HGNC Symbol;Acc:HGNC:29435]	-2.5356345
WWP2	WW domain containing E3 ubiquitin protein ligase 2 [Source:HGNC Symbol;Acc:HGNC:16804]	-1.4202142
XAF1	XIAP associated factor 1 [Source:HGNC Symbol;Acc:HGNC:30932]	-1.1740229
XAGE5	X antigen family member 5 [Source:HGNC Symbol;Acc:HGNC:30930]	1.069012
XBP1	X-box binding protein 1 [Source:HGNC Symbol;Acc:HGNC:12801]	-1.1260592
XCR1	X-C motif chemokine receptor 1 [Source:HGNC Symbol;Acc:HGNC:1625]	-1.2454274
XKR8	XK related 8 [Source:HGNC Symbol;Acc:HGNC:25508]	-1.1272233
XPC	PC complex subunit, DNA damage recognition and repair factor [Source:HGNC Symbol;Acc:HGNC:1281(	-1.2646662
XPNPEP1	X-prolyl aminopeptidase 1 [Source:HGNC Symbol;Acc:HGNC:12822]	-1.1657184
XPO5	exportin 5 [Source:HGNC Symbol;Acc:HGNC:17675]	-1.16941175
XYLT1	xylosyltransferase 1 [Source:HGNC Symbol;Acc:HGNC:15516]	1.1594994
YARS2	tyrosyl-tRNA synthetase 2 [Source:HGNC Symbol;Acc:HGNC:24249]	-1.1056728
YBX2	Y-box binding protein 2 [Source:HGNC Symbol;Acc:HGNC:17948]	-1.1731076
YIPF5	Yip1 domain family member 5 [Source:HGNC Symbol;Acc:HGNC:24877]	1.2504514
YIPF6	Yip1 domain family member 6 [Source:HGNC Symbol;Acc:HGNC:28304]	1.12574
YLPM1	YLP motif containing 1 [Source:HGNC Symbol;Acc:HGNC:17798]	1.209155
YPEL1	yippee like 1 [Source:HGNC Symbol;Acc:HGNC:12845]	-1.205815
YPEL2	yippee like 2 [Source:HGNC Symbol;Acc:HGNC:18326]	-1.8998111
YPEL3	yippee like 3 [Source:HGNC Symbol;Acc:HGNC:18327]	1.229277
YPEL5	yippee like 5 [Source:HGNC Symbol;Acc:HGNC:18329]	-1.3385253
YRDC	yrdC N6-threonylcarbamoyltransferase domain containing [Source:HGNC Symbol;Acc:HGNC:28905]	1.1165332
ZADH2	zinc binding alcohol dehydrogenase domain containing 2 [Source:HGNC Symbol;Acc:HGNC:28697]	1.268781
ZAN	zonadhesin (gene/pseudogene) [Source:HGNC Symbol;Acc:HGNC:12857]	-1.1670691
ZBTB17	zinc finger and BTB domain containing 17 [Source:HGNC Symbol;Acc:HGNC:12936]	-1.2479687
ZBTB24	zinc finger and BTB domain containing 24 [Source:HGNC Symbol;Acc:HGNC:21143]	1.2514802
ZBTB3	zinc finger and BTB domain containing 3 [Source:HGNC Symbol;Acc:HGNC:22918]	-1.0880046
ZBTB4	zinc finger and BTB domain containing 4 [Source:HGNC Symbol;Acc:HGNC:23847]	1.1348364
ZBTB40	zinc finger and BTB domain containing 40 [Source:HGNC Symbol;Acc:HGNC:29045]	-1.0896063
ZBTB45	zinc finger and BTB domain containing 45 [Source:HGNC Symbol;Acc:HGNC:23715]	1.2066642
ZBTB46	zinc finger and BTB domain containing 46 [Source:HGNC Symbol;Acc:HGNC:16094]	-1.5893101
ZBTB49	zinc finger and BTB domain containing 49 [Source:HGNC Symbol;Acc:HGNC:19883]	-1.1526476
ZBTB5	zinc finger and BTB domain containing 5 [Source:HGNC Symbol;Acc:HGNC:23836]	-1.4475629
ZBTB8OS	zinc finger and BTB domain containing 8 opposite strand [Source:HGNC Symbol;Acc:HGNC:24094]	1.1038494
ZC3H12A	zinc finger CCCH-type containing 12A [Source:HGNC Symbol;Acc:HGNC:26259]	-2.0054114
ZC3H4	zinc finger CCCH-type containing 4 [Source:HGNC Symbol;Acc:HGNC:17808]	-1.1597759
ZC3H7B	zinc finger CCCH-type containing 7B [Source:HGNC Symbol;Acc:HGNC:30869]	-2.3005502
ZC3HAV1	zinc finger CCCH-type containing, antiviral 1 [Source:HGNC Symbol;Acc:HGNC:23721]	-1.2117912
ZCCHC2	zinc finger CCHC-type containing 2 [Source:HGNC Symbol;Acc:HGNC:22916]	1.8132063
ZCCHC24	zinc finger CCHC-type containing 24 [Source:HGNC Symbol;Acc:HGNC:26911]	1.1488322
ZCCHC5	zinc finger CCHC-type containing 5 [Source:HGNC Symbol;Acc:HGNC:22997]	1.1156474
ZCWPW1	zinc finger CW-type and PWWP domain containing 1 [Source:HGNC Symbol;Acc:HGNC:23486]	-1.163759
ZDHHC14	zinc finger DHHC-type containing 14 [Source:HGNC Symbol;Acc:HGNC:20341]	-1.2710094
ZDHHC15	zinc finger DHHC-type containing 15 [Source:HGNC Symbol;Acc:HGNC:20342]	1.0505561
ZDHHC20	zinc finger DHHC-type containing 20 [Source:HGNC Symbol;Acc:HGNC:20749]	-1.1040993
ZDHHC21	zinc finger DHHC-type containing 21 [Source:HGNC Symbol;Acc:HGNC:20750]	1.177513
ZDHHC23	zinc finger DHHC-type containing 23 [Source:HGNC Symbol;Acc:HGNC:28654]	-1.123558
ZDHHC9	zinc finger DHHC-type containing 9 [Source:HGNC Symbol;Acc:HGNC:18475]	-1.1001143
ZFAND1	zinc finger AN1-type containing 1 [Source:HGNC Symbol;Acc:HGNC:25858]	1.4367543
ZFAND2A	zinc finger AN1-type containing 2A [Source:HGNC Symbol;Acc:HGNC:28073]	-1.5533625
ZFAND3	zinc finger AN1-type containing 3 [Source:HGNC Symbol;Acc:HGNC:18019]	-1.0846007
ZFAND5	zinc finger AN1-type containing 5 [Source:HGNC Symbol;Acc:HGNC:13008]	-1.2968843
ZFAT	zinc finger and AT-hook domain containing [Source:HGNC Symbol;Acc:HGNC:19899]	-1.084601
ZFHX2	zinc finger homeobox 2 [Source:HGNC Symbol;Acc:HGNC:20152]	-2.465378
ZFP1	ZFP1 zinc finger protein [Source:HGNC Symbol;Acc:HGNC:23328]	1.2775093
ZFP2	ZFP2 zinc finger protein [Source:HGNC Symbol;Acc:HGNC:26138]	1.105657
ZFP36	ZFP36 ring finger protein [Source:HGNC Symbol;Acc:HGNC:12862]	-1.1340337
ZFP36L1	ZFP36 ring finger protein like 1 [Source:HGNC Symbol;Acc:HGNC:1107]	-1.76042705
ZFP37	ZFP37 zinc finger protein [Source:HGNC Symbol;Acc:HGNC:12863]	1.112232
ZFP92	ZFP92 zinc finger protein [Source:HGNC Symbol;Acc:HGNC:12865]	-1.1326898
ZFPM2	zinc finger protein, FOG family member 2 [Source:HGNC Symbol;Acc:HGNC:16700]	-2.0785728
ZFX	zinc finger protein, X-linked [Source:HGNC Symbol;Acc:HGNC:12869]	1.1251149
ZFYVE1	zinc finger FYVE-type containing 1 [Source:HGNC Symbol;Acc:HGNC:13180]	-1.1824155
ZFYVE21	zinc finger FYVE-type containing 21 [Source:HGNC Symbol;Acc:HGNC:20760]	1.1951897

ZGLP1	zinc finger, GATA-like protein 1 [Source:HGNC Symbol;Acc:HGNC:37245]	-1.3089877
ZGPAT	zinc finger CCCH-type and G-patch domain containing [Source:HGNC Symbol;Acc:HGNC:15948]	-1.220549
ZHX3	zinc fingers and homeoboxes 3 [Source:HGNC Symbol;Acc:HGNC:15935]	1.1633196
ZKSCAN5	zinc finger with KRAB and SCAN domains 5 [Source:HGNC Symbol;Acc:HGNC:12867]	-1.1033795
ZMIZ2	zinc finger MIZ-type containing 2 [Source:HGNC Symbol;Acc:HGNC:22229]	-1.293919
ZMPSTE24	zinc metallopeptidase STE24 [Source:HGNC Symbol;Acc:HGNC:12877]	1.3261002
ZMYM3	zinc finger MYM-type containing 3 [Source:HGNC Symbol;Acc:HGNC:13054]	1.3185234
ZMYND8	zinc finger MYND-type containing 8 [Source:HGNC Symbol;Acc:HGNC:9397]	1.497675
ZNF117	zinc finger protein 117 [Source:HGNC Symbol;Acc:HGNC:12897]	1.2713103
ZNF167	zinc finger with KRAB and SCAN domains 7	1.1501613
ZNF17	zinc finger protein 17 [Source:HGNC Symbol;Acc:HGNC:12958]	1.0940615
ZNF175	zinc finger protein 175 [Source:HGNC Symbol;Acc:HGNC:12964]	1.4543692
ZNF177	zinc finger protein 177 [Source:HGNC Symbol;Acc:HGNC:12966]	1.1073717
ZNF18	zinc finger protein 18 [Source:HGNC Symbol;Acc:HGNC:12969]	1.2079582
ZNF182	zinc finger protein 182 [Source:HGNC Symbol;Acc:HGNC:13001]	1.294628
ZNF187	zinc finger and SCAN domain containing 26	1.1880366
ZNF2	zinc finger protein 2 [Source:HGNC Symbol;Acc:HGNC:12991]	-1.1179156
ZNF211	zinc finger protein 211 [Source:HGNC Symbol;Acc:HGNC:13003]	1.1110598
ZNF212	zinc finger protein 212 [Source:HGNC Symbol;Acc:HGNC:13004]	-1.1992812
ZNF226	zinc finger protein 226 [Source:HGNC Symbol;Acc:HGNC:13019]	-1.197759
ZNF227	zinc finger protein 227 [Source:HGNC Symbol;Acc:HGNC:13020]	1.1459317
ZNF229	zinc finger protein 229 [Source:HGNC Symbol;Acc:HGNC:13022]	1.073163
ZNF236	zinc finger protein 236 [Source:HGNC Symbol;Acc:HGNC:13028]	-1.1066201
ZNF239	zinc finger protein 239 [Source:HGNC Symbol;Acc:HGNC:13031]	1.1393025
ZNF253	zinc finger protein 253 [Source:HGNC Symbol;Acc:HGNC:13497]	1.0887128
ZNF254	zinc finger protein 254 [Source:HGNC Symbol;Acc:HGNC:13047]	1.089705
ZNF257	zinc finger protein 257 [Source:HGNC Symbol;Acc:HGNC:13498]	1.0890805
ZNF259	ZPR1 zinc finger	-1.0642402
ZNF273	zinc finger protein 273 [Source:HGNC Symbol;Acc:HGNC:13067]	1.1399068
ZNF274	zinc finger protein 274 [Source:HGNC Symbol;Acc:HGNC:13068]	-1.0999728
ZNF275	zinc finger protein 275 [Source:HGNC Symbol;Acc:HGNC:13069]	-1.2076259
ZNF276	zinc finger protein 276 [Source:HGNC Symbol;Acc:HGNC:23330]	-1.0509992
ZNF28	zinc finger protein 28 [Source:HGNC Symbol;Acc:HGNC:13073]	-1.1353784
ZNF280C	zinc finger protein 280C [Source:HGNC Symbol;Acc:HGNC:25955]	1.2936234
ZNF280D	zinc finger protein 280D [Source:HGNC Symbol;Acc:HGNC:25953]	1.1883162
ZNF3	zinc finger protein 3 [Source:HGNC Symbol;Acc:HGNC:13089]	-1.08778295
ZNF30	zinc finger protein 30 [Source:HGNC Symbol;Acc:HGNC:13090]	1.5336546
ZNF317	zinc finger protein 317 [Source:HGNC Symbol;Acc:HGNC:13507]	-1.1925534
ZNF32	zinc finger protein 32 [Source:HGNC Symbol;Acc:HGNC:13095]	1.1484997
ZNF322A	zinc finger protein 322	1.2514639
ZNF323	zinc finger and SCAN domain containing 31	1.1024401
ZNF334	zinc finger protein 334 [Source:HGNC Symbol;Acc:HGNC:15806]	1.0934173
ZNF347	zinc finger protein 347 [Source:HGNC Symbol;Acc:HGNC:16447]	1.1871732
ZNF362	zinc finger protein 362 [Source:HGNC Symbol;Acc:HGNC:18079]	1.2028642
ZNF365	zinc finger protein 365 [Source:HGNC Symbol;Acc:HGNC:18194]	1.0663108
ZNF366	zinc finger protein 366 [Source:HGNC Symbol;Acc:HGNC:18316]	1.210234
ZNF37BP	zinc finger protein 37B, pseudogene [Source:HGNC Symbol;Acc:HGNC:13103]	1.2542553
ZNF385C	zinc finger protein 385C [Source:HGNC Symbol;Acc:HGNC:33722]	-1.1461304
ZNF385D	zinc finger protein 385D [Source:HGNC Symbol;Acc:HGNC:26191]	-1.2046355
ZNF391	zinc finger protein 391 [Source:HGNC Symbol;Acc:HGNC:18779]	-1.1383842
ZNF394	zinc finger protein 394 [Source:HGNC Symbol;Acc:HGNC:18832]	-1.168117
ZNF395	zinc finger protein 395 [Source:HGNC Symbol;Acc:HGNC:18737]	1.9529835
ZNF396	zinc finger protein 396 [Source:HGNC Symbol;Acc:HGNC:18824]	-1.1696907
ZNF397	zinc finger protein 397 [Source:HGNC Symbol;Acc:HGNC:18818]	1.0773748
ZNF416	zinc finger protein 416 [Source:HGNC Symbol;Acc:HGNC:20645]	-1.0952274
ZNF419	zinc finger protein 419 [Source:HGNC Symbol;Acc:HGNC:20648]	1.1647815
ZNF420	zinc finger protein 420 [Source:HGNC Symbol;Acc:HGNC:20649]	1.1686157
ZNF43	zinc finger protein 43 [Source:HGNC Symbol;Acc:HGNC:13109]	1.134512
ZNF436	zinc finger protein 436 [Source:HGNC Symbol;Acc:HGNC:20814]	-1.1847205
ZNF454	zinc finger protein 454 [Source:HGNC Symbol;Acc:HGNC:21200]	1.1095998
ZNF461	zinc finger protein 461 [Source:HGNC Symbol;Acc:HGNC:21629]	1.0850588
ZNF469	zinc finger protein 469 [Source:HGNC Symbol;Acc:HGNC:23216]	-1.4216908
ZNF471	zinc finger protein 471 [Source:HGNC Symbol;Acc:HGNC:23226]	1.0641251
ZNF474	zinc finger protein 474 [Source:HGNC Symbol;Acc:HGNC:23245]	1.0878664
ZNF492	zinc finger protein 492 [Source:HGNC Symbol;Acc:HGNC:23707]	1.2333165
ZNF501	zinc finger protein 501 [Source:HGNC Symbol;Acc:HGNC:23717]	-1.0514839
ZNF503	zinc finger protein 503 [Source:HGNC Symbol;Acc:HGNC:23589]	1.64230455
ZNF512B	zinc finger protein 512B [Source:HGNC Symbol;Acc:HGNC:29212]	1.297833
ZNF516	zinc finger protein 516 [Source:HGNC Symbol;Acc:HGNC:28990]	-1.1448478
ZNF521	zinc finger protein 521 [Source:HGNC Symbol;Acc:HGNC:24605]	1.52741565
ZNF525	zinc finger protein 525 [Source:HGNC Symbol;Acc:HGNC:29423]	1.129231
ZNF528	zinc finger protein 528 [Source:HGNC Symbol;Acc:HGNC:29384]	1.2033653
ZNF540	zinc finger protein 540 [Source:HGNC Symbol;Acc:HGNC:25331]	1.0857469
ZNF541	zinc finger protein 541 [Source:HGNC Symbol;Acc:HGNC:25294]	-1.2774128
ZNF557	zinc finger protein 557 [Source:HGNC Symbol;Acc:HGNC:28632]	-1.1254169
ZNF561	zinc finger protein 561 [Source:HGNC Symbol;Acc:HGNC:28684]	1.1797386
ZNF562	zinc finger protein 562 [Source:HGNC Symbol;Acc:HGNC:25950]	1.1203179
ZNF564	zinc finger protein 564 [Source:HGNC Symbol;Acc:HGNC:31106]	1.1938845
ZNF568	zinc finger protein 568 [Source:HGNC Symbol;Acc:HGNC:25392]	1.1080962
ZNF569	zinc finger protein 569 [Source:HGNC Symbol;Acc:HGNC:24737]	1.1405696
ZNF57	zinc finger protein 57 [Source:HGNC Symbol;Acc:HGNC:13125]	1.2452599
ZNF570	zinc finger protein 570 [Source:HGNC Symbol;Acc:HGNC:26416]	-1.2491958
ZNF572	zinc finger protein 572 [Source:HGNC Symbol;Acc:HGNC:26758]	1.16986
ZNF575	zinc finger protein 575 [Source:HGNC Symbol;Acc:HGNC:27606]	1.2544311
ZNF594	zinc finger protein 594 [Source:HGNC Symbol;Acc:HGNC:29392]	1.1630619
ZNF597	zinc finger protein 597 [Source:HGNC Symbol;Acc:HGNC:26573]	-1.1273812

ZNF598	zinc finger protein 598 [Source:HGNC Symbol;Acc:HGNC:28079]	1.081171
ZNF599	zinc finger protein 599 [Source:HGNC Symbol;Acc:HGNC:26408]	1.095206
ZNF613	zinc finger protein 613 [Source:HGNC Symbol;Acc:HGNC:25827]	1.1972141
ZNF630	zinc finger protein 630 [Source:HGNC Symbol;Acc:HGNC:28855]	1.0816106
ZNF641	zinc finger protein 641 [Source:HGNC Symbol;Acc:HGNC:31834]	-1.46716195
ZNF642	ZFP69 zinc finger protein	1.1753061
ZNF646	zinc finger protein 646 [Source:HGNC Symbol;Acc:HGNC:29004]	1.0626562
ZNF658	zinc finger protein 658 [Source:HGNC Symbol;Acc:HGNC:25226]	1.2106973
ZNF660	zinc finger protein 660 [Source:HGNC Symbol;Acc:HGNC:26720]	1.077062
ZNF664	zinc finger protein 664	-1.9485675
ZNF665	zinc finger protein 665 [Source:HGNC Symbol;Acc:HGNC:25885]	-1.1918615
ZNF670	zinc finger protein 670 [Source:HGNC Symbol;Acc:HGNC:28167]	-1.2311835
ZNF680	zinc finger protein 680 [Source:HGNC Symbol;Acc:HGNC:26897]	-1.0838039
ZNF69	zinc finger protein 69 [Source:HGNC Symbol;Acc:HGNC:13138]	1.1241014
ZNF692	zinc finger protein 692 [Source:HGNC Symbol;Acc:HGNC:26049]	1.360567
ZNF696	zinc finger protein 696 [Source:HGNC Symbol;Acc:HGNC:25872]	-1.1619598
ZNF697	zinc finger protein 697 [Source:HGNC Symbol;Acc:HGNC:32034]	-1.7919658
ZNF701	zinc finger protein 701 [Source:HGNC Symbol;Acc:HGNC:25597]	1.0940293
ZNF703	zinc finger protein 703 [Source:HGNC Symbol;Acc:HGNC:25883]	1.6431783
ZNF705A	zinc finger protein 705A [Source:HGNC Symbol;Acc:HGNC:32281]	1.1490581
ZNF708	zinc finger protein 708 [Source:HGNC Symbol;Acc:HGNC:12945]	1.1125325
ZNF71	zinc finger protein 71 [Source:HGNC Symbol;Acc:HGNC:13141]	-2.4892387
ZNF718	zinc finger protein 718 [Source:HGNC Symbol;Acc:HGNC:26889]	1.155425
ZNF746	zinc finger protein 746 [Source:HGNC Symbol;Acc:HGNC:21948]	1.1012286
ZNF766	zinc finger protein 766 [Source:HGNC Symbol;Acc:HGNC:28063]	1.2957864
ZNF767	zinc finger family member 767, pseudogene	-1.1369865
ZNF772	zinc finger protein 772 [Source:HGNC Symbol;Acc:HGNC:33106]	-1.0790421
ZNF773	zinc finger protein 773 [Source:HGNC Symbol;Acc:HGNC:30487]	-1.0893824
ZNF780B	zinc finger protein 780B [Source:HGNC Symbol;Acc:HGNC:33109]	1.1300204
ZNF782	zinc finger protein 782 [Source:HGNC Symbol;Acc:HGNC:33110]	1.061037
ZNF790	zinc finger protein 790 [Source:HGNC Symbol;Acc:HGNC:33114]	1.1636827
ZNF791	zinc finger protein 791 [Source:HGNC Symbol;Acc:HGNC:26895]	-1.0760626
ZNF792	zinc finger protein 792 [Source:HGNC Symbol;Acc:HGNC:24751]	1.24532705
ZNF8	zinc finger protein 8 [Source:HGNC Symbol;Acc:HGNC:13154]	-1.137526
ZNF813	zinc finger protein 813 [Source:HGNC Symbol;Acc:HGNC:33257]	-1.0545633
ZNF827	zinc finger protein 827 [Source:HGNC Symbol;Acc:HGNC:27193]	-1.1240923
ZNF830	zinc finger protein 830 [Source:HGNC Symbol;Acc:HGNC:28291]	-1.1392071
ZNF835	zinc finger protein 835 [Source:HGNC Symbol;Acc:HGNC:34332]	-1.1577439
ZNF836	zinc finger protein 836 [Source:HGNC Symbol;Acc:HGNC:34333]	1.0537889
ZNF839	zinc finger protein 839 [Source:HGNC Symbol;Acc:HGNC:20345]	1.0736257
ZNF847P	zinc finger protein 847, pseudogene [Source:HGNC Symbol;Acc:HGNC:34384]	-1.1288737
ZNFX1	zinc finger NFX1-type containing 1 [Source:HGNC Symbol;Acc:HGNC:29271]	-1.55281055
ZNHIT3	zinc finger HIT-type containing 3 [Source:HGNC Symbol;Acc:HGNC:12309]	1.1586044
ZNRF1	zinc and ring finger 1, E3 ubiquitin protein ligase [Source:HGNC Symbol;Acc:HGNC:18452]	-1.2286729
ZNRF2	zinc and ring finger 2, E3 ubiquitin protein ligase [Source:HGNC Symbol;Acc:HGNC:22316]	1.0593784
ZRANB1	zinc finger RANBP2-type containing 1 [Source:HGNC Symbol;Acc:HGNC:18224]	1.1078862
ZRANB2	zinc finger RANBP2-type containing 2 [Source:HGNC Symbol;Acc:HGNC:13058]	1.5242678
ZRSR2	c finger CCCH-type, RNA binding motif and serine/arginine rich 2 [Source:HGNC Symbol;Acc:HGNC:230	-1.1459846
ZSCAN1	zinc finger and SCAN domain containing 1 [Source:HGNC Symbol;Acc:HGNC:23712]	-1.1390978
ZSCAN2	zinc finger and SCAN domain containing 2 [Source:HGNC Symbol;Acc:HGNC:20994]	-1.1851785
ZSCAN22	zinc finger and SCAN domain containing 22 [Source:HGNC Symbol;Acc:HGNC:4929]	-1.1985495
ZSCAN23	zinc finger and SCAN domain containing 23 [Source:HGNC Symbol;Acc:HGNC:21193]	1.0932422
ZSCAN5A	zinc finger and SCAN domain containing 5A [Source:HGNC Symbol;Acc:HGNC:23710]	-1.1876063
ZSCAN5D	zinc finger and SCAN domain containing 5D [Source:HGNC Symbol;Acc:HGNC:37706]	-1.0571538
ZSWIM4	zinc finger SWIM-type containing 4 [Source:HGNC Symbol;Acc:HGNC:25704]	-3.5101073
ZSWIM6	zinc finger SWIM-type containing 6 [Source:HGNC Symbol;Acc:HGNC:29316]	1.5237813
ZWINT	ZW10 interacting kinetochore protein [Source:HGNC Symbol;Acc:HGNC:13195]	1.0930071
ZYG11A	zyg-11 family member A, cell cycle regulator [Source:HGNC Symbol;Acc:HGNC:32058]	1.0674232
ZYG11B	zyg-11 family member B, cell cycle regulator [Source:HGNC Symbol;Acc:HGNC:25820]	1.3063971
ZYX	zyxin [Source:HGNC Symbol;Acc:HGNC:13200]	1.231796

Supplemental table S3: Genes differentially expressed by mixture of flavanol metabolites

Gene symbol	Gene name	Fold-change
PKIG	protein kinase (cAMP-dependent, catalytic) inhibitor gamma	-1.2039772
MEGF8	multiple EGF-like-domains 8	-1.1590909
TACC3	transforming, acidic coiled-coil containing protein 3	-1.1565661
MGAT1	mannosyl (alpha-1,3-)-glycoprotein beta-1,2-N-acetylglucosaminyltransferase	-1.1540344
EIF3C	eukaryotic translation initiation factor 3, subunit C	-1.140174
EXOC7	exocyst complex component 7	-1.1356474
WDR6	WD repeat domain 6	-1.1273559
HDGF	hepatoma-derived growth factor	-1.1193913
TOX2	TOX high mobility group box family member 2	-1.114275
EEF2	eukaryotic translation elongation factor 2	-1.1134382
PTTG1IP	pituitary tumor-transforming 1 interacting protein	-1.1110272
KANSL3	KAT8 regulatory NSL complex subunit 3	-1.1076162
TCF25	transcription factor 25 (basic helix-loop-helix)	-1.107239
TLE1	transducin-like enhancer of split 1 (E(sp1) homolog, Drosophila)	-1.106483
CBX2	chromobox homolog 2	-1.1029116
COL27A1	collagen, type XXVII, alpha 1	-1.1007673
VWCE	von Willebrand factor C and EGF domains	-1.0966772
DBF4B	DBF4 zinc finger B	-1.0953798
CCR9	chemokine (C-C motif) receptor 9	-1.0949962
ADAMTSL1	ADAMTS-like 1	-1.0930561
ST8SIA3	ST8 alpha-N-acetyl-neuraminide alpha-2,8-sialyltransferase 3	-1.0924023
HIP1	huntingtin interacting protein 1	-1.0882293
PRMT1	protein arginine methyltransferase 1	-1.0881253
PBDC1	polysaccharide biosynthesis domain containing 1	-1.0875945
SLC26A1	solute carrier family 26 (anion exchanger), member 1	-1.0874616
ADARB1	adenosine deaminase, RNA-specific, B1	-1.0856616
AGO3	argonaute 3, RISC catalytic component	-1.085366
NME2	NME/NM23 nucleoside diphosphate kinase 2	-1.0847706
SNRPB	small nuclear ribonucleoprotein polypeptides B and B1	-1.0840416
COL26A1	collagen type XXVI alpha 1 chain	-1.0809991
FDXR	ferredoxin reductase	-1.0809367
TUBA1C	tubulin, alpha 1c	-1.0806866
IP6K3	inositol hexakisphosphate kinase 3	-1.0805348
NCAPD2	non-SMC condensin I complex, subunit D2	-1.0796225
LRG_105	serpin peptidase inhibitor, clade G (C1 inhibitor), member 1	-1.079115
GJB4	gap junction protein, beta 4, 30.3kDa	-1.078994
RHOC	ras homolog family member C	-1.07842
ARHGAP27	Rho GTPase activating protein 27	-1.0766033
BLK	BLK proto-oncogene, Src family tyrosine kinase	-1.0765917
FAM187B	family with sequence similarity 187, member B	-1.0760257
FAM150B	family with sequence similarity 150, member B	-1.0758936
HIRA	histone cell cycle regulator	-1.0755848
HM13	histocompatibility (minor) 13	-1.0755416
PIGL	phosphatidylinositol glycan anchor biosynthesis, class L	-1.0751199
CADM4	cell adhesion molecule 4	-1.0731615
PABPN1L	poly(A) binding protein, nuclear 1-like (cytoplasmic)	-1.0730269
CERK	ceramide kinase	-1.072039
DEFA4	defensin, alpha 4, corticostatin	-1.0709122
CAPNS1	calpain, small subunit 1	-1.0708506
PHLPP2	PH domain and leucine rich repeat protein phosphatase 2	-1.0708393
GDPD4	glycerophosphodiester phosphodiesterase domain containing 4	-1.0702958
TMEM26	transmembrane protein 26	-1.0695992
RAPGEF3	Rap guanine nucleotide exchange factor (GEF) 3	-1.0691727
CEP250	centrosomal protein 250kDa	-1.0681767
TMEM44	transmembrane protein 44	-1.06778215
LRG_669	protein kinase C, gamma	-1.0676694
RGS4	regulator of G-protein signaling 4	-1.0674753
MN1	meningioma (disrupted in balanced translocation) 1	-1.0669563
RTN3	reticulon 3	-1.0665742
MMP25	matrix metallopeptidase 25	-1.0661955
DTHD1	death domain containing 1	-1.0660564
FAM71B	family with sequence similarity 71, member B	-1.0654712
CELSR2	cadherin, EGF LAG seven-pass G-type receptor 2	-1.0653684
EMG1	EMG1 N1-specific pseudouridine methyltransferase	-1.065288
TTC28	tetratricopeptide repeat domain 28	-1.0643384
JAKMIP3	Janus kinase and microtubule interacting protein 3	-1.0639915
LOC254057	uncharacterized LOC254057	-1.063947
GDAP1L1	ganglioside induced differentiation associated protein 1-like 1	-1.0631312

T	T, brachyury homolog	-1.0625863
HMG20B	high mobility group 20B	-1.0624989
TMPRSS11B	transmembrane protease, serine 11B	-1.0623791
MTCH1	mitochondrial carrier 1	-1.0619674
LHX9	LIM homeobox 9	-1.0619392
PIDD1	p53-induced death domain protein 1	-1.0614755
CBX6	chromobox homolog 6	-1.0612999
LOC647983	uncharacterized LOC647983	-1.0612423
PPIL2	peptidylprolyl isomerase (cyclophilin)-like 2	-1.0612197
TLE4	transducin-like enhancer of split 4	-1.060471
COLGALT1	collagen beta(1-O)galactosyltransferase 1	-1.0604488
PHLDB3	pleckstrin homology-like domain, family B, member 3	-1.060153
SYNDIG1	synapse differentiation inducing 1	-1.0595171
OR56B1	olfactory receptor, family 56, subfamily B, member 1	-1.0592353
ZNF781	zinc finger protein 781	-1.0592027
FAM157A	family with sequence similarity 157, member A	-1.0589517
PHC2	polyhomeotic homolog 2	-1.0589206
PSAP	prosaposin	-1.0588593
LINC00028	long intergenic non-protein coding RNA 28	-1.0588303
FRS3	fibroblast growth factor receptor substrate 3	-1.0587405
CDC20B	cell division cycle 20B	-1.0580913
OLFM1	olfactomedin 1	-1.0579987
SORCS1	sortilin-related VPS10 domain containing receptor 1	-1.0578002
PPM1G	protein phosphatase, Mg2+/Mn2+ dependent, 1G	-1.0577452
C5AR2	complement component 5a receptor 2	-1.0574327
REM1	RAS (RAD and GEM)-like GTP-binding 1	-1.0573826
TMEM236	transmembrane protein 236	-1.0570358
C3orf33	chromosome 3 open reading frame 33	-1.0569599
C5orf56	chromosome 5 open reading frame 56	-1.0569204
ARHGEF35	Rho guanine nucleotide exchange factor (GEF) 35	-1.0566783
EMX2OS	EMX2 opposite strand/antisense RNA	-1.056536
EXOC3L4	exocyst complex component 3-like 4	-1.0563222
GPR6	G protein-coupled receptor 6	-1.0563198
DTX2P1	DTX2 pseudogene 1	-1.0559713
ZFR2	zinc finger RNA binding protein 2	-1.055905
LGALS16	lectin, galactoside-binding, soluble, 16	-1.0553133
P2RX1	purinergic receptor P2X, ligand gated ion channel, 1	-1.0552719
CDH5	cadherin 5, type 2 (vascular endothelium)	-1.0550908
HIPK2	homeodomain interacting protein kinase 2	-1.0549743
MYBPC2	myosin binding protein C, fast type	-1.0549695
NCAM1	neural cell adhesion molecule 1	-1.0546328
PREX1	phosphatidylinositol-3,4,5-trisphosphate-dependent Rac exchange factor 1	-1.0545554
SCARNA6	small Cajal body-specific RNA 6	-1.054547
IL23R	interleukin 23 receptor	-1.0544063
GSTO1	glutathione S-transferase omega 1	-1.0542344
ZDHHC14	zinc finger, DHHC-type containing 14	-1.053888
OR51A7	olfactory receptor, family 51, subfamily A, member 7	-1.0536222
GLI2	GLI family zinc finger 2	-1.053356
NCR1	natural cytotoxicity triggering receptor 1	-1.0533184
MAP2	microtubule-associated protein 2	-1.0531723
HOXD4	homeobox D4	-1.0530685
CDRT7	CMT1A duplicated region transcript 7 (non-protein coding)	-1.0529283
SEMG2	semenogelin II	-1.0528218
PRKCG	protein kinase C, gamma	-1.0528005
AFF2	AF4/FMR2 family, member 2	-1.0527471
FTCD	formimidoyltransferase cyclodeaminase	-1.0527433
SERPING1	serpin peptidase inhibitor, clade G (C1 inhibitor), member 1	-1.0526729
PSMD2	proteasome (prosome, macropain) 26S subunit, non-ATPase, 2	-1.0525974
ZMIZ1-AS1	ZMIZ1 antisense RNA 1	-1.0517846
LENEP	lens epithelial protein	-1.0517696
PCNXL2	pecanex-like 2 (Drosophila)	-1.0517497
NPHP3-AS1	NPHP3 antisense RNA 1	-1.0516611
MAGEB16	melanoma antigen family B16	-1.0516213
OR2T10	olfactory receptor, family 2, subfamily T, member 10	-1.0515475
MORC2-AS1	MORC2 antisense RNA 1	-1.0513216
TRIQK	triple QxxK/R motif containing	-1.0512347
RBP7	retinol binding protein 7, cellular	-1.051118
ZDHHC8	zinc finger, DHHC-type containing 8	-1.0511082
ANKRD65	ankyrin repeat domain 65	-1.0510427
RASGRP3	RAS guanyl releasing protein 3 (calcium and DAG-regulated)	-1.0505981
LOC728073	uncharacterized LOC728073	-1.0505474

INF2	inverted formin, FH2 and WH2 domain containing	-1.050537
CNTFR-AS1	CNTFR antisense RNA 1	-1.0504599
RUFY1	RUN and FYVE domain containing 1	1.0502021
SOX10	SRY (sex determining region Y)-box 10	1.0502113
LMBR1	limb development membrane protein 1	1.0503908
AGTR1	angiotensin II receptor, type 1	1.0504216
LRG_47	complement factor H	1.0505482
UBA5	ubiquitin-like modifier activating enzyme 5	1.050722
SELV	selenoprotein V	1.0509702
L2HGDH	L-2-hydroxyglutarate dehydrogenase	1.0511317
SIAH2	siah E3 ubiquitin protein ligase 2	1.0512342
IGSF3	immunoglobulin superfamily, member 3	1.0512369
LRG_271	SRY (sex determining region Y)-box 10	1.0512419
SSX8	synovial sarcoma, X breakpoint 8	1.0512534
CLRN3	clarin 3	1.0513512
TMEM229B	transmembrane protein 229B	1.0515218
TXNDC5	thioredoxin domain containing 5 (endoplasmic reticulum)	1.0516245
LRP11	low density lipoprotein receptor-related protein 11	1.0524752
AS3MT	arsenite methyltransferase	1.0525384
CCDC43	coiled-coil domain containing 43	1.052739
FLJ22447	uncharacterized LOC400221	1.0529289
SLC34A2	solute carrier family 34 (type II sodium/phosphate cotransporter), member 2	1.0529337
C3orf33	chromosome 3 open reading frame 33	1.0529912
AP1S2	adaptor-related protein complex 1, sigma 2 subunit	1.0530529
PREX2	phosphatidylinositol-3,4,5-trisphosphate-dependent Rac exchange factor 2	1.0540205
OVGP1	oviductal glycoprotein 1, 120kDa	1.0540551
TP53AIP1	tumor protein p53 regulated apoptosis inducing protein 1	1.0542524
ZEB2	zinc finger E-box binding homeobox 2	1.0542524
SLC44A1	solute carrier family 44 (choline transporter), member 1	1.0544792
ZNF235	zinc finger protein 235	1.0547124
GJC3	gap junction protein, gamma 3, 30.2kDa	1.054886
DIRAS1	DIRAS family, GTP-binding RAS-like 1	1.0549461
MATN3	matrilin 3	1.0552053
ADCY10	adenylate cyclase 10 (soluble)	1.0560186
ZMYND11	zinc finger, MYND-type containing 11	1.0565411
TRPC3	transient receptor potential cation channel, subfamily C, member 3	1.0565642
PTPRT	protein tyrosine phosphatase, receptor type, T	1.0569792
OR2M2	olfactory receptor, family 2, subfamily M, member 2	1.0570612
GRK4	G protein-coupled receptor kinase 4	1.057197
RXFP4	relaxin/insulin-like family peptide receptor 4	1.0578506
LRG_754	son of sevenless homolog 1	1.0581787
IQCG	IQ motif containing G	1.0596243
CCDC170	coiled-coil domain containing 170	1.0603614
ADGRA1A	adhesion G protein-coupled receptor A1	1.0605998
ST7L	suppression of tumorigenicity 7 like	1.0606669
IKZF5	IKAROS family zinc finger 5	1.0610611
LCN10	lipocalin 10	1.0628414
INMT	indolethylamine N-methyltransferase	1.0630426
PLCD4	phospholipase C, delta 4	1.0641334
CDON	cell adhesion associated, oncogene regulated	1.0641468
PPP1R12B	protein phosphatase 1, regulatory subunit 12B	1.0644135
APOL6	apolipoprotein L, 6	1.0648336
SEC23B	Sec23 homolog B (S. cerevisiae)	1.0651723
EXOSC3	exosome component 3	1.0654143
LOC257396	uncharacterized LOC257396	1.0658379
FNBP1	formin binding protein 1	1.0667115
MOB1A	MOB kinase activator 1B	1.0672476
RRN3P2	RNA polymerase I transcription factor homolog (S. cerevisiae) pseudogene 2	1.0685875
SPDYE8P	speedy/RINGO cell cycle regulator family member E8, pseudogene	1.0697902
CDX1	caudal type homeobox 1	1.0699176
GALK2	galactokinase 2	1.070527
LINC00051	long intergenic non-protein coding RNA 51	1.0709138
OS9	osteosarcoma amplified 9, endoplasmic reticulum lectin	1.071141
SLC35A1	solute carrier family 35 (CMP-sialic acid transporter), member A1	1.0716475
XBP1	X-box binding protein 1	1.0724787
DEDD	death effector domain containing	1.0731583
KCNAB1	potassium channel, voltage gated subfamily A regulatory beta subunit 1	1.073209
ATG4C	autophagy related 4C, cysteine peptidase	1.0734856
GNRHR	gonadotropin-releasing hormone receptor	1.0735542
TNIP3	TNFAIP3 interacting protein 3	1.074055
TAB3	TGF-beta activated kinase 1/MAP3K7 binding protein 3	1.0742283

CTAGE1	cutaneous T-cell lymphoma-associated antigen 1	1.0762799
ZNF701	zinc finger protein 701	1.0768218
INSIG2	insulin induced gene 2	1.0777742
CFH	complement factor H	1.0777941
PDE1C	phosphodiesterase 1C, calmodulin-dependent 70kDa	1.0783639
ZNF253	zinc finger protein 253	1.0792263
ABCC5	ATP-binding cassette, sub-family C (CFTR/MRP), member 5	1.0793691
PPY2P	pancreatic polypeptide 2, pseudogene	1.0809271
THNSL1	threonine synthase-like 1 (S. cerevisiae)	1.0811859
CCDC174	coiled-coil domain containing 174	1.0819297
PLAU	plasminogen activator, urokinase	1.0830336
CYB5R4	cytochrome b5 reductase 4	1.0843542
SNRNP48	small nuclear ribonucleoprotein 48kDa (U11/U12)	1.0867411
CD274	CD274 molecule	1.0878695
DUS4L	dihydrouridine synthase 4-like (S. cerevisiae)	1.0881691
RSU1	Ras suppressor protein 1	1.0903277
SGMS2	sphingomyelin synthase 2	1.0916607
ARHGAP42	Rho GTPase activating protein 42	1.0928626
ZNF136	zinc finger protein 136	1.0934138
SOS1	son of sevenless homolog 1 (Drosophila)	1.0946532
TLK1	tousled-like kinase 1	1.094888
MLLT3	myeloid/lymphoid or mixed-lineage leukemia	1.0973772
ZNF542	zinc finger protein 542	1.0977765
NEIL3	nei endonuclease VIII-like 3	1.0986665
LAMP3	lysosomal-associated membrane protein 3	1.100084
NRBF2	nuclear receptor binding factor 2	1.1025711
METTL3	methyltransferase like 3	1.103495
CTAGE4	CTAGE family, member 4	1.1087644
ATF2	activating transcription factor 2	1.1094769
TMEM87B	transmembrane protein 87B	1.1123282
PAM	peptidylglycine alpha-amidating monooxygenase	1.1157597
SRGAP2	SLIT-ROBO Rho GTPase activating protein 2	1.11683
CLDN11	claudin 11	1.1180185
CDKL1	cyclin-dependent kinase-like 1 (CDC2-related kinase)	1.1208974
C5orf56	chromosome 5 open reading frame 56	1.1229732
ABHD3	abhydrolase domain containing 3	1.1268681
CDKN2B-AS1	CDKN2B antisense RNA 1	1.1276822
CYP20A1	cytochrome P450, family 20, subfamily A, polypeptide 1	1.1291639
TMEM68	transmembrane protein 68	1.1453892
LAMC2	laminin, gamma 2	1.1746995
TUBE1	tubulin, epsilon 1	1.1888752
IFRD1	interferon-related developmental regulator 1	1.2011763
HERPUD1	homocysteine-inducible, endoplasmic reticulum stress-inducible, ubiquitin-like domain member 1	1.2108529

Supplemetal table S4: The binding scores expressed in Glide docking Scores (kcal/mol) and Induced fit docking (IFD) scores between biotransformed variants of epicatechin in p38 MAPK active site (Pdb id: 4F9Y model)

**Epicatechin metabolites**	**Glide docking scores**	**Residues involved in H-bonding**
4′-O-methyl(-)-epicatechin-7-β-D-glucuronide (4’MEC7G)	-10.242	Ser-32, Lys-53, His-107, Met-109, Ser-154, **Glu-178**
3′-O-methyl(-)-epicatechin (3’MEC)	-7.246	Met-109, Ala-111, **Glu-178**
(-)-epicatechin-4′-sulfate (EC4’S)	-7.822	Gly-36, Lys-53, His-107, Met-109

Supplemental table S5: Differentially expressed miRNA by TNF and by mixture of flavanol metabolites

**TNF/control**	
miRNA	***FC***
hsa-let-7f	1,0998023
hsa-miR-1246	1,2011293
hsa-miR-139-5p	1,1791074
hsa-miR-146a	2,0559123
hsa-miR-148a	1,5927936
hsa-miR-155	1,3901024
hsa-miR-195	1,2252228
hsa-miR-199a-3p	1,3397902
hsa-miR-199a-5p	1,3601944
hsa-miR-214	1,2171185
hsa-miR-221*	1,2703397
hsa-miR-29b-1*	1,6904817
hsa-miR-369-5p	1,2240878
hsa-miR-455-3p	1,2339121
hsa-miR-574-5p	1,1925792
hsa-miR-575	1,0906901
hsa-miR-584	1,2825359
hsa-miR-638	1,1496371
hsa-miR-98	1,1357678

**Mix**	
miRNA	***FC***
hsa-let-7a	-1,0882393
hsa-let-7f	-1,0843385
hsa-miR-10a	-1,1383361
hsa-miR-10b	-1,1306673
hsa-miR-1290	-1,1568191
hsa-miR-130b	-1,0858847
hsa-miR-134	-1,2618986
hsa-miR-181a*	-1,177034
hsa-miR-221*	-1,1774971
hsa-miR-224	-1,1127338
hsa-miR-30a*	-1,1222104
hsa-miR-30c	-1,1183422
hsa-miR-30e*	-1,1639618
hsa-miR-320a	-1,0500371
hsa-miR-320c	-1,0795624
hsa-miR-320d	-1,0846931
hsa-miR-361-5p	-1,1056353
hsa-miR-365	-1,1605713
hsa-miR-543	-1,1083959
hsa-miR-769-5p	1,2960966

Supplemental table S6: Epigenetic effect of flavanol mixture

Probe_ID	MeanBeta_TNF	MeanBeta_TNF+Mix	Delta_Beta	P.Value	UCSC_Ref Single Gene_Name	UCSC_RefGene_Name	UCSC_RefGene_Accession	UCSC_RefGene_Group	Relation_to_UCSC_CpG_Island
cg23837845	0,9438547	0,773139183	-0,170715517	2,03E-07	GOLGA8A	GOLGA8A	NR_027409	Body	
cg03330820	0,969528317	0,846250361	-0,123277956	3,58E-07	CACHD1	CACHD1	NM_020925	Body	
cg10685961	0,927738469	0,765691393	-0,162047076	7,70E-07	EPC1	EPC1	NM_025209	3UTR	
cg15047610	0,097278026	0,255693661	0,158415635	7,96E-07					S_Shelf
cg22045055	0,946082933	0,83574257	-0,110340364	8,22E-07	MPZL1	MPZL1;MPZL1;MPZL1	NM_001146191;NM_003953;NM_024569	Body;Body;Body	
cg07741992	0,967675467	0,826701822	-0,140973645	9,83E-07					
cg12797771	0,933620565	0,780730575	-0,15288999	1,01E-06	NME7	NME7;NME7	NM_013330;NM_197972	Body;Body	
cg23441030	0,946595781	0,755539541	-0,19105624	1,38E-06	RABGAP1L	RABGAP1L;RABGAP1L	NM_001035230;NM_014857	Body;Body	
cg09906169	0,933007617	0,783423603	-0,149584015	1,89E-06	AP2A2	AP2A2	NM_012305	Body	N_Shore
cg01861042	0,958245062	0,819079834	-0,139165228	2,07E-06					
cg18417772	0,951602675	0,841206166	-0,110396509	2,47E-06					N_Shore
cg07707687	0,975473953	0,844545487	-0,130928466	2,77E-06					N_Shelf
cg26489875	0,047387156	0,169472132	0,122084975	3,10E-06	MALAT1	MALAT1	NR_002819	Body	S_Shelf
cg15031299	0,953646394	0,844261145	-0,109385248	3,61E-06	NOTCH4	NOTCH4	NM_004557	Body	
cg24528604	0,073230662	0,209364515	0,136133852	3,74E-06	PICALM	PICALM;PICALM	NM_001008660;NM_007166	Body;Body	N_Shore
cg14299369	0,952908973	0,812634576	-0,140274397	3,75E-06	TGM5	TGM5;TGM5	NM_004245;NM_201631	TSS1500;TSS1500	
cg01145686	0,782356334	0,921168549	0,138812215	4,14E-06					N_Shore
cg15598650	0,904853849	0,748331815	-0,156522034	4,48E-06	FGFR2	FGFR2;FGFR2;FGFR2;FGFR2;FGFR2;FGFR2;FGFR2;FGFR2;FGFR2	NM_001144914;NM_001144915;NM_022970;NM_001144913;N	Body;Body;Body;Body;Body;B	
cg11918049	0,946600988	0,839472488	-0,1071285	4,59E-06	MAP4K4	MAP4K4;MAP4K4;MAP4K4	NM_145687;NM_004834;NM_145686	Body;Body;Body	
cg24121906	0,952801963	0,845371973	-0,107429991	4,69E-06	SLC35F1	SLC35F1	NM_001029858	Body	
cg18348578	0,935546127	0,819733787	-0,115812341	5,56E-06	AK2	AK2;AK2	NM_013411;NM_001625	Body;Body	N_Shelf
cg04987857	0,882089516	0,730077894	-0,152011622	5,76E-06	FRS2	FRS2;FRS2	NM_006654;NM_001042555	TSS1500;TSS1500	N_Shore
cg19392994	0,937581057	0,821778549	-0,115802508	5,87E-06	IQCH	IQCH;IQCH	NM_001031715;NM_022784	Body;Body	S_Shelf
cg16426510	0,932981513	0,777041356	-0,155940157	6,25E-06	PHF21A	PHF21A;PHF21A	NM_016621;NM_001101802	Body;Body	
cg20631969	0,033373974	0,14753296	0,114158985	6,45E-06	MKRN1	MKRN1;MKRN1	NM_001145125;NM_013446	Body;Body	Island
cg07512814	0,059235765	0,189498747	0,130262983	6,90E-06	LDLR	LDLR	NM_000527	Body	S_Shore
cg07714266	0,833448072	0,584649848	-0,248798224	8,62E-06	JAZF1	JAZF1	NM_175061	Body	
cg02455571	0,942120548	0,694542212	-0,247578336	8,85E-06	NOS1AP	NOS1AP;NOS1AP	NM_014697;NM_001164757	Body;Body	
cg02399415	0,942051642	0,831927941	-0,110123701	8,95E-06					
cg12255352	0,954853459	0,822294506	-0,132558952	9,18E-06	MIPEP	MIPEP	NM_005932	Body	
cg22791307	0,070391526	0,211085784	0,140694258	9,31E-06					
cg10115321	0,906684395	0,788924457	-0,117759937	1,11E-05	CSNK2B	CSNK2B	NM_001320	Body	S_Shore
cg12819873	0,805755426	0,908269214	0,102513788	1,11E-05	PRG2	PRG2	NM_002728	5UTR	
cg02186748	0,792179854	0,912450485	0,120270631	1,22E-05	COL1A1	COL1A1	NM_000088	TSS1500	S_Shore
cg16851127	0,925989746	0,683437393	-0,242552353	1,22E-05	RNF19A	RNF19A;RNF19A	NM_015435;NM_183419	5UTR;5UTR	
cg16754678	0,067097721	0,180111867	0,113014146	1,26E-05					Island
cg11361658	0,903909124	0,747699113	-0,156210011	1,27E-05					
cg16244394	0,932812565	0,750494046	-0,182318519	1,28E-05	CDC42SE2	CDC42SE2;CDC42SE2	NM_001038702;NM_020240	Body;Body	
cg05991929	0,92314612	0,812056354	-0,111089767	1,32E-05					
cg14574951	0,097124624	0,216814796	0,119690172	1,42E-05	ERC2	ERC2	NM_015576	Body	
cg14887500	0,896707877	0,756806991	-0,139900885	1,62E-05	PIWIL1	PIWIL1	NM_004764	Body	S_Shelf
cg13170640	0,7433373	0,87433789	0,131000591	1,65E-05					
cg21522636	0,100282712	0,234546573	0,134263861	1,73E-05	RASSF1	RASSF1;RASSF1;RASSF1;RASSF1	NM_170713;NM_170714;NM_007182;NM_170712	TSS200;Body;Body;5UTR	Island
cg27027668	0,847127994	0,690029168	-0,157098826	1,75E-05					
cg04495687	0,963437477	0,84315568	-0,120281797	1,77E-05	C16orf45	C16orf45	NM_033201	Body	S_Shelf
cg09052626	0,103324094	0,278491297	0,175167203	1,96E-05					S_Shore
cg19561453	0,943028837	0,715075044	-0,227953793	1,98E-05	HSF2	HSF2;HSF2	NM_001135564;NM_004506	Body;Body	S_Shelf
cg24872610	0,865409797	0,700469802	-0,164939995	1,99E-05	PFKP	PFKP	NM_002627	Body	N_Shore
cg01766943	0,759148065	0,86671778	0,107569715	1,99E-05	SPTBN1	SPTBN1;SPTBN1	NM_178313;NM_003128	Body;Body	
cg13088660	0,944501982	0,818091762	-0,12641022	2,04E-05	SPTBN1	SPTBN1	NM_003128	3UTR	
cg00063699	0,902078698	0,665874361	-0,236204337	2,05E-05	PTPN14	PTPN14	NM_005401	Body	
cg03160656	0,963870596	0,854115577	-0,109755019	2,07E-05					
cg18468725	0,949170535	0,785985571	-0,163184964	2,12E-05					
cg15498379	0,081299273	0,206345091	0,125045818	2,16E-05	ZSCAN18	ZSCAN18;ZSCAN18;ZSCAN18;ZSCAN18	NM_001145544;NM_001145542;NM_001145543;NM_023926	TSS1500;Body;TSS1500;TSS	Island
cg14636261	0,770123812	0,893935188	0,123811376	2,21E-05	ABO	ABO	NM_020469	Body	S_Shore
cg09890339	0,80574478	0,910669086	0,104924306	2,26E-05	CACNA1C	CACNA1C;CACNA1C;CACNA1C;CACNA1C;CACNA1C;CACNA1C;CA	NM_001129844;NM_001129827;NM_001129839;NM_00112983	Body;Body;Body;Body;Body;B	
cg03664459	0,78786913	0,901463785	0,113594655	2,36E-05	C2orf90	C2orf90	NM_001013662	TSS200	
cg03724962	0,952953629	0,84427022	-0,108683408	2,39E-05	TRIM2	TRIM2;TRIM2	NM_015271;NM_001130067	3UTR;3UTR	
cg09657431	0,941911027	0,778327736	-0,163583291	2,50E-05	CDC6	CDC6	NM_001254	Body	
cg07444152	0,925443202	0,768935156	-0,156508046	2,50E-05	EHMT2	EHMT2;EHMT2	NM_006709;NM_025256	Body;Body	S_Shelf
cg08385173	0,835712601	0,656006611	-0,17970599	2,60E-05	C17orf80	C17orf80;C17orf80;CPSF4L	NM_001100621;NM_017941;NM_001129885	3UTR;3UTR;Body	
cg22978042	0,132841399	0,265417197	0,132575798	2,65E-05	PSMC1	PSMC1	NM_002802	TSS1500	N_Shore
cg14016620	0,953641589	0,843142967	-0,110498623	2,66E-05	GNAQ	GNAQ	NM_002072	Body	
cg02177951	0,2382407	0,413838622	0,175597922	2,79E-05					Island
cg08218914	0,942831971	0,73710321	-0,205728761	2,79E-05	RGNEF	RGNEF	NM_001080479	3UTR	
cg07494499	0,112127849	0,251204451	0,139076602	2,89E-05	NXN	NXN	NM_022463	Body	
cg15441656	0,665873987	0,827836397	0,161962411	2,95E-05	CLDN23	CLDN23;CLDN23	NM_194284;NM_194284	1stExon;3UTR	S_Shore
cg12707907	0,95002855	0,748650694	-0,201377856	3,06E-05	SLITRK6	SLITRK6	NM_032229	5UTR	
cg21153839	0,946602386	0,825542976	-0,12105941	3,09E-05					N_Shelf
cg00059034	0,065727481	0,206458775	0,140731294	3,10E-05					S_Shelf
cg23065757	0,931214446	0,787780973	-0,143433472	3,10E-05					N_Shelf
cg25199552	0,913355356	0,813153838	-0,100201519	3,21E-05	KDM1A	KDM1A;KDM1A	NM_015013;NM_001009999	Body;Body	S_Shelf
cg17763467	0,779036338	0,881845978	0,10280964	3,23E-05	C6orf186	C6orf186	NM_001123364	Body	
cg26801047	0,080431304	0,202506163	0,122074858	3,25E-05	DLL3	DLL3;DLL3	NM_203486;NM_016941	TSS1500;TSS1500	N_Shore
cg03672203	0,0460714	0,163901291	0,117829892	3,29E-05					Island
cg05055760	0,047014014	0,193626336	0,146612322	3,31E-05					N_Shelf
cg07613023	0,946572328	0,814659993	-0,131912335	3,31E-05	BACH2	BACH2;BACH2	NM_021813;NM_001170794	5UTR;5UTR	
cg03553226	0,896458554	0,774972118	-0,121486435	3,34E-05					N_Shelf
cg18233746	0,130834041	0,300339286	0,169505245	3,52E-05	MGST1	MGST1;MGST1;MGST1	NM_145791;NM_020300;NM_145792	TSS1500;TSS200;5UTR	
cg00089314	0,75114847	0,857980695	0,106832225	3,56E-05	LOC153328	LOC153328	NM_145282	TSS1500	N_Shore
cg25592799	0,923720671	0,815691939	-0,108028732	3,59E-05	LY6G6F	LY6G6F	NM_001003693	TSS1500	S_Shelf
cg01576496	0,948725269	0,817188968	-0,131536301	3,72E-05	PPP1R12A	PPP1R12A;PPP1R12A;PPP1R12A	NM_001143885;NM_002480;NM_001143886	Body;Body;5UTR	
cg23620279	0,129308478	0,282902698	0,15359422	3,75E-05	RPS24	RPS24;RPS24;RPS24;RPS24;RPS24;RPS24	NM_001142285;NM_001142282;NM_001142284;NM_001026;N	TSS200;TSS200;TSS200;TSS	Island
cg14778914	0,957435426	0,811547736	-0,145887689	3,76E-05	ARSG	ARSG	NM_014960	5UTR	S_Shore
cg23828763	0,068746494	0,17062746	0,101880966	3,89E-05	TACC2	TACC2;TACC2	NM_206862;NM_206861	Body;Body	S_Shore
cg04072545	0,119417411	0,302533891	0,183116479	3,94E-05	INS-IGF2	INS-IGF2;IGF2AS;IGF2;IGF2;IGF2AS;IGF2	NR_003512;NR_028044;NM_001127598;NM_001007139;NR_02	Body;TSS1500;Body;5UTR;TS	Island
cg09360796	0,921687521	0,791579279	-0,130108242	3,97E-05	MAGI2	MAGI2	NM_012301	Body	
cg09682361	0,821687513	0,926407831	0,104720318	4,11E-05					
cg11230363	0,915625632	0,714372016	-0,201253616	4,11E-05					N_Shelf
cg10638599	0,069420951	0,182699158	0,113278208	4,11E-05	SHISA7	SHISA7	NM_001145176	TSS1500	Island
cg19099311	0,774900755	0,888350097	0,113449342	4,13E-05	PTPRN2	PTPRN2;PTPRN2;PTPRN2	NM_002847;NM_130842;NM_130843	Body;Body;Body	Island
cg03794862	0,750656338	0,861134495	0,110478157	4,15E-05	WNT2	WNT2;WNT2	NM_003391;NR_024047	Body;Body	N_Shore
cg09372664	0,924566966	0,788131969	-0,136434997	4,17E-05					S_Shelf
cg02950608	0,952353683	0,825003543	-0,127350141	4,20E-05					N_Shore
cg02182587	0,932459147	0,731972001	-0,200487145	4,27E-05					N_Shore
cg21043746	0,753221595	0,860621124	0,107399529	4,30E-05	ADAMTS2	ADAMTS2	NM_014244	Body	Island
cg26074575	0,814129835	0,589340194	-0,224789641	4,31E-05	FNDC3B	FNDC3B;FNDC3B	NM_022763;NM_001135095	5UTR;5UTR	
cg05721199	0,858164039	0,68939665	-0,168767389	4,36E-05	SAMD7	SAMD7	NM_182610	TSS1500	
cg00993881	0,951237519	0,812287384	-0,138950135	4,47E-05	DAAM1	DAAM1	NM_014992	5UTR	
cg09067351	0,93989228	0,831596782	-0,108295498	4,48E-05	BMPR1A	BMPR1A	NM_004329	Body	
cg24453438	0,822920255	0,926084578	0,103164323	4,68E-05					S_Shelf
cg12972064	0,102939004	0,220436291	0,117497287	4,69E-05	FNDC3B	FNDC3B;FNDC3B	NM_001135095;NM_022763	Body;Body	
cg06175912	0,968886404	0,863822477	-0,105063927	4,73E-05	MUC4	MUC4;MUC4;MUC4	NM_018406;NM_138297;NM_004532	Body;Body;Body	
cg06460672	0,841627589	0,954733895	0,113106306	4,74E-05	CES4	CES4	NR_003276	Body	S_Shelf
cg06227406	0,05019546	0,187578549	0,137383088	4,77E-05	PTPRB	PTPRB;PTPRB	NM_001109754;NM_002837	Body;Body	N_Shelf
cg16572410	0,936057727	0,719285027	-0,216772699	4,80E-05	LTBP1	LTBP1;LTBP1;LTBP1;LTBP1;LTBP1	NM_000627;NM_206943;NM_001166265;NM_001166266;NM_0	Body;Body;Body;Body;Body	
cg02498177	0,765972747	0,878359933	0,112387186	4,84E-05	SALL1	SALL1;SALL1	NM_002968;NM_001127892	Body;Body	
cg11141503	0,943380211	0,764059479	-0,179320732	4,92E-05	RFFL	RFFL;RFFL	NM_057178;NM_001017368	5UTR;TSS1500	
cg19076479	0,100981695	0,215333136	0,114351441	4,98E-05	L3MBTL3	L3MBTL3;L3MBTL3	NM_032438;NM_001007102	5UTR;5UTR	Island
cg24624090	0,925073997	0,807078973	-0,117995024	4,99E-05	SLIT3	SLIT3	NM_003062	Body	
cg24768002	0,950768597	0,816042895	-0,134725702	5,05E-05	MAML2	MAML2	NM_032427	Body	
cg20908785	0,092662796	0,199869481	0,107206685	5,07E-05	CDKN3	CDKN3;CDKN3	NM_005192;NM_001130851	TSS1500;TSS1500	Island
cg14549811	0,951510216	0,844434732	-0,107075483	5,17E-05	GCNT1	GCNT1;GCNT1;GCNT1;GCNT1;GCNT1;GCNT1	NM_001097633;NM_001097636;NM_001097635;NM_00109763	5UTR;5UTR;5UTR;5UTR;5UTR	
cg13361585	0,942673412	0,816460767	-0,126212645	5,20E-05					
cg03990732	0,906202594	0,784280575	-0,121922018	5,22E-05					
cg02923628	0,931875583	0,809835734	-0,122039849	5,22E-05					
cg08076912	0,898640617	0,732217837	-0,16642278	5,25E-05	ATRIP	ATRIP;ATRIP	NM_130384;NM_032166	Body;Body	S_Shelf
cg12383621	0,926435126	0,767192216	-0,15924291	5,36E-05	RPA3	RPA3	NM_002947	5UTR	
cg14044707	0,94088235	0,818142691	-0,122739659	5,42E-05	USP20	USP20;USP20;USP20	NM_001008563;NM_001110303;NM_006676	Body;Body;Body	N_Shore
cg06660174	0,868538645	0,685138231	-0,183400414	5,55E-05	GPRC5B	GPRC5B	NM_016235	5UTR	N_Shelf
cg10697882	0,881822713	0,765893697	-0,115929016	5,58E-05	MYO16	MYO16	NM_015011	Body	
cg11377650	0,952561783	0,835865548	-0,116696235	5,64E-05	TRIM39	TRIM39;TRIM39	NM_172016;NM_021253	Body;Body	
cg14976342	0,898398104	0,720216644	-0,17818146	5,72E-05	KIAA1026	KIAA1026;KIAA1026;KIAA1026;KIAA1026	NM_201628;NM_001018001;NM_015209;NM_001018000	Body;TSS1500;Body;Body	S_Shelf
cg12591207	0,931557026	0,822378885	-0,109178142	5,77E-05	GPR64	GPR64;GPR64;GPR64;GPR64	NM_005756;NM_001079859;NM_001079860;NM_001079858	3UTR;3UTR;3UTR;3UTR	
cg00095846	0,941278815	0,83641518	-0,104863635	5,84E-05					N_Shelf
cg22909333	0,951120384	0,850325525	-0,100794859	5,84E-05	DOCK1	DOCK1	NM_001380	Body	
cg04741366	0,814438969	0,916491693	0,102052725	5,85E-05					
cg11141463	0,947461012	0,841423941	-0,106037072	5,86E-05					
cg06819279	0,920774608	0,73261769	-0,188156919	5,92E-05	TBPL1	TBPL1	NM_004865	5UTR	
cg21599081	0,720587774	0,847333921	0,126746147	5,94E-05					N_Shelf
cg04048339	0,780886113	0,895360794	0,114474681	5,96E-05	MCCD1	MCCD1	NM_001011700	TSS200	
cg01634458	0,154734191	0,308058528	0,153324337	6,00E-05					Island
cg15025240	0,941250201	0,775982672	-0,165267528	6,04E-05	C2orf65	C2orf65	NM_138804	Body	
cg24606533	0,723579256	0,840289611	0,116710355	6,06E-05	PPAP2B	PPAP2B;PPAP2B	NM_177414;NM_003713	Body;Body	
cg05739305	0,934550231	0,817138574	-0,117411657	6,16E-05					S_Shelf
cg15851882	0,756900628	0,863376455	0,106475827	6,19E-05	MYO1E	MYO1E	NM_004998	Body	
cg13929323	0,067500284	0,168112613	0,100612329	6,22E-05					S_Shelf
cg06224732	0,956968907	0,820853903	-0,136115005	6,23E-05	PMPCB	PMPCB	NM_004279	TSS1500	N_Shore
cg27016549	0,80458126	0,91300345	0,10842219	6,24E-05	NTM	NTM;NTM;NTM;NTM	NM_016522;NM_001144059;NM_001144058;NM_001048209	TSS1500;TSS1500;TSS1500	N_Shore
cg02005425	0,820434603	0,630861988	-0,189572615	6,28E-05	BMS1P4	BMS1P4	NR_026592	TSS1500	N_Shore
cg03719283	0,077540484	0,178992433	0,101451949	6,29E-05	GUCY1A3	GUCY1A3;GUCY1A3;GUCY1A3;GUCY1A3;GUCY1A3;GUCY1A3;GUC	NM_001130687;NM_001130682;NM_001130683;NM_00113068	1stExon;5UTR;TSS1500;5UT	N_Shore
cg26289871	0,118840036	0,232809808	0,113969772	6,32E-05	LPIN3	LPIN3	NM_022896	TSS200	S_Shore
cg04355791	0,096223909	0,214327757	0,118103848	6,35E-05	MIR129-2	MIR129-2	NR_029697	TSS1500	N_Shore
cg09080030	0,898603481	0,750818681	-0,1477848	6,38E-05	SRRM2	SRRM2	NM_016333	Body	S_Shelf
cg25304680	0,861141697	0,632505712	-0,228635985	6,38E-05	TRIO	TRIO	NM_007118	Body	
cg07691531	0,096600819	0,25524208	0,158641262	6,39E-05	NAPRT1	NAPRT1	NM_145201	TSS200	S_Shore
cg25089026	0,925230049	0,799412674	-0,125817374	6,45E-05	OPCML	OPCML;OPCML	NM_002545;NM_001012393	Body;Body	
cg15627871	0,8966297	0,753871805	-0,142757894	6,49E-05					
cg25149391	0,910277958	0,746640776	-0,163637182	6,53E-05	MTL5	MTL5;MTL5	NM_001039656;NM_004923	TSS1500;TSS1500	S_Shore
cg11258102	0,940780247	0,793868086	-0,146912161	6,56E-05	CD58	CD58;CD58;CD58	NM_001779;NR_026665;NM_001144822	Body;Body;Body	S_Shore
cg03957476	0,950468165	0,847549999	-0,102918166	6,58E-05	VAMP4	VAMP4	NM_003762	Body	N_Shelf
cg04140267	0,089618491	0,2331526	0,143534109	6,61E-05	DHRS7B	DHRS7B	NM_015510	TSS1500	N_Shore
cg04326353	0,959797555	0,732288064	-0,227509491	6,71E-05	SULF1	SULF1;SULF1;SULF1;SULF1	NM_001128204;NM_015170;NM_001128205;NM_001128206	Body;Body;Body;Body	
cg14088282	0,113212543	0,263394093	0,15018155	6,71E-05					
cg18646347	0,850400862	0,740937332	-0,10946353	6,81E-05	PTCH2	PTCH2;PTCH2	NM_001166292;NM_003738	Body;Body	N_Shelf
cg05712973	0,901644134	0,745083692	-0,156560442	6,98E-05	PCDH24	PCDH24	NM_017675	Body	
cg14934141	0,928473873	0,8020538	-0,126420072	7,02E-05	NCAPD3	NCAPD3	NM_015261	Body	
cg10508111	0,862957749	0,71786784	-0,145089909	7,08E-05	PFKM	PFKM;PFKM;PFKM;PFKM	NM_001166687;NM_001166688;NM_001166686;NM_000289	TSS1500;TSS1500;Body;5UT	
cg23836814	0,951018233	0,805937582	-0,14508065	7,10E-05	AGBL2	AGBL2	NM_024783	3UTR	
cg23666618	0,923392908	0,727712688	-0,19568022	7,13E-05					S_Shelf
cg11849213	0,970957217	0,834479149	-0,136478068	7,15E-05	PLEKHG3	PLEKHG3	NM_015549	3UTR	S_Shore
cg12132617	0,911715877	0,743634161	-0,168081716	7,20E-05	ADIPOR1	ADIPOR1;ADIPOR1	NM_001127687;NM_015999	TSS1500;TSS1500	S_Shore
cg02114909	0,076643135	0,247537358	0,170894223	7,23E-05	YWHAE	YWHAE;YWHAE	NM_006761;NR_024058	Body;Body	
cg14044785	0,100888858	0,227654896	0,126766037	7,27E-05	THADA	THADA;THADA	NM_022065;NM_001083953	Body;Body	
cg20106465	0,899009825	0,77996716	-0,119042665	7,29E-05	GPAM	GPAM	NM_020918	3UTR	
cg02305024	0,974563452	0,86115619	-0,113407262	7,36E-05					
cg15275312	0,77770949	0,888886612	0,111177122	7,38E-05	TUSC2	TUSC2	NM_007275	3UTR	N_Shelf
cg02127888	0,074516129	0,199256678	0,124740549	7,54E-05	CRAMP1L	CRAMP1L	NM_020825	Body	S_Shore
cg04815147	0,93494471	0,817277276	-0,117667434	7,57E-05					N_Shore
cg14807553	0,915592369	0,776253313	-0,139339056	7,76E-05	ATP11A	ATP11A;ATP11A	NM_015205;NM_032189	3UTR;Body	N_Shelf
cg17199320	0,080826632	0,218936795	0,138110163	7,83E-05	FAM89A	FAM89A	NM_198552	TSS1500	Island
cg26304930	0,821213323	0,621452855	-0,199760469	7,90E-05					
cg08640046	0,142868449	0,254915918	0,112047469	7,91E-05					S_Shore
cg20298425	0,908218021	0,769738361	-0,13847966	8,07E-05	INTS8	INTS8	NM_017864	Body	
cg13261390	0,943136996	0,812102471	-0,131034525	8,22E-05	LRRC16A	LRRC16A	NM_017640	Body	
cg03384915	0,921074131	0,801805604	-0,119268528	8,22E-05	SIN3B	SIN3B	NM_015260	Body	Island

cg09473585	0,923703955	0,722141123	-0,201562831	8,27E-05	C3orf14	C3orf14	NM_020685	5UTR	S_Shore
cg14428911	0,079991128	0,186522485	0,106531357	8,49E-05	SEMA6D	SEMA6D;SEMA6D;SEMA6D;SEMA6D;SEMA6D;SEMA6D	NM_153617;NM_153616;NM_153619;NM_024966;NM_153618;N	TSS1500;TSS1500;TSS1500	Island
cg02689238	0,742865142	0,868278975	0,125413833	8,51E-05	NPAS2	NPAS2	NM_002518	Body	
cg14774995	0,942379242	0,837461305	-0,104917937	8,53E-05	PVT1	PVT1	NR_003367	Body	
cg22329201	0,745126831	0,852007683	0,106880852	8,58E-05					S_Shelf
cg20821424	0,839058037	0,941900655	0,102842619	8,65E-05	VTCN1	VTCN1	NM_024626	TSS1500	
cg01220469	0,109545224	0,258310679	0,148765455	8,67E-05	RNF26	RNF26	NM_032015	TSS1500	Island
cg17459215	0,925860372	0,813048724	-0,112811648	8,75E-05					
cg19239304	0,896472536	0,793529815	-0,102942722	8,89E-05	LRRFIP1	LRRFIP1;LRRFIP1;LRRFIP1;LRRFIP1;LRRFIP1	NM_001137553;NM_004735;NM_001137550;NM_001137552;N	Body;Body;Body;Body;Body	
cg07977927	0,739339078	0,858666771	0,119327693	8,97E-05	LOC154822	LOC154822	NR_024394	Body	N_Shore
cg17495130	0,11007175	0,228780586	0,118708836	8,98E-05	HOXD13	HOXD13	NM_000523	3UTR	N_Shore
cg26124136	0,942807234	0,832100155	-0,110707079	9,10E-05	PDE4D	PDE4D	NM_001165899	Body	
cg18182475	0,928908482	0,797493295	-0,131415186	9,16E-05	RBPJ	RBPJ;RBPJ;RBPJ;RBPJ	NM_203284;NM_203283;NM_015874;NM_005349	Body;Body;Body;Body	
cg24729690	0,117443555	0,222836648	0,105393093	9,39E-05	CPLX2	CPLX2;CPLX2	NM_001008220;NM_006650	5UTR;5UTR	S_Shore
cg18709545	0,085857973	0,207612668	0,121754695	9,44E-05	VAX1	VAX1;VAX1	NM_001112704;NM_199131	3UTR;Body	N_Shore
cg10902396	0,088873366	0,189660082	0,100786716	9,45E-05	CHN2	CHN2	NM_004067	Body	
cg22723876	0,939170716	0,815603778	-0,123566938	9,47E-05	C6orf10	C6orf10	NM_006781	Body	
cg16657453	0,959533377	0,849506476	-0,110026901	9,66E-05	SREBF1	SREBF1;SREBF1	NM_004176;NM_001005291	Body;Body	N_Shelf
cg10775173	0,713730058	0,829702677	0,115972619	9,74E-05	PRKAG2	PRKAG2	NM_016203	Body	
cg04307594	0,726147683	0,842447949	0,116300266	9,81E-05	LOC284578	LOC284578	NR_027086	TSS200	
cg15477011	0,130182224	0,239723909	0,109541685	0,000100687	FOXP1	FOXP1;FOXP1	NM_001012505;NM_032682	TSS1500;TSS1500	S_Shore
cg26231350	0,970025301	0,8661258	-0,1038995	0,000100784					
cg00460763	0,888924405	0,641998051	-0,246926354	0,000101861	RPSAP58	RPSAP58	NR_003662	TSS1500	N_Shore
cg27570992	0,800462546	0,906303286	0,10584074	0,000102787	ATXN7L1	ATXN7L1;ATXN7L1	NM_152749;NM_020725	Body;Body	
cg10073794	0,789382345	0,897106205	0,10772386	0,000103748					N_Shelf
cg00348623	0,944898066	0,793541363	-0,151356703	0,000104176	CRAMP1L	CRAMP1L	NM_020825	Body	Island
cg18659109	0,895999557	0,737284167	-0,15871539	0,000106217	CDS1	CDS1	NM_001263	Body	S_Shelf
cg11837390	0,928103814	0,81139101	-0,116712804	0,000107147	TSC22D3	TSC22D3;TSC22D3;TSC22D3	NM_198057;NM_004089;NM_001015881	3UTR;3UTR;3UTR	N_Shelf
cg18739392	0,931273803	0,829681831	-0,101591972	0,000107383	DNAJC18	DNAJC18	NM_152686	Body	
cg01723163	0,957948912	0,832563972	-0,125384941	0,000107504					
cg15078825	0,815362504	0,920137266	0,104774762	0,000108392					
cg10523193	0,906445613	0,779103334	-0,127342279	0,000109171	SNORA25	SNORA25	NR_003028	Body	
cg19010223	0,926068472	0,758436288	-0,167632184	0,000109262	CCNB1IP1	CCNB1IP1;CCNB1IP1;CCNB1IP1;CCNB1IP1	NM_182851;NM_021178;NM_182852;NM_182849	TSS1500;5UTR;TSS1500;5U	
cg22646311	0,080505133	0,237651996	0,157146863	0,000109516					S_Shore
cg13451882	0,931418513	0,818984109	-0,112434405	0,000109759					N_Shelf
cg12737285	0,921118046	0,812319455	-0,108798592	0,000110291					
cg08186584	0,884430522	0,766522161	-0,117908361	0,00011063	CCDC101	CCDC101	NM_138414	TSS1500	N_Shore
cg03819338	0,935512228	0,804895427	-0,130616802	0,000110809					N_Shelf
cg01521225	0,93689083	0,791269802	-0,145621028	0,000111881	NID1	NID1	NM_002508	Body	N_Shore
cg19739098	0,794919195	0,902225773	0,107306578	0,000112233	PTPRN2	PTPRN2;PTPRN2;PTPRN2	NM_002847;NM_130842;NM_130843	Body;Body;Body	
cg23498106	0,957660473	0,834117088	-0,123543385	0,000112891					
cg08645827	0,102499852	0,270519578	0,168019726	0,000113809	CCT6B	CCT6B	NM_006584	Body	N_Shelf
cg15913856	0,933591925	0,707198122	-0,226393804	0,000114915	LOC100303728	LOC100303728	NR_028443	Body	N_Shelf
cg07709155	0,914021917	0,79953137	-0,114490538	0,000114983					S_Shore
cg15736360	0,926587684	0,785856954	-0,14073073	0,000115424	KCNMB2	KCNMB2	NM_181361	TSS1500	
cg22902799	0,064735619	0,17334104	0,108605421	0,000115647	CLEC14A	CLEC14A	NM_175060	TSS1500	S_Shore
cg00131557	0,967403804	0,861770037	-0,105633767	0,000116641	DNAJC15	DNAJC15	NM_013238	TSS1500	N_Shore
cg20748012	0,953107851	0,844904337	-0,108203514	0,000117153					
cg21445553	0,721083851	0,82586495	0,104781099	0,000117424	GGTLC1	GGTLC1	NM_178311	5UTR	Island
cg04344233	0,784359962	0,903437138	0,119077176	0,000118068	TNXB	TNXB	NM_019105	Body	
cg12974607	0,938763059	0,821890097	-0,116872962	0,000118136	ASB9	ASB9;ASB9;ASB9;ASB9	NM_001031739;NM_001168531;NM_024087;NM_001168530	Body;3UTR;3UTR;3UTR	
cg14805174	0,93382481	0,761964061	-0,171860749	0,000118412	TJP2	TJP2;TJP2;TJP2;TJP2;TJP2;TJP2	NM_001170414;NM_001170630;NM_001170415;NM_201629;N	Body;Body;Body;Body;Body;B	N_Shore
cg15715477	0,084394563	0,321347923	0,23695336	0,00011925					Island
cg27530186	0,901156808	0,790714128	-0,110442681	0,000119357	CAMKK2	CAMKK2;CAMKK2;CAMKK2;CAMKK2;CAMKK2;CAMKK2;CAMKK2	NM_172215;NM_153499;NM_006549;NM_172214;NM_153500;N	Body;Body;Body;Body;Body;B	
cg25302087	0,853535373	0,710125058	-0,143410314	0,000119652	FLJ22536	FLJ22536	NR_015410	Body	S_Shore
cg26278611	0,893948189	0,702999415	-0,190948774	0,000119925	FAHD2B	FAHD2B	NM_199336	Body	N_Shelf
cg18522626	0,938982628	0,829758461	-0,109224167	0,000119991	PPM1A	PPM1A	NM_177952	TSS1500	
cg06730624	0,725823692	0,846529949	0,120706257	0,000120133					
cg02522311	0,099162913	0,209275338	0,110112425	0,00012021	C6orf48	C6orf48;SNORD48;C6orf48	NM_001040437;NR_002745;NM_001040438	TSS1500;TSS1500;TSS1500	N_Shore
cg25425994	0,736764133	0,873173546	0,136409413	0,000121874					S_Shore
cg21192063	0,242696197	0,141573594	-0,101122602	0,000122903	SMARCD3	SMARCD3;SMARCD3	NM_001003802;NM_003078	Body;Body	Island
cg12726960	0,110024938	0,22720557	0,117180632	0,000123051	STAC	STAC	NM_003149	TSS1500	Island
cg13617087	0,711269047	0,824357002	0,113087954	0,000123397	PSORS1C1	PSORS1C1;PSORS1C2	NM_014068;NM_014069	Body;TSS200	
cg15491540	0,901767386	0,709921689	-0,191845697	0,00012403	SPTLC3	SPTLC3	NM_018327	3UTR	
cg00139496	0,947734341	0,73389565	-0,213838692	0,000124104	GLT8D1	GLT8D1;GLT8D1;GLT8D1	NM_018446;NM_152932;NM_001010983	Body;Body;Body	
cg00877619	0,934063219	0,769749989	-0,16431823	0,000124394	SCN10A	SCN10A	NM_006514	Body	
cg03653845	0,758667246	0,890846387	0,132179141	0,000124735	PRELID2	PRELID2;PRELID2;PRELID2	NM_205846;NM_138492;NM_182960	Body;Body;Body	
cg14200859	0,892520181	0,776394792	-0,116125389	0,000125135	RALGPS1	RALGPS1	NM_014636	5UTR	
cg19564017	0,953927498	0,841234621	-0,112692877	0,00012636	AHI1	AHI1;AHI1;AHI1;AHI1	NM_001134832;NM_001134831;NM_017651;NM_001134830	Body;Body;Body;Body	
cg13319823	0,930899725	0,830850414	-0,100049311	0,000126943	ZNF599	ZNF599	NM_001007248	TSS1500	S_Shore
cg22076764	0,920261166	0,79202665	-0,128234509	0,000127485	LGALS13	LGALS13	NM_013268	TSS1500	
cg01671881	0,730756247	0,834806578	0,104050331	0,00012769	C20orf71	C20orf71;C20orf71	NM_178466;NM_001042439	TSS1500;TSS1500	
cg22465824	0,916530061	0,756354826	-0,160175235	0,000128555	CDK2AP1	CDK2AP1	NM_004642	3UTR	
cg17031475	0,898352355	0,728538954	-0,169813401	0,000128699	GRASP	GRASP	NM_181711	3UTR	S_Shore
cg15863374	0,930341538	0,81696244	-0,113379098	0,00013073	POLR3F	POLR3F	NM_006466	3UTR	
cg08148802	0,731613768	0,848186782	0,116573014	0,000131056	LSP1	LSP1;LSP1;LSP1;LSP1	NM_001013255;NM_001013253;NM_001013254;NM_002339	Body;Body;Body;Body	Island
cg02670503	0,71222598	0,822026486	0,109800507	0,000133281	FRMPD2	FRMPD2;FRMPD2;FRMPD2;FRMPD2	NM_001018071;NM_152428;NM_001018071;NM_152428	5UTR;1stExon;1stExon;5UTR	
cg13865248	0,838337801	0,652461546	-0,185876255	0,000133462					
cg20525486	0,055425978	0,180305505	0,124879528	0,00013363	FOXP1	FOXP1	NM_032682	Body	N_Shore
cg18864227	0,100767215	0,22438667	0,123619456	0,000135108					
cg19330334	0,080979946	0,276246203	0,195266257	0,000136356	IQGAP1	IQGAP1	NM_003870	Body	
cg02484634	0,962062385	0,822582914	-0,139479471	0,00013636					N_Shore
cg09160211	0,928160896	0,807738196	-0,1204227	0,000136821					S_Shelf
cg08811888	0,760679417	0,862632533	0,101953116	0,000137172	CBX6	CBX6	NM_014292	Body	Island
cg17083798	0,892268962	0,702945586	-0,189323376	0,00013833					N_Shelf
cg14709817	0,926175338	0,751613281	-0,174562057	0,000139525	CUGBP1	CUGBP1	NM_006560	5UTR	
cg14552904	0,831712189	0,683350688	-0,148361501	0,000139548	XKR4	XKR4	NM_052898	Body	
cg02118559	0,930932522	0,830774344	-0,100158178	0,000139694	CTNND2	CTNND2	NM_001332	3UTR	
cg26426745	0,313645281	0,197378821	-0,11626646	0,000139877	LOC100132111	LOC100132111;C2CD4D	NR_024237;NM_001136003	TSS1500;Body	Island
cg23571456	0,048558331	0,148864442	0,100306111	0,000140171	BTN2A2	BTN2A2;BTN2A2;BTN2A2;BTN2A2	NM_181531;NM_006995;NM_181531;NM_006995	5UTR;1stExon;1stExon;5UTR	
cg16946271	0,845630238	0,669970352	-0,175659885	0,000140465	MAN1A1	MAN1A1	NM_005907	Body	N_Shelf
cg13880545	0,946626918	0,844169572	-0,102457346	0,000140892	MED27	MED27	NM_004269	Body	
cg15493970	0,809629636	0,91994524	0,110315604	0,000141569	PPFIA4	PPFIA4	NM_015053	3UTR	S_Shore
cg18377446	0,958407184	0,841048004	-0,11735918	0,000141778	AGL	AGL;AGL;AGL;AGL;AGL;AGL	NM_000645;NM_000028;NM_000642;NM_000646;NM_000644;N	Body;Body;Body;Body;Body;B	
cg06489912	0,94833078	0,822820098	-0,125510682	0,000143116	DIP2B	DIP2B	NM_173602	Body	
cg09097839	0,867940837	0,755663117	-0,112277721	0,000143524					
cg15053253	0,869576882	0,747928014	-0,121648868	0,000143904					
cg10722444	0,73010344	0,87020423	0,14010079	0,000144231					Island
cg26135573	0,645501875	0,76520091	0,119699035	0,00014443					Island
cg20160729	0,968156395	0,862453531	-0,105702864	0,000145537	RNF216	RNF216;RNF216	NM_207111;NM_207116	Body;Body	
cg13690543	0,083747344	0,21627921	0,132531866	0,000146747	BAT2	BAT2	NM_080686	TSS1500	N_Shore
cg05714732	0,961892204	0,846612525	-0,115279679	0,000146885					N_Shore
cg24890545	0,950373105	0,842485603	-0,107887502	0,000147208	MAP3K5	MAP3K5	NM_005923	Body	
cg05472062	0,123829833	0,263145884	0,13931605	0,000147496	TNKS2	TNKS2	NM_025235	Body	S_Shore
cg09449927	0,825712979	0,651560401	-0,174152578	0,00014762	MAGI1	MAGI1;MAGI1;MAGI1	NM_015520;NM_001033057;NM_004742	Body;Body;Body	
cg18646208	0,904545247	0,757689008	-0,146856239	0,000147795					N_Shelf
cg25590318	0,936001826	0,816375751	-0,119626074	0,000147926					N_Shelf
cg19598403	0,924684385	0,783012983	-0,141671402	0,000149326	TECPR2	TECPR2	NM_014844	Body	
cg00352872	0,761767314	0,868616189	0,106848874	0,000149866	DHRS7C	DHRS7C	NM_001105571	TSS1500	
cg14279856	0,97048429	0,853199565	-0,117284724	0,000150646	DDR1	DDR1;DDR1	NM_001954;NM_013993	TSS1500;TSS1500	N_Shore
cg12125241	0,854872688	0,745254933	-0,109617755	0,000151033	PDE8A	PDE8A;PDE8A	NM_173454;NM_002605	Body;Body	
cg13486491	0,954469854	0,818279673	-0,136190182	0,000151479	SPG20	SPG20	NM_001142294	TSS1500	
cg21047322	0,919511113	0,785275211	-0,134235902	0,000151996	AKAP12	AKAP12;AKAP12	NM_144497;NM_005100	Body;Body	S_Shelf
cg23473276	0,918177194	0,805993609	-0,112183585	0,000153222					
cg21218627	0,727657556	0,833746748	0,106089192	0,000153868	FREQ	FREQ;FREQ	NM_014286;NM_001128826	3UTR;3UTR	
cg06811800	0,738243533	0,84782538	0,109581847	0,000153913	ATP4B	ATP4B	NM_000705	Body	
cg09497391	0,953894572	0,844587321	-0,109307251	0,000155583	TPST1	TPST1	NM_003596	Body	
cg03087622	0,826385053	0,659722008	-0,166663045	0,000155761	ITGAD	ITGAD	NM_005353	TSS1500	
cg27367142	0,745986078	0,8537583	0,107772222	0,000155905					
cg11996397	0,501051829	0,640135563	0,139083733	0,000156059					
cg03773226	0,115190994	0,226519321	0,111328326	0,000157389	CACNB1	CACNB1;CACNB1;CACNB1	NM_199247;NM_000723;NM_199248	Body;Body;Body	Island
cg18793198	0,645849949	0,77390712	0,128057171	0,000159154					S_Shore
cg22744783	0,923551044	0,77258045	-0,150970593	0,000159451	SEMA5A	SEMA5A	NM_003966	Body	
cg27228210	0,947057234	0,834474886	-0,112582348	0,00016008					
cg19682024	0,922884461	0,785254363	-0,137630099	0,000161593	HEATR4	HEATR4;ACOT1	NM_203309;NM_001037161	5UTR;Body	S_Shelf
cg01871214	0,06112287	0,161372845	0,100249975	0,000161648					Island
cg15082498	0,793010896	0,895731857	0,102720961	0,000163231	JAK1	JAK1	NM_002227	5UTR	
cg07291439	0,150686543	0,265179873	0,11449333	0,00016379	ZIC1	ZIC1	NM_003412	TSS1500	N_Shore
cg19263761	0,877336576	0,692341476	-0,1849951	0,000163807	CECR5	CECR5;CECR5	NM_017829;NM_033070	Body;Body	Island
cg14975061	0,954133356	0,844758954	-0,109374402	0,000164482					
cg06479434	0,921376081	0,76161702	-0,159759061	0,000165329	CALU	CALU;CALU	NM_001130674;NM_001219	Body;Body	
cg27358426	0,0447007	0,159149173	0,114448472	0,000165377					N_Shelf
cg08550689	0,918143771	0,762641328	-0,155502443	0,000166196	CACNB2	CACNB2;CACNB2;CACNB2;CACNB2;CACNB2;CACNB2;CA	NM_201571;NM_201593;NM_000724;NM_201597;NM_201570;N	Body;Body;Body;Body;TSS15	
cg11834729	0,916644693	0,7643627	-0,152281993	0,000166811	MIR488	MIR488;ASTN1;ASTN1	NR_030163;NM_207108;NM_004319	TSS200;Body;Body	
cg03812676	0,865880971	0,639989187	-0,225891785	0,000167377	AIP	AIP	NM_003977	Body	S_Shelf
cg23154743	0,052724903	0,153519457	0,100794554	0,000167467	NFKBIL1	NFKBIL1;NFKBIL1;ATP6V1G2;NFKBIL1;NFKBIL1;ATP6V1G	NM_005007;NM_001144963;NM_130463;NM_005007;NM_0011	5UTR;5UTR;TSS1500;1stExo	
cg18464903	0,9268273	0,763801327	-0,163025973	0,000168402	IGF2BP2	IGF2BP2;IGF2BP2	NM_001007225;NM_006548	Body;Body	
cg21507854	0,9272996	0,756557982	-0,170741618	0,000168776	CCDC77	CCDC77;CCDC77	NM_001130147;NM_001130148	5UTR;5UTR	N_Shelf
cg20100801	0,938225687	0,834782666	-0,103443021	0,000169166					
cg10709282	0,061791751	0,166272374	0,104480624	0,000169674					
cg02680903	0,146797026	0,296214536	0,14941751	0,000170035					S_Shelf
cg19656847	0,813465634	0,919838517	0,106372883	0,000170639					
cg00287711	0,033292837	0,13834375	0,105050913	0,00017085	TMEM126B	TMEM126B;DLG2	NM_018480;NM_001142699	TSS1500;TSS200	N_Shore
cg07258419	0,953778275	0,748643571	-0,205134704	0,000172353					
cg23501520	0,922130473	0,75686979	-0,165260684	0,000173226	PNLIPRP1	PNLIPRP1	NM_006229	Body	N_Shore
cg06601633	0,101117282	0,205899865	0,104782582	0,00017353	TRIM9	TRIM9;TRIM9	NM_015163;NM_052978	1stExon;1stExon	Island
cg23793599	0,729567863	0,839966226	0,110398363	0,000173998	FXYD6	FXYD6	NM_001164831	TSS1500	S_Shore
cg02183531	0,91489899	0,790590231	-0,124308759	0,000174142	CCT4	CCT4	NM_006430	Body	N_Shelf
cg07277618	0,933321884	0,784308863	-0,149013021	0,000175241	SLCO3A1	SLCO3A1;SLCO3A1	NM_001145044;NM_013272	Body;Body	S_Shore
cg20964594	0,214216627	0,383784676	0,169568049	0,000177244	CPZ	CPZ;CPZ;CPZ	NM_003652;NM_001014448;NM_001014447	TSS200;TSS200;TSS200	N_Shore
cg16990532	0,111007427	0,238415976	0,127408549	0,0001773	KIAA1274	KIAA1274	NM_014431	TSS200	Island
cg10435664	0,058204735	0,170750907	0,112546172	0,000177934	PLXNA2	PLXNA2	NM_025179	TSS1500	S_Shore
cg19963120	0,937708027	0,763831082	-0,173876946	0,000178527	UGGT2	UGGT2	NM_020121	Body	
cg04396251	0,811694235	0,914331899	0,102637664	0,000178994	KNDC1	KNDC1	NM_152643	Body	S_Shore
cg22083866	0,911761876	0,791373812	-0,120388064	0,000179155					S_Shelf
cg00740368	0,088373953	0,222440593	0,13406664	0,000179156					S_Shore
cg10841138	0,800685834	0,902200032	0,101514198	0,000179496	RIMBP2	RIMBP2	NM_015347	Body	
cg15910502	0,079023194	0,18356543	0,104542236	0,000180029	KAT2B	KAT2B	NM_003884	Body	S_Shelf
cg01510153	0,940616321	0,783338624	-0,157277697	0,000181367	BRAF	BRAF	NM_004333	Body	N_Shelf
cg00751021	0,921892123	0,811019651	-0,110872472	0,000182565	PARD3B	PARD3B;PARD3B;PARD3B	NM_152526;NM_057177;NM_205863	Body;Body;Body	
cg00591160	0,112493136	0,220511286	0,10801815	0,000182753	ING4	ING4;ING4;ING4;ING4;ING4;ING4;ING4;ING4;ING4;ING4;ING4	NM_001127585;NM_001127583;NM_001127582;NM_00112758	1stExon;5UTR;1stExon;1stEx	Island
cg05383332	0,705620247	0,810040889	0,104420641	0,00018295					
cg18794882	0,922271897	0,7411016	-0,181170297	0,000183316	IAH1	IAH1;CPSF3	NM_001039613;NM_016207	TSS1500;3UTR	N_Shore
cg20391793	0,935244666	0,826552919	-0,108691747	0,000184607					
cg03435901	0,897168472	0,772720192	-0,12444828	0,000184771	IL17RD	IL17RD	NM_017563	3UTR	
cg20602914	0,954023743	0,787368393	-0,166655351	0,000185265	TNRC18	TNRC18	NM_001080495	Body	S_Shelf
cg02312154	0,941761379	0,793479584	-0,148281795	0,000185485	THADA	THADA;THADA	NM_022065;NM_001083953	Body;Body	
cg24111796	0,142085397	0,324081292	0,181995895	0,000185955					S_Shore
cg09140723	0,939785495	0,801312738	-0,138472757	0,000186251	SGPL1	SGPL1	NM_003901	Body	
cg06292709	0,736439379	0,85686187	0,120422492	0,000187386					
cg12403162	0,061766316	0,199635584	0,137869269	0,000189879	ABLIM1	ABLIM1;ABLIM1;ABLIM1	NM_001003408;NM_001003407;NM_002313	Body;Body;Body	S_Shelf
cg00155310	0,041214224	0,142662546	0,101448321	0,000190324	TFAP2B	TFAP2B	NM_003221	3UTR	S_Shore
cg17733824	0,880809948	0,709721878	-0,171088071	0,000191731	SLC12A7	SLC12A7	NM_006598	Body	Island
cg24340565	0,923382927	0,775456584	-0,147926343	0,000191866	VGLL4	VGLL4;VGLL4	NM_014667;NM_001128219	Body;TSS1500	S_Shore
cg05757376	0,859325426	0,653522623	-0,205802803	0,000192103	MIR548F5	MIR548F5;DCLK1	NR_031646;NM_004734	TSS1500;Body	
cg14587391	0,099456735	0,238562715	0,13910598	0,000193877					
cg03274069	0,777608788	0,892136323	0,114527535	0,00019478	AVPR2	AVPR2;AVPR2;AVPR2	NM_001146151;NR_027419;NM_000054	Body;Body;Body	
cg14181345	0,806375295	0,637061679	-0,169313616	0,000194922					N_Shore
cg27631877	0,941714828	0,781604896	-0,160109933	0,00019562	LPIN1	LPIN1	NM_145693	Body	
cg24902196	0,743507693	0,852526332	0,109018639	0,000196908					
cg17993442	0,05247471	0,162193952	0,109719242	0,000197178	ZBTB44	ZBTB44	NM_014155	5UTR	N_Shore

cg21865751	0,769324158	0,879597483	0,110273325	0,000197343					
cg03843656	0,953625268	0,850964981	-0,102660287	0,00019814	WNT5A	WNT5A	NM_003392	3UTR	N_Shelf
cg19611002	0,657988169	0,776154577	0,118166407	0,000199018	ACTL9	ACTL9	NM_178525	TSS1500	S_Shore
cg17847446	0,068115056	0,175516186	0,10740113	0,000199439	PDIA3	PDIA3;CATSPER2P1	NM_005313;NR_002318	Body;TSS1500	S_Shore
cg02138806	0,941976066	0,783449813	-0,158526252	0,000200907					
cg16997732	0,147010494	0,307990405	0,160979911	0,000201316	BAZ1B	BAZ1B	NM_032408	Body	Island
cg02301292	0,784107133	0,884664816	0,100557684	0,000201451					
cg14838188	0,085331411	0,202272627	0,116941216	0,000203634	C10orf26	C10orf26	NM_001083913	TSS200	
cg00747241	0,916128257	0,779658104	-0,136470153	0,000204271	ASPA	ASPA	NM_001128085	TSS200	S_Shelf
cg19887630	0,076676525	0,179216555	0,10254003	0,000204714	MFGE8	MFGE8;MFGE8	NM_001114614;NM_005928	TSS1500;TSS1500	S_Shore
cg19822890	0,945570393	0,818392373	-0,12717802	0,000205667	DOCK9	DOCK9;DOCK9	NM_015296;NM_001130049	Body;Body	
cg06907229	0,908245134	0,755063484	-0,153181649	0,000205684	PSTK	PSTK	NM_153336	3UTR	
cg07939743	0,063230131	0,165411044	0,102180913	0,000206153					
cg09403480	0,930110143	0,809646898	-0,120463245	0,000206591					N_Shelf
cg13753526	0,924385317	0,801950556	-0,122434761	0,000207482	MELK	MELK	NM_014791	3UTR	
cg06834313	0,823681992	0,924749403	0,101067411	0,000207675					
cg11576590	0,884276523	0,687498959	-0,196777565	0,000208344					S_Shelf
cg01326206	0,858342412	0,731116311	-0,127226101	0,000208472	DHX30	DHX30;DHX30	NM_014966;NM_138615	5UTR;5UTR	S_Shore
cg14921485	0,91622758	0,79679724	-0,11943034	0,000209407					N_Shelf
cg17271146	0,878781708	0,757859902	-0,120921806	0,000209936	COPZ1	COPZ1	NM_016057	TSS1500	
cg19777992	0,047558151	0,152713144	0,105154993	0,000210063	NDUFS4	NDUFS4	NM_002495	Body	Island
cg14018414	0,898156876	0,727013397	-0,171143479	0,000211991					S_Shelf
cg01174459	0,952959631	0,843319958	-0,109639673	0,000212006	C12orf75	C12orf75	NM_001145199	3UTR	
cg21933078	0,711625366	0,817478752	0,105853385	0,000214207					N_Shore
cg20718855	0,780827565	0,631811128	-0,149016437	0,000214563	NRXN2	NRXN2;NRXN2	NM_138732;NM_015080	Body;Body	N_Shore
cg07186032	0,707280868	0,818759428	0,11147856	0,000214759	ARHGEF3	ARHGEF3	NM_001128615	Body	
cg13527016	0,083977914	0,186058327	0,102080413	0,000215201	FAM45B	FAM45B;FAM45A	NR_027141;NM_207009	Body;Body	Island
cg25996586	0,847795737	0,659327563	-0,188468174	0,000215745	NPR3	NPR3	NM_000908	3UTR	
cg12785983	0,958345367	0,856819637	-0,10152573	0,000215763	TMEM101	TMEM101	NM_032376	3UTR	N_Shelf
cg06645286	0,728695982	0,576107908	-0,152588074	0,000216122	C20orf46	C20orf46	NM_018354	TSS200	N_Shore
cg18142262	0,052554239	0,175858488	0,123304249	0,000216728					
cg21476463	0,754113675	0,855307559	0,101193884	0,00021774	RBP1	RBP1;RBP1;RBP1	NM_001130992;NM_001130993;NM_002899	TSS1500;TSS1500;TSS1500	S_Shore
cg01866022	0,821448083	0,686907893	-0,134540191	0,000218733					S_Shelf
cg23619338	0,930812145	0,778740829	-0,152071316	0,000218841					
cg10668195	0,814442166	0,919939531	0,105497364	0,0002195					S_Shelf
cg04691944	0,82254284	0,584369105	-0,238173735	0,00022079	MCF2L	MCF2L;MCF2L	NM_001112732;NM_024979	Body;Body	N_Shore
cg03689601	0,912265048	0,793323775	-0,118941273	0,000221111	PUS7L	PUS7L;PUS7L;PUS7L	NM_031292;NM_001098614;NM_001098615	3UTR;3UTR;3UTR	
cg09171882	0,134415077	0,239038163	0,104623086	0,000221145	GLIPR1	GLIPR1	NM_006851	TSS200	
cg09554406	0,767854992	0,885556195	0,117701203	0,000221146	TRPM4	TRPM4	NM_017636	Body	Island
cg10351052	0,951540299	0,851049123	-0,100491176	0,000221463					S_Shelf
cg19921237	0,899979514	0,795998833	-0,103980681	0,000222225	DIP2C	DIP2C	NM_014974	Body	
cg08506775	0,066260696	0,198655618	0,132394922	0,000222326	SERPINE1	SERPINE1;SERPINE1	NM_001165413;NM_000602	TSS200;TSS200	
cg19631779	0,941457627	0,827511396	-0,11394623	0,000223428	TPM1	TPM1;TPM1;TPM1;TPM1;TPM1;TPM1;TPM1	NM_001018007;NM_001018020;NM_001018004;NM_00101800	Body;Body;Body;TSS1500;Bo	N_Shore
cg07850246	0,102153331	0,226530053	0,124376721	0,000223919	C11orf40	C11orf40	NM_144663	TSS1500	
cg08543244	0,95190746	0,84338497	-0,10852249	0,000224927	C7orf31	C7orf31	NM_138811	Body	N_Shelf
cg26318321	0,949717625	0,840822165	-0,10889546	0,000225474					S_Shelf
cg25519723	0,922293809	0,801687446	-0,120606363	0,00022661					
cg22443054	0,910823781	0,80196254	-0,108861241	0,000227436	NCSTN	NCSTN;COPA;COPA	NM_015331;NM_004371;NM_001098398	Body;TSS1500;TSS1500	S_Shore
cg18371410	0,908954619	0,786456844	-0,122497776	0,000227529	LOC285830	LOC285830;LOC285830	NR_026972;NR_026973	Body;Body	
cg26099899	0,939729359	0,813068107	-0,126661252	0,000227732					S_Shelf
cg25969460	0,95265879	0,848897371	-0,103761418	0,000228735	AHCYL2	AHCYL2;AHCYL2;AHCYL2;AHCYL2	NM_001130722;NM_001130723;NM_001130720;NM_015328	Body;Body;Body;Body	
cg08324862	0,939829461	0,817962266	-0,121867196	0,000229481					
cg07589531	0,853982052	0,733257215	-0,120724837	0,000229894	FRMD4A	FRMD4A	NM_018027	Body	
cg19843226	0,910532843	0,775881608	-0,134651235	0,000230052	ESRRB	ESRRB	NM_004452	TSS1500	
cg24495475	0,88705579	0,780948463	-0,106107327	0,000234275					
cg03638479	0,068356667	0,175929249	0,107572581	0,000234626	ABHD14B	ABHD14B;ABHD14B;ABHD14A;ABHD14B	NM_001146314;NM_032750;NM_015407;NR_027476	TSS1500;TSS1500;Body;TSS	S_Shore
cg22918700	0,763052762	0,86422772	0,101174958	0,000234637	FITM1	FITM1	NM_203402	TSS1500	N_Shore
cg05785102	0,776173733	0,876927449	0,100753716	0,00023517	NDUFB10	NDUFB10	NM_004548	TSS1500	N_Shore
cg00391030	0,395665357	0,535169885	0,139504528	0,000235955	PARP11	PARP11	NM_020367	Body	Island
cg04915618	0,918008932	0,81506774	-0,102941192	0,000237668	KALRN	KALRN;KALRN	NM_001024660;NM_007064	Body;Body	S_Shore
cg27315157	0,084225981	0,203382969	0,119156988	0,000238182	IFI44L	IFI44L;IFI44L	NM_006820;NM_006820	5UTR;1stExon	
cg03418552	0,064195242	0,194948037	0,130752795	0,000239464	CADM1	CADM1;CADM1	NM_014333;NM_001098517	TSS200;TSS200	Island
cg02331189	0,707923467	0,813326756	0,105403289	0,000240122					S_Shore
cg15305633	0,905177	0,733575433	-0,171601567	0,000243222	TP53RK	TP53RK	NM_033550	TSS1500	S_Shore
cg17147909	0,783409506	0,88906379	0,105654284	0,000244737					
cg24228532	0,717859684	0,852137186	0,134277502	0,000245495					S_Shelf
cg01515444	0,740638232	0,871554003	0,130915771	0,000246212					N_Shore
cg19125791	0,912140406	0,791041163	-0,121099243	0,000246892	HDAC4	HDAC4	NM_006037	Body	
cg15831722	0,809121205	0,910279531	0,101158326	0,000248363	DENND4B	DENND4B;CRTC2	NM_014856;NM_181715	TSS1500;3UTR	S_Shore
cg21376136	0,15977168	0,314153486	0,154381807	0,000249258	CACNB4	CACNB4;CACNB4;CACNB4;CACNB4	NM_000726;NM_001145798;NM_001005746;NM_001005747	Body;Body;Body;TSS200	
cg08885659	0,058734241	0,200427773	0,141693533	0,000249336	BAT3	BAT3;BAT3;BAT3;BAT3;BAT3	NM_001098534;NM_004639;NM_080702;NM_001098534;NM_0	5UTR;5UTR;5UTR;1stExon;5U	Island
cg12799265	0,037429847	0,143144283	0,105714436	0,000249818	LEPRE1	LEPRE1;LEPRE1;C1orf50	NM_022356;NM_001146289;NM_024097	Body;Body;TSS1500	N_Shore
cg15175581	0,673898773	0,792639516	0,118740743	0,000250652	FMN1	FMN1	NM_001103184	TSS200	
cg00483100	0,903353039	0,7651128	-0,138240239	0,000251468					N_Shore
cg13147721	0,900255333	0,747391299	-0,152864033	0,000252235					S_Shelf
cg07253712	0,199510643	0,329235032	0,129724389	0,000252766	MYO10	MYO10	NM_012334	Body	N_Shelf
cg23967474	0,975451681	0,83028671	-0,145164971	0,000255933	MDN1	MDN1	NM_014611	Body	N_Shelf
cg01985595	0,940340238	0,809564427	-0,130775811	0,00025801	PDE7B	PDE7B	NM_018945	Body	
cg21864706	0,944111155	0,752355639	-0,191755516	0,000259067	GSG1	GSG1;GSG1	NM_001080554;NM_001080555	TSS1500;TSS1500	S_Shelf
cg16709836	0,209576102	0,313726135	0,104150033	0,000259152	BHLHE40	BHLHE40	NM_003670	Body	N_Shore
cg06835074	0,952347271	0,81525412	-0,137093151	0,00025956	LRIT2	LRIT2	NM_001017924	TSS200	
cg02613827	0,139008541	0,252620601	0,11361206	0,000260022					S_Shore
cg08315825	0,957012293	0,847630258	-0,109382035	0,000261005	IGF1R	IGF1R	NM_000875	Body	
cg06588645	0,086028692	0,187052016	0,101023324	0,000261476	TNS3	TNS3	NM_022748	5UTR	Island
cg25124109	0,785301908	0,895051858	0,10974995	0,000261805					Island
cg10973043	0,944003322	0,815116905	-0,128886417	0,000263264	RPL21	RPL21	NM_000982	TSS1500	N_Shore
cg07682041	0,157310787	0,271852843	0,114542056	0,00026388					
cg07410210	0,937051018	0,793760187	-0,143290831	0,000266578	NFU1	NFU1;NFU1;NFU1;NFU1	NM_001002755;NM_001002756;NM_001002757;NM_015700	Body;5UTR;5UTR;5UTR	N_Shore
cg06256785	0,873325055	0,769527075	-0,10379798	0,000267188	ZMYM3	ZMYM3;BCYRN1;ZMYM3;ZMYM3	NM_005096;NR_001568;NM_201599;NM_001171162	3UTR;Body;3UTR;3UTR	
cg05486188	0,084088938	0,198491052	0,114402114	0,000267433	KCNIP2	KCNIP2;KCNIP2;KCNIP2;KCNIP2;KCNIP2;KCNIP2;KCNIP2	NM_173195;NM_173191;NM_173193;NM_173194;NM_173192;N	Body;Body;Body;Body;Body;B	Island
cg13304297	0,113778725	0,232127256	0,118348531	0,000267758	C3orf50	C3orf50	NR_021485	Body	S_Shore
cg05561632	0,827893626	0,92911987	0,101226243	0,000268371					
cg26326607	0,929481987	0,827860655	-0,101621332	0,000269241	MRPL23	MRPL23	NM_021134	Body	N_Shore
cg22933439	0,919740316	0,731743069	-0,187997247	0,000269251	S100A7A	S100A7A	NM_176823	TSS1500	
cg01522880	0,1290592	0,246167242	0,117108042	0,000269335	PSMD7	PSMD7	NM_002811	Body	S_Shore
cg16771104	0,711502657	0,822946112	0,111443455	0,000269349	HESX1	HESX1;HESX1	NM_003865;NM_003865	1stExon;5UTR	
cg01861537	0,930197346	0,746133896	-0,18406345	0,000269474					
cg01432552	0,116649922	0,221555404	0,104905482	0,000272014	PTPRR	PTPRR;PTPRR	NM_130846;NM_002849	Body;Body	
cg20829404	0,084189882	0,214778459	0,130588577	0,000272523	COL9A3	COL9A3	NM_001853	TSS200	Island
cg10015572	0,802603345	0,90956881	0,106965465	0,000273971					
cg07263509	0,922162751	0,771914964	-0,150247787	0,000274545	C2orf86	C2orf86;C2orf86;C2orf86	NM_001042692;NM_015910;NM_001042692	5UTR;Body;1stExon	
cg23075144	0,944748688	0,822558607	-0,122190081	0,000276658	SEC1	SEC1	NR_004401	Body	N_Shelf
cg02136596	0,914241666	0,803877018	-0,110364648	0,000277229	ATAD3C	ATAD3C	NM_001039211	TSS200	
cg25450372	0,903334759	0,749368322	-0,153966437	0,000277325					S_Shelf
cg09621695	0,122529939	0,240767708	0,118237769	0,000277686					S_Shelf
cg18065981	0,907467162	0,780340256	-0,127126906	0,000277888	THAP4	THAP4	NM_015963	Body	N_Shore
cg00512404	0,102632104	0,233593322	0,130961218	0,00027952	ARHGDIB	ARHGDIB	NM_001175	TSS200	
cg13502931	0,930277958	0,767210212	-0,163067746	0,000280002	COL6A3	COL6A3;COL6A3;COL6A3	NM_004369;NM_057166;NM_057167	Body;Body;Body	
cg19232748	0,927998843	0,778614799	-0,149384044	0,000280245					
cg02102533	0,731130031	0,837831109	0,106701078	0,000281109	CCDC102A	CCDC102A	NM_033212	Body	Island
cg27440965	0,870329252	0,731127954	-0,139201299	0,000282064	SDC4	SDC4	NM_002999	3UTR	
cg01437204	0,681659353	0,793916292	0,112256939	0,000282585	PTPN7	PTPN7;PTPN7	NM_080588;NM_002832	TSS1500;TSS1500	
cg07160001	0,771679305	0,599230257	-0,172449048	0,000283727					
cg12423733	0,63730274	0,748794748	0,111492007	0,000283911	MAS1L	MAS1L	NM_052967	1stExon	
cg06958766	0,904594485	0,778638484	-0,125956001	0,00028419	TGFB3	TGFB3	NM_003239	Body	N_Shelf
cg20875427	0,932081402	0,82567863	-0,106402771	0,000284592	NEB	NEB;NEB;NEB	NM_004543;NM_001164507;NM_001164508	Body;Body;Body	
cg25744776	0,925627104	0,824019614	-0,10160749	0,000284766	ADARB2	ADARB2	NM_018702	Body	
cg07633853	0,127152575	0,236963652	0,109811078	0,000285249	FKBP5	FKBP5;FKBP5;FKBP5;FKBP5	NM_001145777;NM_004117;NM_001145776;NM_001145775	Body;Body;Body;Body	
cg08229468	0,940703837	0,796271543	-0,144432295	0,000285473	NDRG4	NDRG4;NDRG4;NDRG4	NM_020465;NM_022910;NM_001130487	Body;Body;Body	S_Shelf
cg19755833	0,938371213	0,778447505	-0,159923708	0,000288662	SLIT3	SLIT3	NM_003062	Body	
cg18712231	0,958640762	0,854074532	-0,10456623	0,000291715	SLMAP	SLMAP	NM_007159	Body	
cg25885684	0,671996657	0,788120475	0,116123818	0,000292698					
cg07893689	0,951316587	0,821781841	-0,129534746	0,000293026					
cg07615497	0,145641534	0,314828191	0,169186657	0,000293618					
cg04751358	0,10906003	0,238411932	0,129351902	0,000293661					Island
cg05592996	0,097604574	0,200319057	0,102714482	0,000293764	SMARCC1	SMARCC1	NM_003074	TSS1500	S_Shore
cg27638035	0,751545395	0,865416941	0,113871546	0,000297146	BRUNOL4	BRUNOL4;BRUNOL4;BRUNOL4;BRUNOL4	NM_001025088;NM_020180;NM_001025089;NM_001025087	Body;Body;Body;Body	
cg05217050	0,620820435	0,724003481	0,103183046	0,000298184	DLGAP4	DLGAP4	NM_014902	Body	S_Shore
cg23228887	0,900985474	0,722955699	-0,178029775	0,000298714	ANKAR	ANKAR	NM_144708	Body	S_Shelf
cg15171911	0,075735187	0,177711977	0,10197679	0,0002988	TCF4	TCF4;TCF4	NM_001083962;NM_003199	Body;Body	
cg08708389	0,942698369	0,820858334	-0,121840035	0,00030045					
cg04709062	0,928028824	0,820940116	-0,107088708	0,000300525					
cg02574254	0,728142546	0,846069692	0,117927146	0,000300623	DAB1	DAB1	NM_021080	Body	
cg17384898	0,046791229	0,168577793	0,121786564	0,000301333	KIAA0226	KIAA0226;FYTTD1;KIAA0226;FYTTD1;FYTTD1	NM_001145642;NM_001011537;NM_001145642;NM_032288;N	1stExon;TSS1500;5UTR;TSS	N_Shore
cg04984078	0,954184012	0,838289215	-0,115894797	0,000301423					
cg23588952	0,792795616	0,901868197	0,109072581	0,000301829	MYOM2	MYOM2	NM_003970	Body	
cg05721376	0,805052742	0,609135107	-0,195917634	0,000302476	SH3BGR	SH3BGR;SH3BGR	NM_001001713;NM_007341	5UTR;TSS1500	
cg17961535	0,056437852	0,162533101	0,106095249	0,000302857					
cg17389813	0,072424784	0,210555912	0,138131128	0,000306901	LOC399959	LOC399959	NR_024430	Body	
cg18687753	0,949816636	0,822046177	-0,127770459	0,000307006	BPTF	BPTF;BPTF	NM_182641;NM_004459	Body;Body	
cg03039387	0,083695225	0,193811014	0,110115789	0,000307145					N_Shelf
cg23805594	0,717748308	0,841354559	0,123606252	0,000307299					
cg16244770	0,732214599	0,832536589	0,10032199	0,000307348	MIR130B	MIR130B;MIR301B	NR_029845;NR_030622	TSS1500;TSS200	S_Shore
cg16242750	0,712775146	0,841383805	0,12860866	0,00030804	ARHGAP6	ARHGAP6;ARHGAP6	NM_013423;NM_013427	Body;Body	S_Shelf
cg07146380	0,924901961	0,82365206	-0,101249901	0,000310108	NDUFAF1	NDUFAF1	NM_016013	Body	
cg26355796	0,73123191	0,837116028	0,105884117	0,000310546					S_Shelf
cg05649077	0,105809709	0,2443048	0,138495091	0,000310557					S_Shelf
cg09446250	0,936679828	0,813437287	-0,123242541	0,000311776					
cg05973555	0,623673759	0,768911955	0,145238196	0,000312994					
cg25770698	0,055609779	0,181161077	0,125551298	0,000315819					
cg06584237	0,091449139	0,228114034	0,136664895	0,000316085	CCPG1	CCPG1;CCPG1	NM_020739;NM_004748	TSS1500;TSS1500	S_Shore
cg26125503	0,824888677	0,648293056	-0,17659562	0,000316864	CTAGE5	CTAGE5;CTAGE5;CTAGE5;CTAGE5	NM_203356;NM_203355;NM_005930;NM_203354	Body;Body;Body;Body	
cg06453172	0,715915822	0,837763615	0,121847794	0,000318259	INPP5A	INPP5A	NM_005539	Body	S_Shore
cg07014020	0,099784465	0,200822556	0,101038091	0,000318735	MCC	MCC	NM_001085377	Body	
cg12386568	0,077622216	0,235036394	0,157414178	0,000319738	CA5B	CA5B	NM_007220	TSS200	Island
cg02873541	0,917716053	0,764026757	-0,153689296	0,000320536					
cg24254658	0,88778142	0,774971938	-0,112809482	0,000320971	MAD1L1	MAD1L1;MAD1L1;MAD1L1	NM_003550;NM_001013837;NM_001013836	Body;Body;Body	N_Shore
cg24397939	0,909902552	0,737617233	-0,172285319	0,000321566					
cg03315431	0,74284987	0,843109034	0,100259163	0,000323417	PLXNB3	PLXNB3;PLXNB3	NM_001163257;NM_005393	Body;Body	Island
cg26451261	0,050322451	0,162365427	0,112042975	0,000324084					
cg00311772	0,923758863	0,804530224	-0,119228638	0,000324709	PPP1R7	PPP1R7	NM_002712	Body	S_Shelf
cg22999536	0,937883835	0,830606405	-0,10727743	0,000324756	DIP2C	DIP2C;C10orf108;C10orf108	NM_014974;NR_027151;NR_027152	Body;Body;Body	
cg18590130	0,899116416	0,762075913	-0,137040502	0,000328263	LOC399959	LOC399959;MIRLET7A2	NR_024430;NR_029477	Body;TSS1500	
cg16925210	0,090337061	0,214970016	0,124632955	0,000330374	PECR	PECR;TMEM169;TMEM169;TMEM169;TMEM169	NM_018441;NM_001142311;NM_001142312;NM_138390;NM_0	TSS200;5UTR;5UTR;5UTR;5U	Island
cg25513090	0,722996297	0,385229047	-0,33776725	0,000331906	DAGLB	DAGLB;DAGLB	NM_001142936;NM_139179	TSS1500;TSS1500	S_Shore
cg17473765	0,114790507	0,261633472	0,146842965	0,000332253					N_Shore
cg23979401	0,048835792	0,151361609	0,102525817	0,000333225	LOC728723	LOC728723	NR_024398	Body	
cg17380693	0,933146006	0,743058707	-0,190087299	0,000334808	IGF2BP3	IGF2BP3	NM_006547	Body	
cg17840367	0,689422489	0,805239675	0,115817186	0,000336405					
cg21132420	0,775754112	0,879942675	0,104188563	0,000336819					S_Shore
cg12662887	0,937639933	0,762767184	-0,174872749	0,000338463	NEURL	NEURL	NM_004210	Body	N_Shore
cg23323276	0,246749951	0,141921072	-0,104828879	0,000339918	FAM127B	FAM127B;FAM127B	NM_001078172;NM_001134321	TSS200;TSS200	Island
cg18170100	0,947466045	0,83725421	-0,110211835	0,000340053					
cg05638883	0,937159045	0,78363972	-0,153519325	0,000341334	C1orf97	C1orf97	NR_026761	Body	N_Shore
cg22375763	0,697471903	0,837656879	0,140184975	0,000341788	LRG1	LRG1	NM_052972	1stExon	N_Shelf
cg00982136	0,053407529	0,157122428	0,103714899	0,000342096	CLK1	CLK1;CLK1;CLK1;CLK1	NR_027855;NM_001162407;NM_004071;NR_027856	Body;Body;Body;Body	N_Shelf
cg20945764	0,64645238	0,748089021	0,101636641	0,000344679					N_Shelf
cg15861156	0,581894478	0,701756949	0,119862471	0,000345139					
cg02159654	0,945498627	0,817868202	-0,127630425	0,000346629					
cg24049621	0,945356066	0,74765094	-0,197705126	0,000348498	ZNF532	ZNF532	NM_018181	TSS1500	N_Shore
cg21482290	0,621340867	0,750760247	0,12941938	0,00034991	HRNBP3	HRNBP3	NM_001082575	5UTR	

cg13539397	0,748953148	0,517519468	-0,23143368	0,000350459	RAD51L1	RAD51L1;RAD51L1;RAD51L1	NM_133509;NM_002877;NM_133510	Body;Body;Body	
cg25846153	0,822096784	0,664007595	-0,158089189	0,000353675	C3orf31	C3orf31	NM_138807	Body	N_Shelf
cg24213248	0,146470004	0,254982271	0,108512267	0,000354176	AGAP1	AGAP1;AGAP1	NM_014914;NM_001037131	Body;Body	
cg19168192	0,693410238	0,818924756	0,125514518	0,000354977	GPX5	GPX5;GPX5	NM_003996;NM_001509	TSS200;TSS200	
cg07298482	0,914703273	0,771140035	-0,143563238	0,000355433	SUNC1	SUNC1;SUNC1	NM_152782;NM_001030019	TSS200;TSS200	
cg04806571	0,665515587	0,774673008	0,109157421	0,00035621					
cg09552641	0,754963232	0,863847964	0,108884732	0,000356796	LILRB4	LILRB4;LILRB4	NM_006847;NM_001081438	TSS1500;TSS1500	
cg15340644	0,071440543	0,174736329	0,103295786	0,00035715	IER2	IER2;STX10	NM_004907;NM_003765	5UTR;TSS1500	Island
cg13784878	0,943524989	0,795682483	-0,147842506	0,000358019	PHACTR4	PHACTR4	NM_001048183	5UTR	S_Shelf
cg01569875	0,761022821	0,867786314	0,106763494	0,000359293	SYT17	SYT17	NM_016524	Body	N_Shore
cg24921858	0,750649745	0,852274846	0,101625101	0,00035993	BCL2L14	BCL2L14;BCL2L14;BCL2L14;BCL2L14	NM_138723;NM_030766;NM_138723;NM_138722	5UTR;TSS1500;1stExon;TSS	
cg21171115	0,128135025	0,255209338	0,127074313	0,000362218	AKAP2	AKAP2;PALM2-AKAP2;PALM2-AKAP2;AKAP2	NM_001004065;NM_147150;NM_007203;NM_001136562	Body;Body;Body;5UTR	
cg12051819	0,161972305	0,371307121	0,209334816	0,000362947					
cg15689180	0,878532495	0,748377272	-0,130155222	0,000365046					S_Shelf
cg26360197	0,790237986	0,896021323	0,105783337	0,000366225	RPTOR	RPTOR;RPTOR	NM_001163034;NM_020761	Body;Body	S_Shelf
cg15202609	0,800247067	0,638534421	-0,161712646	0,000366654					
cg22138302	0,660350976	0,772468173	0,112117197	0,00036779	ABCG4	ABCG4;ABCG4	NM_001142505;NM_022169	TSS1500;TSS1500	N_Shore
cg04956382	0,537590722	0,65455517	0,116964448	0,00036891	MYOT	MYOT;MYOT;MYOT;MYOT	NM_006790;NM_006790;NM_001135940;NM_001135940	1stExon;5UTR;1stExon;5UTR	
cg07103068	0,925209196	0,792453734	-0,132755462	0,000370063	MEIS1	MEIS1	NM_002398	Body	
cg18040354	0,103261916	0,214913692	0,111651775	0,000370399	TMEM100	TMEM100;TMEM100	NM_018286;NM_001099640	TSS1500;5UTR	
cg06102602	0,07267898	0,175144692	0,102465712	0,000372881					
cg15283212	0,915458386	0,76482613	-0,150632255	0,000373379	C18orf45	C18orf45	NM_032933	Body	N_Shelf
cg00845742	0,857986134	0,686517398	-0,171468736	0,000374745					S_Shore
cg22278087	0,258907273	0,43537667	0,176469397	0,00037578	NEBL	NEBL	NM_213569	Body	Island
cg25564319	0,932325432	0,808254932	-0,1240705	0,000376118	SYNPR	SYNPR;SYNPR	NM_144642;NM_001130003	Body;Body	
cg01318188	0,316715827	0,441540968	0,124825142	0,000377185	STAG2	STAG2;STAG2;STAG2;STAG2;STAG2	NM_001042749;NM_001042751;NM_001042749;NM_006603;N	1stExon;TSS1500;5UTR;TSS	S_Shore
cg16255729	0,78397311	0,589295557	-0,194677553	0,000378771	RIN3	RIN3	NM_024832	Body	Island
cg20151617	0,9213356	0,809990508	-0,111345092	0,00037902	SLAMF6	SLAMF6	NM_052931	Body	
cg24119500	0,728011471	0,847794717	0,119783245	0,000379337	BAI3	BAI3	NM_001704	Body	
cg24599951	0,07118437	0,19010886	0,118924491	0,000380043	DYRK3	DYRK3;DYRK3	NM_001004023;NM_003582	TSS1500;TSS1500	N_Shore
cg13226861	0,914995641	0,811310053	-0,103685587	0,00038034					
cg17533142	0,935456033	0,745566272	-0,18988976	0,000380672	SUCLA2	SUCLA2	NM_003850	Body	N_Shelf
cg10308950	0,819413879	0,628085887	-0,191327992	0,000381401	TTC7B	TTC7B	NM_001010854	Body	
cg15824062	0,947848791	0,818676493	-0,129172298	0,000381591	GPHN	GPHN;GPHN	NM_020806;NM_001024218	TSS1500;TSS1500	N_Shore
cg15744108	0,941056084	0,831167445	-0,10988864	0,000384208					N_Shelf
cg16147794	0,952567109	0,803550823	-0,149016286	0,000385178					N_Shelf
cg19656425	0,891653476	0,764645526	-0,12700795	0,000385558					
cg18173184	0,152802007	0,267144158	0,11434215	0,00038846					S_Shore
cg01191157	0,774525027	0,898466356	0,123941329	0,000388644					
cg07855449	0,956729482	0,848265437	-0,108464044	0,000390135	WWC2	WWC2	NM_024949	Body	
cg03145322	0,702798512	0,805310672	0,102512159	0,000390169	T-SP1	T-SP1	NM_198464	Body	
cg04252710	0,862281167	0,71371456	-0,148566607	0,000392761	ACTN1	ACTN1;ACTN1;ACTN1	NM_001130004;NM_001102;NM_001130005	Body;Body;Body	
cg21297314	0,974970882	0,856052584	-0,118918299	0,000394132	HLA-E	HLA-E	NM_005516	TSS1500	N_Shore
cg04600055	0,120999756	0,237489622	0,116489866	0,000397585					Island
cg26700469	0,866566921	0,740111943	-0,126454977	0,000398271	WWOX	WWOX	NM_016373	Body	
cg01183860	0,909795639	0,777253355	-0,132542284	0,000398332					
cg06638935	0,773659223	0,877468616	0,103809393	0,000398701					
cg02668694	0,108538913	0,248294301	0,139755388	0,000399172	NLK	NLK	NM_016231	TSS200	S_Shore
cg25874782	0,073406813	0,18166365	0,108256838	0,000400528	NSMAF	NSMAF;NSMAF	NM_003580;NM_001144772	Body;Body	
cg13604623	0,058350854	0,184873195	0,126522341	0,00040069	USP28	USP28	NM_020886	Body	N_Shore
cg14991562	0,94336528	0,823706995	-0,119658286	0,000401448	CYTH3	CYTH3	NM_004227	Body	
cg04699227	0,834576596	0,670061974	-0,164514622	0,000402085	CD44	CD44;CD44;CD44;CD44;CD44	NM_001001389;NM_001001390;NM_000610;NM_001001391;N	3UTR;3UTR;3UTR;3UTR;3UTR	
cg15359342	0,958560016	0,84764943	-0,110910586	0,000403155	SCARNA9L	SCARNA9L;EIF1AX	NR_023358;NM_001412	TSS200;Body	
cg00774300	0,939811585	0,760635129	-0,179176456	0,000403698	TSFM	TSFM	NM_005726	3UTR	
cg13355032	0,898981387	0,722309119	-0,176672268	0,000404734	TSPAN8	TSPAN8	NM_004616	Body	
cg03850198	0,941700341	0,835400116	-0,106300226	0,000406075					
cg02543101	0,933086649	0,789651179	-0,14343547	0,000406631	PSMD7	PSMD7	NM_002811	Body	S_Shelf
cg02035061	0,892193794	0,718238061	-0,173955732	0,00040726					S_Shelf
cg07564108	0,110215961	0,222519025	0,112303065	0,000408666	CDKAL1	CDKAL1	NM_017774	TSS200	
cg18303847	0,876293722	0,745498286	-0,130795436	0,000408731	RNASEN	RNASEN;RNASEN	NM_013235;NM_001100412	Body;Body	
cg20485144	0,16041675	0,285735088	0,125318338	0,0004091	ID3	ID3	NM_002167	TSS200	S_Shore
cg10735211	0,62672034	0,757142058	0,130421718	0,000409288	ZNF509	ZNF509	NM_145291	Body	
cg17107626	0,930854314	0,816948135	-0,113906179	0,000409544	DLG4	DLG4;DLG4	NM_001365;NM_001128827	Body;Body	N_Shelf
cg05512157	0,908343037	0,799053602	-0,109289436	0,000412113	DIP2B	DIP2B	NM_173602	Body	S_Shelf
cg03547797	0,695724584	0,809967007	0,114242423	0,000412484	GAS2	GAS2;GAS2	NM_177553;NM_001143830	TSS200;5UTR	S_Shore
cg08667786	0,723929914	0,831321807	0,107391894	0,0004129	USP7	USP7	NM_003470	Body	
cg25946965	0,923109094	0,744842385	-0,178266709	0,000414218					
cg16799737	0,107972173	0,220092482	0,112120309	0,000415553	HMBOX1	HMBOX1;HMBOX1	NM_001135726;NM_024567	5UTR;5UTR	
cg02095937	0,062732416	0,187665951	0,124933535	0,000415616	TRIM59	TRIM59	NM_173084	5UTR	N_Shore
cg10000764	0,154506246	0,309971493	0,155465247	0,000416507	UBE2H	UBE2H;UBE2H	NM_003344;NM_182697	Body;Body	
cg08626382	0,930244411	0,806979249	-0,123265162	0,000418554	ASRGL1	ASRGL1;ASRGL1	NM_001083926;NM_025080	Body;Body	S_Shelf
cg16524049	0,249308763	0,429642212	0,180333449	0,00041887	LMX1A	LMX1A	NM_177398	Body	Island
cg19992375	0,921984472	0,787060175	-0,134924297	0,00042074	RASSF3	RASSF3	NM_178169	Body	
cg20073882	0,916284588	0,809311629	-0,106972959	0,000422669	NAA11	NAA11	NM_032693	TSS1500	
cg11471799	0,08754546	0,216920559	0,129375099	0,000426123	DIXDC1	DIXDC1	NM_001037954	TSS1500	N_Shore
cg11718162	0,101156304	0,220269639	0,119113335	0,000429244	TPM3	TPM3;TPM3;NUP210L;TPM3;TPM3;NUP210L	NM_001043353;NM_153649;NM_001159484;NM_001043351;N	3UTR;3UTR;TSS1500;3UTR;3	
cg13997788	0,844035405	0,713537735	-0,13049767	0,000430331	EPAS1	EPAS1	NM_001430	Body	
cg07274490	0,785464385	0,892921419	0,107457034	0,000430549	GPR143	GPR143	NM_000273	Body	N_Shelf
cg08123701	0,644487215	0,761055292	0,116568078	0,000430636	DAB1	DAB1	NM_021080	5UTR	
cg02672530	0,050002961	0,17923058	0,129227619	0,00043143					Island
cg08370787	0,884264602	0,7349081	-0,149356503	0,000431679	ADRB2	ADRB2;ADRB2	NM_000024;NM_000024	1stExon;3UTR	S_Shore
cg09954723	0,121770838	0,239668367	0,117897529	0,000432484	ZKSCAN3	ZKSCAN3;ZNF323;ZKSCAN3;ZNF323	NM_024493;NM_001135215;NM_024493;NM_145909	1stExon;5UTR;5UTR;5UTR	
cg24608458	0,920339516	0,788864161	-0,131475355	0,000433062	PINK1	PINK1	NM_032409	TSS1500	N_Shore
cg11298535	0,944435931	0,835070208	-0,109365723	0,000433508	SMOC2	SMOC2;SMOC2	NM_022138;NM_001166412	Body;Body	S_Shore
cg26603220	0,70598291	0,515803383	-0,190179527	0,000433867	WIPI1	WIPI1	NM_017983	3UTR	
cg22715398	0,915402717	0,771540276	-0,143862441	0,000436754	KIAA1370	KIAA1370	NM_019600	Body	N_Shelf
cg08769212	0,922178945	0,785303313	-0,136875632	0,000438103	TMEM175	TMEM175	NM_032326	5UTR	S_Shelf
cg25224232	0,741242861	0,84508235	0,103839489	0,000439218					
cg05878952	0,937515373	0,774601251	-0,162914123	0,000439462					
cg27471156	0,947169486	0,840610626	-0,10655886	0,000444026	MAP4K4	MAP4K4;MAP4K4;MAP4K4	NM_145687;NM_004834;NM_145686	Body;Body;Body	
cg09272225	0,161230447	0,278829592	0,117599145	0,000446209	NPAS2	NPAS2	NM_002518	5UTR	
cg26526443	0,910038833	0,688446577	-0,221592256	0,00044866					N_Shelf
cg00469814	0,164790537	0,292709798	0,127919262	0,000452019					
cg13915576	0,710090325	0,812907056	0,102816731	0,000452511	PAFAH1B3	PAFAH1B3;PRR19;PAFAH1B3;PAFAH1B3	NM_002573;NM_199285;NM_001145939;NM_001145940	TSS1500;5UTR;TSS1500;TSS	S_Shore
cg22995255	0,904680111	0,778140465	-0,126539646	0,000453069					
cg06382526	0,045665096	0,178582531	0,132917435	0,000453109					
cg01580574	0,714034527	0,47458937	-0,239445157	0,000454146					Island
cg15462372	0,919845399	0,74902408	-0,170821319	0,000457324					
cg23935255	0,888032118	0,751081024	-0,136951094	0,000458917	MIR758	MIR758;MIR329-2;MIR329-1	NR_030406;NR_029968;NR_029967	TSS200;TSS1500;TSS1500	
cg03828603	0,088387605	0,195464918	0,107077313	0,000461045	DCAKD	DCAKD;DCAKD	NM_024819;NM_001128631	5UTR;5UTR	N_Shore
cg04026763	0,688702608	0,794028881	0,105326273	0,000461973					
cg00360840	0,914431617	0,798977203	-0,115454414	0,000462893	C7orf38	C7orf38	NM_145111	3UTR	S_Shelf
cg15246270	0,749325629	0,615695805	-0,133629824	0,000463351	LOC100132963	LOC100132963	NM_001162936	TSS1500	
cg14404298	0,715688	0,819319955	0,103631955	0,000466907	CYP8B1	CYP8B1	NM_004391	TSS1500	N_Shelf
cg23540518	0,087573535	0,201105319	0,113531784	0,000467457	TRIM17	TRIM17;TRIM17;TRIM17;TRIM17;TRIM17;TRIM17;TRIM17;TRIM17	NM_001024940;NM_016102;NM_001024941;NM_016102;NM_0	1stExon;5UTR;1stExon;1stEx	Island
cg10556222	0,234004612	0,39156345	0,157558838	0,000470942	HHAT	HHAT;HHAT;HHAT;HHAT;HHAT;HHAT	NM_001170580;NM_001170588;NM_018194;NM_001122834;N	Body;Body;Body;Body;Body;B	
cg25319656	0,819826868	0,670609124	-0,149217744	0,000471411	PTPRN2	PTPRN2;PTPRN2;PTPRN2	NM_002847;NM_130842;NM_130843	Body;Body;Body	N_Shelf
cg23759053	0,065203147	0,180053395	0,114850248	0,00047149	BMPER	BMPER	NM_133468	Body	
cg22688969	0,937367303	0,733666263	-0,20370104	0,000474045	SC5DL	SC5DL;SC5DL	NM_001024956;NM_006918	Body;Body	
cg18767244	0,928403039	0,810799116	-0,117603923	0,000474526					
cg01367424	0,146456093	0,253313024	0,106856931	0,000476786					Island
cg15761531	0,941496335	0,820570632	-0,120925703	0,000480364	GLT8D1	GLT8D1;GLT8D1;GLT8D1;GLT8D1	NM_001010983;NM_152932;NM_018446;NM_152932	5UTR;1stExon;5UTR;5UTR	N_Shore
cg13603589	0,825453519	0,714882452	-0,110571067	0,000481634	ADAMTS16	ADAMTS16	NM_139056	TSS1500	N_Shore
cg19504782	0,954314951	0,825479648	-0,128835303	0,000481908					S_Shore
cg11028872	0,872497207	0,731794034	-0,140703173	0,000482128					
cg17095731	0,602047463	0,733615167	0,131567704	0,000483873	LRP8	LRP8;LRP8;LRP8;LRP8	NM_033300;NM_017522;NM_001018054;NM_004631	TSS1500;TSS1500;TSS1500	S_Shore
cg03612519	0,916559834	0,806005709	-0,110554125	0,000484294	VGLL4	VGLL4;VGLL4	NM_014667;NM_001128219	Body;TSS1500	S_Shore
cg02421824	0,920697342	0,766414133	-0,154283209	0,00048463					
cg01229787	0,756845033	0,86257322	0,105728188	0,000485521					
cg26644303	0,971402204	0,86766294	-0,103739264	0,000488595					S_Shelf
cg25808094	0,946385173	0,784626922	-0,161758251	0,000488679	TBC1D4	TBC1D4	NM_014832	Body	
cg17584490	0,939196842	0,82912213	-0,110074711	0,000491548	RUNDC1	RUNDC1	NM_173079	3UTR	
cg23655155	0,089712872	0,244320857	0,154607985	0,000493071	POLR1A	POLR1A;PTCD3	NM_015425;NM_017952	Body;TSS1500	Island
cg26282761	0,773267834	0,880084525	0,106816691	0,000494729	C10orf90	C10orf90	NM_001004298	Body	N_Shore
cg05959989	0,079915678	0,202512173	0,122596495	0,000494904	MTL5	MTL5;MTL5	NM_001039656;NM_004923	Body;Body	N_Shore
cg14269918	0,927212964	0,783385948	-0,143827016	0,000496358					
cg02224314	0,624444512	0,743015558	0,118571046	0,000496589	BCL11B	BCL11B;BCL11B	NM_022898;NM_138576	Body;Body	Island
cg08939481	0,108534453	0,231537696	0,123003243	0,000496774	NDRG2	NDRG2;NDRG2;NDRG2;NDRG2;NDRG2;NDRG2;NDRG2	NM_201539;NM_201535;NM_201538;NM_201541;NM_201540;N	TSS1500;TSS1500;TSS1500	Island
cg01644285	0,962988131	0,848646392	-0,114341739	0,000497408					
cg16928526	0,844176196	0,682705297	-0,161470899	0,000497692					Island
cg20662016	0,960575047	0,845064559	-0,115510489	0,000498553	DTWD2	DTWD2	NM_173666	Body	N_Shelf
cg26792295	0,939305088	0,820700237	-0,118604851	0,000499329	RFTN1	RFTN1	NM_015150	5UTR	
cg10647739	0,762741979	0,863897269	0,101155291	0,000502136	SORCS2	SORCS2	NM_020777	Body	
cg15129307	0,053903346	0,169790965	0,115887619	0,000504862	KCNC3	KCNC3	NM_004977	TSS200	N_Shore
cg11935592	0,842069658	0,70910136	-0,132968298	0,000506988	CWH43	CWH43	NM_025087	TSS1500	N_Shore
cg19587000	0,8306784	0,706222598	-0,124455803	0,000507586	TSNARE1	TSNARE1	NM_145003	Body	N_Shore
cg03861798	0,886717022	0,736614823	-0,150102199	0,000507974	BRI3BP	BRI3BP	NM_080626	Body	N_Shelf
cg10897631	0,946859952	0,768807468	-0,178052484	0,000511083					S_Shore
cg00308458	0,693124961	0,803293779	0,110168817	0,000513042					
cg21467930	0,844539548	0,728316639	-0,116222909	0,000514225	TBCD	TBCD	NM_005993	Body	
cg11862959	0,075839288	0,208679734	0,132840446	0,000514471	RDH10	RDH10;RPL7	NM_172037;NM_000971	TSS1500;TSS200	N_Shore
cg23668285	0,158096565	0,28636744	0,128270874	0,000514512	SOX1	SOX1;SOX1	NM_005986;NM_005986	1stExon;3UTR	S_Shore
cg01333242	0,673275231	0,808229276	0,134954045	0,000515981					
cg23939844	0,711450774	0,817253053	0,10580228	0,000515994	LRTM2	LRTM2;LRTM2;CACNA2D4;LRTM2	NM_001039029;NM_001163925;NM_172364;NM_001163926	5UTR;5UTR;Body;5UTR	
cg05872835	0,85228352	0,669065566	-0,183217954	0,000516006	COL12A1	COL12A1;COL12A1	NM_080645;NM_004370	3UTR;3UTR	S_Shore
cg06025906	0,72149759	0,836386328	0,114888738	0,00051893	LOC100128023	LOC100128023	NR_027764	TSS200	
cg12560907	0,72212864	0,829497957	0,107369317	0,000522574					
cg15147534	0,918336487	0,782153482	-0,136183004	0,000528871					
cg14515381	0,937781929	0,836436272	-0,101345656	0,00052935	VCP	VCP	NM_007126	TSS1500	S_Shore
cg00579531	0,648926834	0,763562276	0,114635442	0,000529377	DLGAP2	DLGAP2	NM_004745	5UTR	Island
cg15612186	0,121474685	0,227483481	0,106008795	0,000531096	CREG1	CREG1	NM_003851	TSS200	Island
cg14526579	0,786620404	0,610376584	-0,17624382	0,000531252	HELZ	HELZ	NM_014877	5UTR	N_Shelf
cg04867253	0,722764589	0,597258289	-0,1255063	0,00053205	ZMYM3	ZMYM3;ZMYM3;BCYRN1;ZMYM3;ZMYM3	NM_001171162;NM_201599;NR_001568;NM_001171163;NM_0	Body;Body;Body;Body;Body	N_Shore
cg08903631	0,279666316	0,390949213	0,111282897	0,000532568	CD300LG	CD300LG;CD300LG;CD300LG;CD300LG	NM_145273;NM_001168322;NM_001168324;NM_001168323	TSS200;TSS200;TSS200;TSS	
cg22965079	0,099941054	0,209169687	0,109228633	0,000536412	ZNF333	ZNF333	NM_032433	TSS1500	N_Shore
cg21585438	0,123351634	0,227554642	0,104203009	0,000537695					Island
cg00572889	0,87110202	0,678034438	-0,193067582	0,000537919	PGBD5	PGBD5	NM_024554	Body	S_Shore
cg05182393	0,956508233	0,845731525	-0,110776708	0,000538581	ADRB3	ADRB3	NM_000025	Body	N_Shore
cg13166532	0,907213978	0,776762204	-0,130451774	0,000538804					
cg05624487	0,075548768	0,190436509	0,114887741	0,000541207	MAPK14	MAPK14;MAPK14;MAPK14;MAPK14	NM_139014;NM_139013;NM_139012;NM_001315	Body;Body;Body;Body	S_Shore
cg18822243	0,18479657	0,338518593	0,153722023	0,000544049	SOX5	SOX5	NM_152989	5UTR	
cg04123373	0,091266364	0,207612318	0,116345954	0,00054556	IPO13	IPO13	NM_014652	TSS1500	N_Shore
cg23313885	0,686412349	0,531063753	-0,155348596	0,000545753	TMEM71	TMEM71;TMEM71	NM_001145153;NM_144649	TSS1500;TSS1500	
cg08019151	0,891279055	0,712172913	-0,179106141	0,000546345	SCN10A	SCN10A	NM_006514	TSS1500	
cg09931909	0,929605734	0,824874065	-0,104731669	0,00054678	C6orf150	C6orf150	NM_138441	Body	N_Shore
cg12990174	0,941761921	0,763284195	-0,178477726	0,000547888	KRAS	KRAS;KRAS	NM_004985;NM_033360	3UTR;3UTR	
cg21847036	0,111878923	0,223505636	0,111626713	0,00054894	SHOX2	SHOX2;SHOX2;SHOX2	NM_001163678;NM_006884;NM_003030	Body;Body;Body	S_Shore
cg25887394	0,668432141	0,777518783	0,109086643	0,000549031					
cg03821311	0,936317835	0,790333383	-0,145984452	0,000549255	HIST1H1B	HIST1H1B	NM_005322	TSS1500	S_Shore
cg21182616	0,895302087	0,77913162	-0,116170466	0,000549408	KIAA1530	KIAA1530	NM_020894	Body	N_Shore
cg22468751	0,194267366	0,315390169	0,121122803	0,0005502	MAP7	MAP7	NM_003980	Body	
cg22807241	0,7620782	0,57392997	-0,18814823	0,000552041	MIR548F3	MIR548F3;CNTNAP2	NR_031644;NM_014141	Body;Body	
cg12240358	0,918572468	0,805707988	-0,11286448	0,000554938	HOMER2	HOMER2;HOMER2;HOMER2;HOMER2	NM_199330;NM_004839;NM_199332;NM_199331	Body;Body;Body;Body	N_Shore
cg21794066	0,586204045	0,697557315	0,111353269	0,000555295					
cg00416409	0,910478372	0,781949611	-0,128528761	0,000556097	EBF3	EBF3	NM_001005463	Body	N_Shelf
cg22004443	0,128338897	0,255465754	0,127126858	0,000556238	PDIA6	PDIA6	NM_005742	TSS1500	Island
cg08159406	0,878718592	0,771892499	-0,106826094	0,000560672					
cg20511277	0,924874785	0,816471092	-0,108403693	0,000561411					N_Shore
cg22376827	0,115516119	0,25450819	0,138992071	0,000562043	MSL2	MSL2;MSL2	NM_001145417;NM_018133	TSS200;Body	N_Shore
cg10734240	0,090379341	0,231681224	0,141301883	0,000563089	ZNF790	ZNF790	NM_206894	5UTR	Island
cg05035470	0,921230639	0,723163768	-0,198066871	0,000565913					

cg00223245	0,16447761	0,269280772	0,104803162	0,000567636					
cg26227465	0,786187031	0,894518422	0,108331391	0,000568946	IFNG	IFNG	NM_000619	TSS200	
cg19990476	0,899526111	0,768699293	-0,130826818	0,000569347	DIP2C	DIP2C	NM_014974	Body	N_Shore
cg15558299	0,094842649	0,212121714	0,117279065	0,000569806	ELK4	ELK4;ELK4	NM_021795;NM_001973	5UTR;5UTR	N_Shore
cg10558233	0,056104545	0,174417755	0,11831321	0,000573844					
cg14974147	0,79319939	0,553136207	-0,240063183	0,000577679	FBXO40	FBXO40	NM_016298	TSS1500	
cg18527751	0,956145233	0,834555013	-0,12159022	0,00057912	C18orf25	C18orf25;C18orf25	NM_001008239;NM_145055	5UTR;5UTR	S_Shore
cg16534579	0,915034483	0,811024022	-0,104010461	0,000579129	PARK2	PARK2;PARK2;PARK2	NM_004562;NM_013987;NM_013988	Body;Body;Body	N_Shelf
cg08678397	0,69370882	0,805949432	0,112240613	0,000581337					N_Shore
cg03495768	0,165322682	0,283094146	0,117771464	0,000581817	ZIC2	ZIC2	NM_007129	Body	Island
cg05380003	0,727976953	0,849897331	0,121920378	0,000582731	CLTB	CLTB;CLTB	NM_007097;NM_001834	Body;Body	N_Shore
cg06876040	0,944278911	0,801869249	-0,142409662	0,000583288	UNKL	UNKL	NM_001037125	Body	N_Shore
cg13617280	0,898919121	0,791035794	-0,107883327	0,000585675	SLC15A4	SLC15A4;MGC16384	NM_145648;NR_026666	Body;TSS200	
cg05115791	0,868998963	0,589637019	-0,279361944	0,000588338					
cg17644557	0,108520593	0,237644582	0,129123989	0,000588467	NT5E	NT5E	NM_002526	TSS200	N_Shore
cg22692843	0,871283512	0,653791772	-0,21749174	0,000591681	ZNF828	ZNF828;ZNF828;ZNF828	NM_032436;NM_001164144;NM_001164145	TSS1500;TSS1500;TSS1500	N_Shore
cg03950253	0,064326992	0,181884486	0,117557494	0,00059322	BPGM	BPGM;BPGM	NM_001724;NM_199186	5UTR;5UTR	
cg01147055	0,545130018	0,669628061	0,124498043	0,000593635	RASA3	RASA3	NM_007368	Body	N_Shore
cg04786391	0,089816486	0,218007323	0,128190837	0,000594322					
cg04294990	0,071091974	0,185021043	0,113929069	0,000594609	USP22	USP22	NM_015276	Body	N_Shore
cg14555264	0,938391867	0,822479069	-0,115912797	0,000594683					S_Shelf
cg07577018	0,907447353	0,779522061	-0,127925291	0,000594849	ZNF692	ZNF692;ZNF692	NM_017865;NM_001136036	Body;Body	S_Shelf
cg11353598	0,603013068	0,703938985	0,100925917	0,000595932					
cg19390995	0,971484528	0,751430198	-0,22005433	0,000596994					
cg17013778	0,910616546	0,752622695	-0,157993851	0,000597478					N_Shore
cg05655647	0,322510657	0,509890379	0,187379721	0,000598081	ATF1	ATF1	NM_005171	TSS1500	N_Shore
cg10994639	0,103157189	0,237579225	0,134422037	0,000600676	ADCY10	ADCY10;ADCY10	NM_001167749;NM_018417	Body;Body	Island
cg10499429	0,492588175	0,728837839	0,236249665	0,000600722	SMG1	SMG1	NM_015092	3UTR	S_Shelf
cg21331362	0,917646278	0,784585414	-0,133060864	0,0006013					S_Shelf
cg23523811	0,935622757	0,811478061	-0,124144695	0,000608224	GUCY2C	GUCY2C	NM_004963	Body	
cg24367957	0,087137077	0,216949106	0,129812029	0,000608739					
cg02729747	0,919764691	0,800210936	-0,119553755	0,000608869					S_Shelf
cg05525499	0,050724567	0,175948442	0,125223876	0,000609578	HNRNPF	HNRNPF;HNRNPF;HNRNPF;HNRNPF;HNRNPF;HNRNPF	NM_001098205;NM_001098207;NM_001098208;NM_00109820	5UTR;TSS1500;TSS1500;5U	S_Shore
cg21823918	0,898521021	0,726010343	-0,172510678	0,00060999					
cg03929575	0,930993558	0,786202048	-0,14479151	0,000611007	TMTC1	TMTC1	NM_175861	Body	
cg14298924	0,957951624	0,813024946	-0,144926678	0,000611897					N_Shelf
cg27506609	0,908082695	0,797703182	-0,110379514	0,000613262	TEKT2	TEKT2	NM_014466	TSS1500	N_Shore
cg21813470	0,059571576	0,187033495	0,127461919	0,00061418					
cg03529514	0,939071401	0,838794154	-0,100277247	0,000620718	PPM1L	PPM1L	NM_139245	Body	
cg20003124	0,911346714	0,734679417	-0,176667297	0,000622406					S_Shelf
cg00982952	0,943515606	0,823595171	-0,119920436	0,000624009	GLG1	GLG1;GLG1;GLG1;GLG1;GLG1	NR_027264;NM_001145666;NM_001145667;NR_027265;NM_01	Body;Body;Body;Body;Body	N_Shelf
cg19743254	0,048309069	0,152969215	0,104660146	0,000627355	OPCML	OPCML;OPCML	NM_002545;NM_001012393	Body;Body	
cg14591511	0,13512769	0,299548414	0,164420724	0,000628207					
cg24191463	0,072650372	0,177753907	0,105103534	0,000630648	OTX2OS1	OTX2OS1	NR_029385	Body	N_Shore
cg05584737	0,933495706	0,778122322	-0,155373384	0,000632257	LMAN1	LMAN1	NM_005570	3UTR	
cg03298402	0,880060701	0,724115164	-0,155945538	0,000633272	OSTC	OSTC	NM_021227	3UTR	
cg11424312	0,81685597	0,698004881	-0,118851089	0,000633482	KIF26B	KIF26B	NM_018012	Body	
cg16272554	0,948105502	0,806522271	-0,141583231	0,000633986	COLQ	COLQ;COLQ;COLQ	NM_080539;NM_005677;NM_080538	Body;Body;TSS1500	
cg19204946	0,849600675	0,696854511	-0,152746164	0,000635147	FCAMR	FCAMR;FCAMR;FCAMR;FCAMR;FCAMR;FCAMR;FCAMR;FCAMR	NM_001122979;NM_032029;NM_001122980;NM_001170631;N	5UTR;5UTR;1stExon;1stExon;	
cg12927915	0,839777955	0,694707271	-0,145070685	0,000635911	ARMC1	ARMC1	NM_018120	5UTR	N_Shelf
cg08492615	0,089220012	0,215470986	0,126250974	0,000640244	SACM1L	SACM1L	NM_014016	TSS1500	Island
cg08604505	0,892715982	0,693546325	-0,199169657	0,000640378	PVT1	PVT1	NR_003367	Body	
cg05057083	0,913219843	0,772362561	-0,140857282	0,000644106	NUMB	NUMB;NUMB;NUMB;NUMB	NM_001005743;NM_001005745;NM_003744;NM_001005744	3UTR;3UTR;3UTR;3UTR	
cg00773142	0,899243206	0,733904876	-0,16533833	0,000647574	PLCG2	PLCG2	NM_002661	Body	
cg11753124	0,920502777	0,751555203	-0,168947574	0,000649326					
cg11749543	0,931042439	0,821123174	-0,109919265	0,000650511					
cg12226306	0,17759741	0,289920061	0,112322651	0,000650786	ALCAM	ALCAM	NM_001627	Body	N_Shore
cg16373984	0,888105805	0,743688954	-0,144416851	0,000654311	C14orf143	C14orf143	NM_145231	Body	
cg19008877	0,953348838	0,845182026	-0,108166812	0,000654776	C10orf11	C10orf11	NM_032024	Body	
cg05725686	0,94617077	0,8032629	-0,14290787	0,000657971	SETD6	SETD6;SETD6	NM_001160305;NM_024860	3UTR;3UTR	S_Shelf
cg19929409	0,968473136	0,840347687	-0,128125449	0,000658389	PELI2	PELI2	NM_021255	Body	
cg25223634	0,092702346	0,211526343	0,118823997	0,000661335	C10orf26	C10orf26;C10orf26	NM_017787;NM_001083913	Body;Body	
cg11660418	0,934383463	0,832681714	-0,101701749	0,000661619	UXS1	UXS1	NM_025076	Body	
cg22904133	0,137095286	0,240411876	0,10331659	0,000661951	FAM134A	FAM134A;C2orf24	NM_024293;NM_015680	TSS1500;TSS1500	Island
cg26680608	0,118160504	0,226876373	0,108715869	0,000664362	MAP3K5	MAP3K5	NM_005923	Body	
cg18241351	0,7045567	0,8070724	0,1025157	0,000664949	SCGB1D4	SCGB1D4	NM_206998	TSS200	
cg18489945	0,073092647	0,175691052	0,102598405	0,000665702					N_Shore
cg19481355	0,939437271	0,806388699	-0,133048571	0,000665771					N_Shore
cg15485560	0,716141451	0,844538607	0,128397156	0,000667263					N_Shore
cg09792758	0,130817481	0,235578253	0,104760772	0,000667552	COL4A5	COL4A5;COL4A5;COL4A5;COL4A5;COL4A6;COL4A5;COL4A5	NM_033381;NM_033381;NM_000495;NM_033380;NM_001847;N	5UTR;1stExon;1stExon;5UTR;	
cg20779334	0,559546135	0,684089856	0,124543721	0,000668685					S_Shore
cg06794612	0,900621922	0,736071578	-0,164550344	0,000669444					
cg08870042	0,111536569	0,255355202	0,143818633	0,00067129	TMED7-TICAM2	TMED7-TICAM2;TICAM2;TMED7-TICAM2	NM_001164468;NM_021649;NM_001164469	Body;TSS200;Body	Island
cg06792186	0,925890435	0,787291879	-0,138598556	0,000672299	C7orf54	C7orf54;SND1	NR_027330;NM_014390	Body;Body	
cg04767522	0,906604734	0,729555468	-0,177049265	0,000674406	KIF13A	KIF13A;KIF13A;KIF13A;KIF13A	NM_022113;NM_001105567;NM_001105568;NM_001105566	Body;Body;Body;Body	
cg04071430	0,112652433	0,257593503	0,14494107	0,000676171					S_Shelf
cg15557084	0,230667922	0,344725765	0,114057843	0,000678026	SEMA6A	SEMA6A	NM_020796	5UTR	
cg05656360	0,946972582	0,812144119	-0,134828463	0,000678137					N_Shore
cg25757697	0,942228091	0,813463489	-0,128764602	0,000678288	C3AR1	C3AR1	NM_004054	TSS1500	
cg23099839	0,226431024	0,372996482	0,146565458	0,00068154					
cg00589161	0,884807795	0,736309938	-0,148497858	0,000682075	PHACTR3	PHACTR3;PHACTR3;PHACTR3	NM_183246;NM_183244;NM_080672	TSS1500;TSS1500;Body	
cg07604651	0,0589584	0,180471017	0,121512617	0,000682289	FEZF1	FEZF1;FEZF1	NM_001160264;NM_001024613	TSS200;TSS200	N_Shore
cg05232694	0,089469809	0,192449374	0,102979565	0,000683254					S_Shore
cg12451631	0,075359763	0,18703817	0,111678407	0,000685865	RASGEF1A	RASGEF1A	NM_145313	Body	Island
cg03122247	0,906626505	0,800343629	-0,106282876	0,000685922					N_Shore
cg03938353	0,96444465	0,82110094	-0,143343709	0,000687067	LOC100129637	LOC100129637	NR_024488	TSS1500	N_Shore
cg10378538	0,796003352	0,687269896	-0,108733455	0,000687702					
cg04870110	0,663994568	0,473878512	-0,190116056	0,000688678					N_Shelf
cg22116041	0,650492171	0,754671666	0,104179495	0,00069007	TMEM154	TMEM154	NM_152680	Body	
cg13992728	0,896587804	0,785600326	-0,110987478	0,000691511					
cg12905252	0,710677978	0,825737567	0,115059589	0,000692099	PRDM16	PRDM16;PRDM16	NM_022114;NM_199454	Body;Body	S_Shore
cg06996129	0,206367691	0,369937488	0,163569797	0,00069224					Island
cg10662943	0,738849233	0,839943266	0,101094033	0,000692602	LOC100188949	LOC100188949	NR_024464	Body	
cg10155725	0,722467972	0,602715344	-0,119752628	0,000693976	ME3	ME3;ME3;ME3	NM_006680;NM_001161586;NM_001014811	Body;Body;Body	N_Shore
cg03541799	0,78242192	0,675694516	-0,106727404	0,000697751					S_Shelf
cg22715562	0,169972815	0,324102926	0,154130111	0,000698362	ZNF20	ZNF20	NM_021143	TSS200	S_Shore
cg12242338	0,692077694	0,831453328	0,139375634	0,000699595	TRIM42	TRIM42	NM_152616	1stExon	
cg09622285	0,063472291	0,1654513	0,101979009	0,00070297					N_Shelf
cg01987393	0,687767269	0,793839405	0,106072136	0,000708529					
cg08656816	0,711235493	0,811894743	0,10065925	0,000709372	CCL5	CCL5	NM_002985	TSS200	
cg15474378	0,938616649	0,804306882	-0,134309767	0,00071409	FAM111A	FAM111A;FAM111A;FAM111A;FAM111A;FAM111A	NM_001142520;NM_022074;NM_001142521;NM_198847;NM_0	3UTR;3UTR;3UTR;3UTR;3UTR	
cg12766777	0,784705915	0,621806639	-0,162899276	0,000714733					S_Shelf
cg00572089	0,120308118	0,240684511	0,120376393	0,000716382					
cg00238910	0,656700471	0,764441269	0,107740798	0,000719074	LOC100128023	LOC100128023	NR_027764	TSS1500	
cg24916688	0,901650242	0,789077369	-0,112572873	0,000719832					
cg12597325	0,818640071	0,624810795	-0,193829276	0,000721301	STX2	STX2;STX2	NM_001980;NM_194356	Body;Body	
cg11316751	0,917613794	0,783390274	-0,13422352	0,00072211	FGF12	FGF12	NM_004113	Body	N_Shelf
cg07575466	0,629505451	0,740199866	0,110694415	0,000724469	COL9A2	COL9A2	NM_001852	Body	Island
cg13827584	0,669605577	0,781767067	0,112161489	0,000728357					
cg22741626	0,670308294	0,774034632	0,103726338	0,000728629	GNAS	GNAS;GNAS;GNAS;GNAS	NM_001077490;NM_016592;NR_003259;NM_080425	3UTR;3UTR;TSS1500;Body	N_Shore
cg10117171	0,729389708	0,477115	-0,252274708	0,000728862	RHD	RHD;RHD	NM_016124;NM_001127691	Body;Body	
cg24206111	0,207294848	0,355220187	0,147925339	0,000728886					
cg13573178	0,94314217	0,788956653	-0,154185516	0,000728965	ZNF326	ZNF326;ZNF326	NM_182976;NM_182975	Body;Body	S_Shelf
cg15470836	0,04092108	0,200072908	0,159151827	0,000731086					N_Shore
cg04245630	0,859718986	0,673285711	-0,186433275	0,000733092	PPP1R12B	PPP1R12B;PPP1R12B;PPP1R12B;PPP1R12B	NM_001167857;NM_001167858;NM_032105;NM_002481	TSS1500;TSS1500;TSS1500	N_Shore
cg14795750	0,038435444	0,140464155	0,102028711	0,000733394	PCDH10	PCDH10;PCDH10;PCDH10;PCDH10	NM_032961;NM_020815;NM_032961;NM_020815	1stExon;1stExon;5UTR;5UTR	N_Shore
cg24663455	0,859696866	0,677684704	-0,182012162	0,000733709					
cg21106462	0,898767163	0,731342715	-0,167424448	0,000734912	CXCL5	CXCL5	NM_002994	3UTR	N_Shelf
cg12607106	0,886884678	0,71655732	-0,170327358	0,000734958	SMYD3	SMYD3;SMYD3	NM_022743;NM_001167740	Body;Body	
cg20080580	0,901569782	0,732567923	-0,169001859	0,000735369	APBB2	APBB2;APBB2;APBB2	NM_004307;NM_173075;NM_001166050	5UTR;5UTR;5UTR	
cg20454213	0,907782697	0,801090773	-0,106691924	0,000736503					N_Shelf
cg18048953	0,441694252	0,550839978	0,109145726	0,000740856					N_Shelf
cg25888386	0,033202705	0,141334097	0,108131393	0,000742079					
cg04962978	0,914117942	0,75559924	-0,158518702	0,000742135	SALL1	SALL1;SALL1	NM_002968;NM_001127892	3UTR;3UTR	S_Shore
cg18746334	0,912095411	0,797789569	-0,114305842	0,000745336	LSAMP	LSAMP	NM_002338	Body	
cg00325918	0,829119515	0,699078545	-0,13004097	0,000749063	ACOXL	ACOXL;ACOXL;ACOXL	NM_001142807;NM_001142808;NM_001105516	5UTR;5UTR;5UTR	
cg25106036	0,921217065	0,783949599	-0,137267466	0,0007532	HERV-FRD	HERV-FRD;LOC221710	NM_207582;NM_001135575	TSS1500;Body	
cg15132372	0,709187725	0,818003873	0,108816148	0,000759499					Island
cg06422471	0,583526062	0,698085256	0,114559193	0,000761688	SHANK3	SHANK3	NM_001080420	Body	Island
cg04938549	0,152772578	0,274350902	0,121578324	0,000761813	PAX6	PAX6;PAX6;PAX6	NM_001604;NM_001127612;NM_000280	Body;Body;Body	N_Shore
cg08595433	0,705653776	0,839239731	0,133585954	0,00076331					S_Shore
cg02018802	0,155316793	0,321118062	0,165801269	0,000763918	GMPPA	GMPPA;GMPPA	NM_013335;NM_205847	TSS1500;TSS1500	Island
cg03170665	0,710076084	0,446629063	-0,26344702	0,000763955	FAM138B	FAM138B	NR_026821	TSS1500	
cg18957721	0,93595512	0,761388355	-0,174566765	0,000765352	STK24	STK24	NM_001032296	Body	
cg20969923	0,819808121	0,683253303	-0,136554818	0,000765509	NIN	NIN;NIN;NIN	NM_182946;NM_020921;NM_182944	5UTR;5UTR;5UTR	N_Shore
cg24272907	0,889502117	0,786052227	-0,10344989	0,000765918	RIMBP2	RIMBP2	NM_015347	TSS1500	
cg24886033	0,699363096	0,812960197	0,1135971	0,000766337					S_Shore
cg21097090	0,955586701	0,761369626	-0,194217075	0,000767391	TNFAIP8	TNFAIP8;TNFAIP8	NM_014350;NM_001077654	Body;Body	S_Shelf
cg17155499	0,864452076	0,716434451	-0,148017626	0,000767438	NEIL2	NEIL2;NEIL2;NEIL2;NEIL2	NM_145043;NM_001135748;NM_001135747;NM_001135746	3UTR;3UTR;3UTR;3UTR	
cg05273205	0,121449877	0,2336239	0,112174023	0,000769245					N_Shore
cg02896515	0,894146604	0,727369482	-0,166777121	0,000769987	TSPAN1	TSPAN1	NM_005727	5UTR	
cg02241759	0,647339902	0,753484089	0,106144187	0,00077082	NR1I2	NR1I2;NR1I2;NR1I2	NM_033013;NM_003889;NM_022002	Body;Body;Body	N_Shore
cg15681981	0,913446944	0,792809825	-0,120637119	0,000778867					N_Shelf
cg14232552	0,963774794	0,856552548	-0,107222246	0,000779691	SETD3	SETD3;SETD3	NM_032233;NM_199123	Body;Body	
cg11138719	0,863888184	0,71841416	-0,145474024	0,000779802					
cg22681246	0,656623177	0,760829907	0,10420673	0,00078024					
cg03451731	0,821403924	0,619468247	-0,201935676	0,000780858	GP1BA	GP1BA	NM_000173	Body	
cg01314555	0,801433396	0,563438011	-0,237995385	0,000782603	TMPRSS11E	TMPRSS11E	NM_014058	Body	
cg00833639	0,907768248	0,771291779	-0,13647647	0,000784937	C7orf10	C7orf10	NM_024728	Body	
cg14451115	0,9478775	0,819435343	-0,128442157	0,000786137	GABBR2	GABBR2	NM_005458	3UTR	
cg07068358	0,114313313	0,230085487	0,115772175	0,000786218	HS3ST4	HS3ST4	NM_006040	Body	
cg07870003	0,119807835	0,221357223	0,101549388	0,000787097	MTERFD3	MTERFD3;MTERFD3	NM_001033050;NM_025198	TSS200;TSS200	Island
cg00594099	0,721473375	0,831323364	0,109849989	0,000787345	AHDC1	AHDC1	NM_001029882	5UTR	N_Shore
cg01569306	0,882744218	0,755937659	-0,126806559	0,000793137					
cg01340550	0,96582866	0,820540375	-0,145288285	0,000793165	RNF111	RNF111	NM_017610	3UTR	
cg04496824	0,672477164	0,79839948	0,125922315	0,000793209	MTF1	MTF1	NM_005955	3UTR	S_Shelf
cg15886294	0,721475049	0,827745697	0,106270648	0,000793442					
cg15986587	0,91178811	0,7839605	-0,12782761	0,000793978					N_Shelf
cg19147015	0,940087672	0,800499589	-0,139588083	0,000794179	TRIM39	TRIM39;TRIM39	NM_172016;NM_021253	Body;Body	S_Shelf
cg07617265	0,952664687	0,852581834	-0,100082853	0,000796319	GRINL1A	GRINL1A;GCOM1;GRINL1A;GCOM1;GRINL1A	NR_027390;NM_001018090;NM_015532;NM_001018091;NM_0	Body;Body;Body;Body;Body	
cg05071422	0,943908895	0,77878267	-0,165126225	0,000798724					S_Shelf
cg17499729	0,609373651	0,713991181	0,10461753	0,000799161					S_Shore
cg15564871	0,903690002	0,774845794	-0,128844208	0,000800382	PWRN2	PWRN2	NR_026647	Body	
cg27615095	0,675685212	0,782772394	0,107087182	0,000801283	PPM1H	PPM1H	NM_020700	Body	
cg15406952	0,15564042	0,298933454	0,143293034	0,000801656					
cg11559852	0,923446186	0,805228731	-0,118217455	0,000802651	MAP2K1	MAP2K1;SNAPC5	NM_002755;NM_006049	3UTR;3UTR	
cg04244183	0,95973608	0,827742317	-0,131993763	0,00080326					S_Shore
cg02255918	0,909273706	0,804711791	-0,104561916	0,000805757	RBM6	RBM6;RBM6	NM_001167582;NM_005777	5UTR;5UTR	
cg12363682	0,871175958	0,7539999	-0,117176057	0,00080683	GRM6	GRM6	NM_000843	Body	N_Shore
cg01744412	0,068955072	0,216344265	0,147389193	0,000807083					
cg18067134	0,09626838	0,232905883	0,136637503	0,000807344	FOXP1	FOXP1	NM_032682	Body	
cg10006817	0,948144144	0,79268543	-0,155458714	0,000809958	FMN1	FMN1	NM_001103184	Body	
cg02244233	0,909041595	0,782372277	-0,126669319	0,000811318					
cg07317846	0,078333676	0,23281342	0,154479744	0,000813475					
cg17023395	0,947084431	0,796031524	-0,151052908	0,000815179					
cg12229387	0,745387764	0,572591754	-0,17279601	0,000815453	GAST	GAST	NM_000805	TSS1500	
cg16769595	0,085069146	0,21423946	0,129170314	0,000815559	MOBP	MOBP;MOBP	NM_182935;NR_003090	5UTR;Body	N_Shelf
cg15538264	0,911860509	0,772079248	-0,139781261	0,000816234	FBXO27	FBXO27	NM_178820	Body	N_Shelf
cg19474888	0,726461943	0,840308497	0,113846553	0,000817065					
cg02717866	0,719817067	0,825050111	0,105233044	0,000817088	C11orf66	C11orf66;C11orf66	NM_145017;NM_001170753	5UTR;5UTR	
cg17970482	0,874809922	0,766999012	-0,10781091	0,000818138	TGFB2	TGFB2;TGFB2	NM_003238;NM_001135599	Body;Body	

cg16583987	0,709134547	0,814311774	0,105177227	0,000818503					
cg00152838	0,097749808	0,236560024	0,138810216	0,00081931	TNRC6A	TNRC6A	NM_014494	Body	Island
cg21030419	0,112546236	0,246123219	0,133576984	0,000821121	SNX8	SNX8	NM_013321	TSS1500	S_Shore
cg27476456	0,265912332	0,371915238	0,106002906	0,000824554	RFX1	RFX1	NM_002918	5UTR	N_Shore
cg16858146	0,957691824	0,80178577	-0,155906054	0,000825488	TAF3	TAF3	NM_031923	Body	S_Shelf
cg06049774	0,304968149	0,198605867	-0,106362282	0,000827745	PLAC9	PLAC9	NM_001012973	TSS200	N_Shore
cg05652381	0,101819214	0,213310347	0,111491133	0,000831391	TXNDC6	TXNDC6	NM_178130	5UTR	
cg13202952	0,806098602	0,668742843	-0,137355758	0,00083213	NPM2	NPM2	NM_182795	Body	
cg10180697	0,949780765	0,812948839	-0,136831926	0,000832333	DHFR	DHFR;MSH3	NM_000791;NM_002439	Body;TSS1500	N_Shore
cg02002240	0,958769893	0,855718782	-0,10305111	0,000833254	LHFPL2	LHFPL2	NM_005779	5UTR	
cg15770150	0,933297229	0,798154175	-0,135143054	0,000835899					
cg04017131	0,643955069	0,763296974	0,119341904	0,000836942	ANGPT2	ANGPT2;ANGPT2;ANGPT2;MCPH1	NM_001118888;NM_001147;NM_001118887;NM_024596	Body;Body;Body;Body	
cg19684190	0,846422543	0,703219658	-0,143202885	0,000837128					
cg05531309	0,177736296	0,309528078	0,131791781	0,000837295	CNOT4	CNOT4	NM_013316	3UTR	
cg07600669	0,887390676	0,731354066	-0,156036609	0,00083886	ANKRD11	ANKRD11	NM_013275	5UTR	
cg19674611	0,79872898	0,632629503	-0,166099476	0,000840316					S_Shelf
cg09460500	0,895154435	0,77610365	-0,119050785	0,000841365	CAMLG	CAMLG	NM_001745	Body	S_Shelf
cg09260072	0,165491771	0,282513942	0,117022171	0,000841374	SLC44A3	SLC44A3;SLC44A3;SLC44A3	NM_152369;NM_152369;NM_001114106	5UTR;1stExon;Body	Island
cg18040409	0,891728001	0,765171702	-0,126556299	0,000841849	SAPS2	SAPS2	NM_014678	Body	N_Shelf
cg08463929	0,16410027	0,293480972	0,129380702	0,000847063	FBXO5	FBXO5;FBXO5	NM_012177;NM_001142522	TSS1500;TSS1500	S_Shore
cg05658262	0,930930682	0,820201258	-0,110729424	0,000847725	FAM19A5	FAM19A5;FAM19A5	NM_015381;NM_001082967	Body;Body	N_Shelf
cg12640097	0,125926821	0,233240901	0,10731408	0,000847781	RPL7	RPL7	NM_000971	Body	N_Shore
cg21306843	0,630160049	0,732972325	0,102812276	0,000848576	CDX4	CDX4	NM_005193	TSS1500	N_Shore
cg17146029	0,94787462	0,845156033	-0,102718587	0,000850354	AUTS2	AUTS2;AUTS2	NM_015570;NM_001127231	Body;Body	
cg10809832	0,925776871	0,804580211	-0,12119666	0,000850842	SCARNA10	SCARNA10;NCAPD2	NR_004387;NM_014865	TSS1500;Body	
cg21157357	0,939807669	0,810362796	-0,129444873	0,000851421	PCDHGA4	PCDHGA4;PCDHGA12;PCDHGA11;PCDHGA11;PCDHGA9;PCDHGA1;	NM_018917;NM_003735;NM_032092;NM_018914;NM_018921;N	Body;Body;Body;Body;Body;B	N_Shelf
cg19377326	0,938370534	0,763881241	-0,174489293	0,000851793	PUM1	PUM1;PUM1	NM_014676;NM_001020658	Body;Body	
cg11061434	0,713542509	0,833747431	0,120204922	0,000858693	ODZ2	ODZ2	NM_001122679	Body	
cg03533632	0,902364209	0,763191376	-0,139172833	0,000866417	EXD3	EXD3	NM_017820	Body	Island
cg16311685	0,947230829	0,840311806	-0,106919023	0,000871439	MAGOH	MAGOH	NM_002370	Body	N_Shelf
cg12858231	0,936170142	0,81321674	-0,122953402	0,000871515	FANCI	FANCI;FANCI	NM_001113378;NM_018193	Body;Body	
cg02928957	0,951530631	0,821561483	-0,129969149	0,0008717	VEPH1	VEPH1;VEPH1;VEPH1;VEPH1;VEPH1;VEPH1	NM_001167911;NM_024621;NM_001167917;NM_001167915;N	5UTR;5UTR;TSS1500;5UTR;5	
cg20129314	0,756635904	0,882064973	0,125429069	0,000872162	OCA2	OCA2	NM_000275	Body	
cg22218512	0,837449805	0,704167159	-0,133282646	0,000873778	ACVRL1	ACVRL1;ACVRL1	NM_000020;NM_001077401	Body;Body	N_Shore
cg05239225	0,034230488	0,168685019	0,134454531	0,000877508	AKAP13	AKAP13;AKAP13	NM_006738;NM_007200	5UTR;5UTR	
cg16803837	0,678801224	0,521662638	-0,157138586	0,000878588					
cg17929152	0,92899766	0,732301465	-0,196696195	0,000881989	ARID1B	ARID1B;ARID1B;ARID1B	NM_017519;NM_175863;NM_020732	Body;Body;Body	
cg12736773	0,731963407	0,834926604	0,102963196	0,000884401					
cg25494075	0,63301833	0,773087255	0,140068925	0,000884698					
cg26292018	0,912800001	0,750066718	-0,162733282	0,00088649	PHF20L1	PHF20L1;PHF20L1;PHF20L1	NM_198513;NM_032205;NM_016018	TSS1500;TSS1500;TSS1500	N_Shore
cg21819722	0,949462062	0,805667267	-0,143794796	0,000886641	ATP8A1	ATP8A1;ATP8A1	NM_001105529;NM_006095	Body;Body	
cg21022303	0,906814032	0,804959804	-0,101854228	0,000887155					
cg00197006	0,889635636	0,748532391	-0,141103245	0,000891775	COTL1	COTL1	NM_021149	Body	
cg02033582	0,051553749	0,21220293	0,160649182	0,000892924					
cg01818776	0,052834091	0,169734869	0,116900778	0,000893357	DOCK5	DOCK5	NM_024940	Body	
cg02859098	0,8974162	0,778894438	-0,118521762	0,0008934	UBE2J2	UBE2J2;UBE2J2;UBE2J2;UBE2J2	NM_194315;NM_194457;NM_058167;NM_194458	5UTR;5UTR;5UTR;5UTR	Island
cg01627405	0,732311104	0,847228763	0,114917659	0,000895846	MIR1227	MIR1227;PLEKHJ1	NR_031596;NM_018049	TSS1500;Body	N_Shore
cg14501839	0,93050772	0,709509738	-0,220997983	0,000897076					N_Shelf
cg10208367	0,912028007	0,763483379	-0,148544627	0,00089709	C13orf36	C13orf36	NM_203451	5UTR	
cg14373410	0,933161793	0,832719669	-0,100442125	0,000897102	TSHR	TSHR;TSHR;TSHR	NM_001018036;NM_001142626;NM_000369	Body;Body;Body	
cg22094163	0,790669399	0,614805096	-0,175864304	0,000899652	KDM3A	KDM3A;KDM3A	NM_001146688;NM_018433	Body;Body	
cg17682357	0,782883284	0,885398154	0,10251487	0,000902969	KIAA1486	KIAA1486	NM_020864	Body	
cg22331840	0,946906299	0,841242578	-0,105663721	0,000903135	LOC646405	LOC646405	NR_026730	Body	N_Shelf
cg00907218	0,91560711	0,764097216	-0,151509894	0,000903601					N_Shore
cg01075463	0,923978011	0,789623271	-0,13435474	0,000905381	FAM108A1	FAM108A1;FAM108A1	NM_031213;NM_001130111	TSS1500;TSS1500	S_Shore
cg04170238	0,812851588	0,645836526	-0,167015062	0,000906337					
cg02471760	0,74025556	0,876441733	0,136186172	0,000906338	MIR210	MIR210	NR_029623	TSS1500	S_Shore
cg09367425	0,056921356	0,192413802	0,135492447	0,00091089	FTH1	FTH1	NM_002032	Body	N_Shore
cg18062196	0,7345954	0,84613588	0,11154048	0,000911501	DHX40	DHX40;DHX40	NM_024612;NM_001166301	TSS1500;TSS1500	N_Shore
cg04267605	0,917741165	0,786472022	-0,131269143	0,000911955	LRRC66	LRRC66	NM_001024611	3UTR	
cg08112273	0,755006249	0,856012856	0,101006607	0,000914544					
cg02296916	0,13186991	0,254813226	0,122943315	0,000916946	XRCC4	XRCC4;XRCC4;XRCC4;TMEM167A	NM_022550;NM_003401;NM_022406;NM_174909	TSS1500;TSS1500;TSS1500	N_Shore
cg08698685	0,909631807	0,747846367	-0,161785439	0,000917435	SGK1	SGK1	NM_001143676	Body	
cg07446753	0,639534031	0,750876573	0,111342542	0,000918787					
cg08371951	0,84256345	0,696650653	-0,145912797	0,000919894	SYNGAP1	SYNGAP1	NM_006772	Body	S_Shore
cg23244810	0,218618606	0,329095193	0,110476587	0,000921183					
cg23826993	0,679085771	0,533204475	-0,145881297	0,000921366					N_Shelf
cg00367281	0,750536295	0,860854173	0,110317878	0,000921986	CHRNB3	CHRNB3	NM_000749	TSS200	
cg19201144	0,422697694	0,541189584	0,118491891	0,000923039	CYP27C1	CYP27C1	NM_001001665	Body	
cg08446111	0,690165934	0,792180842	0,102014908	0,000927092	LONP2	LONP2	NM_031490	Body	S_Shore
cg02164335	0,786018769	0,666281325	-0,119737444	0,000930821					S_Shore
cg14826404	0,12948599	0,255259909	0,125773919	0,000930855	MGAT1	MGAT1;MGAT1;MGAT1	NM_001114618;NM_001114618;NM_001114617	1stExon;5UTR;5UTR	N_Shore
cg15219811	0,914298749	0,79312146	-0,121177289	0,000931856	ACTR1A	ACTR1A	NM_005736	Body	N_Shelf
cg06604289	0,360167489	0,236451307	-0,123716182	0,00093364	PRDM2	PRDM2;PRDM2;PRDM2	NM_012231;NM_015866;NM_001135610	Body;Body;Body	
cg03396152	0,052879186	0,160772606	0,10789342	0,000934745	NID1	NID1	NM_002508	Body	
cg13687834	0,266684736	0,398170522	0,131485785	0,000935507					
cg25270206	0,94864662	0,823694261	-0,124952358	0,000937153	PAQR5	PAQR5;PAQR5	NM_001104554;NM_017705	3UTR;3UTR	
cg18077436	0,112557826	0,247186727	0,1346289	0,000937836	DDX3Y	DDX3Y;DDX3Y	NM_004660;NM_001122665	TSS200;5UTR	Island
cg24322139	0,926856749	0,82095188	-0,105904869	0,000938373	SAP18	SAP18	NM_005870	3UTR	
cg22436032	0,867168409	0,701919239	-0,16524917	0,000938395					N_Shelf
cg16480634	0,746509091	0,564812653	-0,181696438	0,000940535	ACTR2	ACTR2;ACTR2	NM_001005386;NM_005722	3UTR;3UTR	
cg00200667	0,555549137	0,682795675	0,127246538	0,000940631					N_Shelf
cg05672026	0,124482282	0,279971069	0,155488788	0,000942546					
cg02175952	0,937476361	0,786954076	-0,150522285	0,000942627	RBM33	RBM33	NM_053043	Body	
cg05716821	0,955439443	0,798606538	-0,156832905	0,000943086	FYN	FYN;FYN;FYN	NM_153048;NM_002037;NM_153047	5UTR;5UTR;TSS1500	
cg08066417	0,649289471	0,7836776	0,134388129	0,000943138	CDH5	CDH5	NM_001795	5UTR	
cg12710567	0,07167237	0,193697868	0,122025498	0,000946095	SIRT7	SIRT7	NM_016538	TSS200	Island
cg24435884	0,753971624	0,854357755	0,100386131	0,00094838	C1orf94	C1orf94;C1orf94	NM_032884;NM_001134734	5UTR;TSS1500	N_Shore
cg15689991	0,044326767	0,164190989	0,119864222	0,00094856	RASSF3	RASSF3	NM_178169	Body	
cg05493094	0,239107464	0,3431118	0,104004337	0,000952586	HIST1H3D	HIST1H3D;HIST1H2BF;HIST1H2AD	NM_003530;NM_003522;NM_021065	TSS1500;1stExon;TSS1500	Island
cg22451002	0,856289593	0,733437003	-0,12285259	0,000956598	TKTL1	TKTL1;TEX28;TKTL1	NM_001145933;NM_001586;NM_012253	TSS1500;TSS200;TSS1500	N_Shore
cg27397787	0,105018824	0,269673849	0,164655025	0,000957415	FLJ26850	FLJ26850	NR_027257	TSS200	N_Shore
cg05451413	0,091711681	0,223373559	0,131661878	0,000958426	ROCK1	ROCK1;ROCK1	NM_005406;NM_005406	5UTR;1stExon	Island
cg10111153	0,957162777	0,824924031	-0,132238745	0,000959342	OBFC2B	OBFC2B	NM_024068	Body	S_Shelf
cg15907287	0,796569099	0,664123686	-0,132445413	0,000959749	DOCK2	DOCK2	NM_004946	Body	
cg25408901	0,132650117	0,368135306	0,23548519	0,000960065	ISL1	ISL1	NM_002202	Body	S_Shore
cg25793812	0,923638306	0,733172517	-0,190465789	0,000961132	LOC93622	LOC93622	NR_015433	Body	S_Shore
cg01966791	0,625152442	0,734363294	0,109210853	0,000961758	MIR1914	MIR1914;UCKL1	NR_031735;NM_017859	Body;Body	S_Shore
cg08253824	0,931363427	0,761748382	-0,169615046	0,000962354	SCN8A	SCN8A	NM_014191	Body	
cg13210558	0,937414883	0,727270691	-0,210144192	0,000963572					N_Shore
cg05979170	0,646020598	0,755476151	0,109455553	0,00096488					
cg26357538	0,04505772	0,159720758	0,114663038	0,000965262	RDH10	RDH10;RPL7	NM_172037;NM_000971	TSS1500;TSS200	N_Shore
cg10579818	0,775476804	0,877194594	0,101717789	0,000967043	CHST8	CHST8;CHST8	NM_001127895;NM_001127896	5UTR;5UTR	S_Shelf
cg07710247	0,937004425	0,8361495	-0,100854926	0,000967833	NARS	NARS	NM_004539	3UTR	
cg27542609	0,290161604	0,188837667	-0,101323938	0,000968625					N_Shore
cg08739478	0,682486422	0,801333388	0,118846965	0,000969639					N_Shelf
cg26362852	0,179129736	0,318887856	0,13975812	0,000970812	RNF145	RNF145	NM_144726	TSS200	N_Shore
cg03831912	0,898236162	0,762278927	-0,135957235	0,000971588	ZNF778	ZNF778	NM_182531	Body	
cg12955305	0,926092233	0,789084607	-0,137007626	0,000976637	TMEM196	TMEM196	NM_152774	3UTR	
cg05061754	0,762775863	0,867724995	0,104949133	0,000979346	SDK1	SDK1;SDK1	NR_027816;NM_152744	Body;Body	
cg00911962	0,832344039	0,642160131	-0,190183908	0,000982336	sept-06	SEPT6;SEPT6;SEPT6;SEPT6	NM_015129;NM_145802;NM_145800;NM_145799	Body;Body;Body;Body	
cg10656204	0,621640713	0,735688864	0,11404815	0,000982974					
cg00061792	0,132448915	0,234790247	0,102341331	0,000983697					
cg11115318	0,949584463	0,82184905	-0,127735413	0,000984					N_Shelf
cg13332807	0,701762401	0,80455113	0,102788728	0,000985204					
cg10631544	0,941653122	0,809236064	-0,132417058	0,000990903					N_Shelf
cg11238820	0,701906877	0,821359948	0,119453071	0,000990948	MIXL1	MIXL1	NM_031944	TSS1500	N_Shore
cg12619656	0,915789135	0,735563575	-0,18022556	0,000991231	POLL	POLL	NM_013274	Body	N_Shelf
cg22013019	0,625216132	0,734530473	0,109314342	0,000991913					S_Shelf
cg05981878	0,872914319	0,718451809	-0,15446251	0,000992486					
cg24546683	0,696052991	0,815570083	0,119517092	0,000992583					
cg09154395	0,826347461	0,551712447	-0,274635014	0,000995087					Island
cg18039643	0,045867668	0,14829074	0,102423072	0,00099639	SNORD27	SNORD27;SNORD26;SNORD29;SNORD28;SLC3A2;SLC3A2;SLC3A	NR_002563;NR_002564;NR_002559;NR_002562;NM_002394;N	TSS1500;TSS200;TSS1500;	N_Shore
cg10410170	0,05184235	0,15385795	0,102015601	0,00099844	NBN	NBN	NM_002485	TSS200	S_Shore
cg20679157	0,78192308	0,888171124	0,106248044	0,00099883	KRT82	KRT82	NM_033033	TSS1500	
cg15084160	0,9457194	0,823133789	-0,122585611	0,001000904	ACBD3	ACBD3	NM_022735	Body	N_Shelf
cg14380045	0,6540998	0,781712294	0,127612494	0,001001086					Island
cg03834116	0,104421309	0,211430762	0,107009454	0,001001286					
cg17098965	0,902950132	0,78492403	-0,118026102	0,00100158	ZNF217	ZNF217;ZNF217	NM_006526;NM_006526	5UTR;1stExon	S_Shore
cg09778867	0,565879267	0,66791317	0,102033903	0,001001643					N_Shore
cg04882739	0,91081776	0,793720992	-0,117096768	0,001002995					
cg08931376	0,912478869	0,788358767	-0,124120102	0,001003045					N_Shore
cg19751560	0,927964169	0,826749215	-0,101214954	0,00100329	MAST4	MAST4;MAST4	NM_001164664;NM_198828	Body;Body	S_Shelf
cg01869896	0,726843689	0,830389049	0,10354536	0,001010317	MFSD2A	MFSD2A;MFSD2A	NM_032793;NM_001136493	TSS1500;TSS1500	N_Shore
cg27316798	0,153792346	0,258249517	0,104457172	0,001012512	ANKH	ANKH	NM_054027	Body	N_Shore
cg06219041	0,091073162	0,211649212	0,12057605	0,001015256	ALOXE3	ALOXE3;ALOXE3	NM_001165960;NM_021628	TSS1500;TSS1500	N_Shore
cg11154715	0,945036632	0,749927704	-0,195108929	0,001020061					N_Shore
cg05916707	0,069758834	0,180359457	0,110600623	0,001020344					
cg26056959	0,799362426	0,632155675	-0,167206751	0,001025553	B3GNTL1	B3GNTL1	NM_001009905	Body	
cg04281898	0,735069842	0,605318304	-0,129751538	0,001026496	ASGR2	ASGR2;ASGR2;ASGR2;ASGR2	NM_080912;NM_001181;NM_080914;NM_080913	Body;Body;Body;Body	
cg04791080	0,630962303	0,762583328	0,131621025	0,001031203					
cg00104348	0,869241941	0,769137432	-0,10010451	0,001032549	STYK1	STYK1	NM_018423	3UTR	
cg19997181	0,896073373	0,786902225	-0,109171148	0,001035061					
cg15703322	0,792135113	0,603094105	-0,189041008	0,001035218	LSM3	LSM3	NM_014463	3UTR	
cg27416337	0,94542827	0,82377975	-0,12164852	0,001035887	CNOT10	CNOT10	NM_015442	Body	
cg02567323	0,888239031	0,762964764	-0,125274267	0,001038075	KLRAQ1	KLRAQ1;KLRAQ1;KLRAQ1;KLRAQ1	NR_024188;NM_001135629;NM_001135630;NM_152994	Body;Body;5UTR;Body	S_Shelf
cg07312099	0,07984183	0,205755801	0,125913971	0,001039271	ASAH1	ASAH1;ASAH1;ASAH1	NM_001127505;NM_004315;NM_177924	TSS200;TSS200;TSS1500	S_Shore
cg03538499	0,204979958	0,335235496	0,130255538	0,001040752	FGR	FGR;FGR;FGR	NM_001042729;NM_001042747;NM_005248	Body;Body;Body	N_Shore
cg06900101	0,934097366	0,820105887	-0,113991479	0,001041064	TTC7B	TTC7B	NM_001010854	Body	
cg18159684	0,941533289	0,827812505	-0,113720784	0,001043477	AZIN1	AZIN1;AZIN1	NM_015878;NM_148174	3UTR;3UTR	
cg15313936	0,920561903	0,704069448	-0,216492455	0,001045498	WDR20	WDR20;WDR20;WDR20;WDR20	NM_181302;NM_181291;NM_144574;NM_181308	Body;Body;Body;Body	S_Shore
cg17184165	0,754237982	0,856980614	0,102742632	0,001047181	CHDH	CHDH	NM_018397	Body	N_Shore
cg25713793	0,923037271	0,815492026	-0,107545245	0,001048989	NRP1	NRP1;NRP1;NRP1	NM_001024628;NM_003873;NM_001024629	Body;Body;Body	
cg15438404	0,129092175	0,266226205	0,137134031	0,001051172	MBD6	MBD6	NM_052897	TSS200	Island
cg15108490	0,665837604	0,773957973	0,108120369	0,001051523	LDLRAD3	LDLRAD3	NM_174902	Body	
cg21738441	0,923217016	0,80594894	-0,117268076	0,001051917	ZNF28	ZNF28	NM_006969	3UTR	
cg22456692	0,106248865	0,210181474	0,103932608	0,001056234	WWC1	WWC1;WWC1;WWC1	NM_001161661;NM_001161662;NM_015238	TSS1500;TSS1500;TSS1500	N_Shore
cg03750367	0,918919106	0,764317623	-0,154601483	0,001059472	GGTA1	GGTA1	NR_003191	TSS1500	S_Shore
cg09583036	0,944789814	0,820034576	-0,124755238	0,001059608	RAB38	RAB38	NM_022337	3UTR	
cg25775721	0,09649796	0,221927572	0,125429612	0,001064161	SNHG3	SNHG3;SNHG3-RCC1;SNHG3-RCC1;SNHG3-RCC1	NR_002909;NR_030726;NM_001048199;NR_030725	TSS1500;TSS1500;TSS1500	
cg16164276	0,098984368	0,233676278	0,13469191	0,001065664	SLC6A1	SLC6A1	NM_003042	TSS200	N_Shore
cg00933542	0,732025682	0,832368306	0,100342625	0,001066701	PCMT1	PCMT1	NM_005389	TSS1500	N_Shore
cg25032124	0,893833698	0,671636454	-0,222197243	0,001068183	HBXIP	HBXIP	NM_006402	Body	N_Shelf
cg21803123	0,752775125	0,622801169	-0,129973955	0,00106947					N_Shelf
cg20208979	0,752493529	0,878399884	0,125906356	0,001069886					
cg00624418	0,755436024	0,862853793	0,10741777	0,001069908	TERT	TERT;TERT	NM_198255;NM_198253	Body;Body	N_Shore
cg15255859	0,297770057	0,419940293	0,122170236	0,001071965	FANCB	FANCB;MOSPD2;FANCB;MOSPD2	NM_001018113;NM_152581;NM_152633;NM_152581	TSS1500;5UTR;TSS1500;1st	Island
cg10113157	0,92000653	0,744200629	-0,175805901	0,001075812					N_Shelf
cg14134015	0,94157168	0,832828318	-0,108743361	0,001077134	ZNRD1	ZNRD1;ZNRD1	NM_170783;NM_014596	Body;Body	S_Shelf
cg16845172	0,891899516	0,706267795	-0,185631721	0,001078121					
cg15433043	0,748970454	0,861198778	0,112228324	0,00107877	MYO7A	MYO7A;MYO7A;MYO7A	NM_000260;NM_001127180;NM_001127179	Body;Body;Body	
cg27182510	0,143581594	0,296321751	0,152740157	0,001079259	PTPRK	PTPRK;PTPRK	NM_002844;NM_001135648	Body;Body	
cg12462499	0,953350944	0,847686467	-0,105664476	0,001079349	FNDC3B	FNDC3B;FNDC3B	NM_001135095;NM_022763	Body;Body	
cg05458554	0,876646067	0,686244824	-0,190401243	0,001082567	TMEM64	TMEM64;TMEM64	NM_001008495;NM_001146273	Body;Body	
cg20613259	0,694390629	0,518974845	-0,175415784	0,001083015	NT5C2	NT5C2;NT5C2	NM_001134373;NM_012229	Body;Body	
cg03610852	0,067553303	0,172388018	0,104834716	0,001084943					S_Shore
cg22891161	0,745985755	0,849930376	0,103944622	0,001086004	MUC20	MUC20;MUC20	NM_152673;NM_001098516	Body;Body	S_Shelf
cg14239359	0,940634776	0,80950141	-0,131133366	0,001088773					S_Shelf
cg06062945	0,903073855	0,753141894	-0,149931961	0,001090987	SUPT7L	SUPT7L	NM_014860	Body	N_Shelf
cg20517050	0,047859369	0,171061171	0,123201802	0,001093514	HOXA5	HOXA5	NM_019102	TSS1500	Island
cg27036054	0,925572695	0,766629463	-0,158943233	0,001099799	RIMS1	RIMS1;RIMS1;RIMS1;RIMS1;RIMS1;RIMS1	NM_001168407;NM_014989;NM_001168410;NM_001168408;N	3UTR;3UTR;3UTR;3UTR;3UTR	

cg01166299	0,928079084	0,782379815	-0,145699269	0,001104503	BANP	BANP;BANP	NM_017869;NM_079837	Body;Body	
cg12630147	0,072369216	0,199421614	0,127052398	0,001106966	TET1	TET1	NM_030625	5UTR	S_Shore
cg18376163	0,063106171	0,164322	0,101215829	0,001108754	KIFC2	KIFC2;CYHR1;CYHR1;CYHR1	NM_145754;NM_001129888;NM_138496;NM_032687	TSS1500;TSS200;TSS1500;	Island
cg18751231	0,07733416	0,188029314	0,110695154	0,001113813	SCHIP1	SCHIP1	NM_014575	Body	
cg01874309	0,907225046	0,758698245	-0,148526801	0,001113831	HEATR4	HEATR4	NM_203309	Body	N_Shelf
cg10448052	0,079908808	0,224048294	0,144139486	0,001119371	AAAS	AAAS	NM_015665	TSS1500	S_Shore
cg26452811	0,910217565	0,776185077	-0,134032488	0,001120679	IGDCC3	IGDCC3	NM_004884	Body	N_Shelf
cg14787753	0,921385614	0,786124381	-0,135261233	0,001121044	STK39	STK39	NM_013233	Body	
cg03846960	0,88822617	0,735404243	-0,152821928	0,001123598	JARID2	JARID2	NM_004973	Body	
cg17600863	0,842855143	0,720056502	-0,122798641	0,001123886	LOC100188947	LOC100188947	NR_024467	TSS1500	
cg06501366	0,23892125	0,118839122	-0,120082128	0,001124182	BHMT2	BHMT2;DMGDH	NM_017614;NM_013391	Body;TSS1500	Island
cg03688085	0,847281222	0,666752302	-0,18052892	0,001127579	KIAA1751	KIAA1751	NM_001080484	Body	Island
cg17413302	0,893708127	0,780071265	-0,113636861	0,00112956					
cg26784822	0,073129338	0,17712896	0,103999621	0,00113304	RND3	RND3	NM_005168	Body	N_Shore
cg08197287	0,054876371	0,185562271	0,130685901	0,001133476	AFF1	AFF1;AFF1	NM_001166693;NM_005935	Body;Body	
cg16314254	0,718257532	0,821356402	0,10309887	0,001134269	GALNT2	GALNT2	NM_004481	Body	
cg26275986	0,06332465	0,17397907	0,11065442	0,00113508	FAM110A	FAM110A;FAM110A	NM_207121;NM_001042353	5UTR;5UTR	N_Shelf
cg05985401	0,929757843	0,825162254	-0,10459559	0,001136368					Island
cg16021939	0,905088693	0,795456835	-0,109631858	0,001137545	LOC284379	LOC284379	NR_002938	TSS200	
cg01766699	0,116513791	0,257508455	0,140994664	0,001139588	DNAH7	DNAH7	NM_018897	Body	
cg00054572	0,911863013	0,726000012	-0,185863	0,001139968					S_Shelf
cg05292788	0,946100563	0,807379009	-0,138721554	0,001143244	GABBR1	GABBR1;GABBR1;GABBR1	NM_021904;NM_021903;NM_001470	Body;Body;Body	
cg23549061	0,629104986	0,737533426	0,10842844	0,001143769	PAK3	PAK3;PAK3	NM_001128167;NM_001128166	TSS1500;TSS1500	
cg12835313	0,689607898	0,807437667	0,117829769	0,001145612	CACNA2D3	CACNA2D3	NM_018398	Body	S_Shelf
cg03920152	0,698725433	0,575470771	-0,123254662	0,001146722					N_Shelf
cg16445317	0,945080282	0,79028815	-0,154792132	0,00114681	UNC119B	UNC119B	NM_001080533	TSS200	N_Shore
cg07401745	0,938534614	0,834063971	-0,104470643	0,001151264	NDUFS4	NDUFS4	NM_002495	Body	
cg27456203	0,250830946	0,376891518	0,126060571	0,00115134	WWP2	WWP2;WWP2	NM_199423;NM_007014	Body;Body	
cg05878332	0,884377256	0,768615524	-0,115761731	0,00115146					
cg27480957	0,921558504	0,761456945	-0,160101559	0,001159608	AXIN1	AXIN1;AXIN1	NM_003502;NM_181050	Body;Body	N_Shore
cg05163325	0,960215156	0,804956617	-0,155258539	0,001163856	SNX9	SNX9	NM_016224	Body	
cg05940236	0,068594888	0,23489595	0,166301062	0,001168154	DDX3Y	DDX3Y;DDX3Y	NM_001122665;NM_004660	Body;Body	Island
cg23605670	0,658876885	0,772026991	0,113150107	0,001172712	BLCAP	BLCAP;BLCAP;BLCAP;BLCAP;NNAT;NNAT;BLCAP	NM_001167821;NM_001167820;NM_001167822;NM_006698;N	5UTR;5UTR;5UTR;5UTR;TSS1	Island
cg21498161	0,916440599	0,811371692	-0,105068907	0,001172737	SPEF2	SPEF2	NM_024867	Body	
cg25116020	0,951314663	0,824731	-0,126583663	0,001175624	TRIM25	TRIM25	NM_005082	3UTR	
cg08380440	0,778376705	0,655541236	-0,122835468	0,001175892					
cg26963790	0,205325191	0,312773455	0,107448263	0,001177085	ZMYND11	ZMYND11;ZMYND11;ZMYND11	NM_212479;NM_001161482;NM_006624	TSS1500;TSS1500;TSS1500	N_Shore
cg04737950	0,154848173	0,333028002	0,178179829	0,001178446					S_Shore
cg26928418	0,226476513	0,383118484	0,15664197	0,001184412	ZNF578	ZNF578	NM_001099694	5UTR	S_Shore
cg20130098	0,873647967	0,720452232	-0,153195735	0,001186422	KALRN	KALRN;KALRN	NM_001024660;NM_003947	Body;Body	N_Shelf
cg19540291	0,924751942	0,729900138	-0,194851804	0,001187724					S_Shelf
cg21101465	0,207642854	0,361644904	0,15400205	0,001189636	PDX1	PDX1	NM_000209	TSS1500	N_Shore
cg00127036	0,091689161	0,273037853	0,181348692	0,00118995	ZNF346	ZNF346	NM_012279	TSS1500	N_Shore
cg17156828	0,677825349	0,77880968	0,100984331	0,001193364	HDAC4	HDAC4	NM_006037	Body	
cg14160911	0,808342664	0,651744908	-0,156597756	0,001193483					S_Shelf
cg12404044	0,781086018	0,886759736	0,105673718	0,001194242					N_Shelf
cg16081992	0,938673912	0,698359116	-0,240314796	0,001196832	SON	SON;SON	NM_032195;NM_138927	3UTR;Body	
cg23537560	0,873204708	0,681959589	-0,191245119	0,001199799					
cg01471111	0,716558127	0,833217027	0,116658899	0,001200332	SLC6A13	SLC6A13	NM_016615	TSS1500	
cg14969981	0,097103511	0,231221355	0,134117844	0,001200746	TMCC2	TMCC2	NM_014858	TSS1500	N_Shore
cg02710015	0,827863381	0,723859849	-0,104003532	0,001200789	KIAA0748	KIAA0748;KIAA0748	NM_001098815;NM_001136030	Body;Body	
cg13434757	0,903096377	0,713513579	-0,189582797	0,001206894	SLC22A1	SLC22A1;SLC22A1	NM_003057;NM_153187	TSS1500;TSS1500	
cg09152949	0,91268645	0,77612368	-0,13656277	0,001213117	TBCD	TBCD	NM_005993	Body	
cg04563996	0,473588885	0,651244149	0,177655264	0,001216137	ARHGEF3	ARHGEF3;SPATA12	NM_001128615;NM_181727	5UTR;TSS1500	
cg15698945	0,95916261	0,852198412	-0,106964198	0,0012168	TAAR9	TAAR9	NM_175057	TSS1500	
cg00973424	0,939177185	0,747872896	-0,191304289	0,001220159					
cg01726890	0,641825814	0,747075744	0,10524993	0,001221198					
cg14470998	0,057083954	0,166440617	0,109356663	0,001223903	TFAP2B	TFAP2B	NM_003221	3UTR	N_Shore
cg13116061	0,91595598	0,792906085	-0,123049895	0,001225265	PAG1	PAG1	NM_018440	5UTR	
cg19995856	0,854803826	0,672670436	-0,18213339	0,001228295					
cg06775361	0,90605273	0,802835967	-0,103216763	0,001229695	SCOC	SCOC	NM_032547	5UTR	
cg14766621	0,858461544	0,756355132	-0,102106412	0,001229742	MYT1L	MYT1L	NM_015025	Body	
cg00547068	0,811714952	0,613059713	-0,198655239	0,001231108	FAM196B	FAM196B;DOCK2	NM_001129891;NM_004946	5UTR;Body	
cg02582191	0,708256176	0,822000962	0,113744786	0,001231292	OR6B2	OR6B2	NM_001005853	TSS1500	
cg16701938	0,876363338	0,732600838	-0,1437625	0,001231336					
cg11940381	0,956508574	0,856304343	-0,100204232	0,001232755	FOXO3	FOXO3;FOXO3	NM_001455;NM_201559	Body;Body	N_Shore
cg22276811	0,877877521	0,72379536	-0,154082161	0,001235233	PSD3	PSD3;PSD3	NM_206909;NM_015310	Body;Body	
cg24034992	0,276967302	0,176418999	-0,100548304	0,001235861	YIPF6	YIPF6	NM_173834	Body	Island
cg05736633	0,942533971	0,808355662	-0,134178308	0,001236094					N_Shelf
cg18412984	0,816596293	0,685867435	-0,130728858	0,001243093	WDR20	WDR20;WDR20;WDR20;WDR20;HSP90AA1	NM_181291;NM_181308;NM_144574;NM_181302;NM_0010179	TSS1500;TSS1500;TSS1500	N_Shore
cg08073874	0,883009772	0,695900341	-0,187109431	0,001244799					
cg14802591	0,938636364	0,821641173	-0,116995191	0,001247166	STT3A	STT3A	NM_152713	Body	
cg11147252	0,889424709	0,742547352	-0,146877357	0,001247451					
cg08831522	0,90372497	0,799584844	-0,104140126	0,001248611	ATP10A	ATP10A	NM_024490	Body	
cg24133724	0,658959995	0,760913434	0,101953439	0,00124969					
cg26266098	0,121670098	0,242396438	0,12072634	0,001249897	LZTS2	LZTS2	NM_032429	TSS200	N_Shore
cg05347985	0,960456249	0,855264827	-0,105191422	0,001251105	AK5	AK5;AK5	NM_174858;NM_012093	Body;5UTR	S_Shelf
cg27409870	0,926993094	0,774439966	-0,152553127	0,001251857					
cg00764981	0,910001511	0,735525569	-0,174475942	0,001251946	ITGAM	ITGAM;ITGAM	NM_001145808;NM_000632	3UTR;3UTR	S_Shore
cg10062617	0,147277853	0,277614281	0,130336427	0,001252067	TSSC4	TSSC4	NM_005706	TSS1500	S_Shore
cg05000275	0,95111809	0,79537212	-0,15574597	0,001252278	SURF6	SURF6	NM_006753	Body	Island
cg17817168	0,648128197	0,750091194	0,101962997	0,001253561	ST8SIA1	ST8SIA1	NM_003034	Body	N_Shore
cg02059980	0,840591415	0,656358275	-0,18423314	0,00125432	SSH1	SSH1;SSH1;SSH1	NM_001161331;NM_001161330;NM_018984	Body;Body;Body	
cg10552603	0,924294332	0,817639926	-0,106654406	0,001256069	PTPRN2	PTPRN2;PTPRN2;PTPRN2	NM_002847;NM_130842;NM_130843	Body;Body;Body	
cg10039523	0,918397507	0,764504088	-0,153893419	0,001257075	VPS13D	VPS13D;VPS13D	NM_015378;NM_018156	Body;Body	
cg24041453	0,125976872	0,242023654	0,116046782	0,001259191	CCDC11	CCDC11	NM_145020	1stExon	N_Shore
cg24608205	0,067939312	0,169886111	0,101946799	0,001259227	SF3B4	SF3B4	NM_005850	TSS1500	S_Shore
cg22825550	0,868958041	0,709596018	-0,159362022	0,001260039					N_Shelf
cg06044945	0,730947171	0,838467491	0,10752032	0,001260942	ARHGEF4	ARHGEF4;ARHGEF4	NM_032995;NM_015320	Body;Body	S_Shore
cg21895314	0,210134118	0,355968107	0,145833989	0,001263698					S_Shore
cg19354579	0,190601927	0,345601442	0,154999515	0,001267182	EPOR	EPOR	NM_000121	Body	Island
cg06067598	0,887270981	0,770199952	-0,11707103	0,00126945	C3orf25	C3orf25	NM_207307	Body	
cg14193550	0,099748534	0,212666042	0,112917508	0,001270109					S_Shelf
cg06425606	0,932868223	0,805199785	-0,127668438	0,001271042	INPP5A	INPP5A	NM_005539	Body	S_Shelf
cg08627621	0,914098263	0,780665876	-0,133432387	0,001271423					
cg23967442	0,924790257	0,756880706	-0,167909551	0,001271955	INPP5A	INPP5A	NM_005539	Body	S_Shelf
cg10506098	0,060187057	0,178334066	0,118147009	0,001273863	TJP1	TJP1;TJP1	NM_175610;NM_003257	Body;Body	N_Shore
cg13362404	0,897253506	0,787338507	-0,109914999	0,001274284					N_Shelf
cg00541544	0,490931836	0,592684505	0,101752669	0,001274654	UNC84B	UNC84B	NM_015374	5UTR	N_Shore
cg23640744	0,825719354	0,685615462	-0,140103892	0,001276064	RASA3	RASA3	NM_007368	Body	Island
cg13875287	0,855929004	0,68454679	-0,171382214	0,001280399	HSPC159	HSPC159	NM_014181	Body	S_Shore
cg13711238	0,796830003	0,609767477	-0,187062525	0,001281967	FAM110B	FAM110B	NM_147189	5UTR	
cg27316828	0,85087564	0,683481912	-0,167393728	0,00128216	ARL3	ARL3	NM_004311	3UTR	
cg00619335	0,211713847	0,111251994	-0,100461853	0,001285429					Island
cg11136235	0,076957914	0,190906675	0,113948761	0,001285476					
cg06931405	0,909534656	0,707762825	-0,201771831	0,001286619	PTK2	PTK2;PTK2	NM_153831;NM_005607	3UTR;3UTR	
cg23234756	0,920353129	0,771753623	-0,148599506	0,001286858	FOXP1	FOXP1;FOXP1	NM_032682;NM_001012505	5UTR;5UTR	
cg20358902	0,938293314	0,773637256	-0,164656059	0,001289674	LCORL	LCORL;LCORL	NM_153686;NM_001166139	Body;Body	N_Shelf
cg07855337	0,754740548	0,858023822	0,103283275	0,001292504					
cg20231025	0,933642985	0,83246562	-0,101177365	0,00129254					S_Shelf
cg14915104	0,878212396	0,775180762	-0,103031635	0,001294399					S_Shelf
cg13236175	0,899801096	0,766121253	-0,133679843	0,001296289					N_Shelf
cg03387145	0,739877938	0,628705813	-0,111172125	0,001296315					N_Shore
cg12063795	0,266559641	0,405919136	0,139359495	0,001297854					Island
cg24330922	0,091239824	0,217570071	0,126330247	0,00129893	MRPS18B	MRPS18B;PPP1R10	NM_014046;NM_002714	TSS1500;5UTR	N_Shore
cg02527496	0,965802155	0,860970824	-0,104831331	0,001301283	MAP3K7IP1	MAP3K7IP1;MAP3K7IP1	NM_153497;NM_006116	TSS1500;TSS1500	N_Shore
cg26154714	0,685583703	0,81506777	0,129484067	0,001303691					N_Shore
cg07851361	0,671850846	0,542994244	-0,128856602	0,001305269					S_Shelf
cg13033509	0,088256995	0,208659201	0,120402206	0,001306551	SRFBP1	SRFBP1	NM_152546	TSS1500	N_Shore
cg15033973	0,869497074	0,676586333	-0,192910741	0,001306554	MIR2114	MIR2114	NR_031748	TSS1500	
cg25646649	0,834094524	0,729482138	-0,104612385	0,001306983					
cg18720622	0,803617611	0,625579699	-0,178037913	0,00130832	SLC6A18	SLC6A18	NM_182632	Body	S_Shore
cg27216788	0,208239268	0,337463546	0,129224277	0,001308802	ST3GAL2	ST3GAL2	NM_006927	3UTR	N_Shore
cg15439745	0,142979898	0,245327737	0,102347839	0,001308898	ATAD5	ATAD5	NM_024857	TSS1500	N_Shore
cg00515408	0,163822314	0,276105586	0,112283271	0,001310191	ANKS1B	ANKS1B;ANKS1B	NM_152788;NM_152788	5UTR;1stExon	N_Shore
cg24445405	0,959659923	0,857963121	-0,101696802	0,001310455	MIS12	MIS12	NM_024039	5UTR	S_Shore
cg26181277	0,857281307	0,737280986	-0,120000321	0,001311256	KIAA0146	KIAA0146	NM_001080394	Body	
cg03800150	0,916193435	0,79396772	-0,122225715	0,001312691	GIMAP7	GIMAP7	NM_153236	5UTR	
cg25804443	0,879661633	0,724957877	-0,154707357	0,001313746	DLGAP1	DLGAP1	NM_004746	Body	N_Shelf
cg26155673	0,108761751	0,230417926	0,121656174	0,00131482	FAM86B1	FAM86B1;FAM86B1	NR_003494;NM_001083537	TSS1500;TSS1500	Island
cg18585581	0,662123111	0,765697228	0,103574117	0,001315967	WDR13	WDR13;WDR13;WDR13	NM_017883;NM_001166426;NR_029427	TSS1500;TSS1500;TSS1500	N_Shore
cg21216570	0,898262614	0,721972552	-0,176290062	0,00131632	COL5A1	COL5A1	NM_000093	Body	S_Shelf
cg23429240	0,072559976	0,185783924	0,113223949	0,001316386	CORO1C	CORO1C	NM_014325	3UTR	
cg08589776	0,237036005	0,416842516	0,179806511	0,001317599					Island
cg10143911	0,932863021	0,806366896	-0,126496124	0,001323121	RAB7A	RAB7A	NM_004637	5UTR	
cg09730349	0,098869528	0,211295881	0,112426353	0,001326523	C14orf143	C14orf143	NM_145231	Body	N_Shore
cg09729155	0,906875408	0,741492266	-0,165383142	0,001329387	MGC2889	MGC2889;HRASLS	NR_026877;NM_020386	Body;5UTR	S_Shore
cg21190742	0,66859878	0,787702815	0,119104035	0,001338149	C9orf171	C9orf171	NM_207417	Body	
cg20891862	0,105506314	0,21190983	0,106403516	0,001339418	RNASEK	RNASEK	NM_001004333	TSS1500	N_Shore
cg05032957	0,659766426	0,772270514	0,112504089	0,001339893	OR5E1P	OR5E1P	NR_027711	TSS1500	
cg10379687	0,65878333	0,773313704	0,114530374	0,001351221	SPINLW1	SPINLW1;SPINLW1	NM_020398;NM_181502	1stExon;TSS1500	
cg16939112	0,949681244	0,797465097	-0,152216147	0,00135273	ADIPOR2	ADIPOR2	NM_024551	5UTR	
cg13793166	0,101090165	0,225793572	0,124703407	0,001353064	PHF19	PHF19;PHF19	NM_001009936;NM_015651	TSS1500;TSS1500	S_Shore
cg21036194	0,445502437	0,940396083	0,494893646	0,001353159	SNCAIP	SNCAIP	NM_005460	Body	
cg18291151	0,936446379	0,783460191	-0,152986188	0,001355928					
cg19358520	0,630714801	0,73138824	0,100673439	0,001356271	SLC22A6	SLC22A6;SLC22A6;SLC22A6;SLC22A6	NM_153276;NM_153277;NM_153278;NM_004790	3UTR;3UTR;3UTR;3UTR	
cg04772833	0,937645003	0,82664745	-0,110997553	0,00135889					N_Shore
cg17330515	0,84294373	0,738711132	-0,104232599	0,001359497	SYCE2	SYCE2	NM_001105578	TSS1500	S_Shore
cg12425283	0,912289806	0,787359104	-0,124930703	0,001360471	STARD7	STARD7	NM_020151	Body	
cg12438044	0,932365816	0,743242648	-0,189123168	0,001362455	ATG10	ATG10;ATG10	NM_001131028;NM_031482	5UTR;5UTR	
cg09151583	0,899202933	0,794109139	-0,105093795	0,001368167	GAK	GAK	NM_005255	Body	S_Shelf
cg14523725	0,918940908	0,749951464	-0,168989444	0,001368414					
cg00094158	0,919149022	0,79940284	-0,119746181	0,001368478					S_Shelf
cg08072217	0,848781433	0,718366147	-0,130415286	0,001371448	MAD1L1	MAD1L1;MAD1L1;MAD1L1	NM_003550;NM_001013837;NM_001013836	Body;Body;Body	
cg09345868	0,80049016	0,569871967	-0,230618193	0,001371771	SAMD3	SAMD3;SAMD3	NM_001017373;NM_152552	5UTR;TSS1500	
cg12562822	0,211947685	0,331127871	0,119180186	0,001371955	SLC6A19	SLC6A19	NM_001003841	Body	S_Shore
cg14709826	0,126621771	0,245904419	0,119282648	0,001374007					
cg14703224	0,243326002	0,351043322	0,107717319	0,001374337	SST	SST	NM_001048	TSS1500	S_Shore
cg00139517	0,953267152	0,836873734	-0,116393417	0,00137473	SNTG1	SNTG1	NM_018967	5UTR	
cg03708188	0,960833773	0,823759994	-0,137073779	0,001375131	TRIM36	TRIM36;TRIM36;TRIM36	NM_018700;NM_001017397;NM_001017398	Body;Body;Body	N_Shore
cg17216092	0,919332102	0,79560559	-0,123726543	0,00137592	PRSS37	PRSS37	NM_001008270	Body	
cg05504279	0,91027153	0,761443788	-0,148827742	0,001376778	RUNDC2A	RUNDC2A	NM_032167	Body	
cg20899681	0,877035924	0,676232543	-0,200803381	0,001379027					
cg11271345	0,947187933	0,8313772	-0,115810733	0,001379872	ACTBL2	ACTBL2	NM_001017992	TSS1500	
cg03642549	0,90768629	0,769505031	-0,138181259	0,001380304	IGHMBP2	IGHMBP2	NM_002180	Body	S_Shore
cg00345744	0,0690372	0,173880694	0,104843494	0,00138262					N_Shelf
cg00507945	0,151068811	0,292367512	0,141298701	0,001386736					N_Shore
cg24239882	0,138537076	0,255565833	0,117028757	0,001389269	ST8SIA1	ST8SIA1	NM_003034	TSS1500	Island
cg08702344	0,041625371	0,15047758	0,108852209	0,001390151	CLDN12	CLDN12	NM_012129	TSS1500	
cg01133413	0,768391372	0,873401381	0,105010009	0,001391068	PAGE5	PAGE5;PAGE5	NM_130467;NM_001013435	TSS200;TSS200	
cg23061121	0,911042353	0,775702164	-0,13534019	0,001391248					
cg08273740	0,683167932	0,78602663	0,102858698	0,001391342	C11orf21	C11orf21;TSPAN32;C11orf21	NR_024621;NM_139022;NM_001142946	Body;TSS1500;Body	
cg08121408	0,603190559	0,708523752	0,105333193	0,001394388	SLC1A7	SLC1A7	NM_006671	Body	
cg11736929	0,917599673	0,785628212	-0,131971461	0,001399726					
cg12449813	0,704713247	0,807991738	0,103278491	0,001399751	LOC145837	LOC145837	NR_026979	TSS1500	
cg20348196	0,855172924	0,670424127	-0,184748797	0,001400914	HLA-DRA	HLA-DRA	NM_019111	Body	
cg07698039	0,935848097	0,831611761	-0,104236336	0,001400945					
cg20251837	0,903016697	0,765768399	-0,137248297	0,001401239	mars-04	MARCH4	NM_020814	Body	
cg05264033	0,615162776	0,725127141	0,109964364	0,001403638	MAGEE2	MAGEE2	NM_138703	TSS1500	
cg10812466	0,922733904	0,694199767	-0,228534137	0,001405393					S_Shelf
cg17033684	0,576708131	0,685803159	0,109095027	0,001406401	SLC13A5	SLC13A5;SLC13A5	NM_177550;NM_001143838	3UTR;3UTR	

cg27112585	0,628670016	0,739548643	0,110878628	0,00141079	SHANK2	SHANK2;SHANK2	NM_012309;NM_133266	Body;Body	
cg06919693	0,908359104	0,769766553	-0,138592551	0,001411905	MEIS1	MEIS1	NM_002398	Body	
cg24507739	0,075165887	0,214338028	0,139172142	0,001414173	MPV17	MPV17	NM_002437	TSS1500	
cg20814026	0,069657011	0,1809621	0,111305089	0,00141437	ANKRD53	ANKRD53;ANKRD53	NM_001115116;NM_024933	TSS200;TSS200	N_Shore
cg15420906	0,876046847	0,686294467	-0,18975238	0,001415314	FAM71D	FAM71D	NM_173526	Body	
cg06454983	0,923425338	0,800299378	-0,123125961	0,001415381	SLMO1	SLMO1;SLMO1	NM_006553;NM_001142405	Body;Body	N_Shelf
cg17120792	0,69937828	0,587360623	-0,112017657	0,001415888	OR5I1	OR5I1	NM_006637	TSS1500	
cg08547534	0,759954952	0,60673127	-0,153223682	0,001424028					
cg15846771	0,887536234	0,71382026	-0,173715974	0,001424578					
cg01607187	0,873889061	0,72723566	-0,146653401	0,001424692	GPA33	GPA33	NM_005814	TSS1500	
cg03529555	0,924030868	0,764977697	-0,159053171	0,001426196	NAV2	NAV2	NM_001111018	Body	
cg13504434	0,66344495	0,765868144	0,102423193	0,00142893	CABP1	CABP1;CABP1;CABP1	NM_004276;NM_031205;NM_001033677	TSS200;TSS200;Body	
cg01936122	0,920360307	0,798305817	-0,12205449	0,001433282	ZNF534	ZNF534;ZNF534	NM_001143938;NM_001143939	TSS1500;TSS1500	
cg19923238	0,703765154	0,813525252	0,109760097	0,001434295	SLC45A4	SLC45A4	NM_001080431	Body	S_Shore
cg16539272	0,153275539	0,260671746	0,107396206	0,001434569	GRID1	GRID1	NM_017551	Body	Island
cg08575688	0,801424782	0,579583758	-0,221841024	0,00144185	CCL20	CCL20;CCL20	NM_001130046;NM_004591	TSS200;TSS200	
cg18079334	0,169673755	0,402476731	0,232802976	0,001441901	ARRDC4	ARRDC4	NM_183376	TSS1500	N_Shore
cg10551645	0,939985126	0,82561003	-0,114375096	0,001448842	CLEC9A	CLEC9A	NM_207345	3UTR	
cg25131079	0,623580032	0,723863728	0,100283696	0,001451954	ADAMTS2	ADAMTS2;ADAMTS2	NM_021599;NM_014244	Body;Body	
cg08492238	0,548256746	0,654656429	0,106399684	0,001455746					S_Shelf
cg15527491	0,878654144	0,743862941	-0,134791204	0,00145604	STMN1	STMN1;STMN1;STMN1;STMN1	NM_203401;NM_203399;NM_005563;NM_001145454	Body;Body;Body;Body	N_Shelf
cg00458317	0,784262017	0,918346468	0,134084452	0,001459731	KRTAP24-1	KRTAP24-1	NM_001085455	TSS200	
cg08839312	0,069829346	0,263692604	0,193863259	0,00146	CDH15	CDH15	NM_004933	TSS200	Island
cg05094429	0,709464364	0,813324039	0,103859675	0,001460945	CCR6	CCR6;CCR6	NM_004367;NM_031409	5UTR;TSS200	
cg20119308	0,954180572	0,822168547	-0,132012025	0,001461149	RASSF1	RASSF1;RASSF1;RASSF1;RASSF1	NM_170713;NM_170712;NM_170714;NM_007182	TSS1500;TSS1500;Body;Bod	N_Shore
cg03276401	0,183844686	0,295783067	0,111938382	0,001462521	TEX2	TEX2	NM_018469	3UTR	S_Shore
cg18191196	0,548534097	0,653715296	0,105181199	0,001465404	CUL5	CUL5	NM_003478	Body	
cg10082114	0,24133061	0,364514554	0,123183944	0,001466313	ATRX	ATRX;ATRX	NM_138270;NM_000489	Body;Body	Island
cg11052668	0,047051353	0,161181269	0,114129917	0,001466633					
cg12352347	0,092903119	0,226850805	0,133947686	0,001467992					
cg19947463	0,927203009	0,820793255	-0,106409753	0,001468197	C7orf50	C7orf50;C7orf50;C7orf50	NM_001134395;NM_032350;NM_001134396	Body;Body;Body	
cg13326647	0,18503112	0,335020774	0,149989653	0,001471					Island
cg18545608	0,862814982	0,761511695	-0,101303287	0,001472589					S_Shelf
cg11233533	0,90860171	0,78725077	-0,12135094	0,001473652	ZNF544	ZNF544	NM_014480	Body	
cg23374834	0,883554518	0,712879368	-0,17067515	0,001475386					
cg27162196	0,123916522	0,225846857	0,101930335	0,001478548	ZFP1	ZFP1	NM_153688	5UTR	S_Shore
cg00584686	0,918895283	0,809771833	-0,10912345	0,001478564	SNTG2	SNTG2	NM_018968	Body	N_Shore
cg15721238	0,189962093	0,338775316	0,148813223	0,001479775	PAK2	PAK2	NM_002577	Body	
cg00682203	0,963249188	0,839692202	-0,123556987	0,001483878	IGF2BP1	IGF2BP1;IGF2BP1	NM_006546;NM_001160423	Body;Body	N_Shelf
cg04823492	0,680169779	0,803192311	0,123022532	0,0014856	ENTPD1	ENTPD1	NM_001098175	TSS1500	
cg18541453	0,850669116	0,691206986	-0,15946213	0,001491281					
cg26228531	0,884241676	0,737330541	-0,146911135	0,001493644					
cg00558031	0,944045431	0,832217965	-0,111827466	0,001498219	C4orf23	C4orf23	NM_152544	Body	
cg15284082	0,776881292	0,879649915	0,102768623	0,001499577					
cg15832574	0,929928085	0,801410034	-0,128518051	0,001502173	ALG6	ALG6	NM_013339	Body	
cg01908508	0,901344301	0,715192107	-0,186152194	0,001502735					N_Shore
cg23421166	0,925899605	0,825211209	-0,100688396	0,001502766	ART3	ART3	NM_001130017	5UTR	
cg26970800	0,737339026	0,842742414	0,105403388	0,00150301	GIF	GIF	NM_005142	TSS1500	
cg25229470	0,122749034	0,265871553	0,14312252	0,001503256	GATA2	GATA2;GATA2;GATA2	NM_001145661;NM_032638;NM_001145662	Body;Body;Body	N_Shore
cg08795861	0,964913507	0,864007977	-0,10090553	0,001505183	MED24	MED24;MED24	NM_001079518;NM_014815	TSS1500;TSS1500	S_Shore
cg24704151	0,939117121	0,818605953	-0,120511168	0,001508649	PDCD5	PDCD5	NM_004708	3UTR	
cg10338338	0,158265316	0,273910292	0,115644976	0,001509368	SLC2A3	SLC2A3	NM_006931	TSS200	
cg15127563	0,1770699	0,34097026	0,163900361	0,001515151	ITM2C	ITM2C;ITM2C;ITM2C	NM_030926;NM_001012516;NM_001012514	TSS200;TSS200;TSS200	N_Shore
cg03488097	0,778209268	0,63153606	-0,146673208	0,001516698					Island
cg11995490	0,787559492	0,620981716	-0,166577776	0,001518363	C7orf50	C7orf50;C7orf50;C7orf50	NM_001134395;NM_032350;NM_001134396	Body;Body;Body	
cg25274185	0,681458055	0,532917176	-0,148540879	0,001523589	FRY	FRY	NM_023037	Body	
cg22792048	0,959126932	0,855145361	-0,103981572	0,001524262					N_Shore
cg17772543	0,104147206	0,249973492	0,145826287	0,00152639					Island
cg16991671	0,60759595	0,709744758	0,102148807	0,001530074	GMFB	GMFB	NM_004124	TSS1500	S_Shore
cg01933276	0,827363151	0,680299063	-0,147064088	0,001535232	SELV	SELV	NM_182704	TSS1500	N_Shore
cg13214474	0,934339072	0,801533611	-0,132805461	0,001536096	KCNK2	KCNK2;KCNK2;KCNK2	NM_001017425;NM_014217;NM_001017424	3UTR;3UTR;3UTR	
cg26813301	0,060413853	0,182301728	0,121887876	0,001543287	LOC652276	LOC652276	NR_015441	TSS200	Island
cg04702045	0,283555238	0,170189296	-0,113365942	0,001544475	OPHN1	OPHN1	NM_002547	TSS1500	Island
cg07099553	0,88842284	0,755730898	-0,132691942	0,001545235					
cg14126863	0,113790148	0,223437774	0,109647625	0,001548773	LSM11	LSM11	NM_173491	Body	S_Shore
cg00727777	0,911684012	0,785260067	-0,126423946	0,001551297					N_Shore
cg25214113	0,945347007	0,826285419	-0,119061588	0,001553901					S_Shelf
cg06621861	0,090153505	0,191376041	0,101222536	0,001555334	DTNB	DTNB;DTNB;DTNB;DTNB;DTNB	NM_183360;NM_033148;NM_021907;NM_033147;NM_183361	5UTR;5UTR;5UTR;5UTR;5UTR	
cg04248332	0,880141738	0,776123066	-0,104018673	0,001555577	SNORD103A	SNORD103A;PUM1;PUM1	NR_004054;NM_014676;NM_001020658	TSS1500;Body;Body	
cg15063715	0,94497405	0,828694523	-0,116279528	0,001558031	CASZ1	CASZ1;CASZ1	NM_001079843;NM_017766	TSS1500;TSS1500	S_Shore
cg00767948	0,959676094	0,816812792	-0,142863301	0,001558811	PTPRG	PTPRG	NM_002841	Body	
cg12600631	0,974479735	0,856666652	-0,117813084	0,001559746					
cg20109495	0,933442097	0,808387757	-0,12505434	0,001566759	OR3A2	OR3A2	NM_002551	TSS1500	
cg05085137	0,26406038	0,390557044	0,126496663	0,001568127	OSR2	OSR2;OSR2	NM_053001;NM_001142462	TSS1500;TSS1500	N_Shore
cg07477947	0,941077153	0,809915941	-0,131161211	0,001568529	ANKRD28	ANKRD28	NM_015199	Body	
cg06058311	0,690303726	0,520890604	-0,169413123	0,001574147	EFR3A	EFR3A	NM_015137	TSS1500	N_Shore
cg18768283	0,943563565	0,817991412	-0,125572154	0,001574822	C15orf55	C15orf55	NM_175741	Body	
cg16694489	0,87477647	0,717288856	-0,157487615	0,001586873	GPR125	GPR125	NM_145290	Body	
cg16357457	0,071757864	0,181644449	0,109886585	0,001589041	SMARCC1	SMARCC1	NM_003074	Body	N_Shore
cg24956253	0,927714378	0,826409689	-0,101304688	0,001591437	CBLN2	CBLN2	NM_182511	Body	N_Shore
cg04257163	0,645259634	0,755943143	0,110683508	0,001592038					
cg06186245	0,666104218	0,77257006	0,106465842	0,001593822	TMEM45A	TMEM45A	NM_018004	5UTR	
cg24641427	0,867634209	0,746681005	-0,120953204	0,001594355					N_Shelf
cg01809675	0,831631444	0,730204008	-0,101427436	0,001598588					S_Shore
cg02491398	0,921342011	0,750206431	-0,17113558	0,001599349					N_Shelf
cg04628742	0,813522216	0,916487915	0,102965699	0,001599527	HLA-J	HLA-J;NCRNA00171	NR_024240;NR_026751	TSS1500;Body	N_Shore
cg15362557	0,940954245	0,835929531	-0,105024713	0,001599977	ASAP2	ASAP2;ASAP2	NM_001135191;NM_003887	Body;Body	
cg05412410	0,679062884	0,786401043	0,107338159	0,001600736	CARD11	CARD11	NM_032415	5UTR	
cg24545100	0,680555543	0,784530427	0,103974884	0,001602338	LINGO4	LINGO4;RORC;RORC	NM_001004432;NM_001001523;NM_005060	TSS1500;3UTR;3UTR	
cg08547637	0,856915726	0,696429448	-0,160486278	0,001602973					
cg01779323	0,779329152	0,881485319	0,102156166	0,00160396	KLRG2	KLRG2	NM_198508	Body	N_Shelf
cg09868496	0,8374626	0,597785326	-0,239677274	0,001612066	RERE	RERE;RERE;RERE	NM_001042682;NM_012102;NM_001042681	5UTR;Body;Body	
cg13180232	0,936632329	0,825018617	-0,111613712	0,0016133	CREM	CREM;CREM;CREM;CREM;CREM;CREM;CREM;CREM	NM_183013;NM_183012;NM_182850;NM_182853;NM_183011;N	5UTR;TSS1500;5UTR;5UTR;T	
cg11423096	0,155393969	0,27336551	0,117971541	0,001618286	UIMC1	UIMC1	NM_016290	5UTR	
cg25703243	0,131788424	0,276925204	0,14513678	0,001619199	HHIP	HHIP	NM_022475	Body	S_Shore
cg24797501	0,755842656	0,86436999	0,108527334	0,001620226					
cg01999476	0,897111417	0,759620799	-0,137490617	0,001624288	C3orf14	C3orf14	NM_020685	Body	S_Shelf
cg12445786	0,626591319	0,754214376	0,127623057	0,001624444					
cg17668981	0,845977222	0,720060185	-0,125917038	0,001627892	MATR3	MATR3	NM_199189	5UTR	
cg01528321	0,085694836	0,230786181	0,145091345	0,001627961	TSPAN14	TSPAN14;TSPAN14	NM_001128309;NM_030927	5UTR;5UTR	Island
cg14059735	0,864593075	0,762488886	-0,102104189	0,001627986					
cg03769090	0,905068172	0,625910782	-0,27915739	0,00163145	C10orf46	C10orf46	NM_153810	Body	
cg06075754	0,589755207	0,451444284	-0,138310923	0,001632942	PSD	PSD	NM_002779	Body	N_Shore
cg24144175	0,10485231	0,261216553	0,156364242	0,001633997	PPP1R10	PPP1R10	NM_002714	5UTR	N_Shore
cg02582194	0,934687347	0,770144674	-0,164542673	0,00163688					S_Shelf
cg20891060	0,614864675	0,735189781	0,120325106	0,001638419					N_Shelf
cg08233331	0,906921884	0,803436166	-0,103485718	0,001638576	C18orf2	C18orf2;C18orf2;C18orf2;C18orf2	NR_023925;NR_023928;NR_023927;NR_023926	TSS200;TSS200;TSS200;TSS	
cg26347359	0,924942754	0,791514519	-0,133428234	0,001640594	PHF17	PHF17;PHF17	NM_024900;NM_199320	Body;Body	
cg19508435	0,084572298	0,248671497	0,164099199	0,00164069	METT5D1	METT5D1;METT5D1	NM_152636;NM_001113528	5UTR;5UTR	Island
cg05601201	0,916382556	0,777577555	-0,138805001	0,001644827	FOXO3	FOXO3;FOXO3	NM_001455;NM_201559	Body;Body	
cg09691134	0,945288646	0,829757115	-0,115531532	0,001649662					N_Shore
cg07520608	0,144555766	0,29480696	0,150251194	0,001650629	KLHL29	KLHL29	NM_052920	5UTR	
cg23766918	0,667789651	0,776639277	0,108849626	0,001652214	USP43	USP43	NM_153210	Body	
cg26462488	0,916630043	0,805395163	-0,11123488	0,001653214	LOC100129550	LOC100129550	NR_024618	TSS200	
cg14073011	0,667614954	0,782856314	0,11524136	0,001653234	CMAH	CMAH	NR_027626	TSS1500	Island
cg02392124	0,81705552	0,709601095	-0,107454425	0,001653392	WAC	WAC;WAC;WAC	NM_100486;NM_016628;NR_024557	Body;Body;Body	
cg24860782	0,837713344	0,596806706	-0,240906638	0,001656022					
cg22350438	0,582683667	0,704772918	0,122089251	0,001657804	SYNC	SYNC;RBBP4;SYNC;RBBP4;RBBP4	NM_030786;NM_005610;NM_001161708;NM_001135255;NM_0	Body;3UTR;Body;3UTR;3UTR	
cg23262550	0,906476507	0,806001455	-0,100475052	0,001662583					
cg18096251	0,734647599	0,896947458	0,162299859	0,001662676					
cg18107960	0,105710555	0,25418097	0,148470416	0,001662676	WDR41	WDR41	NM_018268	Body	N_Shore
cg25389374	0,926909176	0,746895983	-0,180013193	0,001662696					S_Shelf
cg20510272	0,071549162	0,173810658	0,102261496	0,001664569	GOLT1A	GOLT1A	NM_198447	TSS1500	
cg08457298	0,45356827	0,585591823	0,132023553	0,001667492	GOLSYN	GOLSYN;GOLSYN;GOLSYN;GOLSYN	NM_001099743;NM_001099745;NM_001099746;NM_00109974	5UTR;TSS200;TSS200;5UTR	
cg20109856	0,217738932	0,353155694	0,135416762	0,001667753	DLX6AS	DLX6AS	NR_015448	TSS200	N_Shelf
cg26686753	0,876369423	0,744516042	-0,131853381	0,00166833	LEPREL1	LEPREL1	NM_001134418	TSS1500	S_Shelf
cg00981661	0,744243035	0,544859746	-0,199383289	0,001674046	MKLN1	MKLN1	NM_001145354	Body	
cg15665833	0,088713134	0,203255899	0,114542765	0,001674161					N_Shore
cg18780288	0,784673607	0,889561626	0,104888019	0,001676195	XPNPEP1	XPNPEP1;XPNPEP1;XPNPEP1	NR_030724;NM_020383;NM_001167604	Body;Body;Body	
cg21429780	0,959226891	0,858617198	-0,100609693	0,001676373	MAML3	MAML3	NM_018717	Body	N_Shore
cg11216456	0,920442604	0,802473611	-0,117968993	0,001677115	MGMT	MGMT	NM_002412	Body	
cg16139006	0,75556407	0,859967411	0,104403341	0,00167798					S_Shore
cg17276106	0,852556828	0,723374767	-0,12918206	0,001678277	STEAP3	STEAP3;STEAP3;STEAP3	NM_018234;NM_182915;NM_001008410	Body;Body;Body	S_Shore
cg08544841	0,959993973	0,83633475	-0,123659223	0,001679036					
cg00995689	0,920569394	0,808462013	-0,11210738	0,001679206					
cg05482498	0,226729034	0,344480173	0,117751139	0,001680543					S_Shelf
cg21836903	0,360258121	0,473344347	0,113086227	0,001681794	FOXP1	FOXP1	NM_032682	Body	
cg03269320	0,929250185	0,814132322	-0,115117863	0,001683844					
cg23564178	0,074830213	0,179754187	0,104923974	0,001685391					
cg21877473	0,774730148	0,625379892	-0,149350255	0,00168604	CASC4	CASC4;CASC4	NM_138423;NM_177974	Body;Body	
cg14930864	0,860954645	0,713256775	-0,147697871	0,001686304	YWHAQ	YWHAQ	NM_006826	Body	
cg23475480	0,954339519	0,83912349	-0,115216029	0,00168687	FAM161B	FAM161B	NM_152445	Body	N_Shelf
cg19635695	0,637752282	0,760465326	0,122713044	0,001688006	PDE6C	PDE6C;PDE6C	NM_006204;NM_006204	5UTR;1stExon	
cg18920427	0,269087364	0,416695593	0,147608229	0,001689492	GLA	GLA;HNRNPH2;HNRNPH2	NM_000169;NM_001032393;NM_019597	TSS1500;5UTR;5UTR	Island
cg13970942	0,960502887	0,808759894	-0,151742993	0,001689834					N_Shelf
cg04413193	0,919565121	0,798241113	-0,121324008	0,001690219					
cg23736895	0,929801964	0,804771586	-0,125030378	0,001694809					
cg03974193	0,100807463	0,20265037	0,101842907	0,001696762	MAP4K4	MAP4K4;MAP4K4;MAP4K4	NM_145687;NM_004834;NM_145686	Body;Body;Body	S_Shore
cg20596640	0,099717441	0,211351777	0,111634336	0,001697707	ZMYM5	ZMYM5;ZMYM5;ZMYM5	NM_001142684;NM_001039649;NM_001039650	5UTR;5UTR;5UTR	N_Shore
cg27507752	0,92939007	0,741123427	-0,188266643	0,001701538	ASNS	ASNS;ASNS;ASNS	NM_001673;NM_183356;NM_133436	Body;Body;Body	N_Shelf
cg25497157	0,66604094	0,531024354	-0,135016587	0,001703255					S_Shelf
cg04551500	0,887991468	0,758198607	-0,129792861	0,001703522	SYNC	SYNC;SYNC	NM_030786;NM_001161708	TSS1500;TSS1500	S_Shore
cg20356147	0,913384819	0,660058508	-0,253326311	0,001709884	EEFSEC	EEFSEC	NM_021937	Body	
cg12295277	0,882216011	0,661456999	-0,220759013	0,001715766	UXS1	UXS1	NM_025076	3UTR	
cg08119777	0,891884935	0,719195792	-0,172689143	0,001716501					
cg08420232	0,922452902	0,803839464	-0,118613438	0,001721016	KCTD15	KCTD15;KCTD15;KCTD15	NM_001129994;NM_001129995;NM_024076	5UTR;5UTR;5UTR	S_Shore
cg27435867	0,833228392	0,700824465	-0,132403927	0,001727658					
cg06773023	0,055625456	0,159107906	0,10348245	0,001729963	EFR3A	EFR3A	NM_015137	Body	S_Shore
cg02365742	0,152233574	0,281593135	0,129359561	0,001732649	MSX2	MSX2	NM_002449	TSS1500	N_Shore
cg18034501	0,776355962	0,527143331	-0,249212632	0,001733282					
cg10554698	0,937334242	0,828467357	-0,108866885	0,001734971	SPRED2	SPRED2;SPRED2	NM_001128210;NM_181784	Body;Body	S_Shore
cg01768144	0,943186267	0,832534295	-0,110651972	0,001735853	LOC100303728	LOC100303728;SLC25A5	NR_028443;NM_001152	Body;TSS1500	N_Shore
cg13037952	0,83883756	0,708710687	-0,130126874	0,00173882					S_Shelf
cg02741329	0,939452192	0,835627469	-0,103824723	0,001740066	LSAMP	LSAMP	NM_002338	Body	
cg02835823	0,716534149	0,831017834	0,114483685	0,001742348					N_Shelf
cg00402547	0,941371285	0,834244185	-0,1071271	0,001745112	ARHGAP28	ARHGAP28;ARHGAP28	NM_030672;NM_001010000	5UTR;5UTR	
cg00677272	0,924926909	0,76314301	-0,161783899	0,001749203					
cg10796131	0,881488472	0,744635223	-0,136853249	0,001750741					
cg26678575	0,111855069	0,246465286	0,134610217	0,001757281	PTPN21	PTPN21	NM_007039	TSS1500	S_Shore
cg16294414	0,350733725	0,47596074	0,125227015	0,001757419	CROCC	CROCC	NM_014675	Body	S_Shore
cg12792086	0,159900627	0,285008921	0,125108294	0,001765664	CPLX1	CPLX1	NM_006651	Body	Island
cg17187559	0,114551307	0,248864826	0,134313519	0,001765743	ZNF248	ZNF248	NM_021045	TSS200	Island
cg26472089	0,905349764	0,754203177	-0,151146587	0,001767632	MAPK14	MAPK14;MAPK14;MAPK14;MAPK14	NM_139014;NM_139013;NM_139012;NM_001315	Body;Body;Body;Body	
cg06165495	0,78568935	0,888349251	0,102659901	0,001770934					
cg01877595	0,683309344	0,793454564	0,11014522	0,001777792					S_Shelf
cg03839322	0,879376736	0,740204895	-0,13917184	0,00178042	KIFC3	KIFC3;KIFC3;KIFC3	NM_005550;NM_001130100;NM_001130099	Body;Body;5UTR	
cg02148222	0,096011639	0,196075251	0,100063612	0,001780959	ST6GAL1	ST6GAL1;ST6GAL1	NM_173216;NM_173217	5UTR;5UTR	S_Shore
cg10946681	0,845725006	0,697449173	-0,148275833	0,001784068					S_Shelf
cg07511008	0,869337333	0,608523608	-0,260813724	0,001785845	ADAM10	ADAM10	NM_001110	Body	
cg05937603	0,922187359	0,703950735	-0,218236624	0,001789375	SKI	SKI	NM_003036	Body	
cg10439859	0,817448984	0,692143522	-0,125305462	0,001789513	RHBDD1	RHBDD1;RHBDD1	NM_001167608;NM_032276	Body;Body	

cg22657044	0,271712277	0,391332006	0,119619729	0,001793456	RNF114	RNF114	NM_018683	TSS1500	N_Shore
cg06880615	0,743493174	0,61579623	-0,127696945	0,001796956	GRIN2A	GRIN2A;GRIN2A;GRIN2A	NM_001134407;NM_000833;NM_001134408	Body;Body;Body	
cg09802689	0,94459212	0,840703635	-0,103888485	0,001801699					S_Shelf
cg22582187	0,786467698	0,492508361	-0,293959337	0,001805248					
cg24827258	0,12412362	0,230193763	0,106070142	0,001805649	NDUFS6	NDUFS6;MRPL36	NM_004553;NM_032479	TSS1500;TSS1500	Island
cg13965224	0,931576399	0,820118686	-0,111457714	0,001806471	CTBP1	CTBP1;CTBP1	NM_001328;NM_001012614	Body;Body	N_Shore
cg09595415	0,156230248	0,285760252	0,129530003	0,001806487	SRY	SRY	NM_003140	TSS200	N_Shore
cg16531222	0,949704715	0,84448822	-0,105216495	0,001814331	ETV6	ETV6	NM_001987	Body	
cg10827754	0,922969215	0,781852498	-0,141116717	0,001820919					S_Shelf
cg16198930	0,668640551	0,488593811	-0,18004674	0,001824293					
cg19325487	0,767550816	0,867703752	0,100152935	0,001825596					
cg13950362	0,639831685	0,745282821	0,105451136	0,001828022	DEFB123	DEFB123	NM_153324	TSS1500	
cg27386614	0,915575354	0,814314729	-0,101260625	0,001828286	AQR	AQR	NM_014691	TSS1500	S_Shore
cg12970081	0,670089564	0,788395861	0,118306297	0,00183017	GRP32	GRP32	NM_001506	1stExon	
cg08643824	0,741317697	0,846914801	0,105597104	0,001833431	LPXN	LPXN;LPXN	NM_001143995;NM_004811	3UTR;3UTR	
cg26719638	0,925568034	0,795134579	-0,130433455	0,001837619					S_Shelf
cg11053489	0,0995009	0,201415838	0,101914937	0,001838484	ENTPD5	ENTPD5;C14orf45	NM_001249;NM_025057	5UTR;TSS1500	N_Shore
cg13375696	0,935673942	0,757611233	-0,17806271	0,001839272					
cg22795059	0,876840105	0,666081485	-0,210758621	0,001841558					S_Shelf
cg02795700	0,103622541	0,204373521	0,10075098	0,001844196	SPTBN4	SPTBN4;SPTBN4	NM_025213;NM_020971	TSS200;Body	S_Shore
cg17998283	0,735295559	0,563424219	-0,171871339	0,001844619					S_Shelf
cg27565550	0,719824537	0,587830995	-0,131993542	0,001847062					
cg20768399	0,709514168	0,822097664	0,112583496	0,001847474	SNIP1	SNIP1	NM_024700	TSS1500	S_Shore
cg00507855	0,878263243	0,775145783	-0,103117459	0,001849351	EYA2	EYA2;EYA2	NM_172110;NM_005244	5UTR;5UTR	
cg02614372	0,894403443	0,753851933	-0,14055151	0,001850493	NXPH1	NXPH1	NM_152745	Body	S_Shelf
cg17371020	0,271096538	0,37301897	0,101922432	0,001853349	TRIM2	TRIM2;TRIM2	NM_015271;NM_001130067	TSS200;5UTR	
cg18764240	0,841587912	0,701785904	-0,139802008	0,001857758	FAM47C	FAM47C	NM_001013736	1stExon	Island
cg04320632	0,967509732	0,782068427	-0,185441305	0,001867361					N_Shelf
cg13764979	0,212452378	0,348461327	0,136008948	0,001868375					
cg00368577	0,703513012	0,804272939	0,100759927	0,001874162	KRT5	KRT5	NM_000424	TSS1500	
cg01441127	0,894221246	0,78267777	-0,111543476	0,00187509	MR1	MR1	NM_001531	Body	
cg25972924	0,114319006	0,227592816	0,11327381	0,0018764	FOXM1	FOXM1;C12orf32;C12orf32;FOXM1;FOXM1;C12orf32	NM_021953;NR_027363;NR_027365;NM_202003;NM_202002;N	TSS1500;Body;Body;TSS150	S_Shore
cg25806491	0,843254905	0,712496518	-0,130758388	0,001883666					
cg04337937	0,859848535	0,695959861	-0,163888674	0,001883879	SACS	SACS	NM_014363	TSS1500	
cg03211132	0,144705857	0,28765843	0,142952574	0,001884125	SELE	SELE	NM_000450	TSS1500	
cg20609911	0,90895658	0,766103985	-0,142852595	0,001886047	MTIF3	MTIF3;MTIF3;MTIF3;MTIF3	NM_001166262;NM_152912;NM_001166261;NM_001166263	Body;Body;Body;Body	
cg00082656	0,833067218	0,683523932	-0,149543286	0,001886107	SRPK2	SRPK2	NM_182692	Body	S_Shore
cg16379462	0,087898875	0,22111926	0,133221051	0,00189139	SAMD4A	SAMD4A;SAMD4A	NM_001161576;NM_015589	Body;Body	
cg16167782	0,852189472	0,582595789	-0,269593683	0,001892059	TSHZ1	TSHZ1	NM_005786	5UTR	S_Shore
cg19112757	0,910325909	0,801901807	-0,108424101	0,001903402					
cg10332321	0,679698618	0,793619868	0,11392125	0,001904407	CAMTA1	CAMTA1	NM_015215	Body	
cg13772903	0,935899345	0,774395167	-0,161504178	0,001906272	C14orf19	C14orf19	NR_002937	Body	
cg06865839	0,864035308	0,735626187	-0,128409122	0,001907975	KIRREL	KIRREL	NM_018240	Body	
cg19290808	0,978495327	0,858614897	-0,119880431	0,001909672					N_Shelf
cg05356778	0,593996749	0,704607554	0,110610806	0,001911535	WBSCR17	WBSCR17	NM_022479	Body	
cg11085062	0,914855295	0,803479836	-0,111375459	0,001917823	ZNF280D	ZNF280D;ZNF280D;ZNF280D	NM_001002844;NM_001002843;NM_017661	5UTR;5UTR;5UTR	N_Shore
cg16754015	0,913328368	0,772209356	-0,141119012	0,001918614	ADARB2	ADARB2	NM_018702	Body	N_Shore
cg04330597	0,533161183	0,636479656	0,103318473	0,001919252	SH3BP4	SH3BP4	NM_014521	5UTR	
cg24029640	0,914531927	0,80401256	-0,110519367	0,00192631					
cg08614769	0,95586663	0,818664325	-0,137202305	0,001927183	GMDS	GMDS	NM_001500	Body	
cg04894216	0,91530137	0,72932199	-0,18597938	0,00192907	GRM3	GRM3	NM_000840	5UTR	
cg08080848	0,944292044	0,837446785	-0,106845259	0,001929717					S_Shelf
cg10475690	0,954892204	0,846768154	-0,10812405	0,001935835	MTCP1NB	MTCP1NB;MTCP1	NM_001018024;NM_001018025	5UTR;Body	
cg22189125	0,918712098	0,7398882	-0,178823898	0,001948905	ZNF302	ZNF302;ZNF302	NM_001012320;NM_018443	Body;Body	S_Shelf
cg17479811	0,767411395	0,592514463	-0,174896932	0,001951968	ZNF705A	ZNF705A	NM_001004328	Body	N_Shelf
cg15735483	0,907755114	0,689798178	-0,217956936	0,001955924	MAP7D2	MAP7D2;MAP7D2;MAP7D2;MAP7D2	NM_001168465;NM_001168467;NM_152780;NM_001168466	TSS1500;TSS1500;TSS1500	Island
cg20794690	0,94245368	0,744311809	-0,198141871	0,001957181	TXNDC2	TXNDC2;TXNDC2;TXNDC2	NM_001098529;NM_001098530;NM_032243	Body;5UTR;5UTR	
cg07054226	0,324421919	0,45746838	0,133046461	0,001958238	ABLIM1	ABLIM1;ABLIM1;ABLIM1	NM_001003408;NM_001003407;NM_002313	Body;Body;Body	
cg03051777	0,150952833	0,299936014	0,14898318	0,001959014					S_Shore
cg01710189	0,121053775	0,313192664	0,192138889	0,001960231	PDLIM2	PDLIM2	NM_176871	3UTR	N_Shore
cg21842031	0,872703388	0,727194373	-0,145509014	0,001962208					S_Shelf
cg24338491	0,148663271	0,264902056	0,116238785	0,00196399					
cg25707877	0,833077151	0,690200576	-0,142876576	0,00196451	SH3RF3	SH3RF3	NM_001099289	Body	
cg10788939	0,955992705	0,855389001	-0,100603704	0,001964763	EFNA5	EFNA5	NM_001962	Body	
cg13496119	0,887708866	0,724632341	-0,163076525	0,001967924					
cg15742123	0,888205072	0,786714059	-0,101491013	0,001969917	C7orf50	C7orf50;C7orf50;C7orf50	NM_001134395;NM_032350;NM_001134396	Body;Body;Body	N_Shelf
cg14158769	0,933953599	0,817114587	-0,116839012	0,001971008	MIR592	MIR592;GRM8;GRM8;GRM8	NR_030323;NM_001127323;NR_028041;NM_000845	Body;Body;Body;Body	
cg01479664	0,859525363	0,70006016	-0,159465203	0,00197149	TEPP	TEPP;TEPP	NM_199456;NM_199046	Body;Body	N_Shore
cg09617135	0,168927124	0,328958621	0,160031498	0,001975491					Island
cg12151703	0,947058103	0,842684666	-0,104373438	0,001976788					
cg14778264	0,864126647	0,75604201	-0,108084637	0,001979527					
cg01709551	0,81801476	0,621965899	-0,196048862	0,001981138					Island
cg21818540	0,959868215	0,840885806	-0,118982408	0,001983845					
cg22449330	0,531820195	0,396067065	-0,13575313	0,001987357					
cg05584692	0,873176121	0,679896327	-0,193279794	0,001995708					
cg00843538	0,9486725	0,847198237	-0,101474263	0,0019996					
cg13485061	0,930484561	0,774792358	-0,155692202	0,002014003	AMPD1	AMPD1	NM_000036	Body	S_Shelf
cg01982849	0,888538868	0,781934526	-0,106604341	0,002016415					
cg09287295	0,848053653	0,704399879	-0,143653774	0,002038101	ZNF470	ZNF470	NM_001001668	3UTR	
cg08915024	0,932690893	0,797888502	-0,134802391	0,002038904	TSPAN5	TSPAN5	NM_005723	Body	
cg07394259	0,912138561	0,749097909	-0,163040651	0,002042378	FAM110B	FAM110B	NM_147189	5UTR	
cg12632313	0,274828906	0,39362882	0,118799914	0,002043719	RND3	RND3	NM_005168	Body	
cg07296597	0,682906544	0,786485836	0,103579292	0,00204472					
cg20806296	0,434979237	0,306618578	-0,128360659	0,002050691					
cg20592995	0,743035412	0,848375415	0,105340002	0,002051203	SLC6A4	SLC6A4	NM_001045	3UTR	
cg11937778	0,726955809	0,604895304	-0,122060504	0,002055504	SLC37A2	SLC37A2;SLC37A2	NM_001145290;NM_198277	3UTR;3UTR	
cg02249950	0,961114058	0,847750988	-0,11336307	0,002066092	POPDC3	POPDC3;POPDC3	NM_022361;NR_024539	5UTR;Body	
cg04875088	0,927124359	0,786055751	-0,141068608	0,002066369					
cg24500222	0,876026773	0,74294397	-0,133082803	0,002069361	CALN1	CALN1;CALN1	NM_001017440;NM_031468	Body;Body	
cg22218543	0,153942698	0,27527155	0,121328851	0,002069974	ZNF652	ZNF652;ZNF652	NM_001145365;NM_014897	Body;Body	
cg21941987	0,861489547	0,627319271	-0,234170275	0,00207028	ATP4B	ATP4B	NM_000705	3UTR	S_Shore
cg02211519	0,916176878	0,813954311	-0,102222566	0,002070749	CD81	CD81	NM_004356	Body	S_Shore
cg13298827	0,660214102	0,764816339	0,104602238	0,002073623	PCDHA6	PCDHA6;PCDHA2;PCDHA1;PCDHA9;PCDHA7;PCDHA1;PCDHA6;PC	NM_031849;NM_018905;NM_031411;NM_031857;NM_018910;N	Body;Body;Body;Body;Body;B	S_Shore
cg12110437	0,806117611	0,669658137	-0,136459474	0,002076668	LY6E	LY6E;LY6E;LOC100133669	NM_001127213;NM_002346;NR_026913	TSS1500;TSS1500;Body	N_Shore
cg02899473	0,901161774	0,791401433	-0,109760341	0,002076832	PARVA	PARVA	NM_018222	3UTR	
cg18707028	0,889843615	0,783773531	-0,106070084	0,002077025					S_Shelf
cg21996351	0,927601058	0,820367614	-0,107233445	0,002086918	KLHDC8A	KLHDC8A	NM_018203	TSS200	
cg12510923	0,228808308	0,391988734	0,163180426	0,00209452	HDAC6	HDAC6;HDAC6	NM_006044;NM_006044	1stExon;5UTR	Island
cg26106166	0,933901129	0,807347045	-0,126554085	0,002100331	RSU1	RSU1;RSU1	NM_152724;NM_012425	Body;Body	
cg17113883	0,952287658	0,844574402	-0,107713256	0,002101419	THEMIS	THEMIS;THEMIS	NM_001164687;NM_001164687	5UTR;1stExon	
cg11837293	0,86986332	0,728478588	-0,141384732	0,002101946					
cg11308963	0,887669292	0,752232845	-0,135436446	0,002102067	MYOF	MYOF;MYOF	NM_133337;NM_013451	3UTR;3UTR	
cg07156273	0,690076468	0,79074134	0,100664872	0,00210279	BLCAP	BLCAP;BLCAP;BLCAP;BLCAP;NNAT;NNAT;BLCAP	NM_001167821;NM_001167820;NM_001167822;NM_006698;N	5UTR;5UTR;5UTR;5UTR;TSS1	Island
cg18500368	0,089791435	0,202436413	0,112644978	0,002102909	RPL41	RPL41;RPL41	NM_021104;NM_001035267	TSS1500;TSS1500	N_Shelf
cg23398095	0,890069486	0,754306043	-0,135763043	0,002105618	CREM	CREM;CREM;CREM;CREM;CREM;CREM;CREM;CREM;CREM;CREM	NM_182853;NM_183011;NM_183013;NM_182769;NM_182772;N	5UTR;Body;TSS1500;Body;TSS1500;Bo	
cg05997021	0,085376564	0,195921925	0,11054536	0,002109312	DNAJC19	DNAJC19	NM_145261	TSS1500	S_Shore
cg20944143	0,916428266	0,797871794	-0,118556471	0,00211287					N_Shelf
cg19325435	0,971111344	0,82957849	-0,141532854	0,002113706	ZNF280B	ZNF280B	NM_080764	3UTR	
cg25020969	0,880380025	0,758605814	-0,121774211	0,002114101	MAD1L1	MAD1L1;MAD1L1;MAD1L1	NM_003550;NM_001013837;NM_001013836	Body;Body;Body	Island
cg20646500	0,671347957	0,52175110	-0,149596847	0,002115556	UBR2	UBR2	NM_015255	Body	S_Shelf
cg23347120	0,863112952	0,75673788	-0,106375072	0,002116499	GPR111	GPR111	NM_153839	TSS1500	
cg22283115	0,755090458	0,862276822	0,107186364	0,002117895	RADIL	RADIL	NM_018059	Body	
cg14386728	0,15397663	0,262978608	0,109001978	0,002121258	FEZF1	FEZF1;FEZF1	NM_001024613;NM_001160264	3UTR;3UTR	S_Shore
cg15135617	0,070490396	0,175335914	0,104845518	0,002122326					Island
cg16135771	0,769585809	0,869865248	0,100279439	0,002124377	PLP1	PLP1	NM_001128834	TSS1500	
cg19044256	0,160193276	0,263441193	0,103247917	0,00212914	LOC100128811	LOC100128811;GPR158;GPR158	NR_027333;NM_020752;NM_020752	Body;1stExon;5UTR	Island
cg21275039	0,121800215	0,32769787	0,205897655	0,00212933	USP43	USP43	NM_153210	3UTR	
cg22733073	0,111437151	0,229314021	0,117876869	0,002130412	PAIP2	PAIP2;PAIP2	NM_001033112;NM_016480	TSS1500;TSS1500	N_Shore
cg02252828	0,845219745	0,714298959	-0,130920786	0,002138909	MTMR9L	MTMR9L	NR_026850	Body	N_Shore
cg00611818	0,934291883	0,771953598	-0,162338285	0,002141684					N_Shelf
cg10683527	0,655258929	0,764984799	0,10972587	0,002141948	NEIL3	NEIL3	NM_018248	TSS1500	N_Shore
cg25013910	0,813671789	0,592190294	-0,221481494	0,002147374	TPO	TPO;TPO;TPO;TPO	NM_175719;NM_000547;NM_175721;NM_175722	Body;Body;Body;Body	N_Shelf
cg26036288	0,957908769	0,832586595	-0,125322174	0,002149075	HFE2	HFE2;HFE2;HFE2;HFE2	NM_202004;NM_145277;NM_213652;NM_213653	TSS1500;TSS1500;TSS1500	N_Shelf
cg17722012	0,893311343	0,751595323	-0,14171602	0,002151221					
cg15310415	0,952352482	0,811475492	-0,14087699	0,002153528					
cg18825015	0,759929395	0,636703556	-0,123225839	0,002154291					
cg09149118	0,356956508	0,460682608	0,1037261	0,002155279	ITGAV	ITGAV;ITGAV;ITGAV	NM_001144999;NM_001145000;NM_002210	TSS1500;Body;Body	
cg10169183	0,917032924	0,771063819	-0,145969105	0,002159021	C16orf46	C16orf46;C16orf46	NM_152337;NM_001100873	TSS1500;TSS1500	S_Shore
cg22215489	0,900150829	0,792883296	-0,107267532	0,00217223					N_Shelf
cg15494485	0,94641444	0,809230908	-0,137183532	0,002172535	LOC100130872	LOC100130872;LOC100130872-SPON2	NR_024569;NR_033182	Body;Body	S_Shelf
cg03527056	0,944028068	0,80989331	-0,134134758	0,002173227					S_Shelf
cg14249174	0,939551927	0,769174905	-0,170377022	0,002174704	ARFIP1	ARFIP1;ARFIP1;ARFIP1	NM_014447;NM_001025595;NM_001025593	Body;Body;Body	
cg19349713	0,717788508	0,56720349	-0,150585018	0,002175072	CPEB1	CPEB1	NM_030594	Body	
cg19935471	0,874806373	0,745596261	-0,129210112	0,002175142	MATN2	MATN2;MATN2	NM_030583;NM_002380	3UTR;3UTR	
cg13296485	0,954783117	0,824818098	-0,129965019	0,002180434	RAB2A	RAB2A	NM_002865	Body	
cg08317284	0,099768697	0,25975299	0,159984293	0,002182493	NFIX	NFIX	NM_002501	TSS1500	N_Shore
cg09773436	0,06698147	0,216588462	0,149606992	0,002183983	TTC30B	TTC30B	NM_152517	TSS1500	S_Shore
cg07622877	0,938448622	0,793159075	-0,145289547	0,002186287					
cg10262744	0,88587907	0,776801915	-0,108977155	0,002186313	MKRN2	MKRN2	NM_014160	Body	S_Shore
cg21859290	0,713845028	0,814779201	0,100934173	0,002187102	CDK2AP2	CDK2AP2	NM_005851	3UTR	N_Shore
cg11048082	0,802215305	0,692369295	-0,10984601	0,002187933					
cg09896298	0,890940622	0,695363632	-0,195576991	0,002191592	DUPD1	DUPD1	NM_001003892	TSS200	
cg04890713	0,780772233	0,58089178	-0,199880453	0,002195105					S_Shelf
cg15648790	0,947290671	0,809688894	-0,137601777	0,002198991					
cg20212077	0,338791301	0,445477637	0,106686336	0,002199551					
cg14812594	0,946398291	0,844302385	-0,102095906	0,002203072	SOCS5	SOCS5;SOCS5	NM_144949;NM_014011	5UTR;5UTR	
cg03100509	0,918254787	0,783303439	-0,134951348	0,002204639					S_Shelf
cg24414182	0,163241378	0,311550152	0,148308774	0,002204704	MYADM	MYADM;MYADM;MYADM	NM_138373;NM_001020819;NM_001020820	TSS200;TSS200;5UTR	N_Shore
cg03704112	0,476945922	0,596489803	0,119543881	0,002212382					S_Shore
cg12465412	0,836554322	0,637413104	-0,199141218	0,002215302	PCNXL2	PCNXL2	NM_014801	Body	N_Shelf
cg14158968	0,771340583	0,88432057	0,112979987	0,002220882	GALNTL4	GALNTL4	NM_198516	Body	
cg04334564	0,940350517	0,820830622	-0,119519895	0,002224385	MLL	MLL	NM_005933	Body	S_Shelf
cg25589929	0,890377197	0,780582777	-0,109794421	0,002225053	GRB2	GRB2;GRB2	NM_002086;NM_203506	5UTR;5UTR	N_Shelf
cg02353916	0,876595763	0,751056099	-0,125539664	0,002227315	LOC285550	LOC285550	NM_001145191	3UTR	
cg14487075	0,041955784	0,158046071	0,116090287	0,002229226	SUV39H2	SUV39H2	NM_024670	5UTR	S_Shore
cg05080849	0,925404965	0,795440315	-0,12996465	0,002232336					N_Shelf
cg13999795	0,945862742	0,834299521	-0,111563221	0,002233277					
cg00168967	0,912908571	0,776871785	-0,136036785	0,002238184	ASPH	ASPH;ASPH	NM_001164750;NM_004318	Body;Body	
cg17524250	0,089623718	0,204560281	0,114936564	0,002238875	C5orf24	C5orf24;C5orf24	NM_001135586;NM_152409	5UTR;5UTR	S_Shore
cg06513043	0,850507326	0,68442156	-0,166085766	0,002241205					N_Shelf
cg08761206	0,87062414	0,723189843	-0,147434298	0,002249535	TRIO	TRIO	NM_007118	Body	
cg09511741	0,101982428	0,205632703	0,103650274	0,0022562	TRPM4	TRPM4	NM_017636	Body	N_Shore
cg26438665	0,864973325	0,757483005	-0,107490321	0,002256344	BTBD7	BTBD7;BTBD7	NM_018167;NM_001002860	5UTR;5UTR	N_Shelf
cg23104549	0,871133496	0,613919781	-0,257213715	0,002263416	AGK	AGK	NM_018238	5UTR	S_Shelf
cg14187813	0,664086698	0,472941614	-0,191145084	0,002270771	FAM178B	FAM178B	NM_001122646	5UTR	N_Shore
cg09513276	0,568770402	0,680696502	0,1119261	0,002270834	BMP7	BMP7	NM_001719	Body	N_Shelf
cg08500128	0,920918935	0,787000841	-0,133918094	0,002271382	MAGOHB	MAGOHB	NM_018048	Body	N_Shelf
cg16594779	0,348359325	0,448812494	0,100453168	0,002273191	PGRMC1	PGRMC1	NM_006667	Body	Island
cg16095464	0,931772555	0,830117752	-0,101654803	0,002277629	SOX5	SOX5	NM_152989	5UTR	
cg11314617	0,105181302	0,222807465	0,117626163	0,002279836	MSC	MSC	NM_005098	TSS1500	S_Shore
cg14314132	0,427270095	0,318808957	-0,108461138	0,002280349	UPF3B	UPF3B;UPF3B;UPF3B;UPF3B	NM_023010;NM_023010;NM_080632;NM_080632	1stExon;5UTR;1stExon;5UTR	Island
cg12873457	0,91021607	0,759812435	-0,150403635	0,002280813					
cg19390658	0,194818079	0,304647748	0,109829669	0,002282048	GARS	GARS	NM_002047	Body	S_Shore
cg12796469	0,904803588	0,717949352	-0,186854237	0,002288994					
cg08653114	0,818218539	0,681455173	-0,136763366	0,002291505					S_Shelf
cg23285459	0,363341738	0,475263267	0,111921529	0,002294149	GNA12	GNA12	NM_007353	Body	Island
cg02698037	0,905405831	0,783949966	-0,121455865	0,002294674	SNORD11	SNORD11;NOP58	NR_003031;NM_015934	TSS1500;Body	
cg14507014	0,910779042	0,794597341	-0,116181701	0,002299529	WNT5A	WNT5A	NM_003392	Body	N_Shelf
cg14480262	0,79165463	0,597319293	-0,194335338	0,002301693	PAPLN	PAPLN	NM_173462	TSS1500	N_Shore
cg26657293	0,943122141	0,825971239	-0,117150902	0,002306128					
cg11130530	0,888371849	0,763646858	-0,12472499	0,002312185	MAD1L1	MAD1L1;MAD1L1;MAD1L1	NM_003550;NM_001013837;NM_001013836	Body;Body;Body	

cg13835168	0,51728026	0,625701805	0,108421545	0,002315306					
cg27065742	0,953665379	0,787358538	-0,16630684	0,002318514					
cg04900179	0,91335864	0,706359082	-0,206999557	0,002318584					N_Shelf
cg16919093	0,954524185	0,854069392	-0,100454793	0,002324212	BRCA1	BRCA1;BRCA1;BRCA1;BRCA1;BRCA1;BRCA1	NM_007298;NM_007300;NM_007297;NR_027676;NM_007294;N	Body;Body;Body;Body;Body;B	
cg22206740	0,885958931	0,782850423	-0,103108508	0,002326479	ADIG	ADIG	NM_001018082	TSS1500	
cg20307885	0,921697799	0,799623099	-0,1220747	0,002327182					S_Shore
cg17154563	0,111995302	0,242211858	0,130216556	0,002334831	PSMC4	PSMC4;PSMC4	NM_153001;NM_006503	Body;Body	S_Shore
cg02470595	0,106081246	0,212736057	0,10665481	0,002336265	HOXC4	HOXC4;HOXC5;HOXC5	NM_014620;NM_018953;NR_003084	5UTR;TSS200;Body	N_Shore
cg14800883	0,108226173	0,231153101	0,122926928	0,002338491	ARPC4	ARPC4;TADA3;TADA3;ARPC4;ARPC4;TADA3;TADA3	NM_001024960;NM_133480;NM_133480;NM_005718;NM_0010	TSS1500;5UTR;1stExon;TSS	Island
cg22367981	0,044096151	0,159519707	0,115423555	0,002339611	DISC1	DISC1;DISC1;DISC1;DISC1;DISC1;DISC1;DISC1;TSNAX-	NM_001012959;NM_018662;NM_001164539;NM_001012957;N	Body;Body;Body;Body;Body;B	
cg15021629	0,892824236	0,713760306	-0,17906393	0,002339978	ARHGEF7	ARHGEF7;ARHGEF7	NM_003899;NM_001113513	3UTR;3UTR	
cg14364815	0,867294265	0,709199849	-0,158094416	0,002343288	FANK1	FANK1	NM_145235	Body	S_Shelf
cg27405177	0,886773765	0,725368896	-0,161404868	0,002346561	GPR123	GPR123	NM_001083909	Body	Island
cg25288464	0,615510836	0,722012025	0,10650119	0,002348495	HSPA8	HSPA8;HSPA8	NM_006597;NM_153201	Body;Body	N_Shore
cg12970830	0,882064283	0,729744615	-0,152319668	0,002358219					
cg18883951	0,672579614	0,786254261	0,113674646	0,002361048	SCNN1A	SCNN1A	NM_001159575	TSS200	
cg11410080	0,854930837	0,682862508	-0,172068329	0,002362515	FBXL20	FBXL20	NM_032875	Body	
cg10592446	0,037242463	0,141005733	0,10376327	0,002373387	CTSO	CTSO	NM_001334	Body	N_Shore
cg27169559	0,064832972	0,168907097	0,104074125	0,002374052					
cg12337005	0,113876425	0,30319725	0,189320826	0,002375985					Island
cg03697708	0,093828144	0,215724431	0,121896288	0,002386175	CACNA1A	CACNA1A;CACNA1A	NM_001127221;NM_001127222	TSS1500;TSS1500	S_Shore
cg17847861	0,685089169	0,568880001	-0,116209168	0,002387899	SPTBN4	SPTBN4;BLVRB	NM_020971;NM_000713	TSS1500;TSS1500	Island
cg13139361	0,820886127	0,695922503	-0,124963624	0,002388751					
cg14058291	0,943917074	0,811704771	-0,132212303	0,00239299	ADARB2	ADARB2	NM_018702	Body	
cg03897244	0,644816654	0,747560041	0,102743387	0,002393203					N_Shelf
cg26015356	0,968871564	0,86591165	-0,102959914	0,002394091	C17orf50	C17orf50	NM_145272	Body	N_Shore
cg05929755	0,642549717	0,751392645	0,108842927	0,002394159					
cg12521910	0,669011786	0,773853838	0,104842052	0,002400431	XAGE3	XAGE3;XAGE3	NM_133179;NM_130776	TSS1500;TSS1500	
cg08409167	0,884705902	0,784524727	-0,100181176	0,002401656	CSNK1G1	CSNK1G1	NM_022048	Body	
cg27502952	0,933495239	0,784528297	-0,148966942	0,002402649	FARP2	FARP2	NM_014808	Body	
cg05491326	0,942971279	0,784328917	-0,158642363	0,002402861	LDB2	LDB2;LDB2	NM_001290;NM_001130834	Body;3UTR	
cg11957481	0,872645994	0,769859058	-0,102786936	0,002403999	FBL	FBL;DYRK1B;DYRK1B;DYRK1B	NM_001436;NM_004714;NM_006483;NM_006484	3UTR;TSS1500;TSS1500;TSS	S_Shore
cg11797265	0,860333951	0,757236205	-0,103097747	0,002404188	ATXN1	ATXN1;ATXN1	NM_001128164;NM_000332	5UTR;5UTR	
cg12905788	0,801161328	0,632843793	-0,168317535	0,002406196	RPL31	RPL31;RPL31;RPL31	NM_001099693;NM_001098577;NM_000993	3UTR;Body;3UTR	
cg14639562	0,934812738	0,798401034	-0,136411704	0,002411308	CENPP	CENPP;ASPN	NM_001012267;NM_017680	Body;3UTR	
cg26890309	0,94131958	0,810622793	-0,130696786	0,002412293	C1orf63	C1orf63	NM_020317	Body	N_Shelf
cg20129827	0,714573576	0,816173398	0,101599823	0,002415934	KIAA1210	KIAA1210	NM_020721	TSS200	Island
cg25754419	0,948770165	0,817457175	-0,13131299	0,002420075					S_Shelf
cg21951543	0,953304265	0,828107808	-0,125196457	0,002420527	SRPK2	SRPK2;SRPK2	NM_182691;NM_182692	Body;Body	
cg19537741	0,775786358	0,655972653	-0,119813705	0,002420823					
cg26453295	0,933789721	0,808032478	-0,125757243	0,002422498	LILRA4	LILRA4	NM_012276	TSS1500	
cg10309580	0,951753924	0,82338913	-0,128364794	0,002425485	FOXJ3	FOXJ3	NM_014947	5UTR	
cg27111588	0,932765835	0,819234687	-0,113531148	0,002427996					S_Shelf
cg18264020	0,741519802	0,61681245	-0,124707352	0,002431808					N_Shore
cg04246253	0,919198469	0,767971643	-0,151226826	0,00243322					N_Shelf
cg09361367	0,936657764	0,835659143	-0,100998621	0,002434301	PRKG1	PRKG1;PRKG1	NM_001098512;NM_006258	Body;Body	S_Shelf
cg20939322	0,880167313	0,655365282	-0,224802031	0,002436226	FERMT2	FERMT2;FERMT2;FERMT2	NM_006832;NM_001134999;NM_001135000	Body;Body;Body	
cg22921027	0,560237885	0,671522345	0,11128446	0,002438953					
cg00167670	0,357094144	0,457734565	0,100640421	0,002439997					
cg04873627	0,857852648	0,709368054	-0,148484594	0,002440987					
cg16417831	0,919961191	0,812850916	-0,107110274	0,002446485	TCP10L	TCP10L	NM_144659	TSS1500	
cg07522918	0,83955137	0,71333762	-0,126213751	0,002456162					N_Shelf
cg13541588	0,701162195	0,813598465	0,11243627	0,002456676	FLJ36000	FLJ36000	NR_027084	TSS200	
cg09297832	0,911466603	0,806685577	-0,104781026	0,002459064					
cg20296327	0,876992238	0,695515638	-0,181476601	0,002463007					
cg10490746	0,925874563	0,758070498	-0,167804065	0,002464676	ACOT11	ACOT11;FAM151A	NM_015547;NM_176782	Body;TSS1500	
cg21194785	0,899702496	0,776011524	-0,123690972	0,002467156	CHEK2	CHEK2;CHEK2;CHEK2	NM_001005735;NM_007194;NM_145862	Body;Body;Body	
cg12314012	0,721892812	0,824782864	0,102890051	0,002468091					
cg08340023	0,914935786	0,803850973	-0,111084813	0,002468643	USP31	USP31	NM_020718	Body	
cg21828197	0,948229176	0,839723131	-0,108506045	0,0024709	ASPSCR1	ASPSCR1	NM_024083	Body	N_Shore
cg26681139	0,546793531	0,650022093	0,103228562	0,00247523	TMEM163	TMEM163	NM_030923	Body	
cg02951717	0,081602796	0,222885863	0,141283067	0,002477739	ZMYM6	ZMYM6	NM_007167	5UTR	N_Shore
cg10409702	0,926092231	0,791907249	-0,134184983	0,002477773					
cg14468508	0,933247403	0,819951625	-0,113295778	0,002481941	BAT2L1	BAT2L1	NM_013318	TSS1500	
cg14165911	0,641647	0,743534538	0,101887538	0,002484419	TRPM3	TRPM3	NM_001007471	Body	
cg16523850	0,107233175	0,238975422	0,131742247	0,002486581					
cg00174252	0,948417143	0,843696734	-0,104720409	0,002491393	ATP5J	ATP5J;ATP5J;ATP5J;ATP5J;ATP5J	NM_001003697;NM_001003703;NM_001003701;NM_00100369	3UTR;3UTR;3UTR;3UTR;3UTR	
cg05495879	0,943141669	0,824259537	-0,118882131	0,002491472	ZNF470	ZNF470	NM_001001668	Body	S_Shelf
cg02595183	0,921313934	0,764857852	-0,156456081	0,002493176	SH3BP4	SH3BP4	NM_014521	5UTR	
cg03313909	0,943157054	0,762975604	-0,18018145	0,002498028					S_Shelf
cg25154075	0,932399935	0,771305985	-0,16109395	0,002503122	MED13L	MED13L	NM_015335	Body	
cg10599633	0,912481328	0,805782182	-0,106699146	0,002506274					S_Shelf
cg23571452	0,87861174	0,753958077	-0,124653663	0,002512457	PAK1IP1	PAK1IP1	NM_017906	Body	S_Shelf
cg02945266	0,738607275	0,631391299	-0,107215976	0,002514115	TOP3B	TOP3B	NM_003935	TSS1500	S_Shore
cg03314530	0,932938074	0,818036539	-0,114901535	0,002516101	OPCML	OPCML	NM_001012393	Body	
cg22696167	0,138785743	0,245533415	0,106747672	0,002521745					N_Shore
cg05848884	0,784784979	0,674744795	-0,110040184	0,002524282					
cg20988109	0,098894715	0,202453184	0,103558469	0,002524513	LOC492303	LOC492303;ZNF326;ZNF326	NR_002830;NM_182976;NM_182975	TSS1500;Body;Body	Island
cg08506369	0,821582504	0,642790722	-0,178791782	0,002528091					S_Shore
cg03604388	0,873165633	0,661472794	-0,211692838	0,002529014	MED12L	MED12L	NM_053002	3UTR	
cg16644344	0,701228944	0,807750691	0,106521747	0,002539112	PRKCZ	PRKCZ;PRKCZ;PRKCZ	NM_001033581;NM_001033582;NM_002744	Body;Body;Body	
cg04707327	0,145083203	0,247522134	0,102438931	0,00254602	KLHDC1	KLHDC1	NM_172193	TSS1500	N_Shore
cg16881398	0,909487956	0,769006318	-0,140481638	0,002547722	TEX11	TEX11;TEX11	NM_031276;NM_001003811	TSS200;TSS200	
cg16521213	0,943722344	0,798952594	-0,14476975	0,002548286	ACP1	ACP1;ACP1;ACP1	NM_004300;NM_007099;NR_024080	3UTR;3UTR;Body	
cg09769647	0,861153748	0,718925232	-0,142228516	0,002556866	HK2	HK2	NM_000189	TSS200	N_Shore
cg26844633	0,077890835	0,184135328	0,106244493	0,00256116					
cg04947850	0,965691541	0,855494545	-0,110196996	0,002562517	TBC1D14	TBC1D14;TBC1D14	NM_020773;NM_001113361	5UTR;5UTR	N_Shore
cg24506782	0,793860302	0,658643551	-0,135216751	0,002569349	KSR2	KSR2	NM_173598	Body	N_Shelf
cg08817693	0,285849993	0,39308198	0,107231987	0,002571338	C12orf23	C12orf23	NM_152261	TSS1500	N_Shore
cg16661609	0,922302919	0,810821506	-0,111481413	0,002572669	LILRB4	LILRB4;LILRB4	NM_006847;NM_001081438	TSS1500;TSS1500	
cg02869156	0,902058192	0,781223641	-0,120834551	0,002576661	ELF5	ELF5	NM_001422	TSS1500	
cg07929406	0,228614236	0,392839013	0,164224777	0,002589591	SOX3	SOX3	NM_005634	TSS200	Island
cg03276149	0,059331845	0,161221065	0,10188922	0,002590883					
cg19820601	0,849798589	0,710108752	-0,139689837	0,002597423					
cg18191494	0,946922363	0,842259279	-0,104663085	0,002598582	HTR7P	HTR7P	NR_002774	Body	S_Shelf
cg04276396	0,157525434	0,296415924	0,13889049	0,002599301					
cg18982923	0,900507598	0,722956191	-0,177551407	0,002605589	OAT	OAT	NM_000274	3UTR	
cg22254103	0,886961849	0,780313161	-0,106648688	0,002607021					Island
cg21271026	0,83443986	0,684689748	-0,149750113	0,002608253	SLC9A3	SLC9A3	NM_004174	Body	Island
cg10185119	0,218380898	0,323574647	0,105193749	0,002612836	SATB2	SATB2	NM_015265	TSS200	Island
cg19088553	0,91665135	0,815904558	-0,100746792	0,002618684	GRIK2	GRIK2;GRIK2;GRIK2	NM_001166247;NM_175768;NM_021956	Body;Body;Body	
cg08909756	0,91371846	0,752256927	-0,161461533	0,002619257	ALDH1L2	ALDH1L2;ALDH1L2	NR_027752;NM_001034173	TSS1500;TSS1500	S_Shore
cg14368817	0,931756297	0,796525617	-0,13523068	0,002619406	TTC3L	TTC3L	NR_030737	TSS1500	
cg11736230	0,084252473	0,188343486	0,104091014	0,002620041	PPP1R13B	PPP1R13B	NM_015316	Body	N_Shore
cg15865150	0,360739667	0,468098697	0,107359031	0,002620181	AMIGO3	AMIGO3;AMIGO3;RNF123	NM_198722;NM_198722;NM_022064	5UTR;1stExon;Body	Island
cg09289359	0,921049924	0,781602152	-0,139447772	0,002629455					N_Shelf
cg00372940	0,045079317	0,14563697	0,100557653	0,002631926	WDR47	WDR47;WDR47;WDR47	NM_001142551;NM_001142550;NM_014969	5UTR;5UTR;5UTR	N_Shore
cg22356063	0,573815173	0,449427219	-0,124387954	0,002634197	FAM194B	FAM194B	NM_182542	Body	
cg00587523	0,758847943	0,642256301	-0,116591642	0,002639243	SKAP1	SKAP1;SKAP1	NM_001075099;NM_003726	3UTR;3UTR	
cg26688816	0,919193384	0,808112103	-0,111081281	0,002645082	ATP6V1E2	ATP6V1E2	NM_080653	5UTR	
cg07121312	0,928433226	0,795382604	-0,133050621	0,002647936	TBC1D16	TBC1D16	NM_019020	Body	S_Shelf
cg16974308	0,956536933	0,836925719	-0,119611214	0,002648454	PAG1	PAG1	NM_018440	5UTR	
cg19409172	0,077071352	0,193423272	0,11635192	0,002649757	ANKRD50	ANKRD50;ANKRD50	NM_020337;NM_001167882	5UTR;5UTR	N_Shore
cg03980224	0,078918136	0,18360174	0,104683604	0,002653323	RAI14	RAI14;RAI14;RAI14;RAI14;RAI14;RAI14	NM_001145523;NM_015577;NM_001145522;NM_001145520;N	TSS1500;5UTR;5UTR;5UTR;5	
cg09739850	0,911137678	0,780308478	-0,1308292	0,002653779					
cg20086916	0,944945315	0,806354557	-0,138590758	0,002657499	FOXK1	FOXK1	NM_001037165	Body	S_Shore
cg07586235	0,075497081	0,196681292	0,121184211	0,002658907					
cg10951976	0,69488654	0,56773574	-0,127150799	0,002661653					S_Shelf
cg09133692	0,678743517	0,788603616	0,109860099	0,002665753	MMADHC	MMADHC	NM_015702	Body	
cg17444479	0,894581257	0,7581616	-0,136419657	0,002666681	INPP5A	INPP5A	NM_005539	Body	
cg04303509	0,083257525	0,207414736	0,124157211	0,00266684	ZNF792	ZNF792	NM_175872	Body	N_Shore
cg08263106	0,938692729	0,826660889	-0,11203184	0,002667946	GRID2	GRID2	NM_001510	Body	
cg09640621	0,582488578	0,686676017	0,10418744	0,002668795	NPS	NPS	NM_001030013	TSS1500	
cg14958978	0,902731846	0,769646337	-0,133085509	0,002676661	ZNF385B	ZNF385B;ZNF385B;ZNF385B	NM_001113398;NM_152520;NM_001113397	TSS1500;Body;Body	
cg06394876	0,703066127	0,837631013	0,134564887	0,002682966					
cg12744031	0,304715868	0,195841491	-0,108874377	0,002687357					Island
cg13783167	0,68807951	0,495259347	-0,192820163	0,002688307	DLG3	DLG3;DLG3;DLG3	NM_021120;NM_020730;NM_001166278	Body;Body;5UTR	S_Shelf
cg01113811	0,227032893	0,375380238	0,148347345	0,002690871	MAML2	MAML2	NM_032427	Body	
cg07519721	0,054980423	0,165513314	0,110532892	0,002691958	SPG21	SPG21;SPG21;SPG21;SPG21	NM_001127889;NM_001127889;NM_001127890;NM_016630	5UTR;1stExon;5UTR;5UTR	N_Shore
cg22068371	0,92086667	0,802075521	-0,118791149	0,002692491	BTN3A1	BTN3A1;BTN3A1;BTN3A1;BTN3A1	NM_001145008;NM_007048;NM_001145009;NM_194441	Body;Body;Body;Body	
cg27124847	0,305221836	0,196017433	-0,109204402	0,00269297	RBM3	RBM3	NM_006743	5UTR	Island
cg22681495	0,866622443	0,725475121	-0,141147322	0,002693023	AKAP13	AKAP13;AKAP13	NM_007200;NM_006738	Body;Body	
cg03022926	0,909316708	0,794300848	-0,115015861	0,002693439	TC2N	TC2N;TC2N;TC2N	NM_152332;NM_001128595;NM_001128596	3UTR;3UTR;3UTR	
cg03759001	0,820500952	0,682639705	-0,137861247	0,002697655					
cg22401505	0,061979515	0,182845858	0,120866343	0,002701098	RWDD1	RWDD1;RWDD1;RWDD1	NM_015952;NM_001007464;NM_016104	Body;5UTR;5UTR	S_Shore
cg11881146	0,914148896	0,813764832	-0,100384064	0,002704188	UNC119	UNC119;UNC119;PIGS	NM_054035;NM_005148;NM_033198	TSS1500;TSS1500;3UTR	S_Shore
cg00243281	0,671133941	0,779437428	0,108303487	0,002705106	KCNQ1	KCNQ1;KCNQ1	NM_000218;NM_181798	Body;Body	S_Shore
cg11264635	0,635354335	0,736717362	0,101363027	0,00270992	SLC1A6	SLC1A6	NM_005071	TSS200	
cg03362840	0,094949876	0,197023075	0,102073199	0,002712499	CALD1	CALD1;CALD1;CALD1;CALD1;CALD1;CALD1	NM_033157;NM_033157;NM_033138;NM_004342;NM_004342;N	5UTR;1stExon;1stExon;1stEx	
cg11015505	0,070916626	0,190454111	0,119537485	0,002720261	EHD1	EHD1	NM_006795	1stExon	Island
cg14479709	0,601421673	0,704835143	0,10341347	0,002725477					S_Shelf
cg05451078	0,522447274	0,624205046	0,101757772	0,002725624	ZBTB3	ZBTB3	NM_024784	3UTR	N_Shelf
cg01563888	0,862984195	0,716726986	-0,146257209	0,002731299	RHAG	RHAG	NM_000324	Body	
cg18792689	0,855706234	0,743474709	-0,112231525	0,002734035	FNDC7	FNDC7	NM_001144937	TSS1500	
cg23361916	0,926462804	0,821779181	-0,104683623	0,002735396	C6orf163	C6orf163	NM_001010868	Body	
cg13681752	0,934672293	0,82449009	-0,110182203	0,002740509					
cg11067885	0,799831588	0,690485518	-0,109346069	0,002743254	RPGRIP1	RPGRIP1	NM_020366	Body	N_Shelf
cg10792987	0,663393311	0,79515817	0,13176486	0,002746487	ELL	ELL	NM_006532	Body	S_Shelf
cg14818176	0,773053003	0,62715337	-0,145899633	0,002749287	HN1	HN1;HN1;HN1	NM_001002033;NM_016185;NM_001002032	5UTR;Body;Body	N_Shore
cg18867627	0,92117039	0,796500211	-0,124670179	0,002749491	ATP9A	ATP9A	NM_006045	Body	
cg14832266	0,789702819	0,597161622	-0,192541197	0,00275427					N_Shore
cg16701893	0,952488357	0,798548877	-0,15393948	0,002755564	PBX1	PBX1	NM_002585	Body	
cg22314759	0,101506439	0,254147806	0,152641367	0,002757941					
cg15260508	0,89703056	0,719878525	-0,177152035	0,002758153	NAA11	NAA11	NM_032693	3UTR	
cg13598232	0,802047577	0,611164095	-0,190883482	0,00277142	ZBTB22	ZBTB22;ZBTB22	NM_001145338;NM_005453	5UTR;5UTR	N_Shore
cg02398359	0,905091908	0,78271849	-0,122373418	0,002771466	KIAA0922	KIAA0922;KIAA0922	NM_015196;NM_001131007	Body;Body	
cg08075475	0,966325796	0,852244272	-0,114081524	0,002773139	WHSC2	WHSC2	NM_005663	Body	S_Shelf
cg25281584	0,839132615	0,715727695	-0,123404921	0,002775377	AGBL4	AGBL4	NM_032785	Body	
cg14367306	0,933732406	0,787821026	-0,14591138	0,002782052					
cg03018678	0,924027142	0,800839976	-0,123187165	0,002789046					
cg00767135	0,941614235	0,82654615	-0,115068085	0,002789549	ODZ3	ODZ3	NM_001080477	3UTR	N_Shelf
cg06688803	0,77513357	0,635662518	-0,139471052	0,002791485	CLPTM1	CLPTM1	NM_001294	TSS1500	N_Shore
cg12209615	0,921719903	0,754642525	-0,167077378	0,00279987					
cg24671886	0,210681871	0,343098345	0,132416474	0,002803631	CAPZB	CAPZB	NM_004930	Body	
cg22174337	0,80336582	0,661535804	-0,141830016	0,002804116	KCNAB1	KCNAB1;KCNAB1	NM_003471;NM_172160	Body;Body	N_Shelf
cg02663317	0,781495364	0,663313782	-0,118181582	0,002805069	STK38L	STK38L	NM_015000	5UTR	
cg08672203	0,93847928	0,814302299	-0,124176981	0,00280763	PPAP2A	PPAP2A;PPAP2A	NM_176895;NM_003711	Body;Body	
cg06636156	0,854467844	0,692658241	-0,161809603	0,002807965	MTCH1	MTCH1	NM_014341	Body	N_Shelf
cg11425349	0,882675837	0,758831895	-0,123843942	0,002815684	MORF4L2	MORF4L2;MORF4L2;MORF4L2;MORF4L2;MORF4L2;MORF4L2;MORF	NM_001142419;NM_001142432;NM_001142427;NM_00114243	TSS1500;TSS1500;TSS1500	S_Shore
cg11566521	0,071771875	0,214200682	0,142428807	0,002818457					
cg20005629	0,774106171	0,605715114	-0,168391057	0,002821089	PTPRN2	PTPRN2;PTPRN2;PTPRN2	NM_002847;NM_130842;NM_130843	Body;Body;Body	N_Shore
cg10246038	0,915686193	0,797025826	-0,118660367	0,002821853	FAM82A1	FAM82A1;FAM82A1;FAM82A1;FAM82A1	NM_001170793;NM_001170791;NM_144713;NM_001170792	3UTR;3UTR;Body;3UTR	
cg03132556	0,046858851	0,160785749	0,113926898	0,002826222	HOXC11	HOXC11	NM_014212	TSS200	N_Shore
cg12018865	0,823970694	0,582500582	-0,241470112	0,002826256	C2orf58	C2orf58	NR_027252	Body	
cg04615668	0,823215988	0,722767251	-0,100448738	0,002828748	CTNND2	CTNND2	NM_001332	Body	
cg18463607	0,636243819	0,747243143	0,110999323	0,002830106	EXOC1	EXOC1;EXOC1;EXOC1	NM_018261;NM_178237;NM_001024924	TSS1500;TSS1500;TSS1500	N_Shore
cg06103928	0,901668358	0,776847467	-0,124820891	0,002835211	DLC1	DLC1;DLC1;DLC1	NM_006094;NM_182643;NM_001164271	Body;Body;TSS1500	
cg23502966	0,903300654	0,798158575	-0,105142079	0,002839468	TFRC	TFRC;TFRC	NM_001128148;NM_003234	Body;Body	
cg22888311	0,884108496	0,762379861	-0,121728634	0,002848656	PAWR	PAWR	NM_002583	Body	
cg11932644	0,927836129	0,815178692	-0,112657437	0,002850578					
cg19791043	0,759919578	0,863633036	0,103713458	0,002852201	LOC146336	LOC146336	NR_027242	Body	Island

cg27277104	0,915125666	0,788539523	-0,126586143	0,002852863					
cg16058430	0,91637345	0,71060652	-0,20576693	0,002860119	C1orf201	C1orf201	NM_178122	TSS1500	
cg04546557	0,770385973	0,56845128	-0,201934693	0,002860833					N_Shelf
cg13449377	0,26316785	0,36837821	0,10521036	0,002864885	DLX6AS	DLX6AS	NR_015448	Body	N_Shore
cg26198700	0,083319354	0,301252136	0,217932782	0,002866833					
cg01987536	0,651363798	0,754723787	0,10335999	0,002868288	AK7	AK7	NM_152327	Body	N_Shore
cg22641280	0,691619065	0,589944418	-0,101674647	0,002869212	TOX	TOX	NM_014729	Body	N_Shelf
cg21566771	0,605530744	0,721273062	0,115742318	0,002873871	RNF144A	RNF144A	NM_014746	Body	N_Shelf
cg26484090	0,917025401	0,782302259	-0,134723142	0,002876972	USP45	USP45	NM_001080481	5UTR	N_Shelf
cg18258482	0,605045348	0,715664733	0,110619386	0,002877791	ROS1	ROS1	NM_002944	Body	
cg04743106	0,931752115	0,785334944	-0,14641717	0,002887118	PALLD	PALLD;PALLD;PALLD	NM_001166109;NM_016081;NM_001166108	5UTR;Body;Body	
cg00063369	0,578554769	0,687285417	0,108730648	0,002900305					
cg08152588	0,902789633	0,722990872	-0,179798761	0,002905862	C14orf50	C14orf50	NM_172365	TSS1500	N_Shore
cg15963036	0,397828395	0,499024478	0,101196083	0,002908356	C3orf21	C3orf21	NM_152531	Body	
cg03751149	0,158221215	0,277772121	0,119550906	0,002913129					
cg13823415	0,903127449	0,776366489	-0,126760961	0,002916694	TBXAS1	TBXAS1;TBXAS1	NM_001130966;NM_001166254	5UTR;5UTR	
cg06314748	0,898341671	0,772907635	-0,125434036	0,002925271					N_Shelf
cg02350090	0,836652318	0,717507028	-0,11914529	0,002930984					S_Shore
cg05788138	0,521810797	0,641517827	0,119707031	0,002942027	OR5L1	OR5L1	NM_001004738	TSS1500	
cg18929316	0,906976449	0,770791206	-0,136185243	0,00294269	CBX1	CBX1;CBX1	NM_006807;NM_001127228	5UTR;5UTR	N_Shore
cg13414542	0,937637116	0,83672991	-0,100907207	0,002944196					
cg18430295	0,092367108	0,245156122	0,152789014	0,002944325	NRN1	NRN1	NM_016588	TSS1500	N_Shore
cg06835786	0,918154206	0,801938545	-0,116215662	0,002945553					
cg02262585	0,200175988	0,34061714	0,140441152	0,002949081	TESK1	TESK1	NM_006285	TSS1500	Island
cg13894444	0,933593082	0,83059907	-0,102994012	0,002949443	LEPREL1	LEPREL1;LEPREL1	NM_001134418;NM_018192	5UTR;Body	
cg26788616	0,897198987	0,777554487	-0,1196445	0,002951891	CLEC16A	CLEC16A	NM_015226	Body	
cg01602621	0,360079718	0,473344427	0,113264709	0,002961216	ACP5	ACP5;ACP5;ACP5;ACP5	NM_001111034;NM_001611;NM_001111035;NM_001111036	TSS1500;TSS1500;TSS200;	S_Shore
cg07829001	0,965422577	0,860484428	-0,104938149	0,002966743	NPHP4	NPHP4	NM_015102	Body	
cg16726869	0,728925676	0,834745199	0,105819523	0,002968209					
cg10840144	0,93372995	0,804840397	-0,128889553	0,002971517					
cg06814136	0,926958206	0,826896422	-0,100061785	0,002975262	CRYBG3	CRYBG3;MINA;MINA;MINA	NM_153605;NM_153182;NM_001042533;NM_032778	3UTR;3UTR;3UTR;3UTR	
cg00729370	0,505663548	0,372804109	-0,132859439	0,002975464					N_Shore
cg02837279	0,147420393	0,250694483	0,10327409	0,002980112	ZNF252	ZNF252;C8orf77	NR_023392;NR_026974	Body;TSS1500	N_Shore
cg10358939	0,928032697	0,786380578	-0,14165212	0,002986056	HRASLS	HRASLS	NM_020386	Body	
cg08360638	0,765742101	0,868578917	0,102836816	0,002987466					
cg16073467	0,924438151	0,786249986	-0,138188165	0,002995926	GABBR1	GABBR1;GABBR1;GABBR1	NM_021904;NM_021903;NM_001470	Body;Body;Body	
cg13821227	0,777450228	0,669385485	-0,108064743	0,002999916	LOC339674	LOC339674	NR_024355	TSS1500	S_Shelf
cg17486644	0,956729315	0,844295754	-0,11243356	0,003000345	RADIL	RADIL;PAPOLB;PAPOLB	NM_018059;NM_020144;NM_020144	Body;1stExon;3UTR	N_Shore
cg04031344	0,694497609	0,805254855	0,110757245	0,003001483					
cg08128133	0,921504491	0,816217624	-0,105286867	0,003001705	FRAS1	FRAS1;FRAS1	NM_025074;NM_001166133	Body;Body	
cg11394276	0,908879185	0,769118084	-0,139761101	0,003016916	GPAM	GPAM	NM_020918	3UTR	
cg13580749	0,820305029	0,675907816	-0,144397213	0,003018747					
cg01138728	0,069848969	0,171307267	0,101458297	0,003019911	LMTK2	LMTK2	NM_014916	Body	S_Shore
cg21336300	0,949050847	0,796722937	-0,15232791	0,003023474	LRMP	LRMP	NM_006152	TSS1500	
cg17379405	0,921245777	0,784333252	-0,136912525	0,00302522					S_Shore
cg15015070	0,393583494	0,274448849	-0,119134645	0,003025395	GIPC2	GIPC2	NM_017655	Body	
cg17934775	0,118789259	0,244068496	0,125279237	0,003026962	CHD2	CHD2;CHD2	NM_001271;NM_001042572	Body;Body	S_Shelf
cg13871565	0,902934354	0,801740623	-0,101193731	0,003028422					
cg15387278	0,723488519	0,827950295	0,104461776	0,003032872	C1orf127	C1orf127	NM_001170754	TSS1500	
cg20670404	0,895447227	0,775502594	-0,119944632	0,003033459					S_Shelf
cg15138712	0,970497001	0,855082714	-0,115414287	0,003043059	CACHD1	CACHD1	NM_020925	Body	
cg17193551	0,954946793	0,771094387	-0,183852405	0,003044517	FAM49B	FAM49B	NM_016623	5UTR	
cg17894577	0,717838192	0,530225988	-0,187612204	0,003049967	ITGA8	ITGA8	NM_003638	Body	
cg16811129	0,72106829	0,527048833	-0,194019457	0,003052138	ARHGEF10	ARHGEF10	NM_014629	Body	Island
cg22803549	0,788722893	0,665082878	-0,123640015	0,003056949	LGMN	LGMN;LGMN	NM_001008530;NM_005606	TSS1500;TSS1500	
cg07814731	0,849201031	0,714988772	-0,134212259	0,003057521	TSHZ2	TSHZ2	NM_173485	TSS1500	N_Shelf
cg06896770	0,141338007	0,244409267	0,10307126	0,003058571	DOCK7	DOCK7	NM_033407	Body	N_Shore
cg02945866	0,89255865	0,783265291	-0,109293358	0,003059012	PTPRN2	PTPRN2;PTPRN2;PTPRN2	NM_002847;NM_130842;NM_130843	Body;Body;Body	N_Shelf
cg03457472	0,589825463	0,691737989	0,101912527	0,003063454	SDK1	SDK1	NM_152744	TSS1500	N_Shore
cg08677657	0,073690529	0,188967071	0,115276542	0,003068887					N_Shelf
cg10955972	0,940516525	0,742430021	-0,198086503	0,003074644	MAPT	MAPT;MAPT;MAPT;MAPT;LOC100130148;MAPT;MAPT	NM_001123067;NM_016835;NM_016834;NM_001123066;NR_0	5UTR;5UTR;5UTR;5UTR;Body;	S_Shore
cg22179018	0,811423922	0,635965436	-0,175458485	0,003075914	ZNF354B	ZNF354B	NM_058230	TSS1500	N_Shore
cg18949056	0,850371651	0,700830841	-0,14954081	0,003086939	SORBS1	SORBS1;SORBS1	NM_015385;NM_024991	5UTR;5UTR	N_Shelf
cg20746815	0,901326917	0,786329221	-0,114997697	0,003088524	ASB8	ASB8	NM_024095	Body	N_Shelf
cg04605148	0,910328281	0,702812483	-0,207515798	0,003098275	SLC16A9	SLC16A9	NM_194298	5UTR	
cg01312412	0,892706625	0,749609745	-0,143096881	0,003099027					
cg03429968	0,933962477	0,809802182	-0,124160294	0,00309943	EPB41	EPB41;EPB41;EPB41;EPB41;EPB41;EPB41	NM_203342;NM_203343;NM_004437;NM_001166006;NM_0011	5UTR;5UTR;5UTR;5UTR;5UTR	S_Shelf
cg27551625	0,939466677	0,772800448	-0,166666229	0,003101432					
cg13125072	0,904747819	0,771491269	-0,13325655	0,003102464	DNAJC7	DNAJC7;DNAJC7;DNAJC7	NR_029431;NM_001144766;NM_003315	Body;Body;Body	
cg10414747	0,940430232	0,7744337	-0,165996532	0,003125628	FDX1	FDX1	NM_004109	TSS1500	N_Shore
cg16563203	0,939763305	0,818173786	-0,121589519	0,003127667					N_Shelf
cg00091760	0,718389685	0,861926914	0,143537229	0,003131746					N_Shelf
cg09153659	0,876335976	0,650643525	-0,225692451	0,003138466					
cg02483029	0,746429292	0,634256649	-0,112172643	0,003140672	KIAA0146	KIAA0146	NM_001080394	Body	
cg07747559	0,924553584	0,80435296	-0,120200624	0,003150737	NAAA	NAAA;NAAA	NM_001042402;NM_014435	Body;Body	N_Shelf
cg11938951	0,123588494	0,278378479	0,154789984	0,003152044	LOC222699	LOC222699	NR_002936	TSS1500	S_Shore
cg04743609	0,071031028	0,176028011	0,104996983	0,003155313	TM4SF18	TM4SF18	NM_138786	TSS1500	
cg12618806	0,724796482	0,830192101	0,105395618	0,003160196	DLGAP2	DLGAP2	NM_004745	TSS200	
cg16635874	0,947156675	0,804298873	-0,142857802	0,003162028	UBE2E3	UBE2E3;UBE2E3	NM_182678;NM_006357	Body;Body	
cg23997263	0,093902137	0,222455141	0,128553004	0,003162153	FUT9	FUT9	NM_006581	TSS1500	N_Shore
cg13515351	0,966543698	0,831386036	-0,135157662	0,003162516					
cg05584990	0,922656145	0,809411789	-0,113244356	0,003162757					
cg00160902	0,648207714	0,748360498	0,100152784	0,003166016					
cg01812403	0,880253348	0,761671172	-0,118582176	0,003166315					N_Shelf
cg20021572	0,912208259	0,790745934	-0,121462325	0,003180582	LARGE	LARGE;LARGE	NM_133642;NM_004737	5UTR;5UTR	
cg05125455	0,802805231	0,685368284	-0,117436947	0,003182718	CADM1	CADM1;CADM1	NM_001098517;NM_014333	Body;Body	
cg20535931	0,838557107	0,717504502	-0,121052605	0,00318697	OCM2	OCM2	NM_006188	Body	
cg04690319	0,85646121	0,711770991	-0,144690218	0,003193388	ZW10	ZW10	NM_004724	3UTR	
cg15582794	0,380648676	0,254365787	-0,12628289	0,003196414	BEX4	BEX4;BEX4	NM_001080425;NM_001127688	5UTR;5UTR	Island
cg02605800	0,902170027	0,672548546	-0,229621481	0,003197754	SBDS	SBDS	NM_016038	Body	N_Shelf
cg26891237	0,730132901	0,842939711	0,112806809	0,003199406					
cg04352495	0,950020148	0,760097129	-0,18992302	0,003220596					S_Shelf
cg08531017	0,922064798	0,811279102	-0,110785696	0,003222122	DSCAML1	DSCAML1	NM_020693	Body	Island
cg26224473	0,842679532	0,726642695	-0,116036837	0,003225788	PER4	PER4	NR_002790	TSS1500	
cg09910611	0,940183431	0,836885525	-0,103297906	0,003231767					
cg13726313	0,890449089	0,734620026	-0,155829062	0,003234909	FOXP1	FOXP1	NM_032682	Body	
cg18199386	0,897835839	0,777583373	-0,120252466	0,00323497	KPRP	KPRP	NM_001025231	TSS1500	N_Shelf
cg04439376	0,100679197	0,222811759	0,122132563	0,003236859	TMEM87B	TMEM87B	NM_032824	TSS1500	Island
cg19135761	0,086896908	0,216906809	0,1300099	0,003238544	WDR8	WDR8	NM_017818	TSS1500	Island
cg17749509	0,855495047	0,713045307	-0,14244974	0,003245821	SLC16A4	SLC16A4	NM_004696	TSS1500	
cg04537185	0,65179534	0,755185068	0,103389729	0,003246936	TMPRSS3	TMPRSS3;TMPRSS3;TMPRSS3	NM_024022;NR_027348;NM_032405	Body;TSS1500;Body	
cg11744861	0,851639975	0,728363478	-0,123276497	0,003248493	ABCC4	ABCC4;ABCC4	NM_001105515;NM_005845	Body;Body	
cg06770082	0,599926292	0,703953171	0,10402688	0,003249314					
cg05815404	0,668825141	0,771593492	0,102768351	0,003256001	RPTOR	RPTOR;RPTOR	NM_001163034;NM_020761	Body;Body	
cg23266797	0,912161265	0,746828033	-0,165333231	0,003258199	MOG	MOG;MOG;MOG;MOG;MOG;MOG;MOG;MOG;MOG;MOG	NM_206813;NM_001170418;NM_206814;NM_206811;NM_2068	3UTR;Body;3UTR;Body;Body;	
cg05799058	0,903354171	0,788033388	-0,115320783	0,003258349					S_Shelf
cg25273602	0,175835193	0,305124088	0,129288895	0,003262162	YTHDF2	YTHDF2	NM_016258	TSS1500	N_Shore
cg12482564	0,823537985	0,644759033	-0,178778952	0,003268326					
cg21280914	0,936815534	0,722109315	-0,214706219	0,003269414	USP14	USP14;USP14	NM_001037334;NM_005151	3UTR;3UTR	
cg21437722	0,775695779	0,592532756	-0,183163022	0,003271557	C17orf63	C17orf63;C17orf63	NM_018182;NM_001077498	3UTR;3UTR	
cg13487918	0,305458638	0,424556866	0,119098229	0,003276417	PLCD1	PLCD1;PLCD1;PLCD1	NM_001130964;NR_024071;NM_006225	TSS1500;TSS1500;Body	S_Shore
cg07029980	0,101768498	0,213410429	0,111641931	0,003278416					
cg15668468	0,111360402	0,217581432	0,10622103	0,003278861	VAX1	VAX1;VAX1	NM_001112704;NM_199131	3UTR;Body	N_Shore
cg25406374	0,194289834	0,351651734	0,1573619	0,003289623					
cg14289114	0,939842233	0,83809985	-0,101742383	0,003294974	ZNF673	ZNF673;ZNF673;ZNF673;ZNF673	NM_001129898;NM_001129900;NM_001129899;NM_017776	5UTR;5UTR;5UTR;5UTR	S_Shelf
cg02133456	0,872802192	0,748152871	-0,124649321	0,00329648					
cg11423701	0,808152493	0,664076825	-0,144075668	0,003299206	GJA5	GJA5	NM_005266	TSS1500	
cg08023852	0,92848728	0,80348601	-0,125001271	0,003307657	MAGI1	MAGI1;MAGI1;MAGI1	NM_015520;NM_001033057;NM_004742	Body;Body;Body	
cg18691565	0,114476891	0,22529893	0,11082204	0,003309315	ACBD5	ACBD5;ACBD5	NR_024150;NM_001042473	TSS1500;TSS1500	Island
cg26942580	0,931183437	0,780537517	-0,150645921	0,003310026	C15orf41	C15orf41	NM_001130010	Body	
cg23538718	0,201152026	0,313314793	0,112162767	0,003310479	SLC9A9	SLC9A9	NM_173653	1stExon	
cg22920418	0,922443231	0,811805485	-0,110637747	0,003317819	RPS5	RPS5	NM_001009	Body	S_Shelf
cg25844471	0,965525103	0,865010408	-0,100514695	0,003322594	C10orf67	C10orf67	NM_153714	TSS1500	
cg02067994	0,835572194	0,723922835	-0,111649359	0,003323221					
cg03563100	0,105017178	0,207481034	0,102463855	0,003325277	CACNB4	CACNB4;CACNB4;CACNB4	NM_000726;NM_001145798;NM_001005746	Body;Body;Body	N_Shore
cg05213267	0,859141057	0,732165189	-0,126975868	0,003325868	DIP2C	DIP2C	NM_014974	Body	N_Shore
cg13122448	0,864544491	0,74721981	-0,117324681	0,003327456					
cg06898293	0,67599417	0,776291755	0,100297585	0,003332424					
cg02947374	0,634494407	0,745904604	0,111410198	0,003333994					N_Shore
cg19691410	0,806608189	0,669420689	-0,1371875	0,00333656					N_Shelf
cg20454179	0,898245434	0,781944441	-0,116300993	0,003337757					S_Shelf
cg24506197	0,765825355	0,610885807	-0,154939548	0,003338275	PTPRN2	PTPRN2;PTPRN2;PTPRN2	NM_002847;NM_130842;NM_130843	Body;Body;Body	S_Shelf
cg23369647	0,880851964	0,764472867	-0,116379097	0,003346763	SCML4	SCML4	NM_198081	Body	
cg20256216	0,913165556	0,767994761	-0,145170796	0,003360816	ARPC1A	ARPC1A	NM_006409	5UTR	S_Shelf
cg15481858	0,872704841	0,71360452	-0,159100321	0,00336091	TP53BP2	TP53BP2;TP53BP2	NM_001031685;NM_005426	Body;Body	
cg07287345	0,926278137	0,821343218	-0,104934919	0,003378152	SLC2A13	SLC2A13	NM_052885	Body	
cg07589273	0,907620997	0,801405448	-0,106215549	0,003379384	CMTM4	CMTM4;CMTM4	NM_178818;NM_181521	Body;3UTR	
cg06899186	0,12754664	0,230751828	0,103205188	0,003379911	CUL1	CUL1	NM_003592	TSS1500	N_Shore
cg11286063	0,114483177	0,234286079	0,119802901	0,003381954	PCDHB18	PCDHB18	NR_001281	TSS200	N_Shore
cg25721732	0,907150072	0,77192283	-0,135227242	0,003383669	DCTN1	DCTN1;DCTN1;DCTN1;DCTN1	NM_001135040;NM_004082;NM_001135041;NM_023019	Body;Body;TSS1500;TSS150	
cg14618583	0,882346655	0,741142432	-0,141204223	0,003385328	FAM120B	FAM120B	NM_032448	Body	N_Shelf
cg20694425	0,940056326	0,803828103	-0,136228222	0,00339276					
cg12715569	0,082029968	0,183542092	0,101512124	0,003396181					
cg16210719	0,61173735	0,721055617	0,109318267	0,003397866					
cg12343338	0,907831243	0,748359514	-0,159471729	0,003403798	NDUFS6	NDUFS6	NM_004553	Body	
cg04735437	0,819923115	0,681622408	-0,138300707	0,003407267	MUC12	MUC12	NM_001164462	Body	
cg19851526	0,820050481	0,690330371	-0,12972011	0,003408089					
cg11259254	0,943853966	0,784303408	-0,159550558	0,00340843	SGMS1	SGMS1	NM_147156	5UTR	
cg07261419	0,13359997	0,24620819	0,11260822	0,003415101	ETV2	ETV2	NM_014209	TSS200	N_Shore
cg17691233	0,697268772	0,835823625	0,138554853	0,003423958					
cg13574744	0,179175151	0,308728911	0,129553759	0,003426908	NFIC	NFIC;NFIC	NM_005597;NM_205843	Body;Body	Island
cg18198301	0,914807594	0,723287364	-0,19152023	0,003430452					
cg23630869	0,181130168	0,316692042	0,135561873	0,003431659					
cg13877482	0,184143715	0,300989891	0,116846176	0,003437965	LHX4	LHX4	NM_033343	Body	Island
cg15570656	0,666664499	0,772488659	0,10582416	0,003440021					
cg22525743	0,855793815	0,71433133	-0,141462485	0,003440668	FBL	FBL;DYRK1B;DYRK1B;DYRK1B	NM_001436;NM_004714;NM_006483;NM_006484	3UTR;TSS1500;TSS1500;TSS	S_Shore
cg20925726	0,898392346	0,749897501	-0,148494845	0,003452139	CTNNB1	CTNNB1;CTNNB1;CTNNB1	NM_001904;NM_001098209;NM_001098210	5UTR;5UTR;5UTR	
cg27440050	0,053701271	0,154699944	0,100998673	0,003452874	MAPK7	MAPK7;MAPK7;MAPK7;MAPK7	NM_002749;NM_139033;NM_139034;NM_139032	TSS1500;TSS1500;TSS1500	N_Shore
cg08548315	0,905761883	0,795005182	-0,110756701	0,003453852					
cg11553116	0,849892454	0,742417865	-0,107474589	0,003459502					N_Shelf
cg14087715	0,89651647	0,77891001	-0,11760646	0,003459524					N_Shelf
cg14219681	0,818867433	0,682863467	-0,136003966	0,003464287					
cg25223716	0,819515646	0,706169509	-0,113346137	0,003470416	ADAM12	ADAM12;ADAM12	NM_003474;NM_021641	Body;Body	
cg13500489	0,274046686	0,381251695	0,107205008	0,003481333	LCA5L	LCA5L	NM_152505	5UTR	N_Shelf
cg07944396	0,897485979	0,793586095	-0,103899883	0,003483524	A2BP1	A2BP1;A2BP1;A2BP1;A2BP1;A2BP1;A2BP1	NM_001142334;NM_145891;NM_145892;NM_001142333;NM_1	Body;TSS1500;TSS1500;Bod	
cg11122255	0,13178009	0,233567472	0,101787382	0,003484126	ARHGAP21	ARHGAP21	NM_020824	Body	
cg07034145	0,911994648	0,801078815	-0,110915833	0,003489681					
cg08808788	0,730649002	0,55625956	-0,174389443	0,003491842					
cg07336740	0,948249636	0,82532549	-0,122924147	0,00349459	PTPRG	PTPRG	NM_002841	Body	
cg13605781	0,923592408	0,780997534	-0,142594874	0,003499323					
cg25225665	0,920866806	0,797713599	-0,123153207	0,00350664	SVIL	SVIL	NM_003174	5UTR	N_Shore
cg25847633	0,172761395	0,318937305	0,14617591	0,003510547	C6orf126	C6orf126	NM_207409	TSS200	Island
cg20256059	0,918435828	0,782945336	-0,135490492	0,003522826	FRMD6	FRMD6	NM_001042481	5UTR	
cg00098144	0,166115947	0,317876796	0,151760849	0,003524033					Island
cg13231067	0,878753155	0,749287085	-0,12946607	0,003524111	ANKMY1	ANKMY1;ANKMY1	NM_017844;NM_016552	Body;Body	
cg22862635	0,776189787	0,891338135	0,115148348	0,003530492	PTPRN2	PTPRN2;PTPRN2;PTPRN2	NM_002847;NM_130842;NM_130843	Body;Body;Body	
cg10038446	0,928230899	0,808659497	-0,119571402	0,00353256	FLRT3	FLRT3;MACROD2;FLRT3	NM_013281;NM_080676;NM_198391	Body;Body;Body	
cg08544453	0,697998936	0,806196674	0,108197738	0,003536391					
cg11950778	0,653722271	0,764304824	0,110582553	0,003541797	UGT8	UGT8	NM_001128174	TSS1500	N_Shore
cg06385378	0,812390317	0,704295095	-0,108095222	0,003543196					S_Shelf
cg13768242	0,10404376	0,223165204	0,119121444	0,003547597					Island

cg03588007	0,952665487	0,845075979	-0,107589508	0,003547758	TRIM14	TRIM14;TRIM14;TRIM14;TRIM14	NM_033220;NM_033219;NM_014788;NM_033221	3UTR;3UTR;3UTR;3UTR	N_Shore
cg21513537	0,836132598	0,717217775	-0,118914823	0,003550734					
cg15612847	0,808315646	0,642126241	-0,166189406	0,003551019	MITD1	MITD1;MRPL30	NM_138798;NM_145212	Body;TSS1500	N_Shore
cg14962025	0,911940882	0,750798713	-0,161142169	0,003555878					
cg26142661	0,516001213	0,621777954	0,105776741	0,00355736	ZMAT1	ZMAT1	NM_001011657	5UTR	Island
cg17789193	0,142192267	0,270585374	0,128393107	0,003558492					S_Shelf
cg09016610	0,827158166	0,66538961	-0,161768555	0,003568791	SMYD3	SMYD3;SMYD3	NM_022743;NM_001167740	Body;Body	
cg21657334	0,901451373	0,641653349	-0,259798024	0,003571451					
cg17806882	0,913419429	0,771886692	-0,141532737	0,003573699	GIMAP7	GIMAP7	NM_153236	5UTR	
cg04221681	0,900581897	0,69689165	-0,203690247	0,003573974	PHYH	PHYH;PHYH	NM_001037537;NM_006214	5UTR;Body	N_Shelf
cg11063088	0,759428115	0,606185014	-0,153243101	0,003575659	DLG5	DLG5	NM_004747	Body	
cg10171307	0,696300482	0,80138074	0,105080259	0,003579821					S_Shore
cg04265964	0,916003384	0,807862953	-0,108140431	0,00358304					
cg21156970	0,815804505	0,710512146	-0,105292359	0,003586062					
cg09674502	0,898744346	0,791338299	-0,107406047	0,003590728	GFI1	GFI1	NM_005263	TSS1500	S_Shore
cg25493764	0,906577379	0,800081962	-0,106495416	0,003590907					N_Shore
cg26903996	0,888842044	0,785825543	-0,103016501	0,003592525	KLHL3	KLHL3	NM_017415	TSS1500	
cg08002746	0,93493896	0,817010928	-0,117928032	0,003597962					
cg02372283	0,925353021	0,78107502	-0,144278	0,003600865	ZNF264	ZNF264	NM_003417	TSS1500	N_Shore
cg19892287	0,057742552	0,20543666	0,147694108	0,00360405	HOXC4	HOXC4;HOXC5;HOXC6	NM_014620;NR_003084;NM_153693	5UTR;Body;5UTR	N_Shore
cg19136192	0,920760301	0,804468126	-0,116292176	0,003605431	RELL1	RELL1;C4orf19;C4orf19	NM_001085399;NM_018302;NM_001104629	3UTR;3UTR;3UTR	
cg07582862	0,143270311	0,250545201	0,107274889	0,003605695					
cg27221053	0,080995238	0,214737049	0,133741811	0,003609454					
cg13977660	0,626858749	0,508099087	-0,118759663	0,00361116	USP42	USP42	NM_032172	5UTR	S_Shelf
cg11333568	0,850332958	0,711436926	-0,138896032	0,003653251					
cg13730905	0,914439369	0,795290676	-0,119148693	0,003657455	IFIT1L	IFIT1L	NM_001010987	TSS1500	
cg14316231	0,706574387	0,812420734	0,105846347	0,003680353	MYST3	MYST3;MYST3;MYST3	NM_001099413;NM_001099412;NM_006766	Body;Body;Body	
cg25174844	0,696947504	0,813507145	0,116559641	0,003686309					
cg02898665	0,743195499	0,596122694	-0,147072805	0,003690528					N_Shore
cg02375313	0,86065528	0,631587818	-0,229067462	0,003693142	EXOC2	EXOC2;HUS1B	NM_018303;NM_148959	5UTR;TSS1500	S_Shore
cg21288315	0,948460084	0,841798707	-0,106661376	0,003693983	ZNF548	ZNF548	NM_152909	TSS1500	N_Shore
cg18923635	0,907221342	0,782767724	-0,124453618	0,003695628					N_Shore
cg26759036	0,842851909	0,715410894	-0,127441014	0,003696668	SH3BP2	SH3BP2;SH3BP2;SH3BP2;SH3BP2	NM_001122681;NM_001145855;NM_003023;NM_001145856	Body;Body;Body;Body	
cg03000989	0,937726611	0,745203192	-0,192523419	0,003697651	PLEKHA7	PLEKHA7	NM_175058	Body	S_Shelf
cg07368127	0,921147485	0,801769353	-0,119378132	0,00370365	KIAA1267	KIAA1267	NM_015443	Body	
cg08996726	0,919257842	0,672128058	-0,247129784	0,003708908	MAML3	MAML3	NM_018717	Body	
cg24544594	0,883293191	0,731023782	-0,152269409	0,003711475	NCRNA00028	NCRNA00028	NR_024358	Body	S_Shelf
cg09122414	0,937512236	0,82321352	-0,114298716	0,003712375	INPP5B	INPP5B	NM_005540	Body	
cg03915012	0,75916501	0,869570251	0,110405241	0,003714882	GAK	GAK	NM_005255	Body	S_Shelf
cg17100390	0,884324383	0,771906581	-0,112417802	0,003715923					S_Shelf
cg16241309	0,885388924	0,730530161	-0,154858763	0,00372139	C1orf94	C1orf94;C1orf94	NM_001134734;NM_032884	Body;5UTR	
cg01176915	0,938265956	0,834746643	-0,103519312	0,003731842	DSG4	DSG4;DSG4	NM_177986;NM_001134453	TSS1500;TSS1500	
cg06599575	0,854901925	0,75187809	-0,103023835	0,003734712	KIAA1009	KIAA1009	NM_014895	Body	N_Shelf
cg20964082	0,918227617	0,808466755	-0,109760862	0,003740018	ERO1LB	ERO1LB	NM_019891	Body	N_Shelf
cg01555458	0,881576217	0,747742585	-0,133833633	0,003741603					N_Shelf
cg15291905	0,860948297	0,757298852	-0,103649444	0,003744114	INHBA	INHBA;LOC285954;LOC285954	NM_002192;NR_027118;NR_027119	5UTR;Body;Body	N_Shelf
cg25816610	0,908010952	0,798932407	-0,109078546	0,00374649	DSCR8	DSCR8;DSCR8;DSCR8;DSCR8;DSCR8;DSCR4	NR_026838;NR_026840;NR_026841;NR_026842;NR_026839;NM	Body;Body;Body;Body;Body;T	
cg03339057	0,935473872	0,807998411	-0,12747546	0,003749058					
cg25648436	0,352508168	0,25048065	-0,102027518	0,003749147	ZSCAN1	ZSCAN1	NM_182572	Body	Island
cg10623600	0,174859236	0,29044446	0,115585224	0,003749836					Island
cg13117439	0,369389942	0,473877028	0,104487086	0,003752054	psiTPTE22	psiTPTE22	NR_001591	TSS1500	N_Shore
cg12832162	0,963981257	0,85949201	-0,104489248	0,003753228	PTK2B	PTK2B;PTK2B;PTK2B;PTK2B	NM_173175;NM_004103;NM_173174;NM_173176	5UTR;5UTR;5UTR;5UTR	
cg19175386	0,180600508	0,307604487	0,12700398	0,003761747					N_Shore
cg12246945	0,837362547	0,665838035	-0,171524512	0,003763154	HIPK2	HIPK2;HIPK2	NM_022740;NM_001113239	3UTR;3UTR	
cg05678871	0,765982782	0,610057107	-0,155925674	0,003764777					N_Shelf
cg01922700	0,923241552	0,79650844	-0,126733112	0,003773921	CRB1	CRB1	NM_201253	Body	
cg05283871	0,902781322	0,78693553	-0,115845792	0,003775489	PNPLA4	PNPLA4;PNPLA4	NM_001142389;NM_004650	TSS1500;TSS1500	S_Shore
cg20817228	0,900853482	0,77861882	-0,122234662	0,003775616	LOC728855	LOC728855	NR_024510	Body	N_Shore
cg02747612	0,873717635	0,674117818	-0,199599817	0,003777371	ZNF91	ZNF91	NM_003430	TSS1500	
cg17465884	0,891916695	0,729594714	-0,162321981	0,003785071	CDK16	CDK16	NM_033018	TSS1500	N_Shore
cg08319412	0,8255094	0,721182949	-0,104326451	0,003789963					S_Shore
cg18031861	0,774501821	0,653326223	-0,121175598	0,003791833					
cg17860366	0,17691427	0,2774929	0,100578629	0,003796371	ABHD2	ABHD2;ABHD2	NM_152924;NM_007011	5UTR;5UTR	S_Shelf
cg21000919	0,944657922	0,798101566	-0,146556357	0,003798759					N_Shelf
cg13027369	0,828729106	0,686387121	-0,142341985	0,003799245	KPNA4	KPNA4	NM_002268	Body	N_Shelf
cg04150100	0,141303228	0,263804364	0,122501136	0,003799887	SIK2	SIK2	NM_015191	Body	
cg13227162	0,811403612	0,704019989	-0,107383623	0,003810267	RTL1	RTL1	NM_001134888	TSS200	S_Shore
cg20431441	0,816835152	0,710138611	-0,106696541	0,00381114					N_Shelf
cg07236562	0,92805743	0,825188621	-0,102868809	0,003813532					
cg09628499	0,802128685	0,680688699	-0,121439986	0,003816324					S_Shelf
cg02533281	0,935284922	0,727945564	-0,207339358	0,003816698	MED13L	MED13L	NM_015335	3UTR	
cg02399019	0,846610379	0,727223918	-0,119386461	0,003820117	CTBP2	CTBP2;CTBP2	NM_001083914;NM_001329	5UTR;5UTR	
cg21151541	0,900002531	0,797095793	-0,102906738	0,003824526	RBM47	RBM47	NM_001098634	TSS1500	S_Shore
cg17311921	0,913562903	0,804623768	-0,108939135	0,003828491	UIMC1	UIMC1	NM_016290	5UTR	N_Shelf
cg15110917	0,91979892	0,819068861	-0,100730059	0,003832418					
cg09636727	0,91910312	0,783850844	-0,135252276	0,003833982	ST6GALNAC5	ST6GALNAC5	NM_030965	Body	
cg00569673	0,200356898	0,35472519	0,154368292	0,003840846	GPSM3	GPSM3	NM_022107	5UTR	N_Shelf
cg13650965	0,867707893	0,697570255	-0,170137638	0,003854327	TSPO2	TSPO2;TSPO2	NM_001159726;NM_001010873	3UTR;3UTR	
cg19472302	0,820105432	0,688506319	-0,131599113	0,003856983	ACAP2	ACAP2	NM_012287	Body	
cg03022921	0,102565589	0,209904528	0,107338939	0,003861685					Island
cg17492395	0,131923801	0,243459561	0,11153576	0,003862052					
cg02614999	0,923322246	0,760791016	-0,16253123	0,003865523	POLR1B	POLR1B;POLR1B	NM_019014;NM_001137604	3UTR;3UTR	
cg16001323	0,614546471	0,730494293	0,115947821	0,003873413	ADH1B	ADH1B	NM_000668	3UTR	
cg22675645	0,117389516	0,227167816	0,1097783	0,003875835	KCNIP2	KCNIP2;KCNIP2;KCNIP2;KCNIP2;KCNIP2;KCNIP2;KCNIP2	NM_173195;NM_173191;NM_173193;NM_173194;NM_173192;N	Body;Body;Body;Body;Body;B	Island
cg20559537	0,597584133	0,69813602	0,100551887	0,003880733	N4BP3	N4BP3	NM_015111	TSS1500	N_Shore
cg06033194	0,900768984	0,774903122	-0,125865862	0,003886272	DPY19L1	DPY19L1	NM_015283	TSS1500	S_Shore
cg05320533	0,725869544	0,607754169	-0,118115375	0,003888715	ANKRD11	ANKRD11	NM_013275	5UTR	
cg26679158	0,930486907	0,821298027	-0,109188879	0,00389005	C6orf186	C6orf186	NM_001123364	Body	N_Shelf
cg24786705	0,908087141	0,799182646	-0,108904495	0,003890977					
cg19915181	0,929827112	0,811259613	-0,118567499	0,003891081					S_Shelf
cg23864202	0,123351488	0,248645859	0,125294371	0,003895308	BDKRB1	BDKRB1	NM_000710	TSS200	
cg07971797	0,899707851	0,77320836	-0,126499491	0,003895798	PIWIL3	PIWIL3;PIWIL3	NM_001008496;NM_001008496	1stExon;5UTR	
cg08222514	0,143725389	0,246714434	0,102989046	0,003903005	SENP6	SENP6;SENP6	NM_015571;NM_001100409	Body;Body	Island
cg22304239	0,289462244	0,398409946	0,108947702	0,003903461	DAPK3	DAPK3	NM_001348	Body	N_Shelf
cg08818829	0,598031531	0,714315159	0,116283627	0,003910446	DNAH5	DNAH5	NM_001369	Body	Island
cg18409985	0,717171966	0,823512695	0,106340728	0,003922502	ATP11A	ATP11A;ATP11A	NM_015205;NM_032189	Body;Body	
cg19464087	0,530420936	0,666936004	0,136515069	0,003924099	SETD5	SETD5	NM_001080517	5UTR	S_Shelf
cg12251967	0,876395785	0,729633712	-0,146762073	0,003932851	PTPRN2	PTPRN2;PTPRN2;PTPRN2	NM_002847;NM_130842;NM_130843	Body;Body;Body	
cg26700919	0,208858419	0,336588266	0,127729847	0,003933831	C18orf1	C18orf1;C18orf1	NM_181482;NM_181481	5UTR;5UTR	
cg15044181	0,767676274	0,615724466	-0,151951808	0,003936887	IGF2R	IGF2R	NM_000876	Body	
cg23896898	0,85505533	0,687561824	-0,167493506	0,003940167					
cg06557683	0,935170112	0,832418287	-0,102751826	0,003941147	ZNF135	ZNF135;ZNF135;ZNF135;ZNF135	NM_001164530;NM_003436;NM_001164529;NM_007134	3UTR;3UTR;3UTR;3UTR	
cg09948769	0,876703176	0,682778847	-0,193924329	0,003944788					N_Shelf
cg19623588	0,980843126	0,842395575	-0,138447552	0,003948278	REL	REL	NM_002908	3UTR	
cg07513723	0,92282127	0,798768978	-0,124052292	0,003954044	OR6X1	OR6X1	NM_001005188	TSS200	
cg12822286	0,239671146	0,368812864	0,129141717	0,003959101	LOC100009676	LOC100009676;ZBTB11	NR_024407;NM_014415	TSS1500;Body	N_Shore
cg12654155	0,898403831	0,728921179	-0,169482651	0,003960977					
cg22203628	0,837658361	0,737495492	-0,10016287	0,003969033					
cg12814766	0,85878557	0,74448805	-0,11429752	0,003980423	MAP3K9	MAP3K9	NM_033141	Body	
cg03865648	0,283677765	0,491113956	0,207436191	0,003982928					N_Shore
cg12671750	0,955491977	0,850534754	-0,104957223	0,003984449	MYO5B	MYO5B	NM_001080467	3UTR	
cg05524038	0,915579008	0,774955755	-0,140623253	0,003988474	CSF1R	CSF1R	NM_005211	TSS1500	
cg09897656	0,93022755	0,776853401	-0,153374149	0,003988991	SLC26A7	SLC26A7;SLC26A7	NM_052832;NM_134266	Body;Body	
cg21780231	0,08959897	0,196823191	0,107224221	0,003999297	FAM173B	FAM173B;CCT5	NM_199133;NM_012073	TSS1500;Body	S_Shore
cg14600345	0,907457314	0,800007913	-0,107449401	0,004006244					N_Shelf
cg13689591	0,897289012	0,769603093	-0,12768592	0,004008582	KALRN	KALRN	NM_001024660	Body	
cg01874112	0,732552765	0,834885854	0,102333089	0,004020904					
cg12973904	0,63791345	0,741807623	0,103894173	0,004022222					N_Shelf
cg25897891	0,927804063	0,768356374	-0,159447689	0,004024768					N_Shelf
cg22026457	0,122807194	0,253172095	0,130364901	0,00403537					Island
cg14837480	0,823447709	0,66809882	-0,155348889	0,004039127	TRIM9	TRIM9;TRIM9	NM_052978;NM_015163	3UTR;Body	
cg13947735	0,742694065	0,844777497	0,102083432	0,004039869	B3GNTL1	B3GNTL1	NM_001009905	Body	S_Shelf
cg19742492	0,884504529	0,76121095	-0,123293579	0,004040244	ANO1	ANO1;ANO1	NM_018043;NR_030691	Body;Body	S_Shelf
cg14355428	0,095381814	0,197102055	0,101720242	0,004044877	CYP51A1	CYP51A1;CYP51A1	NM_001146152;NM_000786	5UTR;Body	N_Shore
cg15010283	0,908414671	0,73335873	-0,175055941	0,004044941	RAB33B	RAB33B	NM_031296	Body	
cg15664504	0,094518047	0,209434856	0,114916809	0,00405209	LRP1B	LRP1B	NM_018557	Body	
cg04098169	0,888276797	0,769372808	-0,118903989	0,004058521					
cg10269439	0,941877168	0,827028398	-0,11484877	0,004064983	IL1F7	IL1F7;IL1F7;IL1F7;IL1F7;IL1F7;IL1F7;IL1F7;IL1F7	NM_173204;NM_173202;NM_173203;NM_173202;NM_173203;N	5UTR;1stExon;5UTR;5UTR;1s	
cg18150460	0,825636463	0,665899058	-0,159737405	0,004071918	PRDM2	PRDM2;PRDM2;PRDM2;PRDM2	NM_001007257;NM_012231;NM_015866;NM_001135610	Body;Body;Body;Body	
cg22949073	0,920677458	0,793574927	-0,12710253	0,004075177	TP53	TP53;TP53;TP53;TP53;TP53;TP53;TP53	NM_001126114;NM_000546;NM_001126113;NM_001126115;N	3UTR;3UTR;3UTR;3UTR;3UTR	
cg17513789	0,85781064	0,724138723	-0,133671917	0,004086252	XIST	XIST	NR_001564	TSS1500	
cg19419054	0,818025978	0,691269105	-0,126756872	0,004088366	DAAM2	DAAM2	NM_015345	5UTR	
cg08638929	0,942567591	0,833433363	-0,109134228	0,004089742	TSPAN1	TSPAN1	NM_005727	TSS1500	
cg08813545	0,916365423	0,786487789	-0,129877634	0,004091184	CCL20	CCL20;CCL20	NM_001130046;NM_004591	Body;Body	
cg01253545	0,033939891	0,168431746	0,134491856	0,004092628	RNF185	RNF185;RNF185;RNF185;RNF185;RNF185;RNF185;RNF185	NM_001135824;NM_152267;NR_024211;NR_024209;NR_02421	5UTR;5UTR;Body;Body;Body;	Island
cg01080552	0,737605774	0,844740015	0,107134241	0,004099025	ARSF	ARSF	NM_004042	5UTR	
cg24845329	0,708896343	0,820493335	0,111596993	0,004100533	THEG	THEG;THEG	NM_016585;NM_199202	TSS1500;TSS1500	S_Shore
cg12794017	0,926255062	0,820829994	-0,105425068	0,004101663	SPTBN1	SPTBN1	NM_003128	5UTR	
cg19769445	0,853907048	0,753086727	-0,100820321	0,004114038					S_Shelf
cg08780063	0,900716118	0,783824737	-0,116891381	0,004115479	TRIM37	TRIM37;TRIM37	NM_015294;NM_001005207	3UTR;Body	
cg17400829	0,913920463	0,795920425	-0,118000038	0,004127393					S_Shelf
cg05564412	0,544658542	0,656956203	0,112297661	0,004127664	CNGA3	CNGA3;CNGA3	NM_001079878;NM_001298	TSS1500;TSS1500	N_Shore
cg14373760	0,939392018	0,803653342	-0,135738676	0,004139608	GPR183	GPR183;UBAC2;UBAC2;UBAC2	NM_004951;NM_001144072;NM_177967;NR_026644	TSS1500;Body;Body;Body	
cg14550289	0,890569851	0,771477799	-0,119092051	0,004143265	TIA1	TIA1;TIA1	NM_022173;NM_022037	Body;Body	N_Shelf
cg16034411	0,930966245	0,779460547	-0,151505698	0,004162062					
cg03195377	0,847144304	0,703952573	-0,143191731	0,004165103					
cg23105474	0,965911491	0,850201683	-0,115709808	0,004168303					S_Shore
cg20281583	0,822808912	0,579423605	-0,243385307	0,004169033	NEIL3	NEIL3	NM_018248	Body	S_Shelf
cg11143438	0,733682049	0,84455648	0,11087443	0,004180243					N_Shelf
cg14195249	0,680446781	0,781792886	0,101346105	0,004184465					S_Shore
cg23942717	0,842739134	0,71634113	-0,126398004	0,004195234					S_Shore
cg00729885	0,876947977	0,736246692	-0,140701286	0,004209435	NBEA	NBEA	NM_015678	Body	Island
cg13599248	0,665714553	0,765980645	0,100266092	0,004209955	SLC17A5	SLC17A5	NM_012434	TSS1500	S_Shore
cg08335129	0,926883838	0,820048057	-0,106835781	0,004212566	TRERF1	TRERF1	NM_033502	5UTR	
cg15463964	0,921109393	0,809026571	-0,112082822	0,004214513					N_Shelf
cg15166523	0,475024086	0,615807303	0,140783217	0,004220579	VSIG4	VSIG4;VSIG4	NM_001100431;NM_007268	TSS1500;TSS1500	
cg06231362	0,913739353	0,784017023	-0,12972233	0,004236039					N_Shelf
cg19086858	0,242019932	0,377954414	0,135934482	0,004250686	DENND5B	DENND5B	NM_144973	Body	Island
cg04414518	0,153856291	0,280395383	0,126539092	0,004252504	POLR2K	POLR2K	NM_005034	TSS1500	S_Shelf
cg04359351	0,963195905	0,860644982	-0,102550923	0,00425794	TTC15	TTC15	NM_016030	Body	
cg00338529	0,899436846	0,772308308	-0,127128538	0,004263545					N_Shore
cg05412784	0,929700394	0,809440694	-0,1202597	0,004266463	CPA6	CPA6;CPA6	NM_020361;NM_001127445	Body;Body	
cg08699575	0,839405374	0,673314315	-0,166091059	0,004269055					
cg24192909	0,652372058	0,756159816	0,103787758	0,004271042					
cg27183454	0,833852983	0,711210809	-0,122642175	0,004276953	SCAND3	SCAND3	NM_052923	Body	
cg07032258	0,119181747	0,290571926	0,171390179	0,004289529	ARNTL	ARNTL;ARNTL;ARNTL	NM_001030272;NM_001178;NM_001030273	5UTR;5UTR;5UTR	
cg13623290	0,910317051	0,794108299	-0,116208752	0,004300984	CDYL	CDYL;CDYL	NM_004824;NR_026590	Body;Body	S_Shelf
cg19386714	0,935878585	0,792867091	-0,143011493	0,004302401	C18orf55	C18orf55	NM_014177	3UTR	
cg04470054	0,078696899	0,224622796	0,145925898	0,004304878	RPTOR	RPTOR;RPTOR	NM_001163034;NM_020761	Body;Body	N_Shore
cg24768595	0,087218166	0,190660619	0,103442453	0,004311953	CYTH1	CYTH1;CYTH1	NM_017456;NM_004762	Body;Body	
cg00062736	0,093471657	0,19774493	0,104273273	0,004315521	MEF2A	MEF2A;MEF2A;MEF2A;MEF2A	NM_001130927;NM_001130926;NM_001130928;NM_005587	5UTR;5UTR;TSS1500;5UTR	
cg08790301	0,947577947	0,8458535	-0,101724447	0,004318508	RBPMS	RBPMS;RBPMS;RBPMS;RBPMS	NM_001008712;NM_001008710;NM_001008711;NM_006867	Body;Body;Body;Body	
cg08464734	0,928472165	0,790095355	-0,138376811	0,00432349	RASA3	RASA3	NM_007368	Body	Island
cg11234447	0,86867117	0,723127395	-0,145543776	0,004324919					
cg14171944	0,709132319	0,821550023	0,112417704	0,004331524	DENND1A	DENND1A;DENND1A	NM_024820;NM_020946	Body;Body	
cg21107200	0,901191167	0,770447287	-0,13074388	0,004340862	PPP2R5E	PPP2R5E	NM_006246	Body	
cg04843137	0,90441177	0,783654193	-0,120757577	0,004359389	NUDT7	NUDT7;NUDT7	NM_001105663;NR_024154	Body;Body	S_Shelf
cg02307542	0,828318012	0,679672352	-0,14864566	0,004365692					
cg01773144	0,740443713	0,598610887	-0,141832826	0,004376089					N_Shore
cg00890649	0,879185198	0,770875084	-0,108310114	0,004378417	KLHDC3	KLHDC3	NM_057161	Body	N_Shore
cg07259905	0,877081367	0,76955726	-0,107524107	0,004383785					

cg18119394	0,821790664	0,71705499	-0,104735674	0,004387782	IL1RAPL2	IL1RAPL2;TEX13A	NM_017416;NM_031274	Body;5UTR	
cg02175309	0,944169551	0,789313075	-0,154856476	0,004392098	NUBPL	NUBPL	NM_025152	Body	
cg07283217	0,776509521	0,637597351	-0,13891217	0,004396353					
cg20022454	0,947239855	0,838905741	-0,108334114	0,004407	TMCO3	TMCO3	NM_017905	Body	N_Shore
cg00821400	0,917332555	0,800552473	-0,116780081	0,00441164	TBPL2	TBPL2	NM_199047	Body	
cg18198306	0,088140346	0,190989347	0,102849001	0,004417118	FBXO5	FBXO5;FBXO5	NM_001142522;NM_012177	5UTR;Body	N_Shore
cg19198148	0,853833301	0,693443745	-0,160389556	0,004423101	BCL6	BCL6;BCL6;BCL6	NM_001706;NM_001130845;NM_001134738	5UTR;5UTR;5UTR	N_Shelf
cg13628937	0,945130203	0,785958545	-0,159171658	0,00443496					
cg09136597	0,631625407	0,737646298	0,106020891	0,004435773	NPHP4	NPHP4	NM_015102	Body	
cg20945531	0,503129615	0,612768103	0,109638487	0,004437448	FLJ34503	FLJ34503	NR_027060	TSS1500	
cg07479270	0,943966731	0,828081412	-0,115885319	0,004438437					
cg19905504	0,884025137	0,773491317	-0,110533819	0,00444868					
cg24666876	0,41199332	0,520192335	0,108199014	0,004449718	SRPX2	SRPX2;SRPX2	NM_014467;NM_014467	1stExon;5UTR	
cg10839997	0,098705248	0,204486126	0,105780878	0,004452445	B3GNT1	B3GNT1	NM_006876	TSS1500	S_Shore
cg18917023	0,171949558	0,296735156	0,124785598	0,004468691	KCNMA1	KCNMA1;KCNMA1;KCNMA1;KCNMA1	NM_001161353;NM_001161352;NM_002247;NM_001014797	Body;Body;Body;Body	
cg13683424	0,192703383	0,329398832	0,136695449	0,004471949					Island
cg21970438	0,773778793	0,875352964	0,101574171	0,004479708	TTLL2	TTLL2	NM_031949	TSS1500	
cg09247657	0,926520529	0,761083235	-0,165437295	0,004479916	EML1	EML1;EML1	NM_001008707;NM_004434	Body;Body	S_Shelf
cg17217741	0,86544369	0,762636524	-0,102807166	0,004486003					N_Shelf
cg11807290	0,870770648	0,705345391	-0,165425257	0,004489227	ZNF605	ZNF605;ZNF605	NM_183238;NM_001164715	Body;Body	
cg07244927	0,711917851	0,817916612	0,105998761	0,004495446	CDH8	CDH8	NM_001796	5UTR	N_Shore
cg00598866	0,941436121	0,78967175	-0,151764371	0,004496973	INPP4B	INPP4B;INPP4B	NM_003866;NM_001101669	Body;Body	
cg20942931	0,511202599	0,61578436	0,10458176	0,004501098					
cg24661860	0,674646912	0,798787029	0,124140117	0,004502784	CASZ1	CASZ1;CASZ1	NM_001079843;NM_017766	5UTR;5UTR	
cg26373922	0,898245127	0,79211247	-0,106132657	0,004506462	NUP210L	NUP210L;NUP210L	NM_001159484;NM_207308	Body;Body	
cg12136632	0,722143298	0,827690681	0,105547383	0,004509566					Island
cg14373611	0,837564046	0,712866264	-0,124697783	0,004512111	GJB6	GJB6;GJB6;GJB6;GJB6	NM_001110220;NM_001110219;NM_001110221;NM_006783	5UTR;5UTR;5UTR;5UTR	
cg19462116	0,875133217	0,730594629	-0,144538588	0,004517288	DIP2C	DIP2C	NM_014974	Body	
cg26390924	0,814601244	0,712962787	-0,101638457	0,004518063	GABPB1	GABPB1;GABPB1;GABPB1;GABPB1;GABPB1	NM_016655;NM_005254;NM_016654;NM_002041;NM_181427	5UTR;5UTR;5UTR;5UTR;5UTR	
cg24451888	0,929857529	0,818168851	-0,111688678	0,004522192	AFF1	AFF1;AFF1	NM_001166693;NM_005935	Body;Body	
cg10669424	0,947788742	0,841796009	-0,105992733	0,004528475	VGLL4	VGLL4	NM_014667	TSS1500	S_Shore
cg04173714	0,07023318	0,181504342	0,111271162	0,004534719	AKAP13	AKAP13;AKAP13	NM_007200;NM_006738	Body;Body	
cg07387734	0,947312543	0,831215874	-0,116096669	0,004540232	NFYC	NFYC;NFYC;NFYC;NFYC;MIR30E;NFYC	NM_001142589;NM_014223;NM_001142587;NM_001142588;N	Body;Body;Body;Body;TSS15	
cg16859420	0,235182424	0,370216719	0,135034295	0,004550518					
cg25728188	0,137754682	0,248505858	0,110751176	0,004562752					
cg14152778	0,899134563	0,784656794	-0,114477769	0,00456323	RMND5A	RMND5A	NM_022780	3UTR	
cg10277836	0,865971285	0,760516576	-0,105454709	0,004564669	SLC30A6	SLC30A6	NM_017964	3UTR	
cg09638516	0,904584016	0,720998098	-0,183585918	0,004567551	ADI1	ADI1	NM_018269	3UTR	N_Shelf
cg21848671	0,121571036	0,22601937	0,104448334	0,004573023	NAV2	NAV2	NM_001111018	Body	
cg19647111	0,883058262	0,713067108	-0,169991154	0,00457606	TNXB	TNXB	NM_019105	5UTR	
cg00997853	0,041552202	0,160487512	0,11893531	0,004583533	ITPR2	ITPR2	NM_002223	Body	
cg20332865	0,950828868	0,81617712	-0,134651748	0,004583824	PARD3B	PARD3B;PARD3B;PARD3B	NM_152526;NM_057177;NM_205863	Body;Body;Body	
cg06746362	0,876568859	0,656552027	-0,220016832	0,004586572	PTPN21	PTPN21	NM_007039	Body	S_Shore
cg00832547	0,83750074	0,711141885	-0,126358854	0,00459028	RAGE	RAGE	NM_014226	Body	
cg25547520	0,13625311	0,241425083	0,105171973	0,004594093	SMAD3	SMAD3;SMAD3;SMAD3	NM_005902;NM_001145103;NM_001145102	Body;Body;5UTR	
cg07938847	0,637279562	0,513096472	-0,124183091	0,004594317	PRKG2	PRKG2	NM_006259	TSS200	
cg14861497	0,925659454	0,806599595	-0,119059859	0,004595192	KLF5	KLF5	NM_001730	Body	
cg26207102	0,963068267	0,849231237	-0,11383703	0,00460042	TEAD1	TEAD1	NM_021961	5UTR	
cg11835978	0,918459626	0,780411894	-0,138047731	0,004607349	SEMA5A	SEMA5A	NM_003966	3UTR	
cg20972294	0,639714215	0,740193424	0,100479209	0,004608089	OPCML	OPCML	NM_001012393	Body	
cg00671224	0,953353412	0,844964681	-0,108388731	0,00460835					S_Shelf
cg06379478	0,833444058	0,715807928	-0,11763613	0,004612099	LMF1	LMF1	NM_022773	Body	Island
cg24068761	0,952463732	0,847038634	-0,105425098	0,004615215	KCNAB2	KCNAB2;KCNAB2	NM_003636;NM_172130	Body;Body	
cg02942186	0,906267772	0,751142698	-0,155125073	0,004620547	C1orf100	C1orf100	NM_001012970	TSS1500	
cg19763774	0,90400848	0,741831976	-0,162176503	0,004622022	TSPAN2	TSPAN2	NM_005725	Body	
cg23011899	0,842362886	0,711431147	-0,130931738	0,004622894					
cg22473961	0,918923842	0,792957264	-0,125966578	0,004630785	CD63	CD63;CD63	NM_001040034;NM_001780	3UTR;3UTR	N_Shelf
cg27484483	0,882168889	0,732828245	-0,149340645	0,00463136	RRM2B	RRM2B	NM_015713	Body	N_Shore
cg13225596	0,095746553	0,19694985	0,101203297	0,004640244	BAI3	BAI3	NM_001704	TSS1500	N_Shore
cg07170824	0,291884358	0,461286835	0,169402476	0,00464065	ACVRL1	ACVRL1;ACVRL1	NM_000020;NM_000020	1stExon;5UTR	Island
cg00155622	0,127707983	0,242448374	0,114740391	0,004646334	RTF1	RTF1	NM_015138	Body	S_Shore
cg08998414	0,885141815	0,639184714	-0,245957101	0,004648406	UBE2H	UBE2H;UBE2H	NM_003344;NM_182697	Body;Body	
cg10620881	0,90483277	0,796575937	-0,108256833	0,004648488	PTPRN2	PTPRN2;PTPRN2;PTPRN2	NM_002847;NM_130842;NM_130843	Body;Body;Body	N_Shore
cg17717588	0,942900125	0,841532673	-0,101367452	0,00465584	GTDC1	GTDC1;GTDC1	NM_001006636;NM_001164629	TSS1500;5UTR	
cg21887097	0,11265249	0,217885834	0,105233344	0,004656402	LAMB1	LAMB1	NM_002291	Body	
cg24585292	0,911561488	0,783136591	-0,128424897	0,004659232					
cg22822822	0,118732301	0,248878525	0,130146223	0,004664049					N_Shelf
cg07533819	0,922340001	0,799710265	-0,122629736	0,004665003	MAP1B	MAP1B	NM_005909	Body	S_Shelf
cg23748584	0,909462826	0,758526801	-0,150936026	0,004678274					
cg11570233	0,882543981	0,773317186	-0,109226795	0,004680408	IRF6	IRF6	NM_006147	5UTR	N_Shore
cg20795500	0,899441913	0,738764662	-0,160677251	0,004683979	OLA1	OLA1;OLA1	NM_013341;NM_001011708	Body;Body	
cg11791507	0,879326842	0,773033122	-0,10629372	0,004691928	VN1R4	VN1R4	NM_173857	TSS1500	
cg21831183	0,819841182	0,679682954	-0,140158228	0,004702356					
cg02229100	0,945953034	0,840383573	-0,105569462	0,004704515	ZFYVE1	ZFYVE1;ZFYVE1	NM_021260;NM_178441	Body;5UTR	
cg05335876	0,81659772	0,676255989	-0,14034173	0,004714874	TNFSF18	TNFSF18	NM_005092	Body	
cg20502854	0,949116587	0,837499689	-0,111616898	0,004716877	NCKAP5	NCKAP5;NCKAP5	NM_207363;NM_207481	Body;Body	
cg27511525	0,862069622	0,725490431	-0,136579191	0,004717275	ADAMTS17	ADAMTS17	NM_139057	Body	
cg25924196	0,871544664	0,764882993	-0,106661671	0,004732638					
cg01947066	0,074461317	0,217794892	0,143333575	0,004738287	PML	PML;PML;PML;PML;PML;PML;PML;PML;PML	NM_033247;NM_033244;NM_002675;NM_033238;NM_033246;N	Body;Body;Body;Body;Body;B	N_Shore
cg03397616	0,852557126	0,745510676	-0,10704645	0,00473844	FAM55B	FAM55B	NM_182495	Body	
cg18100746	0,895062915	0,667459629	-0,227603286	0,004744258					
cg05390694	0,123276165	0,239286945	0,11601078	0,004748787	KIAA1024	KIAA1024	NM_015206	TSS1500	N_Shore
cg16095660	0,883398175	0,752184252	-0,131213923	0,004749807	PGAP2	PGAP2;PGAP2;PGAP2;PGAP2;PGAP2	NM_001145438;NM_001145439;NR_027015;NR_027017;NR_02	Body;Body;Body;Body;Body	S_Shelf
cg07572909	0,209382186	0,322981032	0,113598846	0,004762301	SLC16A1	SLC16A1;SLC16A1	NM_001166496;NM_003051	Body;Body	
cg07989042	0,938153105	0,82857143	-0,109581675	0,004766313	CALU	CALU;CALU	NM_001130674;NM_001219	3UTR;3UTR	
cg22260627	0,909194565	0,796053384	-0,113141181	0,004779505					S_Shelf
cg17130745	0,903619346	0,785641936	-0,11797741	0,004782813					
cg04351807	0,934161029	0,818603539	-0,115557489	0,004784183	LDLRAD3	LDLRAD3	NM_174902	Body	
cg20359784	0,226414374	0,125112214	-0,10130216	0,004788245	FAM127B	FAM127B;FAM127B	NM_001078172;NM_001134321	TSS200;TSS200	Island
cg09074544	0,239159523	0,366685158	0,127525636	0,004788285	RAB33A	RAB33A	NM_004794	Body	S_Shore
cg05668429	0,084591205	0,234487322	0,149896117	0,004789945	TWF1	TWF1	NM_002822	TSS1500	S_Shore
cg12486493	0,883773769	0,769764137	-0,114009631	0,0047946	IGF1R	IGF1R	NM_000875	Body	
cg01108633	0,733859285	0,631547467	-0,102311817	0,004804549					
cg24684576	0,898062811	0,785240903	-0,112821907	0,004805361	GJC1	GJC1;GJC1	NM_001080383;NM_005497	5UTR;5UTR	
cg05938624	0,588570172	0,706298535	0,117728363	0,004807404					S_Shelf
cg16683080	0,947715102	0,817155748	-0,130559355	0,004808147	RB1CC1	RB1CC1;RB1CC1	NM_001083617;NM_014781	3UTR;3UTR	
cg00747618	0,853510392	0,751048609	-0,102461783	0,004815756					N_Shore
cg20272423	0,109556539	0,229124734	0,119568195	0,004822051	MRFAP1	MRFAP1	NM_033296	3UTR	S_Shore
cg08212230	0,059749428	0,165085503	0,105336075	0,004825729	CDC20B	CDC20B;CDC20B;CDC20B;GPX8	NM_001145734;NM_152623;NM_001170402;NM_001008397	Body;Body;Body;TSS1500	
cg04654677	0,059357721	0,191889226	0,132531505	0,00482593	RNF43	RNF43	NM_017763	TSS1500	
cg00241002	0,714984791	0,54535535	-0,169629442	0,004829413	LOC731789	LOC731789	NR_026794	TSS200	
cg08897150	0,157082279	0,271028858	0,113946579	0,004835146	CCDC81	CCDC81;CCDC81	NM_021827;NM_001156474	TSS1500;TSS1500	N_Shore
cg19030737	0,881102041	0,778446125	-0,102655917	0,004840635	ITPKB	ITPKB	NM_002221	TSS1500	S_Shore
cg24843535	0,747902311	0,563603323	-0,184298989	0,004842233					S_Shelf
cg16051361	0,111992092	0,22279665	0,110804558	0,004843089	RAB21	RAB21	NM_014999	Body	S_Shore
cg02369820	0,102665332	0,2177528	0,115087468	0,004845902	JAKMIP1	JAKMIP1;JAKMIP1	NM_001099433;NM_144720	5UTR;5UTR	N_Shore
cg07796563	0,84112061	0,661110052	-0,180010558	0,004847844	CHFR	CHFR;CHFR;CHFR;CHFR;CHFR	NM_001161345;NM_001161347;NM_018223;NM_001161346;N	Body;Body;Body;Body;Body	S_Shore
cg22925694	0,958249347	0,840489599	-0,117759748	0,004856061	RAB1A	RAB1A;RAB1A	NM_004161;NM_015543	Body;Body	
cg04918002	0,900032252	0,722735406	-0,177296846	0,004866939	FBXO31	FBXO31	NR_024568	Body	N_Shelf
cg11946963	0,779515139	0,664288861	-0,115226278	0,004885098	NCAM1	NCAM1;NCAM1	NM_000615;NM_181351	Body;Body	
cg04462334	0,917229082	0,77662564	-0,140603443	0,00489504	UQCRB	UQCRB	NM_006294	TSS1500	
cg23586172	0,811197075	0,702148298	-0,109048778	0,004900492	MSI2	MSI2;MSI2	NM_138962;NM_170721	Body;Body	
cg06063519	0,844961149	0,709891459	-0,13506969	0,004903165	IGF1R	IGF1R	NM_000875	Body	
cg13820039	0,079536712	0,219964051	0,140427339	0,004905206	C9orf163	C9orf163;SEC16A	NM_152571;NM_014866	TSS1500;5UTR	N_Shore
cg12033622	0,52200386	0,40138759	-0,120616269	0,004908971	COX4I2	COX4I2	NM_032609	TSS200	Island
cg13618604	0,276932689	0,386431506	0,109498817	0,004920644					Island
cg17437930	0,90095906	0,787671073	-0,113287987	0,00492134	MPP6	MPP6	NM_016447	5UTR	
cg00600521	0,96450926	0,863507582	-0,101001678	0,004926303	AASS	AASS	NM_005763	TSS1500	S_Shore
cg03988518	0,805863834	0,917022236	0,111158403	0,004936714					N_Shore
cg09803164	0,94416815	0,808318689	-0,135849462	0,004937451	C7orf63	C7orf63;C7orf63	NM_001039706;NM_001160138	Body;Body	S_Shelf
cg23225911	0,87532545	0,7638832	-0,11144225	0,004938374	ARNTL2	ARNTL2	NM_020183	Body	
cg16255804	0,94258818	0,769323887	-0,173264293	0,004943605	HBS1L	HBS1L;HBS1L	NM_001145158;NM_006620	Body;Body	
cg16352276	0,110466369	0,212251801	0,101785432	0,004943733	RRAS2	RRAS2;RRAS2	NM_012250;NM_001102669	Body;5UTR	N_Shore
cg24682482	0,841535842	0,642387442	-0,1991484	0,004945018					N_Shelf
cg09024920	0,901594633	0,764420821	-0,137173812	0,004956443	SERPINI2	SERPINI2	NM_006217	Body	
cg06719445	0,956252177	0,850078474	-0,106173703	0,00495709	FOXN3	FOXN3	NM_001085471	5UTR	
cg04849320	0,219173645	0,328904394	0,109730749	0,004957508					N_Shore
cg07284261	0,919559404	0,800585747	-0,118973657	0,004960403	CSF1R	CSF1R	NM_005211	TSS200	
cg26419584	0,592356601	0,692660484	0,100303884	0,00496702					S_Shore
cg08414965	0,836287735	0,682922092	-0,153365642	0,004980677	NPFFR2	NPFFR2	NM_004885	Body	S_Shelf
cg06783548	0,274716315	0,169355484	-0,105360831	0,004981749	SYN1	SYN1;SYN1	NM_006950;NM_133499	TSS200;TSS200	Island
cg24482389	0,942208828	0,83834534	-0,103863488	0,004984324	ZNF331	ZNF331	NM_018555	5UTR	S_Shore
cg04344997	0,31506331	0,213845527	-0,101217783	0,004991812	EMD	EMD	NM_000117	TSS1500	Island
cg16528345	0,908458428	0,796210775	-0,112247654	0,004993898					
cg01973920	0,432636353	0,535261878	0,102625525	0,005002193					
cg07906745	0,885047401	0,762639264	-0,122408136	0,005003338	TMEM167B	TMEM167B	NM_020141	3UTR	N_Shelf
cg04322504	0,956681099	0,844753463	-0,111927637	0,005016181	TM9SF3	TM9SF3	NM_020123	Body	
cg06139431	0,729614234	0,629246136	-0,100368097	0,005019881	CACNA1H	CACNA1H;CACNA1H	NM_021098;NM_001005407	Body;Body	N_Shore
cg10052368	0,939680751	0,813432001	-0,12624875	0,005030512					N_Shelf
cg02722633	0,943602553	0,815935672	-0,12766688	0,005040927	ARHGAP26	ARHGAP26;ARHGAP26	NM_015071;NM_001135608	Body;Body	
cg23660123	0,939723344	0,832083542	-0,107639802	0,005050957					N_Shelf
cg20666533	0,88661669	0,767645355	-0,118971335	0,005053154	PCDH9	PCDH9;PCDH9	NM_203487;NM_020403	Body;Body	
cg21425099	0,094825533	0,19947501	0,104649477	0,005057068	CNTN4	CNTN4	NM_175607	TSS1500	Island
cg15287541	0,895555951	0,769977091	-0,12557886	0,005058234	NCRNA00171	NCRNA00171	NR_026751	Body	
cg05829696	0,887777839	0,770396936	-0,117380903	0,005069073					
cg00251875	0,900962906	0,783127751	-0,117835155	0,005069864	AFAP1	AFAP1;AFAP1	NM_198595;NM_001134647	Body;Body	N_Shore
cg25970363	0,940159736	0,834538511	-0,105621225	0,005071457	PDZK1IP1	PDZK1IP1	NM_005764	Body	
cg01188509	0,827007147	0,697219998	-0,129787149	0,005073657	IGF2R	IGF2R	NM_000876	Body	N_Shelf
cg08278290	0,928960848	0,807984438	-0,12097641	0,005075676	BANP	BANP;BANP	NM_017869;NM_079837	Body;Body	N_Shelf
cg08125397	0,82302358	0,665309331	-0,157714249	0,005079501					N_Shore
cg24426822	0,088183106	0,219021854	0,130838748	0,005102597	CACNA2D1	CACNA2D1	NM_000722	Body	
cg26442509	0,898332169	0,779515693	-0,118816476	0,005102919	HRH4	HRH4;HRH4;HRH4	NM_021624;NM_001160166;NM_001143828	TSS1500;TSS1500;TSS1500	
cg21783672	0,832263375	0,71668381	-0,115579565	0,005105499					N_Shelf
cg19988204	0,087544663	0,192402726	0,104858063	0,00510721	MAPK15	MAPK15	NM_139021	TSS200	N_Shore
cg07790870	0,210090852	0,329135065	0,119044213	0,005118767	CBFA2T2	CBFA2T2;CBFA2T2;CBFA2T2;CBFA2T2;CBFA2T2	NM_001032999;NM_005093;NM_001039709;NM_001039709;N	Body;5UTR;5UTR;1stExon;1st	
cg10938899	0,920540074	0,790654879	-0,129885194	0,005121456	GTF2F2	GTF2F2	NM_004128	Body	
cg17964016	0,880595154	0,735611637	-0,144983517	0,005122003	DEPDC6	DEPDC6	NM_022783	3UTR	
cg18716503	0,923406987	0,809587393	-0,113819594	0,005131378	ANO1	ANO1;ANO1	NM_018043;NR_030691	Body;Body	
cg23517954	0,932042142	0,794596748	-0,137445394	0,005134416	PSMD14	PSMD14	NM_005805	5UTR	S_Shelf
cg27388036	0,874035542	0,757129817	-0,116905726	0,005152142					
cg05196487	0,682491493	0,794894914	0,11240342	0,005153586	KIF13B	KIF13B	NM_015254	Body	Island
cg00598125	0,685638539	0,582603707	-0,103034832	0,005172792	HLA-DRB1	HLA-DRB1	NM_002124	Body	S_Shelf
cg25032321	0,373311526	0,483956918	0,110645393	0,005176545	STK24	STK24	NM_001032296	Body	N_Shore
cg05592255	0,870060961	0,742989945	-0,127071015	0,005176668	CYP3A43	CYP3A43;CYP3A43;CYP3A43	NM_022820;NM_057096;NM_057095	Body;Body;Body	
cg05493407	0,933451143	0,79383449	-0,139616653	0,005182404	mars-01	MARCH1	NM_001166373	5UTR	
cg07486669	0,882685386	0,769211903	-0,113473483	0,005187188	FLI1	FLI1;FLI1	NM_001167681;NM_002017	3UTR;3UTR	S_Shore
cg00997931	0,913652804	0,754753485	-0,15889932	0,005188323	YOD1	YOD1	NM_018566	3UTR	N_Shelf
cg11777515	0,083928053	0,184034967	0,100106914	0,005189185	PHOX2B	PHOX2B	NM_003924	TSS1500	Island
cg19351533	0,880900457	0,73515403	-0,145746427	0,005194512	C12orf76	C12orf76	NM_207435	TSS1500	
cg23074762	0,864414261	0,759101428	-0,105312833	0,005195588	CHSY1	CHSY1	NM_014918	Body	
cg24490154	0,846436925	0,689506894	-0,15693003	0,005195676	BCORL1	BCORL1	NM_021946	5UTR	S_Shelf
cg11249120	0,944336843	0,818800136	-0,125536707	0,005197931	SAMD3	SAMD3;SAMD3	NM_001017373;NM_001017373	5UTR;1stExon	
cg08319019	0,190823005	0,329683478	0,138860472	0,005198512	IKZF2	IKZF2	NM_001079526	TSS1500	Island
cg00589541	0,227442936	0,342101686	0,11465875	0,005199748	FNDC3B	FNDC3B;FNDC3B	NM_001135095;NM_022763	Body;Body	
cg12456825	0,158159136	0,267425523	0,109266388	0,005207795	FBXO40	FBXO40	NM_016298	TSS200	
cg08948442	0,702927375	0,572837609	-0,130089765	0,005210332	MAN1C1	MAN1C1	NM_020379	Body	
cg19435731	0,920974679	0,788761139	-0,13221354	0,005210547					
cg01346915	0,816584576	0,690115845	-0,126468731	0,005215524	UBE2I	UBE2I;UBE2I;UBE2I;UBE2I	NM_194261;NM_003345;NM_194260;NM_194259	Body;Body;Body;Body	S_Shore
cg09578524	0,905059942	0,780147428	-0,124912514	0,005221274	BICC1	BICC1	NM_001080512	Body	
cg19223616	0,900970855	0,795652527	-0,105318328	0,005226844	CLCN5	CLCN5;CLCN5;CLCN5	NM_001127898;NM_001127899;NM_000084	Body;Body;5UTR	
cg25241102	0,200537075	0,311184168	0,110647094	0,005227551	HNMT	HNMT;HNMT;HNMT;HNMT;HNMT;HNMT	NM_001024075;NM_006895;NM_001024074;NM_001024075;N	1stExon;1stExon;1stExon;5U	

cg17056747	0,703877463	0,561553723	-0,14232374	0,00523344					S_Shore
cg09127592	0,878272983	0,749064384	-0,1292086	0,00524655	TRIM35	TRIM35	NM_171982	Body	N_Shelf
cg15582138	0,895568475	0,791189242	-0,104379234	0,00525815					
cg17277952	0,924532968	0,783999693	-0,140533275	0,005260737	CCDC109A	CCDC109A	NM_138357	Body	
cg11343657	0,680904662	0,781005019	0,100100357	0,00526146					
cg03044684	0,152898808	0,254487261	0,101588453	0,005266767	HUNK	HUNK	NM_014586	Body	S_Shore
cg10585180	0,863372232	0,698641216	-0,164731015	0,005268724	BAX	BAX;BAX;BAX;BAX;BAX	NM_138763;NM_138764;NM_138761;NM_004324;NR_027882	Body;Body;Body;Body;Body	S_Shelf
cg01921059	0,043667426	0,150913713	0,107246288	0,005270681	MET	MET;MET	NM_000245;NM_001127500	5UTR;5UTR	
cg25999375	0,94076531	0,81062321	-0,130142099	0,005273675	DMD	DMD;DMD;DMD;DMD;DMD;DMD;DMD;DMD;DMD;DMD;DMD;DMD;DMD	NM_004013;NM_004023;NM_004014;NM_004009;NM_004007;N	Body;Body;Body;Body;Body;B	S_Shelf
cg03475756	0,918354791	0,79171421	-0,12664058	0,00528801	COL23A1	COL23A1	NM_173465	Body	N_Shore
cg19026859	0,906419146	0,76893364	-0,137485507	0,005289143					N_Shore
cg00701918	0,861595821	0,713374547	-0,148221274	0,005289573	RPTOR	RPTOR;RPTOR	NM_001163034;NM_020761	Body;Body	
cg16261737	0,101733073	0,206580257	0,104847184	0,00528965	FN1	FN1;FN1;FN1;FN1;FN1;FN1;FN1	NM_212482;NM_002026;NM_212474;NM_212476;NM_212478;N	TSS1500;TSS1500;TSS1500	S_Shore
cg25408575	0,914435246	0,741664293	-0,172770953	0,005291688					
cg06221218	0,082477622	0,191615545	0,109137923	0,005300931	PARS2	PARS2	NM_152268	TSS1500	S_Shore
cg15241130	0,92333531	0,806687358	-0,116647953	0,005319996	MTHFD1L	MTHFD1L	NM_015440	Body	
cg13801307	0,853189174	0,645373341	-0,207815833	0,00532136	DIP2C	DIP2C	NM_014974	Body	Island
cg14507792	0,878599572	0,756508056	-0,122091515	0,005324116	ANKRD19	ANKRD19	NR_026868	Body	S_Shelf
cg00639361	0,935624715	0,828029319	-0,107595396	0,005328048	ZFP64	ZFP64;ZFP64;ZFP64;ZFP64	NM_199426;NM_018197;NM_022088;NM_199427	Body;Body;Body;Body	S_Shelf
cg04876127	0,82285598	0,716954718	-0,105901262	0,005330347	TRIM62	TRIM62	NM_018207	Body	
cg22830701	0,102982749	0,224539634	0,121556886	0,005335973	BDNFOS	BDNFOS;LIN7C	NR_002832;NM_018362	TSS1500;Body	Island
cg22714601	0,929811171	0,777896169	-0,151915003	0,005342588	RUFY3	RUFY3	NM_001037442	3UTR	
cg07963741	0,75242896	0,648648554	-0,103780406	0,0053432					
cg22549437	0,910926198	0,800574725	-0,110351472	0,005343357	RNF144B	RNF144B	NM_182757	3UTR	
cg27408285	0,121308756	0,252106071	0,130797315	0,005348585	CBX5	CBX5;CBX5;CBX5;CBX5	NM_001127321;NM_001127322;NM_001127321;NM_012117	1stExon;TSS200;5UTR;5UTR	
cg05045498	0,793487045	0,684812616	-0,108674429	0,005348609					N_Shore
cg25830692	0,913105807	0,753363121	-0,159742686	0,005350214	ASRGL1	ASRGL1;ASRGL1	NM_001083926;NM_025080	Body;Body	
cg03554199	0,937275262	0,825469994	-0,111805268	0,00536398					N_Shelf
cg12728758	0,939421783	0,822662044	-0,116759739	0,005371345					
cg12944944	0,913809331	0,810649425	-0,103159906	0,00537589	APAF1	APAF1;APAF1;APAF1;APAF1;APAF1	NM_181869;NM_181861;NM_013229;NM_181868;NM_001160	Body;Body;Body;Body;Body	
cg14282126	0,903379919	0,791854744	-0,111525175	0,005376228	MRPL42	MRPL42;MRPL42;MRPL42	NM_172178;NM_014050;NM_172177	TSS1500;TSS1500;TSS1500	N_Shore
cg11404296	0,856263754	0,753682048	-0,102581706	0,005421171					
cg08317738	0,543639268	0,648773654	0,105134386	0,00542742	CBFA2T3	CBFA2T3	NM_005187	Body	Island
cg18489034	0,671741461	0,561617509	-0,110123951	0,00542952					
cg11394611	0,90773145	0,766625611	-0,14110584	0,005433479	RAB4A	RAB4A	NM_004578	Body	
cg06115679	0,589495712	0,475910045	-0,113585667	0,005439204	MACF1	MACF1	NM_012090	Body	
cg21446343	0,187606732	0,295162695	0,107555963	0,005439831	ARVCF	ARVCF	NM_001670	Body	S_Shore
cg00512872	0,290563019	0,438514	0,147950981	0,005448705	CYTH3	CYTH3	NM_004227	Body	
cg04339360	0,929480478	0,754844163	-0,174636315	0,005450284	KLF5	KLF5	NM_001730	Body	S_Shore
cg06006512	0,940300102	0,812857318	-0,127442784	0,005455804	EPHA3	EPHA3;EPHA3	NM_182644;NM_005233	Body;Body	
cg13651986	0,160873669	0,269184042	0,108310373	0,00545681	WIPF1	WIPF1	NM_001077269	TSS1500	S_Shore
cg22233170	0,926937784	0,825824831	-0,101112953	0,005458206	SCAPER	SCAPER;SCAPER	NM_001145923;NM_020843	5UTR;Body	
cg26879084	0,927625963	0,826824163	-0,100801799	0,005458738					
cg27047316	0,881189258	0,77917531	-0,102013948	0,005459688					S_Shelf
cg27461259	0,3030531	0,418677114	0,115624014	0,005461876	PPARGC1A	PPARGC1A	NM_013261	TSS1500	
cg09518245	0,900750338	0,786408696	-0,114341642	0,005476014	STMN1	STMN1;STMN1;STMN1;STMN1	NM_005563;NM_203399;NM_203401;NM_001145454	TSS1500;TSS1500;TSS1500	S_Shore
cg14523095	0,930286022	0,827731804	-0,102554218	0,00547631	GCLC	GCLC	NM_001498	Body	
cg13363124	0,845232864	0,744673051	-0,100559813	0,005486476	ARHGAP8	ARHGAP8;PRR5-ARHGAP8;ARHGAP8	NM_181335;NM_181334;NM_001017526	5UTR;Body;5UTR	Island
cg23569941	0,798794363	0,640318657	-0,158475706	0,005515526	TMBIM1	TMBIM1;PNKD	NM_022152;NM_015488	3UTR;Body	S_Shelf
cg01194073	0,279499313	0,171846189	-0,107653125	0,005515604					Island
cg11022060	0,222757013	0,341199307	0,118442294	0,005526699	CPEB1	CPEB1	NM_030594	Body	
cg26057840	0,692624789	0,565843079	-0,12678171	0,00554042					N_Shore
cg18544450	0,777102877	0,578005623	-0,199097254	0,005545629	LPHN1	LPHN1;LPHN1	NM_014921;NM_001008701	Body;Body	S_Shelf
cg11568379	0,902353807	0,78164309	-0,120710717	0,005545901	TM4SF4	TM4SF4	NM_004617	TSS1500	
cg01872077	0,151567034	0,254956868	0,103389834	0,005566563	CYP27A1	CYP27A1	NM_000784	TSS200	N_Shore
cg02396914	0,798266578	0,619117799	-0,179148779	0,00557149	TAF2	TAF2	NM_003184	3UTR	
cg05258481	0,948232064	0,828464016	-0,119768048	0,005576506	RBMS3	RBMS3;RBMS3;RBMS3	NM_001003792;NM_001003793;NM_014483	Body;Body;Body	
cg18202167	0,146407922	0,292528772	0,14612085	0,005588073	OS9	OS9;OS9;OS9;OS9	NM_001017958;NM_001017957;NM_001017956;NM_006812	TSS200;TSS200;TSS200;TSS	Island
cg18501142	0,122033311	0,246623627	0,124590316	0,00560684	MALAT1	MALAT1	NR_002819	Body	S_Shore
cg25984400	0,122351002	0,245238454	0,122887451	0,005613872					S_Shore
cg22175006	0,108790274	0,21464475	0,105854476	0,005615178	ACSL3	ACSL3;ACSL3	NM_203372;NM_004457	5UTR;5UTR	
cg00589791	0,541330181	0,653658332	0,112328151	0,005616395					
cg04008905	0,931340433	0,826673005	-0,104667428	0,005623746	GPR126	GPR126;GPR126;GPR126;GPR126	NM_020455;NM_001032394;NM_198569;NM_001032395	Body;Body;Body;Body	
cg00191102	0,903168338	0,792704763	-0,110463575	0,005633274					S_Shore
cg26365668	0,629905666	0,77171631	0,141810644	0,005636203	MRPS18C	MRPS18C;HELQ	NM_016067;NM_133636	TSS1500;Body	N_Shore
cg14345213	0,92785137	0,760096089	-0,167755281	0,005644718	TDRD7	TDRD7	NM_014290	5UTR	S_Shore
cg10674438	0,794015175	0,656678727	-0,137336449	0,005649211	LOC152024	LOC152024	NR_026834	TSS1500	
cg02756254	0,826824411	0,714423795	-0,112400616	0,005661783					N_Shore
cg21243154	0,938604141	0,795374803	-0,143229337	0,005664484	TRIM27	TRIM27	NM_006510	Body	
cg21856234	0,152481804	0,254578047	0,102096244	0,005665484					Island
cg00221736	0,91455287	0,808081257	-0,106471613	0,005669478	DMTF1	DMTF1;DMTF1;DMTF1;DMTF1;DMTF1	NM_001142326;NM_021145;NM_001142327;NR_024550;NR_02	Body;Body;Body;Body;Body	
cg16337108	0,914193631	0,801730661	-0,11246297	0,00568587	GCNT2	GCNT2;GCNT2;GCNT2	NM_145649;NM_001491;NM_145655	Body;Body;TSS1500	
cg16781964	0,89414162	0,692099664	-0,202041955	0,00569905	LHFPL1	LHFPL1	NM_178175	TSS1500	
cg27391921	0,932483325	0,794028188	-0,138455137	0,005720258	ACCN1	ACCN1;ACCN1	NM_001094;NM_183377	3UTR;3UTR	
cg17441280	0,077349672	0,193184359	0,115834687	0,005727392	MED20	MED20;BYSL	NM_004275;NM_004053	Body;TSS1500	N_Shore
cg18887458	0,498898144	0,603864694	0,10496655	0,005737125					
cg21117559	0,686113762	0,526874039	-0,159239724	0,005744131	STARD13	STARD13;STARD13;STARD13	NM_052851;NM_178007;NM_178006	5UTR;Body;Body	
cg03128163	0,876077942	0,767496691	-0,108581251	0,005757742	EBF3	EBF3	NM_001005463	Body	N_Shore
cg20691612	0,956307194	0,841049393	-0,115257802	0,005765102	MAST4	MAST4;MAST4	NM_015183;NM_001164664	Body;Body	
cg23193497	0,208127131	0,339850473	0,131723342	0,005767507	PDE10A	PDE10A;PDE10A	NM_001130690;NM_006661	Body;5UTR	
cg06371658	0,885853943	0,771541717	-0,114312227	0,005784945	TATDN2	TATDN2	NM_014760	Body	S_Shore
cg21061553	0,860629154	0,702147031	-0,158482123	0,005786749	SNRPN	SNRPN;SNRPN;SNRPN;SNRPN	NM_022805;NM_022808;NM_022807;NM_022806	5UTR;5UTR;5UTR;5UTR	S_Shore
cg00759894	0,914628116	0,804822499	-0,109805617	0,005819968					N_Shore
cg15836199	0,809029899	0,606910736	-0,202119163	0,005829987	MORN1	MORN1	NM_024848	Body	N_Shelf
cg15328758	0,909808354	0,777793569	-0,132014784	0,005842808					S_Shelf
cg08570346	0,856797071	0,753213869	-0,103583202	0,005843944					
cg17711595	0,807974106	0,654077974	-0,153896132	0,005845039	ZNF239	ZNF239;ZNF239;ZNF239	NM_001099284;NM_001099282;NM_001099283	5UTR;5UTR;5UTR	N_Shore
cg11965966	0,855843563	0,740717159	-0,115126403	0,005850672	PLOD1	PLOD1	NM_000302	Body	S_Shelf
cg14586993	0,884311246	0,763356837	-0,120954409	0,005854949					
cg15384640	0,827774829	0,725996241	-0,101778588	0,005857268					N_Shore
cg24360745	0,94552921	0,790472124	-0,155057086	0,00586625	PUS7	PUS7	NM_019042	Body	
cg11527839	0,946413731	0,786065837	-0,160347893	0,005867981					
cg27267820	0,903995143	0,794099415	-0,109895728	0,005868015					
cg06551007	0,934915682	0,828094843	-0,106820839	0,005880967					
cg23479988	0,935598838	0,827634662	-0,107964177	0,005889274	ABCB1	ABCB1	NM_000927	Body	N_Shelf
cg14141514	0,588600519	0,697500582	0,108900063	0,005895016	PIP	PIP	NM_002652	TSS200	
cg13583272	0,664546144	0,771860992	0,107314848	0,005904355	NXN	NXN	NM_022463	Body	Island
cg04766314	0,936996975	0,794196601	-0,142800374	0,005913567	PCCA	PCCA;PCCA	NM_001127692;NM_000282	Body;Body	
cg21280014	0,133582225	0,27505079	0,141468564	0,00591449	PTPRN2	PTPRN2;PTPRN2;PTPRN2	NM_002847;NM_130842;NM_130843	Body;Body;Body	N_Shelf
cg04142413	0,074661642	0,184026401	0,109364759	0,005914641	MRPS35	MRPS35	NM_021821	TSS1500	N_Shore
cg01526553	0,13394499	0,25480477	0,120859781	0,005915018	MYL6	MYL6;MYL6	NM_079423;NM_021019	Body;Body	
cg06111799	0,195960861	0,315610378	0,119649518	0,005917556	IFT57	IFT57	NM_018010	TSS200	Island
cg19028997	0,897592308	0,780489189	-0,117103119	0,005920854	ELK4	ELK4;ELK4	NM_001973;NM_021795	TSS1500;TSS1500	S_Shore
cg12345953	0,91242118	0,809329064	-0,103092117	0,005929464	PPP3CA	PPP3CA;PPP3CA;PPP3CA	NM_001130691;NM_000944;NM_001130692	Body;Body;Body	
cg26811868	0,902870393	0,773258658	-0,129611735	0,005931587					
cg14174583	0,777571805	0,600189175	-0,17738263	0,00594988	SNTG2	SNTG2	NM_018968	Body	N_Shore
cg02093918	0,714634716	0,566513401	-0,148121315	0,00597247	ATG7	ATG7;ATG7;ATG7	NM_001136031;NM_001144912;NM_006395	Body;Body;Body	
cg16202600	0,932405588	0,830754491	-0,101651098	0,005976791	SERPINE2	SERPINE2;SERPINE2;SERPINE2;SERPINE2	NM_006216;NM_001136530;NM_001136529;NM_001136528	5UTR;Body;5UTR;5UTR	
cg20278154	0,866798486	0,716321834	-0,150476652	0,0059854	CHST11	CHST11	NM_018413	Body	
cg15947466	0,82820026	0,696163853	-0,132036407	0,005987018	C6orf89	C6orf89	NM_152734	TSS1500	N_Shore
cg17604181	0,939892764	0,822133363	-0,117759401	0,005993044	SNORA47	SNORA47;ZBED3	NR_003014;NM_032367	TSS1500;5UTR	S_Shelf
cg11742202	0,642519957	0,757203792	0,114683835	0,005998148	SLC17A5	SLC17A5	NM_012434	TSS1500	S_Shore
cg11742479	0,807571338	0,650882715	-0,156688623	0,006003036	PABPC4	PABPC4;LOC728448;PABPC4;PABPC4	NM_003819;NR_003929;NM_001135654;NM_001135653	3UTR;TSS1500;3UTR;3UTR	
cg07200939	0,900611695	0,736087711	-0,164523984	0,006004413	PLXNC1	PLXNC1	NM_005761	3UTR	
cg03211192	0,216076563	0,334636783	0,11856022	0,006019288	CARD11	CARD11	NM_032415	5UTR	
cg10259761	0,881826965	0,775300325	-0,10652664	0,006023229	CCNY	CCNY;CCNY	NM_145012;NM_181698	Body;5UTR	
cg02077793	0,046049276	0,158659058	0,112609782	0,00602673	CARM1	CARM1	NM_199141	Body	Island
cg03224621	0,125637082	0,25644025	0,130803168	0,006026936					
cg16962838	0,658184535	0,764629838	0,106445304	0,006032357	BEND7	BEND7;BEND7	NM_152751;NM_001100912	Body;Body	Island
cg11107612	0,065402083	0,188702216	0,123300132	0,00603987					S_Shelf
cg03481274	0,112114693	0,238580988	0,126466296	0,006047569	RARB	RARB;RARB	NM_016152;NM_000965	TSS200;TSS200	
cg07925900	0,085380114	0,227786494	0,142406381	0,006048398	SNORD1C	SNORD1C	NR_004397	TSS200	S_Shore
cg08564527	0,130178158	0,247313102	0,117134944	0,006064426	FAM65A	FAM65A	NM_024519	5UTR	Island
cg01039849	0,921494771	0,815586065	-0,105908705	0,00607285	CAMK4	CAMK4	NM_001744	Body	
cg01419670	0,618674952	0,475229213	-0,143445739	0,006085479					N_Shore
cg16033723	0,184808317	0,297021274	0,112212957	0,006095937					
cg01921212	0,594862297	0,712242631	0,117380334	0,006102889	CST9L	CST9L	NM_080610	TSS1500	
cg26252151	0,939413667	0,832765247	-0,10664842	0,006107072	ASAM	ASAM	NM_024769	Body	
cg22530449	0,913522039	0,786165378	-0,127356661	0,006119731	VPS8	VPS8;VPS8	NM_015303;NM_001009921	Body;Body	
cg12367833	0,659802964	0,504559544	-0,15524342	0,006123662					
cg12254614	0,800602844	0,641786902	-0,158815942	0,006124406					
cg12708807	0,074961504	0,179263333	0,104301829	0,006124591	CRYZL1	CRYZL1	NM_145858	Body	
cg09438612	0,052297095	0,20027742	0,147980324	0,006126252	RDH10	RDH10;RPL7	NM_172037;NM_000971	TSS1500;TSS200	N_Shore
cg06870986	0,299310544	0,411645518	0,112334974	0,006131801					Island
cg00729656	0,916140451	0,806832889	-0,109307562	0,006136604	TRRAP	TRRAP	NM_003496	Body	N_Shore
cg12050497	0,218908401	0,33384592	0,114937519	0,00615037	FAM84A	FAM84A	NM_145175	5UTR	Island
cg24835233	0,649226016	0,750846569	0,101620554	0,006151982	PAGE3	PAGE3	NM_001017931	TSS1500	
cg26677546	0,929592712	0,80365532	-0,125937391	0,006154965	TRIM7	TRIM7;TRIM7;TRIM7;TRIM7;TRIM7	NM_203293;NM_203294;NM_033342;NM_203295;NM_203296	Body;5UTR;3UTR;5UTR;5UTR	N_Shore
cg10747185	0,922898726	0,81262329	-0,110275436	0,006167327	TBL1XR1	TBL1XR1	NM_024665	5UTR	
cg15844527	0,911535884	0,797056262	-0,114479622	0,006167854					S_Shelf
cg15666143	0,649720562	0,509942975	-0,139777587	0,006182972					N_Shelf
cg27197746	0,904914593	0,756017171	-0,148897422	0,00619168	ZNF121	ZNF121	NM_001008727	Body	
cg04721485	0,910968068	0,773622364	-0,137345704	0,00620045	WDR33	WDR33;WDR33	NM_018383;NM_001006623	Body;3UTR	
cg13406243	0,748356165	0,606673498	-0,141682667	0,006201158	CACNA1B	CACNA1B	NM_000718	Body	N_Shore
cg00959749	0,785681551	0,618505088	-0,167176462	0,006205481	TMEM48	TMEM48;TMEM48;TMEM48	NM_018087;NR_033142;NM_001168551	Body;Body;Body	N_Shore
cg06881572	0,878577558	0,709412293	-0,169165264	0,006215655					S_Shelf
cg09683440	0,04551515	0,154803958	0,109288808	0,006224155					
cg23804607	0,845277282	0,694299085	-0,150978198	0,006260048					
cg23236301	0,945735303	0,784000857	-0,161734446	0,006261102	BCAR3	BCAR3	NM_003567	TSS1500	S_Shore
cg01918001	0,908615461	0,786060841	-0,12255462	0,006262315					
cg10389936	0,801965389	0,677008743	-0,124956646	0,006264114					
cg08157323	0,771481719	0,626266675	-0,145215045	0,006264699	BRF1	BRF1;BRF1	NM_001519;NM_145685	Body;Body	S_Shelf
cg17783333	0,517229862	0,623513356	0,106283494	0,006270205					
cg00347643	0,179246733	0,299312136	0,120065403	0,00627454	YWHAG	YWHAG	NM_012479	3UTR	N_Shore
cg01182236	0,914685551	0,704912195	-0,209773356	0,006285378	C16orf45	C16orf45	NM_033201	Body	
cg21645572	0,82744	0,666266695	-0,161173304	0,006288789	DEPDC6	DEPDC6	NM_022783	Body	
cg06239143	0,899359106	0,764694344	-0,134664763	0,006288982	C8orf44	C8orf44;C8orf44	NM_019607;NM_019607	5UTR;1stExon	
cg19179687	0,900753528	0,689561029	-0,211192499	0,006294587					
cg16027727	0,915198272	0,803208704	-0,111989568	0,00629649	RPTOR	RPTOR;RPTOR	NM_001163034;NM_020761	Body;Body	
cg15350071	0,801430126	0,563778699	-0,237651426	0,006300553					Island
cg22655561	0,105589825	0,207477674	0,101887849	0,006301686	PRR7	PRR7	NM_030567	3UTR	S_Shore
cg01893030	0,853453305	0,742657681	-0,110795624	0,006308271					
cg19415852	0,962567222	0,838799946	-0,123767275	0,006311206	POLS	POLS	NM_006999	3UTR	S_Shore
cg03689540	0,836021835	0,714318439	-0,121703396	0,006328617	UXT	UXT;UXT	NM_153477;NM_004182	Body;Body	N_Shore
cg14583550	0,834393755	0,646611686	-0,187782069	0,006328967	FANCC	FANCC	NM_000136	5UTR	N_Shelf
cg07510346	0,757209692	0,859478917	0,102269225	0,006335364	MYOM2	MYOM2	NM_003970	Body	N_Shore
cg20363199	0,757168781	0,640368148	-0,116800633	0,00633547	SORCS1	SORCS1;SORCS1	NM_052918;NM_001013031	3UTR;3UTR	
cg09179485	0,105225726	0,248765335	0,143539609	0,006341437	C13orf23	C13orf23;C13orf23;NHLRC3;NHLRC3	NM_170719;NM_025138;NM_001017370;NM_001012754	TSS1500;TSS1500;Body;Bod	Island
cg11082691	0,12183563	0,256836584	0,135000953	0,006364097	TNFRSF19	TNFRSF19;TNFRSF19	NM_018647;NM_148957	Body;Body	
cg27276128	0,847071643	0,723767764	-0,123303879	0,006378162	ZNF528	ZNF528	NM_032423	5UTR	S_Shelf
cg00604188	0,855439194	0,738510209	-0,116928985	0,006384307	PPP2R2D	PPP2R2D;PPP2R2D;PPP2R2D	NR_033191;NM_001003656;NM_018461	Body;Body;Body	
cg04982344	0,137315915	0,242603086	0,10528717	0,006389233	IFT57	IFT57	NM_018010	TSS200	Island
cg16174859	0,740769368	0,849230268	0,1084609	0,006416908	F7	F7;F7	NM_000131;NM_019616	Body;Body	Island
